# Unified Synthesis of Unconventional α‐Polyhalogenated Amines Through Hydroaminoalkylation

**DOI:** 10.1002/anie.1233707

**Published:** 2026-07-10

**Authors:** Angela K. Hofmeister, Giulia Iannelli, Péter Angyal, Augustin Malandain, Daniel Kaiser, Boris Maryasin, Hanspeter Kählig, Matteo Barel, Gaia Novarino, Nuno Maulide

**Affiliations:** ^1^ Institute of Organic Chemistry University of Vienna Vienna Austria; ^2^ Institute of Science and Technology Austria Klosterneuburg Austria

**Keywords:** α‐halomethyl amines, amination, difluoromethyl, polyhalogenation, unactivated alkenes

## Abstract

We report a unified method for the synthesis of α‐polyhalomethyl amines from alkenes and alkynes, enabled by readily available hemiaminal reagents. This operationally simple transformation allows for the introduction of not only well‐established CF_3_ and CF_2_H groups, but also the synthetically (and medicinally) underexplored CF_2_Cl and CFClH motifs—thereby broadening access to previously inaccessible chemical space of halogenated amine scaffolds. The method displays broad substrate scope and functional‐group tolerance while operating under mild conditions. Late‐stage functionalization of drug‐like derivatives of Oxaprozin, Erlotinib, and Ibuprofen (among others) is reported.

## Graphical abstract

A unified, redox‐neutral method that transforms alkenes and alkynes into α‐polyhalomethyl amines using stable hemiaminal reagents is reported. This approach enables access to CF_3_, CF_2_H, CF_2_Cl, and CFClH motifs, expanding halogenated amine space with broad substrate scope, functional group tolerance, and applicability to late‐stage modification of drug‐like molecules.

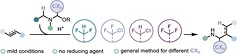

## 1 Introduction

Polyhalogenated compounds feature among pharmaceuticals, agrochemicals, and innovative materials [1, 2, 3, 4] and incorporating halogens, especially fluorine, into the structure of amines dramatically alters their physicochemical properties [5]. Fluorinated alkylamines show major changes in nitrogen basicity (p*K*_a_) [6] and lipophilicity, properties that are tightly linked to bioavailability and biological activity [7, 8]. Additionally, specific fluorination patterns can enhance drug potency and selectivity, while also improving metabolic stability and membrane permeability [9, 10, 11]

**SCHEME 1:**
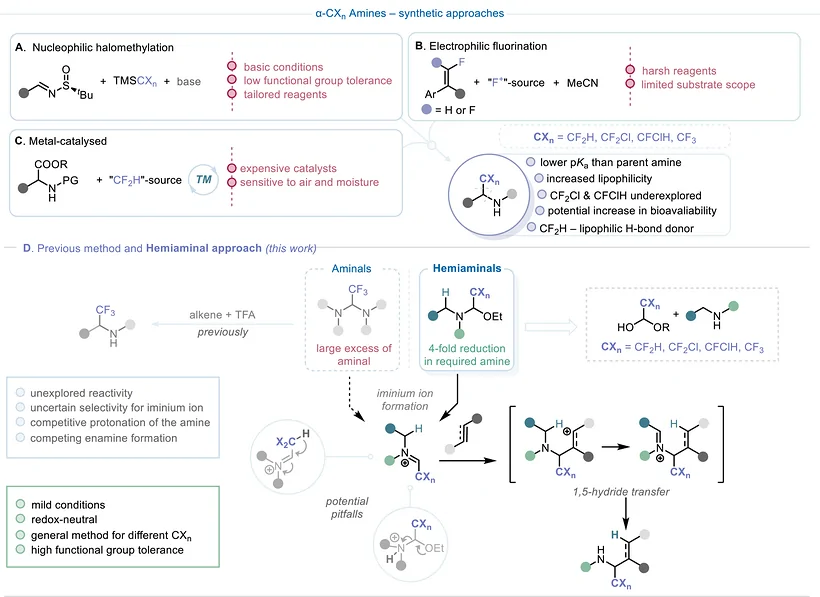
Overview of biologically relevant properties of α‐polyhaloalkylated amines. (A–C) Selected methods for the synthesis of α‐fluoroalkylated amines. (D) General synthesis of α‐halomethyl amines from alkenes and alkynes through the acid‐mediated activation of hemiaminals. TMS = trimethylsilyl; Ar = aryl; PG = protecting group; TM = transition metal; TFA = trifluoroacetic acid.

As a result of these properties of groups such as trifluoromethyl (CF_3_) or difluoromethyl (CF_2_H), fluorination has become an increasingly popular strategy for structure‐based drug development. Recently, the CF_2_H motif in particular has gained increased attention in medicinal chemistry due to its ability to participate in hydrogen bonding [12]. Its lipophilic hydrogen‐bond donor properties allow, for example, α‐difluoromethyl amines to serve as bioisosteric surrogates for β‐amino alcohols and thiols. Another halogen substituent frequently used in medicinal chemistry is chlorine [13, 14], which has been reported to increase dipole effects (with increases of bioactivity up to 100,000 fold) and modulate basicity (akin to fluorine) [15]. Despite these documented successes in drug molecules, only a handful of approaches to the synthesis of amines bearing polyhalogenated groups (CX_n_) have been reported, with the exception of the well‐explored CF_3_ pattern [16, 17]. Work by Hu and coworkers has focused on the development of stereoselective methods for the synthesis of α‐CF_2_H amines, including the combination of *N*‐(*tert*‐butylsulfinyl)imines with a difluoromethylation reagent (Scheme 1A) [18, 19, 20, 21], and reagent‐controlled addition to an imine facilitated by a chiral difluoromethyl sulfoximine (not shown) [22]. Analogous methods for chloromethyl groups have also been developed [23, 24]. While allowing the stereoselective synthesis of fluorinated or chlorinated amines, broader application is impacted by the need for tailored and costly reagents and strong bases. A different approach to the synthesis of α‐fluoromethylated amines can be found in the aminofluorination of fluorinated alkenes (Scheme 1B) [25]. This method employs electrophilic fluorinating agents, such as Selectfluor, with acetonitrile as a nitrogen source [26]. The reaction is applicable to a variety of fluorostyrenes, but encounters limitations due to the oxidative properties and generally poor chemoselectivity of Selectfluor. Conceptually related methods enable direct C–CF_2_H bond formation from alkenes or imines, typically employing CF_3_H [27], TMSCF_3_ [28], or a radical difluoromethyl source [29].

Recent advances in transition metal catalysis have enabled fluoromethylations through engagement of a carbon‐centered radical with a fluoromethyl‐metal complex [30, 31, 32]. One noteworthy example is work by Liu and coworkers, who reported a decarboxylative difluoromethylation of amino acid derivatives (Scheme 1C) [32]. Despite their synthetic utility, these methods remain limited by the cumbersome preparation of suitable halomethyl (CX_n_) sources and the necessity for amine protecting groups. In a recent development, a photochemical approach to the generation of α‐fluoroalkyl(R_F_)‐containing α‐amino radicals enabled the transformation of alkenes into α‐fluoroalkyl amines [33]. Even though this strategy allows the reliable preparation of CF_3_‐containing amines, the limitation of the applied C–R_F_ radicals lies in the strong differences of radical polarities induced by different halogenation patterns [34], resulting in decreased yields and limited scope for fluoroalkyl groups differing from CF_3_.

While CF_2_H and CF_3_ groups have garnered great attention for their biological relevance, the use of alternative halogenation patterns remains largely unexplored, primarily due to limited synthetic access. While the CF_2_Cl group is known to serve as a synthetic intermediate for difluoroalkylation reactions [35, 36, 37, 38, 39], incorporation of CF_2_Cl and CFClH motifs into drug‐like amines is rare [40]—mostly since methods for preparing α‐CF_2_Cl [41, 42, 43] and α‐CFClH [43] amines are scarce and typically rely on the presence of enolizable groups or deprotonation with strong bases. This lack of chemical methodology has hindered systematic studies of polyhalogenated amines.

To address this gap, we have developed a unified strategy for the synthesis of α‐polyhaloalkyl amines from unbiased alkenes and alkynes. Our approach relies on an unusual disconnection in which the CX_n_ group is introduced through hemiaminal reagents which enable acid‐mediated generation of the α‐substituted iminium ions under mild conditions. In the presence of alkenes or alkynes, these iminium ions can undergo addition by the π‐nucleophile, followed by a 1,5‐hydride transfer to afford the desired α‐polyhaloalkyl amines after hydrolysis.

However, the use of hemiaminals in this context is largely unexplored [44, 45] and poses several intrinsic challenges. These include (a) the possibility of preferential protonation at nitrogen leading to ultimate oxocarbenium ion formation, (b) competing enamine formation—particularly for CFXH (X = F or Cl) motifs—given the known failure of related aminal‐based systems derived from enolizable aldehydes. Finally, the anticipated application of a “one‐method‐fits‐all approach” for the incorporation of a range of both fluorine‐ and chlorine‐containing side chains was likely to be impeded by the different electronic properties resulting from changes in the halogenation patterns.

Herein, we describe a method that overcomes these potential limitations. We report a unified, redox‐neutral strategy enabling the synthesis of previously inaccessible α‐polyhalomethyl amines as a general platform to effectively expand the chemical space of polyhalogenated amines.

## 2 Results and Discussion

### 2.1 Optimization

Initial optimization aimed to establish whether hemiaminals could indeed serve as suitable nitrogen sources (Table 1, see the Supporting Information for a complete table of optimization). We were pleased to see that the treatment of 4‐phenyl‐1‐butene (**2**) with hemiaminal **1a** (4 equiv.) in trifluoroacetic acid at ambient temperature led to the formation of the desired product (**3f**) in 62% NMR yield (Entry 1). It is worthy of note that, while still used in excess, the mere use of a hemiaminal (in contrast to previously used aminals for related iminium‐mediated transformations) [44, 45] already de facto cuts the stoichiometry in half and doubles the atom economy of the reaction. We then found that increasing the temperature led to a significant drop in yield (Entry 2) and further investigation of different co‐solvent systems, particularly combinations of 1,2‐dichloroethane, acetonitrile, ethyl acetate, or hexafluoroisopropanol (HFIP) with trifluoroacetic acid, showed successful formation of desired product in all cases, although this was mostly accompanied by a slight loss of conversion (Entries 3–6). The positive effect of employing HFIP, known for its ability to stabilize cationic intermediates [46], is particularly noteworthy (Entry 6), as we found that first increased concentration (Entry 7) and then a shift to HFIP as the sole solvent with a stoichiometric amount of trifluoroacetic acid (TFA, Entry 8) gave excellent results (>80% NMR yield), while also allowing a decrease of the hemiaminal stoichiometry to half of the original amount.

**TABLE 1:** Optimization of reaction conditions using hemiaminal **1a** and 4‐phenylbutene (**2**).

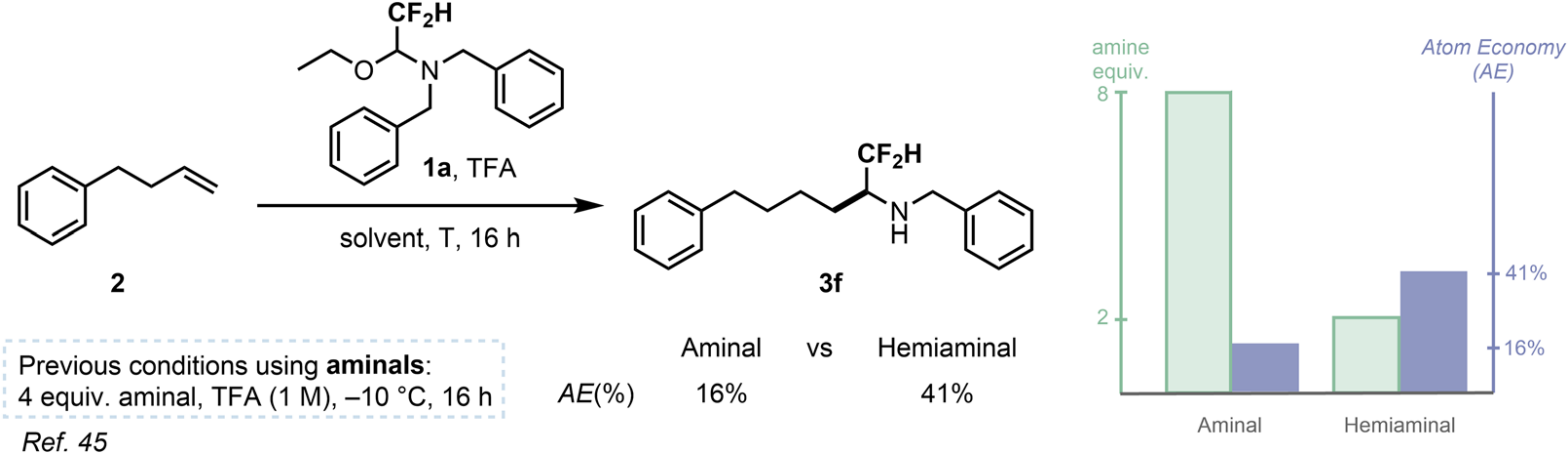				
Entry	Equiv. 1a	Solvent (conc.) and equiv. of TFA (if not used as solvent)	T [°C]	NMR Yield^a^
1	4	TFA (0.1 m)	25	62%
2	4	TFA (0.1 m)	50	20%–30%
3	4	EtOAc/TFA (1:1; 0.1 m)	25	15%
4	4	MeCN/TFA (1:1; 0.1 m)	25	30%
5	4	DCE/TFA (1:1; 0.1 m)	25	35%
6	4	HFIP/TFA (1:1; 0.1 m)	25	62%
7	4	HFIP/TFA (1:1; 0.6 m)	25	84%
**8**	**2**	**HFIP (0.6 M), TFA (2 equiv.)**	**25**	**80% (78%** ^b^)

^a^ Yield determined using 1,3,5‐trimethylbenzene as an internal standard.

^b^ isolated yield.

Atom Economy was calculated using the following equation: *AE* = (M_r_ desired product ÷ ΣM_r_ reactants) × 100. TFA = trifluoroacetic acid; DCE = 1,2‐dichloroethane; HFIP = hexafluoroisopropanol.

### 2.2 Scope of the Reaction

**SCHEME 2:**
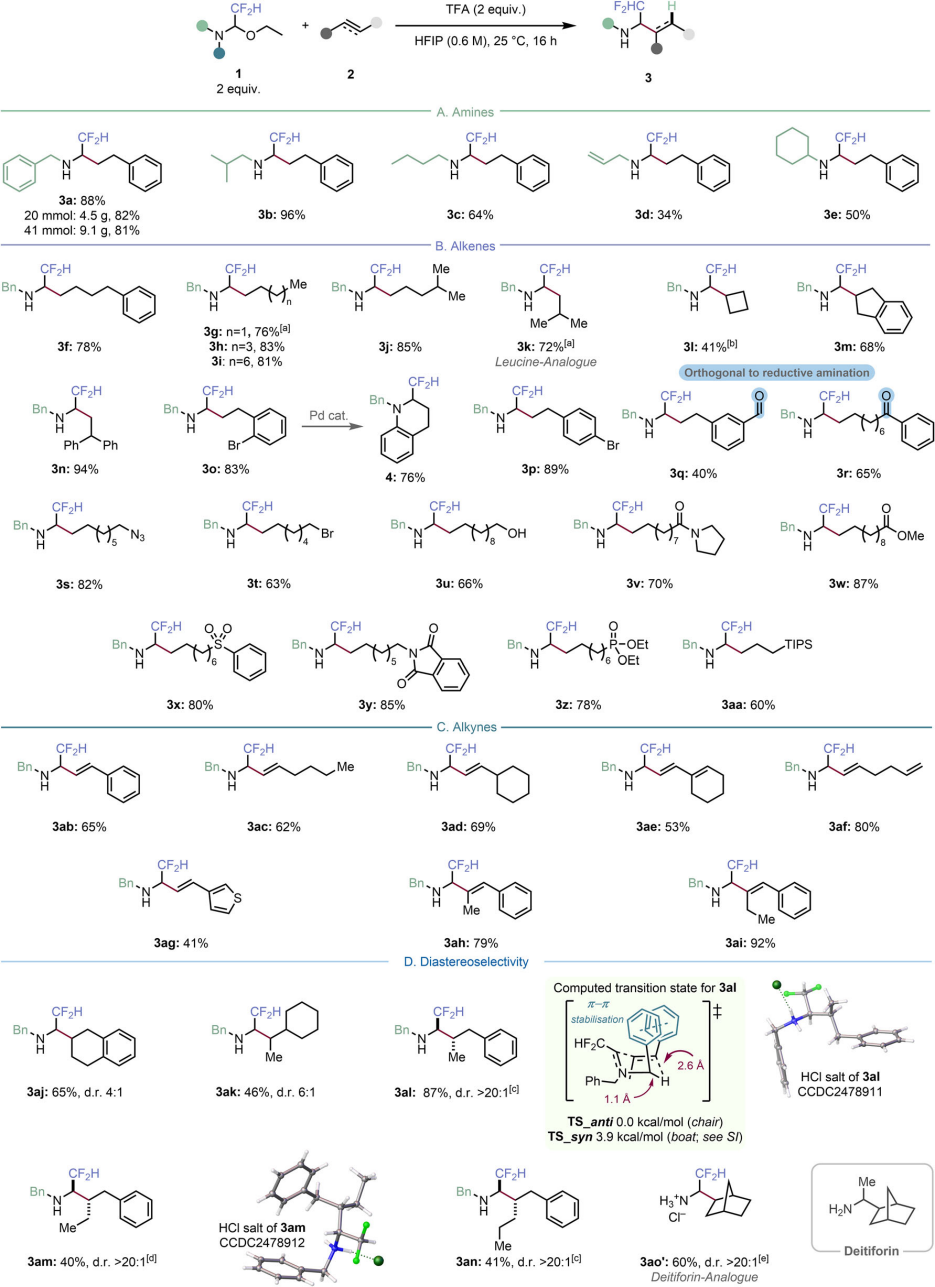
Reaction scope using the CF_2_H hemiaminal to transform alkenes and alkynes into α‐CF_2_H amines. (A) Scope of amines. (B) Scope of alkenes. (C) Scope of alkynes. (D) Examples of diastereoselective reactions. (a) Hexane was used as a co‐solvent; (b) DCM was used as a co‐solvent; (c) from *trans*‐configured alkene; (d) from *cis*‐*trans* mixture; (e) 1 equiv. of hemiaminal **1a** with 2 equiv. of alkene was used; removal of the benzyl group using H_2_, Pd/C afforded **3ao’** in quantitative yield. d.r. = diastereomeric ratio. Relative Gibbs free energy differences between two transition states ΔΔ*G* ^‡^_298, HFIP_ were computed at the B3LYP‐D3(BJ), SMD(HFIP)/def2‐TZVP//B3LYP‐D3(BJ)/def2‐SVP level of theory.

With optimized conditions in hand, the scope of this transformation was explored (Scheme 2) [47]. A range of substituted amine moieties was successfully installed, furnishing products including benzyl (**3a**), branched and linear alkyl (**3b** and **3c**, respectively), allyl (**3d**), and cyclohexyl amines (**3e**). Furthermore, the scalability of the developed reaction was exemplified by the synthesis of styrene‐derived amine **3a** to yield >9 g of product in a single pass (with a comparable yield of >80%). The reaction provided high yields with aliphatic alkenes, including those containing a remote phenyl group (**3f**), as well as varying chain lengths, regardless of branching (**3g–k**).

Owing to the volatility of 1‐butene, isobutene, and cyclobutene, these substrates were employed as commercial solutions (in hexane or dichloromethane), with no deleterious impact on product formation, with **3g**, **3k** and **3l** being formed in high yields even in the presence of these co‐solvents. Internal alkenes likewise undergo the transformation to afford the functionalized amines (**3l–m**). Subsequently, different styrene derivatives were investigated (**3n–q**) with excellent tolerance of a range of substituents on the aromatic ring, many of which could serve as synthetic handles for derivatization (**3o–q**). When bromoarene **3o** was exposed to the conditions of Buchwald–Hartwig coupling [48, 49], α‐difluoromethyl‐substituted tetrahydroisoquinoline **4** was readily obtained, highlighting the potential to access new derivatives of a commonly found scaffold in biology [50]. Importantly, 3‐vinylbenzaldehyde, a substrate bearing an additional aldehyde, afforded the desired product (**3q**) exclusively. Similarly, a ketone‐containing substrate furnished **3r** as the sole product. These examples highlight the complementarity of this protocol to reductive amination, the classical approach to the synthesis of secondary amines. Moreso, by relying exclusively on an internal redox event, this method circumvents the use of external reducing reagents [51]. Gratifyingly, the reaction exhibited further broad functional group tolerance, with alkenes bearing azide (**3s**), bromo (**3t**), alcohol (**3u**), amide (**3v**), ester (**3w**), sulfone (**3x**), phthalimide (**3y**), and phosphonate (**3z**) substituents (many of which incompatible with organometallics or reducing agents) giving the desired products in good yields throughout. The conditions of the transformation, furthermore allow the reaction with an allyl silane to afford product **3aa** without degradation of the TIPS group.

Importantly, we found that the acid‐mediated reaction of hemiaminals with terminal alkynes produced secondary allylic amines in good yields and, due to the *syn*‐nature of the hydride transfer, exclusively as the (*E*)‐stereoisomers (Scheme 2, B, **3ab–ag**). The reaction proceeded selectively, even in the presence of additional alkenes, as demonstrated by the formation of conjugated product **3ae** and compound **3af** from enyne precursors. Notably, the subjection of internal alkynes to the reaction conditions forged the corresponding trisubstituted alkene products, bearing an amine and a CF_2_H group in the allylic position, in high yields (**3ah** and **ai**).

When alkenes allowing the formation of an additional stereocentre were used, we were pleased to see that 1,2‐induction allowed moderate to good levels of diastereoselectivity for a range of both disubstituted and trisubstituted alkenes, affording structures **3aj** and **ak**. Some products, such as those obtained from β‐alkylated styrene derivatives, were even formed as single diastereomers (**3al–an**; see the transition state in Scheme 2 and the Supporting Information for further details) [52], as was amine **3ao’**, obtained from norbornene after debenzylation, which constitutes a fluorinated analogue of the anti‐viral amine Deitiforin [53].

These results demonstrate the versatility of this transformation, providing highly valuable α‐difluoromethylamines from abundant alkenes and providing a mechanistic framework that outcompetes enamine formation [54] and offering valuable insight in the context of hydride‐transfer‐mediated hydroaminoalkylation.

### 2.3 Expanding to Different Polyhalogenated Amines

**SCHEME 3:**
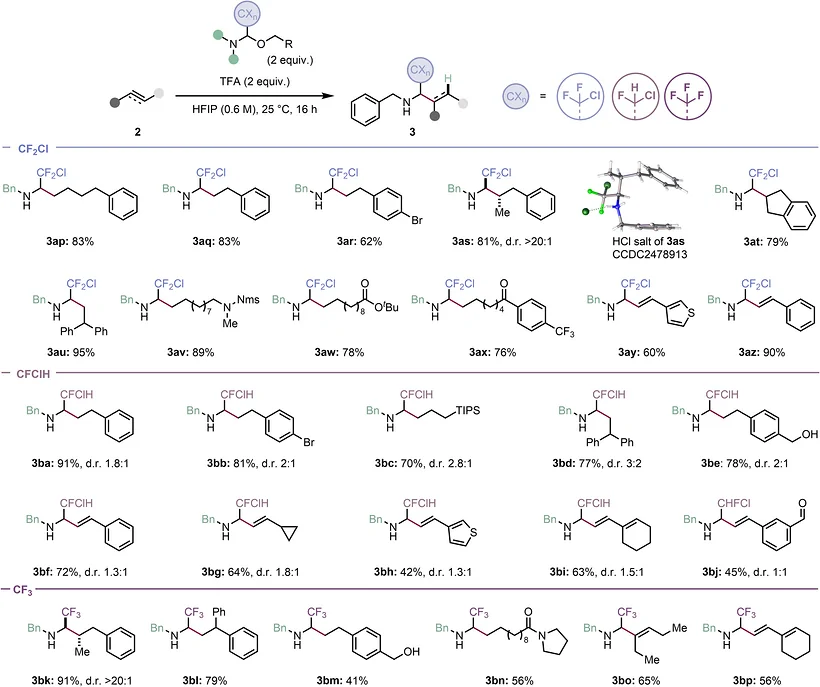
Demonstration of the general application of the reaction conditions to generate different polyhalogenated amines from alkenes and alkynes. Nms = nonafluoromesitylenesulfonyl [55].

To allow investigation of uncommon halogenation patterns, we started from inexpensive polyhalogenated acetates to synthesize a range of related hemiaminal reagents (in a simple and scalable transformation; see the Supporting Information for details) and subjected them to the previously developed conditions (Scheme 3). We were pleased to observe that the reactivity of the polyhalogenated hemiaminals was retained across a broad substrate scope, enabling functionalization of both alkenes and alkynes with diverse halogenation patterns, including CF_2_Cl (**3ap–az**), CFClH (**3ba–bj**), and also CF_3_ (**3bk–bp**). The reaction tolerated various groups such as sulfonamides (**3av**), esters (**3aw**), ketones (**3ax**), benzylic alcohols (**3be** and **3bm**), aldehydes (**3bj**), and amides (**3bn**). Two additional alkynes, including a cyclopropyl‐substituted alkyne (**3bg**) and an internal alkyne (**3bo**), were also successfully explored. Furthermore, consistent with the diastereoselectivity observed for CF_2_H and substituted alkenes, reactions with *trans*‐β‐methyl styrene also delivered **3as** and **3bl** as single diastereomers in high yields. It is worthy of note that the scarcity of approaches to prepare α‐CF_2_Cl and α‐CFClH amines makes this method a particularly valuable contribution to the synthetic toolbox.

### 2.4 Complex Substrates and Application

**SCHEME 4:**
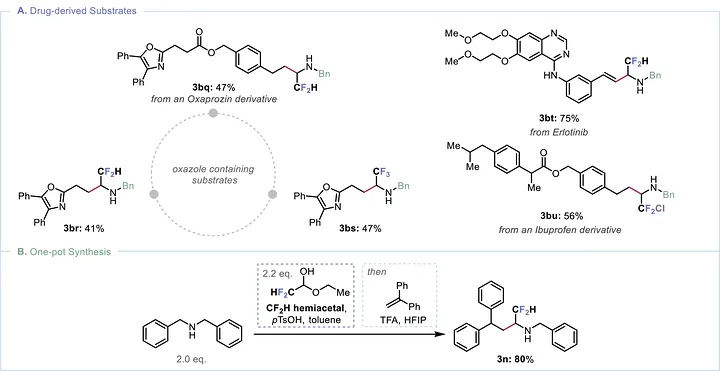
(A) Selected examples of pharmaceutical‐derived amine functionalization. (B) One‐pot procedure for the synthesis of an α‐difluoromethylated amine from an amine.

Methods enabling the synthesis of derivatives of known bioactive agents, as well as the functionalization of complex substrates are in high demand. Initially (Scheme 4), we focused on applying our protocol to the synthesis of derivatives of the non‐steroidal anti‐inflammatory drug Oxaprozin. Pleasingly, in addition to a derivative of Oxaprozin (**3bq**), compounds bearing structural resemblance through an oxazole ring (**3br–3bs**) were also readily obtained, showcasing that our method tolerates moieties prevalent in drug molecules. Notably, the structurally more complex Erlotinib also underwent efficient transformation to its difluoromethylated derivative (**3bt**). Installation of the CF_2_Cl group to an Ibuprofen‐derived alkene also gave the polyhalogenated product in good yield (**3bu**). These examples underscore the versatility of the method in diverse steric and electronic environments. Additionally, the practicality of the method was highlighted by a one‐pot synthesis of **3n** from the commercially available hemiacetal, serving as a CF_2_H synthon. This approach furthermore enhanced the overall efficiency of the conversion, resulting in an 80% overall yield. In contrast, the corresponding stepwise reaction afforded only 61% yield over two steps (largely due to the instability of the hemiaminal during purification on silica gel), underscoring a clear advantage of the one‐pot procedure. This improvement not only conserves valuable resources, but also delivers a compelling message for future synthetic applications.

## 3 Conclusion

We have established a unified, redox‐neutral method for the synthesis of α‐polyhalomethyl amines from alkenes and alkynes using bench‐stable hemiaminal reagents. This operationally simple transformation enables the introduction of not only well‐established CF_3_ and CF_2_H groups, but also the synthetically underexplored CF_2_Cl and CFClH motifs—thereby significantly expanding the accessible chemical space of halogenated amine scaffolds. Notably, the method achieves this with improved stoichiometric efficiency by maximizing the use of amine equivalents, achieving a four‐fold reduction in comparison to previously established hydroaminoalkylations of this kind. The method's successful application to the late‐stage functionalization of drug‐like molecules such as derivatives of Oxaprozin, Erlotinib, and Ibuprofen, as well as its diastereoselectivity and scalability collectively underscore its utility. By enabling modular access to a family of polyhalogenated amines, this methodology is poised to not only facilitate or accelerate structure–activity relationship (SAR) studies in medicinal chemistry, but also to stimulate further investigations of the physicochemical and biological consequences of these underexplored halogenation patterns. In this light, we envision the reaction presented here as a valuable tool for the rational design of next‐generation fluorinated and chlorinated bioactive molecules.

## Author Contributions

**Angela K. Hofmeister**: investigation, writing – review and editing, and writing – original draft. **Giulia Iannelli**: investigation, writing – review and editing, and writing – original draft. **Péter Angyal**: investigation and writing – review and editing. **Augustin Malandain**: investigation and writing – review and editing. **Daniel Kaiser**: supervision, writing – review and editing, and project administration. **Boris Maryasin**: investigation, writing – review and editing, and methodology. **Hanspeter Kählig**: methodology and data curation. **Matteo Barel**: investigation and methodology. **Gaia Novarino**: investigation and methodology. **Nuno Maulide**: conceptualization, funding acquisition, writing – review and editing, and project administration.

## Conflicts of Interest

None of the authors have conflicts of interest to disclose.

## Supporting information




**Supporting File**: The authors have cited additional references within the Supporting Information [56, 57, 58, 59, 60, 61, 62, 63, 64, 65, 66, 67, 68, 69, 70, 71, 72, 73, 74, 75, 76, 77, 78, 79, 80, 81].

## Data Availability

The data that supports the findings of this study are available in the supplementary material of this article.
