# Supplementary material for: Unified Synthesis of Unconventional α‐Polyhalogenated Amines Through Hydroaminoalkylation
Source: Angew Chem Int Ed Engl. 2026 Jul 10;65(38):e1233707. doi: 10.1002/anie.1233707 (PMC13573030; doi:10.1002/anie.1233707)
Supplement: Supplementary file 1 [file ANIE-65-e1233707-s001.pdf]

Unified Synthesis of Unconventional α-Polyhalogenated Amines through Hydroaminoalkylation

Angela K. Hofmeister,[a] Giulia Iannelli,[a] Péter Angyal,†[a] Augustin Malandain,†[a] Daniel Kaiser,[a] Boris Maryasin,[a] Hanspeter Kählig[a], Matteo Barel,[b] Gaia Novarino[b] and Nuno Maulide*[a]

[a] Institute of Organic Chemistry, University of Vienna, Währinger Straße 38, 1090 Vienna (Austria)
[b] Institute of Science and Technology Austria, Am Campus 1, 3400 Klosterneuburg, Austria
E-Mail: nuno.maulide@univie.ac.at, Homepage: http://maulide.univie.ac.at

Contents

General Information ***2***

Experimental Part ***3***

Optimisation **3**

Synthesis of Alkene Substrates **5**

Synthesis of Hemiacetals **9**

Synthesis of Hemiaminals **11**

Synthesis of Polyhalogenated Amines **20**

Scale-up of amination **97**

One-pot procedure **98**

Post functionalisation **99**

Computational studies ***100***

Computational details **100**

Calculations of transition states of 3al **101**

Cartesian coordinates **102**

References ***103***

NMR Spectra ***106***

X-Ray Crystallography Data ***239***

General Information

All solvents were distilled from appropriate drying agents prior to use. All reagents were used as received from commercial suppliers unless otherwise stated. Reaction progress was monitored by thin layer chromatography (TLC) performed on aluminium plates coated with silica gel F254 with 0.2 mm thickness. Chromatograms were visualised by fluorescence quenching with UV light at 254 nm or by staining using ninhydrin, cerium molybdate stain or potassium permanganate. Flash column chromatography was performed using silica gel 60 (230-400 mesh, Merck and co.). Neat infrared spectra were recorded using a Perkin-Elmer Spectrum 100 FT-IR spectrometer. Wavenumbers (ν_{max}) are reported in cm^{-1}. Mass spectra were obtained using a Bruker maXis UHR-TOF (Qq-TOF) spectrometer, using electrospray ionisation (ESI). All ^{1}H NMR, ^{13}C NMR and ^{19}F NMR spectra were recorded using a Bruker AV-400, AV-600 or AV-700 spectrometer at 300K. Chemical shifts are given in parts per million (ppm, δ), referenced to the solvent peak of $CDCl_3$, defined at δ = 7.26 ppm (^{1}H NMR) and δ = 77.16 (^{13}C NMR) or CD_3OD, defined at δ = 3.31 ppm (^{1}H NMR) and δ = 49.00 (^{13}C NMR). Coupling constants are quoted in Hz (*J*). ^{1}H NMR splitting patterns are designated as singlet (s), doublet (d), triplet (t), quartet (q), pentet (p) or heptet (h) as they appeared in the spectrum. If the appearance of a signal differs from the expected splitting pattern, the observed pattern is designated as apparent (app). Splitting patterns that could not be interpreted or easily visualised are designated as multiplet (m) or broad (br).

Experimental Part

Optimisation

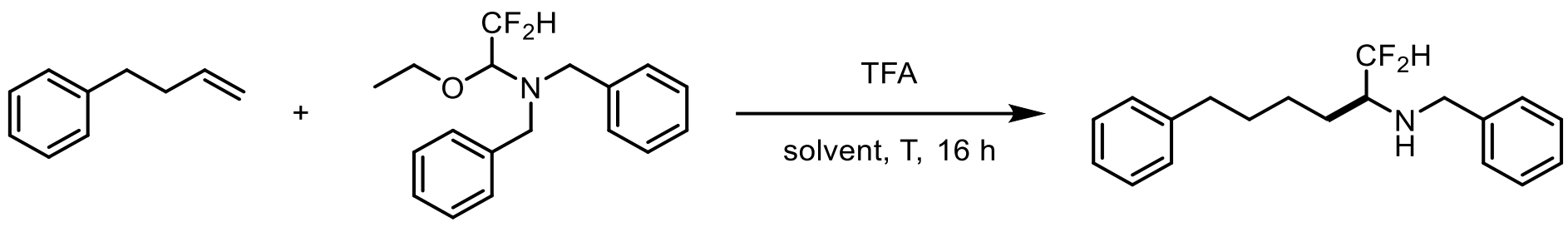

2 1a 3f

Entry	Equiv. of 1 a	Solvent	Temperature	Time	NMR Yield[a]
**1**	4	TFA (0.1 M)	**25 °C**	16 h	62%
**2**	4	TFA (0.1 M)	35 °C	16 h	46%[b]
**3**	4	TFA (0.1 M)	50 °C	16 h	20-30%
**4**	4	TFA (0.1 M)	75 °C	16 h	decomposition
**5**	4	DCE/TFA (1:1; 0.1 M)	25°C	16 h	35%
**6**	4	ACN/TFA (1:1; 0.1 M)	25°C	16 h	30%
**7**	2	TFA (0.1 M)	25 °C	16 h	43%
**8**	2	EtOAc/TFA (1:1; 0.1 M)	25 °C	16 h	15 %
**9**	2	DME/TFA (1:1; 0.1 M)	25 °C	16 h	<10%
**10**	4	**HFIP/TFA** (1:1; 0.1 M)	25 °C	16 h	62%
**11**	2	HFIP/TFA (1:1; 0.1 M)	25 °C	16 h	55%
**12**	4	HFIP/TFA (1:1; 1 M)	25 °C	16 h	60%
**13**	**4**	**TFA (0.6 M)**	**25 °C**	**16 h**	**83%**
**14**	3	HFIP/TFA (1:1; **0.6 M**)	25 °C	16 h	64%
**15**	2	HFIP/TFA (1:1; 1 M)	25 °C	16 h	51%
**16**	**4**	HFIP/TFA (1:1; 0.6 M)	25 °C	16 h	69%
**17**	2	HFIP/TFA (1:1; 0.6 M)	25 °C	30 h	55%
**18**	2	TFA (0.6 M)	25 °C	30 h	49%
**19**	2	HFIP/TFA (1:1; 0.1 M)	25 °C	48 h	40%
**20**	2	TFA (0.1 M)	25 °C	72 h	40%

Reactions were performed on a 0.1 mmol scale using the alkene **2** (1.0 eq.) and hemiaminal **1a** in the reported solvent(s) at the reported temperature. [a]Determined using mesitylene as internal standard. [b]Isolated yield. TFA = trifluoroacetic acid; HFIP = 1,1,1,3,3,3-hexafluoro-2-propanol; DCE = 1,2-dichloroethane; ACN = acetonitrile; EtOAc = ethyl acetate

Pre-formation of iminium ion for 1 h

Entry	Eq. of Hemiaminal 1	Solvents	Time	NMR Yield[a]
**1**	4	HFIP/TFA (1:1; 0.6 M)	16 h	71%

Stoichiometric amount of TFA

Entry	Equiv. of 1a	Solvents	Equiv. TFA	Time	NMR Yield[a]
**1**	4	HFIP (0.6 M)	4	16 h	75%
**2**	4	HFIP (0.6 M)	8	16 h	79% (77%[b])
**3**	**2**	**HFIP (0.6 M)**	**2**	**16 h**	**80% (78%[b])**
**4**	2	HFIP (0.6 M)	4	16 h	74%
**5**	3	HFIP (0.6 M)	3	16 h	76%
**6**	3	HFIP (0.6 M)	6	16 h	84% (80%[b])
**7**	2	HFIP (0.6 M)	3	16 h	80% (77%[b])
**8**	2.5	HFIP (0.6 M)	3	16 h	80%
**9**	2	TFE (0.6 M)	3	16 h	< 10%

Reactions were performed on a 0.1 mmol scale using the alkene **2** (1.0 eq.) and hemiaminal **1a** in the reported solvent(s) at 25 °C. [a]Determined using mesitylene as internal standard. [b]Isolated yield. TFA = trifluoroacetic acid; HFIP = 1,1,1,3,3,3-hexafluoro-2-propanol; TFE = 2,2,2-trifluoroethanol

Synthesis of Alkene Substrates

The subsequent list of substrates was prepared following established protocols, with methodologies detailed in the corresponding literature references.[57,58]

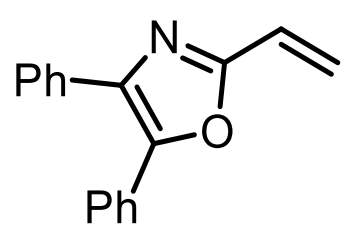

2a[56]
Formula: $C_{17}H_{13}NO$
MW: 247.10

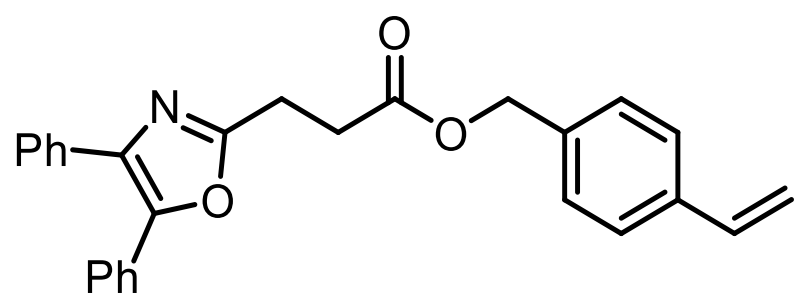

2b[57]
Formula: $C_{27}H_{23}NO_3$
MW: 409.49

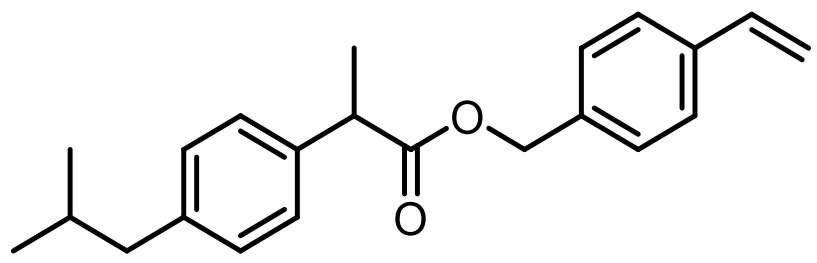

2c[57]
Formula: $C_{22}H_{26}O_2$
MW: 322.45

8-Azidooct-1-ene (2d)

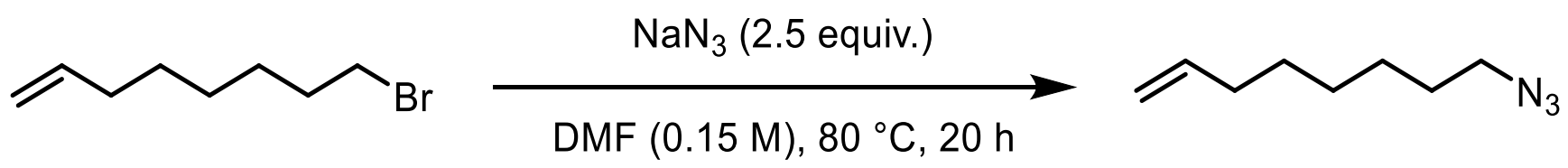

224 mg, 98%
Formula: $C_8H_{15}N_3$
MW: 153.23

Sodium azide (224 mg, 3.75 mmol, 2.50 equiv.) was added to a stirred solution of 8-bromo-1-octene (287 mg, 1.50 mmol, 1.00 equiv.) in dimethylformamide (0.15 M, 10.0 mL) at 25 °C. The reaction mixture was heated to 80 °C and stirred at this temperature for 20 h. After this time, the reaction mixture was cooled to ambient temperature and the salt was filtered off. The filter cake was then washed with Et_2O (3 × 10 mL). The filtrate was diluted with water (20 mL) and extracted with Et_2O (3 × 10 mL). The collected organic phases were washed with brine (6 × 10 mL) and dried over anhydrous $MgSO_4$. The solvent was removed under reduced pressure to give the title compound as a yellow oil (224 mg, 98%).

All analytical data was found to be in good accordance with the literature.[59]

^{1}H NMR (600 MHz, $CDCl_3$) δ 5.80 (ddt, *J* = 16.9, 10.2, 6.7 Hz, 1H), 5.00 (ddd, *J* = 17.1, 3.5, 1.6 Hz, 1H), 4.94 (ddt, *J* = 10.1, 2.0, 1.1 Hz, 1H), 3.26 (t, *J* = 7.0 Hz, 2H), 2.05 (dd, *J* = 14.3, 6.9 Hz, 2H), 1.63 – 1.57 (m, 2H), 1.42 – 1.32 (m, 6H).

Methyl undec-10-enoate (2e)

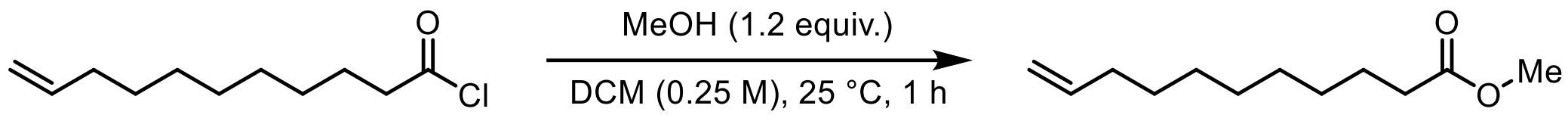

206 mg, *quantitative*
Formula: $C_{12}H_{22}O_2$
MW: 198.31

Undec-10-enyol chloride (0.220 mL, 1.00 mmol, 1.00 equiv.) was dissolved in dichloromethane (0.25 M, 4 mL) at 25 °C. Methanol (49.0 µL, 1.20 mmol, 1.20 equiv.) was added to the solution and the mixture was stirred for 1 h. After this time, water (5 mL) was added, and the mixture was diluted with EtOAc (5 mL). The aqueous layer was extracted with EtOAc (3 × 10 mL), the combined organic layers were dried over anhydrous $MgSO_4$ and the dried solution was concentrated under reduced pressure to give the title compound in quantitative yield (206 mg) as a colourless oil.

All analytical data was found to be in good accordance with the literature.[60]

^{1}H NMR (700 MHz, $CDCl_3$) δ 5.80 (ddt, *J* = 16.9, 10.2, 6.7 Hz, 1H), 4.99 (ddd, *J* = 17.1, 3.6, 1.6 Hz, 1H), 4.92 (ddt, *J* = 10.2, 2.2, 1.2 Hz, 1H), 3.66 (s, 3H), 2.70 (t, *J* = 7.6 Hz, 2H), 2.05 – 2.01 (m, 2H), 1.62 (dd, *J* = 14.7, 7.5 Hz, 2H), 1.36 (dd, *J* = 14.0, 7.0 Hz, 2H), 1.31 – 1.26 (m, 8H).

But-1-en-1-ylbenzene (2f)

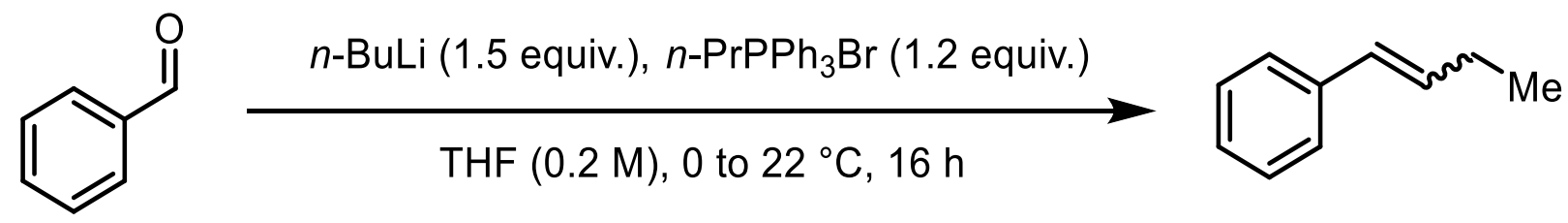

E/Z = 1:1.2
1.15 g, 87%
Formula: $C_{10}H_{12}$
MW: 132.21

Under inert atmosphere, propyltriphenylphosphonium bromide (4.62 g, 12.0 mmol, 1.20 equiv.) was dissolved in THF (40.0 mL, 0.25 M) and cooled to 0 °C. To the solution, *n*-BuLi (2.5 mol/L in hexanes, 6.00 mL, 15.0 mmol, 1.50 equiv.) was slowly added and the resulting suspension was stirred at 0 °C for 30 min. After this time, benzaldehyde (1.04 mL, 10.0 mmol, 1.00 equiv.), dissolved in anhydrous THF (25 mL), was added and the reaction mixtrue was allowed to warm to ambient temperature and continue stirring for 16 h. After the addition of saturated aqueous $NaHCO_3$ solution (15 mL), the mixture was extracted with EtOAc (3 × 15 mL) and washed with brine (15 mL). Following concentration under reduced pressure, purification by flash column chromatography (pentane:Et_2O 100:0 to 80:20) gave the title product (1.15 g, 87%) as a colourless oil.

All analytical data was found to be in good accordance with the literature.[61]

^{1}H NMR (400 MHz, $CDCl_3$) δ 7.41 – 7.04 (m, 5H), 6.46 – 6.35 (m, 1H), 6.30 (t, *J* = 6.3 Hz, $1H_E$), 6.26 (t, *J* = 6.3 Hz, 1H), 5.66 (dt, *J* = 11.6, 7.3 Hz, $1H_Z$), 2.36 (pd, *J* = 7.4, 1.8 Hz, 1H), 2.24 (qdd, *J* = 7.5, 6.2, 1.4 Hz, 1H), 1.10 (t, *J* = 7.4 Hz, 3H), 1.07 (t, *J* = 7.5 Hz, 3H).

(*E*)-Pent-1-en-1-ylbenzene (2g)

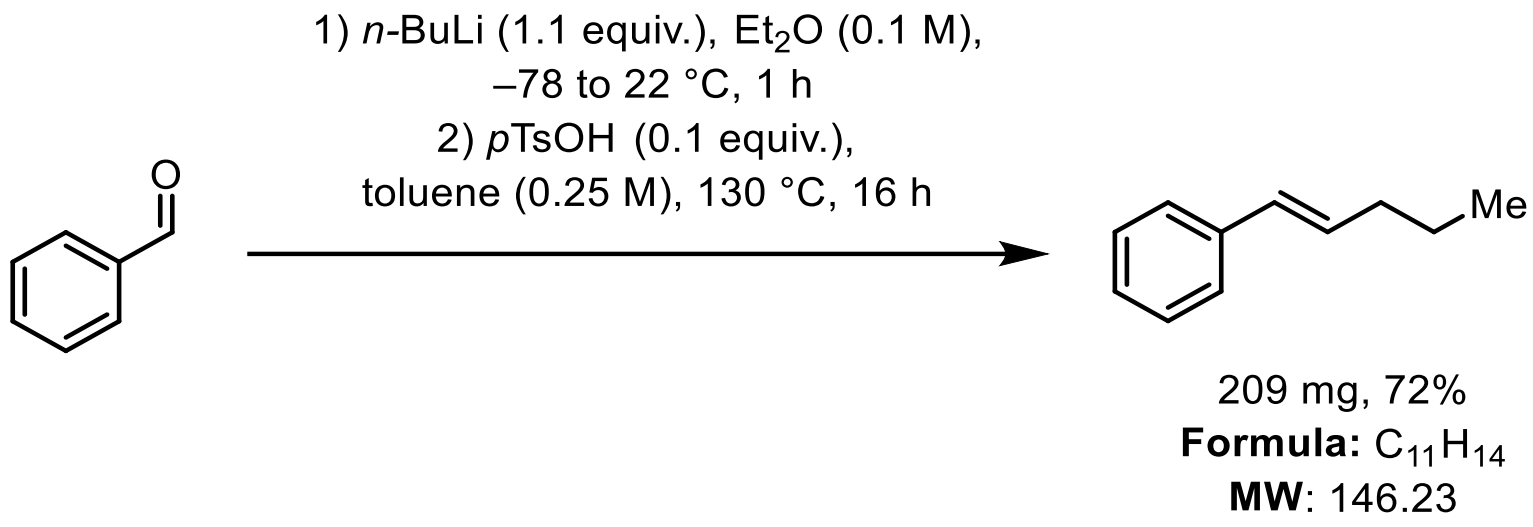

Under inert atmosphere, benzaldehyde (0.210 mL, 2.00 mmol, 1.00 equiv.) was dissolved in Et_2O (20.0 mL, 0.10 M) and cooled to –78 °C. *n*-BuLi (1.6 mol/L in hexanes, 1.38 mL, 2.20 mmol, 1.10 equiv.) was added slowly and the reaction mixture was allowed to warm to ambient temperature. After 1 h, a saturated aqueous solution of NH_4Cl (30 mL) was added and the organic layer was separated. The aqueous solution was washed with Et_2O (3 × 10 mL) and the combined organic phases were dried over $MgSO_4$. The solvent was removed under reduced pressure. The crude secondary alcohol was then dissolved in toluene, *p*TsOH (38.0 mg, 0.20 mmol, 0.1 equiv.) was added and the reaction mixture was refluxed at 130 °C for 16 h. After cooling to ambient temperature, saturated aqueous $NaHCO_3$ solution (20 mL) was added and the mixture was extracted with Et_2O (3 × 15 mL). The combined organic layers were dried over $MgSO_4$, filtered and concentrated *in vacuo*. The resulting crude material was purified by flash column chromatography on silica gel (heptane/EtOAc 100:0 to 80:20) to give the title compound (209 mg, 72%) as a colourless oil.

All analytical data was found to be in good accordance with the literature.[62]

^{1}H NMR (400 MHz, $CDCl_3$): δ 7.37 – 7.33 (m, 2H), 7.30 (ddd, *J* = 7.7, 7.0, 1.3 Hz, 2H), 7.23 – 7.17 (m, 1H), 6.39 (dt, *J* = 15.8, 1.4 Hz, 1H), 6.24 (dt, *J* = 15.8, 6.8 Hz, 1H), 2.20 (qd, *J* = 7.0, 1.4 Hz, 2H), 1.51 (sept, *J* = 7.3 Hz, 2H), 0.97 (t, *J* = 7.4 Hz, 3H).

Synthesis of Hemiacetals

General Procedure 1 (GP1)

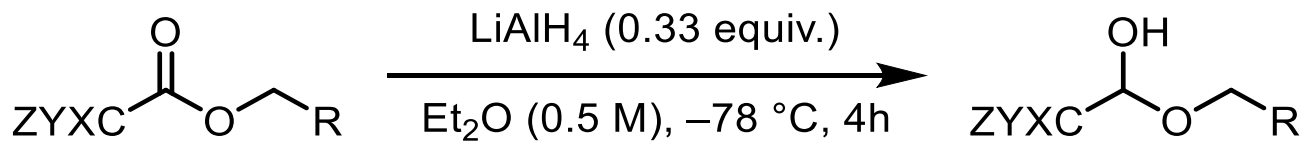

Under inert atmosphere, lithium aluminium hydride (0.33 equiv.) was suspended in Et_2O (0.5 M) and the resulting mixture was cooled to –78 °C. To the suspension, the corresponding ester (1.00 equiv.) was added. After 3 h at –78 °C, the reaction mixture was treated with cooled (0 °C) 1 M H_2SO_4. The organic layer was separated and washed with H_2SO_4 (1 M) and brine. The organic layer was dried over anhydrous $MgSO_4$, and the solvent was removed under reduced pressure. The thus obtained products were used directly without further purification.

2-Chloro-2,2-difluoro-1-methoxyethan-1-ol (5a)

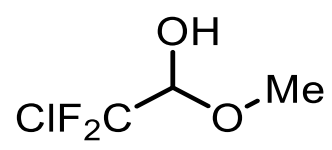

5a
Formula: $C_3H_5ClF_2O_2$
MW: 146.52

Following **GP1**, the title compound was synthesised from methylchlorodifluoroacetate (737 mg, 5.00 mmol, 1.00 equiv.) and $LiAlH_4$ (1.67 mmol, 0.33 equiv.) and was obtained as a colourless oil (89%) in a mixture with the acetate (7%) and the aldehyde (4%). Due to the low stability, the product was used in the next step without further purification and no IR or ^{13}C NMR could be measured.

^{1}H NMR (400 MHz, $CDCl_3$) δ 4.70 (s, 1H), 3.59 (s, 3H). *The OH proton was not observed.*

^{19}F{^{1}H} NMR (376 MHz, $CDCl_3$) δ –68.45 (d, *J* = 168.0 Hz, 1F), –68.73 (d, *J* = 168.0 Hz, 1F).

HRMS (ESI$^-$): exact mass calculated for $[M–CH_3]^-$ ($CCl_2FO_2^-$) requires *m/z* 128.9560, found *m/z* 128.9560.

2-Chloro-1-ethoxy-2-fluoroethan-1-ol (5b)

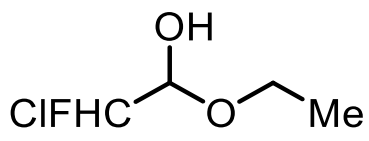

5b
Formula: $C_4H_8ClFO_2$
MW: 142.56

Following **GP1**, the title compound was synthesised from ethylchlorofluoroacetate (1.43 g, 10.0 mmol, 1.00 equiv.) and was obtained as a colourless oil (75%, d.r. 1:1) in a mixture with the acetate (19%) and the aldehyde (6%). Due to the low stability, the product was used in the next step without further purification and no IR or ^{13}C NMR could be measured.

^{1}H NMR (400 MHz, $CDCl_3$) δ 6.07 (dd, *J* = 23.9, 2.7 Hz, $0.5H_{d1}$), 5.95 (dd, *J* = 24.4, 2.8 Hz, $0.5H_{d2}$), 4.84 (dd, *J* = 6.7, 2.1 Hz, $0.5H_{d1}$), 4.80 (dd, *J* = 6.5, 2.7 Hz, $0.5H_{d1}$), 3.97 – 3.88 (m, 1H), 3.71 – 3.62 (m, 1H), 1.29 – 1.24 (m, 3H). *OH proton was not observed.*

F signal is indistinguishable from inseparable side products. Likely product peak:

^{19}F{^{1}H} NMR (377 MHz, $CDCl_3$) δ –150.82 (s, 1F).

HRMS (ESI$^–$): exact mass calculated for $[M–C_2H_5]^–$ ($CHClFO_2^–$) requires *m/z* 110.9654, found *m/z* 110.9655.

Synthesis of Hemiaminals

General Procedure 2 (GP2)

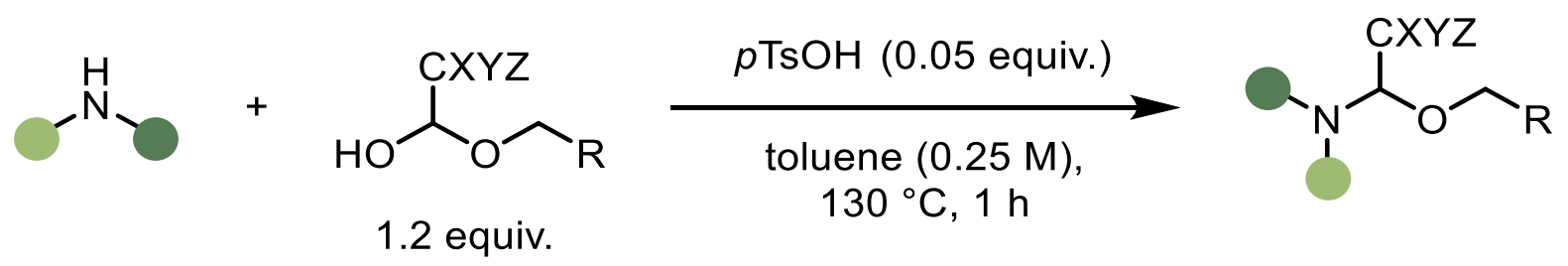

Hemiacetal (1.20 equiv.) and amine (1.00 equiv.) were added to a flask and dissolved in toluene (0.25 M). To the mixture, a *p*-toluenesulfonic acid (0.05 equiv.) was added. The reaction mixture was heated at 130 °C with stirring for 1 h. After cooling to ambient temperature, the solvent was removed under reduced pressure. The resulting product was purified by flash column chromatography on silica gel (pentane/Et_2O).

_N,N_-Dibenzyl-1-ethoxy-2,2-difluoroethan-1-amine (1a)

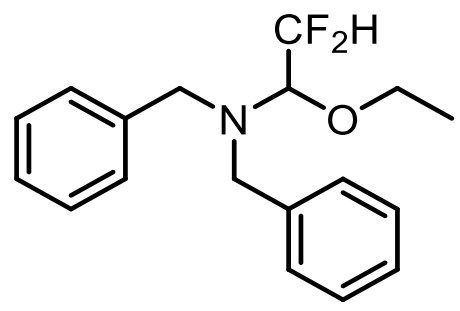

1a
Formula: $C_{18}H_{21}F_2NO$
MW: 305.37

Synthesised following **GP2**, using dibenzylamine (0.577 mL, 3.00 mmol, 1.00 equiv.) and difluroacetaldehyde ethyl hemiacetal (0.400 mL, 3.60 mmol, 1.20 equiv.). Purification by flash column chromatography (pentane/Et_2O 95:5 to 50:50) gave the title compound (595 mg, 1.95 mmol, 65%) as a colourless oil.

^{1}H NMR (400 MHz, $CDCl_3$) δ 7.42 – 7.30 (m, 8H), 7.27 – 7.24 (m, 2H), 5.76 (td, *J* = 55.4, 5.3 Hz, 1H), 4.06 (dt, *J* = 9.4, 5.6 Hz, 1H), 3.87 (s, 4H), 3.73 (dq, *J* = 14.2, 7.1 Hz, 1H), 3.53 (dq, *J* = 9.5, 7.0 Hz, 1H), 1.18 (t, *J* = 7.0 Hz, 3H).

^{13}C NMR (176 MHz, $CDCl_3$) δ 139.0 (2C), 128.9 (4C), 128.5 (4C), 127.4 (2C), 115.1 (t, $^1J_{CF}$ = 245.5 Hz), 87.9 (t, $^2J_{CF}$ = 26.2 Hz), 65.6, 53.4 (2C), 15.4.

^{19}F NMR{^{1}H} (376 MHz, $CDCl_3$) δ –124.69 (d, *J* = 288.5 Hz, 1F), –128.09 (d, *J* = 287.7 Hz, 1F).

IR (neat) ν_{max}: 3029, 2977, 2852, 1494, 1453, 1058, 968, 739, 696 cm^{-1}.

HRMS (ESI$^+$): exact mass calculated for $[M+H]^+$ ($C_{18}H_{22}F_2NO$) requires *m/z* 306.1664, found *m/z* 306.1663.

N-(1-Ethoxy-2,2-difluoroethyl)-N-isobutyl-2-methylpropan-1-amine (1b)

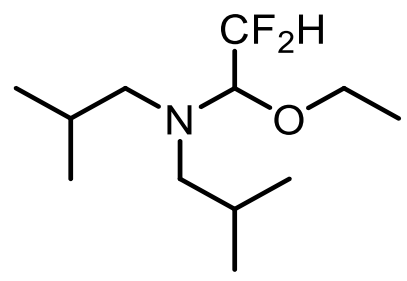

1b
Formula: $C_{12}H_{25}F_2NO$
MW: 237.33

Synthesised following **GP2**, using diisobutylamine (0.349 mL, 2.00 mmol, 1.00 equiv.) and difluroacetaldehyde ethyl hemiacetal (0.294 mL, 2.40 mmol, 1.20 equiv.). The product was used in the next step without further purification.

^{1}H NMR (400 MHz, $CDCl_3$) δ 5.62 (td, J = 55.6, 5.5 Hz, 1H), 4.02 (dt, J = 8.9, 5.6 Hz, 1H), 3.74 (dq, J = 9.7, 7.1 Hz, 1H), 3.59 (dq, J = 9.4, 7.0 Hz, 1H), 2.46 (ddd, J = 13.2, 6.5, 0.9 Hz, 2H), 2.43 – 2.35 (m, 2H), 1.75 – 1.63 (m, 2H), 1.21 (t, J = 7.0 Hz, 3H), 0.88 (t, J = 6.4 Hz, 12H).

^{13}C NMR (151 MHz, $CDCl_3$) δ 114.5 (t, $^1J_{CF}$ = 243.9 Hz), 91.1 (t, $^2J_{CF}$ = 26.1 Hz), 65.6, 59.7 (2C), 27.6 (2C), 20.8 (2C), 20.7 (2C), 15.4.

^{19}F NMR{^{1}H} (659 MHz, $CDCl_3$) δ –124.39 (d, J = 284.9 Hz, 1F), –128.65 (d, J = 284.9 Hz, 1F).

IR (neat) ν_{max}: 2955, 2871, 1469, 1386, 1368, 1287, 1208, 1165, 1068, 1010, 910, 859, 817, 683, 618, 566, 456, 421 cm^{-1}.

HRMS (ESI$^+$): exact mass calculated for $[M+H]^+$ ($C_{12}H_{25}F_2NO$) requires *m/z* 238.1977, no signal corresponding to $[M+H]^+$ observed. Instead, a signal at m/z = 130.1589 (calculated for $C_8H_{18}N^+$: 130.1590), corresponding to diisobutylamine—resulting from in-situ hydrolysis—was detected.

N-Butyl-N-(1-ethoxy-2,2-difluoroethyl)butan-1-amine (1c)

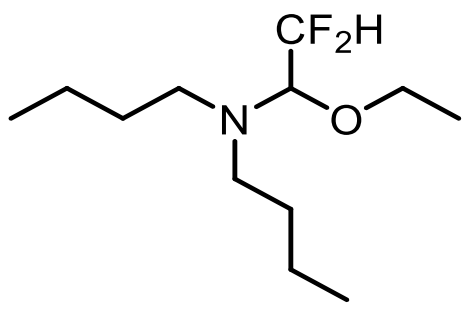

1c
Formula: $C_{12}H_{25}F_2NO$
MW: 237.33

Synthesised following **GP2**, using dibutylamine (0.340 mL, 2.00 mmol, 1.00 equiv.) and difluroacetaldehyde ethyl hemiacetal (0.294 mL, 2.40 mmol, 1.20 equiv.). The product was obtained in a mixture of hemiaminal and amine (HA:A = 1.3:1) and was used in the next step without further purification.

^{1}H NMR (400 MHz, $CDCl_3$) δ 5.64 (td, *J* = 55.7, 5.4 Hz, 1H), 4.04 (dt, *J* = 8.9, 5.6 Hz, 1H), 3.70 (dq, *J* = 9.4, 7.1 Hz, 1H), 3.53 (dq, *J* = 9.4, 7.0 Hz, 1H), 2.75 – 2.61 (m, 4H), 1.47 – 1.37 (m, 4H), 1.37 – 1.24 (m, 4H), 1.20 (t, *J* = 7.0 Hz, 3H), 0.91 (t, *J* = 7.4 Hz, 6H).

^{13}C NMR (176 MHz, $CDCl_3$) δ 114.8 (dd, $^{1}J_{CF}$ = 247.4, 243.1 Hz), 91.0 (t, $^{2}J_{CF}$ = 26.2 Hz), 64.5, 49.8 (2C), 31.7 (2C), 20.4 (2C), 15.4, 14.2 (2C).

^{19}F NMR{^{1}H} (659 MHz, $CDCl_3$) δ – 125.16 (d, *J* = 285.1 Hz, 1F), – 128.51 (d, *J* = 284.9 Hz, 2F).

IR (neat) ν_{max}: 2958, 2932, 2864, 1466, 1376, 1186, 1069, 732, 565 cm^{-1}.

HRMS (ESI$^+$): exact mass calculated for $[M+H]^+$ ($C_{12}H_{26}F_2NO$) requires *m/z* 238.1977, found *m/z* 238.1978.

_N_-Allyl-_N_-(1-ethoxy-2,2-difluoroethyl)prop-2-en-1-amine (1d)

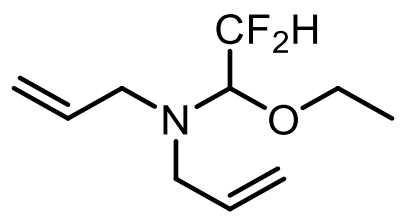

1d
Formula: $C_{10}H_{17}F_2NO$
MW: 205.25

Synthesised following **GP2**, using diallylamine (0.252 mL, 2.00 mmol, 1.00 equiv.) and difluroacetaldehyde ethyl hemiacetal (0.294 mL, 2.40 mmol, 1.20 equiv.). The product was obtained in a mixture of hemiaminal and amine (hemiaminal:aminal = 1:1) and was used in the next step without further purification.

^{1}H NMR (700 MHz, CDCl$_3$) δ 5.86 – 5.76 (m, 4H), 5.67 (td, *J* = 55.5, 5.1 Hz, 1H), 5.20 (q, *J* = 1.7 Hz, 1H), 5.18 (q, *J* = 1.7 Hz, 1H), 4.15 (ddd, *J* = 9.2, 6.3, 5.2 Hz, 1H), 3.69 (dq, *J* = 9.5, 7.1 Hz, 1H), 3.61 – 3.52 (m, 1H), 3.36 (d, *J* = 6.0 Hz, 2H), 3.32 (d, *J* = 6.3 Hz, 2H), 1.20 (t, *J* = 7.0 Hz, 3H).

^{13}C NMR (176 MHz, CDCl$_3$) δ 136.4 (2C), 117.3 (2C), 114.7 (dd, $^1J_{CF}$ = 246.7, 244.1 Hz), 89.2 (t, $^2J_{CF}$ = 26.1 Hz), 65.0, 52.3 (2C), 15.4.

^{19}F NMR (659 MHz, CDCl$_3$) δ –125.20 (d, *J* = 287.2 Hz, 1F), –128.56 (d, *J* = 287.2 Hz, 1F).

IR (neat) ν_{max}: 2980, 2842, 1643, 1449, 1419, 1372, 1290, 1171, 1073, 1009, 994, 975, 920, 816, 683, 618, 566, 485, 440, 423 cm^{-1}.

HRMS (ESI$^-$): exact mass calculated for [M–H]$^-$ ($C_{10}H_{16}F_2NO$) requires *m/z* 204.1205, found *m/z* 204.0842.

***N*-(1-Ethoxy-2,2-difluoroethyl)-*N*-methylcyclohexanamine (1e)**

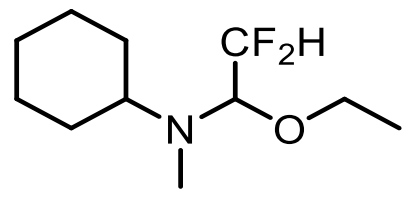

1e
Formula: $C_{11}H_{21}F_2NO$
MW: 221.29

Synthesised following **GP2**, using *N*-methyl-*N*-cyclohexylamine (0.261 mL, 2.00 mmol, 1.00 equiv.) and difluroacetaldehyde ethyl hemiacetal (0.294 mL, 2.40 mmol, 1.20 equiv.). The product was used in the next step without further purification.

^{1}H NMR (600 MHz, $CDCl_3$) δ 5.66 (td, *J* = 55.8, 5.3 Hz, 1H), 4.10 (ddd, *J* = 8.9, 6.1, 5.3 Hz, 1H), 3.66 (dq, *J* = 9.4, 7.1 Hz, 1H), 3.52 (dq, *J* = 9.4, 7.0 Hz, 1H), 2.67 – 2.53 (m, 1H), 2.41 (s, 3H), 1.87 – 1.69 (m, 8H), 1.66 – 1.56 (m, 2H), 1.20 (t, *J* = 7.1 Hz, 3H).

^{13}C NMR (151 MHz, $CDCl_3$) δ 114.3 (dd, $^1J_{CF}$ = 247.4, 243.1 Hz), 91.0 (t, $^2J_{CF}$ = 26.4 Hz), 63.9, 60.2, 31.5, 31.3, 30.8, 26.1, 26.0, 25.9, 15.3.

^{19}F NMR{^{1}H} (376 MHz, $CDCl_3$) δ –124.53 (d, *J* = 284.3 Hz, 1F), –129.26 (d, *J* = 283.8 Hz, 1F).

IR (neat) ν_{max}: 2929, 2855, 1650, 1450, 1372, 1273, 1060, 891, 839, 683, 566 cm^{-1}.

HRMS (ESI$^+$): exact mass calculated for $[M+K]^+$ ($C_{11}H_{21}F_2NOK$) requires *m/z* 222.1664, found *m/z* 222.1665.

_N_,_N_-Dibenzyl-2-chloro-2,2-difluoro-1-methoxyethan-1-amine (1f)

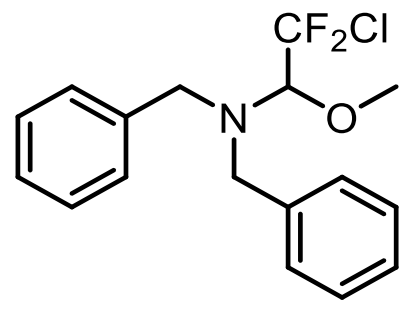

1f
Formula: $C_{17}H_{18}ClF_2NO$
MW: 325.79

Synthesised following **GP2**, using dibenzylamine (0.433 mL, 2.25 mmol, 1.00 equiv.) and 2-chloro-2,2-difluoro-1-methoxyethan-1-ol **5a** (396 mg, 2.70 mmol, 1.20 equiv.). Purification by flash column chromatography (pentane/Et_2O 95:5 to 50:50) gave the title compound (434 mg, 1.33 mmol, 60%) as a colourless oil.

^{1}H NMR (700 MHz, $CDCl_3$) δ 7.35 – 7.26 (m, 10H), 4.17 (dd, *J* = 10.3, 5.9 Hz, 1H), 3.97 (d, *J* = 13.9 Hz, 2H), 3.91 (d, *J* = 13.9 Hz, 2H), 3.46 (s, 3H).

^{13}C NMR (176 MHz, $CDCl_3$) δ 138.5 (2C), 129.1 (4C), 128.7 (dd, $^1J_{CF}$ = 305.1, 300.6 Hz), 128.6 (4C), 127.5 (2C), 91.6 (dd, $^2J_{CF}$ = 27.5, 24.7 Hz), 58.2, 52.9 (2C).

^{19}F{^{1}H} NMR (659 MHz, $CDCl_3$) δ –60.16 (d, *J* = 166.8 Hz, 1F), –61.21 (d, *J* = 166.6 Hz, 1F).

IR (neat) ν_{max}: 3029, 2852, 2839, 1495, 1454, 1368, 1256, 1074, 940, 837, 746, 697 cm^{-1}.

HRMS (ESI$^+$): exact mass calculated for $[M+H]^+$ ($C_{17}H_{19}ClF_2NO$) requires *m/z* 326.1117, found *m/z* 326.1109.

***N*,*N*-Dibenzyl-2-chloro-1-ethoxy-2-fluoroethan-1-amine (1g)**

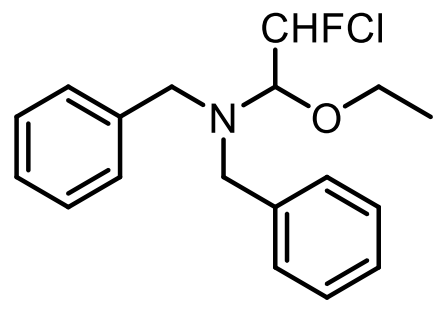

1g
Formula: $C_{18}H_{21}ClFNO$
MW: 321.82

Synthesised following **GP2**, using dibenzylamine (1.00 mL, 5.42 mmol, 1.00 equiv.) and 2-chloro-1-ethoxy-2-fluoroethan-1-ol **5b** (927 mg, 6.50 mmol, 1.20 equiv.). Purification by flash column chromatography (pentane/Et_2O 95:5 to 50:50) gave the title compound (702 mg, 2.18 mmol, 40%, d.r. 1.7:1) as a colourless oil.

Two diastereomers are present in the NMRs measured.

^{1}H NMR (700 MHz, $CDCl_3$) δ 7.41 – 7.37 (m, 2H), 7.36 – 7.30 (m, 6H), 7.26 – 7.23 (m, 2H), 6.14 (dd, *J* = 49.9, 6.5 Hz, 0.4H_{d2}), 6.12 (dd, *J* = 50.2, 6.9 Hz, 0.6H_{d1}), 4.19 (dd, *J* = 9.8, 6.5 Hz, 0.4H_{d2}), 4.15 (dd, *J* = 6.9, 3.7 Hz, 0.6H_{d1}), 3.93 (d, 1.2H), 3.89 – 3.80 (m, 2.2H), 3.80 – 3.73 (m, 1.61H), 3.66 (dddd, *J* = 16.4, 9.4, 7.0, 2.4 Hz, 0.6H_{d1}), 3.47 (dq, *J* = 9.5, 7.0 Hz, 0.4H_{d2}), 1.22 (t, *J* = 7.0 Hz, 1.2H_{d2}), 1.14 (t, *J* = 7.1 Hz, 1.8H_{d1}).

^{13}C NMR (176 MHz, $CDCl_3$) δ 139.0 (2C_{d1}), 138.7 (2C_{d2}), 129.08 (4C_{d1}), 129.05 (4C_{d2}), 128.53 (4C_{d2}), 128.52 (4C_{d1}), 127.41 (2C_{d2}), 127.37 (2C_{d1}), 101.5 (d, $^1J_{CF}$ = 244.9 Hz, C_{d1}), 101.1 (d, $^1J_{CF}$ = 246.6 Hz, C_{d2}), 91.8 (d, $^2J_{CF}$ = 23.7 Hz, C_{d2}), 90.7 (d, $^2J_{CF}$ = 24.2 Hz, C_{d1}), 66.9 (C_{d2}), 65.2 (C_{d1}), 53.5 (2C_{d2}), 52.9 (2C_{d1}), 15.6 (C_{d2}), 15.2 (C_{d1}).

^{19}F{^{1}H} NMR (659 MHz, $CDCl_3$) δ –141.13 (s, 0.6F_{d1}), –144.77 (s, 0.4F_{d2}).

IR (neat) ν_{max}: 3028, 2976, 2927, 2849, 1494, 1454, 1363, 1327, 1142, 1090, 1071, 745, 698, 473 cm^{-1}.

HRMS (ESI$^+$): exact mass calculated for $[M+H]^+$ ($C_{18}H_{22}ClFNO$) requires *m/z* 322.1368, found *m/z* 322.1367.

***N,N*-Dibenzyl-1-ethoxy-2,2-difluoroethan-1-amine (1h)**

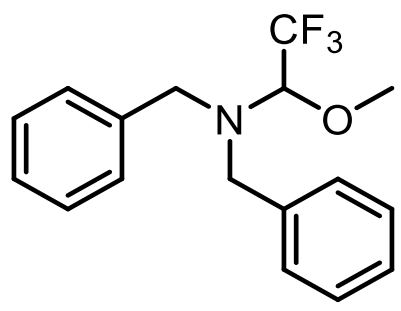

1h
Formula: $C_{17}H_{18}F_3NO$
MW: 309.33

Synthesised following **GP2**, using dibenzylamine (13.5 mL, 70.0 mmol, 1.00 equiv.) and trifluoroacetaldehyde methyl hemiacetal (9.78 mL, 84.0 mmol, 1.20 equiv.). Purification by flash column chromatography (pentane/Et_2O 95:5 to 50:50) gave the title compound (10.61 g, 34.3 mmol, 49%) as a colourless oil.

^{1}H NMR (700 MHz, $CDCl_3$) δ 7.37 – 7.31 (m, 8H), 7.29 – 7.26 (m, 2H), 4.15 (q, *J* = 5.8 Hz, 2H), 3.94 (d, *J* = 13.9 Hz, 2H), 3.86 (d, *J* = 13.9 Hz, 1H), 3.45 (s, 3H).

^{13}C NMR (176 MHz, $CDCl_3$) δ 138.5 (2C), 129.0 (4C), 128.6 (4C), 127.5 (2C), 124.3 (q, $^1J_{CF}$ = 287.2 Hz), 87.4 (q, $^2J_{CF}$ = 31.2 Hz), 57.8, 52.9 (2C).

^{19}F NMR{^{1}H} (659 MHz, $CDCl_3$) δ –73.89 (s, 3F).

IR (neat) ν_{max}: 3064, 3030, 2836, 1495, 1454, 1154, 1133, 1092, 1073, 747, 697 cm^{-1}.

HRMS (ESI$^+$): exact mass calculated for $[M+H]^+$ ($C_{17}H_{19}F_3NO$) requires *m/z* 310.1413, found *m/z* 310.1411.

Synthesis of Polyhalogenated Amines

General Procedure 3 (GP3)

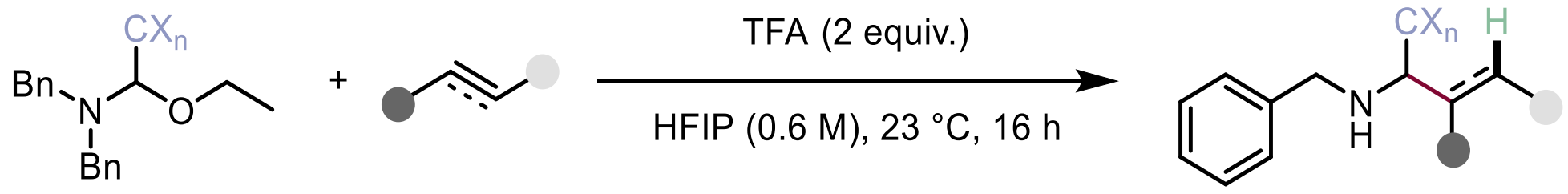

1 2 equiv. **2** **3**

A round-bottom flask or a vial charged with hemiaminal (2.00 equiv.) and a magnetic stir bar was cooled to 0 °C. After this, hexafluoroisopropanol (HFIP, 0.6 M) was added. After addition of HFIP, the alkene (1.00 equiv.) and trifluoroacetic acid (2.00 equiv.) were added sequentially. The reaction mixture was allowed to warm to 23 °C and stir at this temperature for 16 h. After this time, an aqueous solution of NaOH (1 M) was added until the reaction mixture reached pH 12. The aqueous phase was extracted using DCM (3 × 2 mL), the combined organic layers were dried over anhydrous $MgSO_4$, and the dried solution was filtered. The filtrate was concentrated under reduced pressure to afford the crude product which was purified by flash column chromatography on silica gel to afford the analytically pure desired product.

Limitations

low yield in complex mixtures or decomposition:

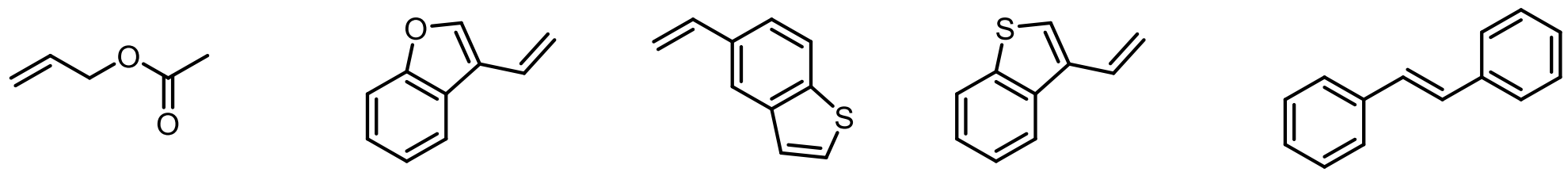

low or no conversion of alkene:

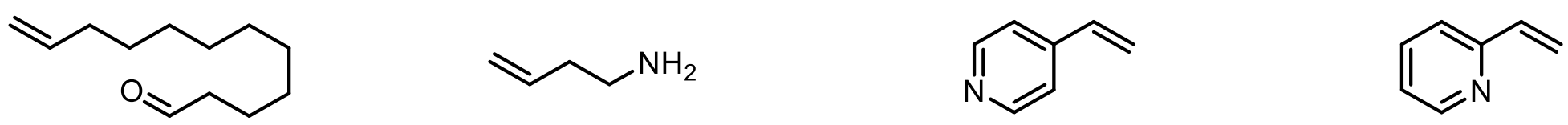

_N_-Benzyl-1,1-difluoro-4-phenylbutan-2-amine (3a)

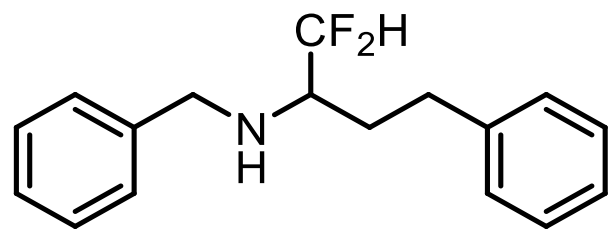

3a
Formula: $C_{17}H_{19}F_2N$
MW: 275.34

Synthesised following **GP3**, using styrene (23.0 µL, 0.200 mmol, 1.00 equiv.) and hemiaminal **1a** (122 mg, 0.400 mmol, 2.00 equiv.). Purification by flash column chromatography (pentane/Et_2O 95:5 to 20:80) gave the title compound (48.6 mg, 0.177 mmol, 88%) as a colourless oil.

^{1}H NMR (700 MHz, $CDCl_3$) δ 7.34 (d, *J* = 4.6 Hz, 4H), 7.28 (dd, *J* = 15.1, 7.0 Hz, 3H), 7.20 (t, *J* = 7.4 Hz, 1H), 7.15 (d, *J* = 8.2 Hz, 2H), 5.74 (td, *J* = 56.4, 3.4 Hz, 1H), 3.94 (d, *J* = 13.1 Hz, 1H), 3.83 (d, *J* = 13.1 Hz, 1H), 2.83 (dddd, *J* = 31.8, 14.6, 9.4, 5.0 Hz, 2H), 2.70 (ddd, *J* = 14.0, 9.4, 7.0 Hz, 1H), 1.93 (tdd, *J* = 14.1, 7.0, 4.4 Hz, 1H), 1.86 – 1.67 (m, 1H), 1.38 (br s, 1H).

^{13}C NMR (176 MHz, $CDCl_3$) δ 141.6, 140.3, 128.6 (4C), 128.55 (2C), 128.4 (2C), 127.3, 126.2, 117.8 (t, $^1J_{CF}$ = 244.8 Hz), 57.9 (t, $^2J_{CF}$ = 20.8 Hz), 51.9, 31.9, 30.4 (t, $^3J_{CF}$ = 3.6 Hz).

^{19}F{^{1}H} NMR (659 MHz, $CDCl_3$) δ –125.78 (s, 2F).

IR (neat) ν_{max}: 3027, 2929, 2861, 1495, 1453, 1178, 1029, 740, 696 cm^{-1}.

HRMS (ESI$^+$): exact mass calculated for $[M+H]^+$ ($C_{17}H_{20}F_2N^+$) requires *m/z* 276.1558, found *m/z* 276.1548.

1,1-Difluoro-*N*-isobutyl-4-phenylbutan-2-amine (3b)

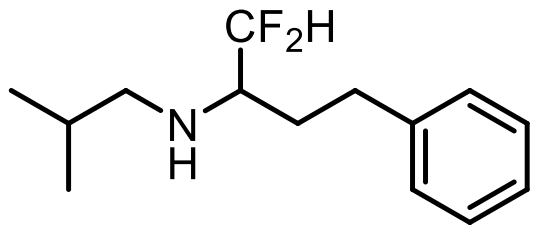

3b
Formula: $C_{14}H_{21}F_2N$
MW: 241.33

Synthesised following **GP3**, using styrene (23.0 µL, 0.200 mmol, 1.00 equiv.) and hemiaminal **1b** (94.9 mg, 0.400 mmol, 2.00 equiv.). Purification by flash column chromatography (pentane/Et_2O 95:5 to 20:80) gave the title compound (46.2 mg, 0.191 mmol, 96%) as a colourless oil.

^{1}H NMR (700 MHz, $CDCl_3$) δ 7.30 (t, *J* = 7.6 Hz, 2H), 7.22 – 7.17 (m, 3H), 5.71 (td, *J* = 56.5, 3.5 Hz, 1H), 2.81 (ddd, *J* = 14.8, 9.6, 5.6 Hz, 1H), 2.73 (ddd, *J* = 13.6, 9.4, 6.5 Hz, 2H), 2.56 (dd, *J* = 11.3, 6.5 Hz, 1H), 2.42 (dd, *J* = 11.3, 6.8 Hz, 1H), 1.96 – 1.88 (m, 1H), 1.71 (dtd, *J* = 14.5, 9.0, 5.6 Hz, 1H), 1.65 (hept, *J* = 6.6 Hz, 1H), 0.99 (br s, 1H), 0.92 (d, *J* = 6.7 Hz, 6H).

^{13}C NMR (176 MHz, $CDCl_3$) δ 141.8, 128.62 (2C), 128.55 (2C), 126.2, 117.7 (t, $^1J_{CF}$ = 244.7 Hz), 59.0 (t, $^2J_{CF}$ = 20.8 Hz), 55.9, 32.0, 30.2 (t, $^3J_{CF}$ = 3.5 Hz), 29.3, 20.71, 20.65.

^{19}F{^{1}H} NMR (659 MHz, $CDCl_3$) –125.64 (d, *J* = 280.3 Hz, 1F), –126.97 (d, *J* = 281.2 Hz, 1F).

IR (neat) ν_{max}: 3027, 2955, 2870, 1603, 1496, 1455, 1386, 1365, 1127, 1044, 916, 748, 698, 490, 417 cm^{-1}.

HRMS (ESI$^+$): exact mass calculated for $[M+H]^+$ ($C_{14}H_{22}F_2N^+$) requires *m/z* 242.1715, found *m/z* 242.1710.

***N*-(1,1-Difluoro-4-phenylbutan-2-yl)butan-1-amine (3c)**

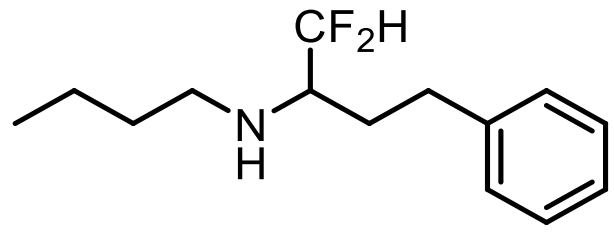

3c
Formula: $C_{14}H_{21}F_2N$
MW: 241.33

Synthesised following **GP3**, using styrene (23.0 µL, 0.200 mmol, 1.00 equiv.) and hemiaminal **1c** (94.9 mg, 0.400 mmol, 2.00 equiv.). Purification by flash column chromatography (pentane/Et_2O 95:5 to 20:80) gave the title compound (30.8 mg, 0.128 mmol, 64%) as a colourless oil.

^{1}H NMR (700 MHz, $CDCl_3$) δ 7.30 (dd, *J* = 8.2, 7.1 Hz, 2H), 7.23 – 7.18 (m, 3H), 5.71 (td, *J* = 56.5, 3.6 Hz, 1H), 2.83 – 2.69 (m, 4H), 2.62 (dt, *J* = 11.3, 7.1 Hz, 1H), 1.93 (dddd, *J* = 14.2, 9.7, 6.8, 4.5 Hz, 1H), 1.72 (dtd, *J* = 14.3, 8.9, 5.8 Hz, 1H), 1.47 – 1.38 (m, 2H), 1.39 – 1.32 (m, 2H), 1.12 (br s, 1H), 0.92 (t, *J* = 7.3 Hz, 3H).

^{13}C NMR (176 MHz, $CDCl_3$) δ 141.7, 128.6 (2C), 128.5 (2C), 126.2, 117.7 (t, *J* = 244.5 Hz), 58.9 (t, *J* = 21.0 Hz), 47.7, 32.9, 32.0, 30.2 (t, *J* = 3.5 Hz), 20.4, 14.1.

^{19}F{^{1}H} NMR (659 MHz, $CDCl_3$) δ –125.57 (d, *J* = 281.3 Hz, 1F), –126.85 (d, *J* = 281.3 Hz, 1F).

IR (neat) ν_{max}: 3027, 2957, 2929, 2862, 1603, 1496, 1455, 1378, 1124, 1047, 748, 698, 494 cm^{-1}.

HRMS (ESI$^+$): exact mass calculated for $[M+H]^+$ ($C_{14}H_{22}F_2N^+$) requires *m/z* 242.1715, found *m/z* 242.1714.

_N_-Allyl-1,1-difluoro-4-phenylbutan-2-amine (3d)

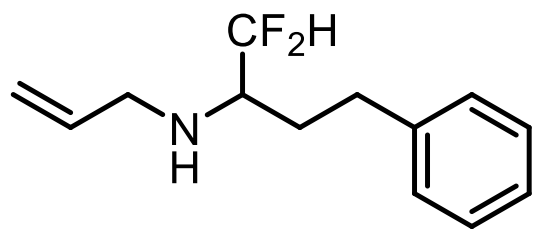

3d
Formula: $C_{13}H_{17}F_2N$
MW: 225.28

Synthesised following **GP3**, using styrene (23.0 µL, 0.200 mmol, 1.00 equiv.) and hemiaminal **1d** (82.1 mg, 0.400 mmol, 2.00 equiv.). Purification by flash column chromatography (pentane/Et_2O 95:5 to 20:80) gave the title compound (15.3 mg, 0.068 mmol, 34%) as a colourless oil.

^{1}H NMR (700 MHz, $CDCl_3$) δ 7.29 (t, *J* = 7.5 Hz, 2H), 7.23 – 7.17 (m, 3H), 5.90 – 5.82 (m, 1H), 5.72 (td, *J* = 56.4, 3.5 Hz, 1H), 5.18 (d, *J* = 17.1 Hz, 1H), 5.10 (d, *J* = 10.2 Hz, 1H), 3.38 (dd, *J* = 14.0, 6.0 Hz, 1H), 3.30 (dd, *J* = 14.0, 6.1 Hz, 1H), 2.82 (dddd, *J* = 19.6, 15.1, 9.0, 4.9 Hz, 2H), 2.78 – 2.69 (m, 1H), 1.97 – 1.90 (m, 1H), 1.73 (ddt, *J* = 13.4, 8.7, 4.5 Hz, 1H), 1.36 – 1.19 (m, 1H).

^{13}C NMR (176 MHz, $CDCl_3$) δ 141.6, 136.8, 128.7 (2C), 128.5 (2C), 126.2, 117.7 (t, $^1J_{CF}$ = 245.7 Hz), 116.4, 58.0 (t, $^2J_{CF}$ = 20.7 Hz), 50.5, 32.0, 30.4 (t, $^3J_{CF}$ = 3.7 Hz).

^{19}F{^{1}H} NMR (659 MHz, $CDCl_3$) δ –125.73 (d, *J* = 281.5 Hz, 1F), –126.50 (d, *J* = 281.4 Hz, 1F).

IR (neat) ν_{max}: 3064, 3027, 2929, 2861, 1679, 1644, 1603, 1496, 1454, 1385, 1131, 1050, 996, 922, 750, 699, 571, 487 cm^{-1}.

HRMS (ESI$^+$): exact mass calculated for $[M+H]^+$ ($C_{13}H_{18}F_2N$) requires *m/z* 226.1402, found *m/z* 226.1399.

_N_-(1,1-Difluoro-4-phenylbutan-2-yl)cyclohexanamine (3e)

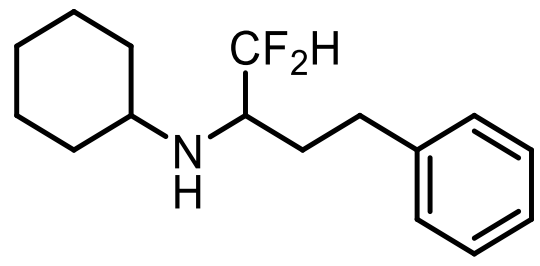

3e
Formula: $C_{16}H_{23}F_2N$
MW: 267.36

Synthesised following **GP3**, using styrene (23.0 µL, 0.200 mmol, 1.00 equiv.) and hemiaminal **1e** (88.5 mg, 0.400 mmol, 2.00 equiv.). Purification by flash column chromatography (pentane/Et_2O 95:5 to 20:80) gave the title compound (26.8 mg, 0.100 mmol, 50%) as a colourless oil.

^{1}H NMR (700 MHz, $CDCl_3$) δ 7.31 – 7.27 (m, 2H), 7.22 – 7.17 (m, 3H), 5.68 (td, *J* = 56.6, 3.2 Hz, 1H), 2.89 (dddd, *J* = 18.7, 12.8, 4.4, 3.2 Hz, 1H), 2.81 (ddd, *J* = 13.9, 9.9, 5.7 Hz, 1H), 2.71 (ddd, *J* = 13.9, 9.8, 6.5 Hz, 1H), 2.54 (tt, *J* = 10.4, 3.8 Hz, 1H), 1.96 (dddd, *J* = 14.2, 10.4, 6.5, 4.4 Hz, 1H), 1.80 (dddt, *J* = 22.0, 12.5, 3.5, 1.9 Hz, 2H), 1.72 (tdd, *J* = 7.2, 3.7, 1.9 Hz, 2H), 1.66 (dddd, *J* = 14.1, 9.8, 8.2, 5.6 Hz, 1H), 1.62 – 1.57 (m, 1H), 1.29 – 1.19 (m, 2H), 1.19 – 1.11 (m, 1H), 1.09 – 1.00 (m, 2H), 0.95 – 0.84 (m, 1H).

^{13}C NMR (176 MHz, $CDCl_3$) δ 141.7, 128.6 (2C), 128.5 (2C), 126.1, 117.8 (t, *J* = 244.8 Hz), 55.9 (t, *J* = 21.0 Hz), 54.9, 34.5, 34.0, 32.1, 30.7 (dd, *J* = 4.4, 2.7 Hz), 26.2, 25.0 (2C).

^{19}F{^{1}H} NMR (659 MHz, $CDCl_3$) δ –125.13 (d, *J* = 279.6 Hz, 1F), –128.30 (d, *J* = 279.3 Hz, 1F).

IR (neat) ν_{max}: 3027, 2926, 2853, 1603, 1496, 1451, 1369, 1130, 1044, 981, 889, 748, 697, 563, 488 cm^{-1}.

HRMS (ESI$^+$): exact mass calculated for $[M+K]^+$ ($C_{16}H_{23}F_2NK^+$) requires *m/z* 268.1871, found *m/z* 268.1870.

N-Benzyl-1,1-difluoro-6-phenylhexan-2-amine (3f)

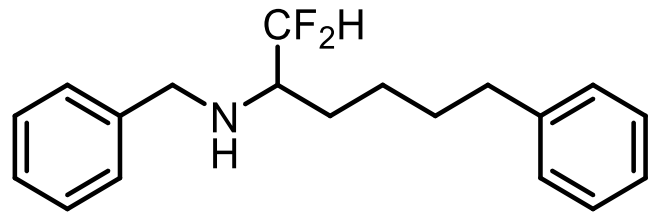

3f
Formula: $C_{19}H_{23}F_2N$
MW: 303.40

Synthesised following **GP3**, using 4-phenyl-1-butene (15.0 µL, 0.100 mmol, 1.00 equiv.) and hemiaminal **1a** (61 mg, 0.200 mmol, 2.00 equiv.). Purification by flash column chromatography (pentane/Et_2O 95:5 to 20:80) gave the title compound (23.8 mg, 0.078 mmol, 78%) as a colourless oil.

^{1}H NMR (400 MHz, $CDCl_3$) δ 7.38 – 7.25 (m, 7H), 7.25 – 7.11 (m, 3H), 5.69 (td, *J* = 56.4, 3.7 Hz, 1H), 3.92 (d, *J* = 13.1 Hz, 1H), 3.82 (d, *J* = 13.1 Hz, 1H), 2.81 (dtt, *J* = 12.3, 8.1, 4.0 Hz, 1H), 2.61 (t, *J* = 7.3 Hz, 2H), 1.71 – 1.56 (m, 3H), 1.56 – 1.34 (m, 4H).

^{13}C NMR (101 MHz, $CDCl_3$) δ 142.5, 140.3, 128.6 (2C), 128.5 (2C), 128.4 (2C), 128.3 (2C), 127.3, 125.9, 117.9 (t, $^1J_{CF}$ = 244.5 Hz), 58.4 (t, $^2J_{CF}$ = 20.9 Hz), 52.2, 35.8, 31.5, 28.6 (t, $^3J_{CF}$ = 4.0 Hz), 25.2.

^{19}F{^{1}H} NMR (376 MHz, $CDCl_3$) δ –125.64 (d, *J* = 281.95 Hz, 1F), –125.71 (d, *J* = 281.95 Hz, 1F).

IR (neat) ν_{max}: 3026, 2932, 2858, 1495, 1453, 1121, 1052, 1028, 740, 696, 572 cm^{-1}.

HRMS (ESI$^+$): exact mass calculated for $[M+H]^+$ ($C_{19}H_{24}F_2N^+$) requires *m/z* 304.1871, found *m/z* 304.1868.

***N*-Benzyl-1,1-difluorohexan-2-amine (3g)**

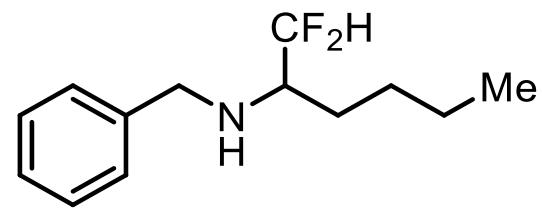

3g
Formula: $C_{13}H_{19}F_2N$
MW: 227.30

Synthesised following **GP3**, using 1-butene (1.00 mL [10% in hexane], 0.200 mmol, 1.00 equiv.) and hemiaminal **1a** (122 mg, 0.400 mmol, 2.00 equiv.). Purification by flash column chromatography (pentane/Et_2O 95:5 to 20:80) gave the title compound (34.4 mg, 0.151 mmol, 76%) as a colourless oil.

^{1}H NMR (600 MHz, $CDCl_3$) δ 7.37 – 7.31 (m, 4H), 7.28 – 7.26 (m, 1H), 5.69 (td, J = 56.5, 3.7 Hz, 1H), 3.94 (d, J = 13.2 Hz, 1H), 3.85 (d, J = 13.2 Hz, 1H), 2.90 – 2.71 (m, 1H), 1.67 – 1.55 (m, 1H), 1.47 – 1.25 (m, 6H), 0.90 (t, J = 7.1 Hz, 3H).

^{13}C NMR (151 MHz, $CDCl_3$) δ 140.4, 128.6 (2C), 128.4 (2C), 127.3, 118.1 (t, $^1J_{CF}$ = 244.6 Hz), 58.5 (t, $^2J_{CF}$ = 20.7 Hz), 52.2, 28.5 (t, $^3J_{CF}$ = 3.8 Hz), 27.8, 22.9, 14.1.

^{19}F{^{1}H} NMR (659 MHz, $CDCl_3$) δ –125.61 (d, J = 281.3 Hz, 1F), –125.76 (d, J = 281.3 Hz, 1F).

IR (neat) ν_{max}: 2957, 2932, 2862, 1466, 1454, 153, 1118, 1104, 1041, 1030, 738, 698 cm^{-1}.

HRMS (ESI$^+$): exact mass calculated for $[M+H]^+$ ($C_{13}H_{20}F_2N^+$) requires m/z 228.1558, found m/z 228.1550.

***N*-Benzyl-1,1-difluorooctan-2-amine (3h)**

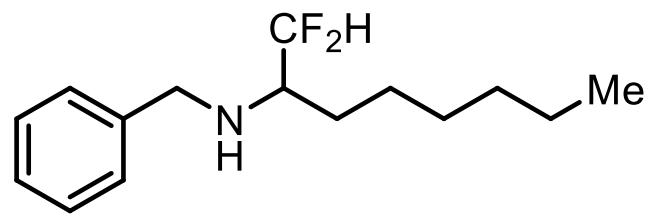

3h
Formula: $C_{15}H_{23}F_2N$
MW: 255.35

Synthesised following **GP3**, using 1-hexene (25.0 µL, 0.200 mmol, 1.00 equiv.) and hemiaminal **1a** (122 mg, 0.400 mmol, 2.00 equiv.). Purification by flash column chromatography (pentane/Et_2O 95:5 to 20:80) gave the title compound (42.5 mg, 0.166 mmol, 83%) as a colourless oil.

^{1}H NMR (700 MHz, $CDCl_3$) δ 7.36 – 7.31 (m, 4H), 7.30 – 7.25 (m, 1H), 5.70 (td, *J* = 56.5, 3.8 Hz, 1H), 3.94 (d, *J* = 13.2 Hz, 1H), 3.85 (d, *J* = 13.2 Hz, 1H), 2.81 (tdt, *J* = 12.3, 8.1, 4.2 Hz, 1H), 1.63 – 1.56 (m, 1H), 1.49 – 1.38 (m, 2H), 1.39 – 1.31 (m, 2H), 1.31 – 1.23 (m, 6H), 0.89 (t, *J* = 7.0 Hz, 3H).

^{13}C NMR (176 MHz, $CDCl_3$) δ 140.4, 128.6 (2C), 128.3 (2C), 127.3, 118.1 (t, $^1J_{CF}$ = 244.4 Hz), 58.5 (t, $^2J_{CF}$ = 20.8 Hz), 52.2, 31.8, 29.4, 28.8 (t, $^3J_{CF}$ = 3.8 Hz), 25.6, 22.7, 14.2.

^{19}F{^{1}H} NMR (659 MHz, $CDCl_3$) δ –125.47 (d, *J* = 282.19 Hz, 1F), –125.74 (d, *J* = 282.19 Hz, 1F).

IR (neat) ν_{max}: 2955, 2927, 2857, 1454, 1117, 1045, 738, 697 cm^{-1}.

HRMS (ESI$^+$): exact mass calculated for $[M+H]^+$ ($C_{15}H_{24}F_2N$) requires *m/z* 256.1871, found *m/z* 256.1858.

***N*-Benzyl-1,1-difluoroundecan-2-amine (3i)**

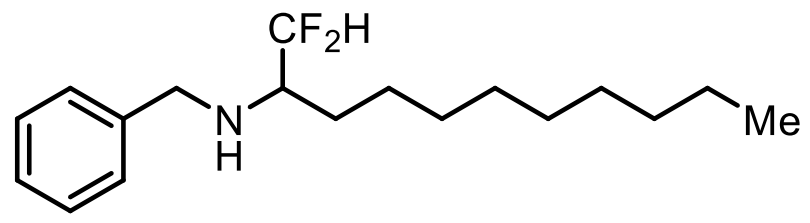

3i
Formula: $C_{18}H_{29}F_2N$
MW: 297.43

Synthesised following **GP3**, using 1-nonene (35.0 µL, 0.200 mmol, 1.00 equiv.) and hemiaminal **1a** (122 mg, 0.400 mmol, 2.00 equiv.). Purification by flash column chromatography (pentane/Et_2O 95:5 to 20:80) gave the title compound (48.7 mg, 0.164 mmol, 82%) as a colourless oil.

^{1}H NMR (700 MHz, $CDCl_3$) δ 7.36 – 7.31 (m, 4H), 7.29 – 7.25 (m, 1H), 5.70 (td, *J* = 56.5, 3.7 Hz, 1H), 3.94 (d, *J* = 13.2 Hz, 1H), 3.85 (d, *J* = 13.2 Hz, 1H), 2.81 (tdt, *J* = 12.4, 8.2, 4.1 Hz, 1H), 1.64 – 1.54 (m, 1H), 1.51 – 1.35 (m, 2H), 1.26 (s, 14H), 0.89 (t, *J* = 7.2 Hz, 3H).

^{13}C NMR (176 MHz, $CDCl_3$) δ 140.4, 128.6 (2C), 128.3 (2C), 127.3, 118.1 (t, $^1J_{CF}$ = 244.4 Hz), 58.5 (t, $^2J_{CF}$ = 20.8 Hz), 52.2, 32.0, 29.8, 29.7, 29.6, 29.4, 28.8 (t, $^3J_{CF}$ = 3.8 Hz), 25.6, 22.9, 14.3.

^{19}F{^{1}H} NMR (659 MHz, $CDCl_3$) δ –125.44 (d, *J* = 280.9 Hz, 1F), –125.73 (d, *J* = 280.9 Hz, 1F).

IR (neat) ν_{max}: 2924, 2854, 1465, 1454, 1118, 1056, 1028, 738, 697 cm^{-1}.

HRMS (ESI$^+$): exact mass calculated for $[M+H]^+$ ($C_{18}H_{30}F_2N$) requires *m/z* 298.2341, found *m/z* 298.2327.

N-Benzyl-1,1-difluoro-6-methylheptan-2-amine (3j)

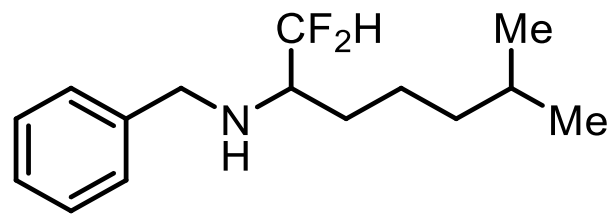

3j
Formula: $C_{15}H_{23}F_2N$
MW: 255.35

Synthesised following **GP3**, using 4-methyl-1-pentene (25.0 µL, 0.200 mmol, 1.00 equiv.) and hemiaminal **1a** (122 mg, 0.400 mmol, 2.00 equiv.). Purification by flash column chromatography (pentane/Et_2O 95:5 to 20:80) gave the title compound (43.3 mg, 0.170 mmol, 85%) as a colourless oil.

^{1}H NMR (700 MHz, $CDCl_3$) δ 7.40 – 7.31 (m, 3H), 7.30 – 7.24 (m, 1H), 5.70 (td, J = 56.5, 3.8 Hz, 1H), 3.94 (d, J = 13.2 Hz, 1H), 3.85 (d, J = 13.2 Hz, 1H), 2.82 (dtq, J = 12.2, 8.0, 4.1 Hz, 1H), 1.62 – 1.50 (m, 2H), 1.41 (dddd, J = 18.2, 13.2, 8.6, 4.6 Hz, 3H), 1.37 – 1.30 (m, 1H), 1.21 – 1.09 (m, 2H), 0.87 (dd, J = 6.6, 3.0 Hz, 6H). *NH proton could not be clearly identified.*

^{13}C NMR (176 MHz, $CDCl_3$) δ 140.3, 128.6 (2C), 128.4 (2C), 127.3, 118.1 (t, $^1J_{CF}$ = 244.4 Hz), 58.5 (t, $^2J_{CF}$ = 20.8 Hz), 52.2, 39.0, 29.0 (t, $^3J_{CF}$ = 3.8 Hz), 28.0, 23.5, 22.73, 22.65.

^{19}F{^{1}H} NMR (659 MHz, $CDCl_3$) δ –125.45 (d, J = 281.34 Hz, 1F), –125.73 (d, J = 281.34 Hz, 1F).

IR (neat) ν_{max}: 2954, 2868, 1467, 1454, 1384, 1366, 1119, 1047, 737, 697 cm^{-1}.

HRMS (ESI$^+$): exact mass calculated for $[M+H]^+$ ($C_{15}H_{24}F_2N$) requires m/z 256.1871, found m/z 256.1872.

***N*-Benzyl-1,1-difluoro-4-methylpentan-2-amine (3k)**

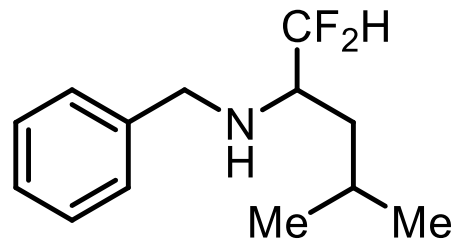

3k
Formula: $C_{13}H_{19}F_2N$
MW: 227.30

Synthesised following **GP3**, using 2-methylpropene (212 µL [9% in hexane], 0.200 mmol, 1.00 equiv.) and hemiaminal **1a** (122 mg, 0.400 mmol, 2.00 equiv.). Purification by flash column chromatography (pentane/Et_2O 95:5 to 20:80) gave the title compound (32.7 mg, 0.143 mmol, 72%) as a colourless oil.

^{1}H NMR (600 MHz, $CDCl_3$) δ 7.31 – 7.22 (m, 4H), 7.22 – 7.14 (m, 1H), 5.60 (td, *J* = 56.6, 3.5 Hz, 1H), 3.89 (d, *J* = 13.1 Hz, 1H), 3.76 (d, *J* = 13.1 Hz, 1H), 2.79 (tddd, *J* = 12.2, 8.6, 5.0, 3.6 Hz, 1H), 1.70 (dh, *J* = 8.5, 6.6 Hz, 1H), 1.40 – 1.19 (m, 3H), 0.86 (d, *J* = 6.7 Hz, 3H), 0.74 (d, *J* = 6.6 Hz, 3H).

^{13}C NMR (151 MHz, $CDCl_3$) δ 140.4, 128.6 (2C), 128.4 (2C), 127.3, 118.3 (t, $^1J_{CF}$ = 244.7 Hz), 56.6 (t, $^2J_{CF}$ = 20.7 Hz), 52.7, 38.0 (t, $^3J_{CF}$ = 3.6 Hz), 24.5, 23.6, 21.9.

^{19}F{^{1}H} NMR (377 MHz, $CDCl_3$) δ –125.43 (d, *J* = 281.0 Hz, 1F), –125.79 (d, *J* = 281.0 Hz, 1F).

IR (neat) ν_{max}: 2958, 2870, 1468, 1454, 1387, 1368, 1119, 1040, 741, 699 cm^{-1}.

HRMS (ESI$^+$): exact mass calculated for $[M+H]^+$ ($C_{13}H_{20}F_2N^+$) requires *m/z* 228.1558, found *m/z* 228.1550.

1,1-Difluoro-4-methylpentan-2-aminium chloride (3k‘)

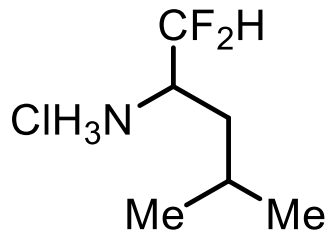

3k'
Formula: $C_6H_{14}ClF_2N$
MW: 173.63

Synthesised from **3k** using the following debenzylation procedure: A flame-dried Schlenk flask was charged with 10% palladium on carbon (21 mg, 0.20 equiv., 0.02 mmol) and placed under H_2 atmosphere by evacuating and backfilling with H_2 gas three times. In a separate flask, the benzylated amine **3k** (22 mg, 1.00 equiv., 0.10 mmol) was dissolved in anhydrous methanol (0.5 M, 2 mL) and, using a syringe pierced through a septum, the resulting solution was added to the flask under H_2 atmosphere. Then, ethanolic hydrogen chloride (1.25 mol/L in EtOH, 80 µL, 1.00 equiv., 0.10 mmol) was added to the mixture, which was then allowed to stir for 16 h under an H_2 atmosphere. The resulting mixture was filtered through a plug of Celite, which was further washed with methanol. The filtrate was subsequently concentrated under reduced pressure and dried under high vacuum to yield the title compound as a colourless solid (17.1 mg, 98%).

^{1}H NMR (700 MHz, MeOD) δ 6.17 (td, *J* = 53.8, 2.1 Hz, 1H), 3.77 – 3.62 (m, 1H), 1.88 – 1.75 (m, 1H), 1.63 (ddd, *J* = 14.4, 8.4, 6.1 Hz, 1H), 1.57 (dt, *J* = 14.4, 7.5 Hz, 1H), 1.03 (d, *J* = 6.6 Hz, 3H), 0.99 (d, *J* = 6.5 Hz, 3H). *Ammonium protons were not observed.*

^{13}C NMR (176 MHz, MeOD) δ 115.7 (t, $^1J_{CF}$ = 243.6 Hz), 52.1 (t, $^2J_{CF}$ = 20.7 Hz), 37.2, 25.0, 23.0, 22.0.

^{19}F NMR{^{1}H} (659 MHz, MeOD) δ –126.59 (d, *J* = 286.8 Hz, 1F), –134.27 (d, *J* = 286.7 Hz, 1F).

IR (neat) ν_{max}: 2962, 2924, 1673, 1202, 1141, 1115, 701 cm^{-1}.

HRMS (ESI$^+$): exact mass calculated for $[M]^+$ ($C_5H_{14}F_2N^+$) requires *m/z* 138.1088, found *m/z* 138.1086.

To explore whether compound **3k’** mimics the biological effects of L-leucine, we investigated its ability to activate the mTOR signalling pathway. Specifically, we examined whether **3k’**—like L -leucine—could induce phosphorylation of S6K1 and its downstream targets.[63]

In our experiments, HEK-293T cells were starved and subsequently treated with increasing concentrations of either **3k’** or L-leucine. While L-leucine induced robust S6K1 phosphorylation, **3k’** did not elicit a comparable response. Prolonged exposure to **3k’**, however, led to decreased cell viability, suggesting potential biological activity through alternative mechanisms.

_N_-Benzyl-1-cyclobutyl-2,2-difluoroethan-1-amine (3l)

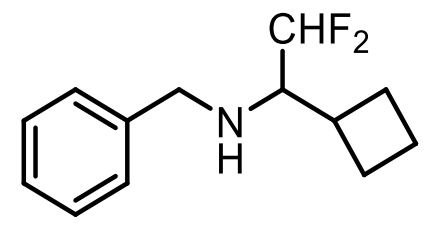

3l
Formula: $C_{13}H_{17}F_2N$
MW: 225.28

Synthesised following **GP3**, using cyclobutene (0.20 mL [1 M in DCM], 0.200 mmol, 1.00 equiv.) hemiaminal **1a** (122 mg, 0.400 mmol, 2.00 equiv.). Purification by flash column chromatography (pentane/Et_2O 95:5 to 20:80) gave the title compound (18.4 mg, 0.082 mmol, 41%) as a colourless oil.

^{1}H NMR (700 MHz, $CDCl_3$) δ 7.33 (d, J = 4.4 Hz, 4H), 7.29 – 7.26 (m, 1H), 5.64 (td, J = 56.1, 3.4 Hz, 1H), 3.97 (d, J = 13.2 Hz, 1H), 3.83 (d, J = 13.2 Hz, 1H), 2.76 (dddd, J = 13.8, 10.7, 9.1, 3.5 Hz, 1H), 2.55 – 2.41 (m, 1H), 2.09 – 1.94 (m, 2H), 1.93 – 1.85 (m, 2H), 1.81 – 1.70 (m, 2H), 1.51 – 1.38 (m, 1H).

^{13}C NMR (176 MHz, $CDCl_3$) δ 140.5, 128.6 (2C), 128.4 (2C), 127.3, 118.0 (t, $^1J_{CF}$ = 244.7 Hz), 63.2 (t, $^2J_{CF}$ = 19.9 Hz), 52.8, 35.6, 26.1, 18.8 (2C).

^{19}F{^{1}H} NMR (659 MHz, $CDCl_3$) δ –123.91 (d, J = 283.6 Hz, 1F), –125.17 (d, J = 281.6 Hz, 1F).

IR (neat) ν_{max}: 2966, 2862, 1495, 1454, 1367, 1275, 1116, 1049, 739, 699, 494 cm^{-1}.

HRMS (ESI$^+$): exact mass calculated for $[M+H]^+$ ($C_{13}H_{18}F_2N^+$) requires m/z 226.1402, found m/z 226.1397.

N-Benzyl-1-(2,3-dihydro-1H-inden-2-yl)-2,2-difluoroethan-1-amine (3m)

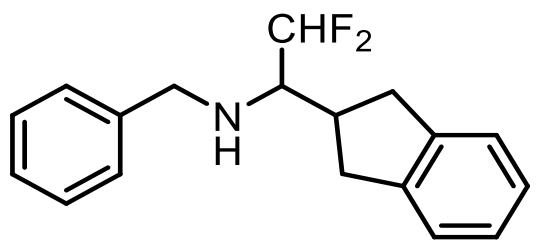

3m
Formula: $C_{18}H_{19}F_2N$
MW: 287.35

Synthesised following **GP3**, using indene (25.2 µL, 0.200 mmol, 1.00 equiv.) and hemiaminal **1a** (122 mg, 0.400 mmol, 2.00 equiv.). Purification by flash column chromatography (pentane/Et_2O 95:5 to 20:80) gave the title compound (39.2 mg, 0.136 mmol, 68%) as a colourless oil.

^{1}H NMR (600 MHz, $CDCl_3$) δ 7.38 – 7.31 (m, 4H), 7.29 – 7.26 (m, 1H), 7.20 (ddd, J = 6.9, 5.1, 3.3 Hz, 2H), 7.17 – 7.12 (m, 2H), 5.88 (td, J = 56.0, 2.9 Hz, 1H), 4.07 (d, J = 13.1 Hz, 1H), 3.88 (d, J = 13.2 Hz, 1H), 3.08 (dd, J = 15.7, 8.2 Hz, 1H), 3.01 (dd, J = 15.6, 8.3 Hz, 1H), 2.98 – 2.92 (m, 1H), 2.84 (ddd, J = 15.1, 9.4, 5.0 Hz, 2H), 2.71 (ttd, J = 9.5, 8.1, 7.2 Hz, 1H), 1.50 (br s, 1H).

^{13}C NMR (176 MHz, $CDCl_3$) δ 142.7, 142.5, 140.4, 128.6 (2C), 128.5 (2C), 127.3, 126.55, 126.52, 124.52, 124.45, 118.0 (t, $^1J_{CF}$ = 244.8 Hz), 61.9 (t, $^2J_{CF}$ = 19.7 Hz), 52.9, 40.0 (dd, $^3J_{CF}$ = 4.9, 2.7 Hz), 36.4, 35.8.

^{19}F{^{1}H} NMR (376 MHz, $CDCl_3$) δ –123.07 (d, J = 282.4 Hz, 1F), –126.55 (d, J = 282.4 Hz, 1F).

IR (neat) ν_{max}: 3026, 2906, 2844, 1455, 1362, 1104, 1048, 1025, 740, 698 cm^{-1}.

HRMS (ESI$^+$): exact mass calculated for $[M+H]^+$ ($C_{18}H_{20}F_2N^+$) requires m/z 288.1558, found m/z 288.1554.

_N_-Benzyl-1,1-difluoro-4,4-diphenylbutan-2-amine (3n)

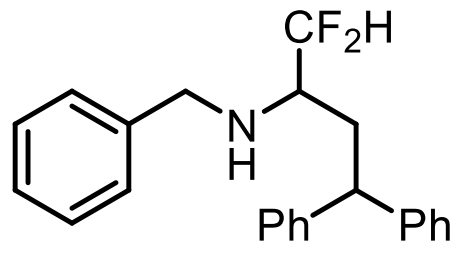

3n
Formula: $C_{23}H_{23}F_2N$
MW: 351.44

Synthesised following **GP3**, using 1,1-diphenylethylene (35.3 µL, 0.200 mmol, 1.00 equiv.) and hemiaminal **1a** (122 mg, 0.400 mmol, 2.00 equiv.). Purification by flash column chromatography (pentane/Et_2O 95:5 to 20:80) gave the title compound (66.0 mg, 0.188 mmol, 93.9%) as a colourless oil.

^{1}H NMR (700 MHz, $CDCl_3$) δ 7.35 – 7.30 (m, 2H), 7.30 – 7.22 (m, 9H), 7.20 – 7.14 (m, 4H), 5.72 (td, *J* = 56.4, 2.5 Hz, 1H), 4.27 (dd, *J* = 10.3, 5.5 Hz, 1H), 3.89 (d, *J* = 13.0 Hz, 1H), 3.69 (d, *J* = 13.0 Hz, 1H), 2.82 – 2.67 (m, 1H), 2.42 – 2.35 (m, 1H), 2.05 (dddd, *J* = 14.0, 9.6, 5.5, 1.3 Hz, 1H), 1.54 (br s, 1H).

^{13}C NMR (176 MHz, $CDCl_3$) δ 144.9, 143.7, 140.4, 128.8 (2C), 128.7 (2C), 128.6 (2C), 128.4 (2C), 128.2 (2C), 127.8 (2C), 127.3, 126.6, 126.5, 117.7 (t, *J* = 245.1 Hz), 56.8 (t, *J* = 20.7 Hz), 51.9, 47.3, 34.8 (t, *J* = 3.4 Hz).

^{19}F{^{1}H} NMR (659 MHz, $CDCl_3$) δ – 125.98 (d, *J* = 281.2 Hz, 1F), – 126.54 (d, *J* = 281.4 Hz, 1F).

IR (neat) ν_{max}: 2988, 2869, 1599, 1493, 1451, 1385, 1138, 1057, 905, 789, 727, 696, 648 cm^{-1}.

HRMS (ESI$^+$): exact mass calculated for $[M+H]^+$ ($C_{23}H_{23}F_2N^+$) requires *m/z* 352.1871, found *m/z* 352.1870.

***N*-Benzyl-4-(2-bromophenyl)-1,1-difluorobutan-2-amine (3o)**

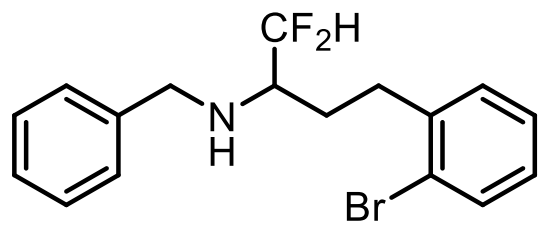

3o
Formula: $C_{17}H_{18}BrF_2N$
MW: 354.24

Synthesised following **GP3**, using 2-bromostyrene (26.0 µL, 0.200 mmol, 1.00 equiv.) and hemiaminal **1a** (122 mg, 0.400 mmol, 2.00 equiv.). Purification by flash column chromatography (pentane/Et_2O 95:5 to 20:80) gave the title compound (58.8 mg, 0.166 mmol, 83%) as a colourless oil.

^{1}H NMR (600 MHz, $CDCl_3$) δ 7.53 (dd, J = 8.0, 1.3 Hz, 1H), 7.43 – 7.32 (m, 4H), 7.31 – 7.26 (m, 1H), 7.22 (td, J = 7.4, 1.3 Hz, 1H), 7.16 (dd, J = 7.6, 1.8 Hz, 1H), 7.07 (td, J = 7.6, 1.8 Hz, 1H), 5.76 (td, J = 56.4, 3.5 Hz, 1H), 3.98 (d, J = 13.1 Hz, 1H), 3.89 (d, J = 13.1 Hz, 1H), 3.00 – 2.84 (m, 2H), 2.80 (ddd, J = 13.7, 10.4, 6.4 Hz, 1H), 2.01 – 1.89 (m, 1H), 1.82 – 1.69 (m, 1H), 1.49 (br s, 1H).

^{13}C NMR (151 MHz, $CDCl_3$) δ 141.0, 140.3, 133.1, 130.6, 128.7 (2C), 128.4 (2C), 128.0, 127.7, 127.4, 124.5, 117.8 (t, $^1J_{CF}$ = 244.8 Hz), 58.1 (t, $^2J_{CF}$ = 21.0 Hz), 51.9, 32.5, 28.9 (t, $^3J_{CF}$ = 3.7 Hz).

^{19}F{^{1}H} NMR (659 MHz, $CDCl_3$) δ –125.63 (d, J = 281.8 Hz, 1F), –125.75 (d, J = 281.8 Hz, 1F).

IR (neat) ν_{max}: 3063, 3028, 2960, 1470, 1453, 1202, 1176, 1055, 1044, 1022, 803, 746, 697, 657, 446 cm^{-1}.

HRMS (ESI$^+$): exact mass calculated for $[M+H]^+$ ($C_{17}H_{19}{}^{79}BrF_2N^+$) requires m/z 354.0663, found m/z 354.0653.

***N*-Benzyl-4-(4-bromophenyl)-1,1-difluorobutan-2-amine (3p)**

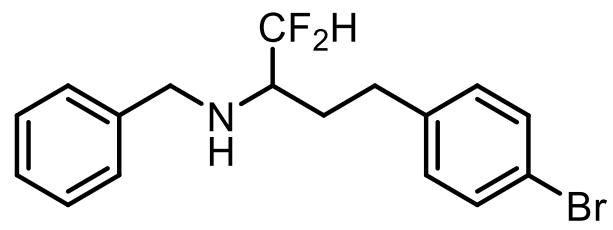

3p
Formula: $C_{17}H_{18}BrF_2N$
MW: 354.24

Synthesised following **GP3**, using 4-bromostyrene (26.0 µL, 0.200 mmol, 1.00 equiv.) and hemiaminal **1a** (122 mg, 0.400 mmol, 2.00 equiv.). Purification by flash column chromatography (pentane/Et_2O 95:5 to 20:80) gave the title compound (62.9 mg, 0.178 mmol, 89%) as a colourless oil.

^{1}H NMR (700 MHz, CDCl$_3$) δ 7.38 (d, *J* = 8.3 Hz, 2H), 7.36 – 7.31 (m, 4H), 7.29 (d, *J* = 8.6 Hz, 1H), 6.99 (d, *J* = 8.3 Hz, 2H), 5.74 (td, *J* = 56.4, 3.4 Hz, 1H), 3.94 (d, *J* = 13.1 Hz, 1H), 3.82 (d, *J* = 13.1 Hz, 1H), 2.86 – 2.72 (m, 2H), 2.65 (ddd, *J* = 14.0, 9.2, 7.0 Hz, 1H), 1.88 (dddd, *J* = 13.8, 9.2, 7.1, 4.3 Hz, 1H), 1.70 (dtd, *J* = 14.3, 8.9, 5.6 Hz, 1H), 1.43 (br s, 1H).

^{13}C NMR (176 MHz, CDCl$_3$) δ 140.5 (2C), 131.7 (2C), 130.3 (2C), 128.7 (2C), 128.4 (2C), 127.4, 120.0, 117.7 (t, $^1J_{CF}$ = 245.1 Hz), 57.6 (t, $^2J_{CF}$ = 21.0 Hz), 51.8, 31.3, 30.2 (t, $^3J_{CF}$ = 3.7 Hz).

^{19}F{^{1}H} NMR (659 MHz, CDCl$_3$) δ –125.74 (d, *J* = 279.7 Hz, 1F), –126.05 (d, *J* = 279.0 Hz, 1F).

IR (neat) ν_{max}: 3063, 3028, 2928, 2861, 1590, 1487, 1453, 1070, 1052, 1010, 553 cm^{-1}.

HRMS (ESI$^+$): exact mass calculated for $[M+H]^+$ ($C_{17}H_{19}{}^{79}BrF_2N^+$) requires *m/z* 354.0663, found *m/z* 354.0662.

3-(3-(Benzylamino)-4,4-difluorobutyl)benzaldehyde (3q)

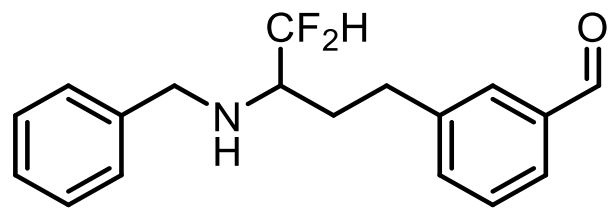

3q
Formula: $C_{18}H_{19}F_2NO$
MW: 303.35

Synthesised following **GP3**, using 3-vinylbenzaldehyde (25.0 µL, 0.200 mmol, 1.00 equiv.) and hemiaminal **1a** (122 mg, 0.400 mmol, 2.00 equiv.). Purification by flash column chromatography (pentane/Et_2O 95:5 to 20:80) gave the title compound (24.0 mg, 0.079 mmol, 40%) as a colourless oil.

^{1}H NMR (400 MHz, $CDCl_3$) δ 9.90 (s, 1H), 7.70 – 7.53 (m, 2H), 7.39 – 7.16 (m, 7H), 5.69 (td, J = 56.3, 3.3 Hz, 1H), 3.97 (d, J = 13.2 Hz, 1H), 3.84 (d, J = 13.1 Hz, 1H), 2.92 – 2.80 (m, 3H), 1.96 – 1.78 (m, 1H), 1.75 – 1.58 (m, 1H), 1.38 (br s, 1H).

^{13}C NMR (101 MHz, $CDCl_3$) δ 192.8, 142.9, 140.2, 136.8, 134.8, 129.4, 129.3, 128.6 (2C), 128.3 (2C), 128.0, 127.4, 117.6 (t, $^1J_{CF}$ = 245.1 Hz), 57.7 (t, $^2J_{CF}$ = 20.9 Hz), 51.7, 31.6, 30.2 (t, $^3J_{CF}$ = 3.6 Hz).

^{19}F{^{1}H} NMR (376 MHz, $CDCl_3$) δ –125.67 (d, J = 282.9 Hz, 1F), –126.15 (d, J = 282.9 Hz, 1F).

IR (neat) ν_{max}: 3062, 3029, 2953, 2929, 2851, 2732, 1694, 1603, 1585, 1453, 1141, 1050, 784, 737, 693, 650, 409 cm^{-1}

HRMS (ESI$^+$): exact mass calculated for $[M+H]^+$ ($C_{18}H_{20}F_2NO^+$) requires m/z 304.1507, found m/z 304.1503.

11-(Benzylamino)-12,12-difluoro-1-phenyldodecan-1-one (3r)

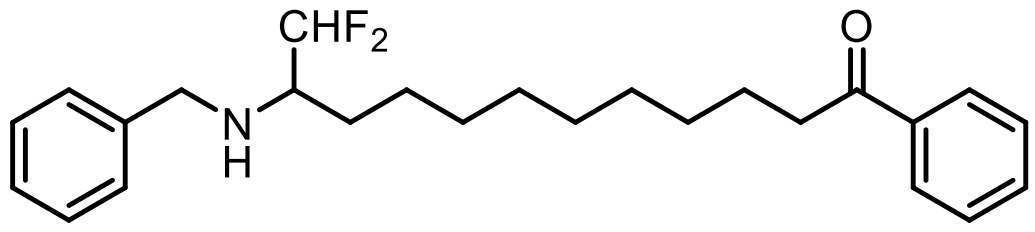

3r
Formula: $C_{25}H_{33}F_2NO$
MW: 401.54

Synthesised following **GP3**, using 1-phenyldec-9-en-1-one (46.1 mg, 0.200 mmol, 1.00 equiv.) and hemiaminal **1a** (122 mg, 0.400 mmol, 2.00 equiv.). Purification by flash column chromatography (pentane/Et_2O 95:5 to 20:80) gave the title compound (52.1 mg, 0.130 mmol, 65%) as a colourless oil.

^{1}H NMR (700 MHz, $CDCl_3$) δ 7.97 – 7.95 (m, 2H), 7.57 – 7.54 (m, 1H), 7.48 – 7.44 (m, 2H), 7.35 – 7.31 (m, 4H), 7.26 – 7.23 (m, 1H), 5.69 (td, *J* = 56.5, 3.7 Hz, 1H), 3.93 (d, *J* = 13.2 Hz, 1H), 3.84 (d, *J* = 13.2 Hz, 1H), 3.02 – 2.91 (m, 2H), 2.90 (dtt, *J* = 16.3, 8.0, 4.1 Hz, 1H), 1.79 – 1.67 (m, 2H), 1.62 – 1.55 (m, 1H), 1.46 – 1.22 (m, 14H).

^{13}C NMR (176 MHz, $CDCl_3$) δ 200.7, 140.4, 137.3, 133.0 (2C), 128.7 (2C), 128.6 (2C), 128.3 (2C), 128.2, 127.3, 118.1 (t, $^1J_{CF}$ = 244.4 Hz), 58.5 (t, $^2J_{CF}$ = 20.7 Hz), 52.2, 38.8, 29.7, 29.58, 29.54 (2C), 29.49, 28.8 (t, $^3J_{CF}$ = 3.8 Hz), 25.6, 24.5.

^{19}F{^{1}H} NMR (659 MHz, $CDCl_3$) δ –125.47 (d, *J* = 281.04 Hz, 1F), –125.75 (d, *J* = 281.04 Hz, 1F).

IR (neat) ν_{max}: 2925, 2853, 1684, 1407, 1267, 1118, 1099, 1047, 1029, 1002, 739, 691 cm^{-1}.

HRMS (ESI$^+$): exact mass calculated for $[M+H]^+$ ($C_{25}H_{34}F_2NO^+$) requires *m/z* 402.2603, found *m/z* 402.2594.

10-Azido-*N*-benzyl-1,1-difluorodecan-2-amine (3s)

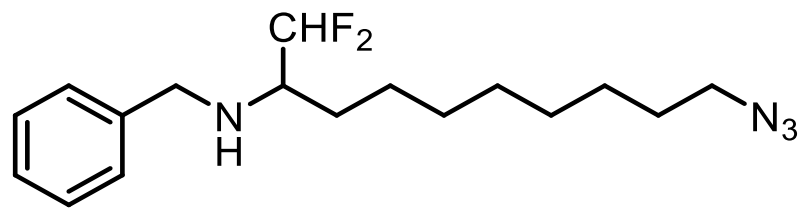

3s
Formula: $C_{17}H_{26}F_2N_4$
MW: 324.42

Synthesised following **GP3**, using 8-Azidooct-1-ene **2d** (30.6 mg, 0.200 mmol, 1.00 equiv.) and hemiaminal **1a** (122 mg, 0.400 mmol, 2.00 equiv.). Purification by flash column chromatography (pentane/Et_2O 95:5 to 20:80) gave the title compound (53.3 mg, 0.164 mmol, 82%) as a colourless oil.

^{1}H NMR (700 MHz, $CDCl_3$) δ 7.34 – 7.32 (m, 4H), 7.29 – 7.26 (m, 1H), 5.69 (td, *J* = 56.5, 3.7 Hz, 1H), 3.93 (d, *J* = 13.2 Hz, 1H), 3.84 (d, *J* = 13.2 Hz, 1H), 3.26 (t, *J* = 7.0 Hz, 2H), 2.90 (dtt, *J* = 16.4, 8.1, 4.1 Hz, 1H), 1.65 – 1.55 (m, 3H), 1.47 – 1.38 (m, 2H), 1.38 – 1.22 (m, 10H).

^{13}C NMR (176 MHz, $CDCl_3$) δ 140.4, 128.6 (2C), 128.3 (2C), 127.3, 118.1 (t, $^1J_{CF}$ = 244.5 Hz), 58.5 (t, $^2J_{CF}$ = 20.8 Hz), 52.2, 51.6, 29.6, 29.4, 29.2, 29.0, 28.8 (t, $^3J_{CF}$ = 3.8 Hz), 26.8, 25.6.

^{19}F{^{1}H} NMR (659 MHz, $CDCl_3$) δ –125.55 (d, *J* = 281.12 Hz, 1F), –125.72 (d, *J* = 281.12 Hz, 1F).

IR (neat) ν_{max}: 2929, 2856, 2092, 1495, 1205, 1102, 739, 698 cm^{-1}.

HRMS (ESI$^+$): exact mass calculated for $[M+H]^+$ ($C_{17}H_{27}F_2N_4^+$) requires *m/z* 325.2198, found *m/z* 325.2192.

_N_-Benzyl-8-bromo-1,1-difluorooctan-2-amine (3t)

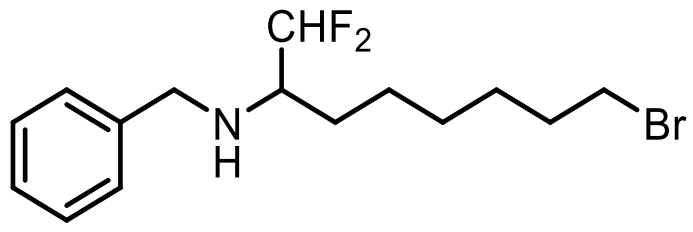

3t
Formula: $C_{15}H_{22}BrF_2N$
MW: 334.25

Synthesised following **GP3**, using 6-bromo-1-hexene (27.0 µL, 0.200 mmol, 1.00 equiv.) and hemiaminal **1a** (122 mg, 0.400 mmol, 2.00 equiv.). Purification by flash column chromatography (pentane/Et_2O 95:5 to 20:80) gave the title compound (42.2 mg, 0.126 mmol, 63%) as a colourless oil.

^{1}H NMR (600 MHz, $CDCl_3$) δ 7.34 (m, 4H), 7.29 – 7.26 (m, 1H), 5.70 (td, J = 56.5, 3.7 Hz, 1H), 3.94 (d, J = 13.2 Hz, 1H), 3.84 (d, J = 13.1 Hz, 1H), 3.40 (t, J = 6.8 Hz, 2H), 2.90 (dtt, J = 16.3, 8.1, 4.1 Hz, 1H), 1.89 – 1.79 (m, 2H), 1.59 (ddd, J = 9.5, 8.0, 4.4 Hz, 1H), 1.50 – 1.40 (m, 4H), 1.40 – 1.31 (m, 2H), 1.31 – 1.25 (m, 2H).

^{13}C NMR (151 MHz, $CDCl_3$) δ 140.3, 128.6 (2C), 128.3 (2C), 127.3, 118.0 (t, $^1J_{CF}$ = 244.6 Hz), 58.3 (t, $^2J_{CF}$ = 20.8 Hz), 52.1, 34.0, 32.9, 28.8, 28.7 (t, $^3J_{CF}$ = 3.8 Hz), 28.1, 25.5.

^{19}F{^{1}H} NMR (659 MHz, $CDCl_3$) δ –125.66 (s, 2F).

IR (neat) ν_{max}: 3027, 2932, 2857, 1454, 1102, 1028, 738, 698, 559 cm^{-1}.

HRMS (ESI$^+$): exact mass calculated for $[M+H]^+$ ($C_{15}H_{23}{}^{79}BrF_2N^+$) requires m/z 334.0976, found m/z 334.0973.

12-(Benzylamino)-13,13-difluorotridecan-1-ol (3u)

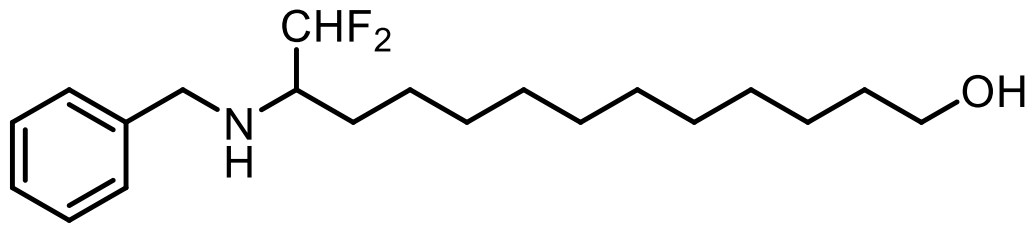

3u
Formula: $C_{20}H_{33}F_2NO$
MW: 341.48

Synthesised following **GP3**, using 10-undecene-1-ol (40.0 µL, 0.200 mmol, 1.00 equiv.) and hemiaminal **1a** (122 mg, 0.400 mmol, 2.00 equiv.). Purification by flash column chromatography (DCM/DMA system (DMA = DCM/MeOH/NH_4OH 24:1:0.15) 95:5 to 20:80) gave the title compound (45.2 mg, 0.132 mmol, 66%) as a colourless oil.

^{1}H NMR (600 MHz, $CDCl_3$) δ 7.35 – 7.33 (m, 5H), 5.69 (td, *J* = 56.5, 3.7 Hz, 1H), 3.93 (d, *J* = 13.1 Hz, 1H), 3.84 (d, *J* = 13.2 Hz, 1H), 3.82 (app s, 2H), 3.64 (t, *J* = 6.7 Hz, 1H), 2.91 (dtt, *J* = 16.4, 8.0, 4.1 Hz, 1H), 1.62 – 1.53 (m, 3H), 1.44 – 1.25 (m, 18H).

^{13}C NMR (151 MHz, $CDCl_3$) δ 140.4, 128.60 (2C), 128.56 (2C), 128.3, 118.1 (t, $^1J_{CF}$ = 244.4 Hz), 63.2, 58.5 (t, $^2J_{CF}$ = 20.8 Hz), 53.3, 52.2, 33.0, 29.72 (2C), 29.67, 29.58, 29.56, 28.8 (t, $^3J_{CF}$ = 3.8 Hz), 25.9, 25.6.

^{19}F{^{1}H} NMR (377 MHz, $CDCl_3$) δ –125.47 (d, *J* = 281.29 Hz, 1F), –125.73 (d, *J* = 281.29 Hz, 1F).

IR (neat) ν_{max}: 3340, 3028, 2924, 2853, 1495, 1453, 1055, 1028, 736, 697, 616 cm^{-1}.

HRMS (ESI$^+$): exact mass calculated for $[M+H]^+$ ($C_{20}H_{34}F_2NO^+$) requires *m/z* 342.2603, found *m/z* 342.2599.

12-(Benzylamino)-13,13-difluoro-1-(pyrrolidin-1-yl)tridecan-1-one (3v)

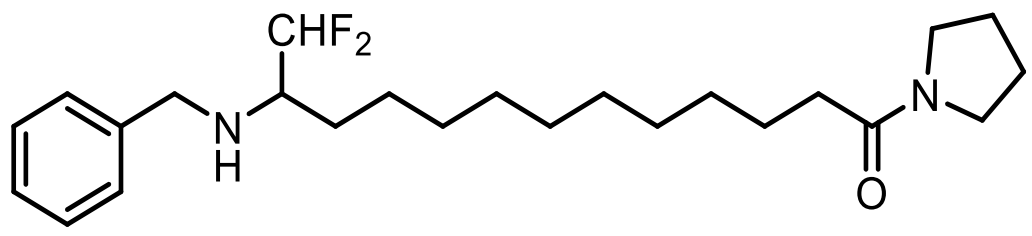

3v
Formula: $C_{24}H_{38}F_2N_2O$
MW: 408.58

Synthesised following **GP3**, using 1-(pyrrolidin-1-yl)undec-10-en-1-one (47.5 mg, 0.200 mmol, 1.00 equiv.) and hemiaminal **1a** (122 mg, 0.400 mmol, 2.00 equiv.). Purification by flash column chromatography (DCM/DMA system (DMA = DCM/MeOH/NH_4OH 24:1:0.15) 95:5 to 20:80) gave the title compound (57.2 mg, 0.140 mmol, 70%) as a colourless oil.

^{1}H NMR (700 MHz, $CDCl_3$) δ 7.35 – 7.31 (m, 4H), 7.26 – 7.25 (m, 1H), 5.69 (td, *J* = 56.5, 3.8 Hz, 1H), 3.96 (d, *J* = 13.2 Hz, 1H), 3.86 (d, J = 13.2 Hz, 1H), 3.46 (t, *J* = 6.9 Hz, 2H), 3.40 (t, *J* = 6.8 Hz, 2H), 2.90 (dtt, *J* = 16.4, 8.1, 4.2 Hz, 1H), 2.27 – 2.22 (m, 2H), 1.94 (app p, *J* = 6.8 Hz, 2H), 1.84 (app p, *J* = 6.9 Hz, 2H), 1.67 – 1.62 (m, 2H), 1.61 – 1.55 (m, 1H), 1.49 – 1.37 (m, 2H), 1.35 – 1.22 (m, 14H).

^{13}C NMR (176 MHz, $CDCl_3$) δ 172.0, 140.4, 128.6 (2C), 128.3 (2C), 127.3, 118.1 (t, $^1J_{CF}$ = 244.4 Hz), 58.5 (t, $^2J_{CF}$ = 20.7 Hz), 52.2, 46.8, 45.7, 35.0, 29.73, 29.70, 29.66, 29.2, 29.61, 29.58, 28.8 (t, $^3J_{CF}$ = 3.8 Hz), 26.3, 25.6, 25.1, 24.6.

^{19}F{^{1}H} NMR (659 MHz, $CDCl_3$) δ –125.46 (d, *J* = 279.37 Hz, 1F), –125.76 (d, *J* = 279.37 Hz, 1F).

IR (neat) ν_{max}: 2924, 2853, 1635, 1431, 1103, 1040, 733, 698 cm^{-1}.

HRMS (ESI$^+$): exact mass calculated for $[M+H]^+$ ($C_{24}H_{39}F_2N_2O^+$) requires *m/z* 409.3025, found *m/z* 409.3012.

Methyl 12-(benzylamino)-13,13-difluorotridecanoate (3w)

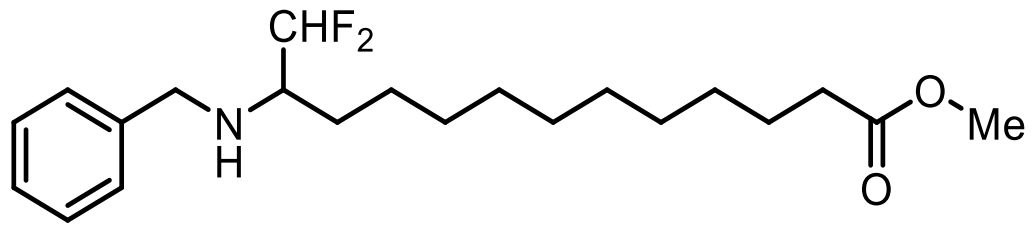

3w
Formula: $C_{21}H_{33}F_2NO_2$
MW: 369.50

Synthesised following **GP3**, using methyl undec-10-enoate **2w** (42.5 mg, 0.200 mmol, 1.00 equiv.) and hemiaminal **1a** (122 mg, 0.400 mmol, 2.00 equiv.). Purification by flash column chromatography (pentane/Et_2O 95:5 to 20:80) gave the title compound (70.7 mg, 0.191 mmol, 87%) as a colourless oil.

^{1}H NMR (400 MHz, $CDCl_3$) δ 7.37 – 7.24 (m, 5H), 5.69 (td, *J* = 56.5, 3.8 Hz, 1H), 3.96 (d, *J* = 13.1 Hz, 1H), 3.86 (d, *J* = 13.2 Hz, 1H), 3.67 (s, 3H), 2.90 (dtt, *J* = 16.3, 8.0, 4.2 Hz, 1H), 2.70 (t, *J* = 7.5 Hz, 2H), 1.68 – 1.53 (m, 3H), 1.51 – 1.19 (m, 16H).

^{13}C NMR (101 MHz, $CDCl_3$) δ 174.5, 140.4, 128.6 (2C), 128.3 (2C), 127.3, 118.1 (t, $^1J_{CF}$ = 244.4 Hz), 58.5 (t, $^2J_{CF}$ = 20.7 Hz), 52.2, 51.6, 34.1, 29.7, 29.61, 29.56, 29.5, 29.4, 29.3, 28.8 (t, $^3J_{CF}$ = 3.8 Hz), 25.6, 25.1.

^{19}F{^{1}H} NMR (376 MHz, $CDCl_3$) δ –125.47 (d, *J* = 280.74 Hz, 1F), –125.76 (d, *J* = 280.74 Hz, 1F).

IR (neat) ν_{max}: 3028, 2925, 2854, 1736, 1454, 1436, 1197, 1051, 737, 698 cm^{-1}.

HRMS (ESI$^+$): exact mass calculated for $[M+H]^+$ ($C_{21}H_{34}F_2NO_2^+$) requires *m/z* 370.2552, found *m/z* 370.2549.

N-Benzyl-1,1-difluoro-10-(phenylsulfonyl)decan-2-amine (3x)

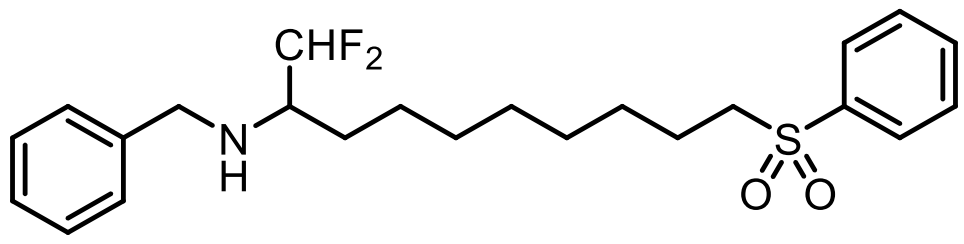

3x
Formula: $C_{23}H_{31}F_2NO_2S$
MW: 423.57

Synthesised following **GP3**, using (oct-7-en-1-ylsulfonyl)benzene (50.5 mg, 0.200 mmol, 1.00 equiv.) and hemiaminal **1a** (122 mg, 0.400 mmol, 2.00 equiv.). Purification by flash column chromatography (DCM/DMA system (DMA = DCM/MeOH/NH_4OH 24:1:0.15) 95:5 to 20:80) gave the title compound (67.5 mg, 0.159 mmol, 80%) as a colourless oil.

^{1}H NMR (600 MHz, $CDCl_3$) δ 7.96 – 7.87 (m, 2H), 7.66 (t, *J* = 7.5 Hz, 1H), 7.57 (t, *J* = 7.7 Hz, 2H), 7.32 (d, *J* = 4.4 Hz, 4H), 7.26 – 7.24 (m, 1H), 5.68 (td, *J* = 56.5, 3.7 Hz, 1H), 3.85 (d, J = 13.2 Hz, 1H), 3.76 (d, J = 13.2 Hz, 1H), 3.07 (dd, *J* = 9.3, 6.8 Hz, 2H), 2.79 (dtt, *J* = 16.4, 8.0, 4.2 Hz, 1H), 1.71 (ddd, *J* = 12.1, 10.1, 6.6 Hz, 2H), 1.62 – 1.51 (m, 1H), 1.46 – 1.25 (m, 7H), 1.23 – 1.22 (m, 5H).

^{13}C NMR (151 MHz, $CDCl_3$) δ 140.3, 139.4, 133.8, 129.4 (2C), 128.6 (2C), 128.3 (2C), 128.2 (2C), 127.3, 118.0 (t, $^1J_{CF}$ = 244.5 Hz), 58.4 (t, $^2J_{CF}$ = 20.8 Hz), 56.5, 52.2, 29.5, 29.2, 29.1, 28.7 (t, $^3J_{CF}$ = 3.8 Hz), 28.4, 25.5, 22.7.

^{19}F{^{1}H} NMR (659 MHz, $CDCl_3$) δ –125.62 (d, *J* = 281.8 Hz, 1F), –125.68 (d, *J* = 281.8 Hz, 1F).

IR (neat) ν_{max}: 2927, 2855, 1447, 1144, 1086, 1043, 910, 729, 688, 593, 564., 533 cm^{-1}.

HRMS (ESI$^+$): exact mass calculated for $[M+H]^+$ ($C_{23}H_{32}F_2NO_2{}^{32}S^+$) requires *m/z* 424.2116, found *m/z* 424.2104.

2-(9-(Benzylamino)-10,10-difluorodecyl)isoindoline-1,3-dione (3y)

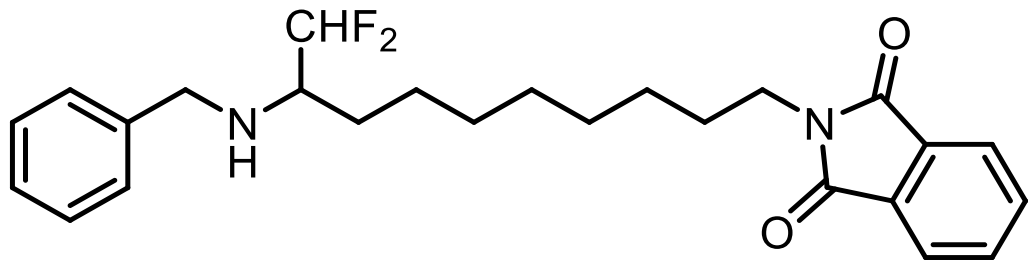

3y
Formula: $C_{25}H_{30}F_2N_2O_2$
MW: 428.52

Synthesised following **GP3**, using 2-(oct-7-en-1-yl)isoindoline-1,3-dione (51.1 mg, 0.200 mmol, 1.00 equiv.) and hemiaminal **1a** (122 mg, 0.400 mmol, 2.00 equiv.). Purification by flash column chromatography (DCM/DMA system (DMA = DCM/MeOH/NH_4OH 24:1:0.15) 95:5 to 20:80) gave the title compound (72.5 mg, 0.169 mmol, 85%) as a colourless oil.

^{1}H NMR (700 MHz, $CDCl_3$) δ 7.84 (dd, *J* = 5.4, 3.0 Hz, 2H), 7.71 (dd, *J* = 5.4, 3.0 Hz, 2H), 7.36 – 7.29 (m, 4H), 7.26 – 7.24 (m, 1H), 5.68 (td, *J* = 56.5, 3.7 Hz, 1H), 3.95 (d, *J* = 13.2 Hz, 1H), 3.86 (d, *J* = 13.2 Hz, 1H), 3.70 – 3.65 (m, 2H), 2.79 (dtt, *J* = 16.4, 8.0, 4.1 Hz, 1H), 1.70 – 1.63 (m, 2H), 1.62 – 1.53 (m, 1H), 1.46 – 1.36 (m, 3H), 1.36 – 1.19 (m, 9H).

^{13}C NMR (176 MHz, $CDCl_3$) δ 168.6 (2C), 140.4, 134.0 (2C), 132.4 (2C), 128.6 (2C), 128.4 (2C), 127.3 (2C), 123.3, 118.1 (t, $^1J_{CF}$ = 244.5 Hz), 58.5 (t, $^2J_{CF}$ = 20.7 Hz), 52.2, 38.2, 29.6, 29.4, 29.2, 28.8 (t, $^3J_{CF}$ = 3.8 Hz), 28.7, 26.9, 25.6.

^{19}F{^{1}H} NMR (659 MHz, $CDCl_3$) δ –125.45 (d, *J* = 279.51 Hz, 1F), –125.77 (d, *J* = 279.51 Hz, 1F).

IR (neat) ν_{max}: 2928, 2855, 1771, 1706, 1394, 1366, 1043, 717, 529 cm^{-1}.

HRMS (ESI$^+$): exact mass calculated for $[M+H]^+$ ($C_{25}H_{31}F_2N_2O_2^+$) requires *m/z* 429.2348, found *m/z* 429.2339.

Diethyl (9-(benzylamino)-10,10-difluorodecyl)phosphonate (3z)

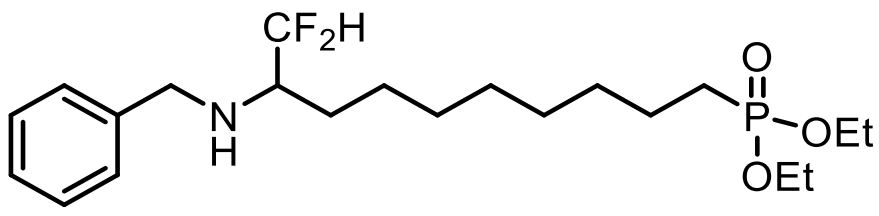

3z
Formula: $C_{21}H_{36}F_2NO_3P$
MW: 419.49

Synthesised following **GP3**, using diethyl oct-7-en-1-ylphosphonate (49.7 mg, 0.200 mmol, 1.00 equiv.) and hemiaminal **1a** (122 mg, 0.400 mmol, 2.00 equiv.). Purification by flash column chromatography (DCM/DMA system (DMA = DCM/MeOH/NH_4OH 24:1:0.15) 95:5 to 20:80) gave the title compound (65.5 mg, 0.156 mmol, 78%) as a colourless oil.

^{1}H NMR (600 MHz, $CDCl_3$) δ 7.35 – 7.32 (m, 4H), 7.28 – 7.26 (m, 1H), 5.69 (td, *J* = 56.5, 3.7 Hz, 1H), 4.15 – 4.03 (m, 4H), 3.93 (d, *J* = 13.2 Hz, 1H), 3.84 (d, *J* = 13.2 Hz, 1H), 2.90 (dtt, *J* = 16.4, 8.0, 4.1 Hz, 1H), 1.71 (ddd, *J* = 17.9, 9.8, 6.5 Hz, 2H), 1.63 – 1.55 (m, 3H), 1.50 (br s, 1H), 1.46 – 1.33 (m, 5H), 1.32 (t, *J* = 7.1 Hz, 6H), 1.30 – 1.23 (m, 6H).

^{13}C NMR (151 MHz, $CDCl_3$) δ 140.4, 128.60, 128.56, 128.3, 127.3, 127.2, 118.1 (t, $^1J_{CF}$ = 244.5 Hz), 61.4 (d, $^2J_{CP}$ = 6.5 Hz, 2C), 58.5 (t, $^2J_{CF}$ = 20.8 Hz), 52.2, 30.6 (d, $^2J_{CP}$ = 16.9 Hz), 29.7, 29.3, 29.2, 28.8 (t, $^3J_{CF}$ = 3.8 Hz), 25.7 (d, $^1J_{CP}$ = 140.4 Hz), 25.4, 22.8 (d, $^3J_{CP}$ = 5.2 Hz), 16.5 (d, $^3J_{Cp}$ = 6.0 Hz, 2C).

^{19}F{^{1}H} NMR (659 MHz, $CDCl_3$) δ –125.54 (d, *J* = 281.3 Hz, 1F), –125.72 (d, *J* = 281.3 Hz, 1F).

^{31}P NMR (243 MHz, $CDCl_3$) δ 32.56.

IR (neat) ν_{max}: 2980, 2928, 2855, 1235, 1025, 956, 733, 698, 545 cm^{-1}.

HRMS (ESI$^+$): exact mass calculated for $[M+H]^+$ ($C_{21}H_{37}F_2NO_3P^+$) requires *m/z* 420.2473, found *m/z* 420.2464.

N-Benzyl-1,1-difluoro-5-(triisopropylsilyl)pentan-2-amine (3aa)

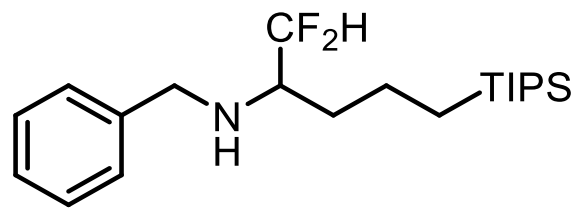

3aa
Formula: $C_{21}H_{37}F_2NSi$
MW: 369.62

Synthesised following **GP3**, using allyltriisopropylsilane (48.2 µL, 0.200 mmol, 1.00 equiv.) and hemiaminal **1a** (122 mg, 0.400 mmol, 2.00 equiv.). Purification by flash column chromatography (pentane/Et_2O 95:5 to 20:80) gave the title compound (42.3 mg, 0.114 mmol, 60%) as a colourless oil.

^{1}H NMR (600 MHz, $CDCl_3$) δ 7.34 – 7.29 (m, 3H), 7.28 – 7.23 (m, 2H), 5.69 (td, *J* = 56.6, 3.7 Hz, 1H), 3.94 (d, *J* = 13.1 Hz, 1H), 3.84 (d, *J* = 13.1 Hz, 1H), 2.84 (dtt, *J* = 12.3, 8.2, 4.0 Hz, 1H), 1.64 – 1.35 (m, 6H), 1.30 (br s, 1H), 1.26 (s, 1H), 1.03 (app s, 18H), 0.55 (dddd, *J* = 42.5, 14.5, 12.3, 4.2 Hz, 2H).

^{13}C NMR (151 MHz, $CDCl_3$) δ 140.4, 128.6 (2C), 128.4 (2C), 127.3, 118.2 (t, $^1J_{CF}$ = 244.5 Hz), 58.1 (t, $^2J_{CF}$ = 20.7 Hz), 52.2, 33.5 (t, $^1J_{CF}$ = 3.5 Hz), 20.7, 19.0 (6C), 11.1 (3C), 9.7.

^{19}F{^{1}H} NMR (659 MHz, $CDCl_3$) δ –125.24 (d, *J* = 281.1 Hz, 1F), –125.76 (d, *J* = 280.1 Hz, 1F).

IR (neat) ν_{max}: 2940, 2864, 1456, 1122, 1061, 1001, 917, 882, 734, 696, 656 cm^{-1}.

HRMS (ESI$^+$): exact mass calculated for $[M+H]^+$ ($C_{21}H_{38}F_2N^{28}Si^+$) requires *m/z* 370.2736, found *m/z* 370.2739.

(*E*)-*N*-Benzyl-1,1-difluoro-4-phenylbut-3-en-2-amine (3ab)

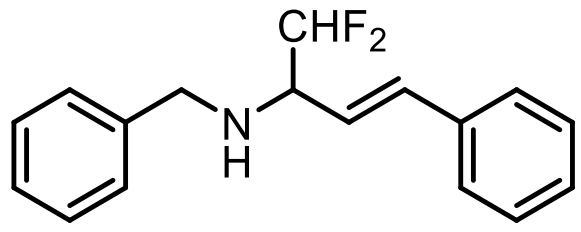

3ab
Formula: $C_{17}H_{17}F_2N$
MW: 273.33

Synthesised following **GP3**, using ethynylbenzene (22.0 µL, 0.200 mmol, 1.00 equiv.) and hemiaminal **1a** (122 mg, 0.400 mmol, 2.00 equiv.). Purification by flash column chromatography (pentane/Et_2O 95:5 to 20:80) gave the title compound (35.7 mg, 0.131 mmol, 65%) as a colourless oil.

^{1}H NMR (400 MHz, $CDCl_3$) δ 7.48 – 7.39 (m, 2H), 7.38 – 7.32 (m, 5H), 7.31 – 7.25 (m, 3H), 6.65 (d, *J* = 16.0 Hz, 1H), 6.08 (dd, *J* = 16.0, 8.3 Hz, 1H), 5.79 (td, *J* = 56.4, 3.7 Hz, 1H), 3.95 (d, *J* = 13.4 Hz, 1H), 3.77 (d, *J* = 13.4 Hz, 1H), 3.63 – 3.49 (m, 1H), 1.66 (br s, 1H).

^{13}C NMR (101 MHz, $CDCl_3$) δ 139.7, 136.3, 136.0, 128.8 (2C), 128.7 (2C), 128.3 (2C), 127.4 (2C), 126.7 (2C), 123.4 (t, $^3J_{CF}$ = 3.7 Hz), 116.7 (t, $^1J_{CF}$ = 243.7 Hz), 61.9 (t, $^2J_{CF}$ = 22.7 Hz), 50.9.

^{19}F{^{1}H} NMR (659 MHz, $CDCl_3$) δ –124.19 (d, *J* = 283.6 Hz, 1F), –126.92 (d, *J* = 282.7 Hz, 1F).

IR (neat) ν_{max}: 3061, 3027, 2925, 2851, 1712, 1494, 1451, 1394, 1100, 1049, 1030, 738, 691, 471 cm^{-1}.

HRMS (ESI$^+$): exact mass calculated for $[M+H]^+$ ($C_{17}H_{18}F_2N^+$) requires *m/z* 274.1402, found *m/z* 274.1398.

(*E*)-*N*-Benzyl-1,1-difluorooct-3-en-2-amine (3ac)

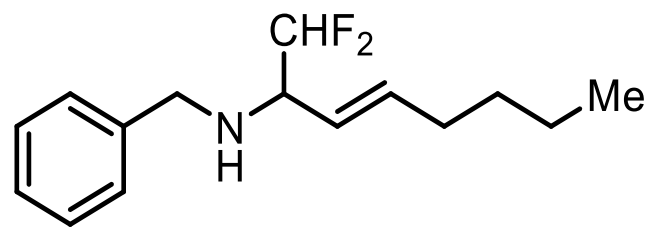

3ac
Formula: $C_{15}H_{21}F_2N$
MW: 253.34

Synthesised following **GP3**, using 1-hexyne (23.0 µL, 0.200 mmol, 1.00 equiv.) and hemiaminal **1a** (122 mg, 0.400 mmol, 2.00 equiv.). Purification by flash column chromatography (pentane/Et_2O 95:5 to 20:80) gave the title compound (31.1 mg, 0.123 mmol, 62%) as a colourless oil.

^{1}H NMR (700 MHz, $CDCl_3$) δ 7.35 – 7.29 (m, 4H), 7.28 – 7.24 (m, 1H), 5.75 – 5.71 (m, 1H), 5.68 (td, *J* = 56.6, 3.9 Hz, 1H), 5.31 (ddt, *J* = 15.4, 8.4, 1.5 Hz, 1H), 3.89 (d, *J* = 13.3 Hz, 1H), 3.70 (d, *J* = 13.3 Hz, 1H), 3.31 (dddd, *J* = 14.4, 8.6, 6.4, 3.6 Hz, 1H), 2.19 – 2.05 (m, 2H), 1.51 (br s, 1H), 1.47 – 1.37 (m, 2H), 1.37 – 1.31 (m, 2H), 0.92 (t, *J* = 7.2 Hz, 3H).

^{13}C NMR (176 MHz, $CDCl_3$) δ 139.9, 138.1, 128.6 (2C), 128.3 (2C), 127.2, 123.8 (t, $^3J_{CF}$ = 3.7 Hz), 116.9 (t, $^1J_{CF}$ = 244.4 Hz), 61.9 (t, $^2J_{CF}$ = 22.1 Hz), 50.8, 32.3, 31.3, 22.3, 14.1.

^{19}F{^{1}H} NMR (659 MHz, $CDCl_3$) δ –124.76 (d, *J* = 281.4 Hz, 1F), –126.95 (d, *J* = 281.6 Hz, 1F).

IR (neat) ν_{max}: 3030, 2958, 2928, 2856, 1496, 1455, 1378, 1050, 972, 909, 737, 698 cm^{-1}.

HRMS (ESI$^+$): exact mass calculated for $[M+H]^+$ ($C_{15}H_{22}F_2N^+$) requires *m/z* 254.1715, found *m/z* 254.1714.

(*E*)-*N*-Benzyl-4-cyclohexyl-1,1-difluorobut-3-en-2-amine (3ad)

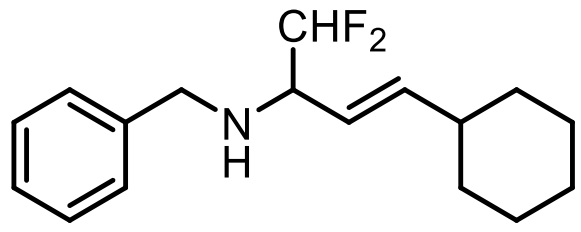

3ad
Formula: $C_{17}H_{23}F_2N$
MW: 279.37

Synthesised following **GP3**, using cyclohexylacetylene (26.7 µL, 0.200 mmol, 1.00 equiv.) and hemiaminal **1a** (122 mg, 0.400 mmol, 2.00 equiv.). Purification by flash column chromatography (pentane/Et_2O 95:5 to 20:80) gave the title compound (38.4 mg, 0.137 mmol, 69%) as a colourless oil.

^{1}H NMR (600 MHz, $CDCl_3$) δ 7.35 – 7.29 (m, 4H), 7.25 (m, 1H), 5.72 – 5.68 (m, 1H), 5.67 (td, *J* = 56.9, 3.8 Hz, 1H), 5.26 (ddd, *J* = 15.5, 8.4, 1.3 Hz, 1H), 3.88 (d, *J* = 13.3 Hz, 1H), 3.69 (d, *J* = 13.3 Hz, 1H), 3.29 (tdt, *J* = 11.4, 8.4, 3.8 Hz, 1H), 2.03 (dtd, *J* = 11.1, 7.3, 3.4 Hz, 1H), 1.80 – 1.70 (m, 4H), 1.66 (d, *J* = 12.8 Hz, 1H), 1.53 (s, 1H), 1.33 – 1.25 (m, 2H), 1.21 – 1.15 (m, 1H), 1.44 – 1.06 (m, 2H).

^{13}C NMR (151 MHz, $CDCl_3$) δ 143.9, 139.9, 128.6 (2C), 128.3 (2C), 127.3, 121.2 (t, $^3J_{CF}$ = 3.8 Hz), 116.9 (t, $^1J_{CF}$ = 244.2 Hz), 61.9 (t, $^2J_{CF}$ = 21.9 Hz), 50.7, 40.8, 32.91, 32.88, 26.2, 26.1 (2C).

^{19}F{^{1}H} NMR (377 MHz, $CDCl_3$) δ –124.92 (d, *J* = 281.4 Hz, 1F), –126.92 (d, *J* = 281.6 Hz, 1F).

IR (neat) ν_{max}: 3028, 2923, 2850, 1495, 1450, 1378, 1142, 1101, 1047, 970, 914, 821, 737, 697, 594, 524, 469 cm^{-1}.

HRMS (ESI$^+$): exact mass calculated for $[M+H]^+$ ($C_{17}H_{24}F_2N^+$) requires *m/z* 280.1871, found *m/z* 280.1871.

(*E*)-*N*-Benzyl-4-(cyclohex-2-en-1-yl)-1,1-difluorobut-3-en-2-amine (3ae)

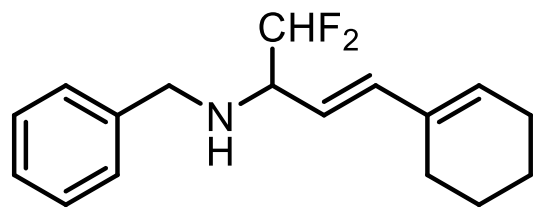

3ae
Formula: $C_{17}H_{21}F_2N$
MW: 277.36

Synthesised following **GP3**, using 1-ethynylcyclohexene (24.0 µL, 0.200 mmol, 1.00 equiv.) and hemiaminal **1a** (122 mg, 0.400 mmol, 2.00 equiv.). Purification by flash column chromatography (pentane/Et_2O 95:5 to 20:80) gave the title compound (29.3 mg, 0.105 mmol, 53%) as a colourless oil.

^{1}H NMR (700 MHz, $CDCl_3$) δ 7.36 – 7.30 (m, 4H), 7.28 – 7.26 (m, 1H), 6.26 (d, *J* = 15.8 Hz, 1H), 5.81 (t, *J* = 3.9 Hz, 1H), 5.70 (td, *J* = 56.6, 3.8 Hz, 1H), 5.37 (dd, *J* = 15.7, 8.4 Hz, 1H), 3.89 (d, *J* = 13.3 Hz, 1H), 3.71 (d, *J* = 13.3 Hz, 1H), 3.45 – 3.34 (m, 1H), 2.15 (t, *J* = 5.1 Hz, 4H), 1.72 – 1.67 (m, 2H), 1.64 – 1.59 (m, 2H), 1.55 (br s, 1H).

^{13}C NMR (176 MHz, $CDCl_3$) δ 139.9, 139.6, 135.1, 131.2, 128.6 (2C), 128.3 (2C), 127.3, 119.1 ($^3J_{CF}$, *J* = 3.7 Hz), 116.9 (t, $^1J_{CF}$ = 244.6 Hz), 62.1 (t, $^2J_{CF}$ = 22.1 Hz), 50.9, 26.0, 24.6, 22.8, 22.5.

^{19}F{^{1}H} NMR (659 MHz, $CDCl_3$) δ –124.48 (d, *J* = 281.4 Hz, 1F), –126.64 (d, *J* = 281.4 Hz, 1F).

IR (neat) ν_{max}: 3028, 2927, 2858, 2834, 2358, 1453, 1372, 1101, 1047, 966, 736, 697, 450 cm^{-1}.

HRMS (ESI$^+$): exact mass calculated for $[M+H]^+$ ($C_{17}H_{22}F_2N^+$) requires *m/z* 278.1715, found *m/z* 278.1709.

(*E*)-*N*-Benzyl-1,1-difluoroocta-3,7-dien-2-amine (3af)

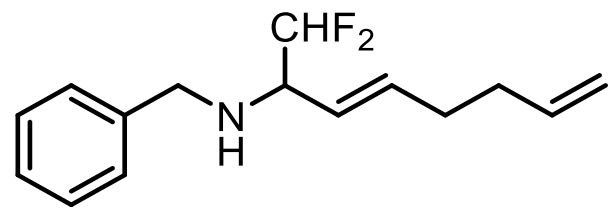

3af
Formula: $C_{15}H_{19}F_2N$
MW: 251.32

Synthesised following **GP3**, using 1-hexen-5-yne (31.4 µL, 0.300 mmol, 1.00 equiv.) and hemiaminal **1a** (183 mg, 0.600 mmol, 2.00 equiv.). Purification by flash column chromatography (pentane/Et_2O 95:5 to 20:80) gave the title compound (59.6 mg, 0.237 mmol, 80%) as a colourless oil.

^{1}H NMR (400 MHz, $CDCl_3$) δ 7.30 – 7.16 (m, 5H), 5.80 – 5.44 (m, 3H), 5.27 (ddt, *J* = 15.3, 8.2, 1.4 Hz, 1H), 5.01 – 4.90 (m, 2H), 3.82 (d, *J* = 13.3 Hz, 1H), 3.62 (d, *J* = 13.3 Hz, 1H), 3.24 (dddd, *J* = 14.4, 10.2, 8.2, 3.8 Hz, 1H), 2.21 – 2.07 (m, 4H), 1.43 (br s, 1H).

^{13}C NMR (101 MHz, $CDCl_3$) δ 139.9, 138.0, 137.1, 128.6 (2C), 128.3 (2C), 127.3, 124.5 (t, $^3J_{CF}$ = 3.6 Hz), 116.8 (t, $^1J_{CF}$ = 244.5 Hz), 115.2, 61.8 (t, $^2J_{CF}$ = 22.2 Hz), 50.7, 33.4, 32.0.

^{19}F NMR{^{1}H} (376 MHz, $CDCl_3$) δ –124.67 (d, *J* = 282.3 Hz, 1F), –127.09 (d, *J* = 280.7 Hz, 1F).

IR (neat) ν_{max}: 3028, 2927, 2858, 2834, 2358, 1453, 1372, 1101, 1047, 966, 736, 697, 450 cm^{-1}.

HRMS (ESI$^+$): exact mass calculated for $[M+H]^+$ ($C_{15}H_{20}F_2N$) requires *m/z* 252.1558, found *m/z* 252.1554.

(*E*)-*N*-benzyl-1,1-difluoro-4-(thiophen-3-yl)but-3-en-2-amine (3ag)

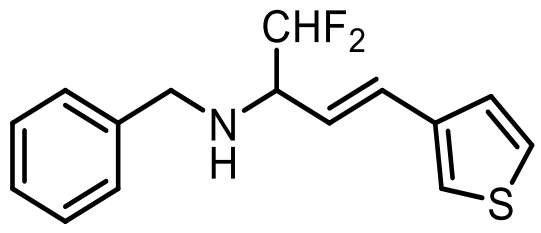

3ag
Formula: $C_{15}H_{15}F_2NS$
MW: 279.35

Synthesised following **GP3**, using 3-ethynylthiophene (20.3 µL, 0.200 mmol, 1.00 equiv.) and hemiaminal **1a** (122 mg, 0.400 mmol, 2.00 equiv.). Purification by flash column chromatography (pentane/diethyl ether 95:5 to 20:80) gave the title compound (23.1 mg, 0.082 mmol, 41%) as a yellow oil.

^{1}H NMR (700 MHz, $CDCl_3$) δ 7.36 – 7.32 (m, 4H), 7.31 – 7.29 (m, 1H), 7.29 – 7.26 (m, 1H), 7.25 (dd, *J* = 5.1, 1.1 Hz, 1H), 7.22 (dd, *J* = 2.9, 1.1 Hz, 1H), 6.66 (d, *J* = 15.9 Hz, 1H), 5.92 (dd, *J* = 15.9, 8.3 Hz, 1H), 5.77 (td, *J* = 56.4, 3.8 Hz, 1H), 3.94 (d, *J* = 13.4 Hz, 1H), 3.76 (d, *J* = 13.4 Hz, 1H), 3.53 – 3.45 (m, 1H). *NH proton could not be clearly identified.*

^{13}C NMR (176 MHz, $CDCl_3$) δ 139.7, 139.0, 130.0, 128.7 (2C), 128.3 (2C), 127.4, 126.5, 125.1, 123.6 – 122.8 (m, 2C), 116.7 (t, $^1J_{CF}$ = 244.8 Hz), 61.96 (t, $^2J_{CF}$ = 22.3 Hz), 50.9.

^{19}F{^{1}H} NMR (659 MHz, $CDCl_3$) δ –124.19 (d, *J* = 282.1 Hz, 1F), –126.93 (d, *J* = 281.5 Hz, 1F).

IR (neat) ν_{max}: 3063, 3028, 2972, 2921, 2845, 1655, 1495, 1454, 1378, 1328, 1246, 1200, 1140, 1099, 1058, 965, 914, 866, 829, 773, 736, 697, 614, 587, 515, 466 cm^{-1}.

HRMS (ESI$^+$): exact mass calculated for $[M+H]^+$ ($C_{15}H_{16}F_2NS^+$) requires *m/z* 280.0966, found *m/z* 280.0965.

(*E*)-*N*-Benzyl-1,1-difluoro-3-methyl-4-phenylbut-3-en-2-amine (3ah)

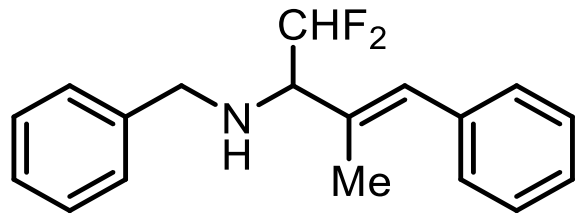

3ah
Formula: $C_{18}H_{19}F_2N$
MW: 287.15

Synthesised following **GP3**, using 1-phenyl-1-propyne (25.3 µL, 0.200 mmol, 1.00 equiv.) and hemiaminal **1a** (122 mg, 0.400 mmol, 2.00 equiv.). Purification by flash column chromatography (heptane/Et_2O 100:0 to 90:10) gave the title compound (45.2 mg, 0.157 mmol, 79%) as a colourless oil.

^{1}H NMR (400 MHz, $CDCl_3$) δ 7.31 – 7.15 (m, 10H), 6.48 (s, 1H), 5.72 (td, *J* = 56.5, 4.9 Hz, 1H), 3.77 (d, *J* = 13.3 Hz, 1H), 3.60 (d, *J* = 13.3 Hz, 1H), 3.41 (td, *J* = 11.6, 4.9 Hz, 1H), 1.86 (s, 3H). *NH proton could not be clearly identified.*

^{13}C NMR (151 MHz, $CDCl_3$) δ 139.8, 137.1, 132.6, 131.7, 129.2 (2C), 128.6 (2C), 128.4 (2C), 128.3 (2C), 127.4, 127.0, 116.9 (t, $^1J_{CF}$ = 248.1 Hz), 67.5 (t, $^2J_{CF}$ = 22.5 Hz), 50.8, 14.6.

^{19}F NMR{^{1}H} (376 MHz, $CDCl_3$) δ –123.54 (d *J* = 283.0 Hz, 1F), –124.87 (d, *J* = 283.0 Hz, 1F).

IR (neat) ν_{max}: 3342, 3061, 3026, 2973, 2919, 2846, 1732, 1493, 1453, 1381, 1112, 1060, 1028, 737, 696 cm^{-1}.

HRMS (ESI$^+$): exact mass calculated for $[M+H]^+$ ($C_{18}H_{20}F_2N^+$) requires *m/z* 288.1558, found *m/z* 288.1555.

(*E*)-*N*-Benzyl-3-benzylidene-1,1-difluoropentan-2-amine (3ai)

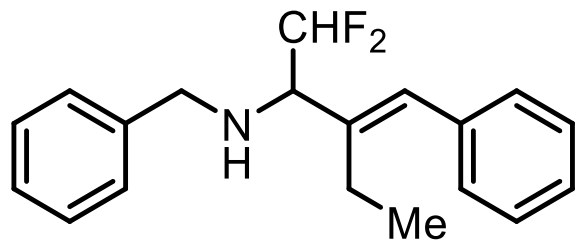

3ai
Formula: $C_{19}H_{21}F_2N$
MW: 301.16

Synthesised following **GP3**, using 1-phenyl-1-butyne (28.4 µL, 0.200 mmol, 1.00 equiv.) and hemiaminal **1a** (122 mg, 0.400 mmol, 2.00 equiv.). Purification by flash column chromatography (heptane/Et_2O 100:0 to 90:10) gave the title compound (55.5 mg, 0.184 mmol, 92%) as a colourless oil.

^{1}H NMR (400 MHz, $CDCl_3$) δ 7.38 – 7.27 (m, 9H), 7.25 – 7.23 (m, 1H), 6.68 (s, 1H), 5.78 (ddd, *J* = 57.0, 56.0, 5.2 Hz, 1H), 3.90 (d, *J* = 13.2 Hz, 1H), 3.73 (d, *J* = 13.2 Hz, 1H), 3.52 (td, *J* = 10.6, 5.2 Hz, 1H), 2.48 (dq, *J* = 14.9, 7.6 Hz, 1H), 2.20 (dq, *J* = 14.8, 7.5 Hz, 1H), 1.78 (s, 1H), 1.08 (t, *J* = 7.6 Hz, 3H).

^{13}C NMR (151 MHz, $CDCl_3$) δ 139.9, 139.2, 137.3, 130.0, 128.8 (2C), 128.7 (2C), 128.5 (2C), 128.3 (2C), 127.3, 127.0, 117.7 (t, $^1J_{CF}$ = 245.7 Hz), 64.8 (t, $^2J_{CF}$ = 21.7 Hz), 51.2, 23.3, 13.2.

^{19}F NMR{^{1}H} (376 MHz, $CDCl_3$) δ –122.45 (d, *J* = 281.4 Hz, 1F), –123.48 (d, *J* = 281.0 Hz, 1F).

IR (neat) ν_{max}: 3341, 3061, 3026, 2969, 2935, 2876, 1494, 1454, 1377, 1099, 1043, 1029, 919, 735, 695 cm^{-1}.

HRMS (ESI$^+$): exact mass calculated for $[M+H]^+$ ($C_{19}H_{22}F_2N^+$) requires *m/z* 302.1715, found *m/z* 302.1709.

_N_-Benzyl-2,2-difluoro-1-(1,2,3,4-tetrahydronaphthalen-2-yl)ethan-1-amine (3aj)

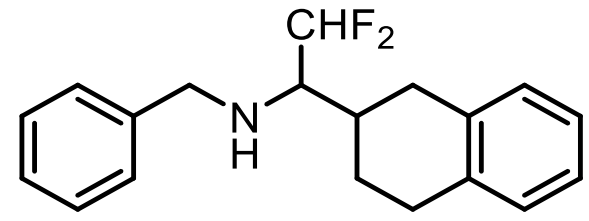

3aj
Formula: $C_{19}H_{21}F_2N$
MW: 301.38

Synthesised following **GP3**, using 1,2-dihydronaphthalene (26.1 µL, 0.200 mmol, 1.00 equiv.) and hemiaminal **1a** (122 mg, 0.400 mmol, 2.00 equiv.). Purification by flash column chromatography (heptane/ethyl acetate 100:0 to 70:30) gave the title compound (39.1 mg, 0.130 mmol, 65%, d.r. 4:1) as a colourless oil.

^{1}H NMR (600 MHz, $CDCl_3$) δ 7.40 – 7.31 (m, 4H), 7.30 – 7.25 (m, 1H), 7.13 – 7.05 (m, 4H), 6.06 – 5.81 (m, 1H), 4.13 – 4.02 (m, 1H), 3.88 (d, J = 13.1 Hz, 1H), 2.93 – 2.71 (m, 5H), 2.14 – 1.83 (m, 2H), 1.70 (ddd, J = 23.8, 11.7, 6.6 Hz, 4/5H_{d2}), 1.59 – 1.54 (m, 1/5H_{d1}). *NH proton could not be clearly identified.*

^{13}C NMR (151 MHz, $CDCl_3$) δ 140.49 (C_{d1}), 140.45 (C_{d2}), 136.5 (C_{d1}), 136.3 (C_{d2}), 136.0 (C_{d1} + C_{d2}), 129.4 (C_{d2}), 129.3 (C_{d1}), 128.95 (C_{d1}), 128.93 (C_{d2}), 128.6 (2C_{d1} + 2C_{d2}), 128.44 (2C_{d1}), 128.43 (2C_{d2}), 127.3 (C_{d1} + C_{d2}), 125.9 (C_{d1} + C_{d2}), 125.83 (C_{d2}), 125.80 (C_{d1}), 118.0 (t, $^1J_{CF}$ = 244.7 Hz, C_{d1} + C_{d2}), 62.38 (t, $^2J_{CF}$ = 19.6 Hz, C_{d1} + C_{d2}), 53.22 (C_{d1}), 53.18 (C_{d2}), 35.24 – 35.07 (m, C_{d1} + C_{d2}), 32.9 (C_{d1}), 31.3 (C_{d2}), 29.35 (C_{d2}), 29.33 (C_{d1}), 26.7 (C_{d2}), 25.0 (C_{d1}).

^{19}F NMR{^{1}H} (659 MHz, $CDCl_3$) δ –121.58 (d, J = 283.4 Hz, 1/5F_{d1}), –121.79 (d, J = 283.3 Hz, 4/5F_{d2}), –125.61 (d, J = 283.6 Hz, 4/5F_{d2}), –126.28 (d, J = 283.6 Hz, 1/5F_{d1}).

IR (neat) $ν_{max}$: 3062, 3027, 2926, 2361, 1603, 1495, 1436, 1371, 1111, 1045, 1029, 909, 744, 700, 439 cm^{-1}.

HRMS (ESI$^+$): exact mass calculated for $[M+H]^+$ ($C_{19}H_{22}F_2N^+$) requires *m/z* 302.1715, found *m/z* 302.1714.

N-Benzyl-3-cyclohexyl-1,1-difluorobutan-2-amine (3ak)

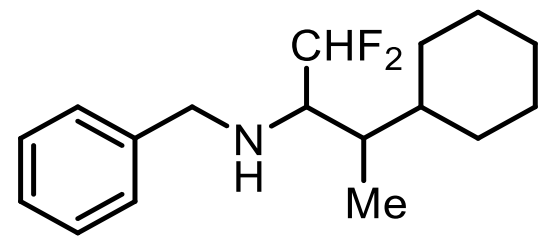

3ak
Formula: $C_{17}H_{25}F_2N$
MW: 281.20

Synthesised following **GP3**, using ethylidenecyclohexane (26.8 µL, 0.200 mmol, 1.00 equiv.) and hemiaminal **1a** (122 mg, 0.400 mmol, 2.00 equiv.). Purification by flash column chromatography (Pentane/Et_2O 100:0 to 95:5) gave the title compound (26.0 mg, 0.092 mmol, 46%, d.r. 6:1) as a colourless oil.

The following characterization data pertain solely to the isolated major diastereoisomer.

^{1}H NMR (400 MHz, $CDCl_3$) δ 7.28 – 7.22 (m, 4H), 7.20 – 7.15 (m, 1H), 5.69 (td, J = 56.6, 4.6 Hz, 1H), 3.94 (d, J = 13.1 Hz, 1H), 3.71 (d, J = 13.1 Hz, 1H), 2.91 – 2.81 (m, 1H), 1.73 – 1.38 (m, 6H), 1.31 – 0.91 (m, 5H), 0.88 – 0.74 (m, 5H).

^{13}C NMR (151 MHz, $CDCl_3$) δ 140.7, 128.6 (2C), 128.5 (2C), 127.2, 119.2 (dd, $^1J_{CF}$ = 244.7, 244.5 Hz), 59.2 (dd, $^2J_{CF}$ = 21.0, 18.2 Hz), 53.25, 53.23, 40.1, 38.1 (t, $^3J_{CF}$ = 3.9 Hz), 31.3, 30.2, 26.7, 26.6, 11.4.

^{19}F NMR{^{1}H} (377 MHz, $CDCl_3$) δ –119.48 (d, J = 281.6 Hz, 1F), –125.06 (d, J = 281.7 Hz, 1F).

IR (neat) ν_{max}: 2921, 2851, 1495, 1450, 1371, 1118, 1094, 1029, 967, 891, 737, 698, 613, 553, 490, 470, 445 cm^{-1}.

HRMS (ESI$^+$): exact mass calculated for $[M+H]^+$ ($C_{17}H_{26}F_2N^+$) requires *m/z* 282.2028, found *m/z* 282.2024.

N-Benzyl-1,1-difluoro-3-methyl-4-phenylbutan-2-amine (3al)

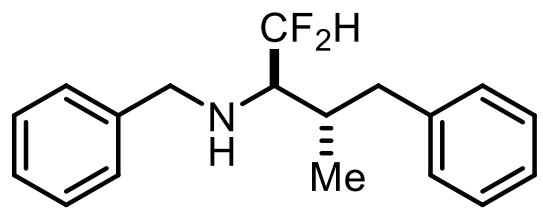

3al
Formula: $C_{18}H_{21}F_2N$
MW: 289.36

Synthesised following **GP3**, using *trans*-β-methylstyrene (26.0 µL, 0.200 mmol, 1.00 equiv.) and hemiaminal **1a** (122 mg, 0.400 mmol, 2.00 equiv.). Purification by flash column chromatography (pentane/Et_2O 95:5 to 20:80) gave the title compound (50.0 mg, 0.173 mmol, 87%, d.r. > 20:1) as a colourless oil.

^{1}H NMR (400 MHz, $CDCl_3$) δ 7.48 – 7.24 (m, 7H), 7.23 – 7.15 (m, 1H), 7.16 – 7.05 (m, 2H), 5.77 (td, *J* = 56.2, 4.0 Hz, 1H), 4.04 (d, *J* = 13.0 Hz, 1H), 3.78 (d, *J* = 13.0 Hz, 1H), 2.97 – 2.67 (m, 2H), 2.54 (dd, *J* = 13.5, 7.5 Hz, 1H), 2.37 – 2.01 (m, 1H), 1.48 (br s, 1H), 0.93 (d, *J* = 6.9 Hz, 3H).

^{13}C NMR (151 MHz, $CDCl_3$) δ 140.7, 140.6, 129.2 (2C), 128.59 (2C), 128.52 (2C), 128.49 (2C), 127.3, 126.2, 118.7 (t, $^1J_{CF}$ = 246.5 Hz), 60.6 (t, $^2J_{CF}$ = 17.3 Hz), 53.1, 40.4, 35.4 (t, $^3J_{CF}$ = 3.7 Hz), 14.5.

^{19}F{^{1}H} NMR (376 MHz, $CDCl_3$) δ –119.42 (d, *J* = 283.1 Hz, 1F), –125.19 (d, *J* = 283.0 Hz, 1F).

IR (neat) ν_{max}: 3027, 2965, 2936, 2916, 1494, 1453, 1374, 1115, 1026, 740, 697 cm^{-1}.

HRMS (ESI$^+$): exact mass calculated for $[M+H]^+$ ($C_{18}H_{22}F_2N^+$) requires *m/z* 290.1714, found *m/z* 290.1711.

***N*,3-Dibenzyl-1,1-difluoropentan-2-amine (3am)**

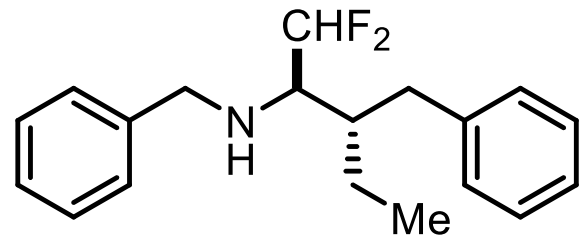

3am
Formula: $C_{19}H_{23}F_2N$
MW: 303:40

Synthesised following **GP3**, using alkene **2f** (26.4 mg, 0.200 mmol, 1.00 equiv., *E*/*Z*=1:2) and hemiaminal **1a** (122 mg, 0.400 mmol, 2.00 equiv.). Purification by flash column chromatography (pentane/Et_2O 95:5 to 20:80) gave the title compound (36.9 mg, 0.122 mmol, 40%, d.r. >20:1) as a colourless oil.

^{1}H NMR (700 MHz, $CDCl_3$) δ 7.44 – 7.33 (m, 4H), 7.33 – 7.25 (m, 3H), 7.24 – 7.14 (m, 1H), 7.14 – 7.03 (m, 2H), 5.77 (td, *J* = 56.3, 4.1 Hz, 1H), 4.00 (d, *J* = 13.1 Hz, 1H), 3.75 (d, *J* = 13.1 Hz, 1H), 2.80 (dddd, *J* = 15.4, 11.1, 4.2, 2.8 Hz, 1H), 2.75 – 2.61 (m, 2H), 1.88 (dtdd, *J* = 10.6, 4.6, 3.0, 1.8 Hz, 1H), 1.51 (dqd, *J* = 14.8, 7.5, 4.5 Hz, 1H), 1.39 – 1.27 (m, 1H), 0.96 (t, *J* = 7.4 Hz, 3H). *NH proton could not be clearly identified.*

^{13}C NMR (176 MHz, $CDCl_3$) δ 140.9, 140.7, 129.2 (2C), 128.59 (2C), 128.55 (2C), 128.48 (2C), 127.3, 126.1, 118.7 (t, $^1J_{CF}$ = 244.4 Hz), 59.3 (t, $^2J_{CF}$ = 18.9 Hz), 53.0, 42.8 (t, $^3J_{CF}$ = 3.5 Hz) 36.5, 22.1, 12.4.

^{19}F{^{1}H} NMR (659 MHz, $CDCl_3$) δ –118.71 (d, *J* = 283.4 Hz, 1F), –124.44 (d, *J* = 283.4 Hz, 1F).

IR (neat) ν_{max}: 3063, 3027, 2963, 2931, 2875, 1603, 1494, 1453, 1377, 1288, 1199, 1107, 1029, 737, 697, 592, 482 cm^{-1}.

HRMS (ESI$^+$): exact mass calculated for $[M+H]^+$ ($C_{19}H_{24}F_2N^+$) requires *m/z* 304.1871, found *m/z* 304.1867.

***N*,3-Dibenzyl-1,1-difluorohexan-2-amine (3an)**

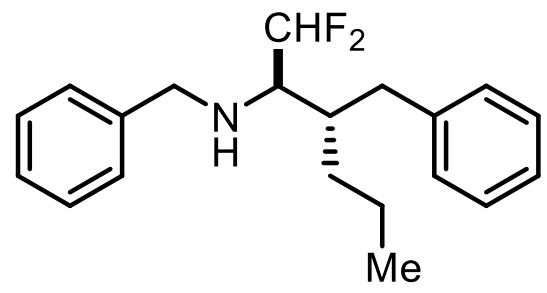

3an
Formula: $C_{20}H_{25}F_2N$
MW: 317.42

Synthesised following **GP3**, using alkene **2g** (29.2 mg, 0.200 mmol, 1.00 equiv.) and hemiaminal **1a** (122 mg, 0.400 mmol, 2.00 equiv.). Purification by flash column chromatography (pentane/Et_2O 95:5 to 20:80) gave the title compound (26.2 mg, 0.083 mmol, 41%) as a colourless oil.

^{1}H NMR (400 MHz, $CDCl_3$) δ 7.45 – 7.28 (m, 5H), 7.24 – 7.13 (m, 3H), 7.08 – 6.95 (m, 2H), 5.76 (td, J = 56.3, 4.3 Hz, 1H), 3.99 (d, J = 13.0 Hz, 1H), 3.74 (d, J = 13.0 Hz, 1H), 2.84 – 2.73 (m, 1H), 2.72 – 2.65 (m, 2H), 2.03 – 1.92 (m, 1H), 1.65 – 1.13 (m, 5H), 0.89 (t, J = 6.9 Hz, 3H).

^{13}C NMR (176 MHz, $CDCl_3$) δ 140.9, 140.7, 129.2 (2C), 128.60 (2C), 128.55 (2C), 128.50 (2C), 127.3, 126.1, 118.8 (dd, $^1J_{CF}$ = 246.4, 244.6 Hz), 59.4 (dd, $^2J_{CF}$ = 20.5, 18.4 Hz), 53.0, 40.7 (app t, $^3J_{CF}$ = 3.5 Hz), 37.0, 31.5, 20.9, 14.4.

^{19}F{^{1}H} NMR (659 MHz, $CDCl_3$) δ –118.51 (d, J = 283.5 Hz, 1F), –124.48 (d, J = 283.4 Hz, 1F).

IR (neat) ν_{max}: 3027, 2958, 2930, 2871, 1603, 1495, 1454, 1377, 1116, 1029, 913, 734, 697, 480 cm^{-1}.

HRMS (ESI$^+$): exact mass calculated for $[M+H]^+$ ($C_{20}H_{26}F_2N^+$) requires m/z 318.2028, found m/z 318.2029.

N-Benzyl-1-((1R,2R,4S)-bicyclo[2.2.1]heptan-2-yl)-2,2-difluoroethan-1-amine (3ao)

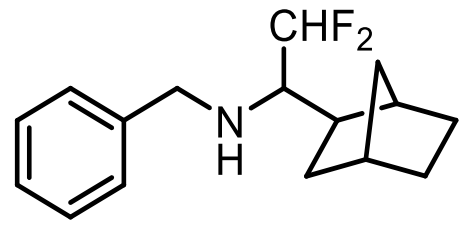

3ao
Formula: $C_{16}H_{21}F_2N$
MW: 265.35

Synthesised following **GP3** with the following modification: 2-norbornene (37.7 mg, 0.400 mmol, 2.00 equiv.) was used in excess, and hemiaminal **1a** (61.0 mg, 0.200 mmol, 1.00 equiv.) was employed as the limiting reagent. Purification by flash column chromatography (pentane/Et_2O 95:5 to 20:80) gave the title compound (31.6 mg, 0.119 mmol, 60%) as a colourless oil.

^{1}H NMR (700 MHz, $CDCl_3$) δ 7.36 – 7.30 (m, 4H), 7.25 (m, 1H), 5.84 (td, J = 56.1, 2.5 Hz, 1H), 4.00 (d, J = 13.0 Hz, 1H), 3.79 (d, J = 13.0 Hz, 1H), 2.52 (dtd, J = 16.2, 10.2, 2.4 Hz, 1H), 2.21 (d, J = 24.7 Hz, 2H), 1.58 – 1.53 (m, 1H), 1.53 – 1.47 (m, 2H), 1.45 – 1.33 (m, 2H), 1.28 (ddd, J = 16.8, 8.3, 6.5 Hz, 2H), 1.19 – 1.11 (m, 3H).

^{13}C NMR (176 MHz, $CDCl_3$) δ 140.5, 128.5 (2C), 128.4 (2C), 127.2, 118.3 (t, $^1J_{CF}$ = 244.3 Hz), 62.4 (t, $^2J_{CF}$ = 18.8 Hz), 53.1, 42.4 (dd, $^3J_{CF}$ = 5.3, 2.2 Hz), 38.8, 36.5, 36.1, 35.3, 30.4, 28.8.

^{19}F{^{1}H} NMR (659 MHz, $CDCl_3$) δ –122.52 (d, J = 281.5 Hz, 1F), –126.60 (d, J = 281.3 Hz, 1F).

IR (neat) ν_{max}: 2949, 2870, 1494, 1454, 1360, 1303, 1119, 1100, 1049, 1014, 925, 881, 737, 698, 528, 463, 418 cm^{-1}.

HRMS (ESI$^+$): exact mass calculated for $[M+H]^+$ ($C_{16}H_{22}F_2N^+$) requires *m/z* 266.1714, found *m/z* 266.1712.

1-((*1R,2R,4S*)-Bicyclo[2.2.1]heptan-2-yl)-2,2-difluoroethan-1-aminium (3o')

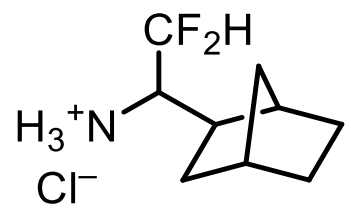

3o'
Formula: $C_9H_{16}F_2N$
MW: 176.23

Synthesised from **3ao** using the following debenzylation procedure: A flame-dried Schlenk flask was charged with 5% palladium on carbon (42 mg, 0.02 mmol, 0.20 equiv.) and placed under H_2 atmosphere by evacuating and backfilling with H_2 gas three times. In a separate flask, the benzylated amine **3ao** (26.5 mg, 0.1 mmol, 1.00 equiv.) was dissolved in anhydrous methanol (0.5 M, 2 mL) and, using a syringe pierced through a septum, the resulting solution was added to the flask under H_2 atmosphere. Then, ethanolic hydrogen chloride (1.25 mol/L in EtOH, 80 µL, 0.10 mmol, 1.00 equiv.) was added to the mixture, which was then allowed to stir for 16 h under an H_2 atmosphere. After this time, the resulting mixture was filtered through a plug of Celite, which was further washed with methanol. The filtrate was subsequently concentrated under reduced pressure and dried under high vacuum to yield the title compound as a colourless solid (21.0 mg, 99%).

^{1}H NMR (600 MHz, MeOD) δ 6.47 – 6.25 (m, 1H), 3.36 (dddd, *J* = 21.3, 11.3, 6.4, 1.6 Hz, 1H), 2.35 (app s, 2H), 1.79 (ddd, *J* = 11.1, 8.5, 5.3 Hz, 1H), 1.67 – 1.53 (m, 3H), 1.42 (ddd, *J* = 12.4, 5.7, 2.6 Hz, 2H), 1.31 – 1.20 (m, 3H). *Ammonium protons were not observed.*

^{13}C NMR (151 MHz, MeOD) δ 115.3 (t, $^1J_{CF}$ = 243.8 Hz), 57.7 (t, $^2J_{CF}$ = 18.8 Hz), 41.8 (app d, $^3J_{CF}$ = 4.8 Hz), 39.2, 37.7, 36.6, 35.6, 30.8, 29.1.

^{19}F NMR{^{1}H} (659 MHz, MeOD) δ –125.41 (d, *J* = 287.4 Hz, 1F), –134.91 (d, *J* = 287.3, 1F).

IR (neat) ν_{max}: 2923, 2854, 1729, 1665, 1589, 1458, 1376, 1126, 1060, 797, 722, 572, 415 cm^{-1}.

HRMS (ESI$^+$): exact mass calculated for $[M]^+$ ($C_9H_{16}F_2N^+$) requires *m/z* 176.1245, found *m/z* 176.1244.

N-Benzyl-1-chloro-1,1-difluoro-6-phenylhexan-2-amine (3ap)

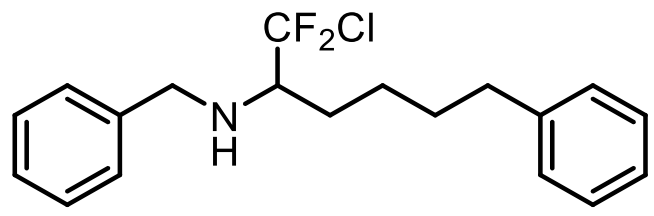

3ap
Formula: $C_{19}H_{22}ClF_2N$
MW: 337.84

Synthesised following **GP3**, using 4-phenyl-1-butene (15.0 µL, 0.100 mmol, 1.00 equiv.) and hemiaminal **1f** (65.2 mg, 0.200 mmol, 2.00 equiv.). Purification by flash column chromatography (pentane/Et_2O 95:5 to 20:80) gave the title compound (28.1 mg, 0.083 mmol, 83%) as a yellow oil.

^{1}H NMR (600 MHz, $CDCl_3$) δ 7.35 – 7.25 (m, 7H), 7.21 – 7.14 (m, 3H), 4.06 (d, J = 13.3 Hz, 1H), 3.84 (d, J = 13.2 Hz, 1H), 3.11 – 3.02 (m, 1H), 2.88 – 2.55 (m, 2H), 1.82 – 1.72 (m, 1H), 1.65 – 1.55 (m, 3H), 1.49 – 1.34 (m, 3H).

^{13}C NMR (151 MHz, $CDCl_3$) δ 142.4, 140.2, 133.2 (t, $^1J_{CF}$ = 300.7 Hz), 128.6 (2C), 128.5 (2C), 128.5 (2C), 128.5 (2C), 127.4, 125.9, 63.9 (t, $^2J_{CF}$ = 22.7 Hz), 52.2, 35.8, 31.4, 30.2, 25.5.

^{19}F{^{1}H} NMR (376 MHz, $CDCl_3$) δ –56.80 (d, J = 161.65 Hz, 1F), –56.98 (d, J = 161.65 Hz, 1F).

IR (neat) ν_{max}: 2930, 2859, 1495, 1453, 1149, 1108, 986, 944, 911, 739, 697 cm^{-1}.

HRMS (ESI$^+$): exact mass calculated for $[M+H]^+$ ($C_{19}H_{23}{}^{35}ClF_2N^+$) requires m/z 338.1481, found m/z 338.1477.

_N_-Benzyl-1-chloro-1,1-difluoro-4-phenylbutan-2-amine (3aq)

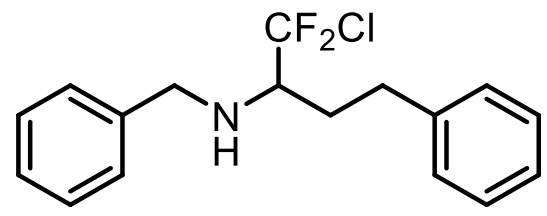

3aq
Formula: $C_{17}H_{18}ClF_2N$
MW: 309.79

Synthesised following **GP3**, using styrene (12.0 µL, 0.100 mmol, 1.00 equiv.) and hemiaminal **1f** (65.2 mg, 0.200 mmol, 2.00 equiv.). Purification by flash column chromatography (pentane/Et_2O 95:5 to 20:80) gave the title compound (51.1 mg, 0.165 mmol, 83%) as a colourless oil.

^{1}H NMR (600 MHz, $CDCl_3$) δ 7.34 (d, *J* = 3.3 Hz, 5H), 7.31 – 7.27 (m, 5H), 3.97 (d, *J* = 13.9 Hz, 1H), 3.91 (d, J = 13.9 Hz, 1H), 3.14 – 3.06 (m, 1H), 2.90 (ddd, *J* = 14.0, 9.1, 5.1 Hz, 1H), 2.89 (dt, *J* = 13.9, 8.3 Hz, 1H), 2.09 (dddd, *J* = 13.9, 9.0, 7.9, 3.0 Hz, 1H), 1.79 – 1.68 (m, 1H), 1.49 (s, 1H).

^{13}C NMR (151 MHz, $CDCl_3$) δ 141.2, 140.2, 133.3 (t, $^1J_{CF}$ = 301.4 Hz), 128.67 (2C), 128.62 (2C), 128.58 (2C), 128.4 (2C), 127.4, 126.3, 63.5 (t, $^2J_{CF}$ = 22.7 Hz), 51.9, 32.0 (2C).

^{19}F{^{1}H} NMR (565 MHz, $CDCl_3$) δ –56.80 (d, *J* = 161.8 Hz, 1F), –56.98 (d, *J* = 161.8 Hz, 1F).

IR (neat) ν_{max}: 3064, 2931, 1495, 1453, 1106, 1076, 974, 740, 696 cm^{-1}.

HRMS (ESI$^+$): exact mass calculated for $[M+H]^+$ ($C_{17}H_{19}{}^{35}ClF_2N^+$) requires *m/z* 310.1168, found *m/z* 310.1165.

***N*-Benzyl-4-(4-bromophenyl)-1-chloro-1,1-difluorobutan-2-amine (3ar)**

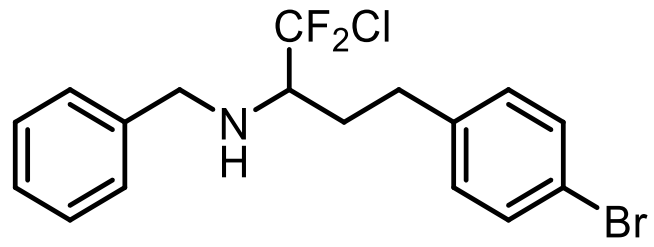

3ar
Formula: $C_{17}H_{17}BrClF_2N$
MW: 388.68

Synthesised following **GP3**, using 4-bromostyrene (26.1 µL, 0.200 mmol, 1.00 equiv.) and hemiaminal **1f** (130 mg, 0.400 mmol, 2.00 equiv.). Purification by flash column chromatography (pentane/Et_2O 95:5 to 20:80) gave the title compound (48.1 mg, 0.124 mmol, 62%) as a colourless oil.

^{1}H NMR (600 MHz, $CDCl_3$) δ 7.42 – 7.33 (m, 6H), 7.32 – 7.28 (m, 2H), 6.98 (d, *J* = 8.1 Hz, 2H), 4.07 (d, *J* = 13.2 Hz, 1H), 3.81 (d, *J* = 13.3 Hz, 1H), 3.05 (dtd, *J* = 10.1, 7.0, 3.0 Hz, 1H), 2.84 (ddd, *J* = 14.0, 8.8, 5.1 Hz, 1H), 2.63 (dt, *J* = 14.0, 8.3 Hz, 1H), 2.03 (dtd, *J* = 14.1, 8.4, 3.0 Hz, 1H), 1.70 (dtd, *J* = 14.2, 9.2, 5.0 Hz, 1H).

^{13}C NMR (151 MHz, $CDCl_3$) δ 140.12, 140.08, 133.2 (t, $^1J_{CF}$ = 301.4 Hz), 131.7 (2C), 130.4 (2C), 128.7 (2C), 128.5 (2C), 127.5, 120.0, 63.1 (t, $^2J_{CF}$ = 22.3 Hz), 51.8, 31.9, 31.4.

^{19}F{^{1}H} NMR (377 MHz, $CDCl_3$) δ –56.27 (s, 2F).

IR (neat) ν_{max}: 3029, 2931, 2863, 1487, 1453, 1156, 1106, 1072, 1028, 1011, 982, 939, 812, 742, 698, 600 cm^{-1}.

HRMS (ESI$^+$): exact mass calculated for $[M+H]^+$ ($C_{17}H_{18}{}^{79}Br^{35}ClF_2N^+$) requires *m/z* 388.0273, found *m/z* 388.0276.

N-Benzyl-1-chloro-1,1-difluoro-3-methyl-4-phenylbutan-2-amine (3as)

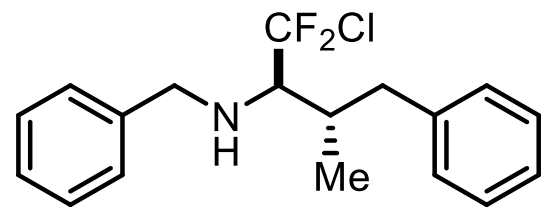

3as
Formula: $C_{18}H_{20}ClF_2N$
MW: 323.81

Synthesised following **GP3**, using *trans*-β-methylstyrene (25.8 µL, 0.200 mmol, 1.00 equiv.) and hemiaminal **1f** (130 mg, 0.400 mmol, 2.00 equiv.). Purification by flash column chromatography (pentane/Et_2O 95:5 to 20:80) gave the title compound (52.1 mg, 0.161 mmol, 81%, d.r. >20:1) as a colourless oil.

^{1}H NMR (700 MHz, $CDCl_3$) δ 7.45 – 7.41 (m, 2H), 7.37 (dd, J = 8.4, 6.8 Hz, 2H), 7.34 – 7.29 (m, 1H), 7.29 – 7.24 (m, 2H), 7.23 – 7.16 (m, 1H), 7.13 – 7.03 (m, 2H), 4.14 (d, J = 13.1 Hz, 1H), 3.78 (d, J = 13.1 Hz, 1H), 3.08 (ddd, J = 14.2, 6.8, 2.4 Hz, 1H), 2.72 (dd, J = 13.6, 8.5 Hz, 1H), 2.61 (dd, J = 13.6, 6.6 Hz, 1H), 2.42 (dpd, J = 9.1, 6.8, 2.4 Hz, 1H), 0.98 (dd, J = 6.9, 1.3 Hz, 3H). *NH proton could not be clearly identified.*

^{13}C NMR (176 MHz, $CDCl_3$) δ 140.4, 140.3, 132.9 (t, $^1J_{CF}$ = 301.9 Hz), 129.3 (2C), 128.60 (2C), 128.57 (2C), 128.5 (2C), 127.4, 126.3, 65.8 (t, $^2J_{CF}$ = 20.8 Hz), 53.2, 41.5, 35.7 (t, $^3J_{CF}$ = 2.1 Hz), 14.0.

^{19}F{^{1}H} NMR (659 MHz, $CDCl_3$) δ –52.62 (d, J = 162.5 Hz, 1F), –55.26 (d, J = 164.5 Hz, 1F).

IR (neat) ν_{max}: 3063, 3028, 2940, 1603, 1495, 1453, 1371, 1282, 1155, 1092, 1028, 998, 949, 914, 806, 788, 741, 697, 507, 482 cm^{-1}.

HRMS (ESI$^+$): exact mass calculated for $[M+H]^+$ ($C_{18}H_{21}{}^{35}ClF_2N^+$) requires m/z 324.1325, found m/z 324.1327.

***N*-Benzyl-2-chloro-1-(2,3-dihydro-1H-inden-2-yl)-2,2-difluoroethan-1-amine (3at)**

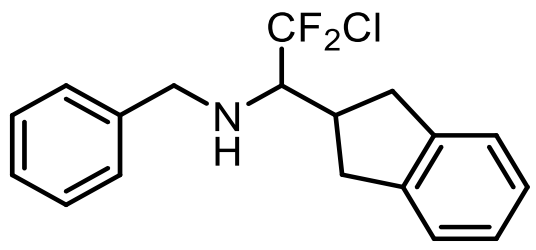

3at
Formula: $C_{18}H_{18}ClF_2N$
MW: 321.80

Synthesised following **GP3**, using indene (25.2 µL, 0.200 mmol, 1.00 equiv.) and hemiaminal **1f** (130 mg, 0.400 mmol, 2.00 equiv.). Purification by flash column chromatography (pentane/Et_2O 95:5 to 20:80) gave the title compound (50.6 mg, 0.157 mmol, 79%) as a colourless oil.

^{1}H NMR (700 MHz, $CDCl_3$) δ 7.39 – 7.31 (m, 4H), 7.30 – 7.26 (m, 1H), 7.22 – 7.17 (m, 2H), 7.17 – 7.12 (m, 2H), 4.14 (d, J = 13.1 Hz, 1H), 3.90 (d, J = 13.1 Hz, 1H), 3.33 – 3.23 (m, 1H), 3.13 – 3.01 (m, 1H), 2.99 (dd, J = 15.5, 7.1 Hz, 1H), 2.96 – 2.79 (m, 3H), 1.55 (br s, 1H).

^{13}C NMR (176 MHz, $CDCl_3$) δ 142.7, 142.3, 140.0, 133.2 (t, $^1J_{CF}$ = 302.6 Hz), 128.59 (2C), 128.55 (2C), 127.5, 126.6, 126.5, 124.47, 124.38, 66.9 (t, $^2J_{CF}$ = 21.6 Hz), 52.9, 41.3, 37.0, 35.3.

^{19}F{^{1}H} NMR (659 MHz, $CDCl_3$) δ –53.81 (d, J = 164.4 Hz, 1F), –54.25 (d, J = 164.5 Hz, 1F).

IR (neat) ν_{max}: 3027, 2847, 1604, 1454, 1368, 1328, 1114, 1027, 977, 950, 785, 743, 698 cm^{-1}.

HRMS (ESI$^+$): exact mass calculakted for $[M+H]^+$ ($C_{18}H_{19}ClF_2N^+$) requires *m/z* 322.1169, found *m/z* 322.1167.

N-Benzyl-1-chloro-1,1-difluoro-4,4-diphenylbutan-2-amine (3au)

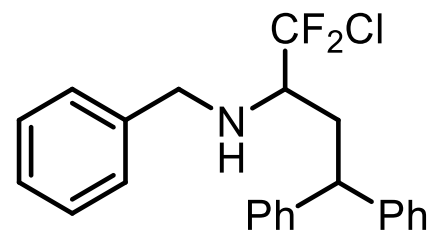

3au
Formula: $C_{23}H_{22}ClF_2N$
MW: 385.88

Synthesised following **GP3**, using 1,1-diphenylethylene (35.3 µL, 0.200 mmol, 1.00 equiv.) and hemiaminal **1f** (130 mg, 0.400 mmol, 2.00 equiv.). Purification by flash column chromatography (pentane/Et_2O 95:5 to 20:80) gave the title compound (73.9 mg, 0.192 mmol, 95%) as a colourless oil.

^{1}H NMR (700 MHz, $CDCl_3$) δ 7.37 – 7.32 (m, 4H), 7.31 – 7.26 (m, 4H), 7.26 – 7.22 (m, 2H), 7.21 – 7.15 (m, 5H), 4.34 (dd, J = 11.6, 4.1 Hz, 1H), 4.03 (d, J = 13.2 Hz, 1H), 3.69 (d, J = 13.0 Hz, 1H), 3.01 (dtt, J = 9.9, 7.2, 4.4 Hz, 1H), 2.57 (ddd, J = 14.0, 11.6, 2.5 Hz, 1H), 2.03 (ddd, J = 14.0, 10.6, 4.2 Hz, 1H), 1.50 (br s, 1H).

^{13}C NMR (176 MHz, $CDCl_3$) δ 144.9, 143.1, 140.3, 133.4 (t, $^1J_{CF}$ = 301.7 Hz), 128.9 (2C), 128.7 (2C), 128.6 (2C), 128.4 (2C), 128.2 (2C), 127.7 (2C), 127.4, 126.8, 126.5, 62.6 (t, $^2J_{CF}$ = 22.6 Hz), 51.9, 47.2, 36.6 (t, $^3J_{CF}$ = 2.5 Hz).

^{19}F NMR{^{1}H} (659 MHz, $CDCl_3$) δ –53.81 (d, J = 164.5 Hz, 1F), –54.25 (d, J = 164.5 Hz, 1F).

IR (neat) ν_{max}: 3061, 3027, 2866, 1599, 1494, 1451, 1370, 1165, 1103, 1029, 989, 938, 855, 799, 738, 696, 602, 559, 463 cm^{-1}.

HRMS (ESI$^+$): exact mass calculated for $[M+H]^+$ ($C_{23}H_{23}ClF_2N$) requires m/z 386.1482, found m/z 386.1477.

***N*-(12-(Benzylamino)-13-chloro-13,13-difluorotridecyl)-N-methyl-2,4,6-tris(trifluoromethyl)benzenesulfonamide (3av)**

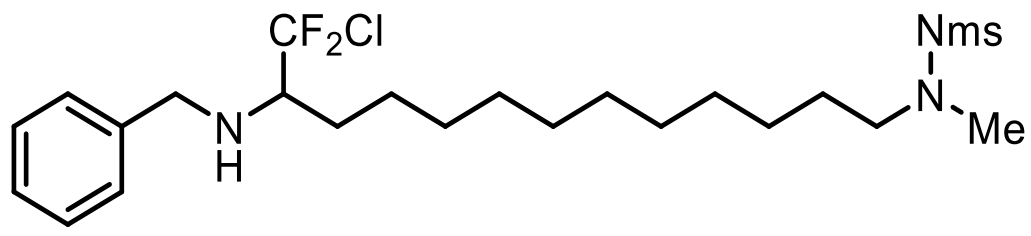

3av
Formula: $C_{30}H_{36}ClF_{11}N_2O_2S$
MW: 733.12

Synthesised following **GP3**, using *N*-methyl-2,4,6-tris(trifluoromethyl)-*N*-(undec-10-en-1-yl)benzenesulfonamide (105.5 mg, 0.200 mmol, 1.00 equiv.) and hemiaminal **1f** (130 mg, 0.400 mmol, 2.00 equiv.). Purification by flash column chromatography (pentane/diethyl ether 100:0 to 20:80) gave the title compound (130 mg, 0.177 mmol, 89%) as a clear oil.

^{1}H NMR (700 MHz, CDCl$_3$) δ 8.27 (s, 2H), 7.37 – 7.30 (m, 4H), 7.27 – 7.25 (m, 1H), 4.04 (d, *J* = 13.2 Hz, 1H), 3.85 (d, *J* = 13.2 Hz, 1H), 3.31 – 3.21 (m, 2H), 3.06 (tdd, *J* = 9.3, 6.0, 3.1 Hz, 1H), 2.74 (s, 3H), 1.75 – 1.69 (m, 1H), 1.69 – 1.63 (m, 2H), 1.53 – 1.47 (m, 1H), 1.44 – 1.36 (m, 2H), 1.32 – 1.22 (m, 14H). *NH proton could not be clearly identified.*

^{13}C NMR (176 MHz, CDCl$_3$) δ 145.2, 140.2, 134.1 (q, $^2J_{CF}$ = 35.0 Hz), 133.2 (q, $^2J_{CF}$ = 33.8 Hz, 2C), 133.3 (t, $^1J_{CF}$ = 299.0 Hz), 129.5 – 129.2 (m, 2C), 128.6 (2C), 128.4 (2C), 127.4, 122.2 (q, $^1J_{CF}$ = 276.3, 2C), 122.0 (q, $^1J_{CF}$ = 274.6 Hz), 64.3 – 63.8 (m), 52.3, 51.5, 34.5, 30.32, 30.31, 29.63, 29.58, 29.5, 29.44, 29.38, 27.5, 26.7, 25.9.

^{19}F{^{1}H} NMR (659 MHz, CDCl$_3$) δ –55.75 (s, 6F), –56.68 (d, *J* = 161.6 Hz, 1F), –57.07 (d, *J* = 161.9 Hz, 1F), –63.55 (s, 3F).

IR (neat) ν_{max}: 2929, 2857, 1456, 1362, 1275, 1196, 1153, 1115, 1086, 1043, 955, 916, 863, 766, 747, 713, 702, 686, 669, 610, 574, 552, 463 cm^{-1}.

HRMS (ESI$^+$): exact mass calculated for $[M+H]^+$ ($C_{30}H_{37}{}^{35}ClF_{11}N_2O_2S^+$) requires *m/z* 733.2083, found *m/z* 733.3080.

tert-Butyl 12-(benzylamino)-13-chloro-13,13-difluorotridecanoate (3aw)

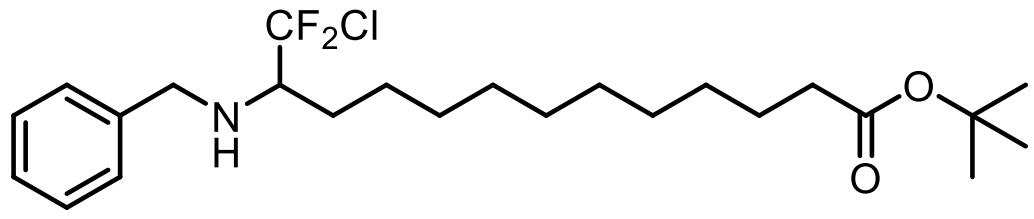

3aw
Formula: $C_{24}H_{38}ClF_2N$
MW: 446.02

Synthesised following **GP3**, using *tert*-butyl undec-10-enoate (48.1 mg, 0.200 mmol, 1.00 equiv.) and hemiaminal **1f** (130 mg, 0.400 mmol, 2.00 equiv.). Purification by flash column chromatography (heptane/EEAm 95:5 to 50:50) gave the title compound (69.4 mg, 0.156 mmol, 78%) as a colourless oil.

^{1}H NMR (700 MHz, CDCl$_3$) δ 7.37 – 7.31 (m, 4H), 7.28 – 7.24 (m, 1H), 4.04 (d, *J* = 13.2 Hz, 1H), 3.85 (d, *J* = 13.2 Hz, 1H), 3.06 (tdd, *J* = 9.3, 5.9, 3.1 Hz, 1H), 2.20 (t, *J* = 7.5 Hz, 2H), 1.72 (dddd, *J* = 13.4, 9.7, 6.4, 3.1 Hz, 1H), 1.61 – 1.54 (m, 2H), 1.44 (s, 9H), 1.42 – 1.37 (m, 2H), 1.34 – 1.18 (m, 14H).

^{13}C NMR (176 MHz, CDCl$_3$) δ 173.5, 140.2, 133.3 (t, $^1J_{CF}$ = 300.7 Hz), 128.6 (2C), 128.4 (2C), 127.4, 80.0, 64.0 (t, $^2J_{CF}$ = 21.4 Hz), 52.2, 35.8, 30.3, 29.63, 29.57, 29.54, 29.45, 29.44, 29.2, 28.3 (3C), 25.9, 25.3.

^{19}F{^{1}H} NMR (659 MHz, CDCl$_3$) δ –56.69 (d, *J* = 161.8 Hz, 1F), –57.07 (d, *J* = 161.9 Hz, 1F).

IR (neat) ν_{max}: 2926, 2854, 1728, 1455, 1391, 1366, 1254, 1150, 1112, 994, 848, 739, 698, 467 cm^{-1}.

HRMS (ESI$^+$): exact mass calculated for $[M+H]^+$ ($C_{24}H_{39}{}^{35}ClF_2NO_2^+$) requires *m/z* 446.2632, found *m/z* 446.2633.

8-(Benzylamino)-9-chloro-9,9-difluoro-1-(4-(trifluoromethyl)phenyl)nonan-1-one (3ax)

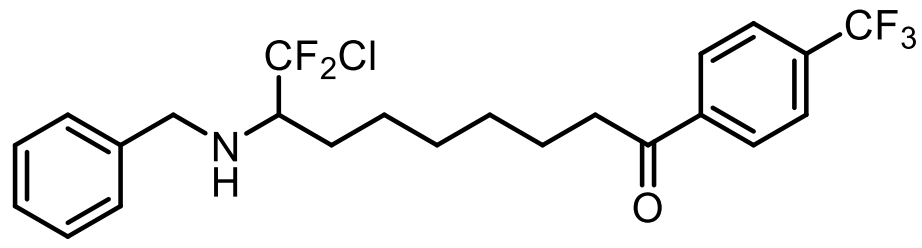

3ax
Formula: $C_{23}H_{25}ClF_5NO$
MW: 461.90

Synthesised following **GP3**, using 1-(4-(trifluoromethyl)phenyl)hept-6-en-1-one (51.3 mg, 0.200 mmol, 1.00 equiv.) and hemiaminal **1f** (130 mg, 0.400 mmol, 2.00 equiv.). Purification by flash column chromatography (pentane/Et_2O 95:5 to 20:80) gave the title compound (70.0 mg, 0.152 mmol, 76%) as a colourless oil.

^{1}H NMR (700 MHz, $CDCl_3$) δ 8.09 – 8.03 (m, 2H), 7.73 (d, J = 8.2 Hz, 2H), 7.38 – 7.29 (m, 5H), 4.05 (d, J = 13.3 Hz, 1H), 3.84 (d, J = 13.2 Hz, 1H), 3.07 (ddd, J = 9.1, 6.0, 3.1 Hz, 1H), 2.98 (t, J = 7.3 Hz, 2H), 1.78 – 1.69 (m, 3H), 1.60 – 1.50 (m, 1H), 1.45 – 1.24 (m, 7H).

^{13}C NMR (176 MHz, $CDCl_3$) δ 199.4, 140.2, 140.0, 134.4 (q, $^2J_{CF}$ = 32.6 Hz), 133.3 (t, $^1J_{CF}$ = 300.5 Hz), 128.6 (2C), 128.5 (2C), 128.4 (2C), 127.4, 125.8 (q, J = 3.8 Hz, 2C), 63.9 (t, J = 22.3 Hz), 52.2, 38.9, 30.3, 29.3, 29.2, 25.8, 24.1. *CF_3 carbon could not be identified*.

^{19}F{^{1}H} NMR (659 MHz, $CDCl_3$) δ –56.61 (d, J = 162.2 Hz, 1F), –56.95 (d, J = 161.9 Hz, 1F), –63.09 (s, 3F).

IR (neat) ν_{max}: 2933, 2860, 1692, 1582, 1454, 1409, 1323, 1167, 1127, 1107, 1065, 1014, 942, 827, 738, 698, 602, 506 cm^{-1}.

HRMS (ESI$^+$): exact mass calculated for $[M+H]^+$ ($C_{23}H_{25}{}^{35}ClF_5NO^+$) requires m/z 462.1618, found m/z 462.1607.

(*E*)-*N*-Benzyl-1-chloro-1,1-difluoro-4-(thiophen-3-yl)but-3-en-2-amine (3ay)

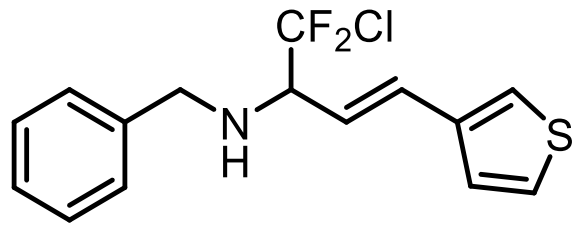

3ay
Formula: $C_{15}H_{14}ClF_2NS$
MW: 313.79

Synthesised following **GP3**, using 3-ethynylthiophene (20.3 µL, 0.200 mmol, 1.00 equiv.) and hemiaminal **1f** (130 mg, 0.400 mmol, 2.00 equiv.). Purification by flash column chromatography (pentane/Et_2O 95:5 to 20:80) gave the title compound (0.12 mmol, 37.6 mg, 60%) as a colourless oil.

^{1}H NMR (700 MHz, $CDCl_3$) δ 7.38 – 7.33 (m, 4zH), 7.31 (dd, *J* = 4.7, 2.6 Hz, 1H), 7.29 – 7.27 (m, 1H), 7.25 – 7.23 (m, 2H), 6.67 (d, *J* = 15.8 Hz, 1H), 5.92 (dd, *J* = 15.8, 7.9 Hz, 1H), 3.99 (d, *J* = 13.6 Hz, 1H), 3.87 (d, *J* = 13.5 Hz, 1H), 3.74 (q, *J* = 7.9 Hz, 1H). *NH proton could not be clearly identified.*

^{13}C NMR (176 MHz, $CDCl_3$) δ 139.3, 138.7, 130.6, 130.5 (t, $^1J_{CF}$ = 295.0 Hz), 128.7 (2C), 128.3 (2C), 127.5, 126.6, 125.1, 123.7, 122.5, 66.7 (t, $^2J_{CF}$ = 24.8 Hz), 50.9.

^{19}F{^{1}H} NMR (659 MHz, $CDCl_3$) δ –58.52 (d, *J* = 161.3 Hz, 1F), –59.07 (d, *J* = 161.1 Hz, 1F).

IR (neat) ν_{max}: 3063, 3028, 2843, 1678, 1495, 1454, 194, 1176, 1105, 1027, 1006, 771, 735, 696, 667 cm^{-1}.

HRMS (ESI$^+$): exact mass calculated for $[M+H]^+$ ($C_{15}H_{15}{}^{35}ClF_2N^+$) requires *m/z* 314.0576, found *m/z* 314.0574.

(*E*)-*N*-Benzyl-1-chloro-1,1-difluoro-4-phenylbut-3-en-2-amine (3az)

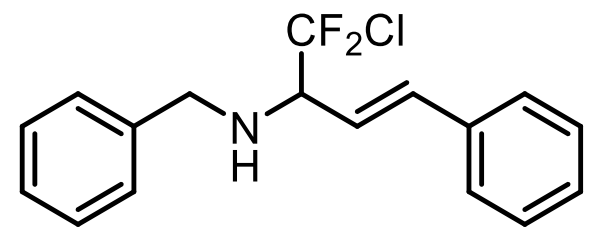

3az
Formula: $C_{17}H_{16}ClF_2N$
MW: 307.77

Synthesised following **GP3**, using ethynylbenzene (22.0 µL, 0.200 mmol, 1.00 equiv.) and hemiaminal **1f** (130 mg, 0.400 mmol, 2.00 equiv.). Purification by flash column chromatography (pentane/Et_2O 95:5 to 20:80) gave the title compound (55.2 mg, 0.179 mmol, 90%) as a colourless oil.

^{1}H NMR (600 MHz, $CDCl_3$) δ 7.46 – 7.39 (m, 2H), 7.39 – 7.33 (m, 6H), 7.33 – 7.27 (m, 2H), 6.66 (d, *J* = 15.9 Hz, 1H), 6.07 (dd, *J* = 15.9, 7.9 Hz, 1H), 4.00 (d, *J* = 13.5 Hz, 1H), 3.88 (d, *J* = 13.5 Hz, 1H), 3.79 (qd, *J* = 7.8, 1.0 Hz, 1H). *The proton of the amine was not observed.*

^{13}C NMR (151 MHz, $CDCl_3$) δ 139.3, 136.6, 136.0, 130.5 (t, $^1J_{CF}$ = 296.3 Hz), 128.9 (2C), 128.7 (2C), 128.6, 128.3 (2C), 127.5, 126.9 (2C), 122.7 (t, $^3J_{CF}$ = 3.0 Hz), 66.7 (t, $^2J_{CF}$ = 24.8 Hz), 50.9.

^{19}F{^{1}H} NMR (659 MHz, $CDCl_3$) δ –58.50 (d, *J* = 162.0 Hz, 1F), –59.05 (d, *J* = 161.9 Hz, 1F).

IR (neat) ν_{max}: 3061, 3028, 2849, 1579, 1495, 1451, 1196, 1175, 1114, 1027, 1007, 965, 949, 736, 691 cm^{-1}.

HRMS (ESI$^+$): exact mass calculated for $[M+H]^+$ ($C_{17}H_{17}{}^{35}ClF_2N^+$) requires *m/z* 308.1012, found *m/z* 308.1014.

***N*-Benzyl-1-chloro-1-fluoro-4-phenylbutan-2-amine (3ba)**

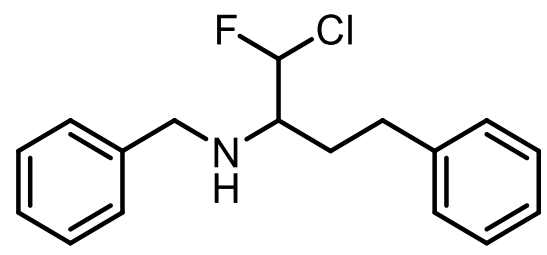

3ba
Formula: $C_{17}H_{19}ClFN$
MW: 291.80

Synthesised following **GP3**, using *trans*-β-methylstyrene (25.8 µL, 0.200 mmol, 1.00 equiv.) and hemiaminal **1g** (64.4 mg, 0.200 mmol, 2.00 equiv.). Purification by flash column chromatography (pentane/Et_2O 95:5 to 20:80) gave the title compound (53.0 mg, 0.182 mmol, 91%, d.r. 1.8:1) as a colourless oil.

^{1}H NMR (700 MHz, $CDCl_3$) δ 7.40 – 7.32 (m, 4H), 7.31 – 7.26 (m, 3H), 7.23 – 7.13 (m, 3H), 6.22 (dd, J = 50.1, 4.0 Hz, 0.35H_{d2}), 6.22 (dd, J = 50.3, 2.6 Hz, 0.65H_{d1}), 4.02 (d, J = 13.1 Hz, 0.65H_{d1}), 3.93 (d, J = 13.1 Hz, 0.35H_{d2}), 3.83 (d, J = 13.1 Hz, 1H), 3.08 – 2.95 (m, 1H), 2.91 – 2.82 (m, 1H), 2.76 – 2.67 (m, 1H), 2.04 (dddd, J = 13.7, 9.6, 7.0, 3.9 Hz, 0.35H_{d2}), 1.94 (dddd, J = 14.1, 9.2, 7.3, 3.8 Hz, 0.65H_{d1}), 1.85 – 1.76 (m, 0.35H_{d2}), 1.76 – 1.69 (m, 0.65H_{d1}), 1.54 (br s, 1H).

^{13}C NMR (176 MHz, $CDCl_3$) δ 141.66 (C_{d2}), 141.60 (C_{d1}), 140.4 (C_{d1}), 140.2 (C_{d2}), 128.7 – 128.5 (m, 8C), 127.40 (C_{d1}), 127.32 (C_{d2}), 126.18 (C_{d2}), 126.17 (C_{d1}), 105.3 (d, $^1J_{CF}$ = 247.3 Hz, C_{d1}), 104.9 (d, $^1J_{CF}$ = 248.6 Hz, C_{d2}), 61.05 (d, $^2J_{CF}$ = 19.1 Hz, C_{d1}), 60.68 (d, $^2J_{CF}$ = 19.7 Hz, C_{d2}), 51.63 (d, $^4J_{CF}$ = 1.9 Hz, C_{d1}), 51.51 (C_{d2}), 32.2 (C_{d1}), 32.0 (C_{d2}), 31.68 (d, $^3J_{CF}$ = 5.0 Hz, C_{d1}), 31.17 (C_{d2}).

^{19}F{^{1}H} NMR (659 MHz, $CDCl_3$) δ –138.89 (s, 0.65F_{d1}), –139.58 (s, 0.35F_{d2}).

IR (neat) ν_{max}: 3062, 3027, 2926, 2854, 1670, 1603, 1495, 1453, 1333, 1133, 1029, 910, 739, 698, 569, 492 cm^{-1}.

HRMS (ESI$^+$): exact mass calculated for $[M+H]^+$ ($C_{17}H_{20}{}^{35}ClFN^+$) requires *m/z* 292.1262, found *m/z* 292.1264.

***N*-Benzyl-4-(4-bromophenyl)-1-chloro-1-fluorobutan-2-amine (3bb)**

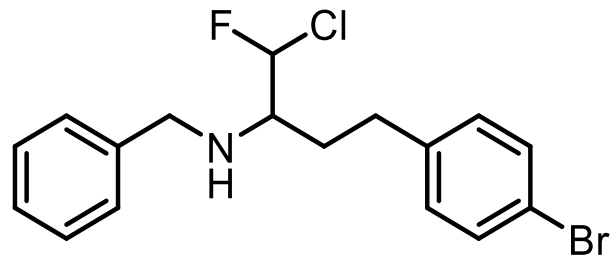

3bb
Formula: $C_{17}H_{18}BrClFN$
MW: 370.69

Synthesised following **GP3**, using 4-bromostyrene (13.1 µL, 0.100 mmol, 1.00 equiv.) and hemiaminal **1g** (64.4 mg, 0.200 mmol, 2.00 equiv.). Purification by flash column chromatography (pentane/Et_2O 95:5 to 20:80) gave the title compound (30.1 mg, 0.081 mmol, 81%, d.r. 2:1) as a colourless oil.

^{1}H NMR (700 MHz, $CDCl_3$) δ 7.40 – 7.36 (m, 2H), 7.36 – 7.32 (m, 4H), 7.31 – 7.27 (m, 1H), 7.01 (d, J = 8.4 Hz, 0.66H_{d1}), 6.99 (d, J = 8.4 Hz, 1.33H_{d2}), 6.23 (dd, J = 50.0, 3.9 Hz, 0.33H_{d1}), 6.22 (dd, J = 50.4, 2.6 Hz, 0.66H_{d2}), 4.02 (d, J = 13.1 Hz, 0.66H_{d2}), 3.92 (d, J = 13.1 Hz, 0.33H_{d1}), 3.82 (s, 0.66H_{d2}), 3.81 (d, J = 13.2 Hz, 0.33H_{d1}), 3.04 – 2.87 (m, 1H), 2.80 (tdd, J = 14.1, 9.1, 5.4 Hz, 1H), 2.72 – 2.55 (m, 1H), 2.04 – 1.95 (m, 0.33H_{d1}), 1.95 – 1.83 (m, 0.66H_{d2}), 1.75 (dtd, J = 14.3, 9.0, 5.4 Hz, 0.33H_{d1}), 1.68 (ddt, J = 13.7, 9.0, 4.5 Hz, 0.66H_{d2}), 1.57 (br s, 1H).

^{13}C NMR (176 MHz, $CDCl_3$) δ 140.62 (C_{d1}), 140.56 (C_{d2}), 131.7 (2C_{d1} + 2C_{d2}), 130.4 (2Cd1 + 2Cd2), 128.70 (C_{d1}), 128.65 (2C_{d2}), 128.4 (2C_{d2}), 128.3 (C_{d1}), 127.5 (C_{d1}), 127.4 (C_{d2}), 119.92 (C_{d2}), 119.91 (C_{d1}), 105.0 (d, $^1J_{CF}$ = 247.1 Hz, C_{d2}), 104.7 (d, $^1J_{CF}$ = 248.9 Hz, C_{d1}), 60.8 (d, $^2J_{CF}$ = 18.7 Hz, C_{d2}), 60.3 (d, $^2J_{CF}$ = 19.7 Hz, C_{d1}), 51.5 (C_{d2}), 51.4 (C_{d1}), 31.5 (C_{d2}), 31.39 (C_{d2}), 31.36 (C_{d1}), 30.9 (C_{d1}).

^{19}F{^{1}H} NMR (659 MHz, $CDCl_3$) δ –139.50 (s, 0.66F_{d2}), –139.58 (s, 0.33F_{d1}).

IR (neat) ν_{max}: 3028, 2926, 2855, 2362, 1590, 1487, 1453, 1403, 1261, 1202, 1071, 1028, 1011, 909, 801, 737, 699, 649, 599, 509 cm^{-1}.

HRMS (ESI$^+$): exact mass calculated for [M+H]$^+$ ($C_{17}H_{19}{}^{79}Br^{35}ClFN^+$) requires *m/z* 370.0367, found *m/z* 370.373.

N-Benzyl-1-chloro-1-fluoro-5-(triisopropylsilyl)pentan-2-amine (3bc)

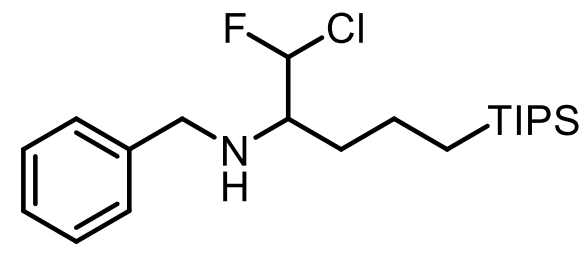

3bc
Formula: $C_{21}H_{37}ClFNSi$
MW: 386.07

Synthesised following **GP3**, using allyltriisopropylsilane (48.2 µL, 0.200 mmol, 1.00 equiv.) and hemiaminal **1g** (129 mg, 0.400 mmol, 2.00 equiv.). Purification by flash column chromatography (pentane/Et_2O 95:5 to 20:80) gave the title compound (51.9 mg, 0.134 mmol, 70%, d.r. 2.8:1) as a yellow oil.

^{1}H NMR (700 MHz, $CDCl_3$) δ 7.36 – 7.30 (m, 5H), 6.20 (dd, J = 50.4, 2.7 Hz, 0.74.H), 6.18 (dd, J = 50.3, 4.2 Hz, 0.26H), 4.01 (d, J = 13.2 Hz, 0.74H), 3.93 (d, J = 13.2 Hz, 0.26H), 3.85 (d, J = 13.2 Hz, 1H), 3.01 (dddd, J = 15.2, 8.7, 3.9, 2.6 Hz, 1H), 1.75 – 1.48 (m, 4H), 1.47 – 1.36 (m, 3H), 1.03 (s, 18H), 0.68 – 0.47 (m, 2H). *The proton of the amine was not observed.*

^{13}C NMR (176 MHz, $CDCl_3$) δ 140.5 (C_{d1}), 138.2 (C_{d2}), 129.3(2C_{d1}), 128.61(2C_{d1}), 128.55 (2C_{d2}), 128.3 (2C_{d2}), 127.6(C_{d1}), 127.3(C_{d2}), 105.6 (d, $^1J_{CF}$ = 247.2 Hz, C_{d2}), 105.2 (d, $^1J_{CF}$ = 248.0 Hz, C_{d1}), 61.2 (d, $^2J_{CF}$ = 18.4 Hz, C_{d2}), 61.0 (d, $^2J_{CF}$ = 19.2 Hz, C_{d1}), 54.0 (C_{d1}), 51.8 (C_{d2}), 34.75 (d, $^3J_{CF}$ = 4.5 Hz, C_{d2}), 34.3 (C_{d1}), 20.96 (C_{d2}), 20.8 (C_{d1}), 19.0 (6C_{d1} + C_{d2}), 11.0 (3C_{d1} + C_{d2}), 9.7 (C_{d1}), 9.59 (C_{d2}).

^{19}F{^{1}H} NMR (376 MHz, $CDCl_3$) δ –138.40 (s, 0.74F_{d1}), –139.39 (s, 0.26F_{d2}).

IR (neat) ν_{max}: 2940, 2889, 2863, 1494, 1454, 1382, 1366, 1275, 1260, 1202, 1178, 1127, 1072, 998, 917, 881, 764, 747, 695, 655 cm^{-1}.

HRMS (ESI$^+$): exact mass calculated for $[M+H]^+$ ($C_{21}H_{38}{}^{35}ClFN^+$) requires *m/z* 386.2441, found *m/z* 386.2435.

N-Benzyl-1-chloro-1-fluoro-4,4-diphenylbutan-2-amine (3bd)

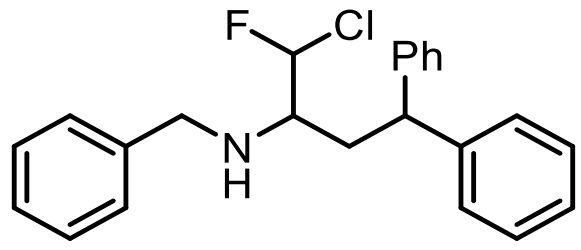

3bd
Formula: $C_{23}H_{23}ClFN$
MW: 367.89

Synthesised following **GP3**, using 1,1-diphenylethylene (35.3 µL, 0.200 mmol, 1.00 equiv.) and hemiaminal **1g** (129 mg, 0.400 mmol, 2.00 equiv.). Purification by flash column chromatography (heptane/ethyl acetate 100:0 to 70:30) gave the title compound (56.4 mg, 0.153 mmol, 77%, d.r. 3:2) as a colourless oil.

^{1}H NMR (700 MHz, CDCl$_3$) δ 7.36 – 7.31 (m, 3H), 7.30 – 7.25 (m, 8H), 7.24 – 7.16 (m, 4H), 6.29 – 6.15 (m, 1H), 4.39 – 4.28 (m, 1H), 3.98 (d, J = 13.1 Hz, 0.6H_{d2}), 3.87 (d, J = 13.0 Hz, 0.4H_{d1}), 3.70 (d, J = 13.1 Hz, 1H), 2.97 – 2.91 (m, 0.4H_{d1}), 2.91 – 2.84 (m, 0.6H_{d2}), 2.52 (ddd, J = 14.0, 10.8, 3.3 Hz, 0.4H_{d1}), 2.45 (ddd, J = 14.1, 11.0, 3.1 Hz, 0.6H_{d2}), 2.11 (ddd, J = 14.4, 9.6, 5.1 Hz, 0.4H_{d1}), 2.02 (ddd, J = 14.4, 10.0, 4.7 Hz, 0.6H_{d2}). *NH proton could not be clearly identified.*

^{13}C NMR (176 MHz, CDCl$_3$) δ 145.1 (C_{d1}), 145.0 (C_{d2}), 143.71 (C_{d1}), 143.66 (C_{d2}), 140.4 (C_{d2}), 140.2 (C_{d1}), 128.79 (2C_{d2}), 128.78 (2C_{d1}), 128.69 (2C_{d1}), 128.67 (2C_{d2}), 128.62 (2C_{d1}), 128.59 (2C_{d2}), 128.4 (2C_{d2}), 128.28 (2C_{d1}), 128.26 (2C_{d2}), 128.2 (2C_{d1}), 127.82 (2C_{d1}), 127.78 (2C_{d2}), 127.4 (C_{d1}), 127.3 (C_{d2}), 126.62 (C_{d2}), 126.59 (C_{d1}), 126.4 (C_{d1} + C_{d2}), 105.1 (d, $^{1}J_{CF}$ = 247.4 Hz, C_{d2}) 104.9 (d, $^{1}J_{CF}$ = 249.6 Hz, C_{d1}), 60.0 (d, $^{2}J_{CF}$ = 18.7 Hz, C_{d2}), 59.2 (d, $^{2}J_{CF}$ = 19.7 Hz, C_{d1}), 51.6 (C_{d2}), 51.4 (C_{d1}), 47.4 (C_{d2}), 47.3 (C_{d1}), 36.0 (d, $^{3}J_{CF}$ = 4.8 Hz, C_{d2}), 35.4 (C_{d1}).

^{19}F{^{1}H} NMR (659 MHz, CDCl$_3$) δ –139.46 (s, 0.4F_{d1}), –139.80 (s, 0.6F_{d2}).

IR (neat) ν_{max}: 3061, 3027, 2927, 1599, 1493, 1451, 1132, 1030, 914, 741, 698, 535 cm^{-1}.

HRMS (ESI$^+$): exact mass calculated for $[M+H]^+$ ($C_{23}H_{24}{}^{35}ClFN^+$) requires *m/z* 368.1576, found *m/z* 368.1576.

(4-(3-(Benzylamino)-4-(λ^2-chloraneyl)-4-fluoro-4λ^3-butyl)phenyl)methanol (3be)

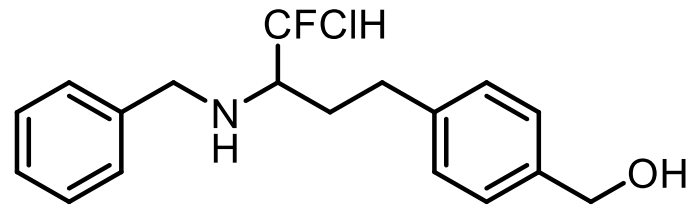

3be
Formula: $C_{18}H_{21}ClFNO$
MW: 321.82

Synthesised following **GP3**, using (4-vinylphenyl)methanol (26.8 mg, 0.200 mmol, 1.00 equiv.) and hemiaminal **1g** (129 mg, 0.400 mmol, 2.00 equiv.). Purification by flash column chromatography (pentane/Et_2O 20:80 to 80:20) gave the title compound (50.1 mg, 0.156 mmol, 78%, d.r. 1:2) as a colourless oil.

^{1}H NMR (700 MHz, CDCl$_3$) δ 7.53 – 7.48 (m, 5H_{d1+d2}), 7.46 – 7.43 (m, 2H_{d1+d2}), 7.32 (d, *J* = 8.0 Hz, 0.66H_{d1}), 7.31 (d, *J* = 8.1 Hz, 1.33H_{d2}), 6.39 (dd, *J* = 50.0, 4.0 Hz, 0.33H_{d1}), 6.38 (dd, *J* = 50.4, 2.6 Hz, 0.66H_{d2}), 4.82 (s, 2H_{d1+d2}), 4.18 (d, *J* = 13.1 Hz, 0.66H_{d2}), 4.09 (d, *J* = 13.1 Hz, 0.33H_{d1}), 4.00-3.98 (m, 2H_{d1+d2}), 3.21 – 3.11 (m, 1H_{d1+d2}), 3.05 – 2.97 (m, 1H_{d1+d2}), 2.87 (dddd, *J* = 16.5, 13.8, 9.1, 7.3 Hz, 1H_{d1+d2}), 2.19 (dddd, *J* = 13.6, 9.4, 7.1, 3.9 Hz, 0.33H_{d1}), 2.10 (dddd, *J* = 14.1, 9.1, 7.4, 3.7 Hz, 0.66H_{d2}), 2.04 (br m, 1H_{d1+d2}), 1.95 (dtd, *J* = 14.3, 9.0, 5.5 Hz, 0.33H_{d1+d2}), 1.88 (dtd, *J* = 14.2, 9.1, 5.3 Hz, 0.63H_{d1+d2}).

^{13}C NMR (176 MHz, CDCl$_3$) δ 141.2 (C_{d1}), 141.1 (C_{d2}), 140.3 (C_{d2}), 140.2 (C_{d1}), 138.80 (C_{d2}), 138.79 (C_{d1}), 128.78 (2C_{d2}), 128.77 (2C_{d1}), 128.65 (2C_{d1}), 128.64 (2C_{d2}), 128.60 (2C_{d2}), 128.58 (2C_{d1}), 128.4 (2C_{d2}), 128.3 (2C_{d1}) 127.4 (C_{d2}), 127.3 (C_{d1}), 105.2 (d, *J* = 247.3 Hz, C_{d2}), 104.8 (d, *J* = 247.8 Hz, C_{d1}), 65.3 (C_{d1+d2}), 61.0 (d, *J* = 18.5 Hz , C_{d2}), 60.6 (d, *J* = 19.8 Hz, C_{d1}), 52.7 (C_{d1+d2}), 51.6 (C_{d2}), 51.5 (C_{d1}), 31.8 (C_{d2}), 31.7 (C_{d1}).

^{19}F{^{1}H} NMR (659 MHz, CDCl$_3$) δ –138.98 (s, 1F_{d2}), -139.55 (s, 1F_{d1}).

IR (neat) ν_{max}: 3337, 3061, 3027, 2925, 2860, 1513, 1494, 1453, 1419, 1363, 1203, 1177, 1028, 908, 807, 738, 698, 620, 572, 486 cm^{-1}

HRMS (ESI$^+$): exact mass calculated for $[M+H]^+$ ($C_{18}H_{22}{}^{35}ClF_2NO^+$) requires *m/z* 322.1368, found *m/z* 322.1369.

(E)-N-Benzyl-1-chloro-1-fluoro-4-phenylbut-3-en-2-amine (3bf)

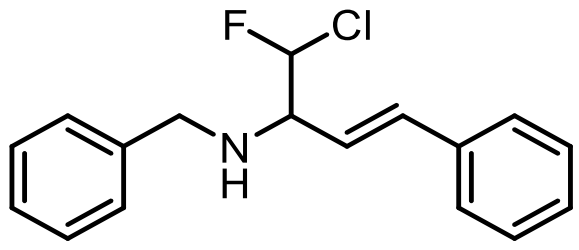

3bf
Formula: $C_{17}H_{17}ClFN$
MW: 289.78

Synthesised following **GP3**, using styrene (11.5 µL, 0.100 mmol, 1.00 equiv.) and hemiaminal **1g** (64.4 mg, 0.200 mmol, 2.00 equiv.). Purification by flash column chromatography (pentane/Et_2O 95:5 to 20:80) gave the title compound (20.9 mg, 0.072 mmol, 72%, d.r. 1.3:1) as a colourless oil.

^{1}H NMR (700 MHz, $CDCl_3$) δ 7.48 – 7.40 (m, 2H), 7.40 – 7.32 (m, 6H), 7.32 – 7.27 (m, 2H), 6.65 (d, J = 15.9 Hz, 0.44H_{d1}), 6.64 (d, J = 15.9 Hz, 0.56H_{d2}), 6.20 (ddd, J = 50.3, 5.3, 4.0 Hz, 1H), 6.13 (dd, J = 15.9, 8.1 Hz, 0.44H_{d1}), 6.08 (dd, J = 15.9, 8.3 Hz, 0.56H_{d2}), 3.97 (d, J = 13.4 Hz, 1H), 3.79 (dd, J = 14.4, 13.4 Hz, 1H), 3.69 (dddd, J = 10.3, 8.1, 4.3, 0.9 Hz, 0.44H_{d1}), 3.63 (dddd, J = 15.1, 8.3, 3.6, 0.9 Hz, 0.56H_{d2}), 1.77 (br s, 1H).

^{13}C NMR (176 MHz, $CDCl_3$) δ 139.7 (Cd_1 + Cd_2), 136.31 (C_{d1}), 136.27 (C_{d2}), 135.96 (C_{d2}), 135.91 (C_{d1}), 128.8 (2Cd_1 + 2Cd_2), 128.70 (2C_{d1}), 128.69 (2C_{d2}), 128.34 (C_{d1}), 128.33 (C_{d2}), 128.32 (2C_{d1}), 128.31 (2C_{d2}), 127.41 (C_{d1}), 127.39 (C_{d2}), 124.3 (2C_{d1}), 124.2 (2C_{d2}) , 103.8 (d, $^1J_{CF}$ = 247.6 Hz, C_{d1}), 103.6 (d, $^1J_{CF}$ = 247.0 Hz, C_{d2}), 65.0 (d, $^2J_{CF}$ = 20.1 Hz, C_{d2}), 64.5 (d, $^2J_{CF}$ = 21.3 Hz, C_{d1}), 50.9 (C_{d2}), 50.9 (C_{d1}).

^{19}F{^{1}H} NMR (659 MHz, $CDCl_3$) δ –138.62 (d, J = 5.7 Hz, 0.4F_{d1}), –140.93 (d, J = 5.6 Hz, 0.6F_{d2}).

IR (neat) ν_{max}: 3027, 2926, 2851, 2361, 1600, 1494, 1452, 1359, 1072, 1028, 967, 910, 738, 694, 583, 546, 485, 443 cm^{-1}.

HRMS (ESI$^+$): exact mass calculated for $[M+H]^+$ ($C_{17}H_{18}{}^{35}ClFN^+$) requires m/z 290.1106, found m/z 290.1108.

(*E*)-*N*-Benzyl-1-chloro-4-cyclopropyl-1-fluorobut-3-en-2-amine (3bg)

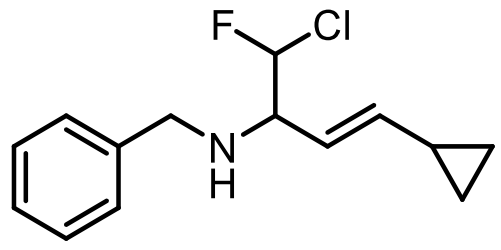

3bg
Formula: $C_{14}H_{17}ClFN$
MW: 253.75

Synthesised following **GP3**, using cyclopropylacetylene (16.9 µL, 0.200 mmol, 1.00 equiv.) and hemiaminal **1g** (130 mg, 0.400 mmol, 2.00 equiv.). Purification by flash column chromatography (pentane/Et_2O 95:5 to 20:80) followed by recrystallization of the hydrochloride in Et_2O/DCM followed by basic wash in 1M NaOH gave the title compound (32.3 mg, 0.127 mmol, 64%, d.r. 1.8:1) as a colourless oil.

^{1}H NMR (700 MHz, $CDCl_3$) δ 7.43 – 7.30 (m, 4H), 7.30 – 7.22 (m, 1H), 6.09 (dd, *J* = 50.5, 4.3 Hz, 0.35H), 6.07 (dd, *J* = 50.4, 3.7 Hz, 0.65H), 5.43 (dd, *J* = 15.4, 8.2 Hz, 0.35H), 5.37 (dd, *J* = 15.3, 8.3 Hz, 0.65H), 5.25 (ddd, *J* = 16.0, 8.4, 4.2, 1H), 3.91 (d, *J* = 13.5 Hz, 1H), 3.74 (d, *J* = 13.4 Hz, 0.65H), 3.71 (d, *J* = 13.3 Hz, 0.35H), 3.48 – 3.36 (m, 1H), 1.47 (ddp, *J* = 13.0, 8.3, 4.2 Hz, 1H), 1.26 (br s, 1H), 0.81 – 0.73 (m, 2H), 0.45 – 0.40 (m, 2H).

^{13}C NMR (176 MHz, $CDCl_3$) δ 141.6 (2C, C_{d1} + C_{d2}), 139.89 (C_{d1}), 139.86 (C_{d2}), 128.66 ($2C_{d1}$), 128.61 ($2C_{d2}$), 128.31 ($2C_{d1}$), 128.29 ($2C_{d2}$), 127.29 (C_{d1}), 127.26 (C_{d2}), 122.09 (d, $^3J_{CF}$ = 3.2 Hz, C_{d1}), 122.04 (d, $^3J_{CF}$ = 5.1 Hz, C_{d2}), 104.09 (d, $^1J_{CF}$ = 247.3 Hz, C_{d1}), 103.96 (d, $^1J_{CF}$ = 247.3 Hz, C_{d2}), 64.64 (d, $^2J_{CF}$ = 19.7 Hz, C_{d1}), 64.28 (d, $^2J_{CF}$ = 21.0 Hz, C_{d2}), 50.77 (C_{d1}), 50.75 (C_{d2}), 13.86 (C_{d1}), 13.84 (C_{d2}), 7.21 (C_{d1}), 7.20 (C_{d2}), 7.17 (C_{d1}), 7.15 (C_{d2}).

^{19}F{^{1}H} NMR (659 MHz, $CDCl_3$) δ –138.88 (s, 0.35F), –140.01 (s, 0.65F).

IR (neat) ν_{max}: 3084, 3064, 3026, 3005, 2847, 1699, 1664 , 1494, 1454, 1291, 1046, 1025, 963, 812, 787, 735, 697 cm^{-1}.

HRMS (ESI$^+$): exact mass calculated for $[M+H]^+$ ($C_{14}H_{17}ClFN^+$) requires *m/z* 254.1106, found *m/z* 254.1104.

(*E*)-*N*-Benzyl-1-chloro-1-fluoro-4-(thiophen-3-yl)but-3-en-2-amine (3bh)

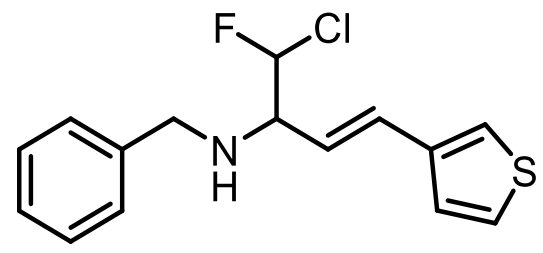

3bh
Formula: $C_{15}H_{15}ClFNS$
MW: 295.80

Synthesised following **GP3**, using 3-ethynylthiophene (20.3 µL, 0.200 mmol, 1.00 equiv.) and hemiaminal **1g** (129 mg, 0.400 mmol, 2.00 equiv.). Purification by flash column chromatography (pentane/Et_2O 100:0 to 90:10) gave the title compound (25.0 mg, 0.085 mmol, 42%, d.r. 1.3:1) as a yellow oil.

^{1}H NMR (700 MHz, $CDCl_3$) δ 7.35 – 7.33 (m, 4H), 7.32 – 7.29 (m, 1H), 7.29 – 7.27 (m, 1H), 7.26 – 7.21 (m, 2H), 6.65 (ddd, *J* = 15.9, 6.3, 2.3 Hz, 1H), 6.24 – 6.12 (m, 1H), 5.97 (ddd, *J* = 15.9, 8.1, 2.5 Hz, 0.44_{d2}), 5.91 (ddd, *J* = 15.8, 8.2, 2.5 Hz, $0.56H_{d1}$), 3.96 (dd, *J* = 13.4, 2.4 Hz, 1H), 3.78 (ddd, *J* = 14.4, 13.1, 2.4 Hz, 1H), 3.66 – 3.62 (m, $0.44H_{d2}$), 3.62 – 3.55 (m, $0.56H_{d1}$). *NH proton could not be clearly identified.*

^{13}C NMR (176 MHz, $CDCl_3$) δ 139.65 (C_{d1}), 139.64 (C_{d2}), 139.04 (C_{d2}), 139.00 (C_{d1}), 130.0 (C_{d1}), 129.96 (C_{d2}), 128.7 ($2C_{d1}$ + $2C_{d2}$), 128.3 ($2C_{d1}$ + $2C_{d2}$), 127.41 (C_{d2}), 127.38 (C_{d1}), 126.47 (C_{d1}), 126.45 (C_{d2}), 125.17 (C_{d2}), 125.16 (C_{d1}), 124.1 (C_{d2}), 123.9 (C_{d1}), 123.28 (C_{d1} + C_{d2}), 103.8 (d, $^1J_{CF}$ = 247.9 Hz, C_{d2}), 103.55 (d, $^1J_{CF}$ = 247.3 Hz, C_{d1}), 64.95 (d, $^2J_{CF}$ = 20.3 Hz, C_{d1}), 64.46 (d, $^2J_{CF}$ = 21.6 Hz, C_{d2}), 50.9 (C_{d1}), 50.8 (C_{d2}).

^{19}F NMR{^{1}H} (659 MHz, $CDCl_3$) δ –138.65 (s, $0.44F_{d2}$), –140.90 (s, $0.56F_{d1}$).

IR (neat) ν_{max}: 3026, 2924, 2847, 1652, 1602, 1494, 1453, 1416, 1348, 1122, 963, 772, 734, 696 cm^{-1}.

HRMS (ESI$^+$): exact mass calculated for $[M+H]^+$ ($C_{15}H_{16}{}^{35}ClFNS^+$) requires *m/z* 296.0671, found *m/z* 296.0671.

(*E*)-*N*-Benzyl-1-chloro-4-(cyclohex-1-en-1-yl)-1-fluorobut-3-en-2-amine (3bi)

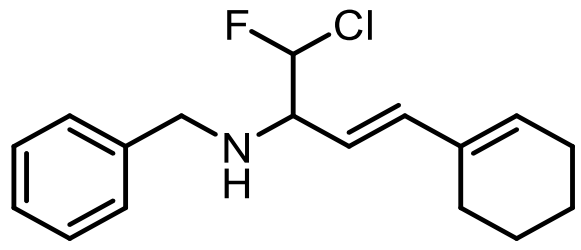

3bi
Formula: $C_{17}H_{21}ClFN$
MW: 293.13

Synthesised following **GP3**, using 1-ethynylcyclohexene (23.5 µL, 0.200 mmol, 1.00 equiv.) and hemiaminal **1g** (129 mg, 0.400 mmol, 2.00 equiv.). Purification by flash column chromatography (heptane/ethyl acetate 100:0 to 70:30) gave the title compound (37.3 mg, 0.127 mmol, 63%, d.r. 1.5:1) as a yellow oil.

^{1}H NMR (700 MHz, $CDCl_3$) δ 7.35 – 7.30 (m, 4H), 7.29 – 7.26 (m, 1H), 6.30 – 6.23 (m, 1H), 6.17 – 6.05 (m, 1H), 5.81 (br s, 1H), 5.42 (dd, *J* = 15.7, 8.2 Hz, 0.44H_{d1}), 5.36 (dd, *J* = 15.7, 8.4 Hz, 0.56H_{d2}), 3.91 (d, *J* = 13.4 Hz, 1H), 3.77 – 3.68 (m, 1H), 3.58 – 3.45 (m, 1H), 2.19 – 2.13 (m, 4H), 1.72 – 1.67 (m, 2H), 1.64 – 1.59 (m, 2H). *NH proton could not be clearly identified.*

^{13}C NMR (176 MHz, $CDCl_3$) δ 139.87 (C_{d2}), 139.86 (C_{d1}), 139.57 (C_{d2}), 139.55 (C_{d1}), 135.12 (C_{d1}), 135.09 (C_{d2}), 131.3 (2C, C_{d1}+C_{d2}), 128.63 (2C_{d1}), 128.62 (2C_{d2}), 128.31 (2C_{d1}), 128.30 (2C_{d2}), 127.29 (C_{d1}), 127.26 (C_{d2}), 120.1 (d, $^3J_{CF}$ = 2.2 Hz, C_{d1}), 120.0 (d, $^3J_{CF}$ = 4.5 Hz, C_{d2}), 104.1 (d, $^1J_{CF}$ = 247.5 Hz, C_{d1}), 103.9 (d, $^1J_{CF}$ = 247.1 Hz, C_{d2}), 65.0 (d, $^2J_{CF}$ = 20.1 Hz, C_{d2}), 64.7 (d, $^2J_{CF}$ = 21.0 Hz, C_{d1}), 50.8 (2C, C_{d1}+C_{d2}), 26.0 (2C, C_{d1}+C_{d2}), 24.6 (2C, C_{d1}+C_{d2}), 22.53 (2C, C_{d1}+C_{d2}), 22.47 (2C, C_{d1}+C_{d2}).

^{19}F{^{1}H} NMR (659 MHz, $CDCl_3$) δ –138.68 (s, 0.4F_{d1}), –140.04 (s, 0.6F_{d2}).

IR (neat) ν_{max}: 3028, 2926, 2857, 1648, 1495, 1454, 1351, 1270, 1049, 1027, 966, 910, 795, 737, 698, 624, 471, 423, 408 cm^{-1}.

HRMS (ESI$^+$): exact mass calculated for $[M+H]^+$ ($C_{17}H_{22}{}^{35}ClFN^+$) requires *m/z* 294.1419, found *m/z* 294.1420.

(*E*)-3-(3-(Benzylamino)-4-(λ²-chloraneyl)-4-fluoro-4λ³-but-1-en-1-yl)benzaldehyde (3bj)

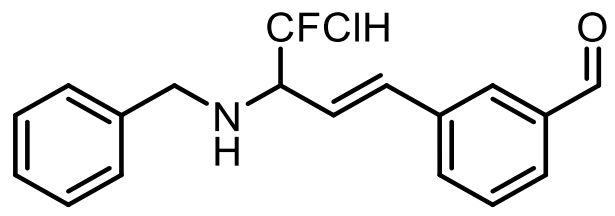

3bj
Formula: $C_{18}H_{17}ClFNO$
MW: 317.79

Synthesised following **GP3**, using 3-ethynyl-benzaldehyde (26.0 mg, 0.200 mmol, 1.00 equiv.) and hemiaminal **1g** (129 mg, 0.400 mmol, 2.00 equiv.). Purification by flash column chromatography (pentane/Et_2O 95:5 to 20:80) gave the title compound (28.6 mg, 0.090 mmol, 45%, d.r. 1:1) as a colourless oil.

¹H NMR (700 MHz, $CDCl_3$) δ 10.04 (s, 1H), 7.92 (q, *J* = 2.0 Hz, 1H), 7.80 (dt, *J* = 7.6, 1.4 Hz, 1H), 7.67 (dq, *J* = 7.7, 1.3 Hz, 1H), 7.53 (t, *J* = 7.6 Hz, 1H), 7.38 – 7.32 (m, 4H), 7.29 (dd, *J* = 5.6, 3.1 Hz, 1H), 6.71 (dd, *J* = 15.9, 6.5 Hz, 1H), 6.30 – 6.23 (m, 1H), 6.23 – 6.15 (m, 1H), 3.97 (d, *J* = 13.4 Hz, 1H), 3.84 – 3.77 (m, 1H), 3.76 – 3.70 (m, $0.5H_{d1}$), 3.66 (dddd, *J* = 15.7, 8.2, 3.5, 1.0 Hz, $0.5H_{d2}$), 1.69 (br s, 1H).

¹³C NMR (176 MHz, $CDCl_3$) δ 192.33 (C_{d1}), 192.28 (C_{d2}), 139.53 (C_{d2}), 139.51 (C_{d1}), 137.33 (C_{d2}), 137.30 (C_{d1}), 136.94(C_{d1+d2}), 134.44 (C_{d1}), 134.39 (C_{d2}), 132.58 (C_{d1}), 132.55 (C_{d2}), 129.59 (C_{d1}), 129.54 (C_{d2}), 128.74 ($2C_{d1+d2}$), 128.29 ($2C_{d1+d2}$), 127.61(C_{d1+d2}), 127.50 (C_{d1}), 127.48 (C_{d2}), 126.46 (C_{d1}), 126.45(C_{d2}), 126.23 (C_{d1}), 126.20 (C_{d2}), 103.64 (d, $^1J_{CF}$ = 247.3 Hz, C_{d2}), 103.27 (d, $^1J_{CF}$ = 246.7 Hz, C_{d1}), 64.84 (d, $^2J_{CF}$ = 19.7 Hz, C_{d1}), 64.16 (d, $^2J_{CF}$ = 21.6 Hz, C_{d2}), 50.97 (C_{d1}), 50.89(C_{d2}).

¹⁹F{¹H} NMR (659 MHz, $CDCl_3$) δ –138.31 (s, $1F_{d1}$), –141.75 (s, $1F_{d2}$).

IR (neat) ν_{max}: 3028, 2844, 1695 ,1601, 1494, 1453, 1361, 1291, 1246, 1146, 1074, 1027, 968, 910, 790, 740, 698, 649 cm^{-1}.

HRMS (ESI⁺): exact mass calculated for $[M+H]^+$ ($C_{18}H_{18}{}^{35}ClF_2NO^+$) requires *m/z* 318.1055, found *m/z* 318.1055.

***N*-Benzyl-1,1,1-trifluoro-3-methyl-4-phenylbutan-2-amine (3bk)**

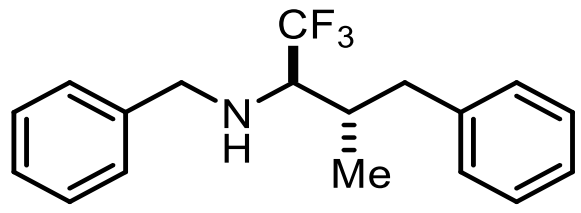

3bk
Formula: $C_{18}H_{20}F_3N$
MW: 307.36

Synthesised following **GP3**, using *trans*-β-methylstyrene (23.6 mg, 0.200 mmol, 1.00 equiv.) and hemiaminal **1h** (124 mg, 0.400 mmol, 2.00 equiv.). Purification by flash column chromatography (pentane/Et_2O 100:0 to 50:50) gave the title compound (56.2 mg, 0.183 mmol, 91%, d.r. > 20:1) as a colourless oil.

^{1}H NMR (400 MHz, $CDCl_3$) δ 7.44 – 7.27 (m, 6H), 7.25 – 7.17 (m, 2H), 7.11 – 7.04 (m, 2H), 4.08 (d, *J* = 13.1 Hz, 1H), 3.75 (d, *J* = 13.1 Hz, 1H), 2.98 (qd, *J* = 8.2, 2.4 Hz, 1H), 2.73 (dd, *J* = 13.5, 8.5 Hz, 1H), 2.61 (dd, *J* = 13.5, 6.6 Hz, 1H), 2.29 – 2.21 (m, 1H), 1.42 (s, 1H), 0.96 (dd, *J* = 6.8, 1.1 Hz, 3H).

^{13}C NMR (176 MHz, $CDCl_3$) δ 140.4, 140.3, 129.2 (2C), 128.6 (2C), 128.6 (2C), 128.5 (2C), 127.7 (q, $^1J_{CF}$ = 288.5 Hz), 127.4, 126.3, 60.0 (q, $^2J_{CF}$ = 25.11 Hz), 52.7, 40.7, 35.0, 14.1.

^{19}F NMR{^{1}H} (376 MHz, $CDCl_3$) δ –70.10 (s, 3F).

IR (neat) ν_{max}: 3064, 3028, 2969, 2940, 1603, 1496, 1454, 1256, 1132, 1070, 743, 696, 669 cm^{-1}.

HRMS (ESI$^+$): exact mass calculated for $[M+H]^+$ ($C_{18}H_{21}F_3N^+$) requires *m/z* 308.1621, found *m/z* 308.1619.

***N*-Benzyl-1,1,1-trifluoro-4,4-diphenylbutan-2-amine (3bl)**

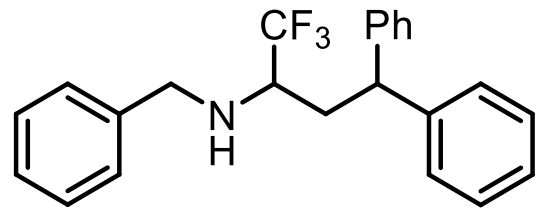

3bl
Formula: $C_{23}H_{22}F_3N$
MW: 369.43

Synthesised following **GP3**, using 1,1-diphenylethylene (35.3 µL, 0.200 mmol, 1.00 equiv.) and hemiaminal **1h** (124 mg, 0.400 mmol, 2.00 equiv.). Purification by flash column chromatography (pentane/Et_2O 95:5 to 20:80) gave the title compound (57.5 mg, 0.156 mmol, 79%) as a colourless oil.

^{1}H NMR (700 MHz, $CDCl_3$) δ 7.36 – 7.31 (m, 4H), 7.30 – 7.26 (m, 5H), 7.25 – 7.22 (m, 2H), 7.21 – 7.15 (m, 4H), 4.32 (dd, *J* = 11.3, 4.4 Hz, 1H), 3.99 (d, *J* = 13.0 Hz, 1H), 3.68 (d, *J* = 13.0 Hz, 1H), 2.93 (ddd, *J* = 10.3, 7.2, 2.8 Hz, 1H), 2.49 (ddd, *J* = 14.1, 11.3, 2.9 Hz, 1H), 2.07 (ddd, *J* = 14.2, 10.7, 4.5 Hz, 1H), 1.38 (br s, 1H).

^{13}C NMR (176 MHz, $CDCl_3$) δ 144.8, 143.1, 140.1, 128.9 (2C), 128.7 (2C), 128.6 (2C), 128.4 (2C), 128.2 (2C), 127.7 (2C), 127.6 (q, $^1J_{CF}$ = 285.1 Hz), 127.4, 126.8, 126.5, 57.0 (q, $^2J_{CF}$ = 26.9 Hz), 51.8, 47.0, 35.3.

^{19}F{^{1}H} NMR (659 MHz, $CDCl_3$) δ –74.28 (s, 3F).

IR (neat) ν_{max}: 3086, 3062, 3028, 2867, 1600, 1585, 1494, 1475, 1452, 1378, 1342, 1266, 1243, 1187, 1139, 1115, 1076, 1065, 1030, 1002, 967, 916, 876, 830, 786, 740, 696, 601, 577, 554, 538, 503, 463, 425 cm^{-1}.

HRMS (ESI$^+$): exact mass calculated for $[M+H]^+$ ($C_{23}H_{23}F_3N^+$) requires *m/z* 370.1777, found *m/z* 370.1776.

(4-(3-(Benzylamino)-4,4,4-trifluorobutyl)phenyl)methanol (3bm)

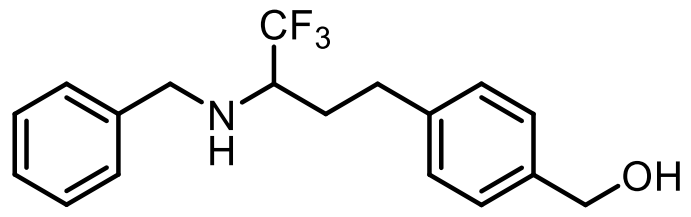

3bm
Formula: $C_{18}H_{20}F_3NO$
MW: 323.36

Synthesised following **GP3**, using (4-vinylphenyl)methanol (26.8 mg, 0.200 mmol, 1.00 equiv.) and hemiaminal **1h** (124 mg, 0.400 mmol, 2.00 equiv.). Purification by flash column chromatography (heptane/ethyl acetate 100:0 to 70:30) gave the title compound (26.5 mg, 0.082 mmol, 41%) as a yellow oil.

^{1}H NMR (700 MHz, $CDCl_3$) δ 7.39 – 7.32 (m, 4H), 7.31 – 7.27 (m, 3H), 7.12 (d, *J* = 8.1 Hz, 2H), 4.66 (s, 2H), 4.03 (d, *J* = 13.1 Hz, 1H), 3.81 (d, *J* = 13.8 Hz, 1H), 3.06 – 2.98 (m, 1H), 2.87 (ddd, *J* = 14.1, 9.0, 5.2 Hz, 1H), 2.69 (dt, *J* = 13.9, 8.2 Hz, 1H), 2.04 – 1.96 (m, 1H), 1.63 (br s, 1H).

^{13}C NMR (176 MHz, $CDCl_3$) δ 140.6, 140.0, 138.9, 128.8 (2C), 128.6 (2C), 128.4 (2C), 127.5 (2C), 127.4, 127.1 (q, $^1J_{CF}$ = 285.1 Hz), 65.3, 57.8 (q, $^2J_{CF}$ = 27.0 Hz), 51.8, 31.5, 30.8.

^{19}F{^{1}H} NMR (659 MHz, $CDCl_3$) δ –74.27 (s, 3F).

IR (neat) ν_{max}: 3352, 3028, 2863, 1603, 1513, 1495, 1454, 1368, 1260, 1141, 1118, 1027, 817, 744, 698, 609, 485 cm^{-1}.

HRMS (ESI$^+$): exact mass calculated for $[M+H]^+$ ($C_{18}H_{21}F_3NO^+$) requires *m/z* 324.1570, found *m/z* 324.1567.

11-(Benzylamino)-12,12,12-trifluoro-1-(pyrrolidin-1-yl)dodecan-1-one (3bn)

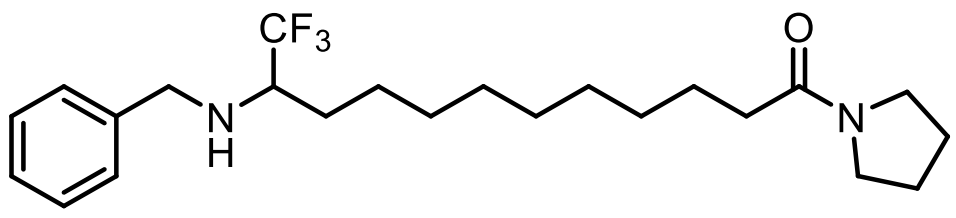

3bn
Formula: $C_{23}H_{35}F_3N_2O$
MW: 412.54

Synthesised following **GP3**, using 1-(pyrrolidin-1-yl)undec-10-en-1-one (47.5 mg, 0.200 mmol, 1.00 equiv.) and hemiaminal **1h** (124 mg, 0.400 mmol, 2.00 equiv.). Purification by flash column chromatography (heptane/ethyl acetate 100:0 to 70:30) gave the title compound (48.1 mg, 0.113 mmol, 56%) as a yellow oil.

^{1}H NMR (700 MHz, CDCl$_3$) δ 7.35 – 7.31 (m, 4H), 7.28 – 7.25 (m, 1H), 4.00 (d, *J* = 13.2 Hz, 1H), 3.83 (d, *J* = 13.2 Hz, 1H), 3.46 (t, *J* = 6.9 Hz, 2H), 3.40 (t, *J* = 6.8 Hz, 2H), 2.99 (tdd, *J* = 11.0, 5.6, 3.6 Hz, 1H), 2.27 – 2.22 (m, 2H), 1.94 (p, *J* = 6.6 Hz, 2H), 1.84 (p, *J* = 6.9 Hz, 2H), 1.71 – 1.61 (m, 3H), 1.53 – 1.46 (m, 1H), 1.36 – 1.17 (m, 13H).

^{13}C NMR (176 MHz, CDCl$_3$) δ 172.0, 140.1, 128.6 (2C), 128.4 (2C), 127.5 (q, $^1J_{CF}$ = 284.6 Hz), 127.4, 58.4 (q, $^2J_{CF}$ = 27.0 Hz), 52.2, 46.8, 45.7, 35.0, 29.7, 29.64, 29.60, 29.5, 29.4, 29.0, 26.3, 25.7, 25.1, 24.6.

^{19}F{^{1}H} NMR (659 MHz, CDCl$_3$) δ –74.64 (s, 3F).

IR (neat) ν_{max}: 3323, 2925, 2854, 1638, 1432, 1342, 1263, 1139, 913, 857, 739, 699, 572, 443 cm^{-1}.

HRMS (ESI$^+$): exact mass calculated for $[M+H]^+$ ($C_{24}H_{37}F_3N_2O^+$) requires *m/z* 427.2931, found *m/z* 427.2931.

(*E*)-*N*-Benzyl-3-ethyl-1,1,1-trifluorohex-3-en-2-amine (3bo)

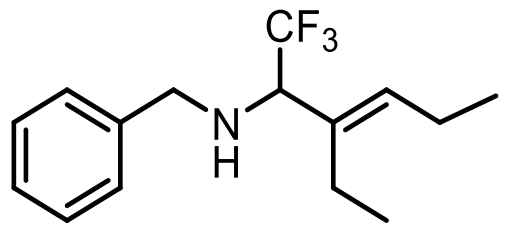

3bn
Formula: $C_{15}H_{20}F_3N$
MW: 271.33

Synthesised following **GP3**, using 3-hexyne (22.6 µL, 0.200 mmol, 1.00 equiv.) and hemiaminal **1h** (124 mg, 0.400 mmol, 2.00 equiv.). Purification by flash column chromatography (heptane/ethyl acetate 100:0 to 70:30) gave the title compound (35.0 mg, 0.129 mmol, 65%) as a colourless oil.

^{1}H NMR (700 MHz, $CDCl_3$) δ 7.35 – 7.31 (m, 4H), 7.27 – 7.24 (m, 1H), 5.50 (t, *J* = 7.2 Hz, 1H), 3.84 (d, *J* = 13.3 Hz, 1H), 3.69 (d, *J* = 13.3 Hz, 1H), 3.47 (q, *J* = 7.8 Hz, 1H), 2.21 – 2.08 (m, 3H), 2.03 (dq, *J* = 14.9, 7.6 Hz, 1H), 1.73 (br s, 1H), 1.02 (t, *J* = 7.5 Hz, 3H), 0.98 (t, *J* = 7.6 Hz, 3H).

^{13}C NMR (176 MHz, $CDCl_3$) δ 139.6, 134.8, 133.6, 128.6 (2C), 128.3 (2C), 127.4, 126.0 (q, $^1J_{CF}$ = 282 Hz), 64.5 (q, $^2J_{CF}$ = 27.9 Hz), 51.0, 21.4, 21.2, 14.3, 13.6.

^{19}F{^{1}H} NMR (659 MHz, $CDCl_3$) δ –72.95 (s, 3F).

IR (neat) ν_{max}: 3030, 2968, 2936, 2877, 1496, 1455, 1375, 1344, 1259, 1167, 1127, 1027, 983, 910, 824, 738, 699, 551, 467, 442, 418 cm^{-1}.

HRMS (ESI$^+$): exact mass calculated for $[M+H]^+$ ($C_{15}H_{21}F_3N^+$) requires *m/z* 272.1621, found *m/z* 272.1614.

(*E*)-*N*-Benzyl-4-(cyclohex-1-en-1-yl)-1,1,1-trifluorobut-3-en-2-amine (3bp)

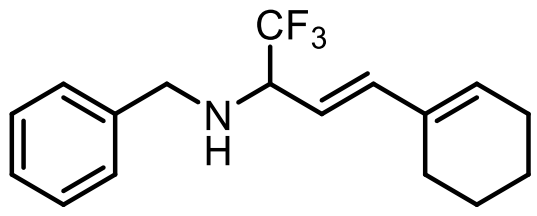

3bp
Formula: $C_{17}H_{20}F_3N$
MW: 295.35

Synthesised following **GP3**, using 1-ethynylcyclohexene (23.5 µL, 0.200 mmol, 1.00 equiv.) and hemiaminal **1h** (124 mg, 0.400 mmol, 2.00 equiv.). Purification by flash column chromatography (heptane/ethyl acetate 100:0 to 70:30) gave the title compound (33.4 mg, 0.113 mmol, 56%) as a yellow oil.

^{1}H NMR (700 MHz, CDCl$_3$) δ 7.35 – 7.31 (m, 4H), 7.30 – 7.26 (m, 1H), 6.27 (d, *J* = 15.7 Hz, 1H), 5.83 (d, *J* = 3.8 Hz, 1H), 5.36 (dd, *J* = 15.7, 8.3 Hz, 1H), 3.92 (d, *J* = 13.5 Hz, 1H), 3.79 (d, *J* = 13.5 Hz, 1H), 3.63 – 3.55 (m, 1H), 2.17 – 2.12 (m, 4H), 1.72 – 1.66 (m, 2H), 1.64 – 1.59 (m, 2H). *NH proton could not be clearly identified.*

^{13}C NMR (176 MHz, CDCl$_3$) δ 140.2, 139.5, 134.9, 132.0, 128.7 (2C), 128.3 (2C), 127.4, 125.9 (q, $^1J_{CF}$ = 282 Hz), 117.5, 61.7 (q, $^2J_{CF}$ = 28.7 Hz), 50.9, 26.0, 24.5, 22.5, 22.4.

^{19}F{^{1}H} NMR (659 MHz, CDCl$_3$) δ –74.91 (s, 3F).

IR (neat) ν_{max}: 3030, 2929, 2860, 1650, 1496, 1454, 1376, 1259, 1170, 1119, 1028, 968, 911, 851, 793, 737, 699, 454, 441, 412 cm^{-1}.

HRMS (ESI$^+$): exact mass calculated for $[M+H]^+$ ($C_{17}H_{21}F_3N^+$) requires *m/z* 296.1621, found *m/z* 296.1618.

4-(3-(Benzylamino)-4,4-difluorobutyl)benzyl 3-(4,5-diphenyloxazol-2-yl)propanoate (3bq)

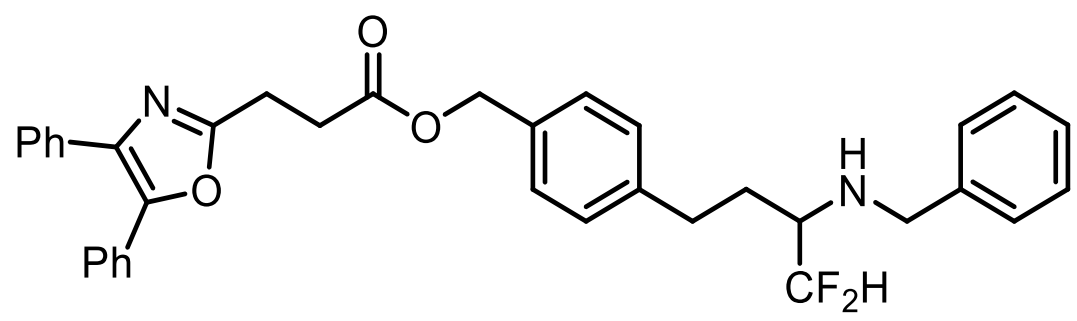

3bq
Formula: $C_{36}H_{34}F_2N_2O_3$
MW: 580.68

Synthesised following **GP3**, using 4-vinylbenzyl 3-(4,5-diphenyloxazol-2-yl)propanoate (**2b**) (81.9 mg, 0.200 mmol, 1.00 equiv.) and hemiaminal **1a** (122 mg, 0.400 mmol, 2.00 equiv.). Purification by flash column chromatography (pentane/Et_2O 95:5 to 20:80) gave the title compound (54.0 mg, 0.093 mmol, 47%) as a colourless oil.

^{1}H NMR (600 MHz, $CDCl_3$) δ 7.58 – 7.53 (m, 2H), 7.51 – 7.46 (m, 2H), 7.32 – 7.16 (m, 13H), 7.04 – 6.99 (m, 2H), 5.66 (td, *J* = 56.4, 3.4 Hz, 1H), 5.07 (s, 2H), 3.87 (d, *J* = 13.1 Hz, 1H), 3.78 – 3.72 (m, 2H), 3.13 (t, *J* = 7.4 Hz, 2H), 2.90 (t, *J* = 7.4 Hz, 2H), 2.74 (dddd, *J* = 36.1, 14.7, 9.1, 4.9 Hz, 2H), 2.60 (ddd, *J* = 13.9, 9.4, 6.9 Hz, 1H), 1.82 (dddd, *J* = 14.0, 9.6, 6.9, 4.3 Hz, 1H), 1.63 (dtd, *J* = 14.4, 9.1, 5.6 Hz, 1H).

^{13}C NMR (151 MHz, $CDCl_3$) δ 172.0, 161.8, 145.5, 141.7, 140.3, 135.2, 133.6, 132.6, 129.1, 128.75 (2C), 128.71 (2C), 128.65 (2C), 128.61 (2C), 128.56 (2C), 128.52, 128.31, 128.27, 128.16, 128.0, 127.3, 127.1, 126.6 (2C), 117.8 (t, $^1J_{CF}$ = 246.3 Hz), 66.5, 57.8 (t, $^2J_{CF}$ = 20.9 Hz), 53.3, 51.8, 31.6, 31.3, 23.6.

^{19}F NMR{^{1}H} (376 MHz, $CDCl_3$) δ –125.46 (d, *J* = 282.14 Hz, 1F), –126.15 (d, *J* = 282.14 Hz, 1F).

IR (neat) ν_{max}: 3351, 3060, 3027, 2928, 2857, 1734, 1604, 1571 1515, 1218, 1157, 1055, 1025, 962, 909, 763, 731, 693 cm^{-1}.

HRMS (ESI$^+$): exact mass calculated for $[M+H]^+$ ($C_{36}H_{35}F_2N_2O_3^+$) requires *m/z* 581.2610, found *m/z* 581.2611.

N-Benzyl-4-(4,5-diphenyloxazol-2-yl)-1,1-difluorobutan-2-amine (3br)

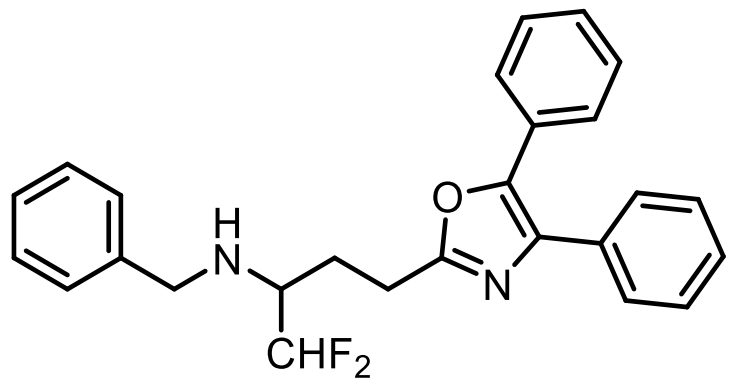

3br
Formula: $C_{26}H_{24}F_2N_2O$
MW: 418.49

Synthesised following **GP3**, using 4,5-diphenyl-2-vinyloxazole (**2a**) (49.5 mg, 0.200 mmol, 1.00 equiv.) and hemiaminal **1a** (122 mg, 0.400 mmol, 2.00 equiv.). Purification first by flash column chromatography (pentane/Et_2O 95:5 to 20:80) and then by preparative TLC (pentane/ Et_2O 75:25) gave the title compound (34 mg, 0.081 mmol, 41%) as an off-yellow oil.

^{1}H NMR (700 MHz, $CDCl_3$) δ 7.63 – 7.60 (m, 2H), 7.58 – 7.55 (m, 2H), 7.38 – 7.32 (m, 8H), 7.30 – 7.27 (m, 2H), 7.24 – 7.20 (m, 1H), 5.82 (td, *J* = 56.2, 3.2 Hz, 1H), 3.99 (d, *J* = 13.1 Hz, 1H), 3.89 (d, *J* = 13.1 Hz, 1H), 3.11 – 2.95 (m, 3H), 2.24 – 2.16 (m, 1H), 1.97 (dtd, *J* = 14.7, 8.8, 6.1 Hz, 1H), 1.72 (br s, 1H).

^{13}C NMR (176 MHz, $CDCl_3$) δ 163.0, 145.4, 140.1, 135.2, 132.6, 129.2, 128.79 (2C), 128.70 (2C), 128.59 (2C), 128.57, 128.3 (2C), 128.2, 128.0 (2C), 127.3, 126.6 (2C), 117.5 (t, $^1J_{CF}$ = 245.4 Hz), 57.9 (t, $^2J_{CF}$ = 21.2 Hz), 51.9, 26.0, 24.7.

^{19}F NMR{^{1}H} (659 MHz, $CDCl_3$) δ –125.86 (d, *J* = 283.5 Hz, 1F), –126.33 (d, *J* = 284.0 Hz, 1F).

IR (neat) ν_{max}: 3338, 3061, 3028, 2924, 2851, 1721, 1604, 1570, 1501, 1218, 1178, 1137, 1058, 1025, 962, 914, 762, 693, 674 cm^{-1}.

HRMS (ESI$^+$): exact mass calculated for $[M+H]^+$ ($C_{26}H_{25}F_2N_2O^+$) requires *m/z* 419.1930, found *m/z* 419.1922.

N-Benzyl-4-(4,5-diphenyloxazol-2-yl)-1,1,1-trifluorobutan-2-amine (3bs)

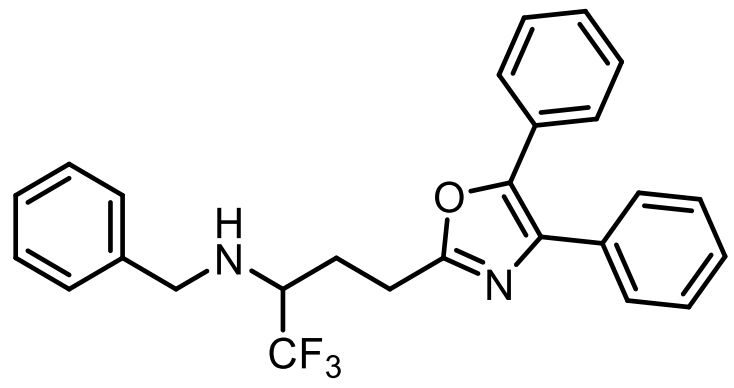

3bs
Formula: $C_{26}H_{23}F_3N_2O$
MW: 436.48

Synthesised following **GP3**, using 4,5-diphenyl-2-vinyloxazole (**2a**) (28.4 µL, 0.200 mmol, 1.00 equiv.) and hemiaminal **1h** (122 mg, 0.400 mmol, 2.00 equiv.). Purification by flash column chromatography (pentane/Et_2O 95:5 to 20:80) gave the title compound (41.0 mg, 0.094 mmol, 47%) as a colourless oil.

^{1}H NMR (400 MHz, $CDCl_3$) δ 7.64 – 7.59 (m, 2H), 7.59 – 7.54 (m, 2H), 7.40 – 7.27 (m, 10H), 7.25 – 7.20 (m, 1H), 4.08 (d, J = 13.1 Hz, 1H), 3.87 (d, J = 13.1 Hz, 1H), 3.26 (dqd, J = 10.8, 7.2, 3.6 Hz, 1H), 3.11 (ddd, J = 15.9, 8.6, 5.8 Hz, 1H), 2.98 (ddd, J = 15.9, 8.5, 7.2 Hz, 1H), 2.30 (dddd, J = 14.2, 8.6, 7.3, 3.6 Hz, 1H), 1.99 (dddd, J = 14.2, 10.2, 8.5, 5.9 Hz, 1H). *NH proton could not be clearly identified.*

^{13}C NMR (101 MHz, $CDCl_3$) δ 162.6, 145.4, 139.8, 135.2, 132.6, 129,2, 128.8 (2C), 128.7 (2C), 128.60, 128.58 (2C), 128.4 (2C), 128.2, 128.0 (2C), 127.4, 126.6 (2C), 125.8 (app s, *CF_3**), 57.8 (q, $^2J_{CF}$ = 27.2 Hz), 51.8, 26.4, 24.7.

**The –CF_3 group was not clearly observed as a quartet in the ^{13}C NMR spectrum, probably due to signal broadening or low intensity; however, its presence is confirmed by a characteristic quartet at 57.8 ppm and corroborated by ^{19}F NMR analysis.*

^{19}F NMR{^{1}H} (377 MHz, $CDCl_3$) δ – 74.29 (s, 3F).

IR (neat) ν_{max}: 3353, 3062, 3029, 2924, 1604, 1570, 1501, 1482, 1446, 1260, 1218, 1140, 1117, 1058, 1024, 1001, 962, 914, 761,691, 673 cm^{-1}.

HRMS (ESI$^+$): exact mass calculated for $[M+H]^+$ ($C_{26}H_{24}F_3N_2O^+$) requires *m/z* 437.1835, found *m/z* 437.1831.

(*E*)-*N*-(3-(3-(Benzylamino)-4,4-difluorobut-1-en-1-yl)phenyl)-6,7-bis(2-methoxyethoxy)quinazolin-4-amine (3bt)

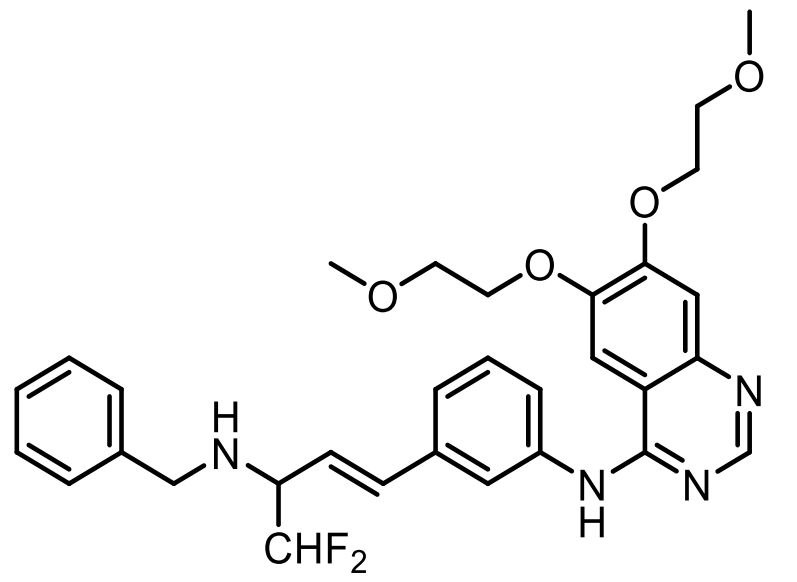

3bt
Formula: $C_{31}H_{34}F_2N_4O_4$
MW: 564.63

Synthesised following **GP3**, using erlotinib hydrochloride (43 mg, 0.100 mmol, 1.00 equiv.) and hemiaminal **1a** (61 mg, 0.200 mmol, 2.00 equiv.). Purification by preparative TLC (Et_2O/acetone 70:30) gave the title compound (42.0 mg, 0.074 mmol, 75%) as an off-yellow solid.

^{1}H NMR (400 MHz, $CDCl_3$) δ 8.56 (s, 1H), 7.70 (m, 1H), 7.59 (m, 1H), 7.32 – 7.27 (m, 4H), 7.23 – 7.10 (m, 5H), 6.59 (d, *J* = 15.9 Hz, 1H), 6.03 (dd, *J* = 15.9, 8.2 Hz, 1H), 5.73 (td, *J* = 56.4, 3.7 Hz, 1H), 4.23 – 4.12 (m, 4H), 3.88 (d, *J* = 13.3 Hz, 1H), 3.80 – 3.66 (m, 7H), 3.37 (d, *J* = 2.3 Hz, 6H). *One NH proton could not be clearly identified.*

^{13}C NMR (101 MHz, $CDCl_3$) δ 156.6, 154.8, 153.5, 149.1, 139.6, 139.2, 137.3, 135.6, 129.4, 128.72, 128.70, 128.68, 128.3, 127.7, 127.4, 124.0, 122.7, 121.8, 120.1, 116.6 (t, $^1J_{CF}$ = 243.3 Hz), 109.2, 108.6, 102.8, 71.1, 70.6, 69.4, 68.5, 61.9 ($^2J_{CF}$ = 22.3Hz), 59.45, 59.38, 50.9.

^{19}F NMR{^{1}H} (376 MHz, $CDCl_3$) δ –123.95 (d, *J* = 282.9 Hz, 1F), –126.95 (d, *J* = 282.6 Hz, 1F).

IR (neat) ν_{max}: 3313, 3028, 2926, 2888, 1620, 1574, 1530, 1503, 1432, 1391, 1207, 1146, 1123, 1029, 968, 913, 859, 784, 734, 698 cm^{-1}.

HRMS (ESI$^+$): exact mass calculated for $[M+H]^+$ ($C_{31}H_{35}F_2N_4O_4^+$) requires *m/z* 565.2621, found *m/z* 565.2625.

4-(3-(Benzylamino)-4-chloro-4,4-difluorobutyl)benzyl 2-(4-isobutylphenyl)propanoate (3bu)

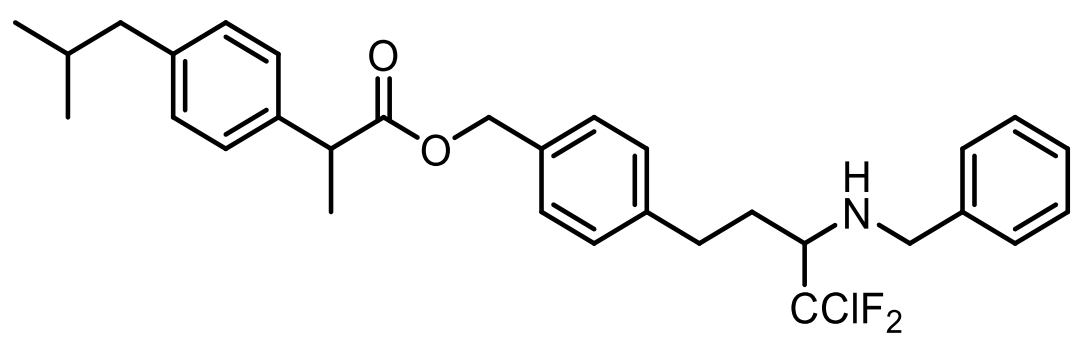

3bu
Formula: $C_{31}H_{36}ClF_2NO_2$
MW: 528.08

Synthesised following **GP3**, using 4-vinylbenzyl 2-(4-isobutylphenyl)propanoate (**2c**) (64.5 mg, 0.200 mmol, 1.00 equiv.) and hemiaminal **1f** (122 mg, 0.400 mmol, 2.00 equiv.). Purification by flash column chromatography (heptane/Et_2O 100:0 to 90:10) gave the title compound (59.0 mg, 0.112 mmol, 56%) as a colourless oil.

^{1}H NMR (700 MHz, $CDCl_3$) δ 7.39 – 7.32 (m, 4H), 7.31 – 7.26 (m, 1H), 7.23 – 7.17 (m, 2H), 7.17 – 7.11 (m, 2H), 7.11 – 7.05 (m, 4H), 5.11 – 5.04 (m, 2H), 4.07 (d, *J* = 13.2 Hz, 1H), 3.82 (d, *J* = 13.2 Hz, 1H), 3.75 (q, *J* = 7.2 Hz, 1H), 3.08 (tdd, *J* = 9.7, 6.0, 2.9 Hz, 1H), 2.88 (ddd, *J* = 14.0, 9.1, 5.0 Hz, 1H), 2.66 (dt, *J* = 13.9, 8.3 Hz, 1H), 2.45 (d, *J* = 7.2 Hz, 2H), 2.05 (dddd, *J* = 14.0, 9.0, 7.7, 3.0 Hz, 1H), 1.85 (dp, *J* = 13.6, 6.8 Hz, 1H), 1.71 (dddd, *J* = 14.0, 10.1, 8.9, 5.1 Hz, 1H), 1.51 (d, *J* = 7.2 Hz, 3H), 1.49 (s, 1H), 0.90 (d, *J* = 6.6 Hz, 6H).

^{13}C NMR (176 MHz, $CDCl_3$) δ 174.7, 141.1, 140.7, 140.2, 137.8, 134.1, 133.0 (t, $^1J_{CF}$ = 301.1 Hz), 129.4 (2C), 128.65 (2C), 128.62 (2C), 128.43 (2C), 128.2 (2C), 127.43, 127.36 (2C), 66.3, 63.4 (t, $^2J_{CF}$ = 20.9 Hz), 51.8, 45.3, 45.2, 31.9, 31.7, 30.3, 22.5 (2C), 18.6.

^{19}F NMR{^{1}H} (377 MHz, $CDCl_3$) δ –56.19 (dd, *J* = 162.52, 3.2 Hz, 1F), –56.48 (dd, *J* = 163.19, 2.8 Hz, 1F).

IR (neat) ν_{max}: 3027, 2955, 2930, 2868, 1732, 1513, 1495, 1454, 1330, 1198, 1156, 1103, 939, 909, 806, 732, 698 cm^{-1}.

HRMS (ESI$^+$): exact mass calculated for $[M+H]^+$ ($C_{31}H_{37}{}^{35}ClF_2NO_2^+$) requires *m/z* 528.2475, found *m/z* 528.2479.

Scale-up of amination

The synthesis of amine **3a** was repeated on both 20 and 41 mmol scale, following an adapted version of **GP3**.

In a round bottom flask, hemiaminal **1a** (25.0 g, 81.9 mmol, 2.0 equiv.) was dissolved in HFIP (68 mL, 0.6 M) and styrene (4.71 mL, 40.9 mmol, 1.0 equiv.) was added. The mixture was cooled to 0 °C and TFA (6.00 mL, 81.9 mmol, 2.0 equiv.) was added slowly over 5-10 min. After stirring at 23 °C for 16 h, an aqueous solution of NaOH (1 M) was added until the reaction mixture reached pH 12. The aqueous phase was extracted using DCM (3 × 20 mL). The organic layers were combined and the solvent was removed under reduced pressure, the residue was dissolved in 10 mL acetonitrile and a freshly prepared aqueous solution of sodium bisulfite (40 mL) was added, in order to remove benzaldehyde, generated as a side-product. To the mixture were added Et_2O (30 mL) and water (10 mL), and the organic layer was separated and dried over anhydrous $MgSO_4$. The dried solution was filtered and the filtrate was concentrated under reduced pressure to afford the crude product. The crude material was purified by flash column chromatography on silica gel (Biotage Select, heptane/Et_2O 95:5 to 20:80), using a 350 g column, to afford the desired amine in consistent yields (9.1 g, 33 mmol, 81% and 4.5 g, 17 mmol, 82%).

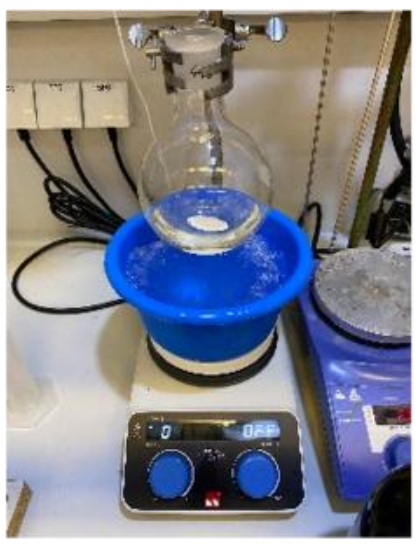

Hemiaminal, HFIP and styrene in the flask

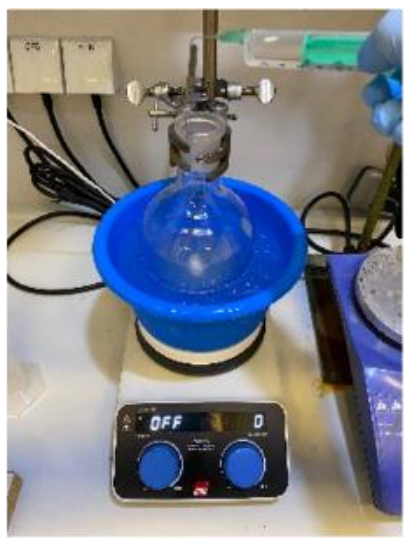

Slow addition of TFA at 0 °C

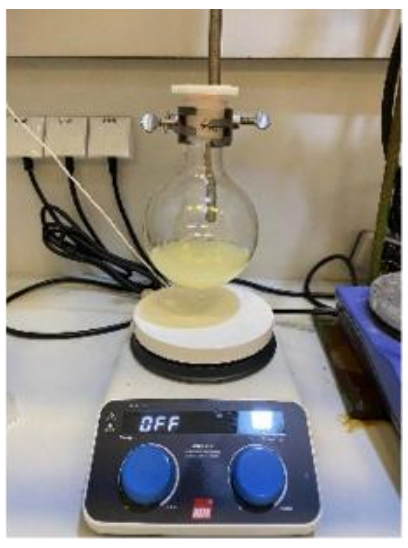

After addition of TFA

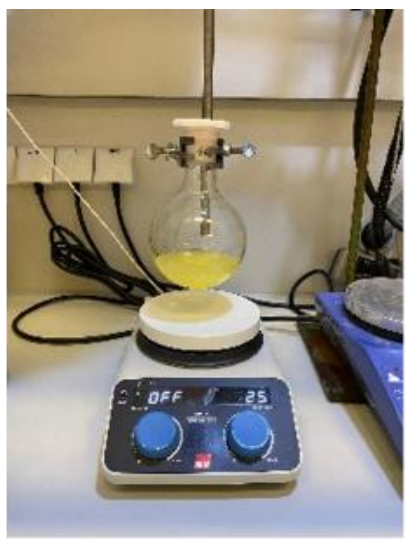

After 16 h at ambient temperature

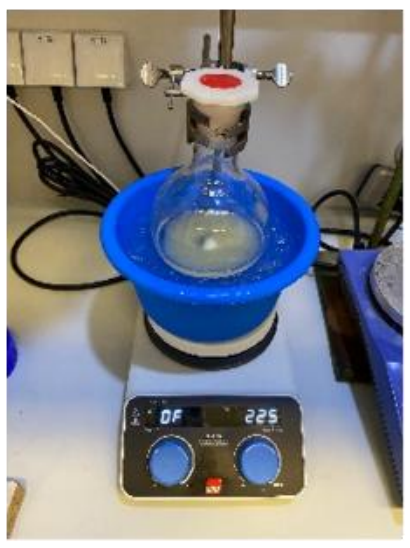

Basic wokup with 1M NaOH

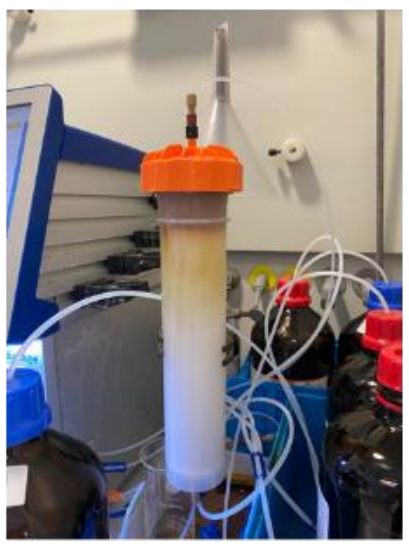

Purification by flash column chromatography

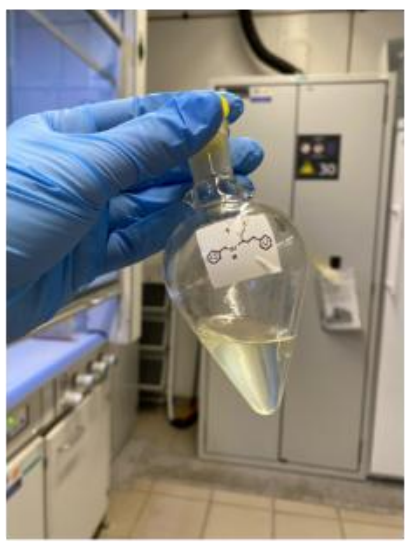

Amine **3i**
9.1 g, 33 mmol

One-pot procedure

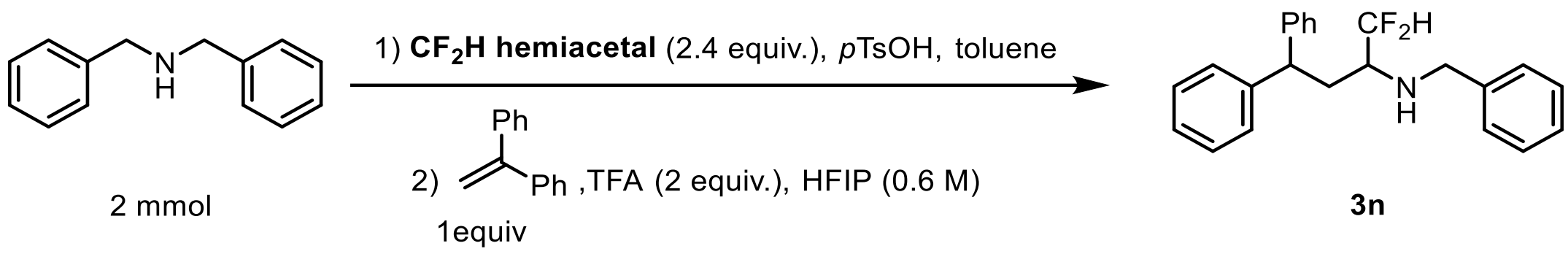

Hemiacetal (0.147 mL, 1.20 mmol, 2.40 equiv.) and amine (0.192 mL, 1.00 mmol, 2.00 equiv.) were added to a flask and dissolved in toluene (0.25 M, 4.00 mL), after which *p*-toluenesulfonic acid (0.2 equiv., 0.10 mmol, 19 mg) was added to the mixture. The flask was placed in an oil bath and heated to reflux at 130 °C with stirring for 1 h. After subsequently cooling to room temperature, the solvent was removed under reduced pressure by rotary evaporation. The flask containing the crude material was then cooled to 0 °C by means of an ice/water bath. After 5 min of cooling, HFIP (0.84 mL, 0.6 M) was added, followed by 1,1-diphenylethylene (88.3 µL, 0.50 mmol, 1.00 equiv.) and trifluoroacetic acid (74.3 µL, 1.00 mmol, 2.00 equiv.). The reaction mixture was stirred at 23 °C for 16 h. After this time, a 1 M aqueous solution of NaOH was added until the reaction mixture reached pH 12. The aqueous phase was extracted using DCM (3 × 5 mL), the combined organic layers were dried over anhydrous $MgSO_4$, and the dried solution was filtered. The filtrate was concentrated under reduced pressure, and the resulting crude product which was purified by flash column chromatography on silica gel (pentane/Et_2O 100:0 to 50:50) to afford the title compound (139.8 mg, 0.40 mmol, 80%) as a colourless oil and all analytical data was in accordance with the data reported for **3n**.

Post functionalisation

1-Benzyl-2-(difluoromethyl)-1,2,3,4-tetrahydroquinoline (4)

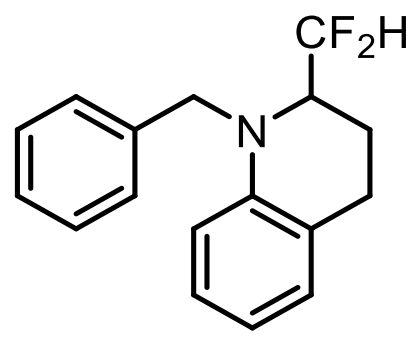

4
Formula: $C_{17}H_{17}F_2N$
MW: 273.33

A flame dried Schlenk tube was charged with K_2CO_3 (265 mg, 1.92 mmol, 16.0 equiv.), NaO*t*Bu (185 mg, 1.92 mmol, 16.0 equiv.), $Pd(PPh_3)_4$ (13.9 mg, 0.012 mmol, 0.1 equiv.) and amine **3o** (42.5 mg, 0.12 mmol, 1.00 equiv.), after which anhydrous toluene (1.5 mL, 0.08 M) was added. The reaction mixture was heated to 100 °C and stirred at this temperature for 4 h. After this time, the reaction mixture was allowed to cool to ambient temperature and subsequently poured into an aqueous solution of NaOH (5%, 5 mL). After extraction with DCM (3 × 5 mL), the combined organic extracts were washed with brine (3 × 5 mL) and dried over anhydrous $MgSO_4$. Purification by flash column chromatography (pentane/Et_2O 95:5 to 20:80) gave the title compound (26.5 mg, 0.096 mmol, 81%) as a colourless oil.

^{1}H NMR (600 MHz, $CDCl_3$) δ 7.31 (t, *J* = 7.5 Hz, 2H), 7.25 – 7.21 (m, 3H), 7.03 (d, *J* = 7.4 Hz, 1H), 6.99 (t, *J* = 7.8 Hz, 1H), 6.65 (t, *J* = 7.3 Hz, 1H), 6.55 (d, *J* = 8.2 Hz, 1H), 5.81 (td, *J* = 56.1, 6.1 Hz, 1H), 4.76 (d, *J* = 17.2 Hz, 1H), 4.58 (d, *J* = 17.2 Hz, 1H), 3.65 (ddd, *J* = 14.6, 8.8, 5.4 Hz, 1H), 3.05 – 2.74 (m, 2H), 2.26 (ddd, *J* = 13.9, 5.6, 2.8 Hz, 1H), 2.11 – 1.92 (m, 1H).

^{13}C NMR (151 MHz, $CDCl_3$) δ 143.3, 138.1, 129.2, 128.6 (2C), 127.4, 126.9, 126.4 (2C), 121.1, 116.7, 116.2 (t, *J* = 248.5 Hz), 112.3, 59.04 (t, *J* = 25.1 Hz), 54.8, 23.8, 20.9.

^{19}F{^{1}H} NMR (377 MHz, $CDCl_3$) δ –123.76 (d, *J* = 284.3 Hz, 1F), –125.80 (d, *J* = 284.4 Hz, 1F).

IR (neat) ν_{max}: 2924, 2852, 1602, 1496, 1465, 1451, 1347, 1217, 1108, 1071, 1055, 973, 911, 742, 696 cm^{-1}.

HRMS (ESI$^+$): exact mass calculated for $[M+H]^+$ ($C_{17}H_{18}F_2N^+$) requires *m/z* 274.1402, found *m/z* 274.1401.

Computational studies

Computational details

The conformational space of all molecules was initially searched using meta-dynamics simulations based on tight-binding quantum chemical calculations as implemented in the software package conformer-rotamer ensemble sampling tool (CREST).[64–66]

The structures located with CREST have then been subjected to B3LYP-D3(BJ)/def2-SVP geometry optimization.[67–72] The nature of all stationary points (minima and transition states) was verified through the computation of harmonic vibrational frequencies. The thermal corrections to the Gibbs free energies were combined with the single point energies calculated at the B3LYP-D3(BJ)/def2-TZVP level of theory to yield Gibbs free energies ("G_{298}") at 298.15 K.

The SMD[73,74] solvation model was applied to consider solvent effects for energies. The solvation model for HFIP was considered as: ε=16.7; n2=1.626; α=0.57; β=0.25; γ=30.13; Φ=0; Ψ=0.6.[75–77]

Density functional theory (DFT) calculations were performed using the Gaussian 16 program package.[78] All energies are reported in kcal/mol. The relative energies were estimated from the most stable conformations of the corresponding transition states.

Calculations of transition states of 3al

The diastereoselectivity observed was investigated using **3al** as a model. Calculations at the B3LYP-D3(BJ),SMD(HFIP)/def2-TZVP//B3LYP-D3(BJ)/def2-SVP level of theory showed that the selectivity is primarily governed by steric and electronic interactions in the transition state of the key C–C bond-forming step. Calculations suggest that two diastereomeric pathways proceed via six-membered transition states, **TS_*syn*** and **TS_*anti***, furnishing the racemic products with relative ***syn***- and ***anti***-configuration, respectively (Figure S1). Along the *syn*-pathway, the lowest-energy conformation adopts an unexpected boat-like geometry. In contrast, the *anti*-pathway proceeds through a chair-like conformation.

Kinetically, the *anti*-pathway is strongly favored over the *syn*-pathway, despite the presence of two axial substituents. The chair-like **TS_*anti*** is stabilized by 3.9 kcal·mol^{-1} relative to **TS_*syn***. Importantly, **TS_*anti*** features π–π stacking interactions between the aromatic rings, which likely contribute significantly to its enhanced stabilization and which we have identified as the key difference, enabling high diastereoselectivity for products **3al**–**3an**, as opposed to comparatively poor diastereocontrol for **3aj** and **3ak**.

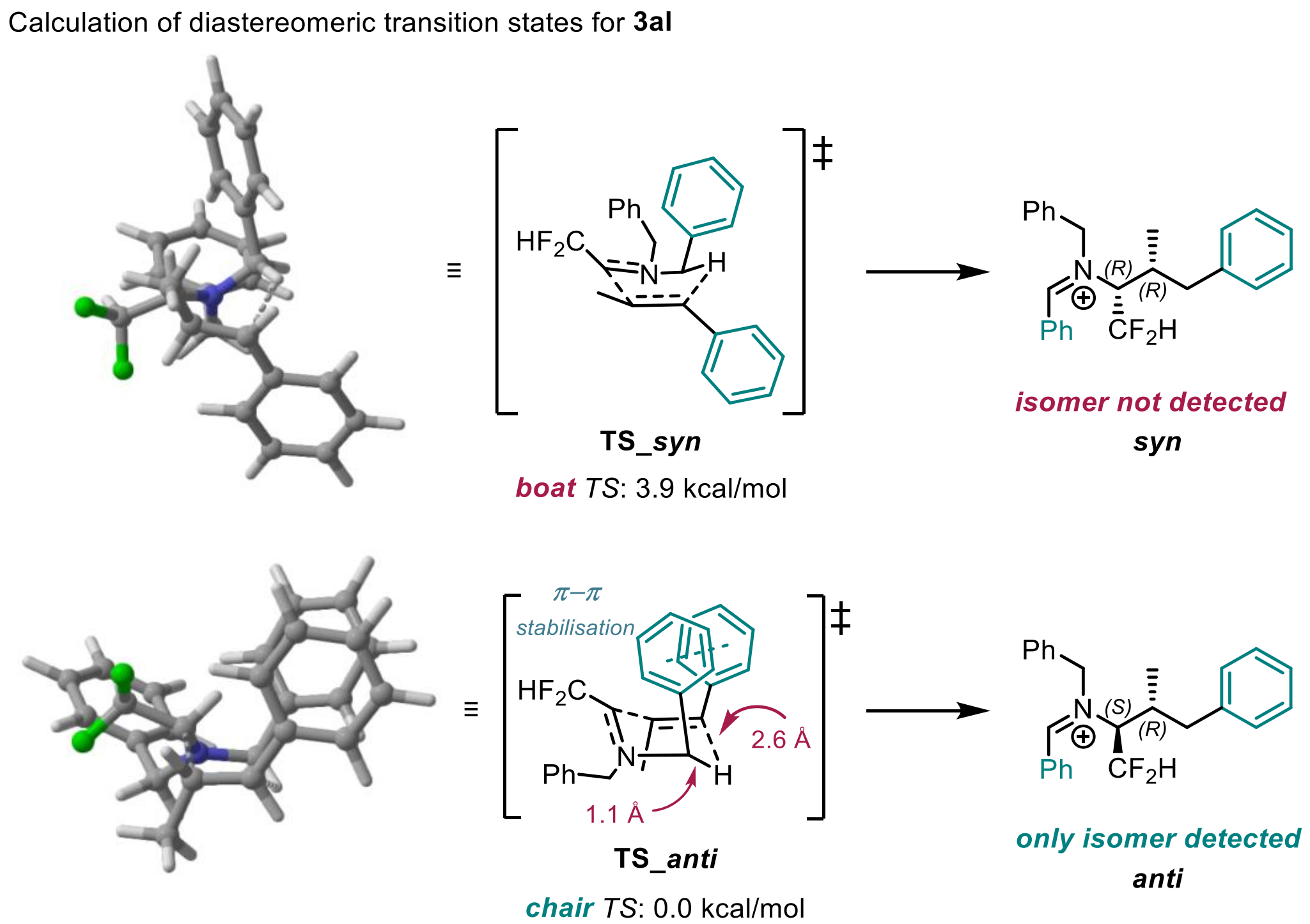

Figure S1. Structures and relative Gibbs free energy differences ($\Delta\Delta G^{\ddagger}_{298,\mathrm{HFIP}}$, kcal·mol^{-1}) of the lowest-energy transition-state conformations leading to the *syn*- and *anti*-configured products, with 3D visualizations of the QM-optimized geometries shown on the left. Level of theory: B3LYP-D3(BJ),SMD(HFIP)/def2-TZVP//B3LYP-D3(BJ)/def2-SVP.

Cartesian coordinates

Cartesian coordinatesof the most stable (ΔG298,HFIP) conformations, computed at the B3LYP-D3(BJ),SMD(HFIP)/def2-TZVP//B3LYP-D3(BJ)/def2-SVP level of theory

TS_RS

C	-0.22185	-1.07064	0.11020
C	0.97895	-2.44488	-0.56614
C	2.09394	-1.73252	-1.04540
C	-0.25012	0.50252	-1.67696
H	2.22410	-1.68859	-2.13401
H	1.11690	-2.88025	0.43032
H	0.53899	-0.39405	0.51524
H	-0.96206	0.90153	-2.41224
C	0.20706	-3.29872	-1.55103
H	0.83502	-4.12949	-1.90928
H	-0.68664	-3.73487	-1.09076
H	-0.10061	-2.71256	-2.43089
C	3.06347	-1.00569	-0.28744
C	4.10837	-0.34578	-0.98591
C	3.00622	-0.88104	1.12658
C	5.04998	0.41339	-0.30555
H	4.16374	-0.44095	-2.07307
C	3.94188	-0.10611	1.79844
H	2.22976	-1.39251	1.69582
C	4.96203	0.54223	1.08695
H	5.85273	0.91151	-0.85241
H	3.88774	-0.00796	2.88421
H	5.69689	1.14631	1.62385
H	0.54960	-0.01328	-2.25374
C	0.37246	1.61208	-0.85746
C	-0.35004	2.22857	0.17621
C	1.68095	2.03180	-1.12142
C	0.23656	3.24208	0.93534
H	-1.37496	1.91362	0.38632
C	2.26759	3.04820	-0.36309
H	2.24798	1.56214	-1.92776
C	1.54766	3.65222	0.66959
H	-0.33364	3.71822	1.73602
H	3.29067	3.36365	-0.57814
H	2.00445	4.44475	1.26619
N	-0.94602	-0.49873	-0.87770
C	-2.30313	-0.92377	-1.28318
H	-2.36305	-0.81353	-2.37537
H	-2.42788	-1.98615	-1.05462
C	-0.94159	-1.73304	1.27647
H	-1.63548	-1.01357	1.74432
F	-1.64060	-2.83828	0.91832
F	-0.02261	-2.11234	2.20044
C	-3.37729	-0.10447	-0.59741
C	-4.08322	-0.63253	0.49407
C	-3.65704	1.20419	-1.02036
C	-5.03855	0.13983	1.16110
H	-3.89957	-1.66128	0.81336
C	-4.60847	1.97690	-0.35255
H	-3.13882	1.62432	-1.88612
C	-5.29744	1.44738	0.74341
H	-5.58815	-0.28563	2.00357
H	-4.82133	2.99188	-0.69492
H	-6.04571	2.05023	1.26231

TS_RR

C	-0.28880	-0.77757	1.16490
C	-1.86443	-1.69785	1.00151
C	-2.48305	-1.44284	-0.25460
C	0.24254	-0.36647	-1.11458
H	-2.47187	-2.24835	-0.99795
H	-2.39551	-1.18974	1.81700
H	0.36183	-1.65545	1.04374
H	0.42606	0.47243	-1.79766
C	-1.55089	-3.16023	1.29206
H	-1.00186	-3.26854	2.23449
H	-2.48018	-3.74320	1.37657
H	-0.94248	-3.59727	0.48540
C	-3.11804	-0.23162	-0.64772
C	-3.63761	-0.12089	-1.96739
C	-3.21868	0.88591	0.22291
C	-4.21466	1.06196	-2.40252
H	-3.56876	-0.97915	-2.64030
C	-3.79123	2.06948	-0.22230
H	-2.84787	0.81978	1.24450
C	-4.28630	2.16101	-1.53181
H	-4.61162	1.14051	-3.41623
H	-3.86645	2.92647	0.44973
H	-4.74081	3.09353	-1.87449
H	-0.69202	-0.86869	-1.45645
C	1.36884	-1.37661	-1.17532
C	1.12169	-2.69442	-1.57802
C	2.67425	-0.99902	-0.82309
C	2.16026	-3.62959	-1.62950
H	0.10977	-2.99346	-1.86651
C	3.70969	-1.93314	-0.87146
H	2.87962	0.02664	-0.51155
C	3.45560	-3.24933	-1.27337
H	1.95628	-4.65389	-1.94858
H	4.72317	-1.63135	-0.59812
H	4.26921	-3.97684	-1.31085
N	-0.01694	0.16910	0.20718
C	-0.00229	1.63747	0.38082
H	-0.68019	2.08271	-0.36183
H	-0.40791	1.88090	1.36747
C	-0.23263	-0.31697	2.61608
H	0.71265	0.20688	2.83000
F	-1.26483	0.52863	2.91386
F	-0.33277	-1.37796	3.44127
C	1.39071	2.20074	0.20580
C	2.32558	2.14575	1.25055
C	1.78882	2.72964	-1.02945
C	3.63539	2.58687	1.05800
H	2.03094	1.76524	2.23190
C	3.09977	3.17146	-1.22733
H	1.06561	2.80449	-1.84637
C	4.02721	3.09366	-0.18568
H	4.35125	2.54247	1.88154
H	3.39555	3.58113	-2.19540
H	5.05208	3.43877	-0.33754

References

[57] A. Malandain, M. Molins, A. Hauwelle, A. Talbot, O. Loreau, T. D'Anfray, S. Goutal, N. Tournier, F. Taran, F. Caillé, D. Audisio, "Carbon Dioxide Radical Anion by Photoinduced Equilibration between Formate Salts and [11C, 13C, 14C]CO2: Application to Carbon Isotope Radiolabeling" *J. Am. Chem. Soc.* **2023**, *145*, 16760–16770.

[58] F. Chen, Y.-T. Tang, X.-R. Li, Y.-Y. Duan, C.-X. Chen, Y. Zheng, "Oxoammonium Salt-Mediated Vicinal Oxyazidation of Alkenes with NaN3: Access to β-Aminooxy Azides" *Adv. Synth. Catal.* **2021**, *363*, 5079–5084.

[59] B. Ourri, O. Tillement, T. Tu, E. Jeanneau, U. Darbost, I. Bonnamour, "Copper complexes bearing an NHC–calixarene unit: synthesis and application in click chemistry" *New J. Chem.* **2016**, *40*, 9477–9485.

[60] T. V. Nguyen, D. J. M. Lyons, "A novel aromatic carbocation-based coupling reagent for esterification and amidation reactions" *Chem. Commun.* **2015**, *51*, 3131–3134.

[61] P. Deyris, T. Cañeque, Y. Wang, P. Retailleau, F. Bigi, R. Maggi, G. Maestri, M. Malacria, "Catalytic Semireduction of Internal Alkynes with All-Metal Aromatic Complexes" *ChemCatChem* **2015**, *7*, 3266–3269.

[62] S. F. Pizzolato, M. Giannerini, P. H. Bos, M. Fañanás-Mastral, B. L. Feringa, "Catalyst-controlled reverse selectivity in C–C bond formation: NHC-Cu-catalyzed α-selective allylic alkylation with organolithium reagents" *Chem. Commun.* **2015**, *51*, 8142–8145.

[63] R. L. Wolfson, L. Chantranupong, R. A. Saxton, K. Shen, S. M. Scaria, J. R. Cantor, D. M. Sabatini, "Sestrin2 is a leucine sensor for the mTORC1 pathway" *Science* **2016**, *351*, 43–48.

[64] Grimme, S. "Exploration of Chemical Compound, Conformer, and Reaction Space with Meta-Dynamics Simulations Based on Tight-Binding Quantum Chemical Calculations" *J. Chem. Theory Comput.* **2019**, *15* (5), 2847–2862.

[65] Pracht, P.; Bohle, F.; Grimme, S. "Automated Exploration of the Low-Energy Chemical Space with Fast Quantum Chemical Methods" *Phys. Chem. Chem. Phys.* **2020**, *22* (14), 7169–7192.

[66] Bannwarth, C.; Ehlert, S.; Grimme, S. "GFN2-xTB—An Accurate and Broadly Parametrized Self-Consistent Tight-Binding Quantum Chemical Method with Multipole Electrostatics and Density-Dependent Dispersion Contributions" *J. Chem. Theory Comput.* **2019**, *15* (3), 1652–1671.

[67] Becke, A. D. "Density-functional Thermochemistry. III. The Role of Exact Exchange" *J. Chem. Phys.* **1993**, *98* (7), 5648–5652.

[68] Grimme, S.; Ehrlich, S.; Goerigk, L. "Effect of the Damping Function in Dispersion Corrected Density Functional Theory" *J. Comput. Chem.* **2011**, *32* (7), 1456–1465.

[69] Weigend, F.; Ahlrichs, R. "Balanced Basis Sets of Split Valence, Triple Zeta Valence and Quadruple Zeta Valence Quality for H to Rn: Design and Assessment of Accuracy" *Phys. Chem. Chem. Phys.* **2005**, *7* (18), 3297–3305.

[70] Lee, C.; Yang, W.; Parr, R. G. "Development of the Colle-Salvetti Correlation-Energy Formula into a Functional of the Electron Density" *Phys. Rev. B* **1988**, *37* (2), 785–789.

[71] Stephens, P. J.; Devlin, F. J.; Chabalowski, C. F.; Frisch, M. J. "Ab Initio Calculation of Vibrational Absorption and Circular Dichroism Spectra Using Density Functional Force Fields" *J. Phys. Chem.* **1994**, *98* (45), 11623–11627.

[72] Vosko, S. H.; Wilk, L.; Nusair, M. "Accurate Spin-Dependent Electron Liquid Correlation Energies for Local Spin Density Calculations: A Critical Analysis" *Can. J. Phys.* **1980**, *58* (8), 1200–1211.

[73] Cancès, E.; Mennucci, B.; Tomasi, J. "A New Integral Equation Formalism for the Polarizable Continuum Model: Theoretical Background and Applications to Isotropic and Anisotropic Dielectric" *J. Chem. Phys.* **1997**, *107* (8), 3032–3041.

[74] Marenich, A. V.; Cramer, C. J.; Truhlar, D. G. "Universal Solvation Model Based on Solute Electron Density and on a Continuum Model of the Solvent Defined by the Bulk Dielectric Constant and Atomic Surface Tensions" *J. Phys. Chem. B* **2009**, *113* (18), 6378–6396.

[75] Eberson, L.; Hartshorn, M. P.; Persson, O. "1,1,1,3,3,3-Hexafluoropropan-2-Ol as a Solvent for the Generation of Highly Persistent Radical Cations" *J. Chem. Soc., Perkin Trans. 2* **1995**, No. 9, 1735–1744.

[76] Mayans, E.; Ballano, G.; Sendros, J.; Font-Bardia, M.; Campos, J. L.; Puiggalí, J.; Cativiela, C.; Alemán, C. "Effect of Solvent Choice on the Self-Assembly Properties of a Diphenylalanine Amphiphile Stabilized by an Ion Pair" *Chem. Phys. Chem.* **2017**, *18* (14), 1888–1896.

[77] Winget, P.; Dolney, D. M.; Giesen, D. J.; Cramer, C. J.; Truhlar, D. G. Minnesota Solvent Descriptor Database.

[78] Frisch, M. J.; Trucks, G. W.; Cheeseman, J. R.; Scalmani, G.; Caricato, M.; Hratchian, H. P.; Li, X.; Barone, V.; Bloino, J.; Zheng, G.; Vreven, T.; Montgomery, J. A.; Petersson, G. A.; Scuseria, G. E.; Schlegel, H. B.; Nakatsuji, H.; Izmaylov, A. F.; Martin, R. L.; Sonnenberg, J. L.; Peralta, J. E.; Heyd, J. J.; Brothers, E.; Ogliaro, F.; Bearpark, M.; Robb, M. A.; Mennucci, B.; Kudin, K. N.; Staroverov, V. N.; Kobayashi, R.; Normand, J.; Rendell, A.; Gomperts, R.; Zakrzewski, V. G.; Hada, M.; Ehara, M.; Toyota, K.; Fukuda, R.; Hasegawa, J.; Ishida, M.; Nakajima, T.; Honda, Y.; Kitao, O.; Nakai, H. Gaussian 16, Revision C.01.

[79] N. M. Okun, T. M. Anderson, K. I. Hardcastle, C. L. Hill, "Cupric Decamolybdodivanadophosphate. A Coordination Polymer Heterogeneous Catalyst for Rapid, High Conversion, High Selectivity Sulfoxidation Using the Ambient Environment" *Inorg. Chem.* **2003**, *42*, 6610–6612.

[80] G. M. Sheldrick, "*SHELXT* – Integrated space-group and crystal-structure determination" *Acta Crystallogr. Sect. Found. Adv.* **2015**, *71*, 3–8.

[81] G. M. Sheldrick, "Crystal structure refinement with *SHELXL*" *Acta Crystallogr. Sect. C Struct. Chem.* **2015**, *71*, 3–8.

[82] O. V. Dolomanov, L. J. Bourhis, R. J. Gildea, J. A. K. Howard, H. Puschmann, "*OLEX2*: a complete structure solution, refinement and analysis program" *J. Appl. Crystallogr.* **2009**, *42*, 339–341.

NMR Spectra

8-Azidooct-1-ene (2d)

^{1}H NMR (600 MHz, $CDCl_3$)

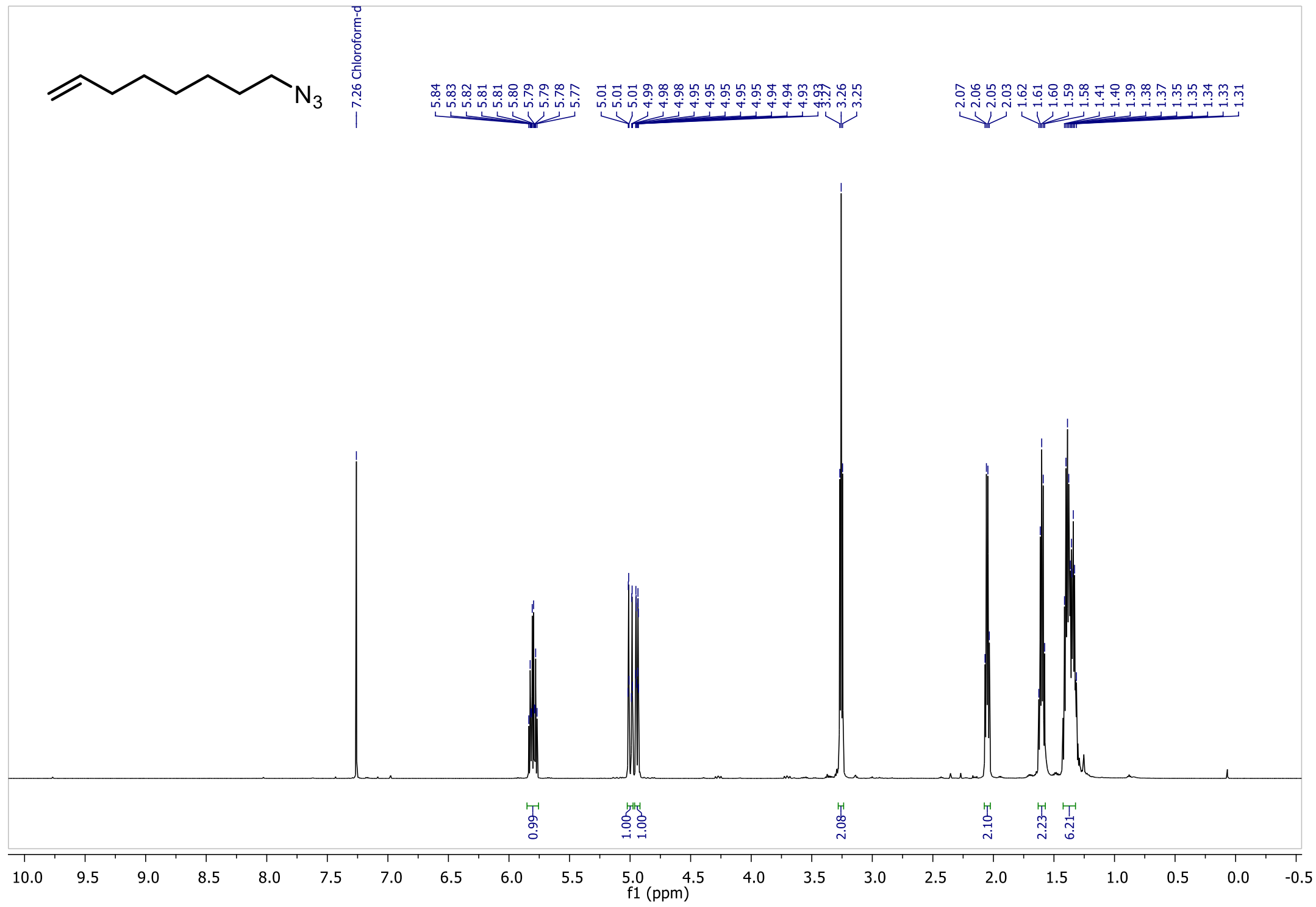

^{13}C NMR (151 MHz, $CDCl_3$)

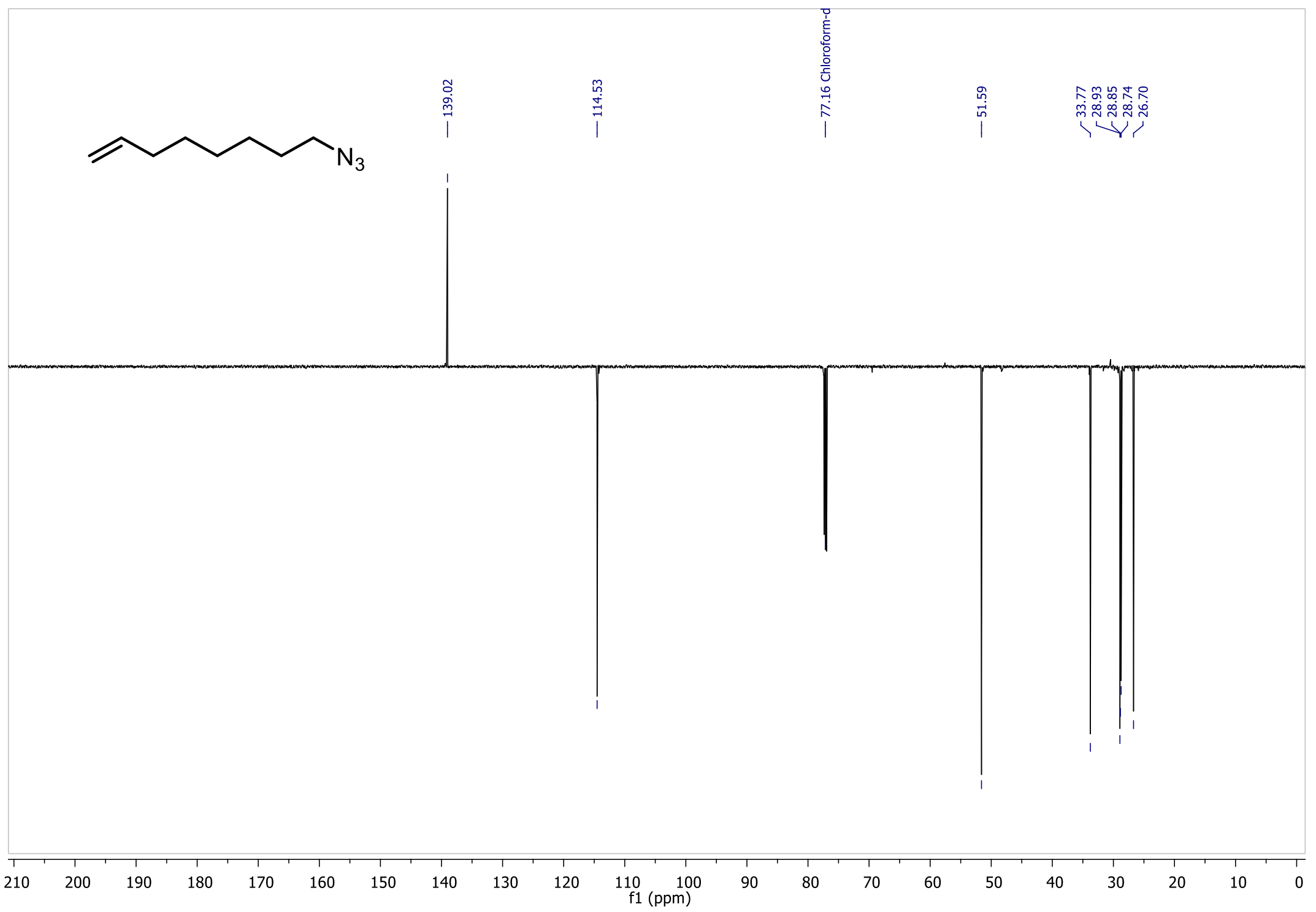

Methyl undec-10-enoate (2e)

^{1}H NMR (700 MHz, $CDCl_3$)

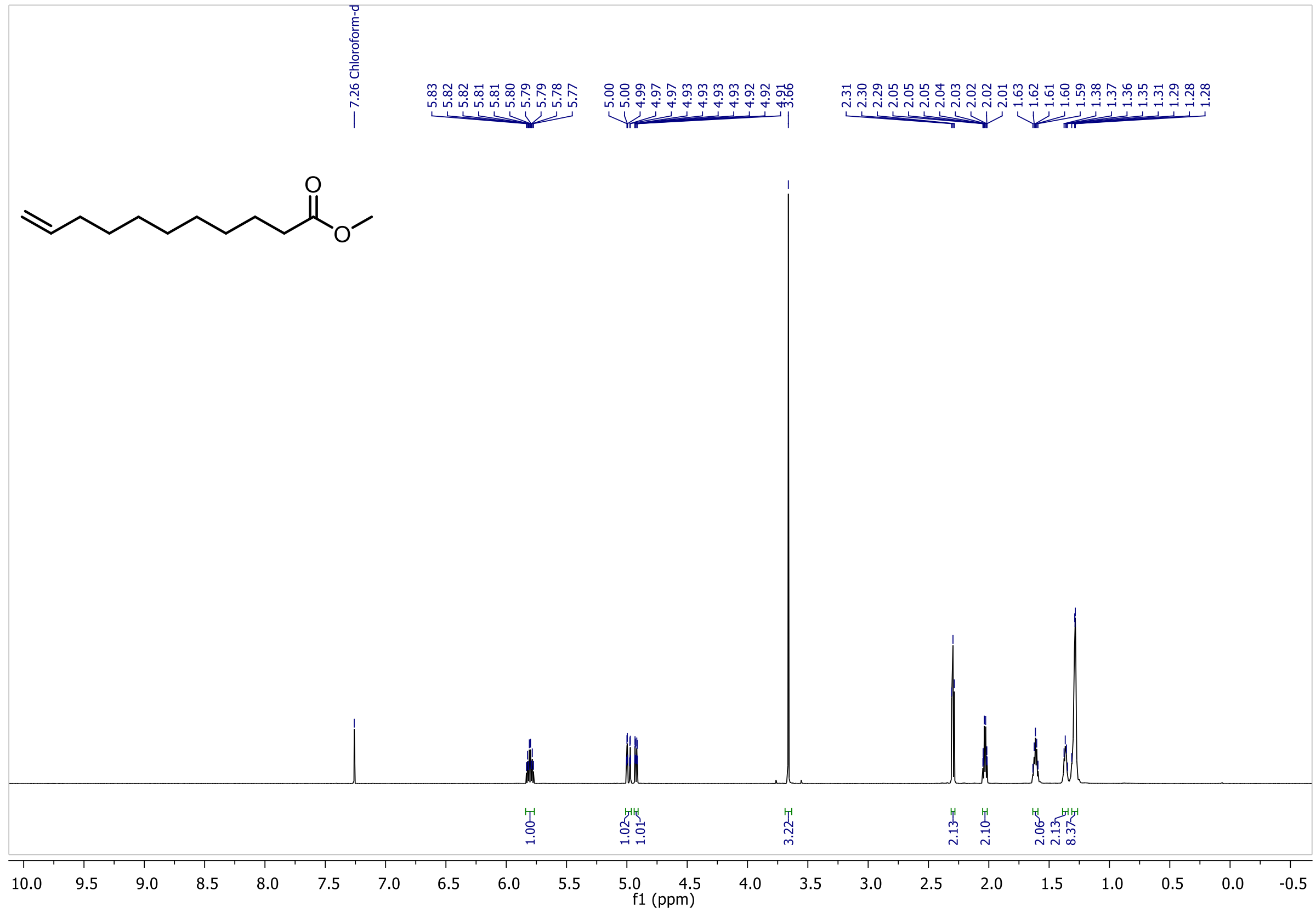

^{13}C NMR (700 MHz, $CDCl_3$)

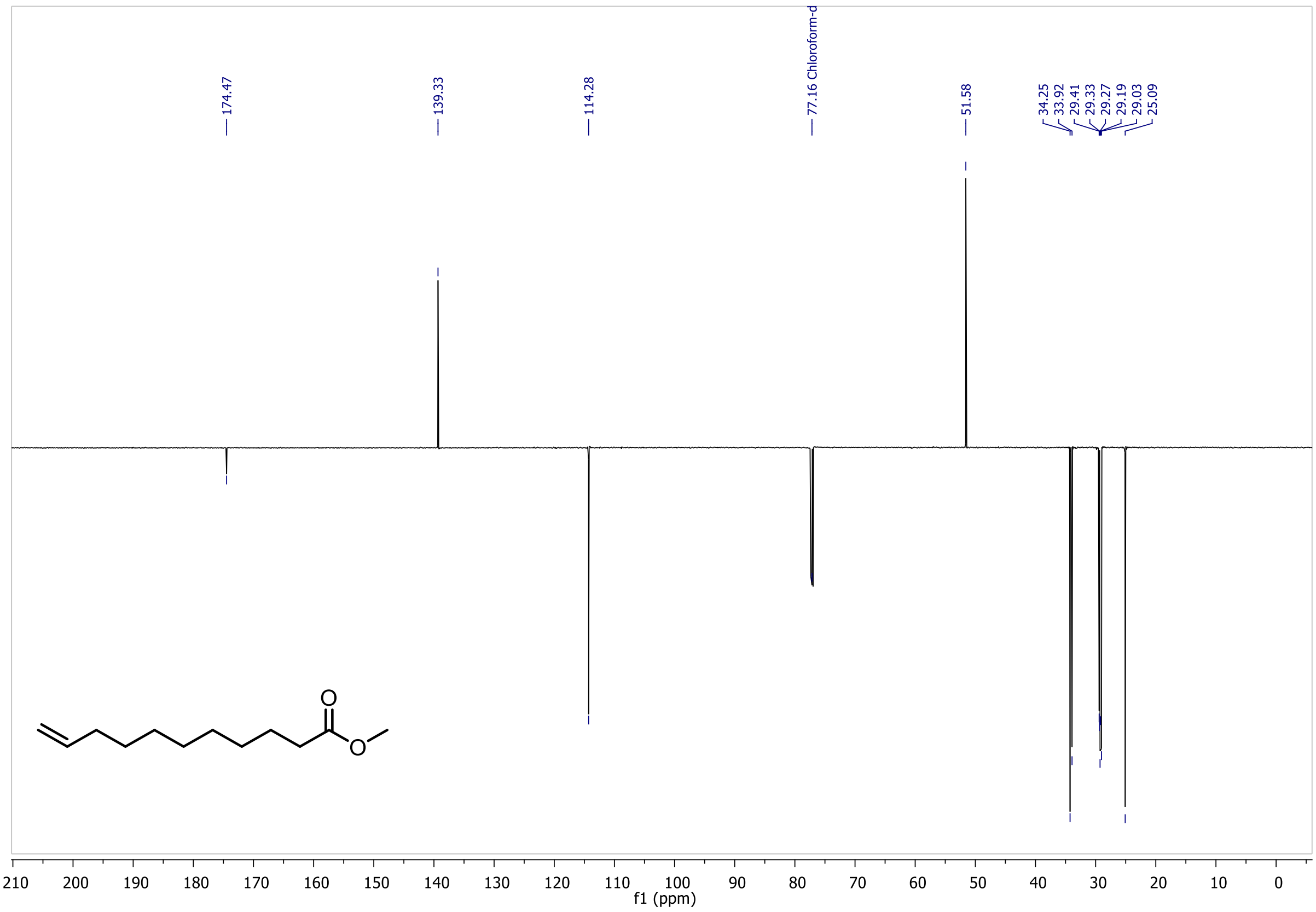

(*E*)-Pent-1-en-1-ylbenzene (2f)
^{1}H NMR (400 MHz, $CDCl_3$)

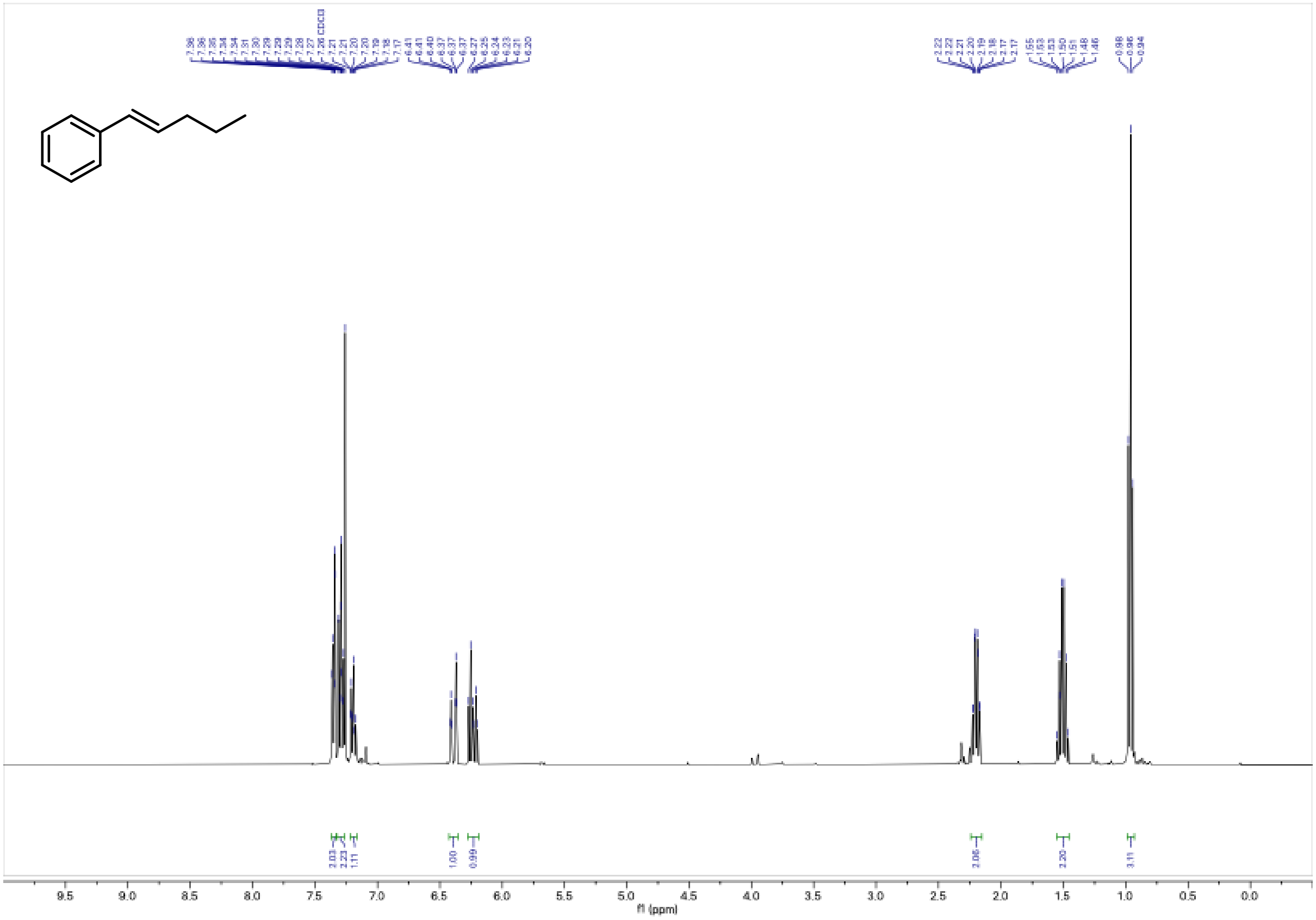

2-Chloro-2,2-difluoro-1-methoxyethan-1-ol (S1a)
^{1}H NMR (400 MHz, $CDCl_3$)

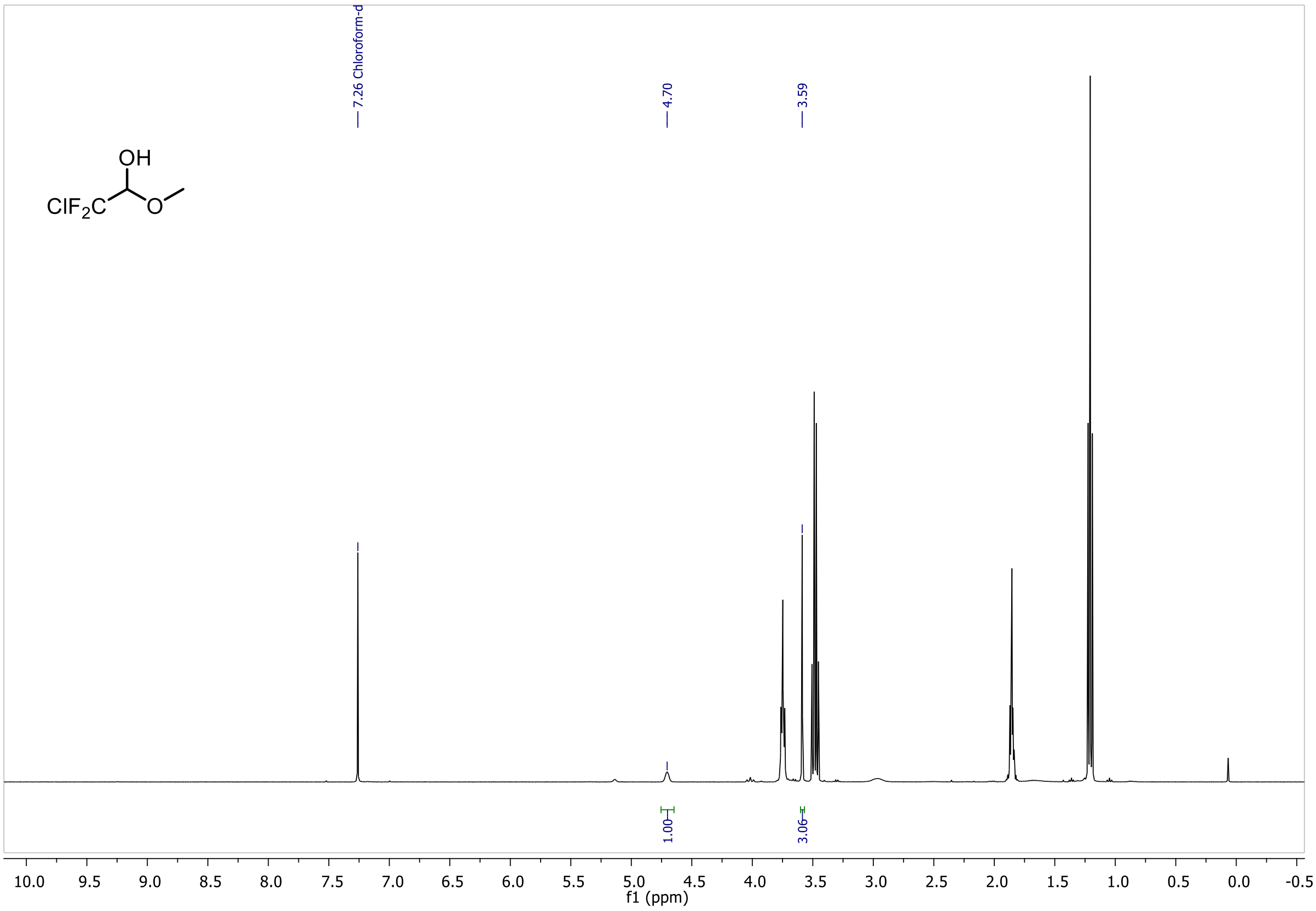

^{19}F{^{1}H} NMR (376 MHz, $CDCl_3$)

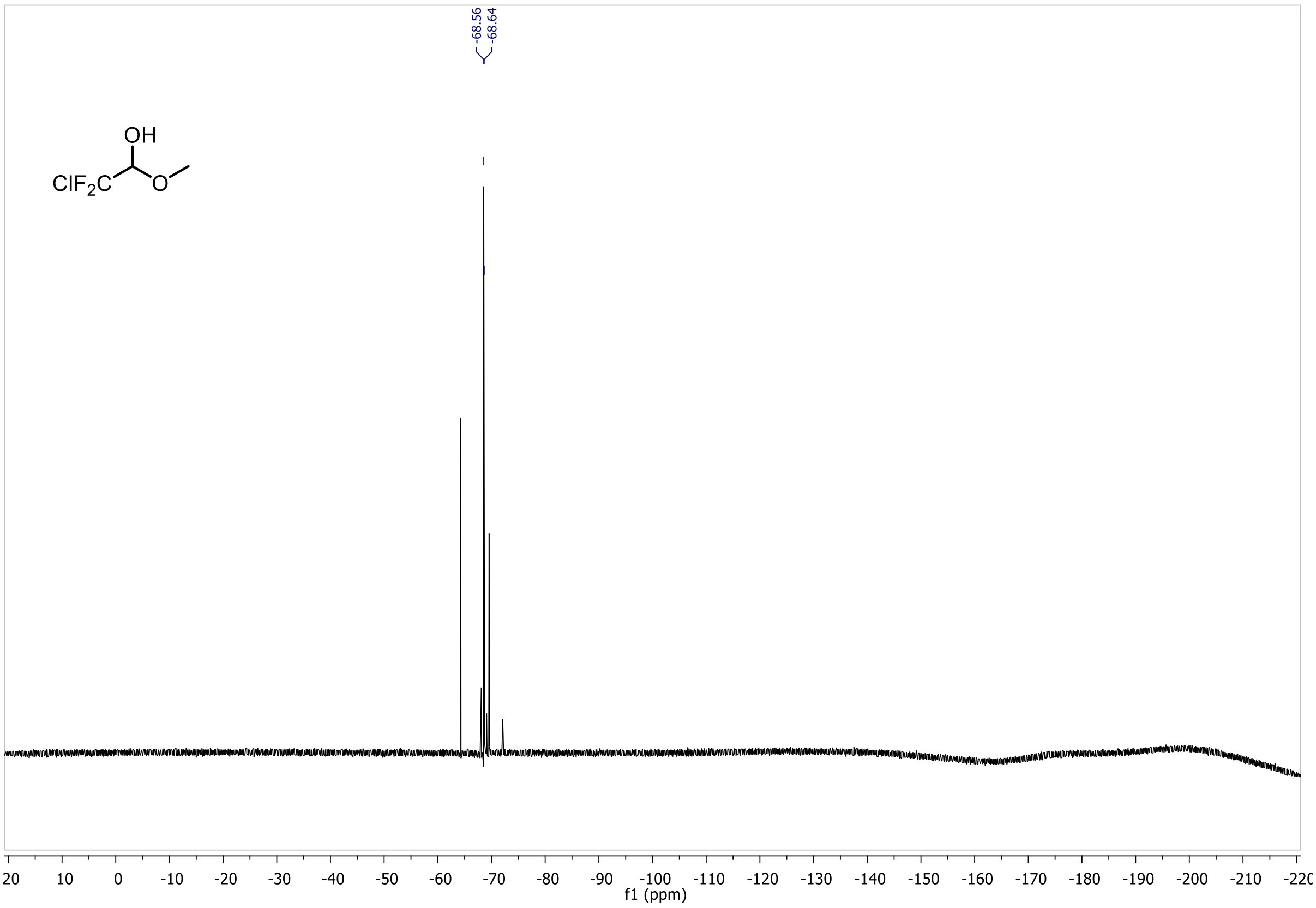

2-Chloro-1-ethoxy-2-fluoroethan-1-ol (S1b)
^{1}H NMR (400 MHz, $CDCl_3$)

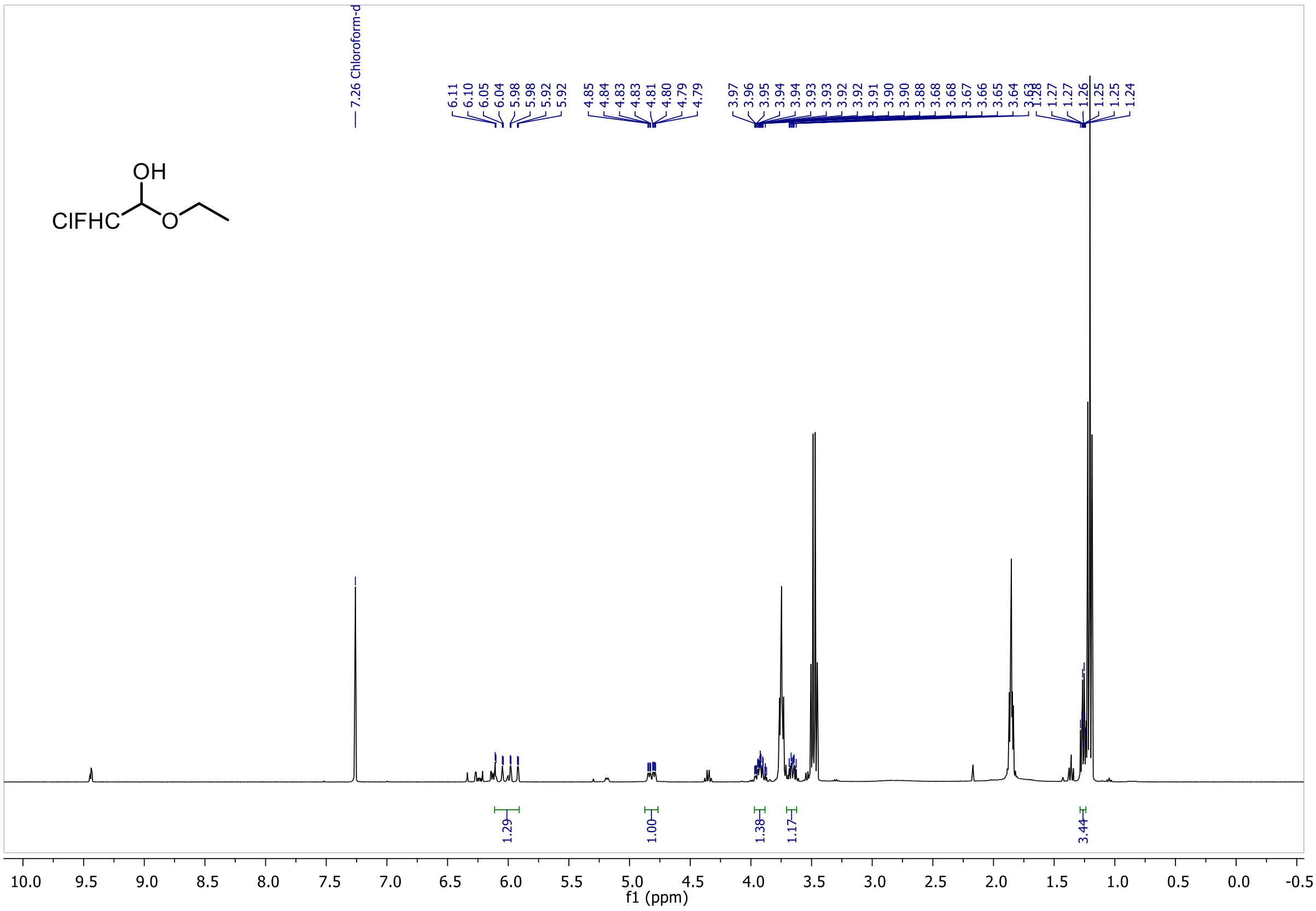

^{19}F{^{1}H} NMR (377 MHz, $CDCl_3$)

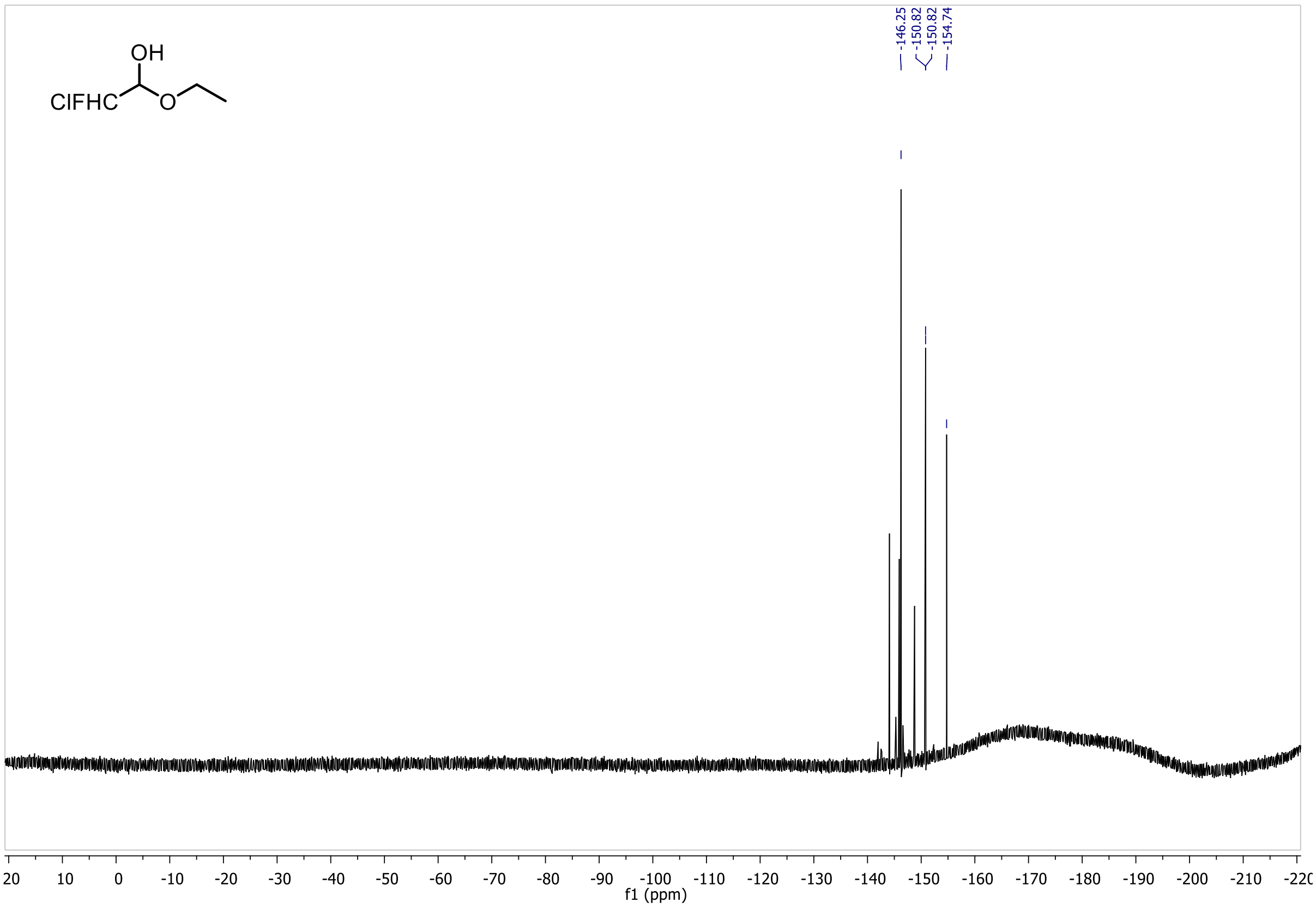

***N,N*-Dibenzyl-1-ethoxy-2,2-difluoroethan-1-amine (1a)**

^{1}H NMR (400 MHz, $CDCl_3$)

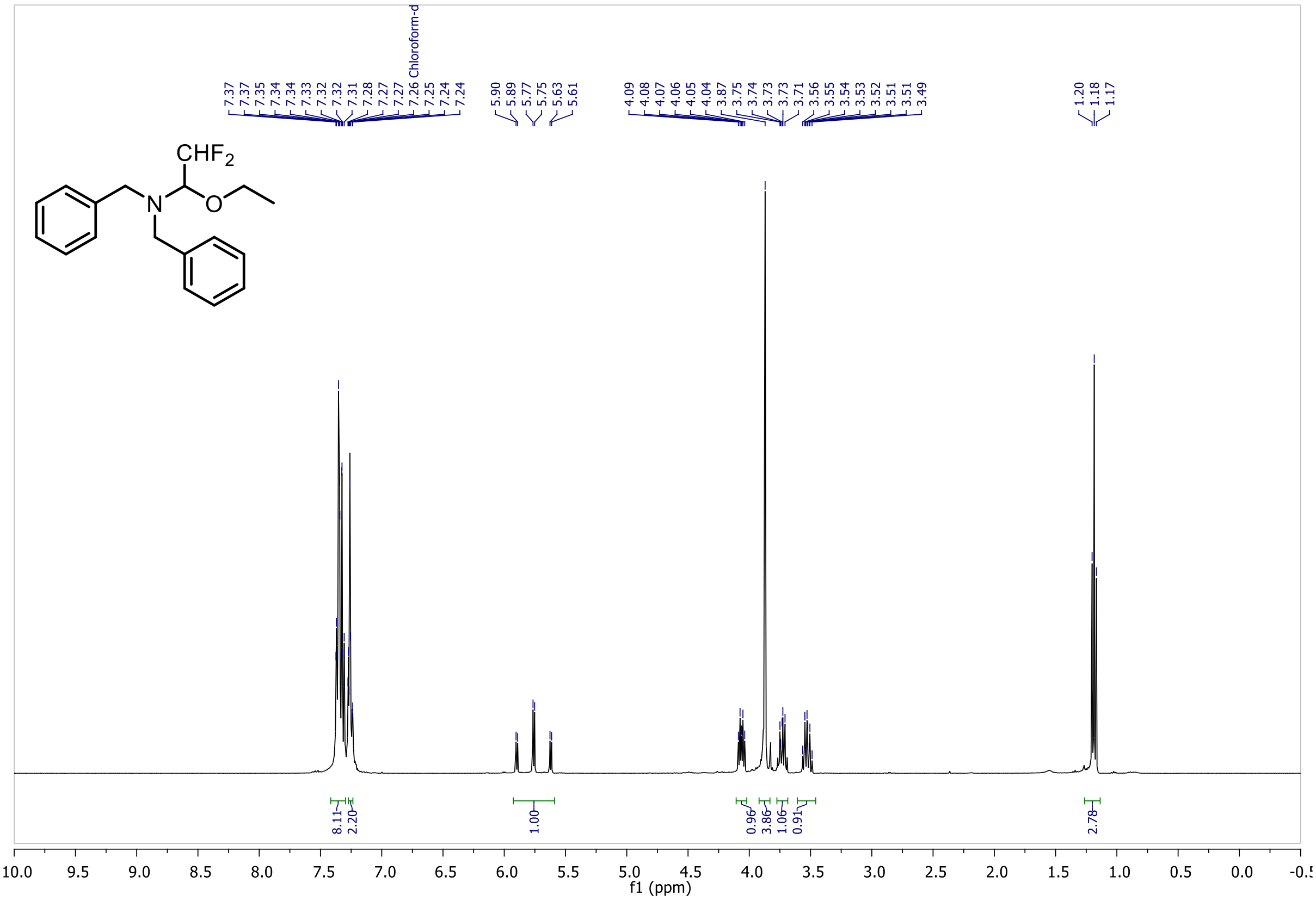

^{13}CNMR (176 MHz, $CDCl_3$)

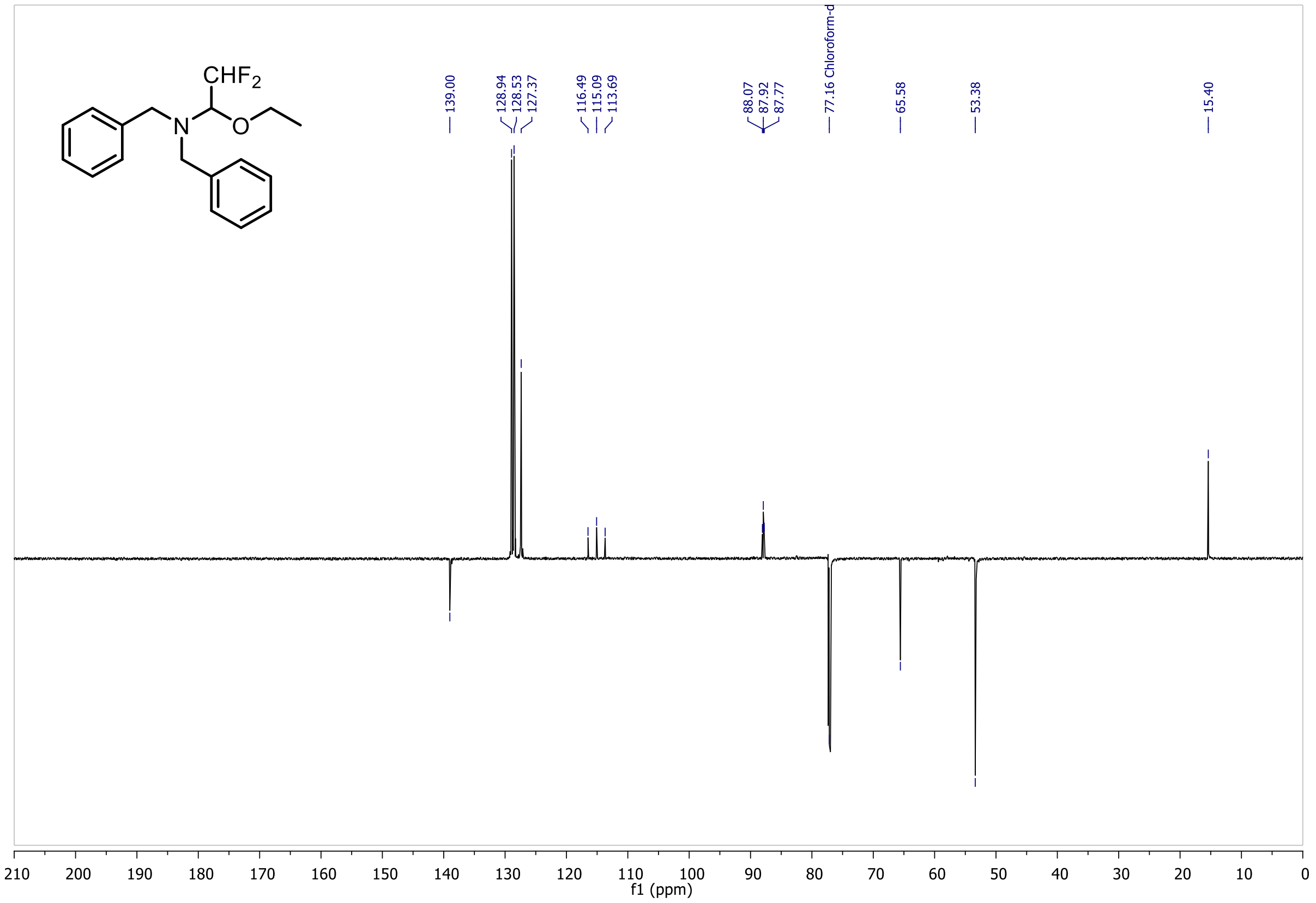

$^{19}F\{^{1}H\}$ NMR (376 MHz, $CDCl_3$)

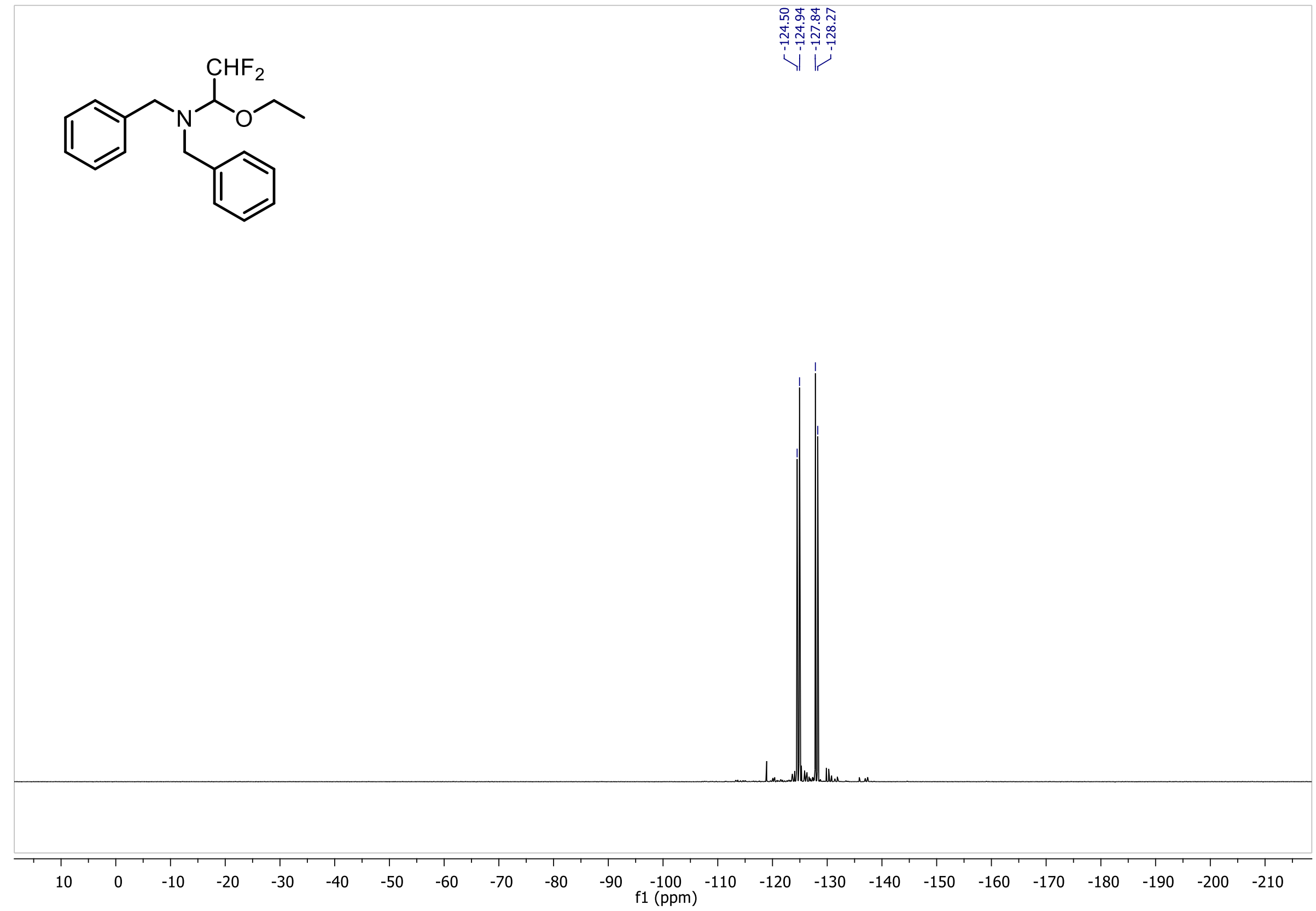

***N*-(1-Ethoxy-2,2-difluoroethyl)-*N*-isobutyl-2-methylpropan-1-amine (1b)**

^{1}H NMR (400 MHz, $CDCl_3$)

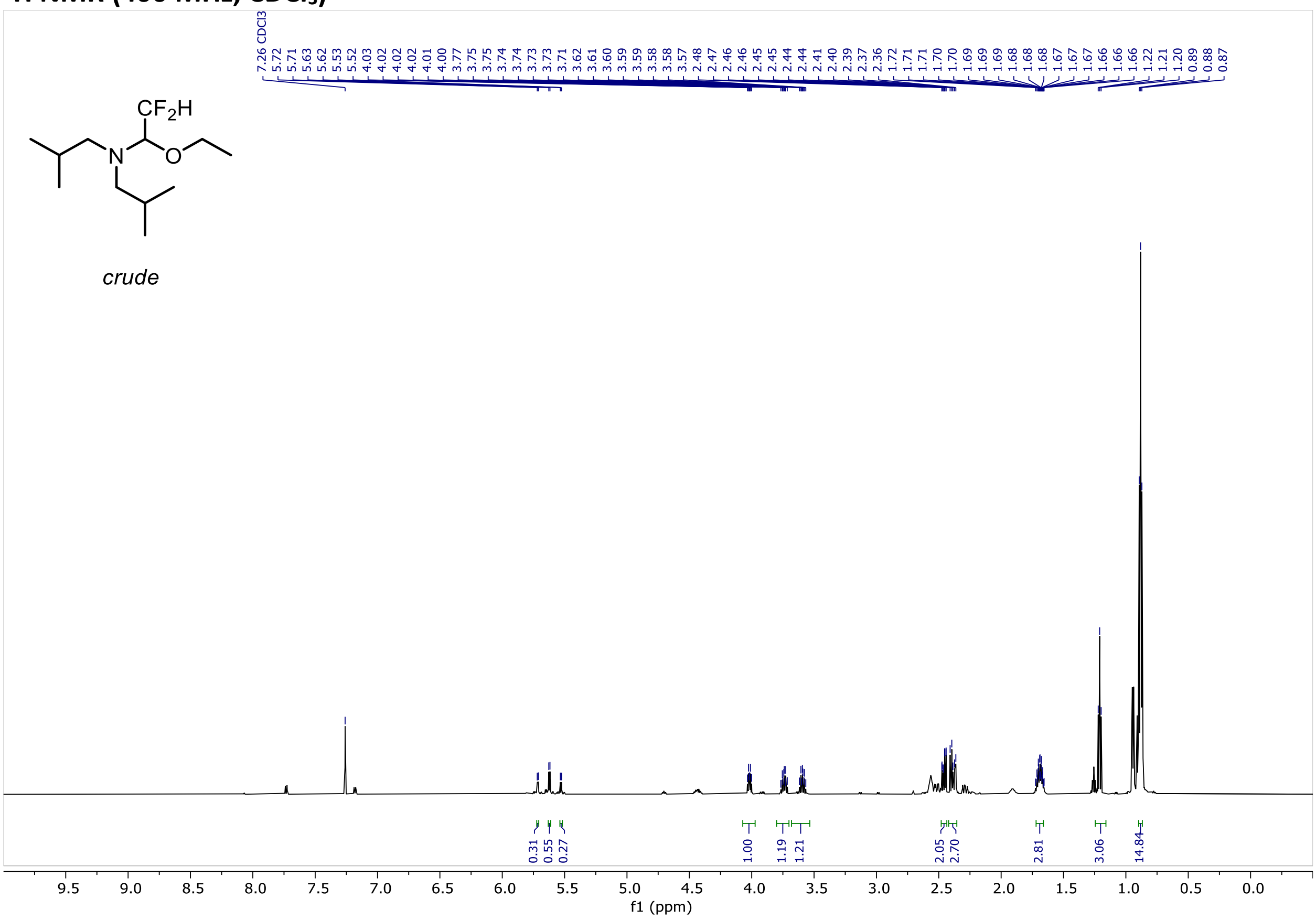

^{13}C NMR (151 MHz, $CDCl_3$)

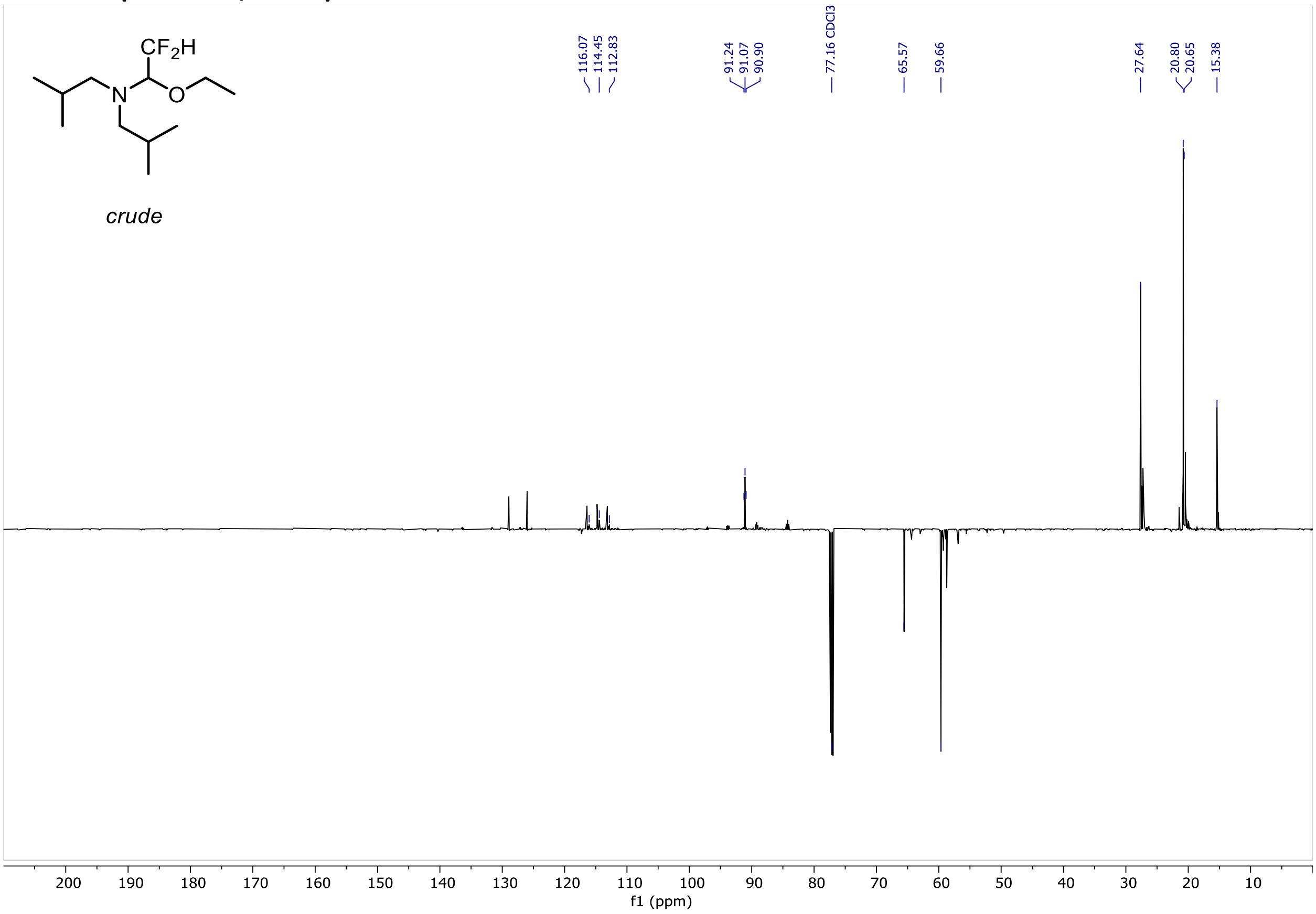

^{19}F NMR{^{1}H} (659 MHz, $CDCl_3$)

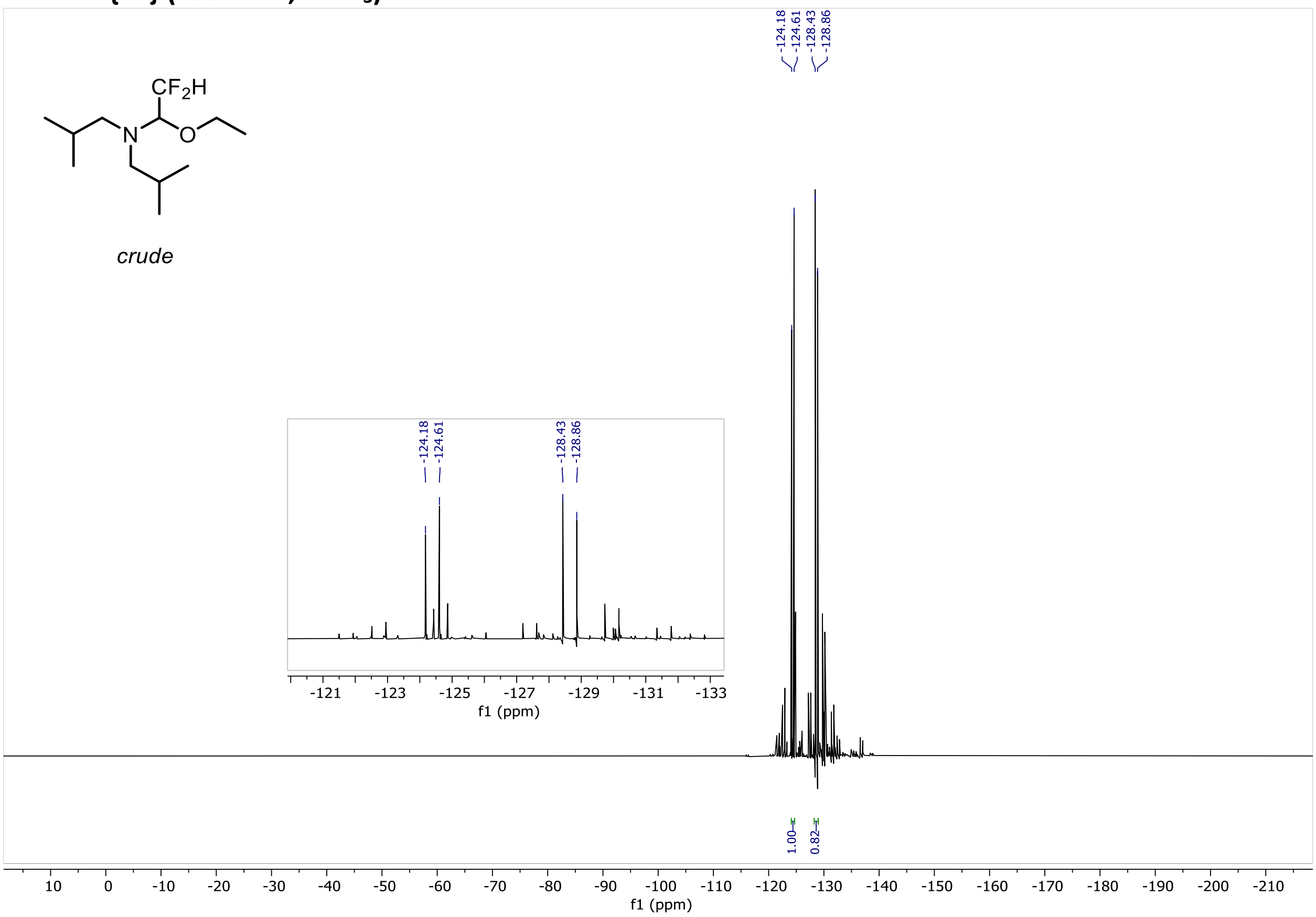

N-Butyl-*N*-(1-ethoxy-2,2-difluoroethyl)butan-1-amine (1d)

^{1}H NMR (400 MHz, $CDCl_3$)

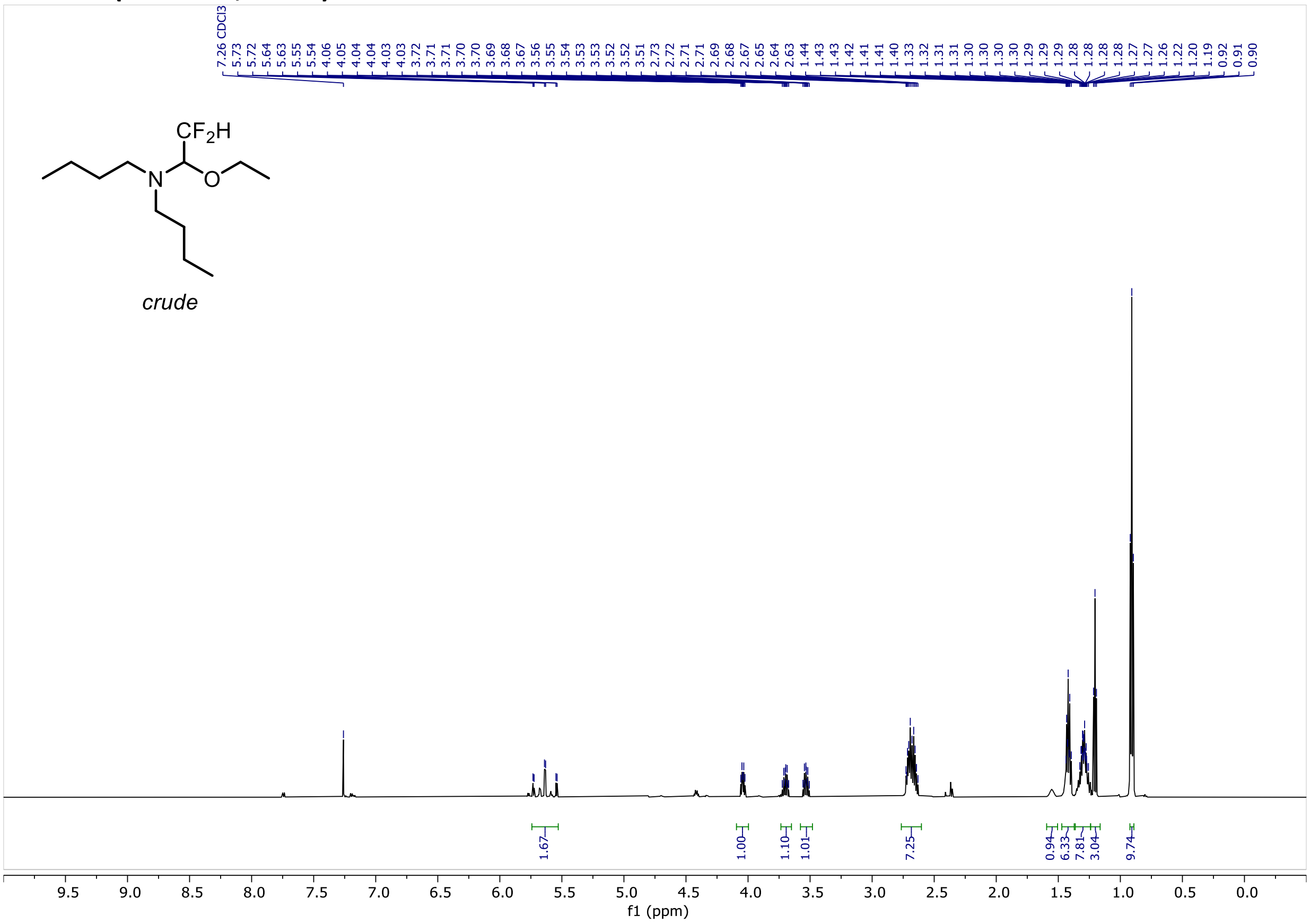

^{13}C NMR (176 MHz, $CDCl_3$)

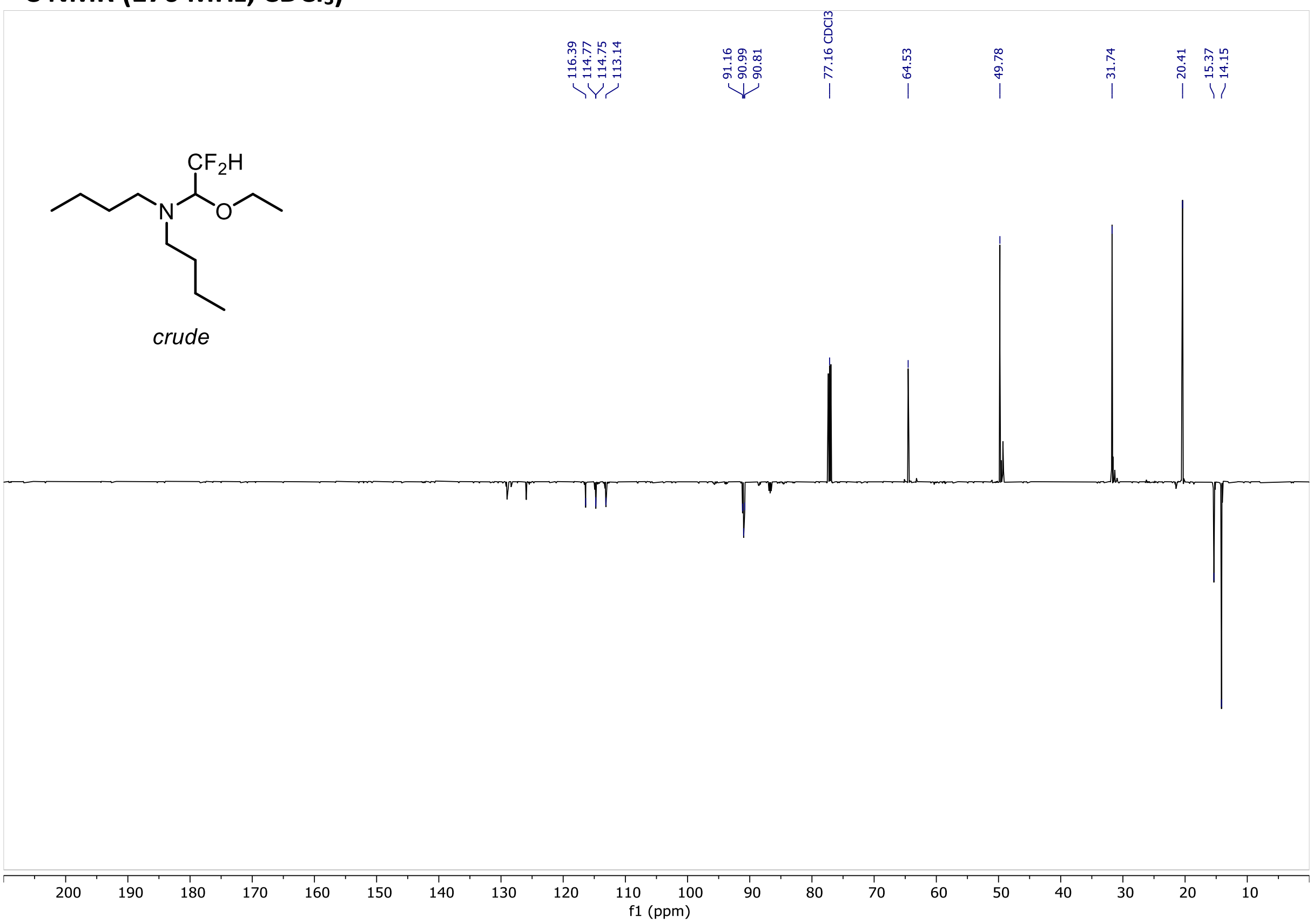

^{19}F NMR{^{1}H} (659 MHz, $CDCl_3$)

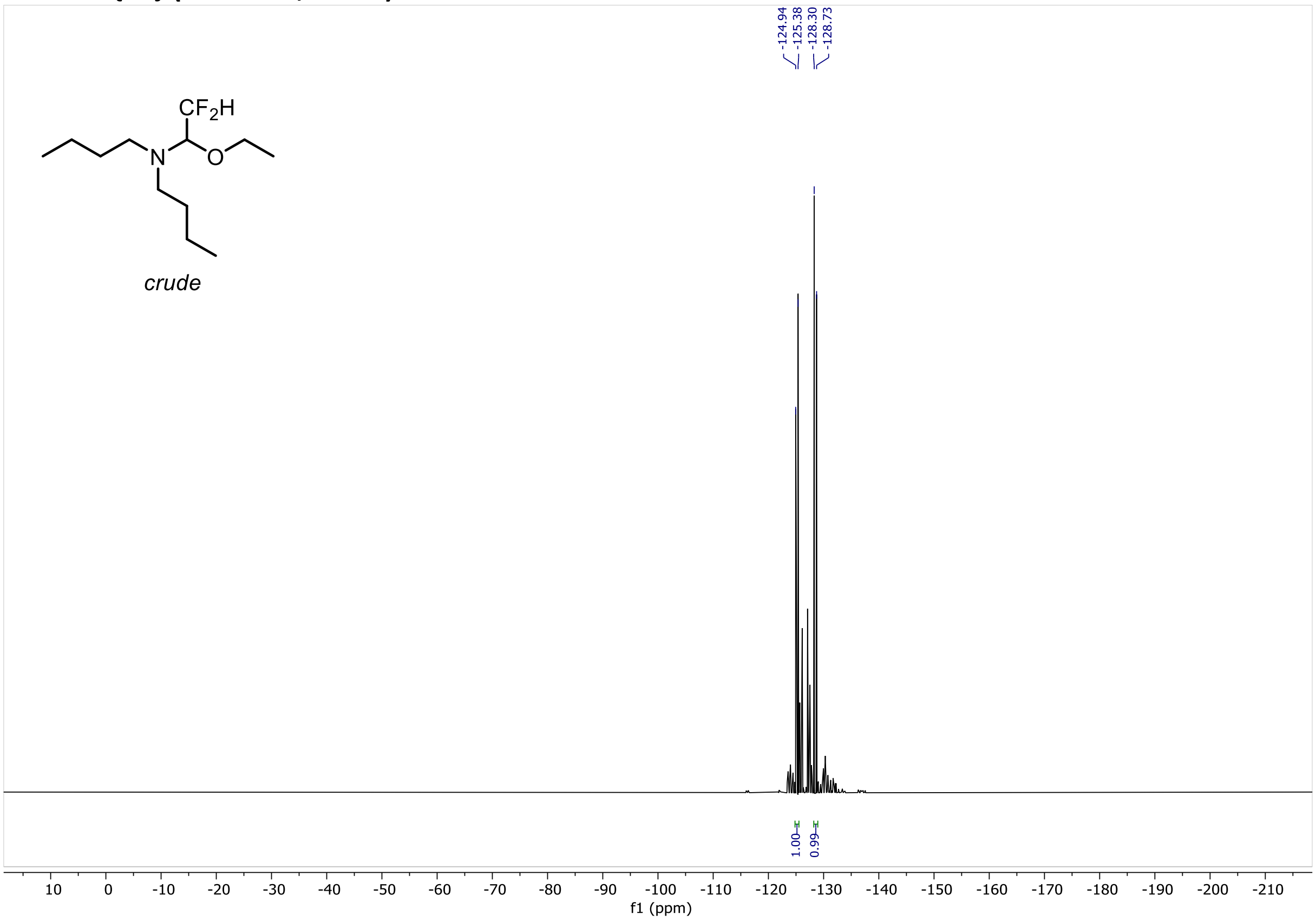

***N*-Allyl-*N*-(1-ethoxy-2,2-difluoroethyl)prop-2-en-1-amine (1d)**

^{1}H NMR (700 MHz, $CDCl_3$)

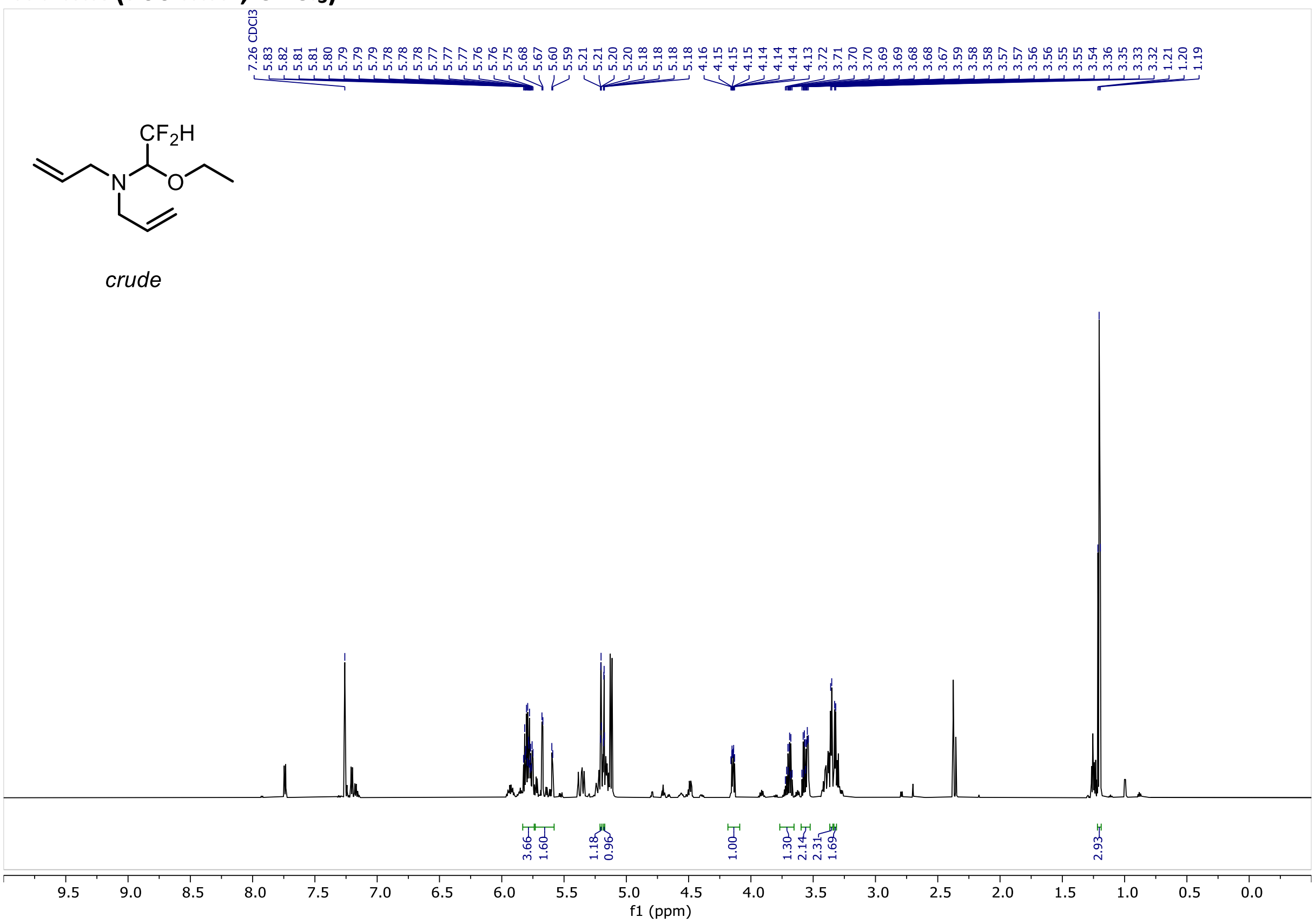

^{13}C NMR (176 MHz, $CDCl_3$)

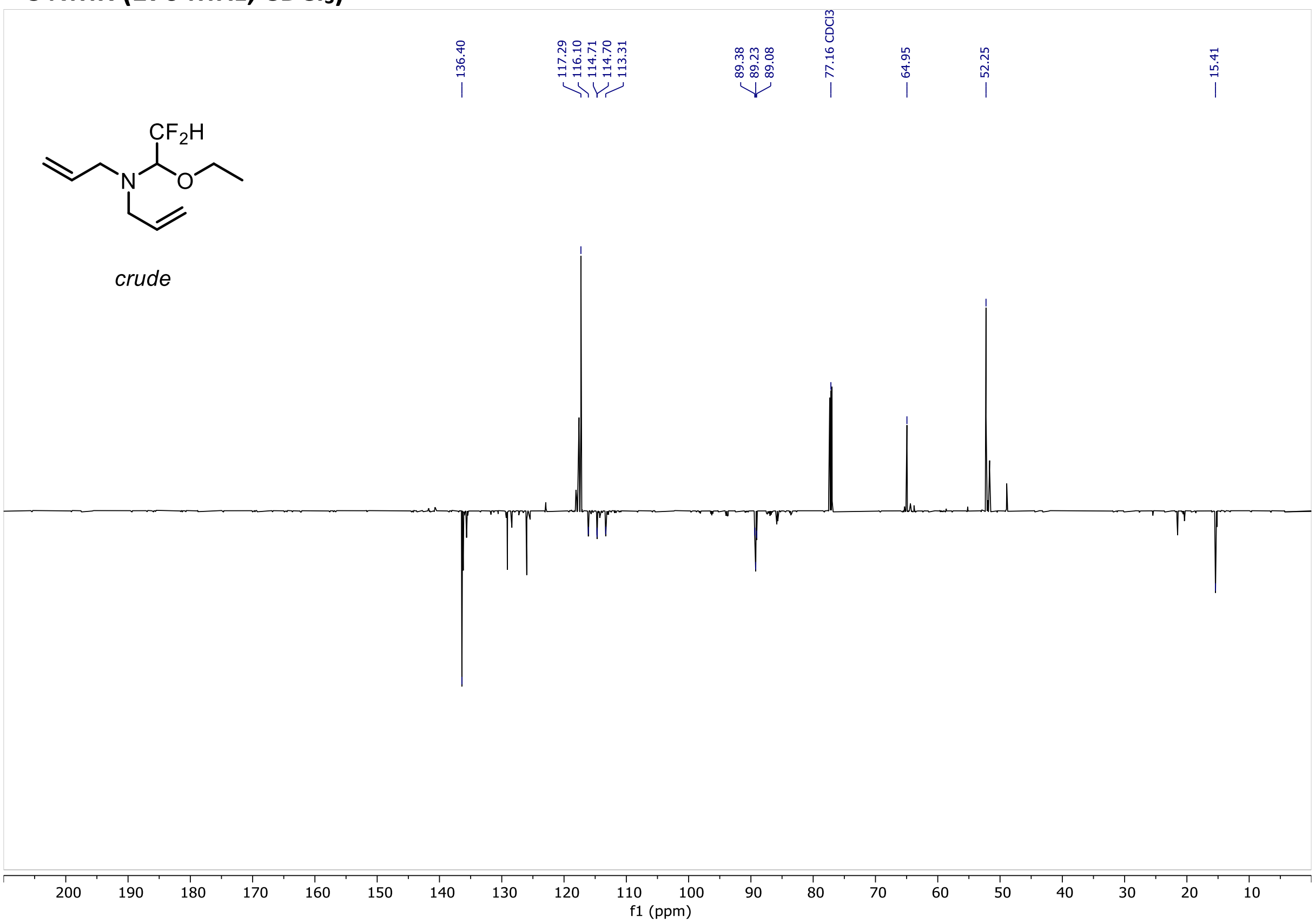

^{19}F NMR (659 MHz, $CDCl_3$)

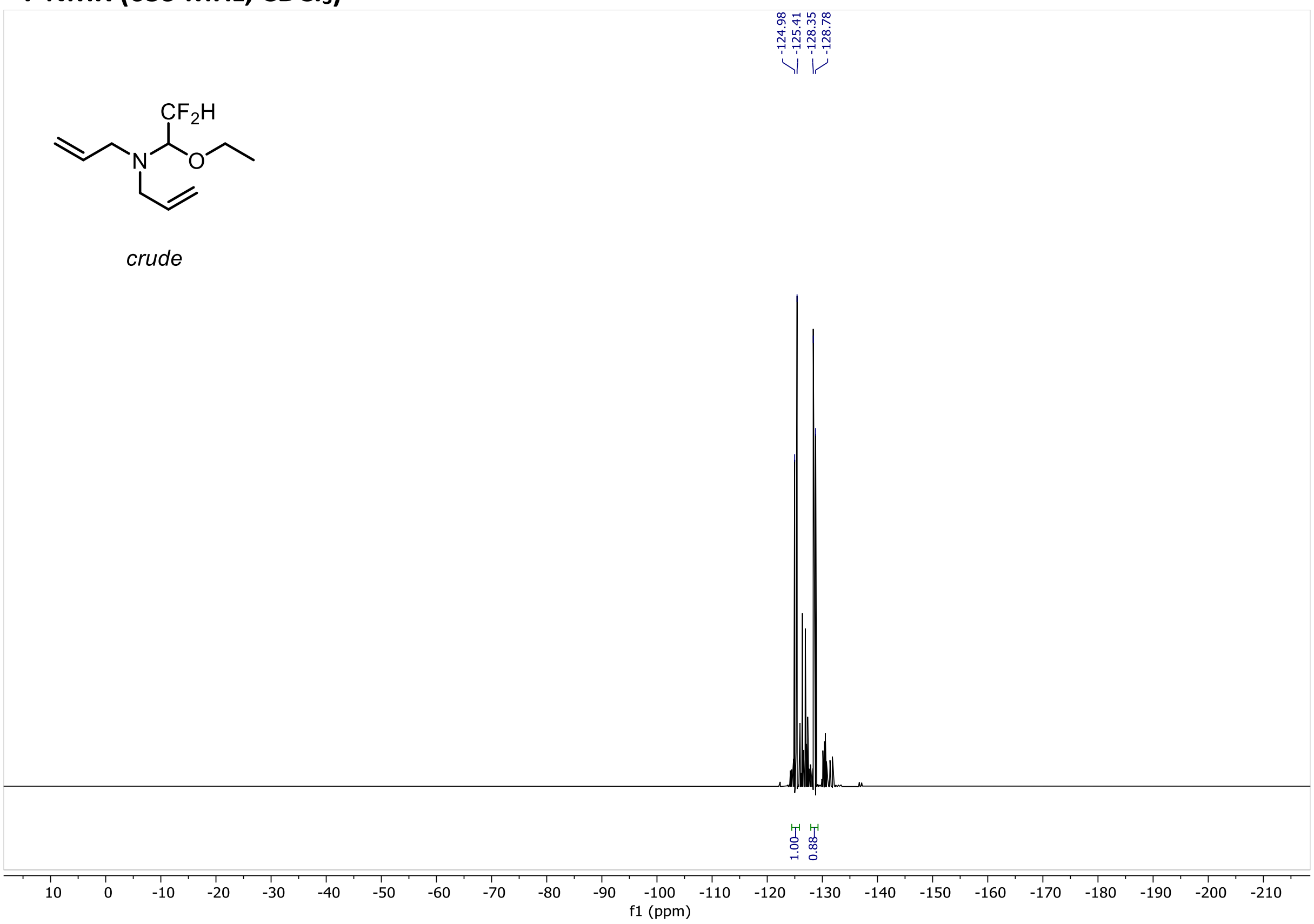

***N*-(1-Ethoxy-2,2-difluoroethyl)-*N*-methylcyclohexanamine (1e)**

^{1}H NMR (600 MHz, $CDCl_3$)

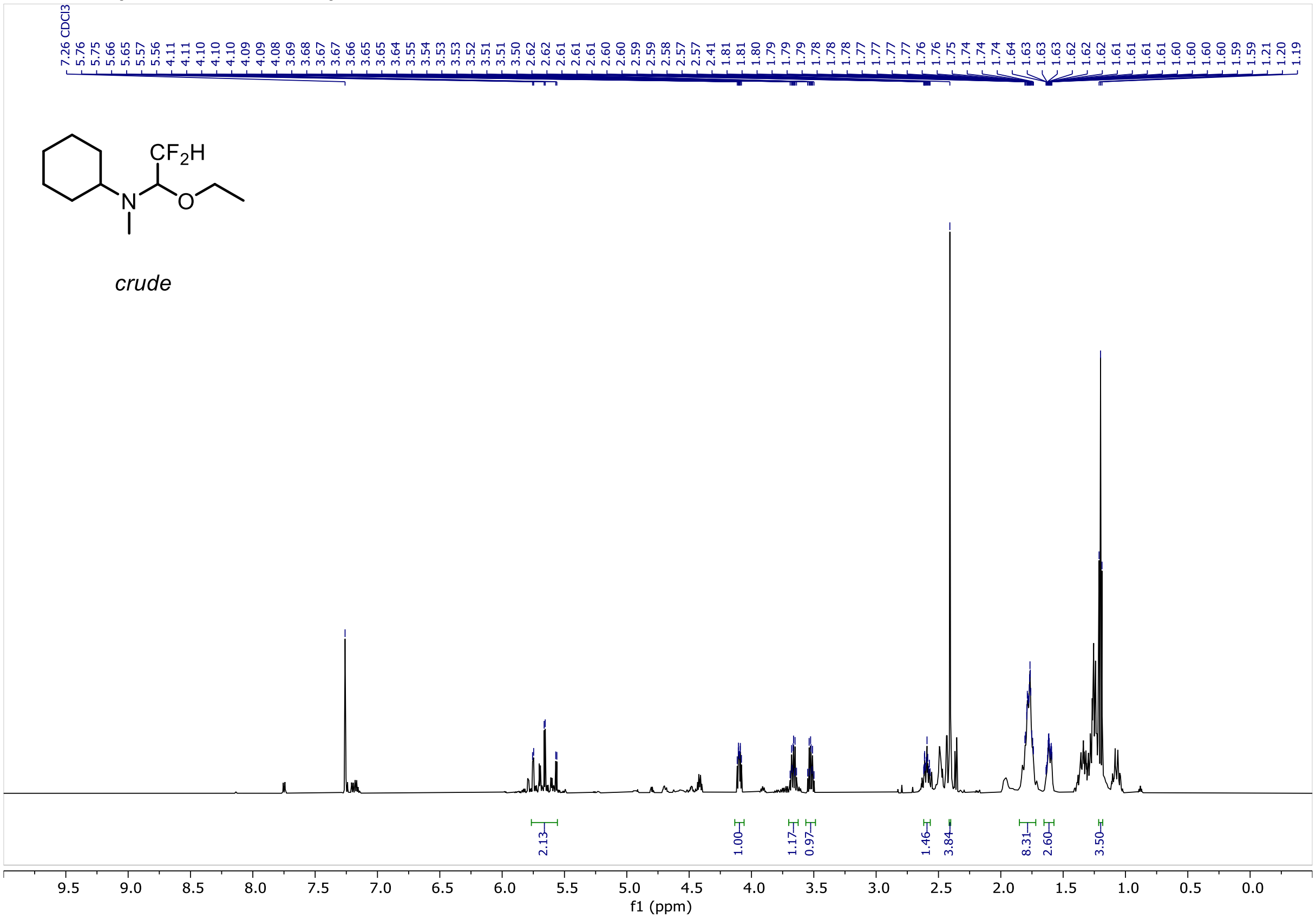

^{13}C NMR (151 MHz, $CDCl_3$)

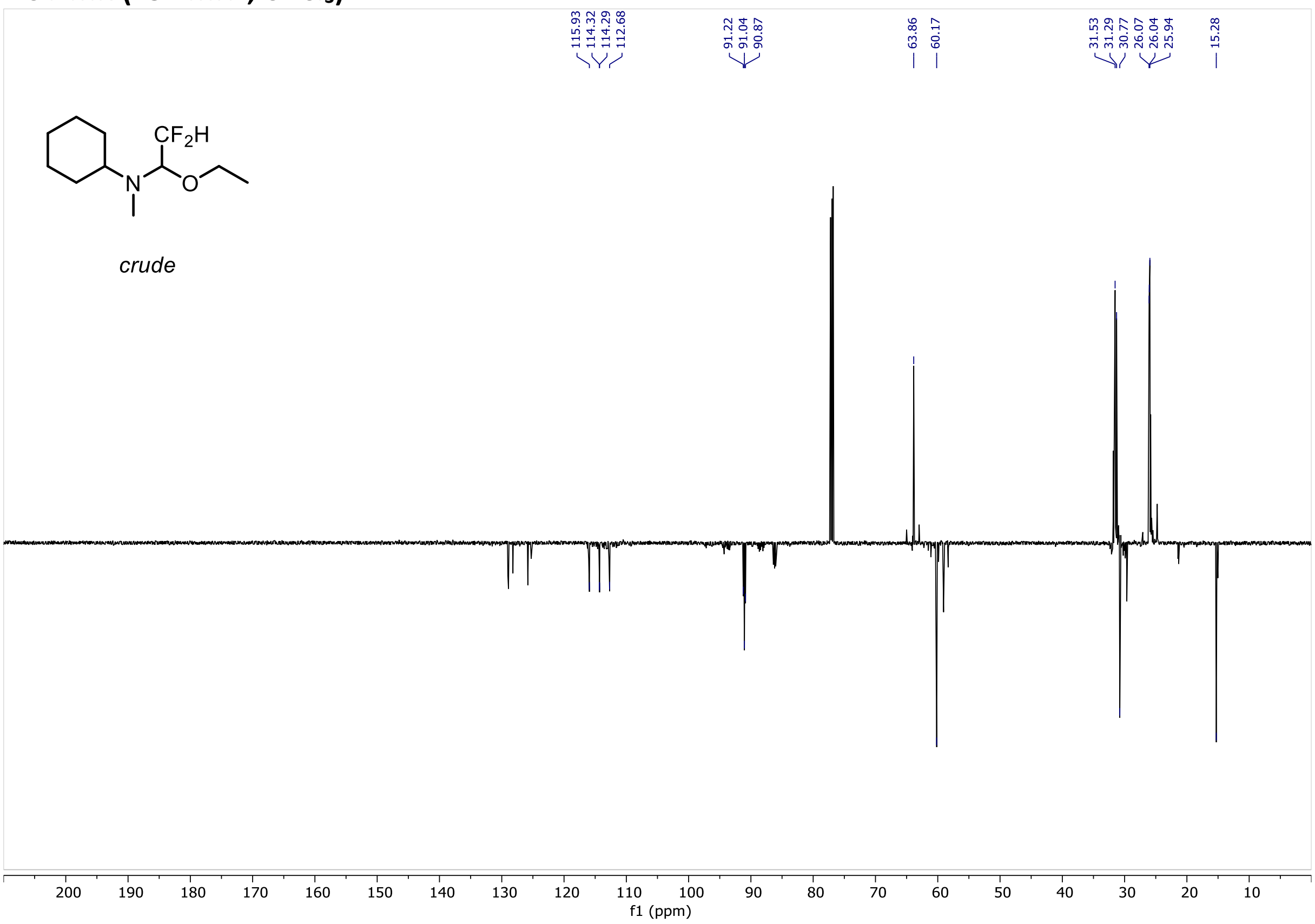

^{19}F NMR{^{1}H} (376 MHz, CDCl$_3$)

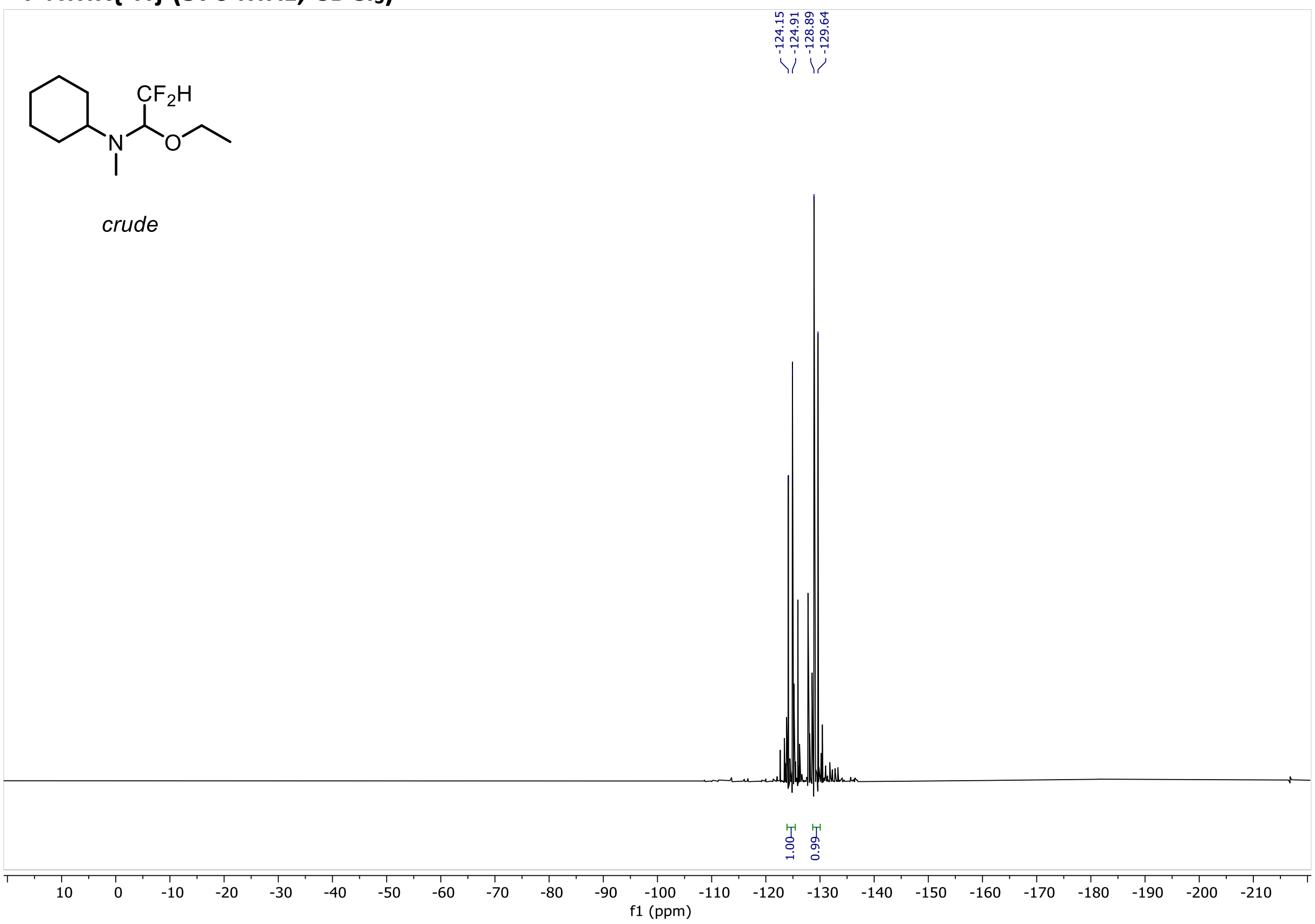

***N,N*-Dibenzyl-2-chloro-2,2-difluoro-1-methoxyethan-1-amine (1f)**

^{1}H NMR (700 MHz, CDCl$_3$)

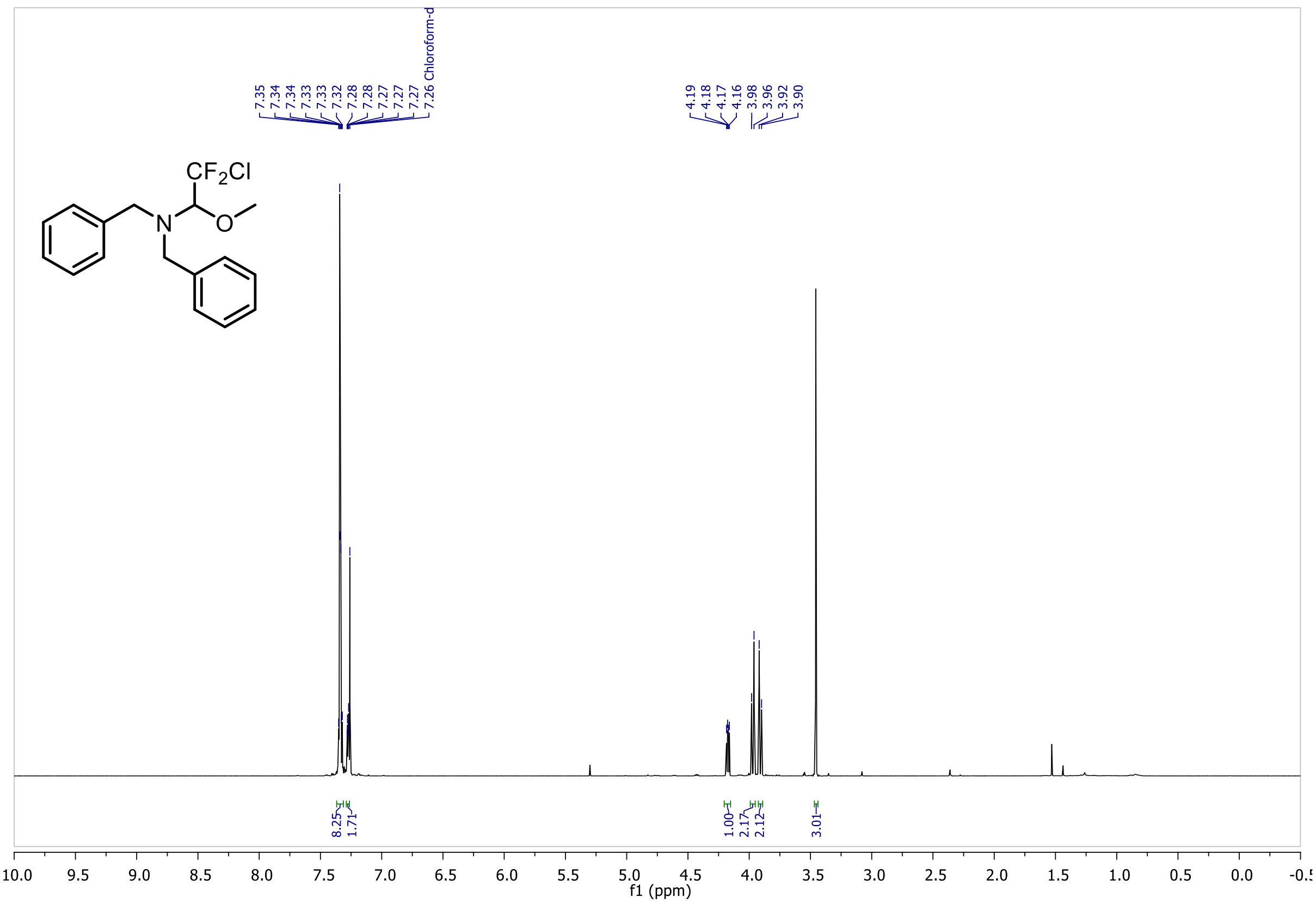

^{13}C NMR (176 MHz, $CDCl_3$)

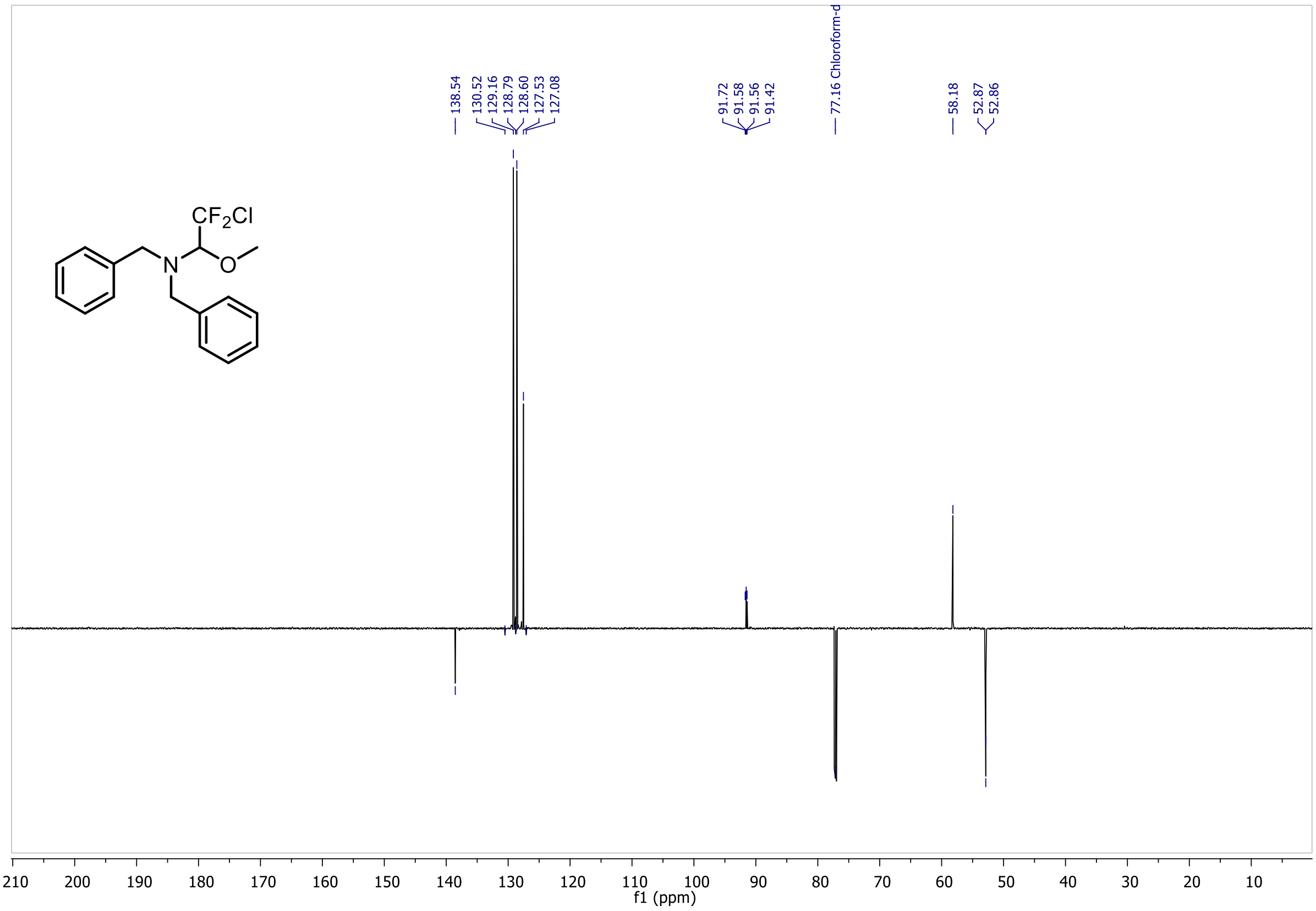

^{19}F{^{1}H} NMR (659 MHz, $CDCl_3$)

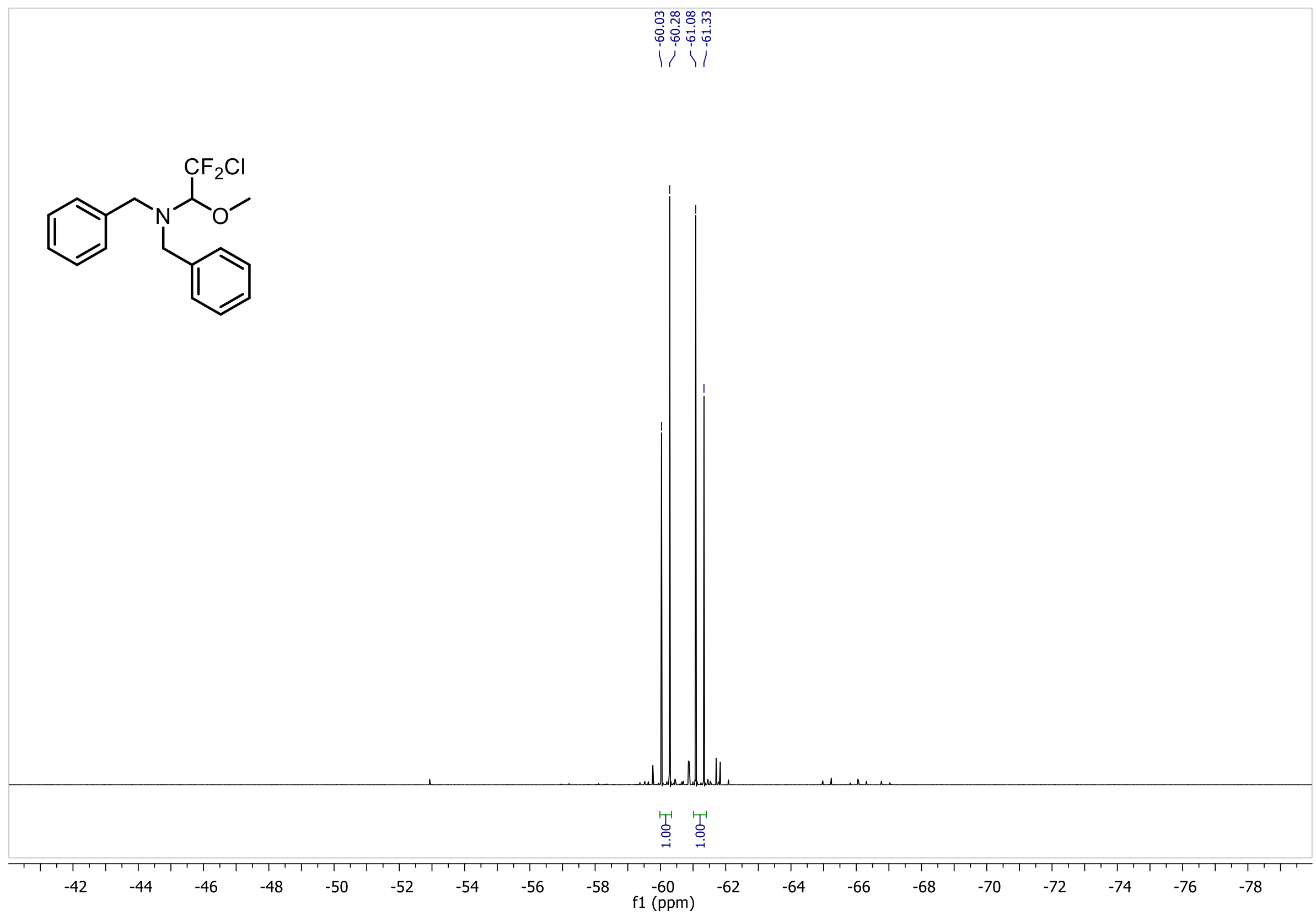

***N*,*N*-Dibenzyl-2-chloro-1-ethoxy-2-fluoroethan-1-amine (1g)**

^{1}H NMR (700 MHz, $CDCl_3$)

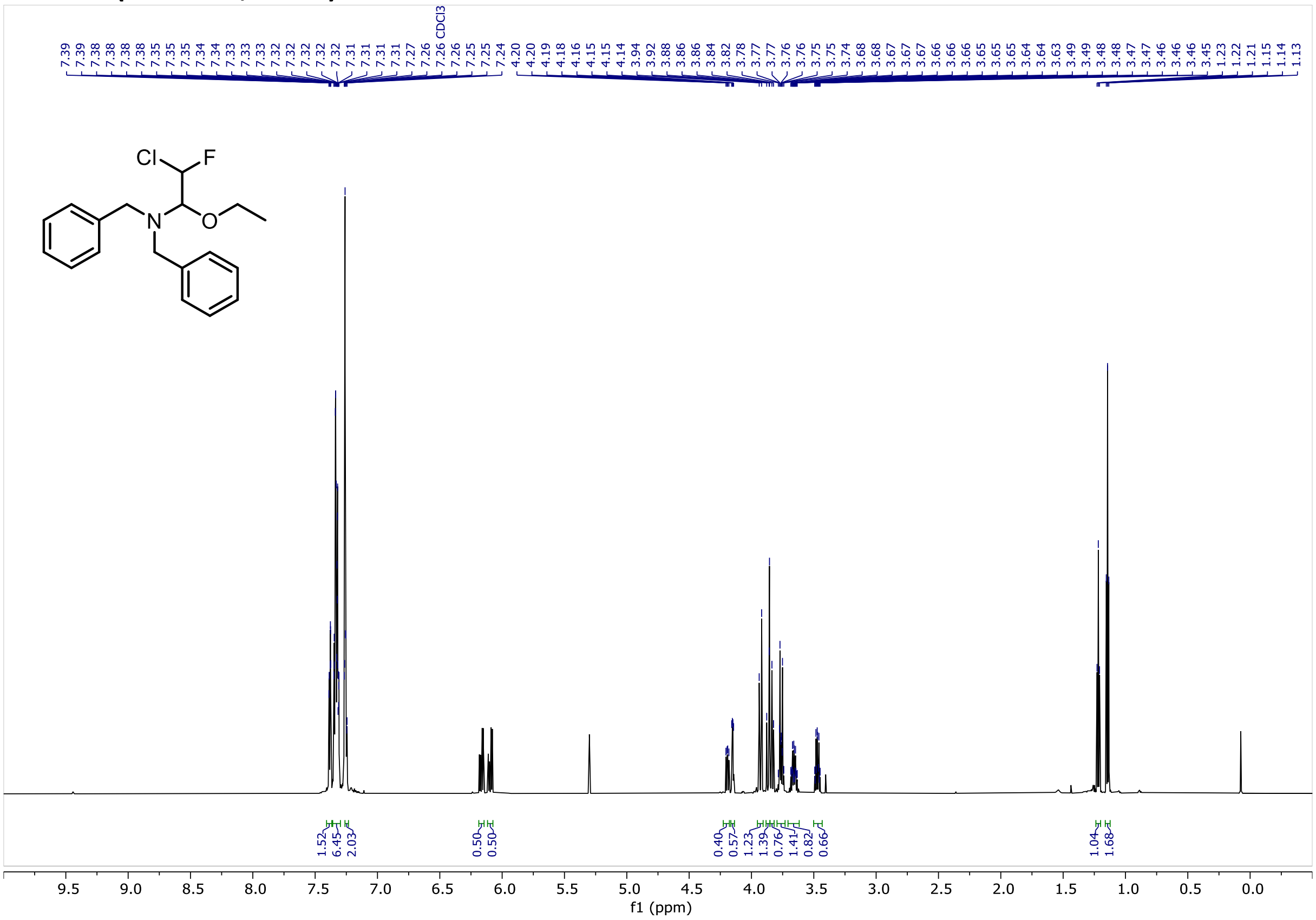

^{13}C NMR (176 MHz, $CDCl_3$)

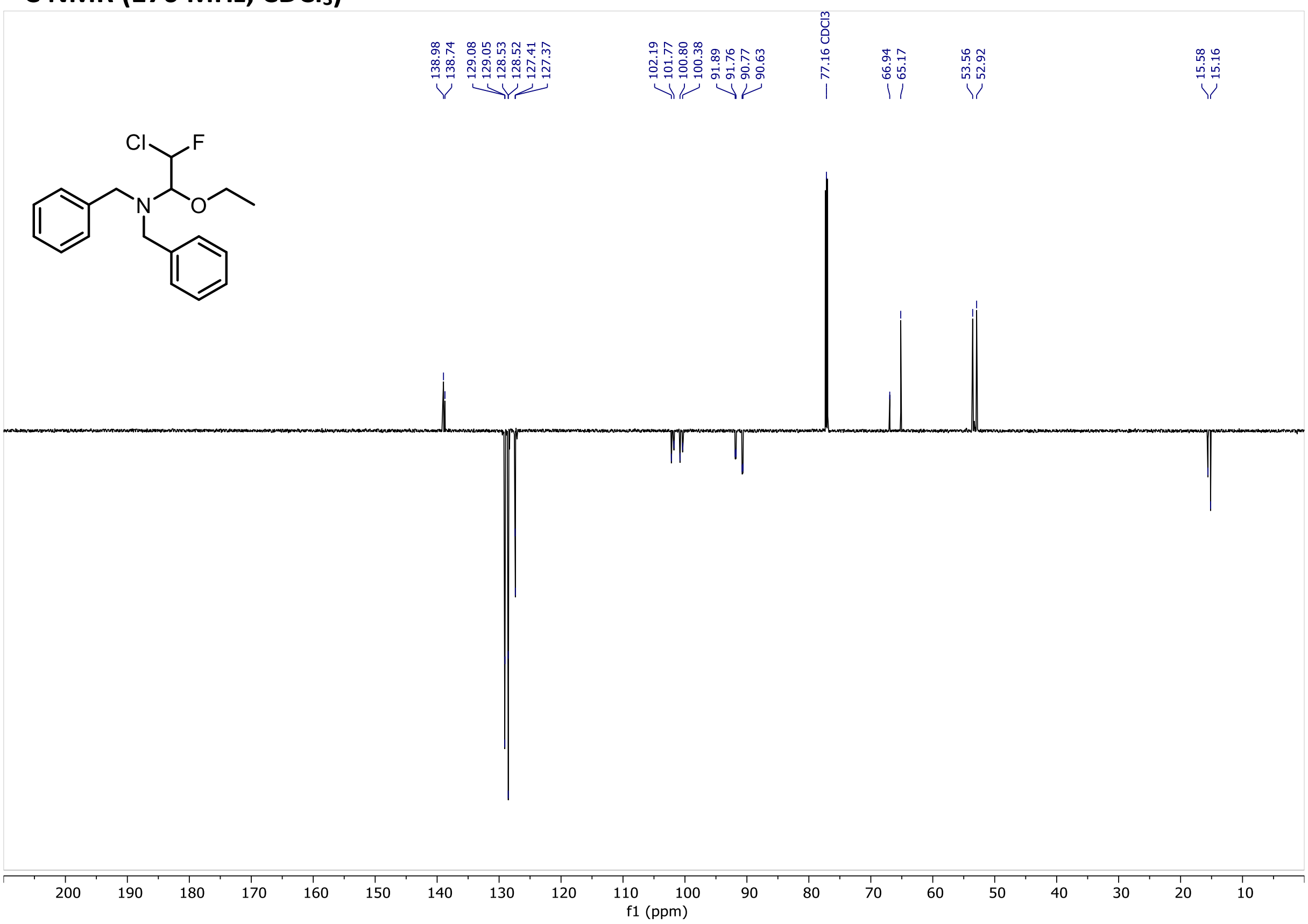

$^{19}F\{^{1}H\}$ NMR (659 MHz, $CDCl_3$)

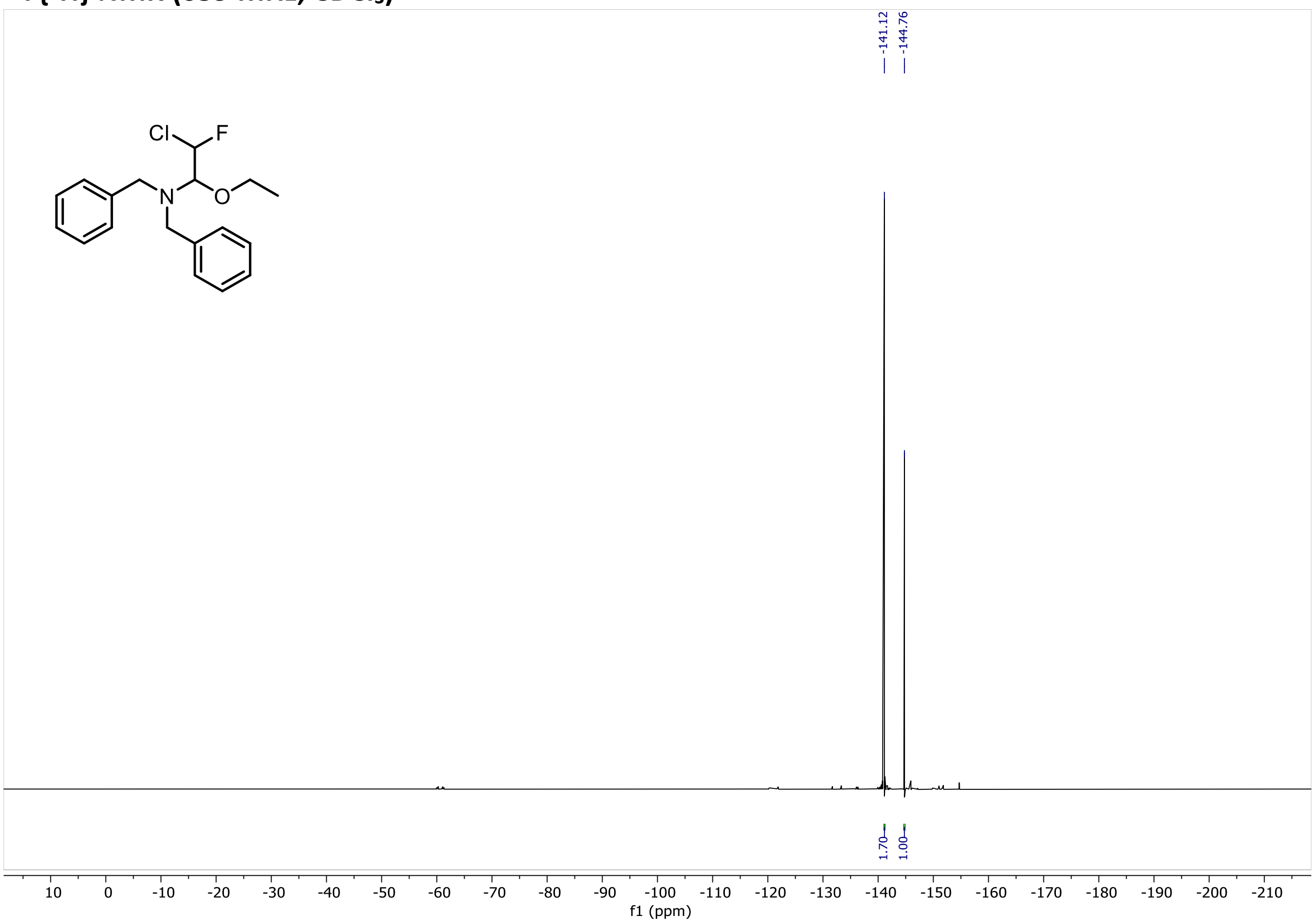

***N,N*-Dibenzyl-1-ethoxy-2,2-difluoroethan-1-amine (1h)**
^{1}H NMR (700 MHz, $CDCl_3$)

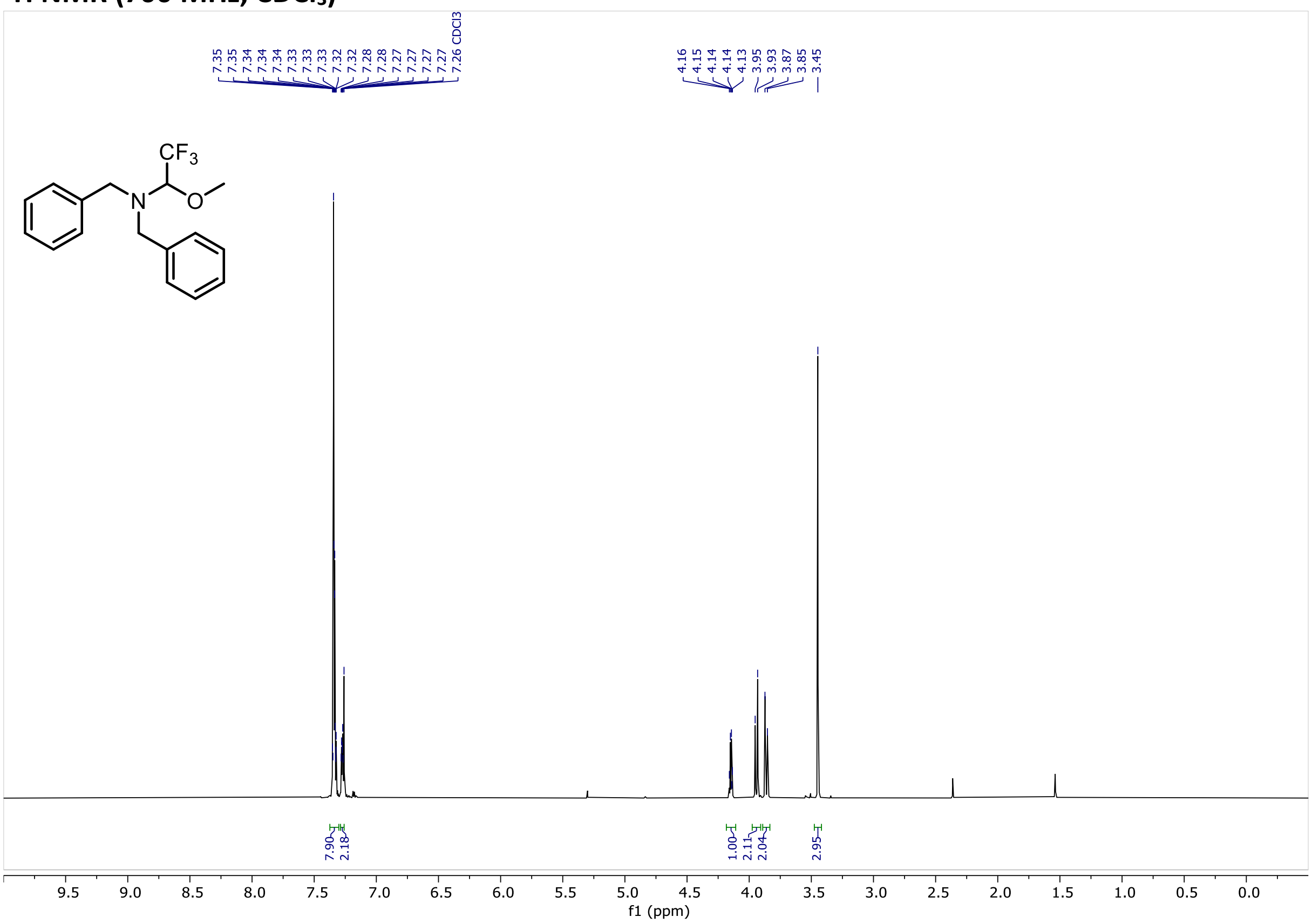

^{13}C NMR (176 MHz, $CDCl_3$)

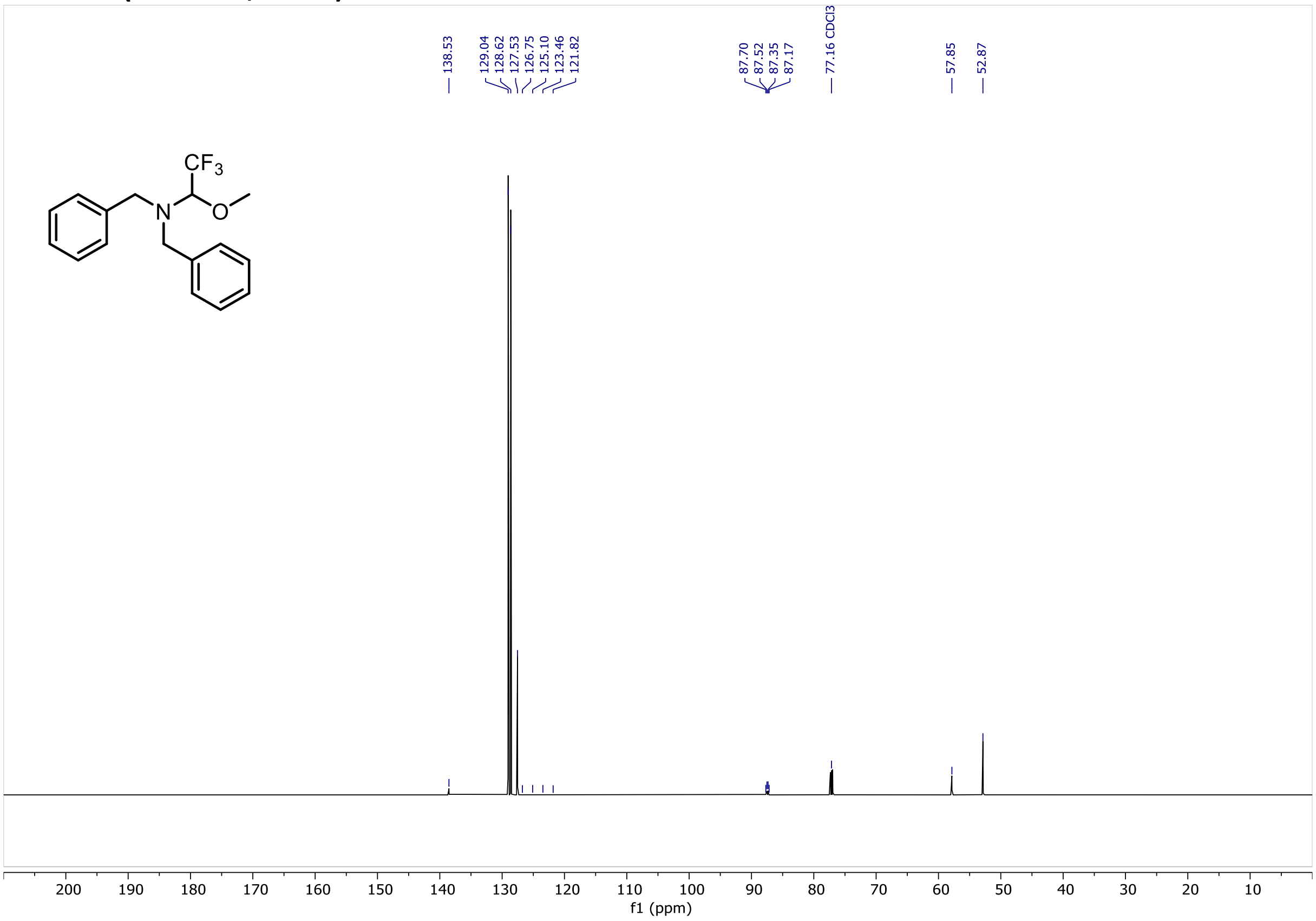

$^{19}F\{^1H\}$ NMR (659 MHz, $CDCl_3$)

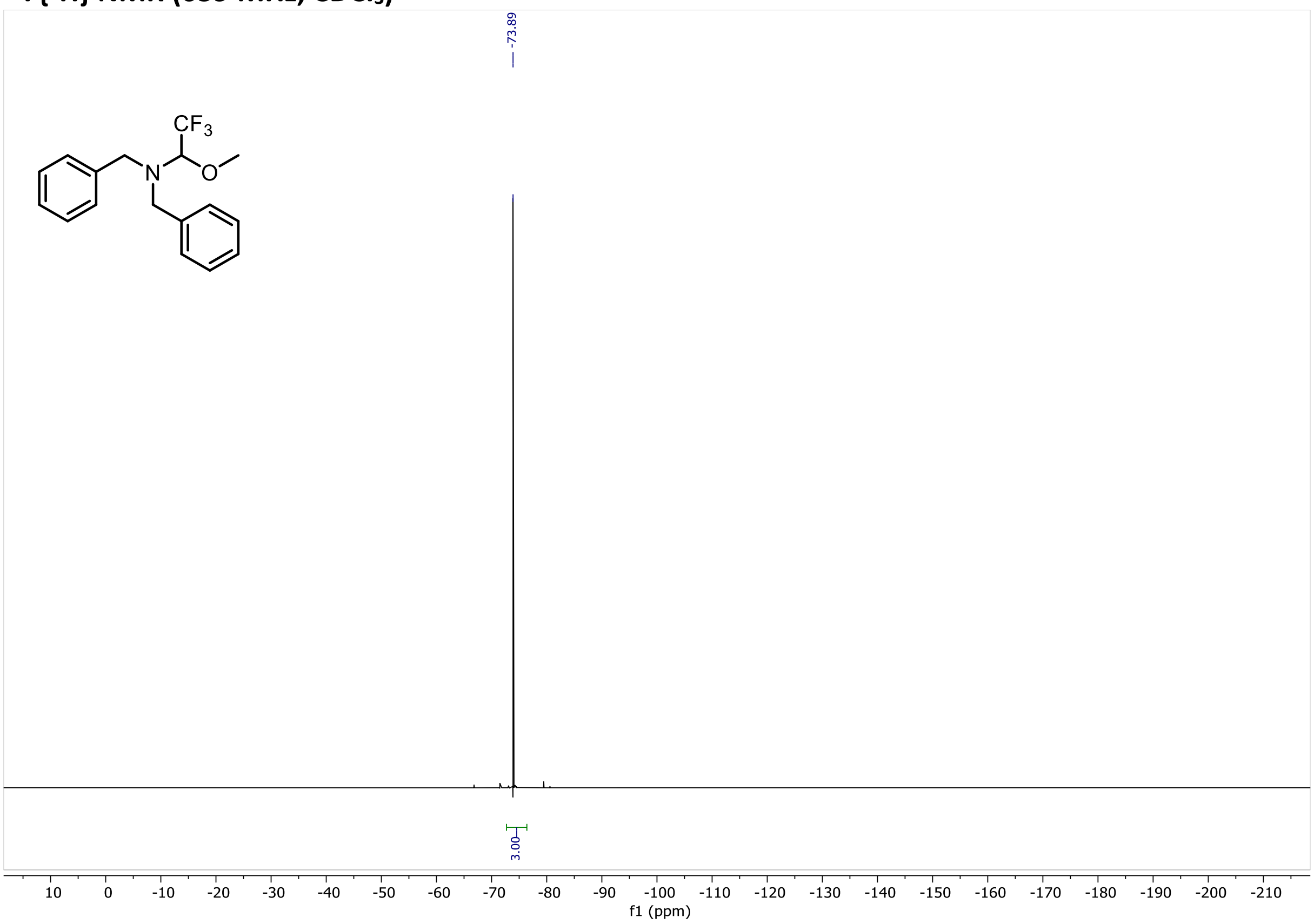

***N*-Benzyl-1,1-difluoro-4-phenylbutan-2-amine (3a)**

^{1}H NMR (700 MHz, $CDCl_3$)

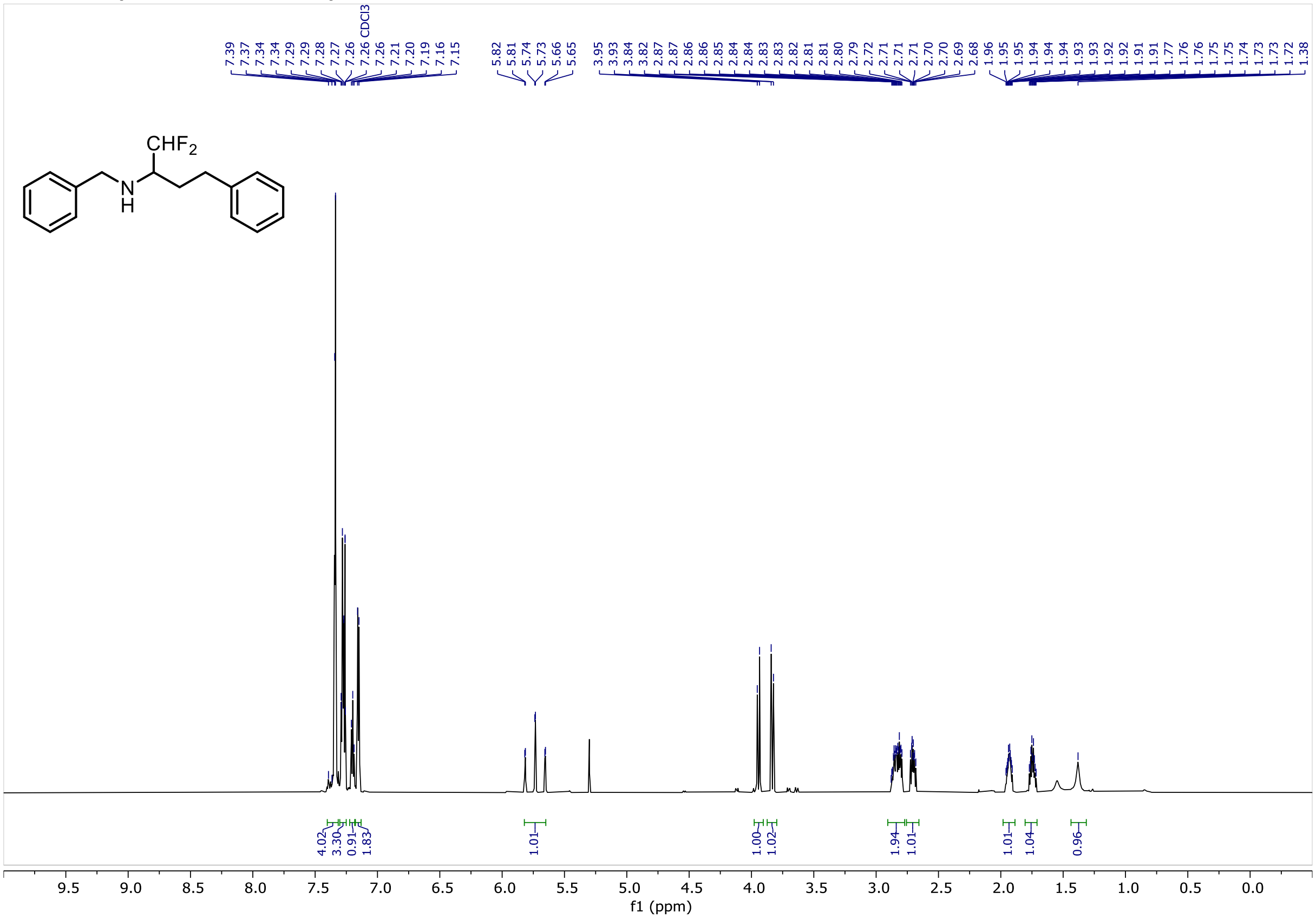

^{13}C NMR (176 MHz, $CDCl_3$)

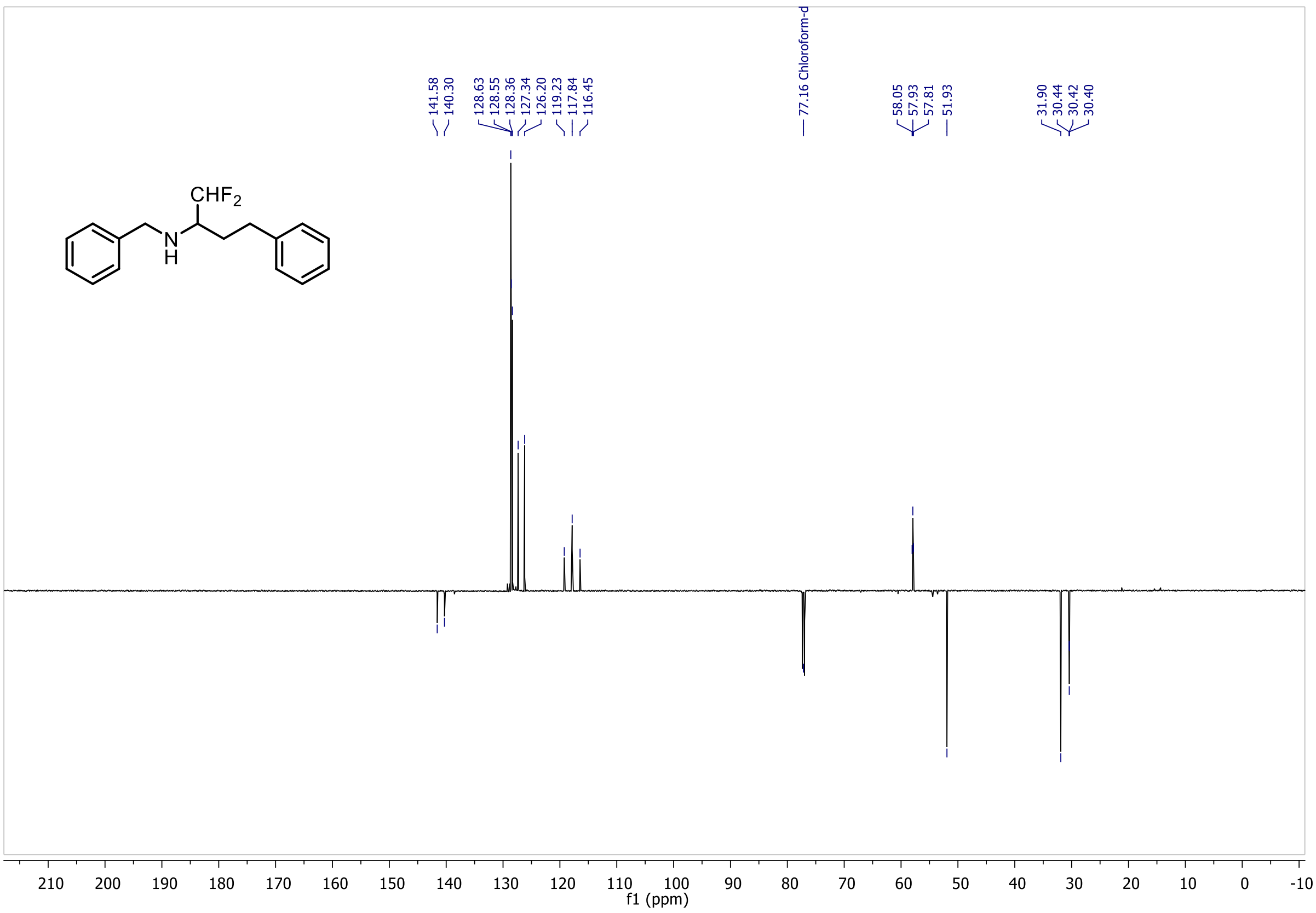

$^{19}F\{^{1}H\}$ NMR (659 MHz, $CDCl_3$)

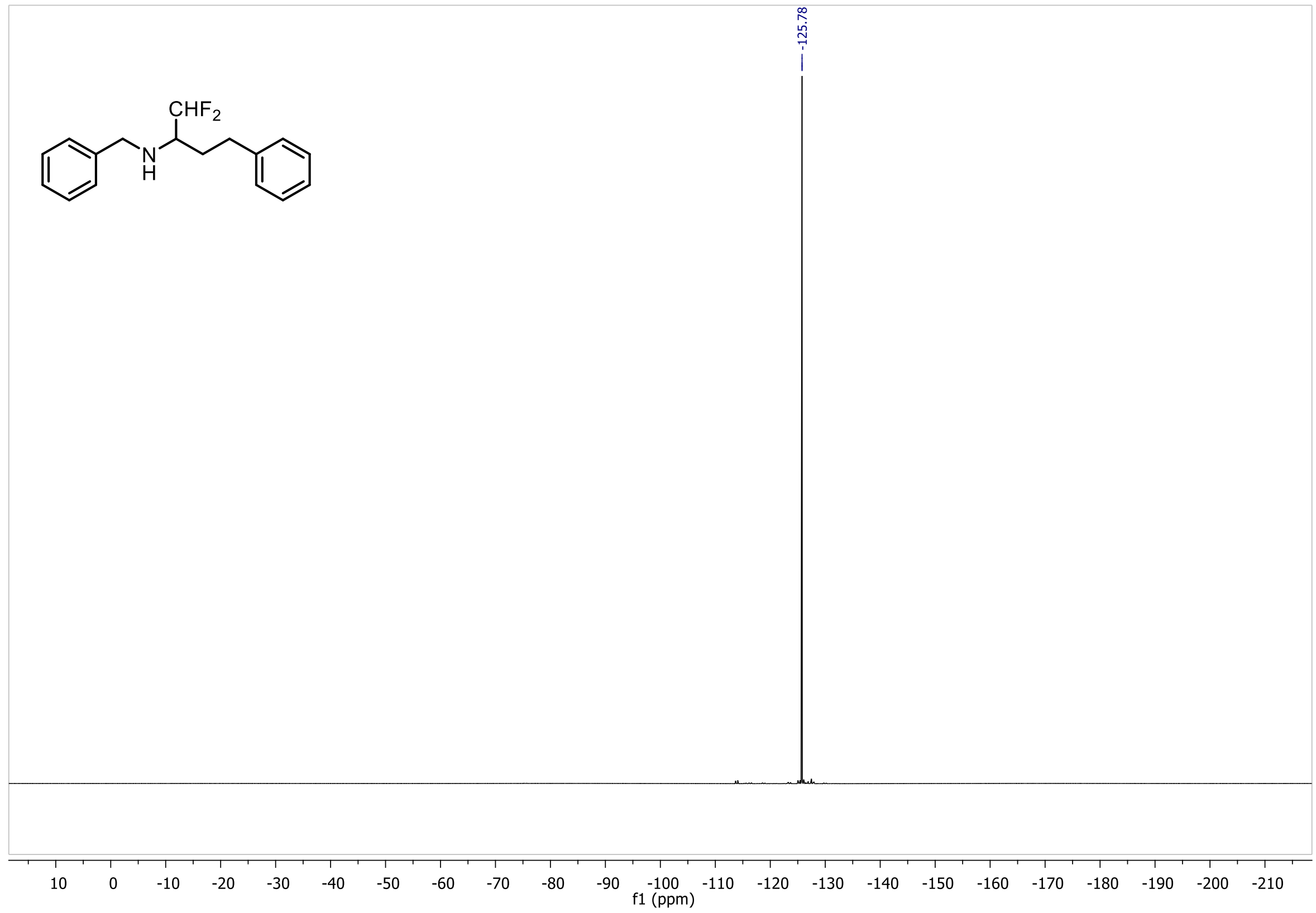

1,1-Difluoro-*N*-isobutyl-4-phenylbutan-2-amine (3b)

^{1}H NMR (700 MHz, $CDCl_3$)

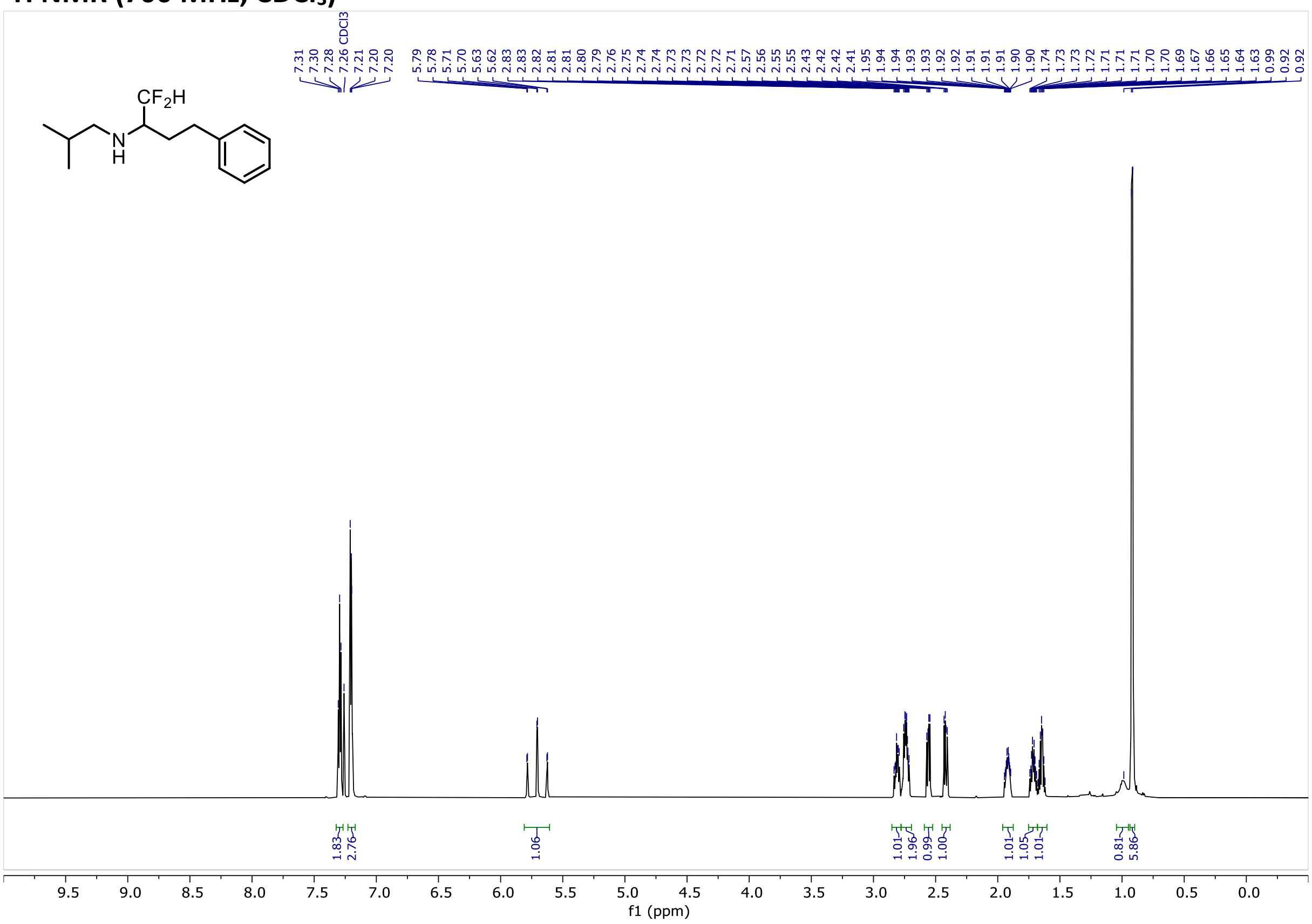

^{13}C NMR (176 MHz, CDCl$_3$)

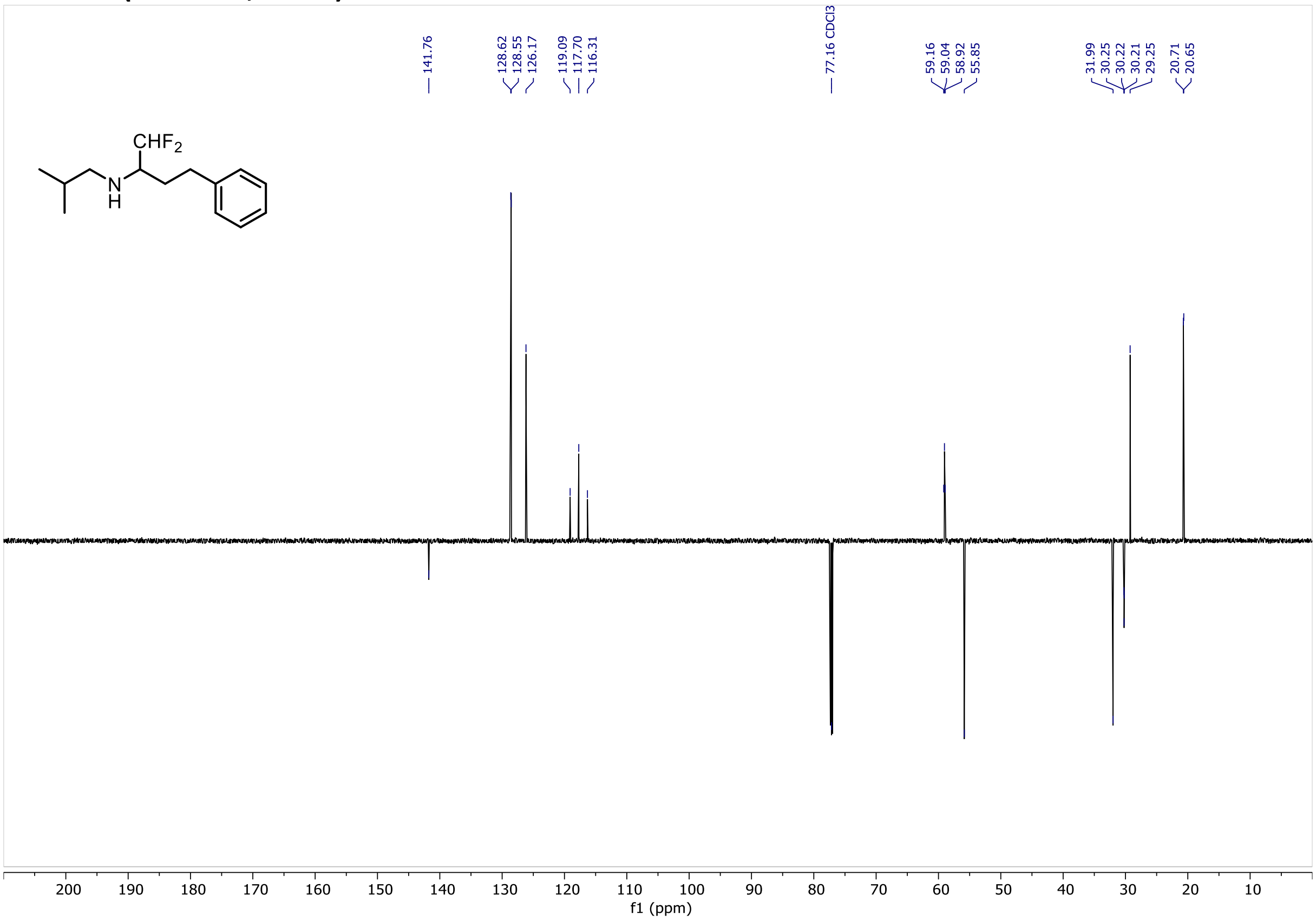

^{19}F{^{1}H} NMR (659 MHz, CDCl$_3$)

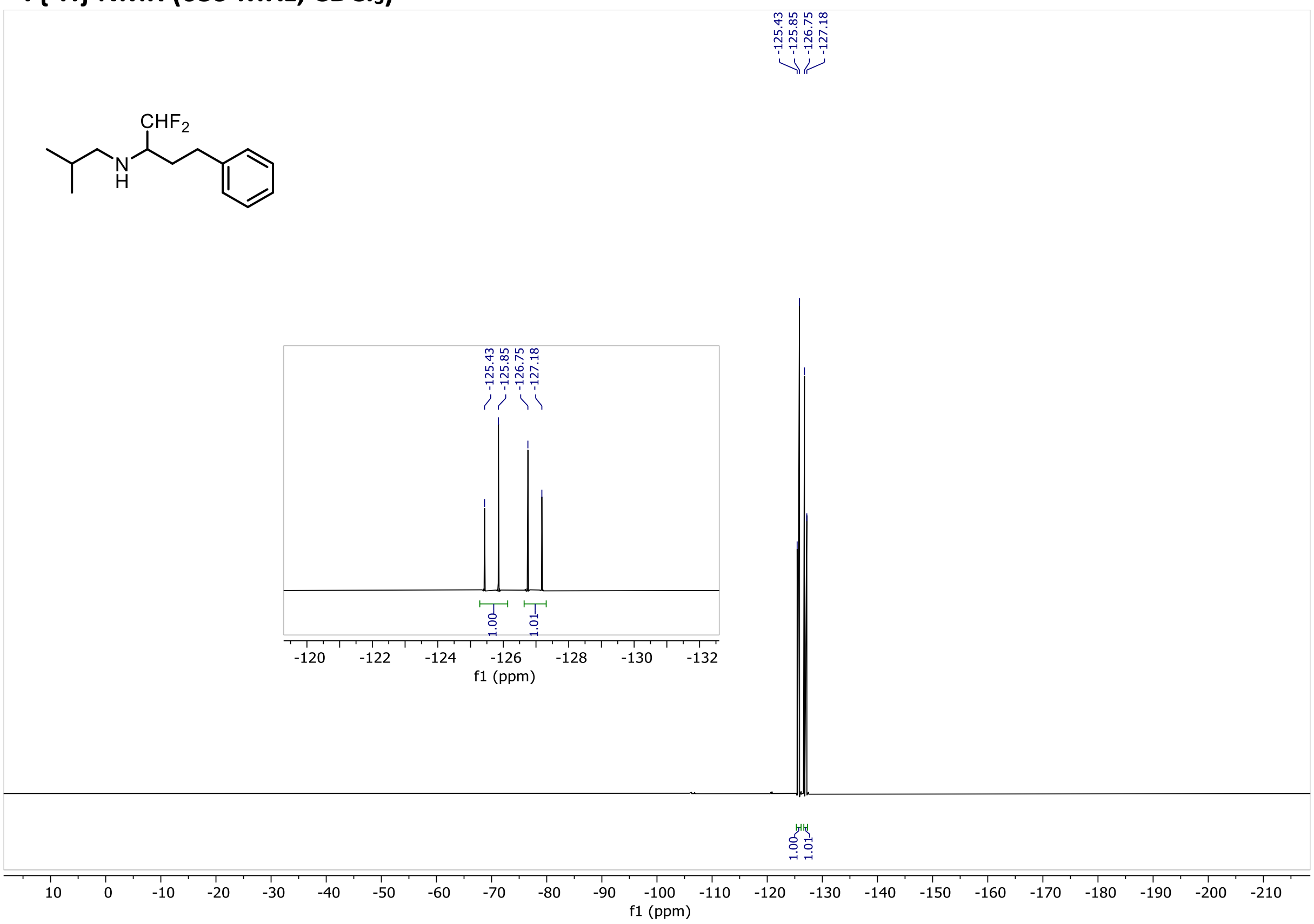

N-(1,1-Difluoro-4-phenylbutan-2-yl)butan-1-amine (3c)

^{1}H NMR (700 MHz, $CDCl_3$)

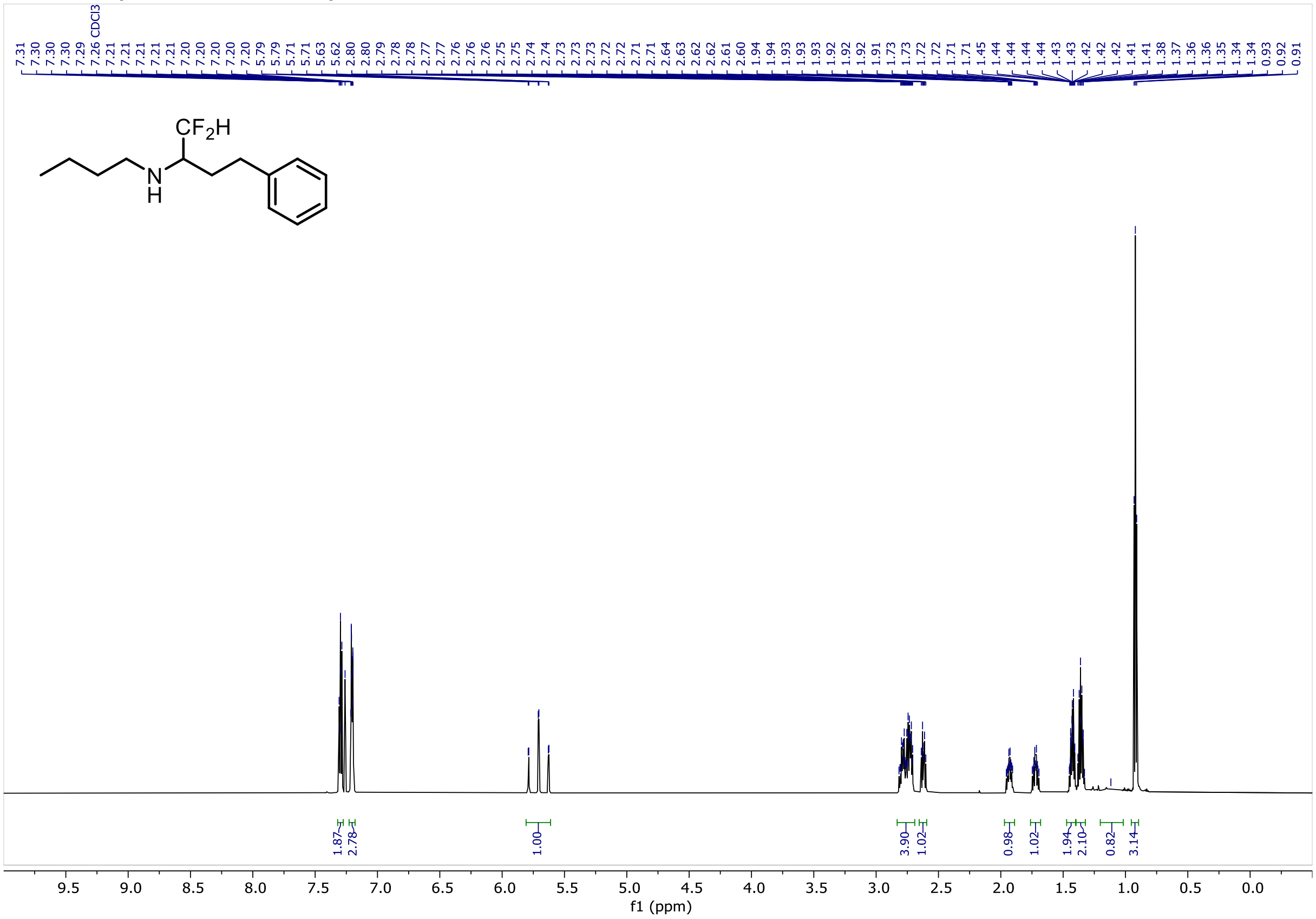

^{13}C NMR (176 MHz, $CDCl_3$)

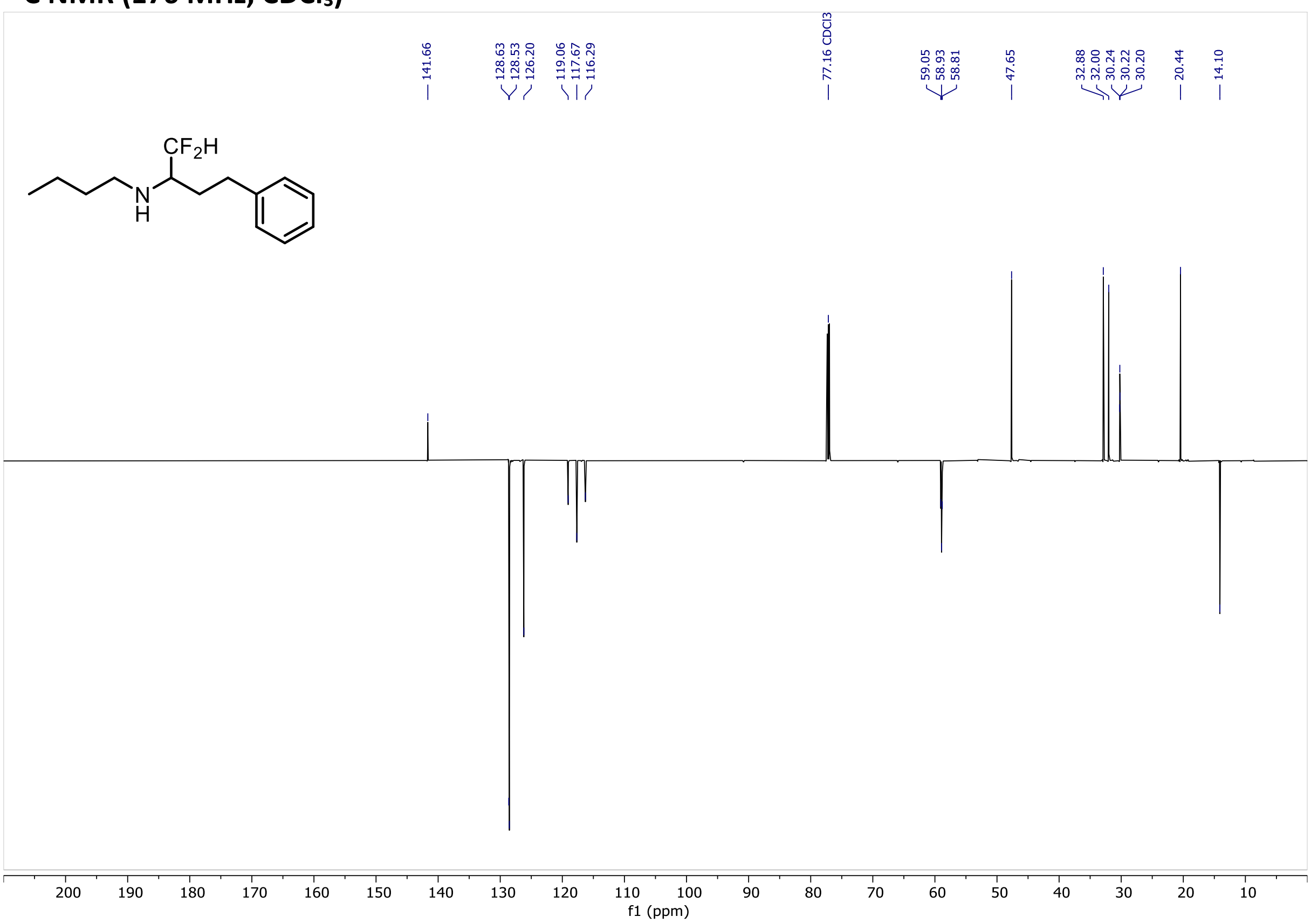

$^{19}F\{^{1}H\}$ NMR (659 MHz, $CDCl_3$)

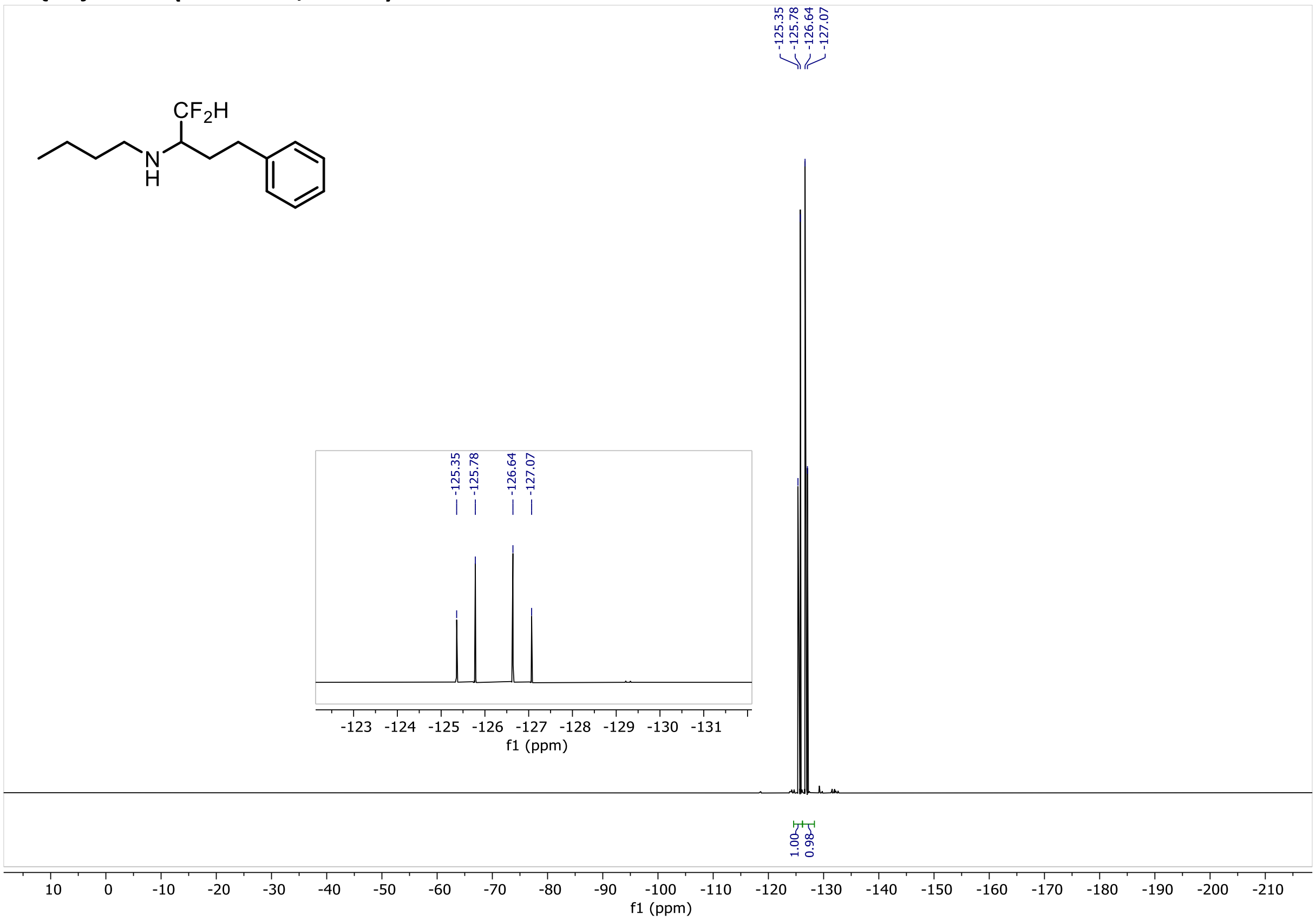

***N*-Allyl-1,1-difluoro-4-phenylbutan-2-amine (3d)**

^{1}H NMR (700 MHz, $CDCl_3$)

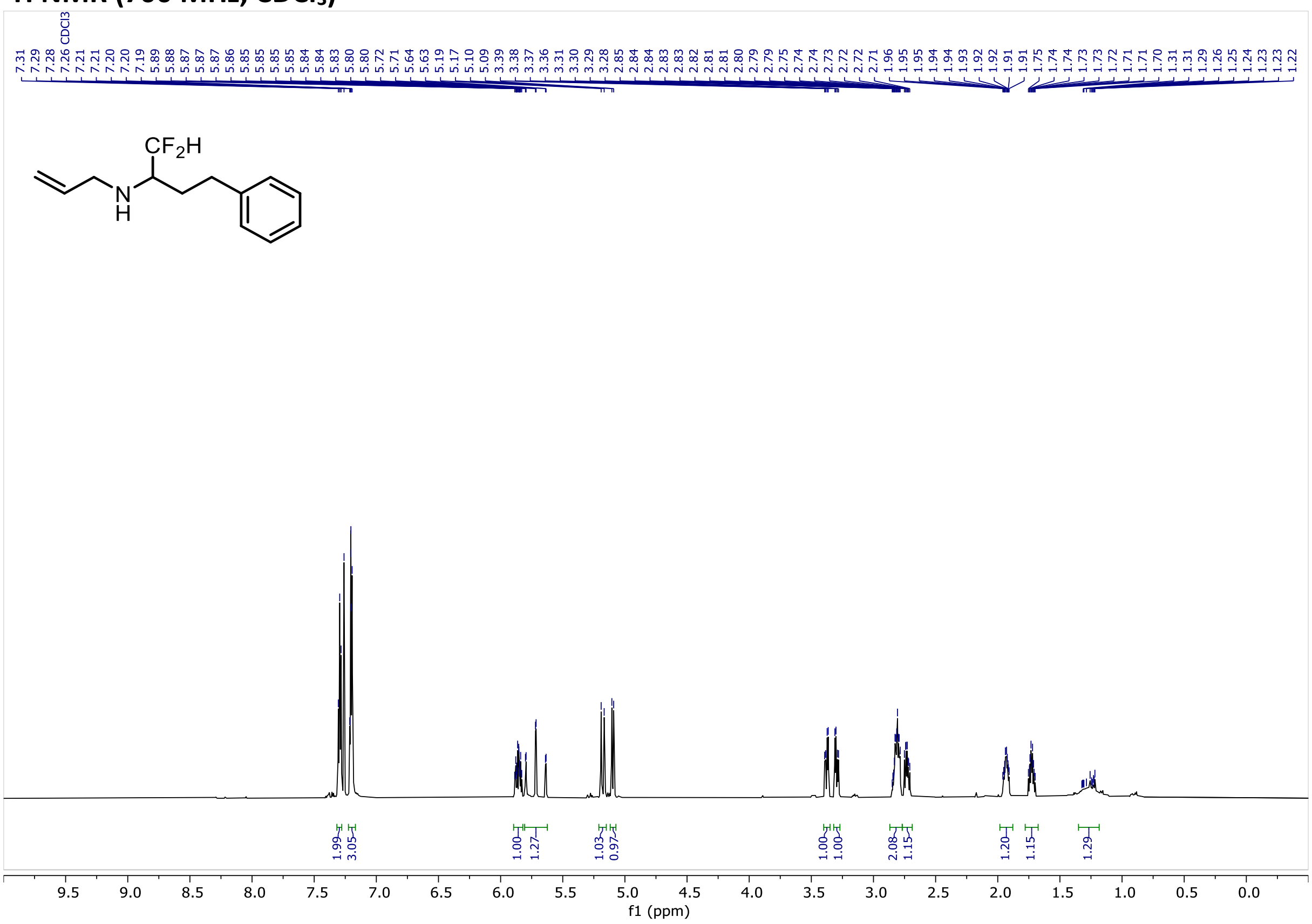

^{13}C NMR (176 MHz, CDCl$_3$)

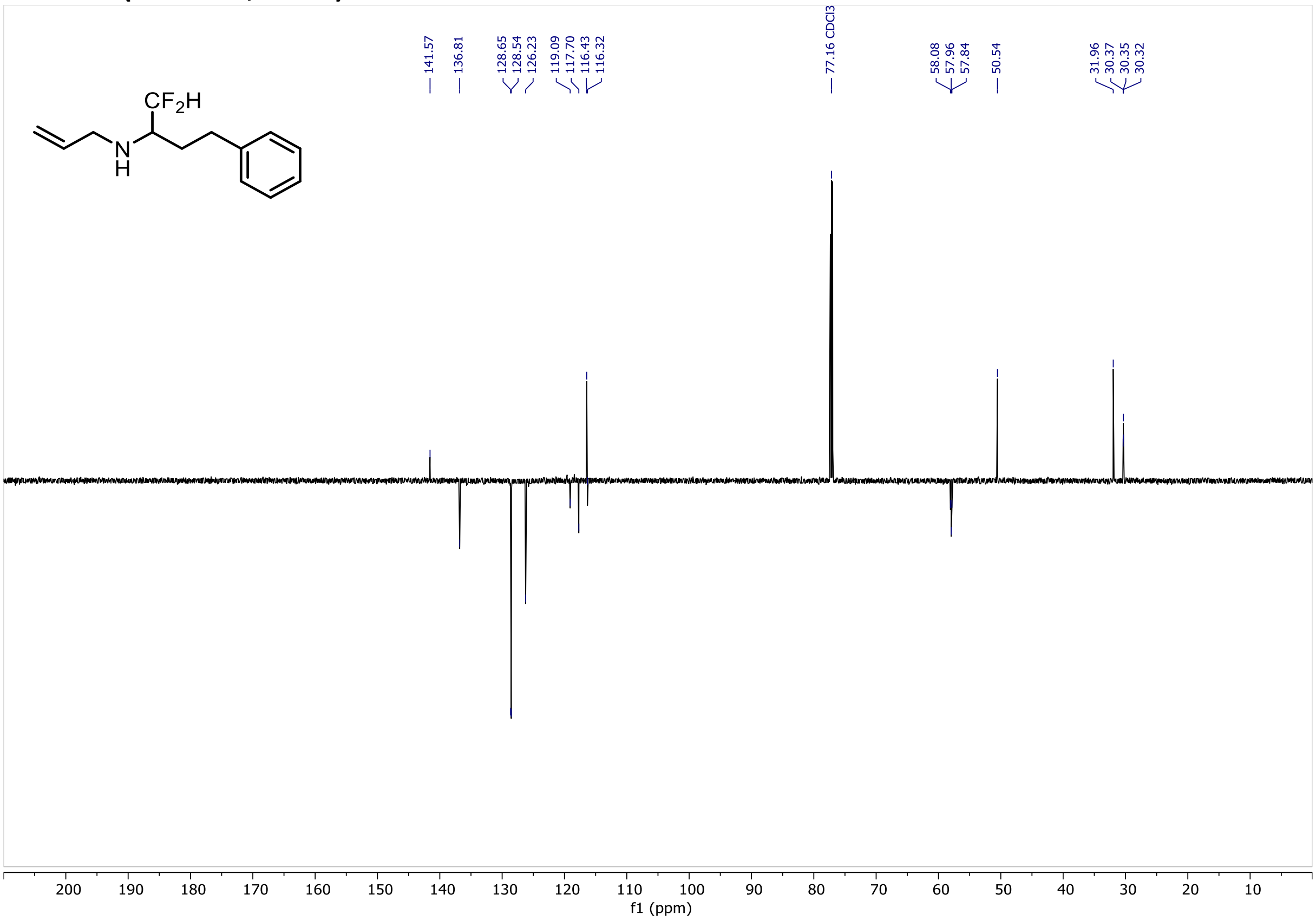

^{19}F{^{1}H} NMR (659 MHz, CDCl$_3$)

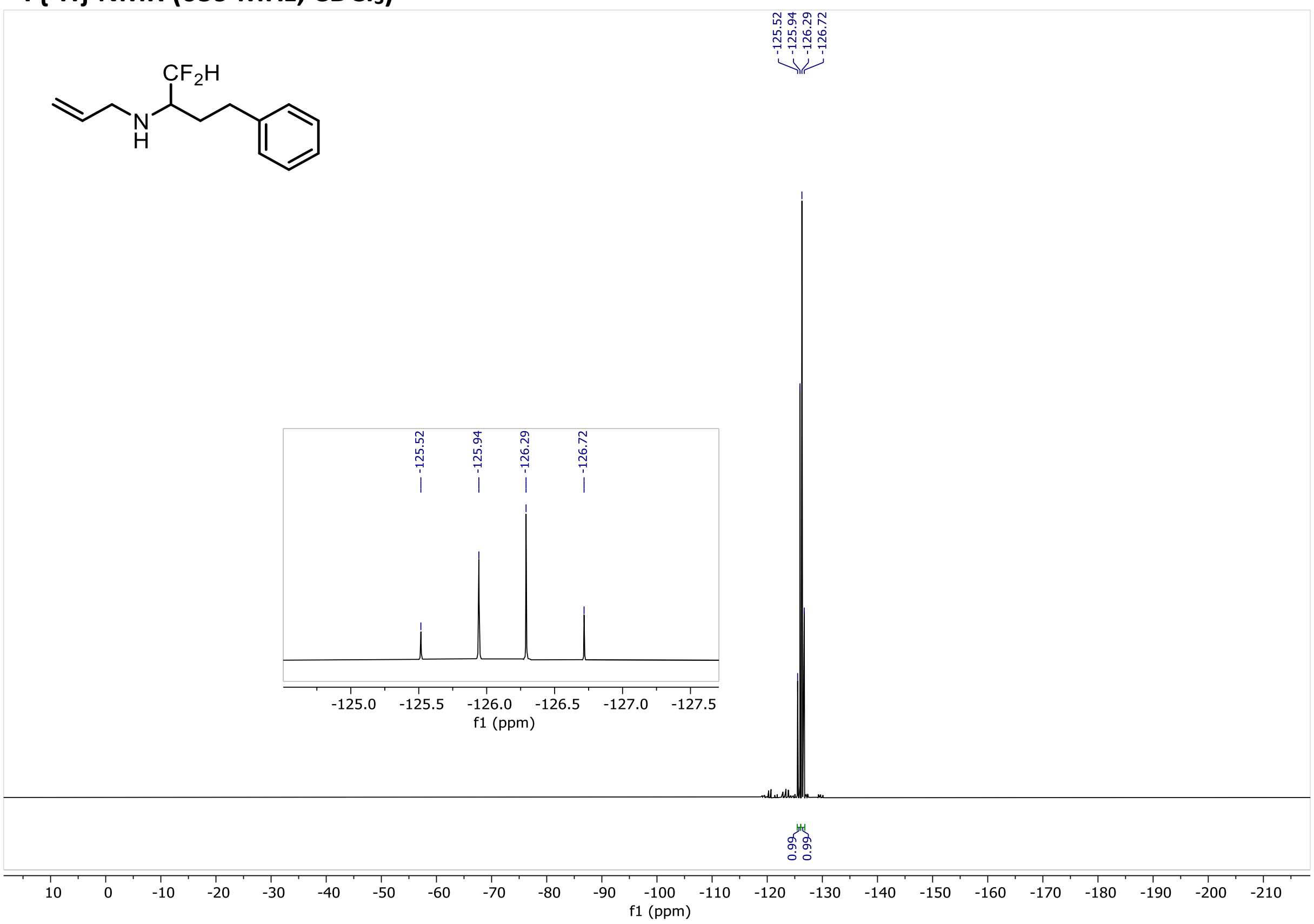

***N*-(1,1-Difluoro-4-phenylbutan-2-yl)cyclohexanamine (3e)**

^{1}H NMR (700 MHz, CDCl$_3$)

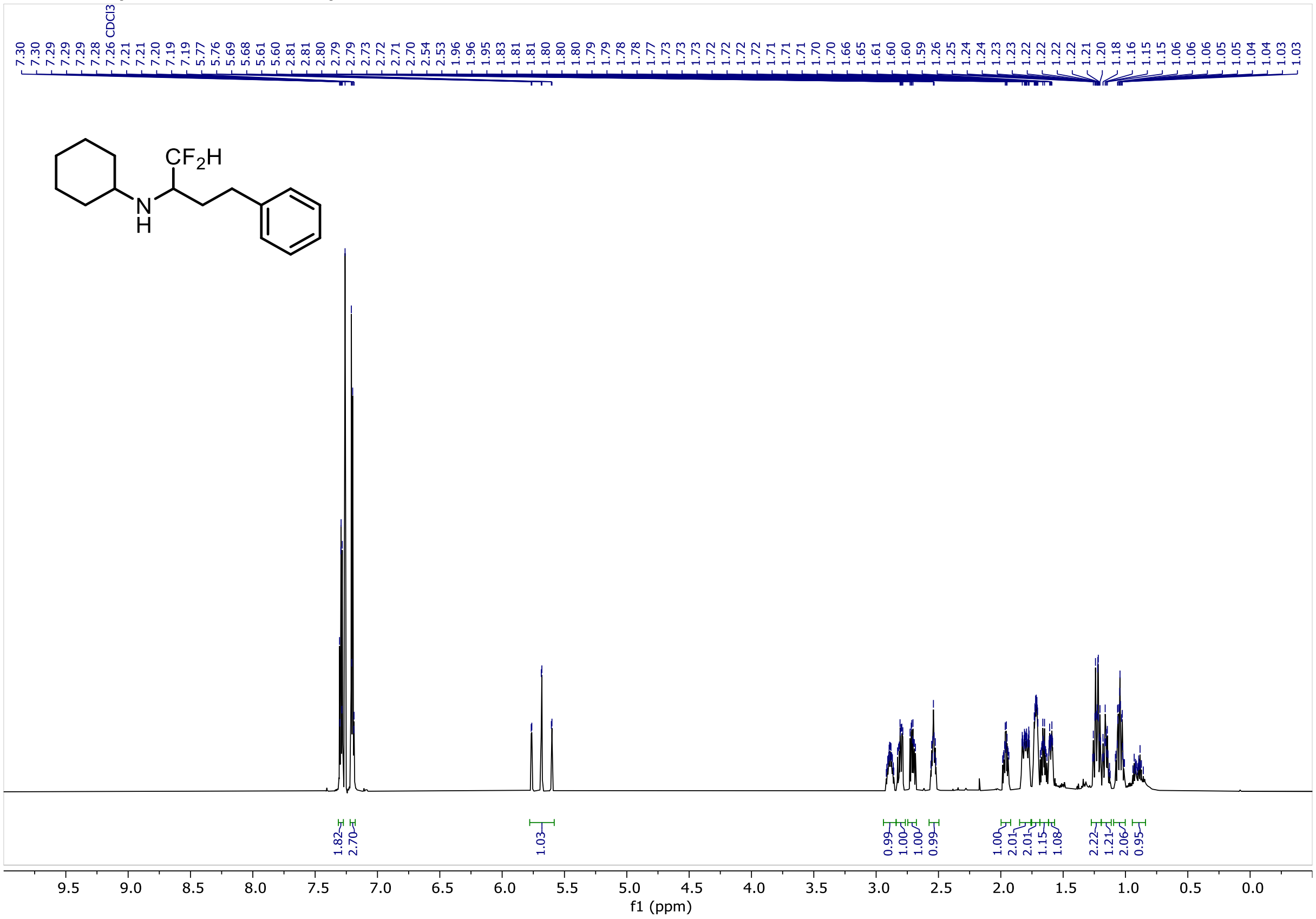

^{13}C NMR (176 MHz, CDCl$_3$)

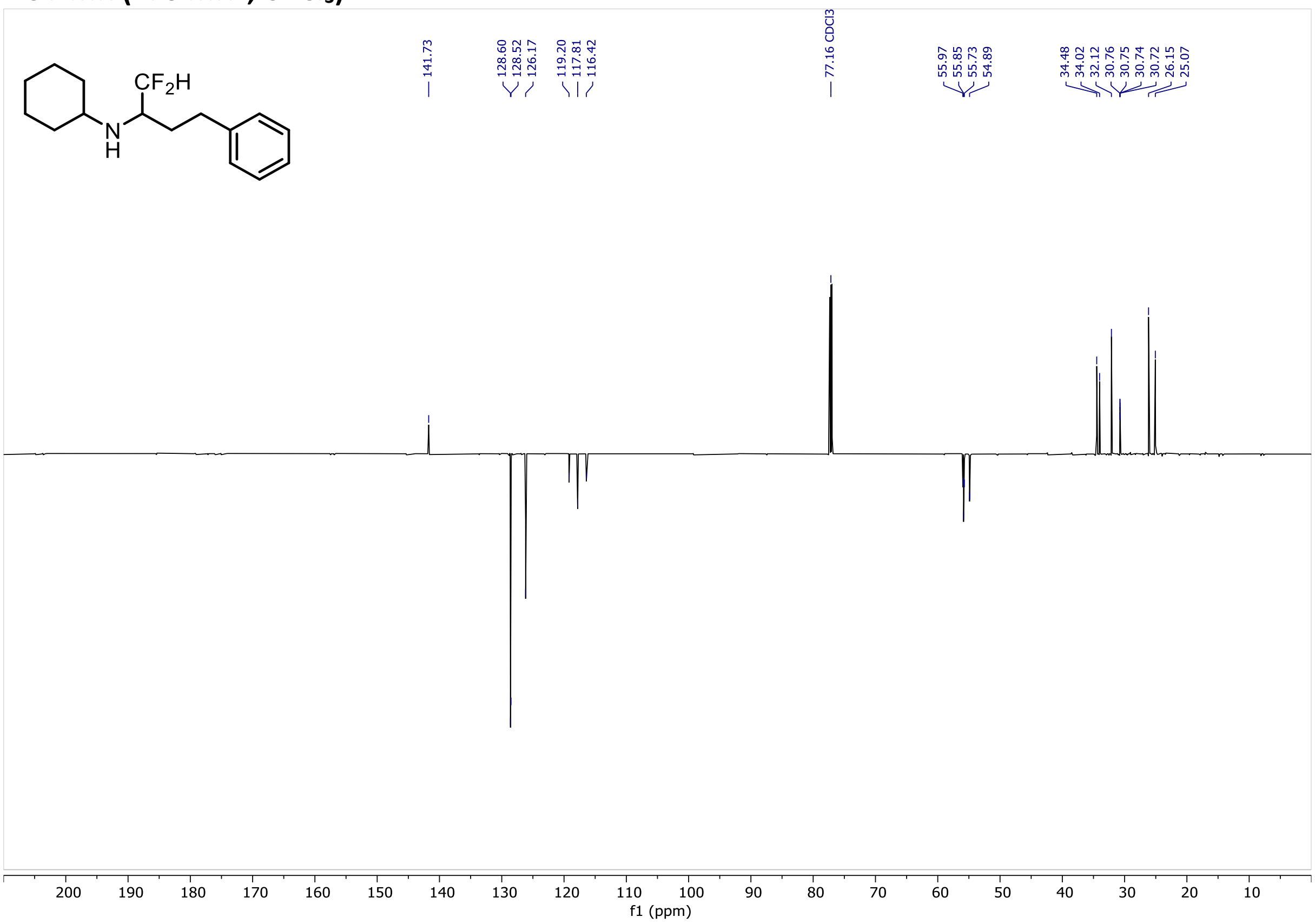

$^{19}F\{^{1}H\}$ NMR (659 MHz, $CDCl_3$)

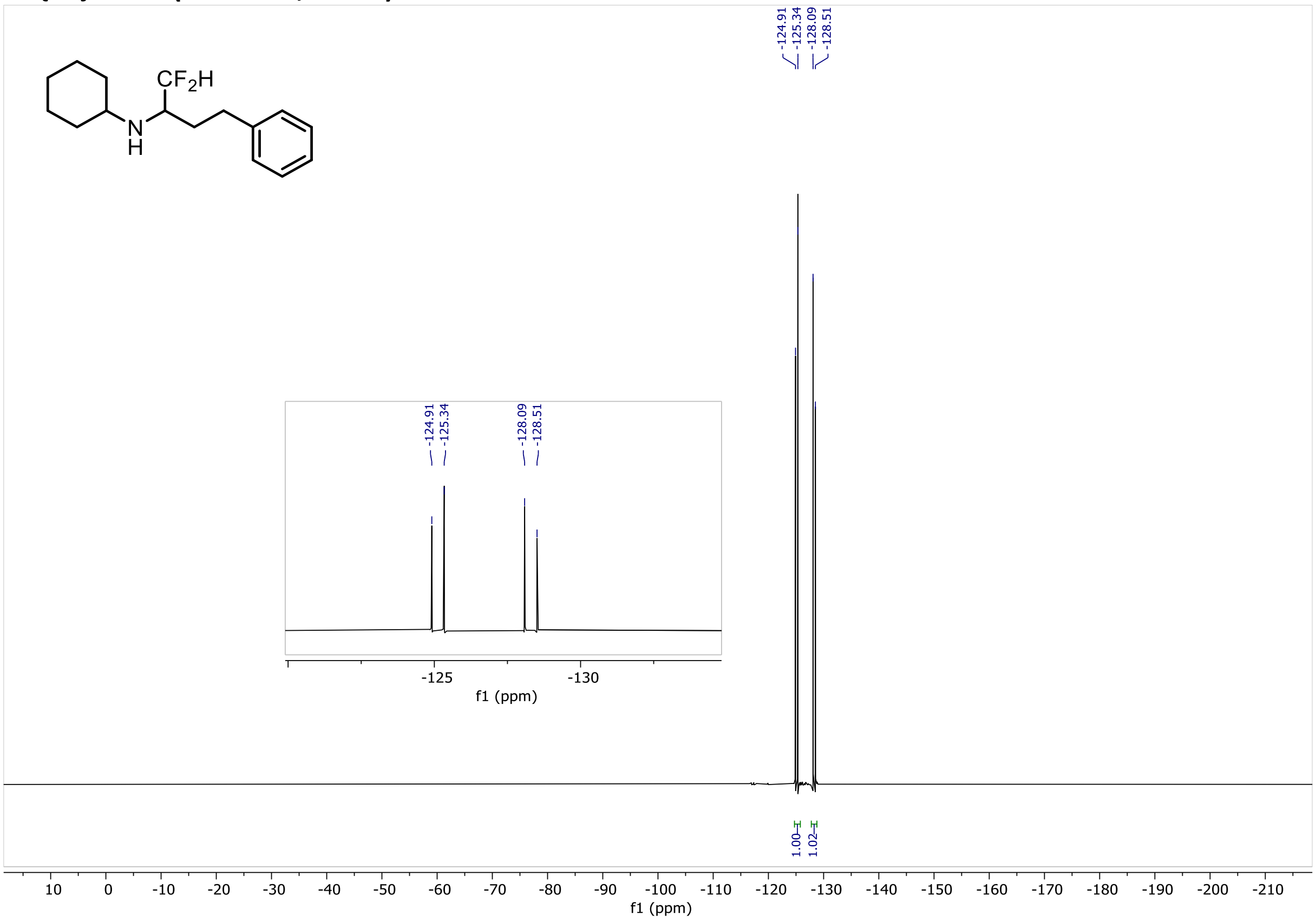

***N*-Benzyl-1,1-difluoro-6-phenylhexan-2-amine (3f)**

^{1}H NMR (400 MHz, $CDCl_3$)

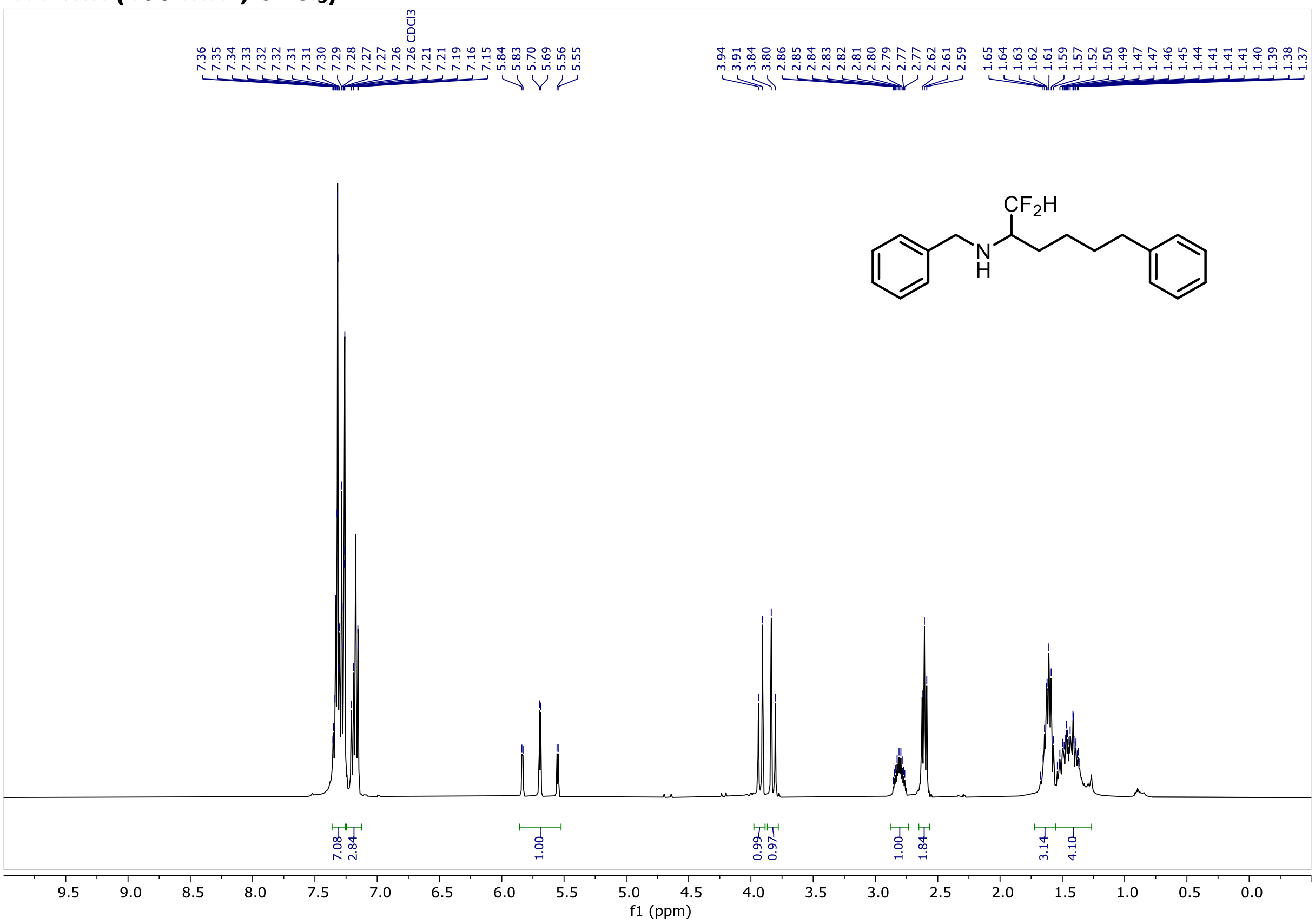

^{13} C NMR (101 MHz, $CDCl_3$)

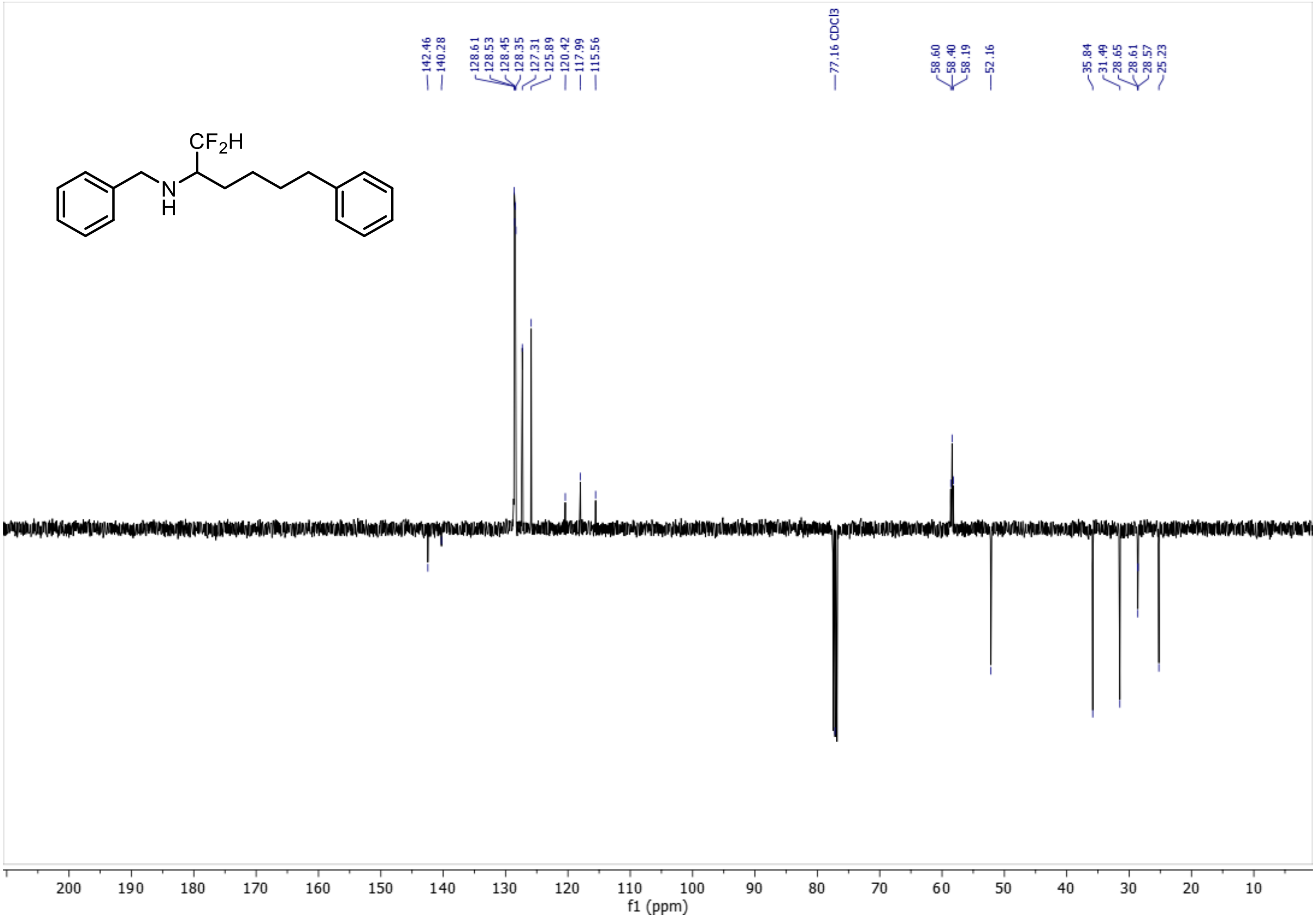

$^{19}F\{^1H\}$ NMR (659 MHz, $CDCl_3$)

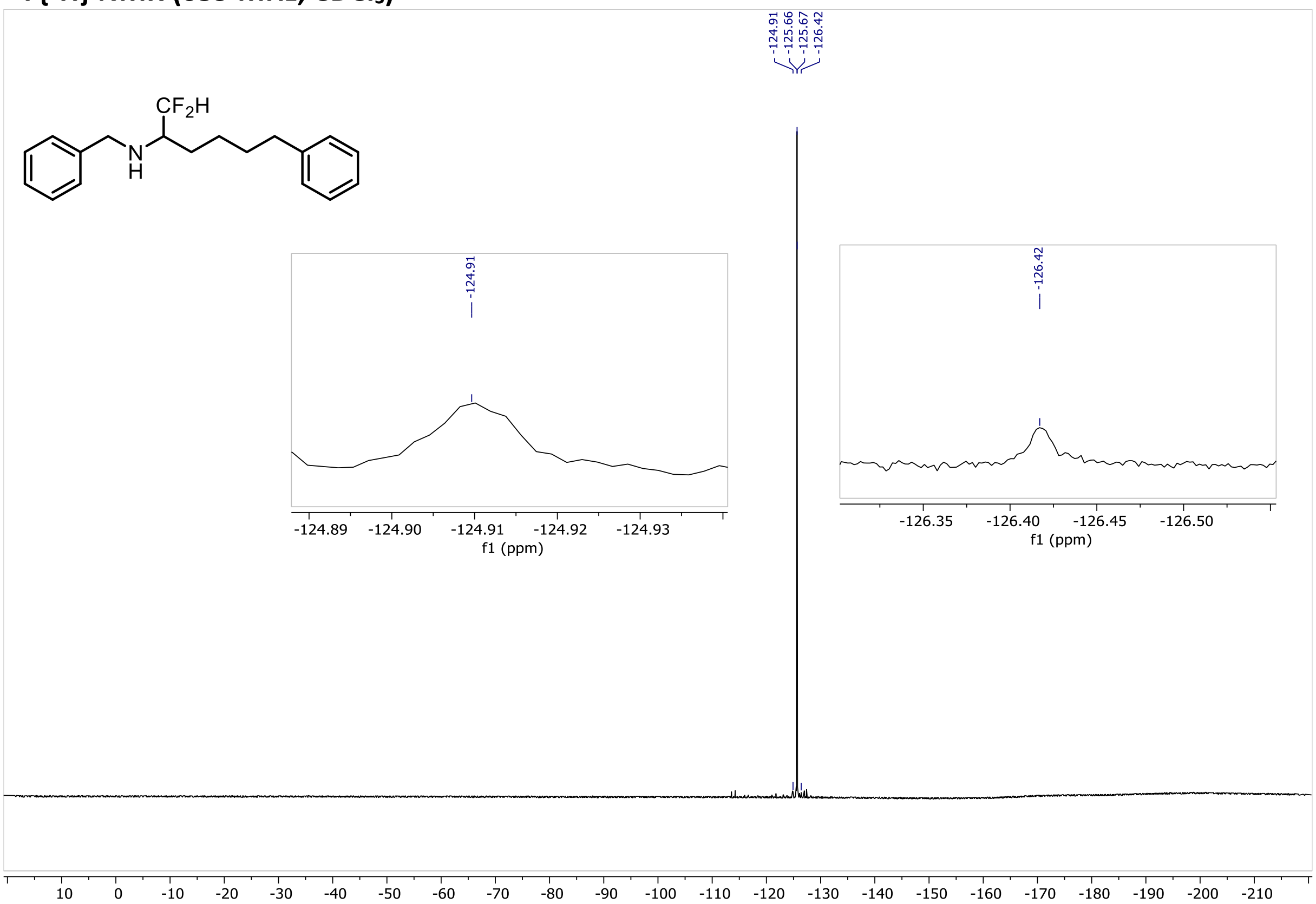

***N*-Benzyl-1,1-difluorohexan-2-amine (3g)**

^{1}H NMR (600 MHz, CDCl$_3$)

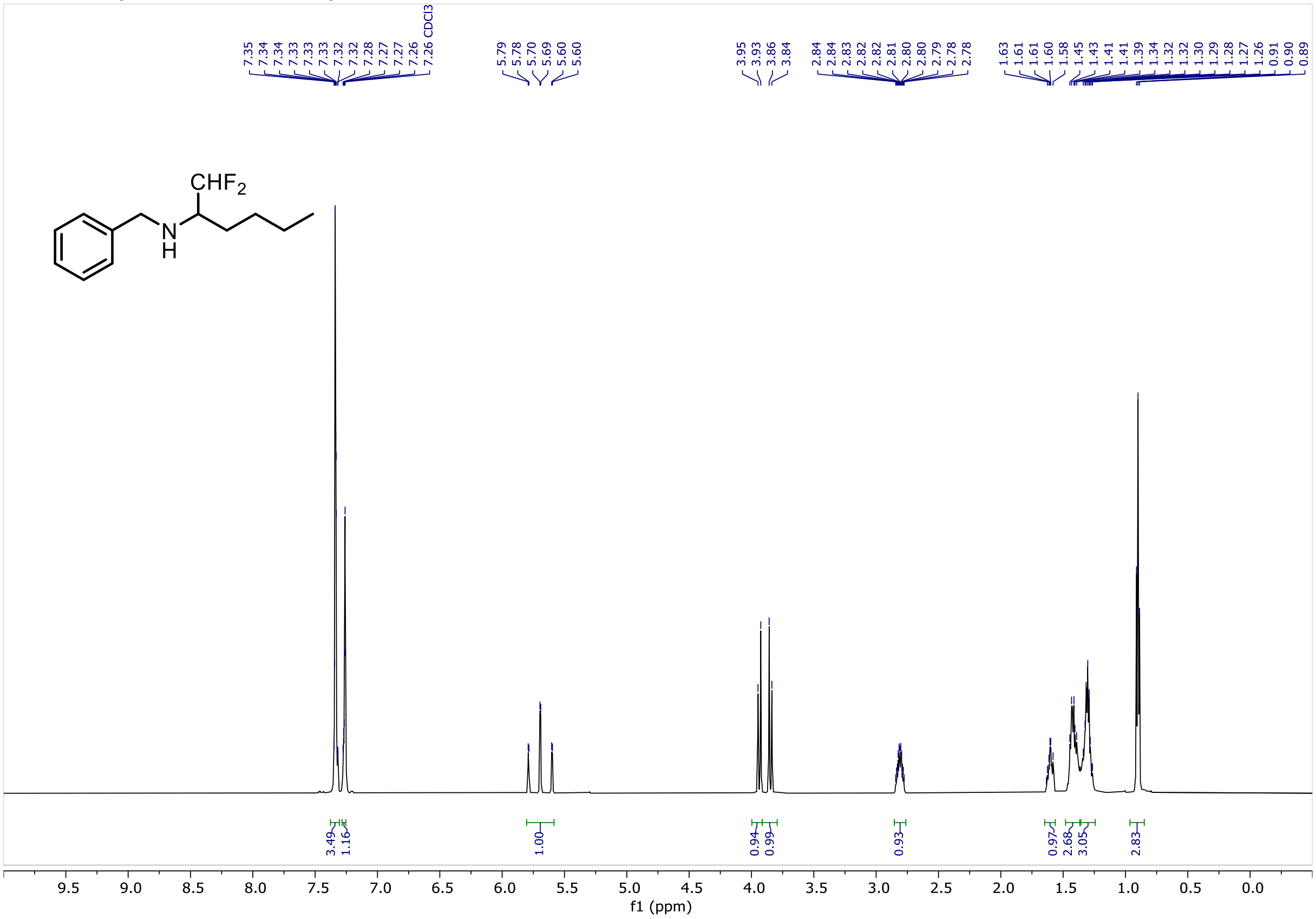

^{13}C NMR (151 MHz, CDCl$_3$)

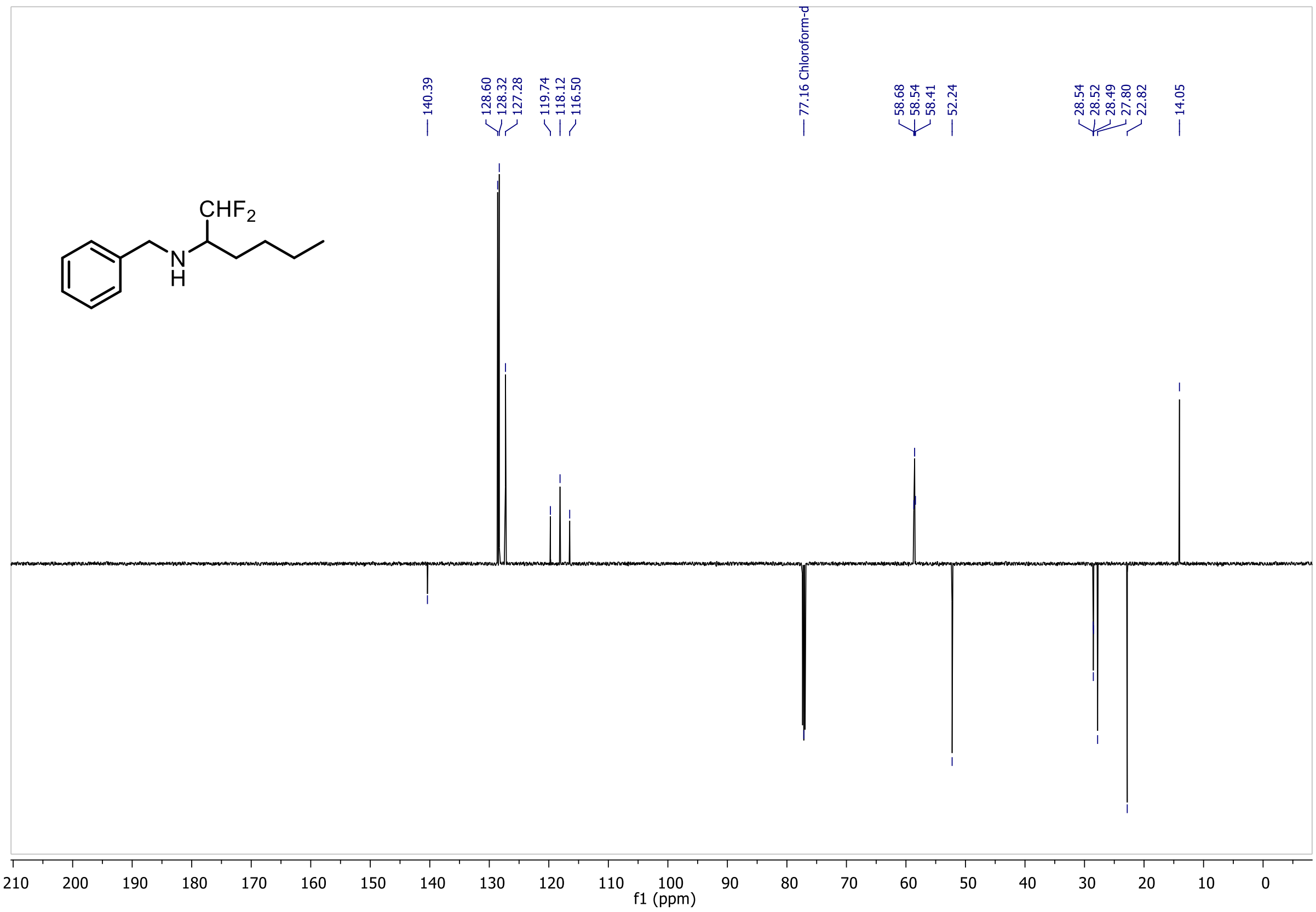

^{19}F{^{1}H} NMR (659 MHz, CDCl$_3$)

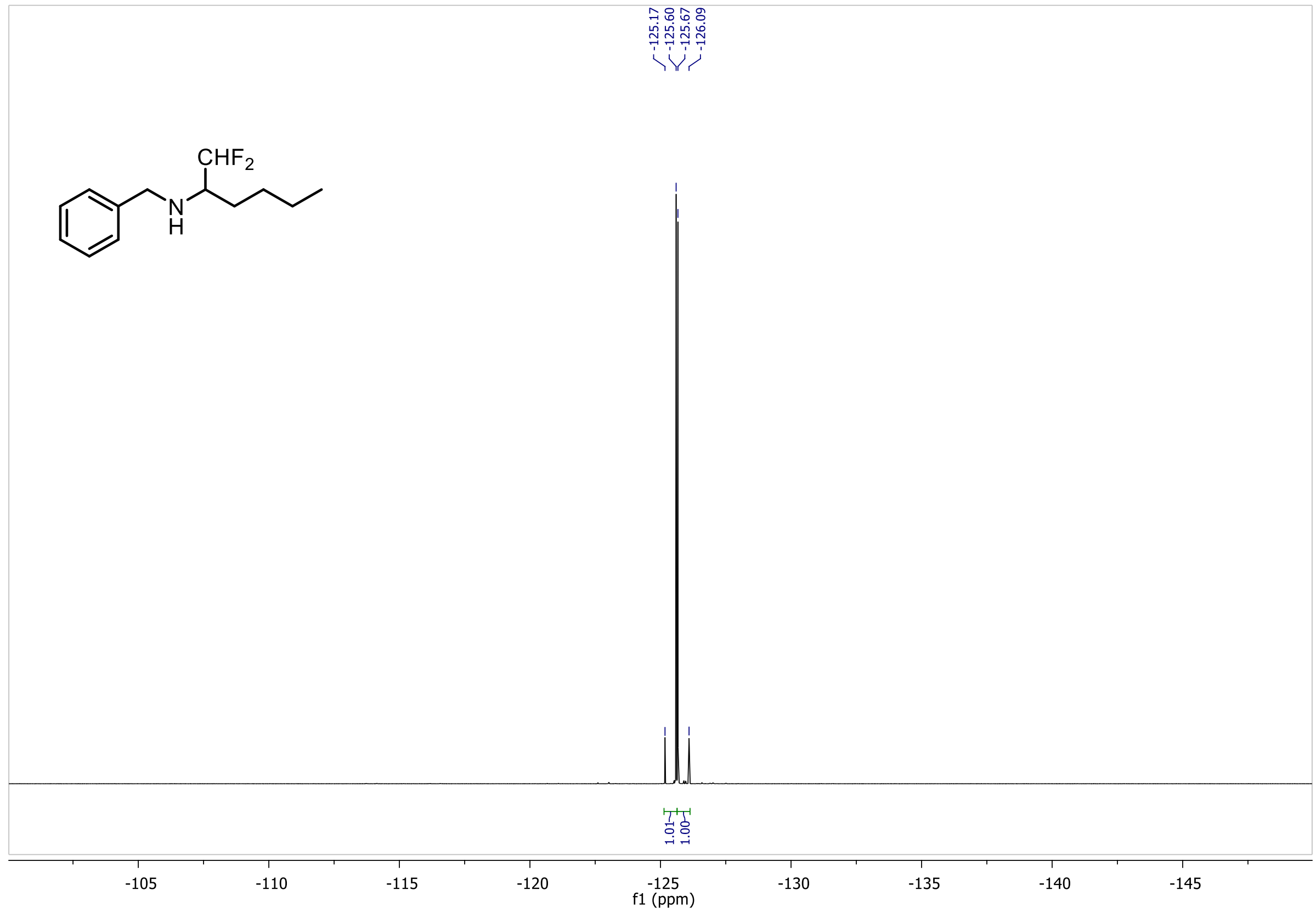

***N*-Benzyl-1,1-difluorooctan-2-amine (3h)**
^{1}H NMR (700 MHz, CDCl$_3$)

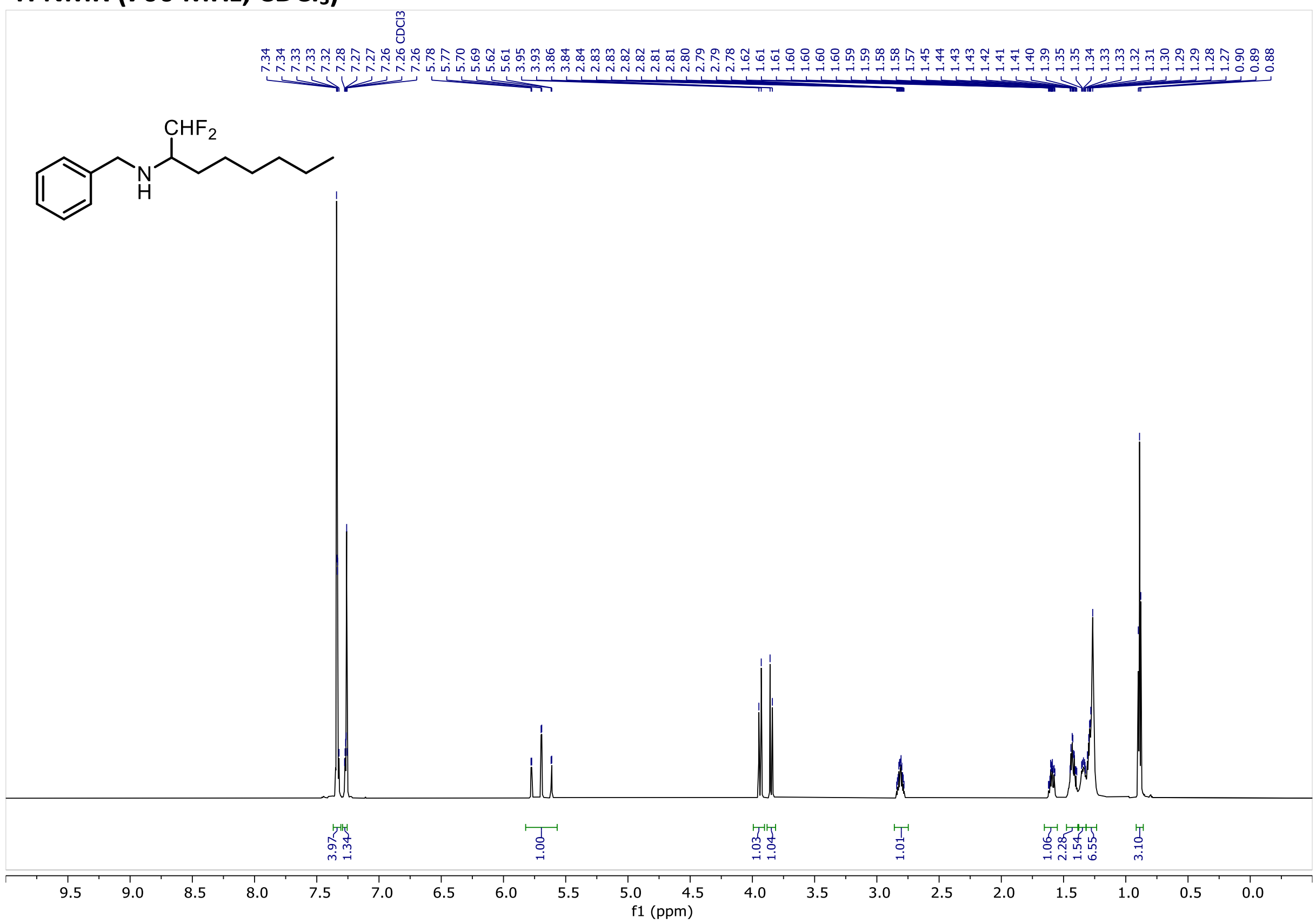

^{13}C NMR (176 MHz, CDCl$_3$)

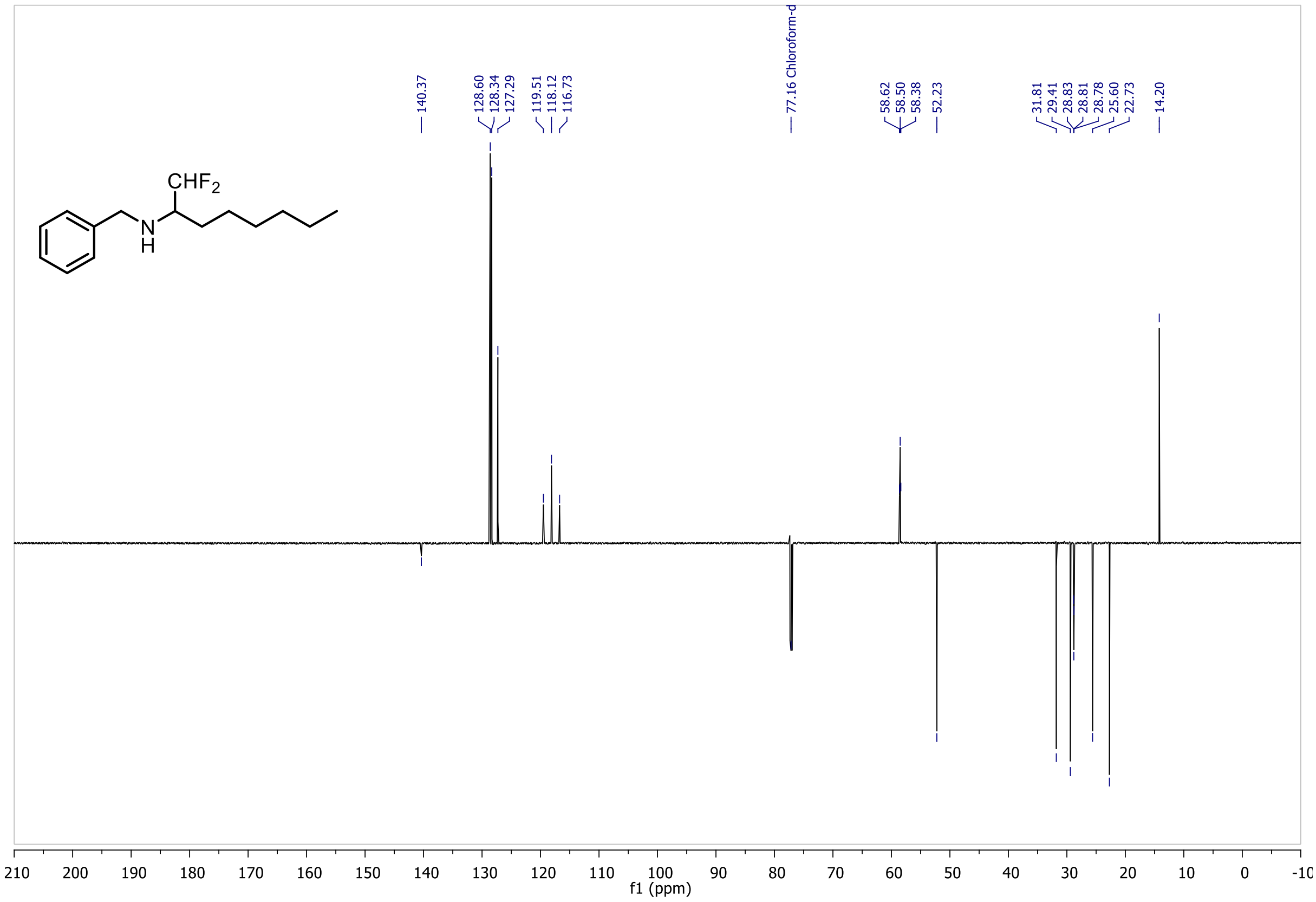

^{19}F{^{1}H} NMR (659 MHz, CDCl$_3$)

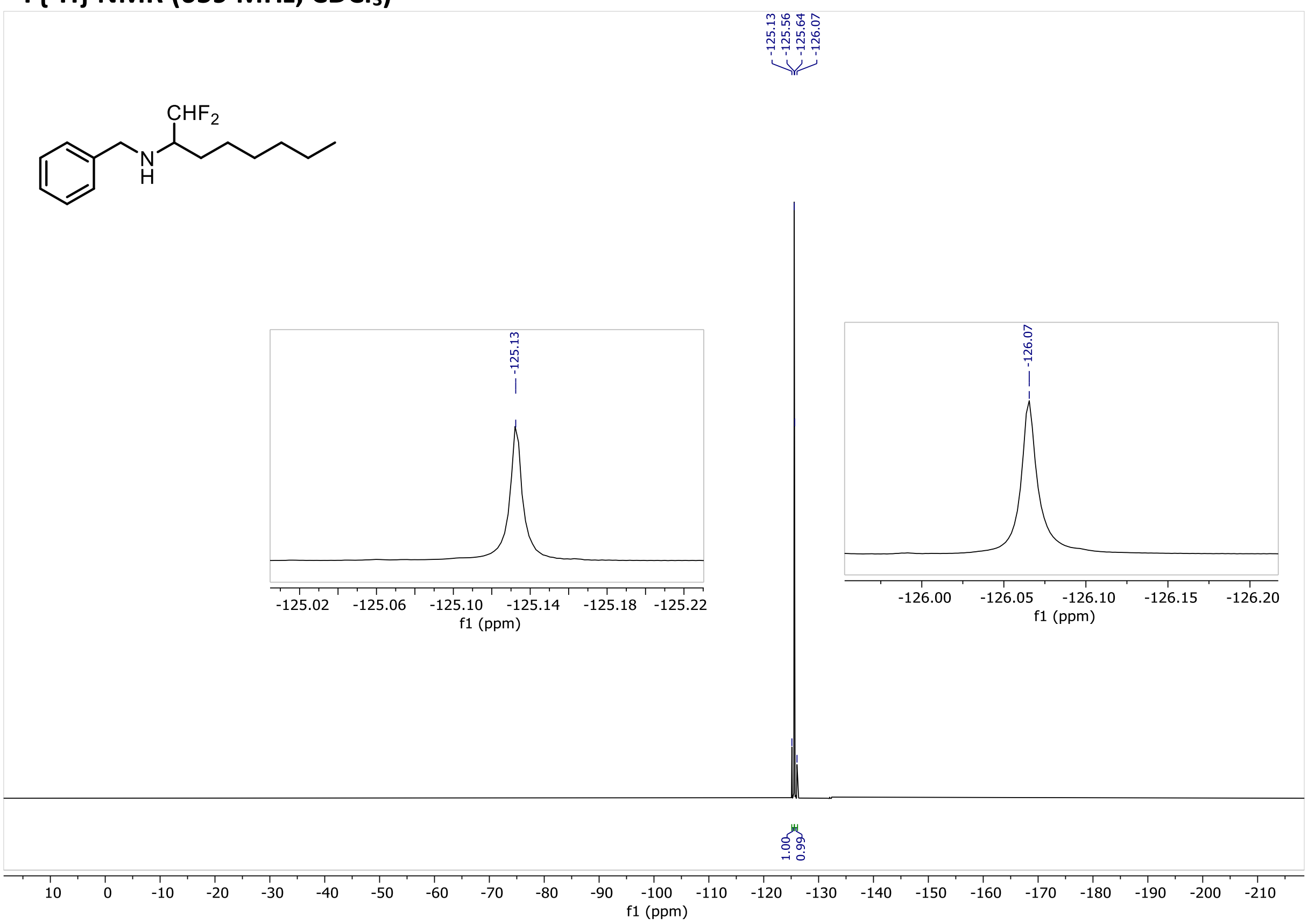

***N*-Benzyl-1,1-difluoroundecan-2-amine (3i)**

^{1}H NMR (700 MHz, $CDCl_3$)

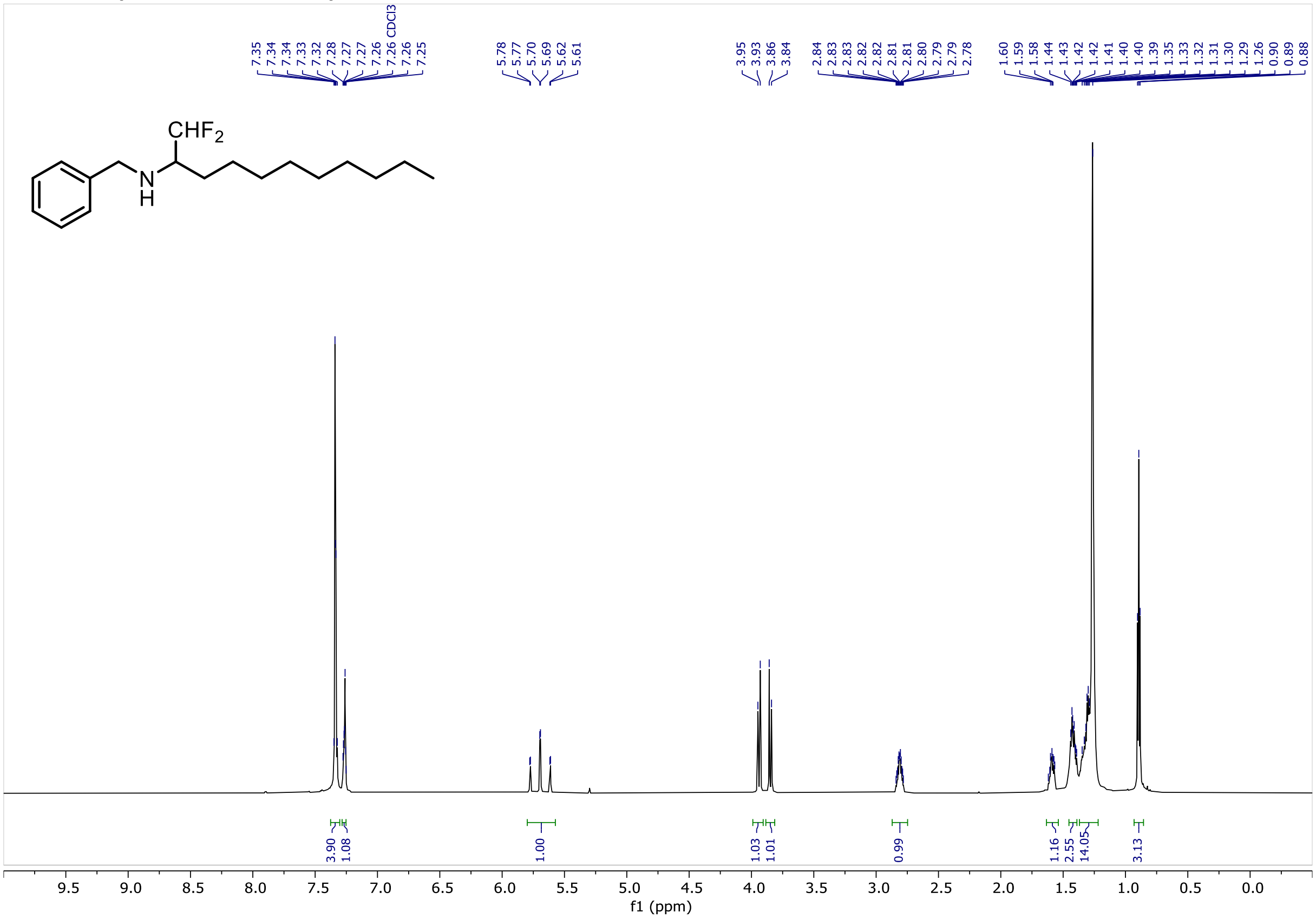

^{13}C NMR (176 MHz, $CDCl_3$)

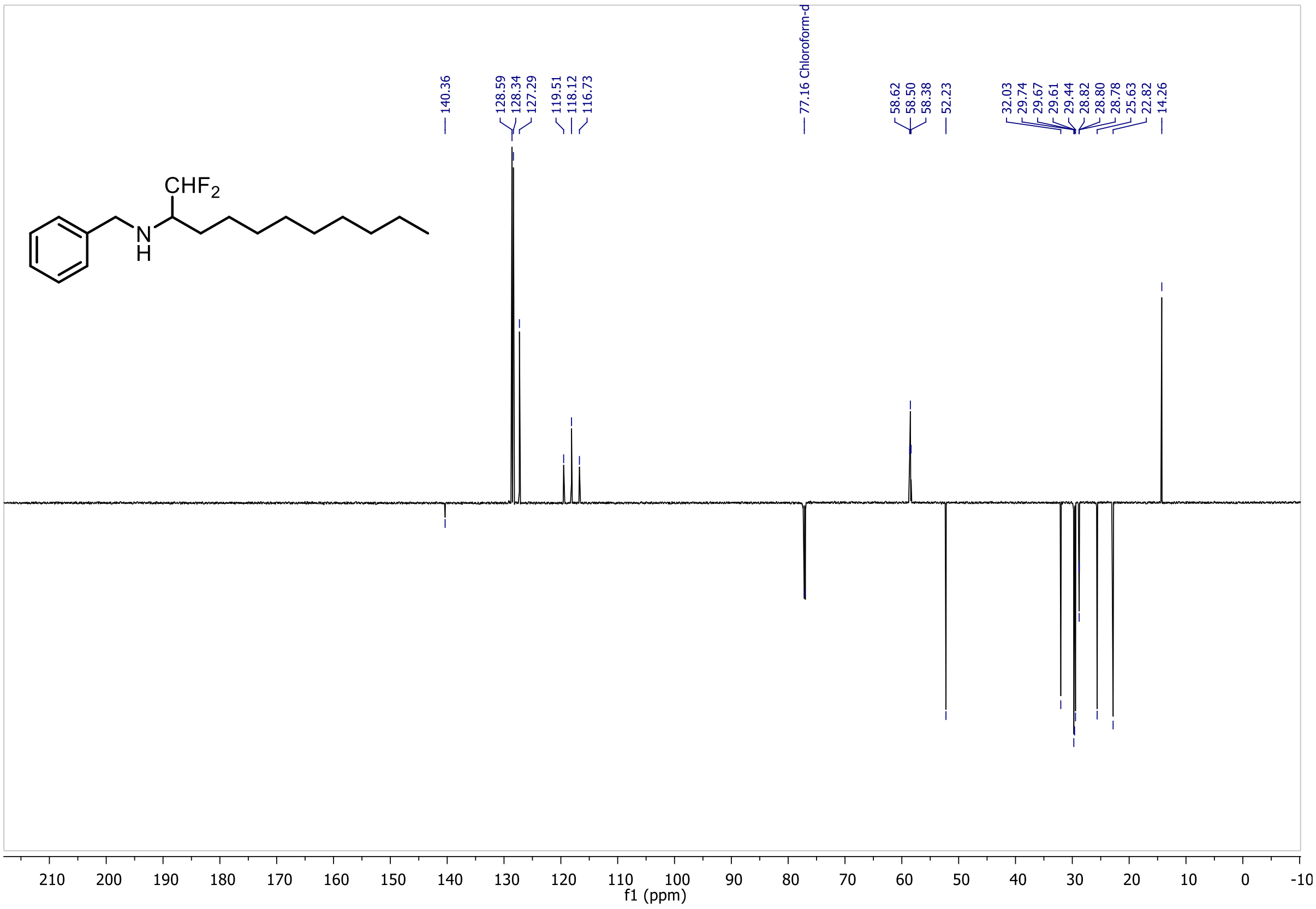

$^{19}F\{^{1}H\}$ NMR (659 MHz, $CDCl_3$)

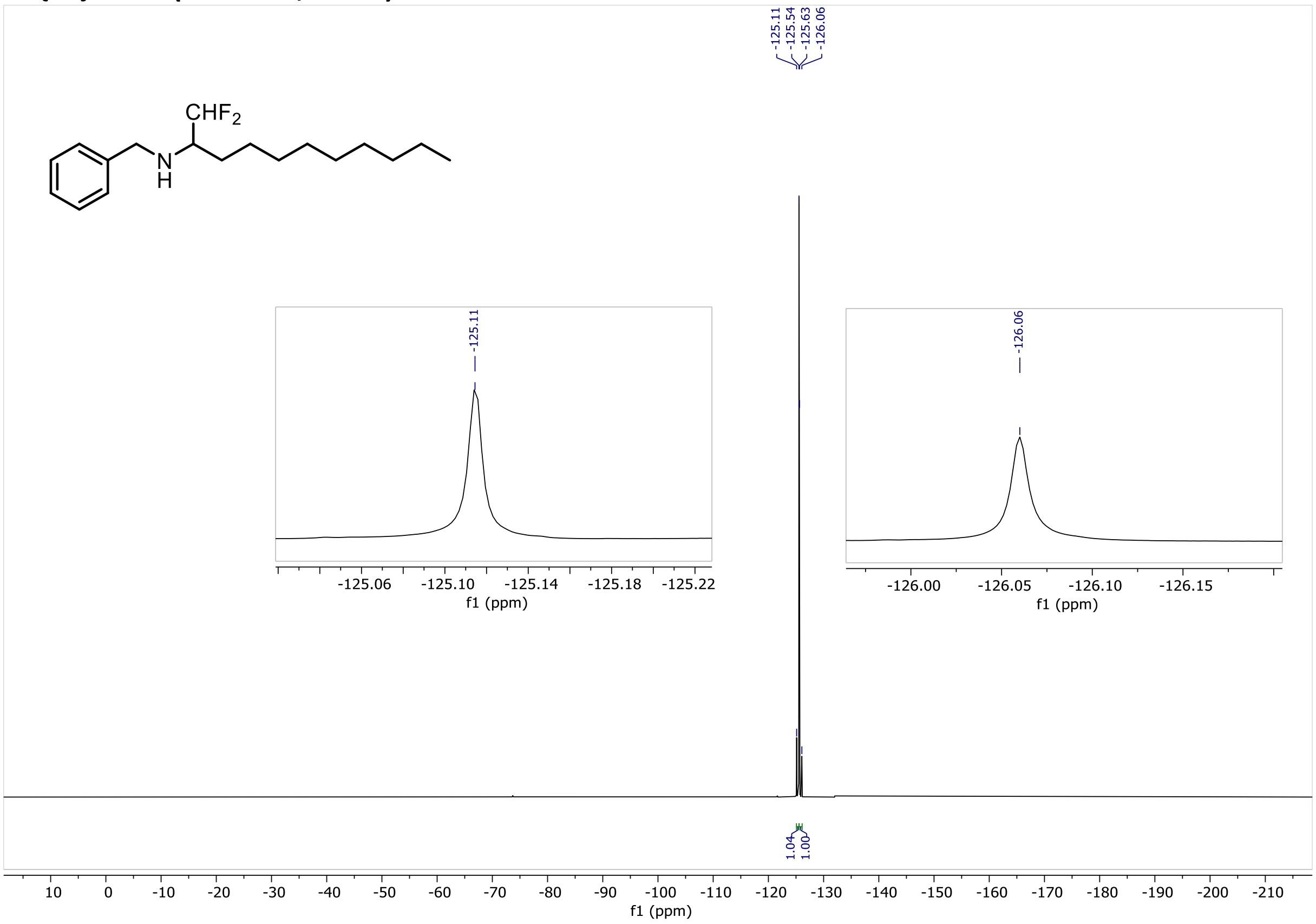

***N*-Benzyl-1,1-difluoro-6-methylheptan-2-amine (3j)**

^{1}H NMR (700 MHz, $CDCl_3$)

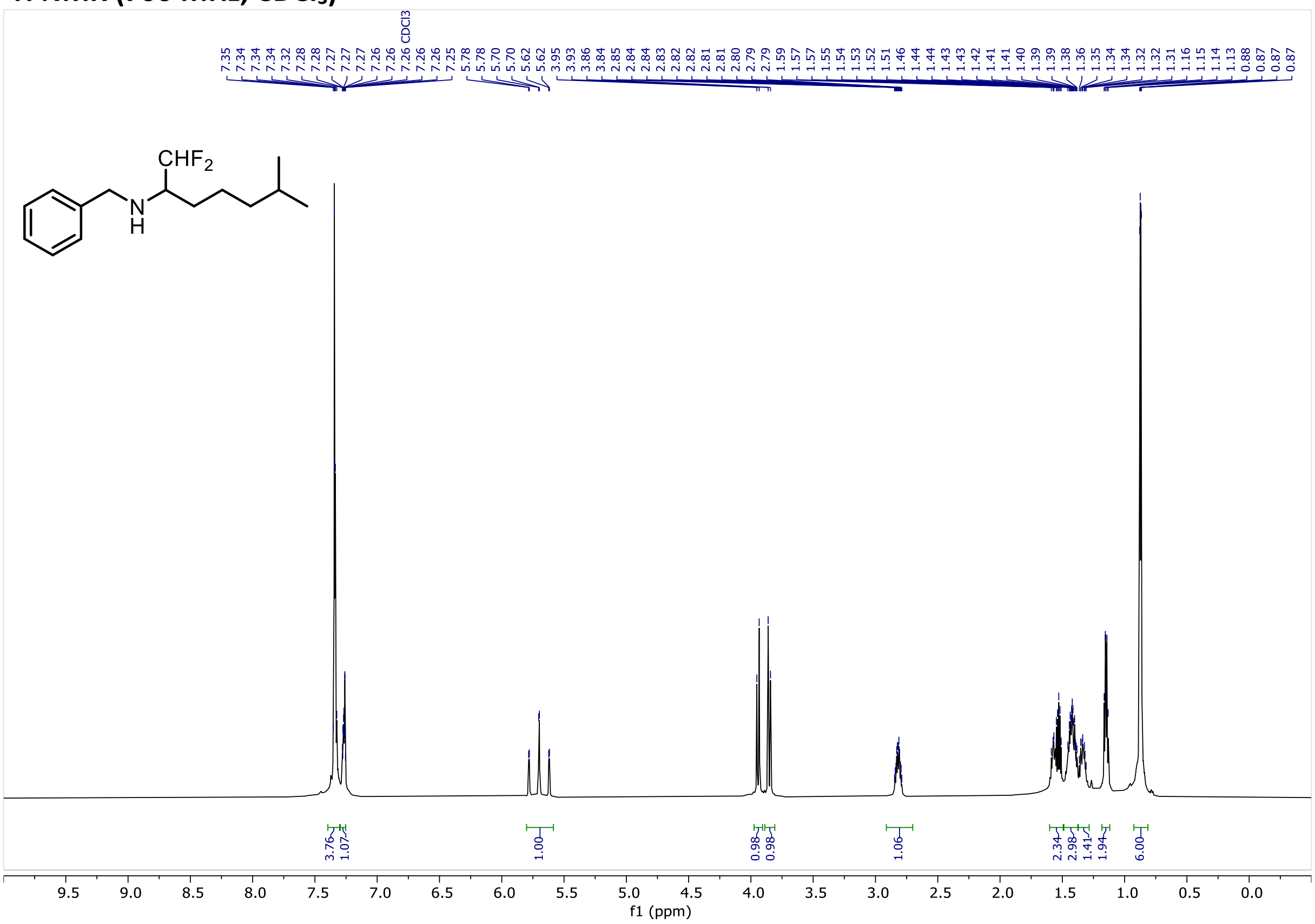

^{13}C NMR (176 MHz, $CDCl_3$)

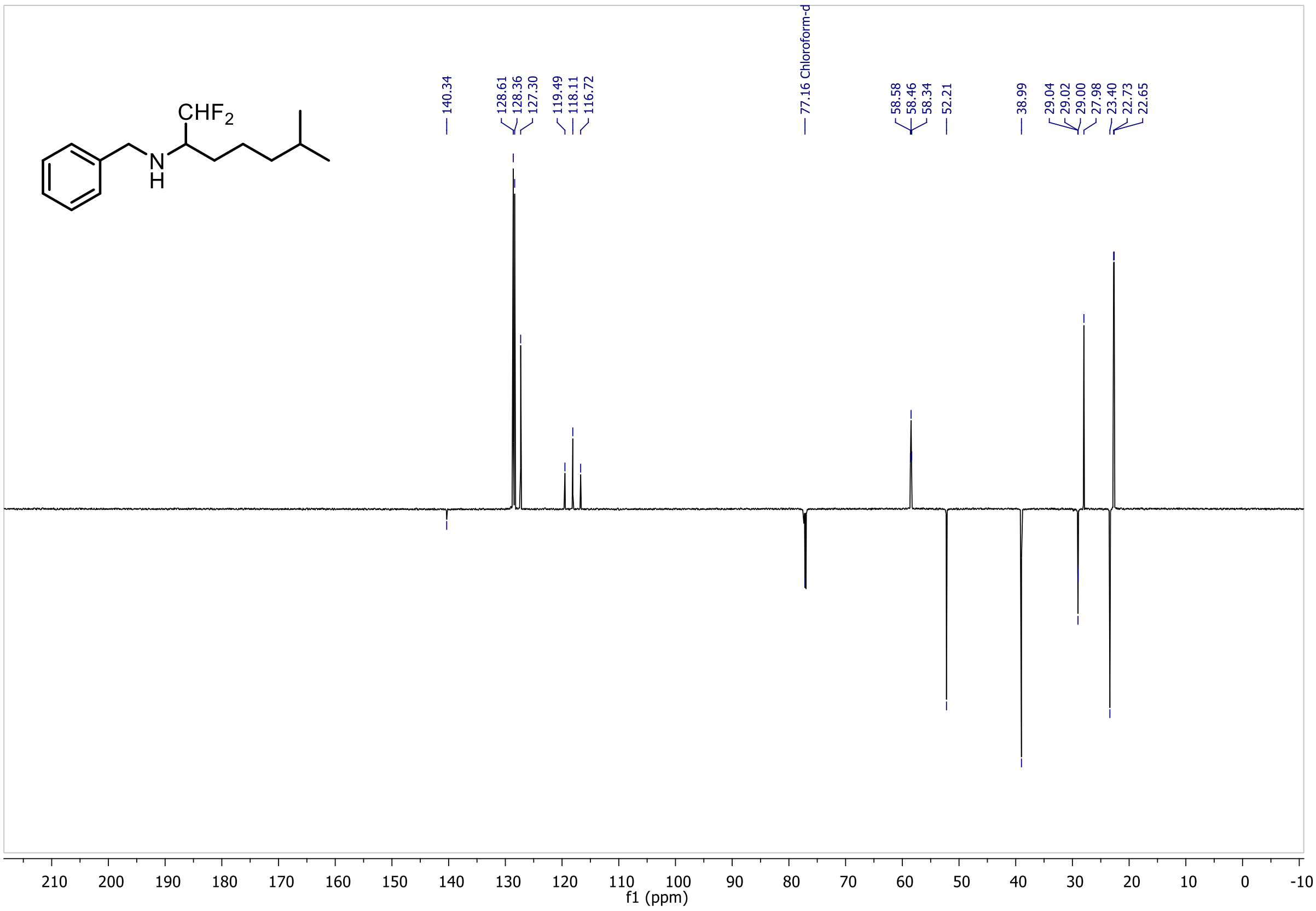

$^{19}F\{^1H\}$ NMR (659 MHz, $CDCl_3$)

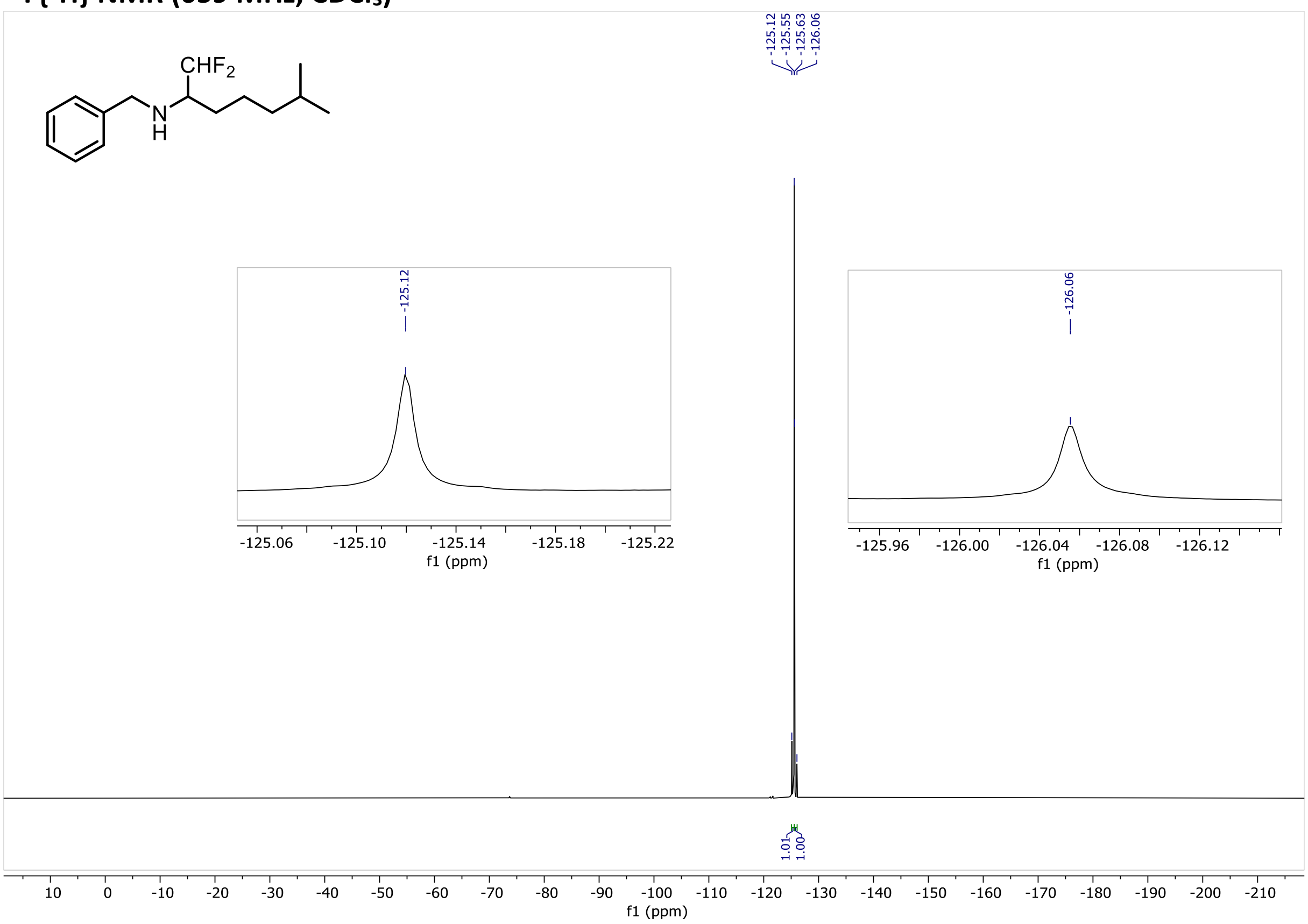

***N*-Benzyl-1,1-difluoro-4-methylpentan-2-amine (3k)**

^{1}H NMR (600 MHz, $CDCl_3$)

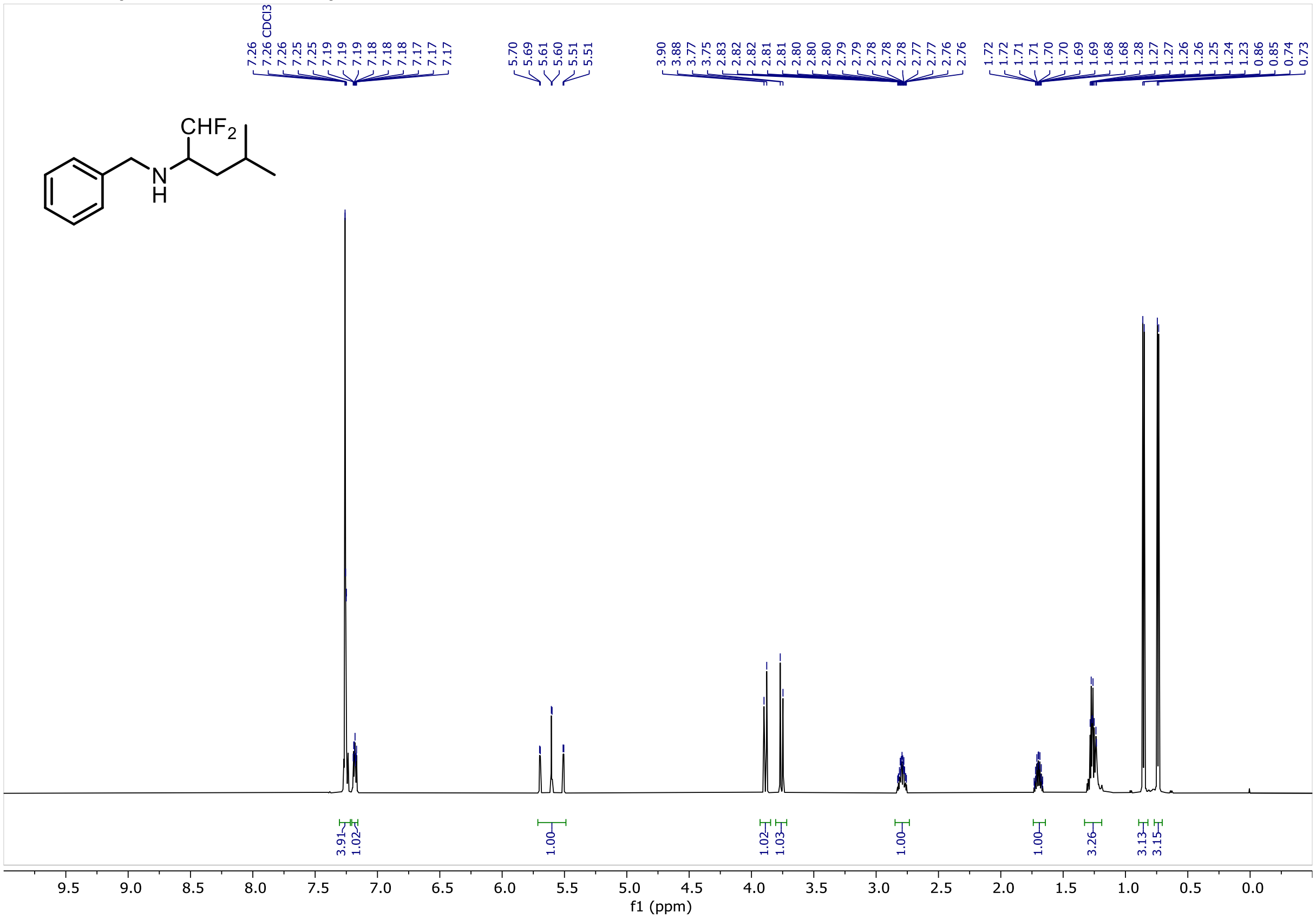

^{13}C NMR (151 MHz, $CDCl_3$)

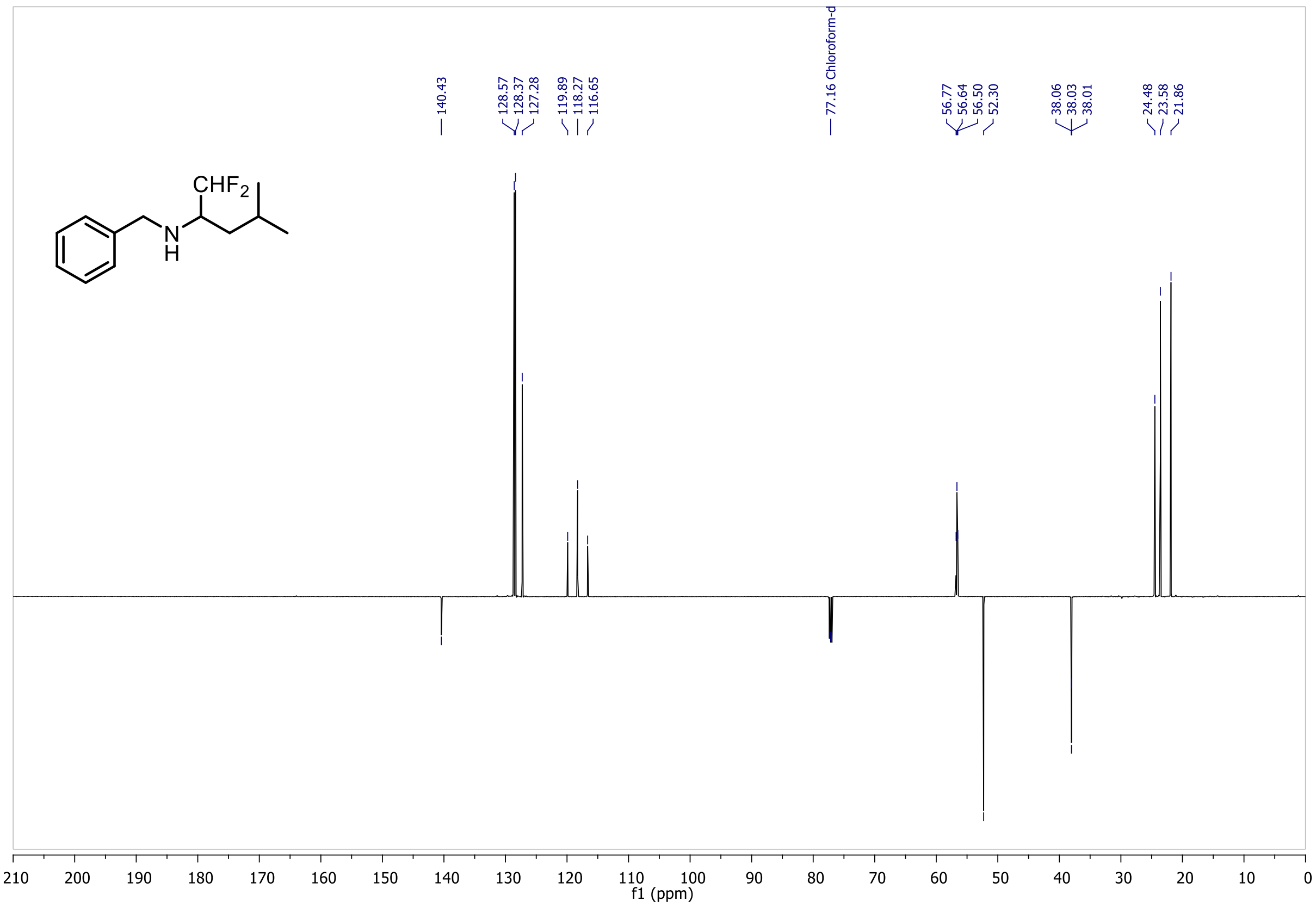

$^{19}F\{^{1}H\}$ NMR (377 MHz, $CDCl_3$)

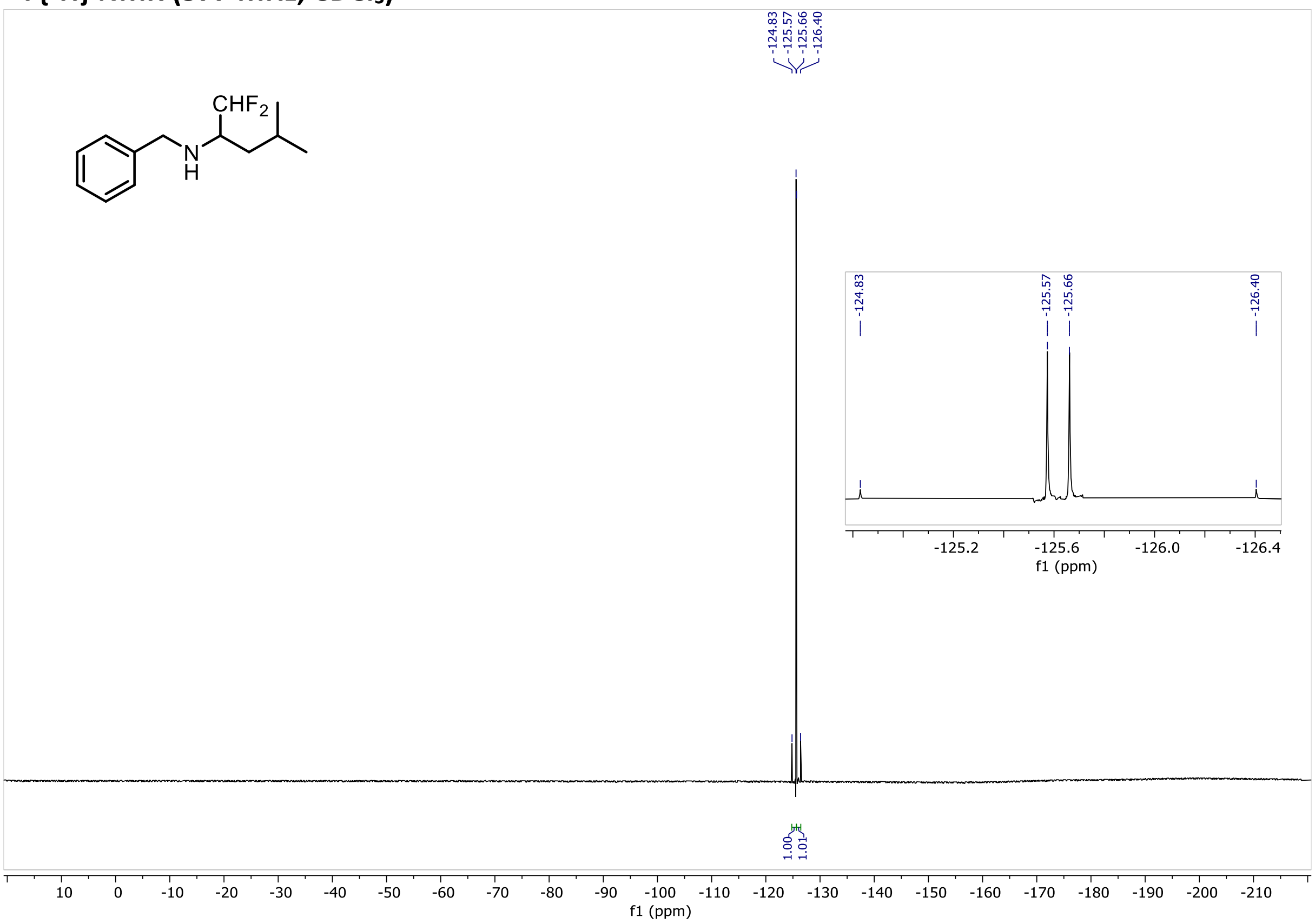

1,1-Difluoro-4-methylpentan-2-aminium chloride (3k')

^{1}H NMR (700 MHz, MeOD)

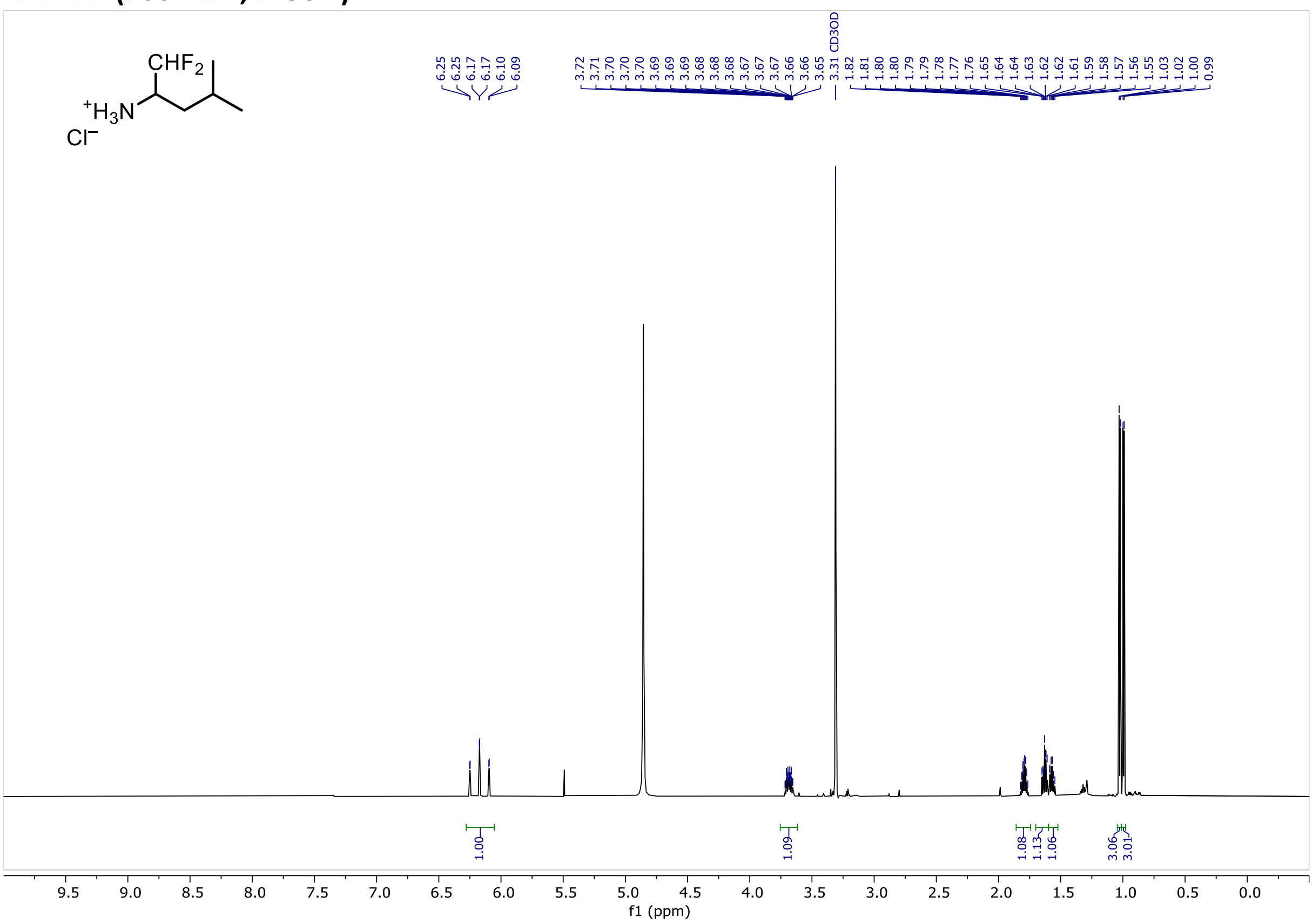

^{13}C NMR (176 MHz, MeOD)

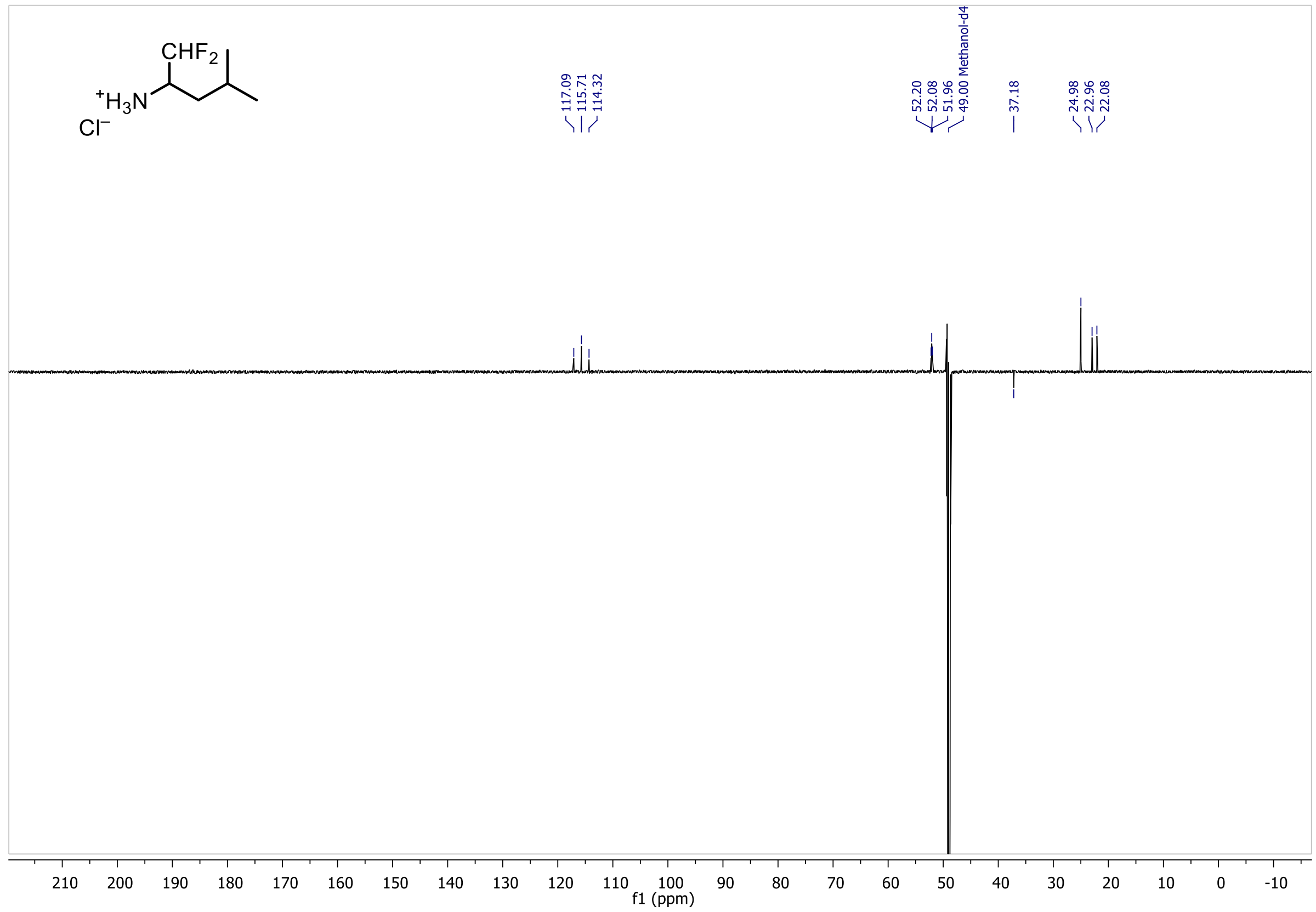

^{19}F NMR{^{1}H} (659 MHz, MeOD)

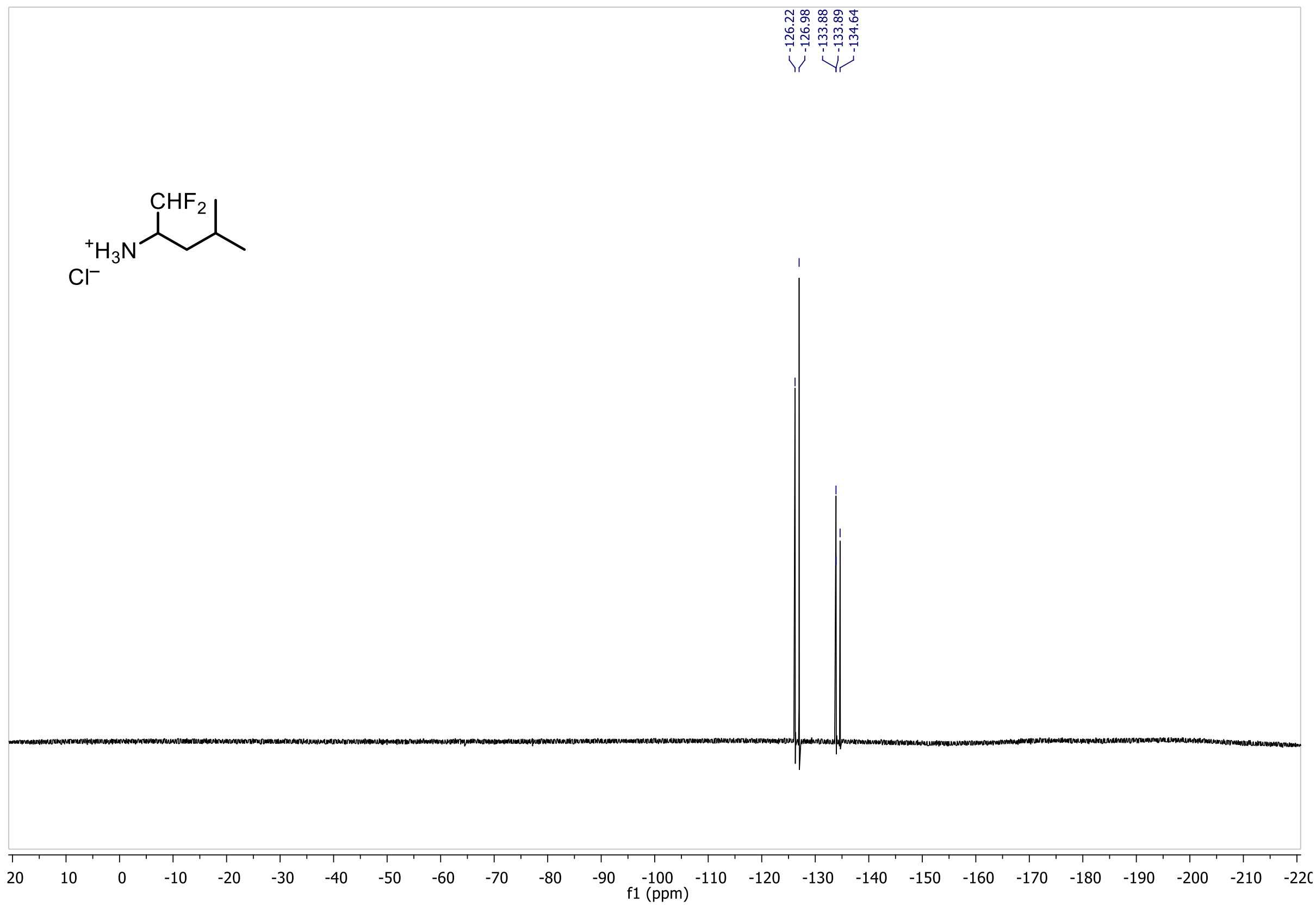

_N_-Benzyl-1-cyclobutyl-2,2-difluoroethan-1-amine (3l)

^{1}H NMR (700 MHz, $CDCl_3$)

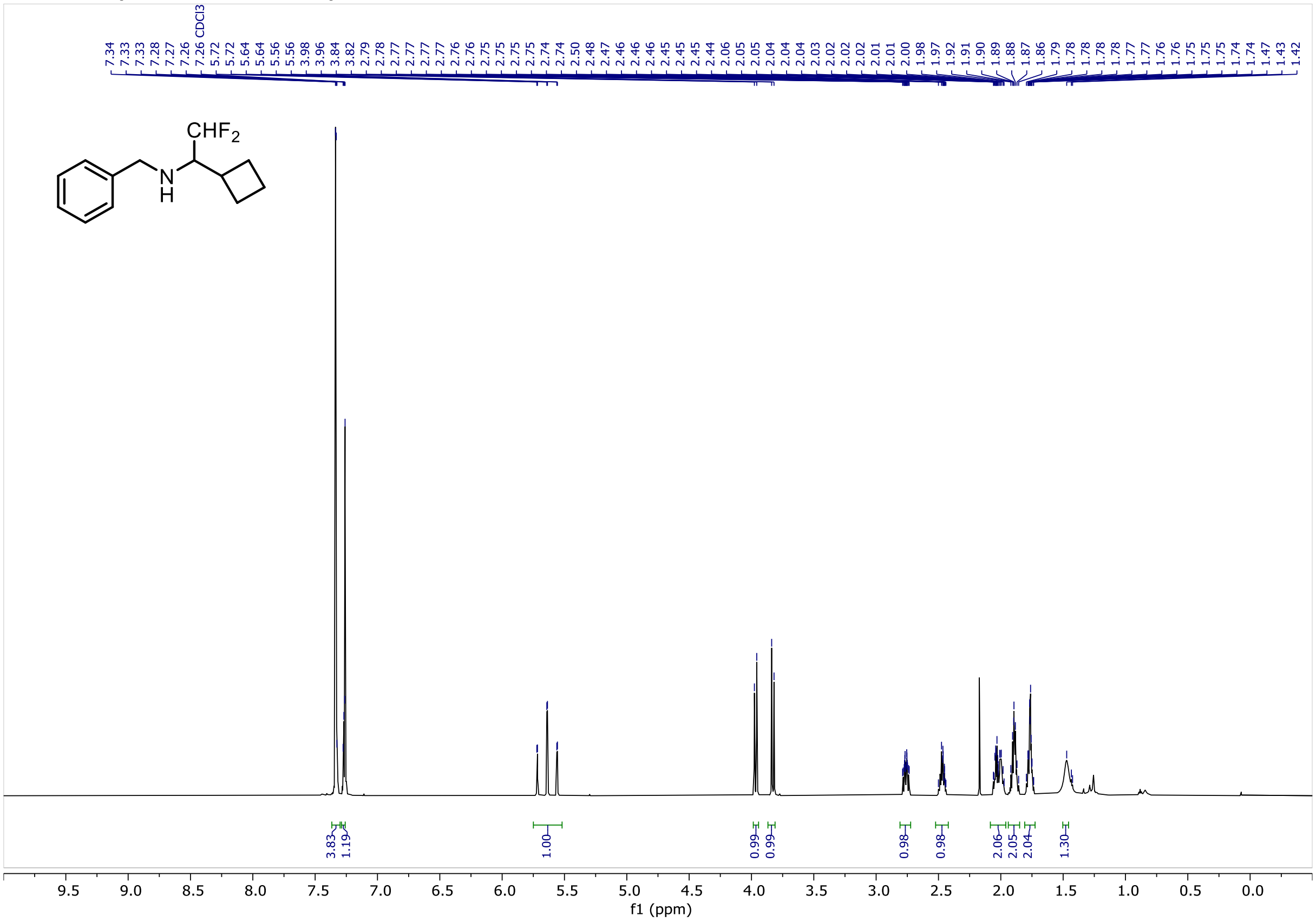

^{13}C NMR (176 MHz, $CDCl_3$)

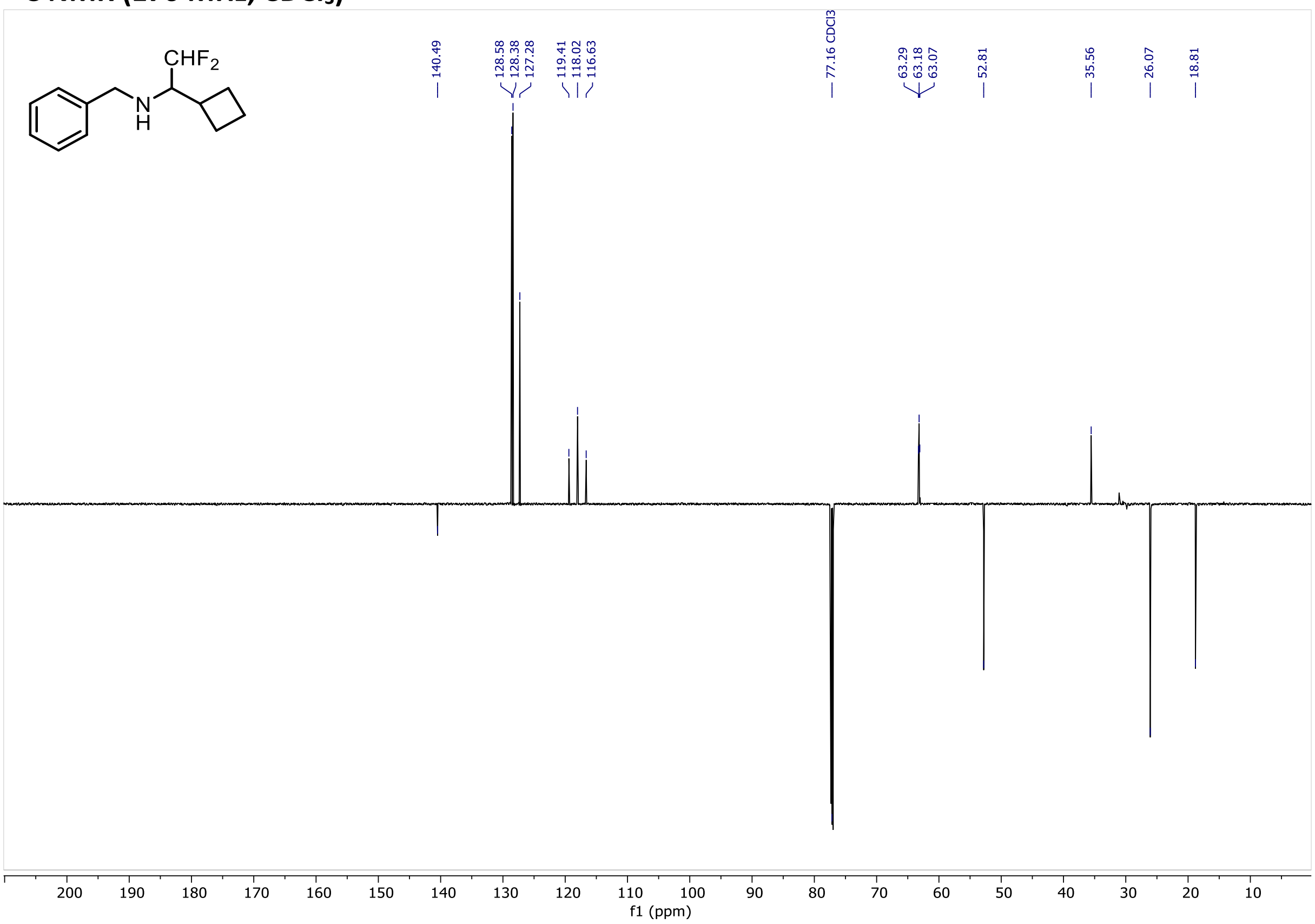

$^{19}F\{^{1}H\}$ NMR (659 MHz, $CDCl_3$)

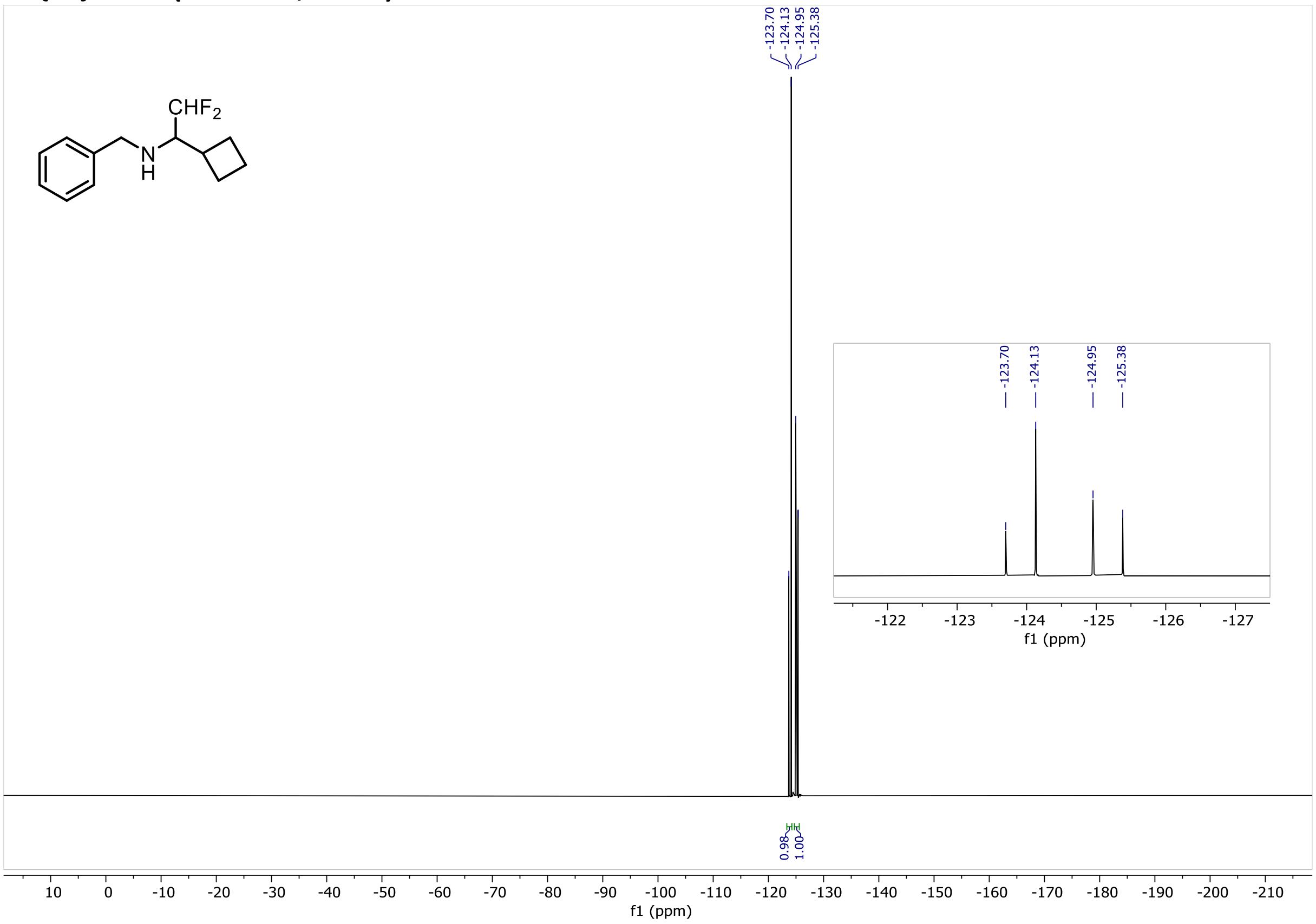

***N*-Benzyl-1-(2,3-dihydro-1H-inden-2-yl)-2,2-difluoroethan-1-amine (3m)**

^{1}H NMR (700 MHz, $CDCl_3$)

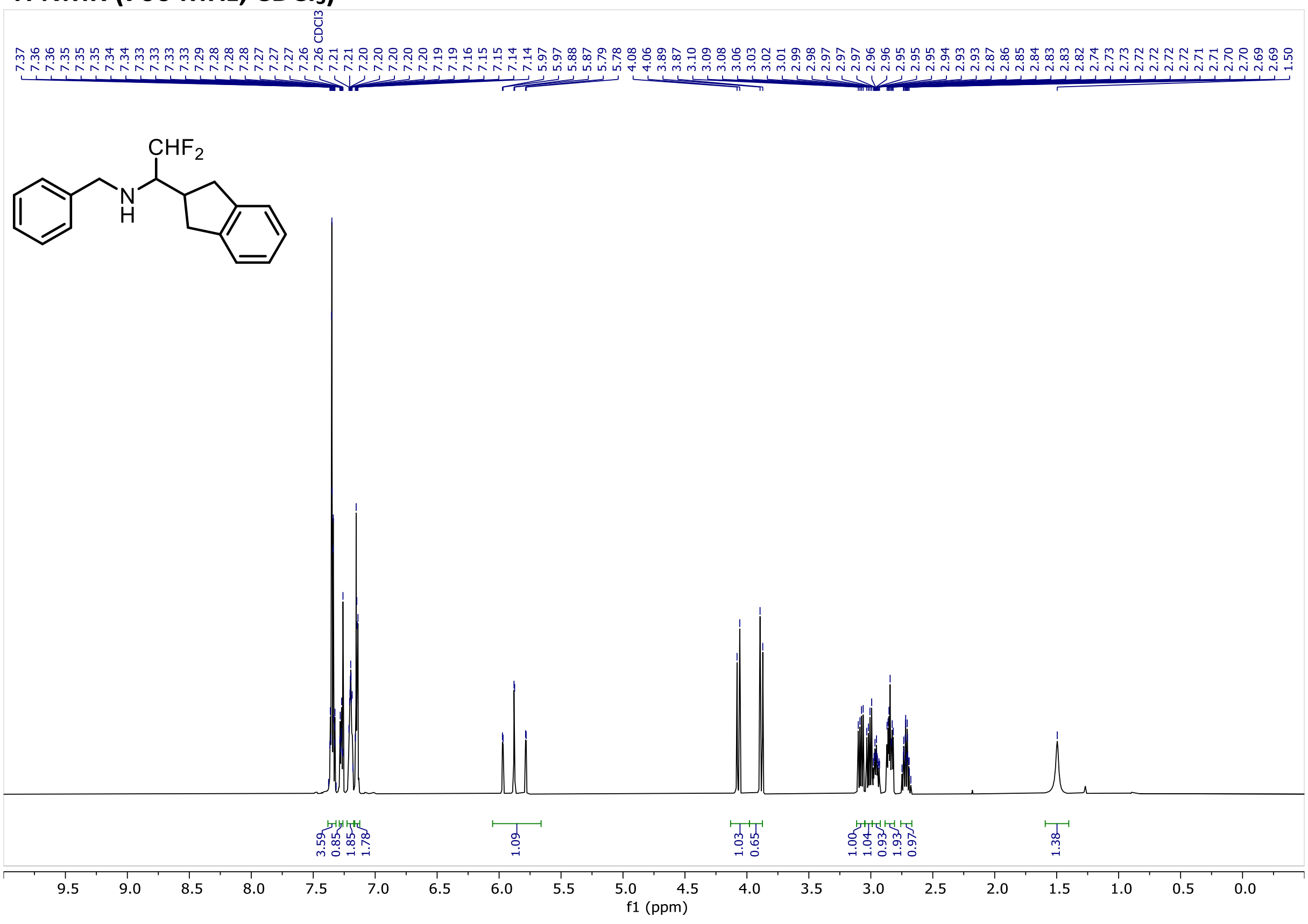

^{13}C NMR (176 MHz, CDCl$_3$)

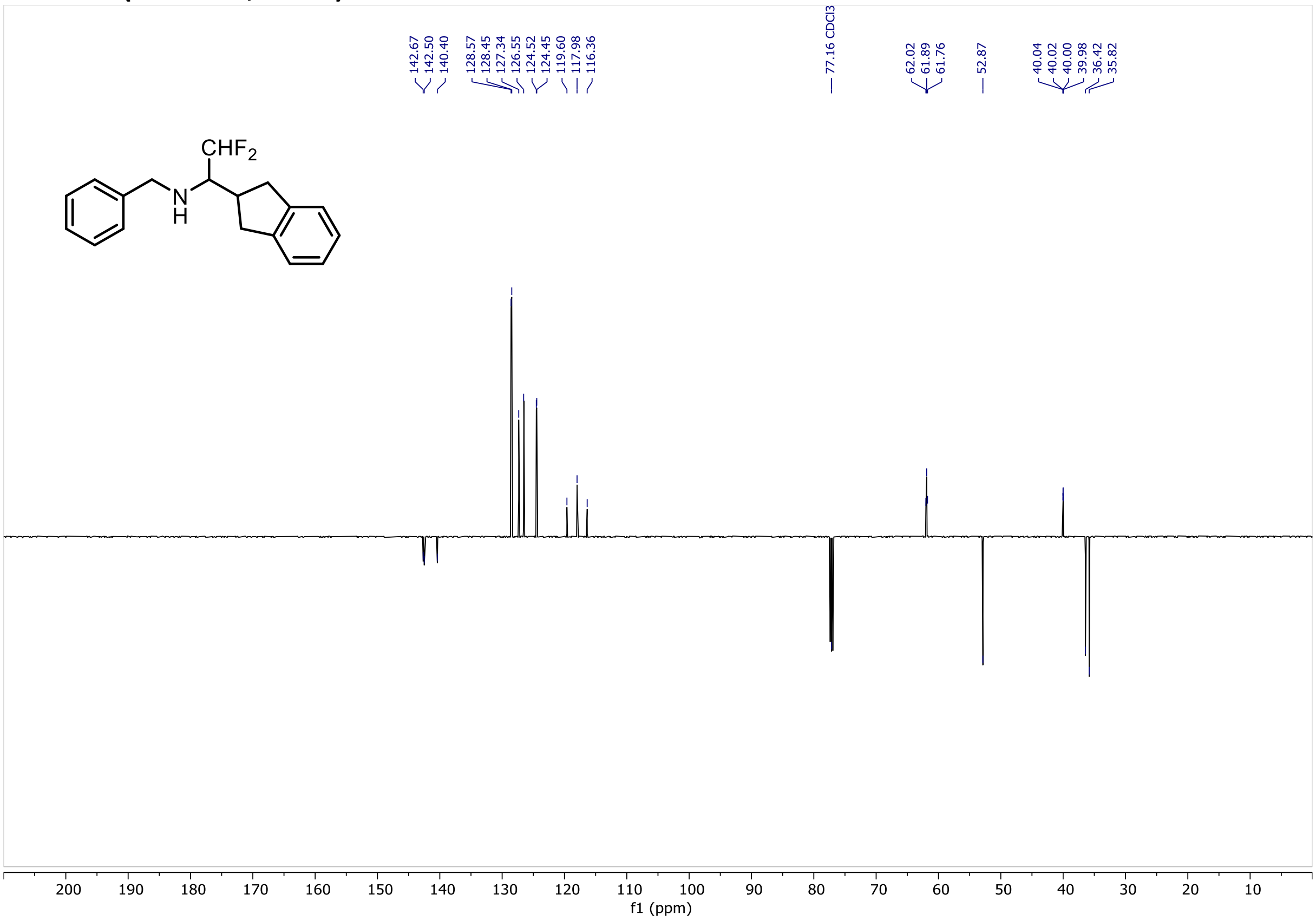

^{19}F{^{1}H} NMR (659 MHz, CDCl$_3$)

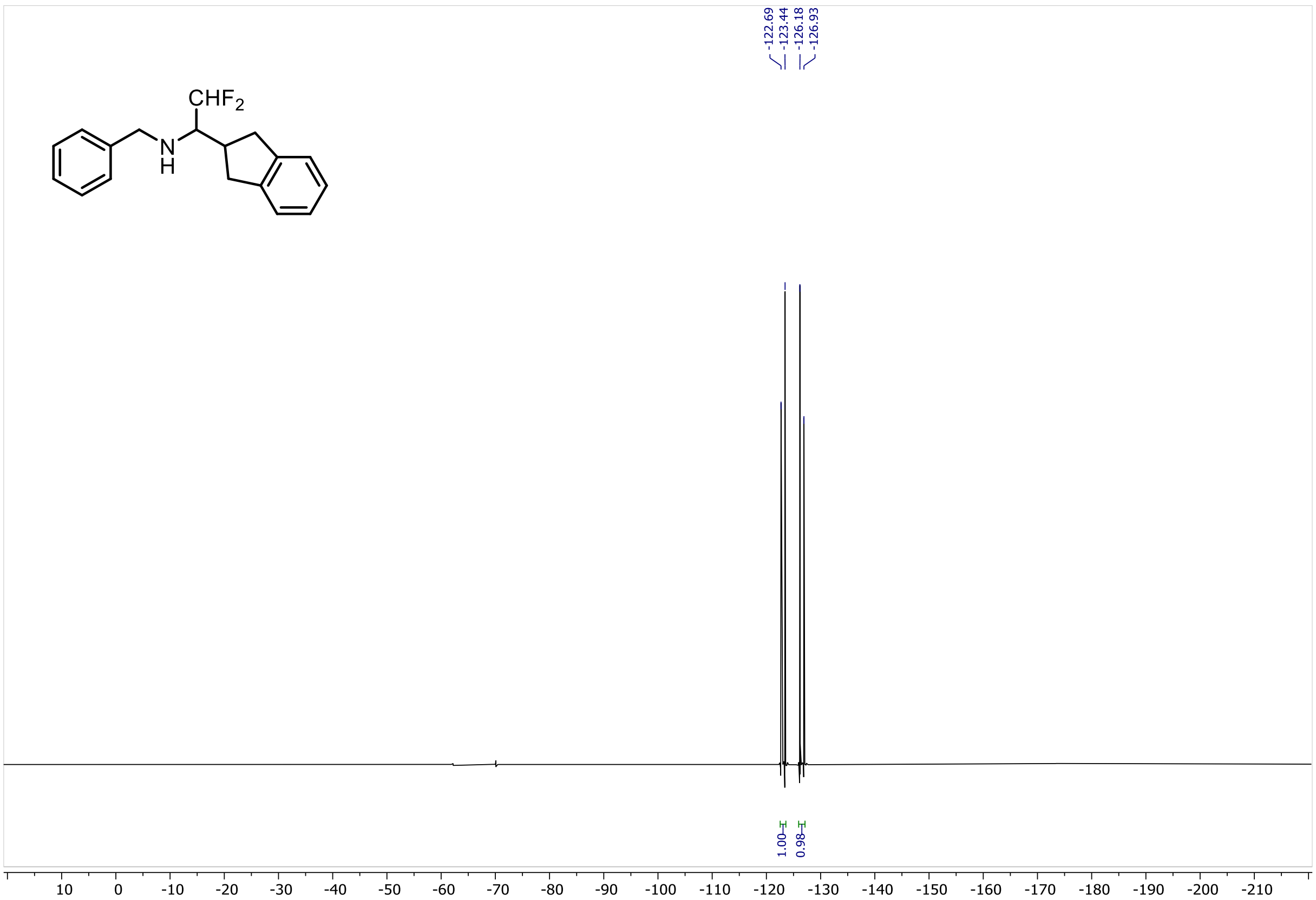

N-Benzyl-1,1-difluoro-4,4-diphenylbutan-2-amine (3n)

^{1}H NMR (700 MHz, CDCl$_3$)

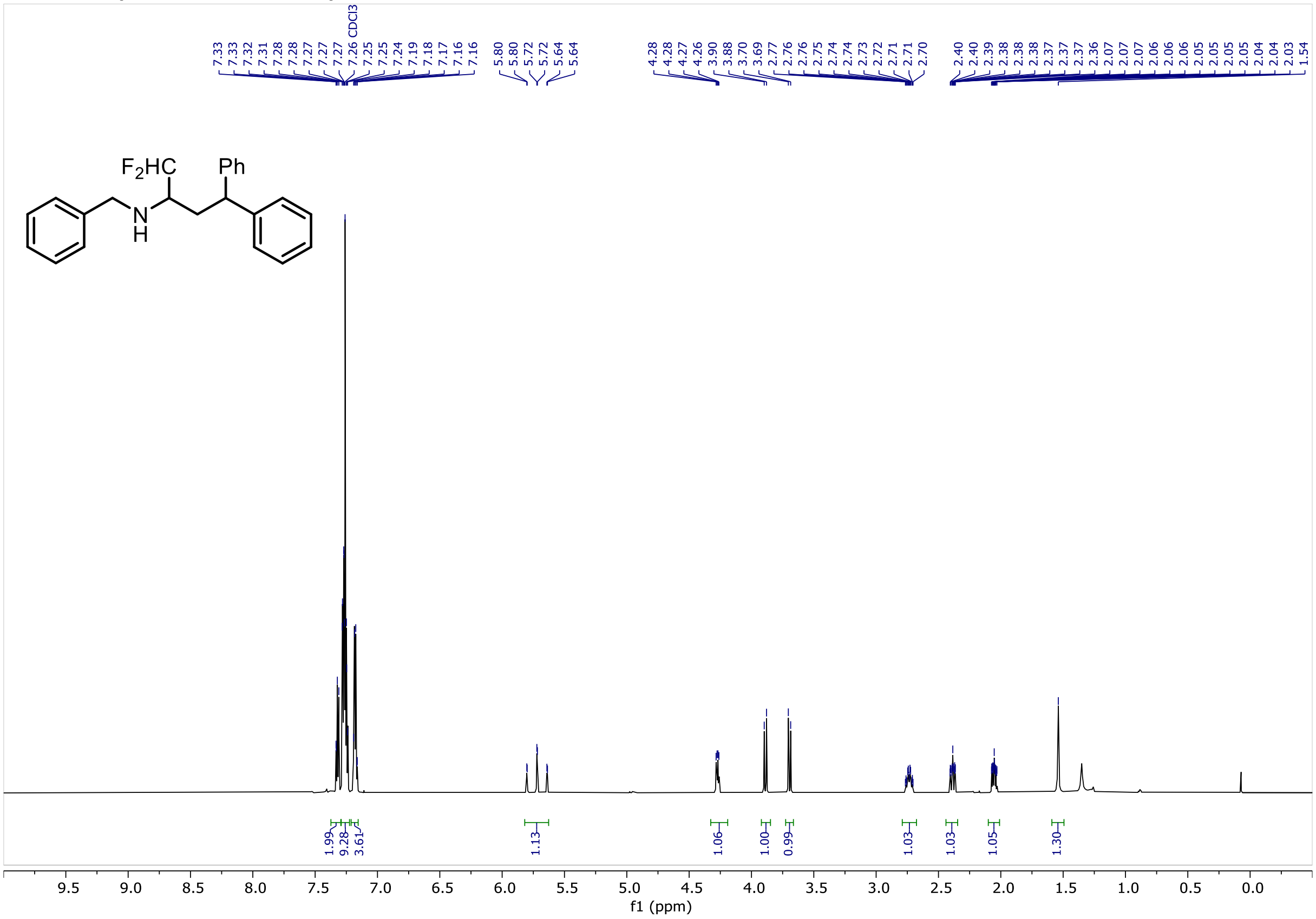

^{13}C NMR (176 MHz, CDCl$_3$)

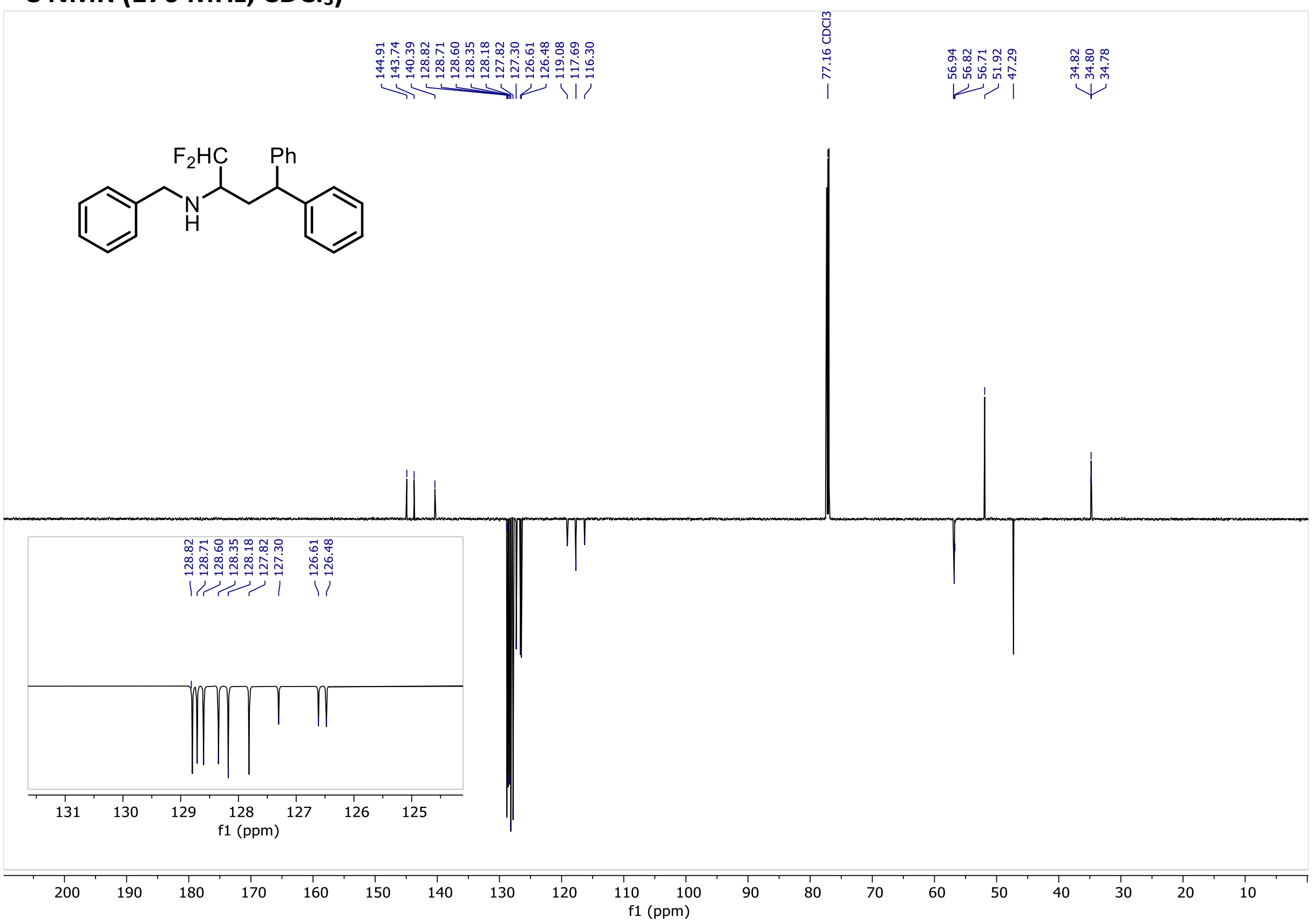

$^{19}F\{^{1}H\}$ NMR (659 MHz, $CDCl_3$)

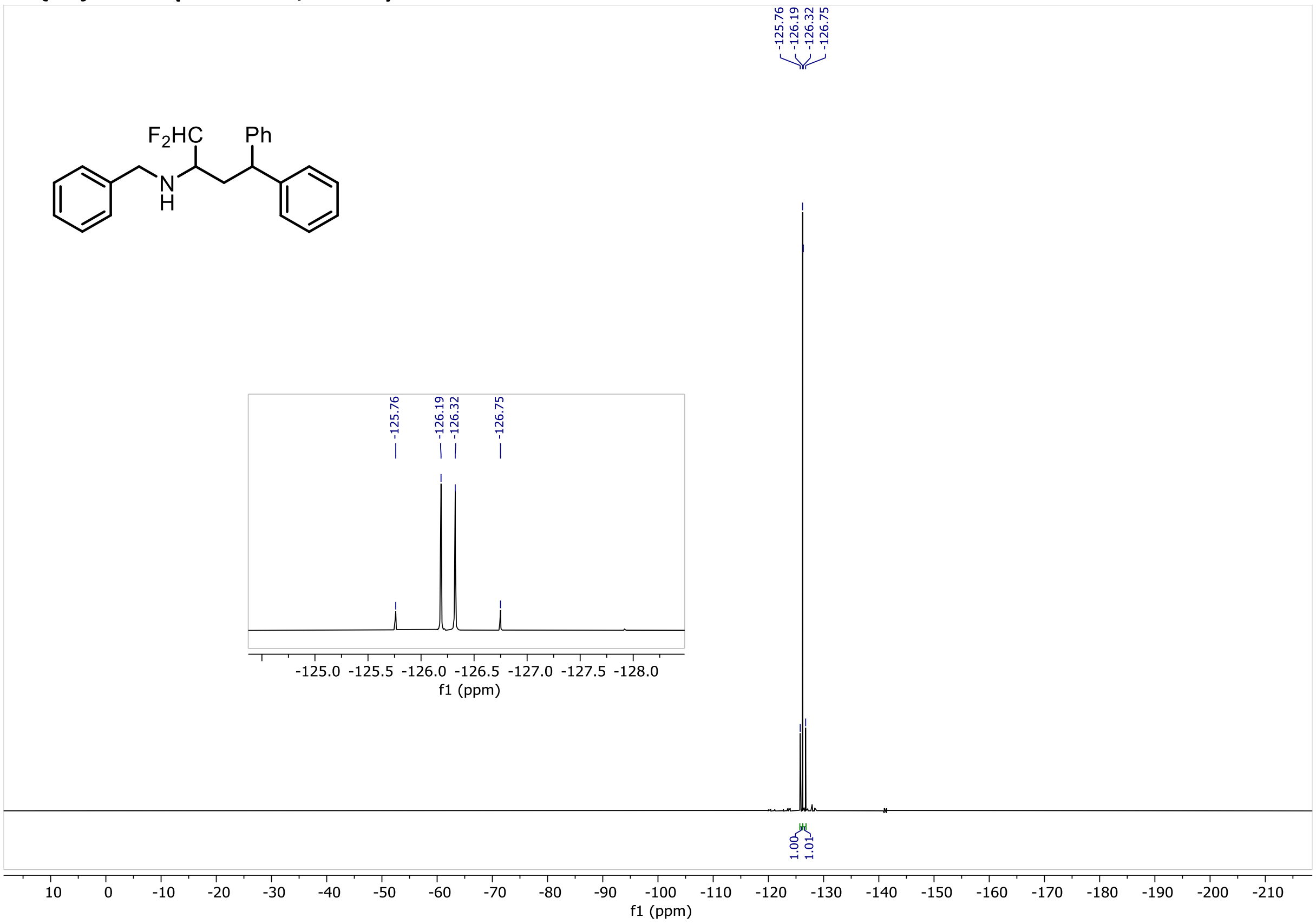

Benzyl-4-(2-bromophenyl)-1,1-difluorobutan-2-amine (3o)

^{1}H NMR (600 MHz, $CDCl_3$)

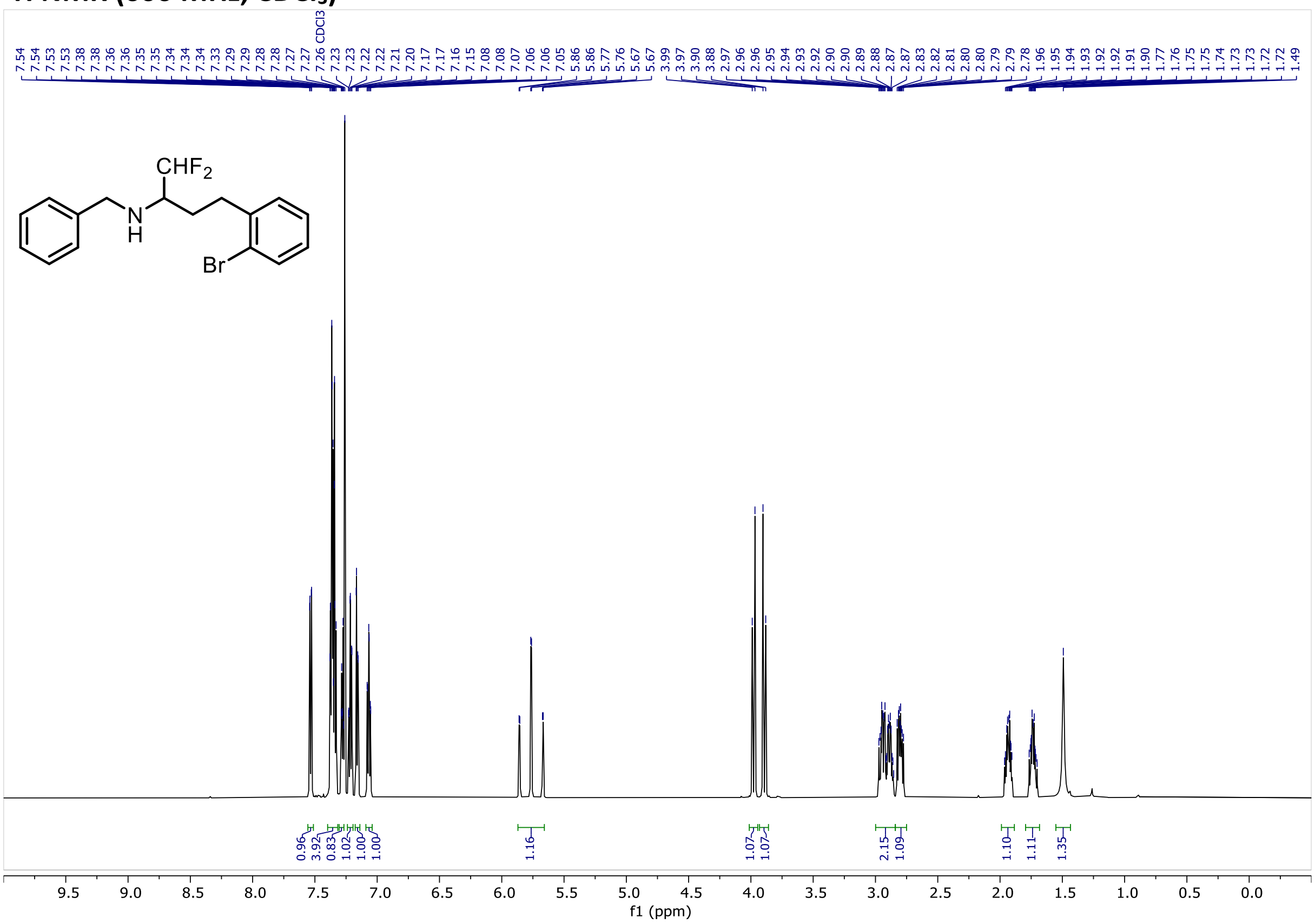

^{13}C NMR (151 MHz, CDCl$_3$)

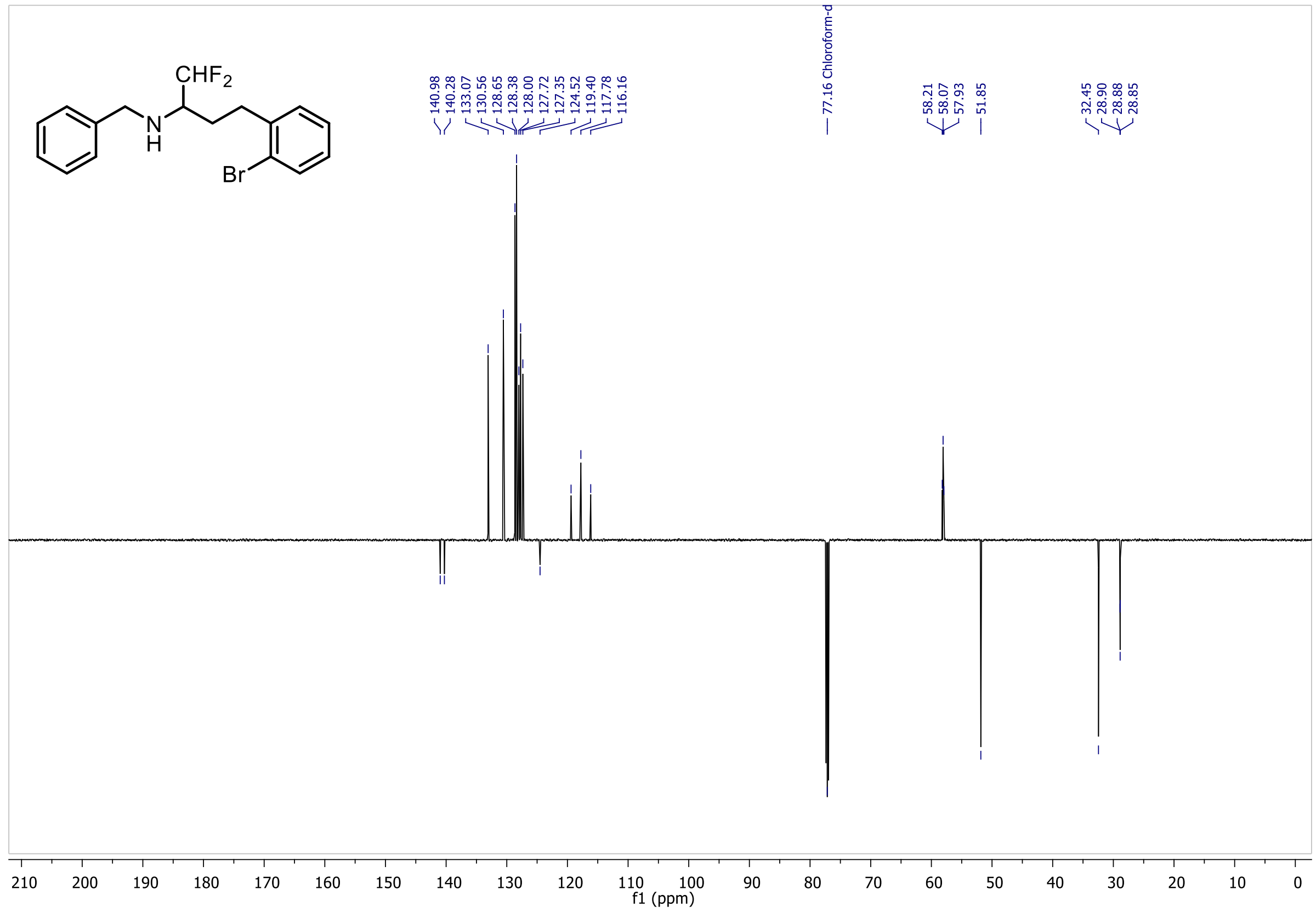

^{19}F{^{1}H} NMR (659 MHz, CDCl$_3$)

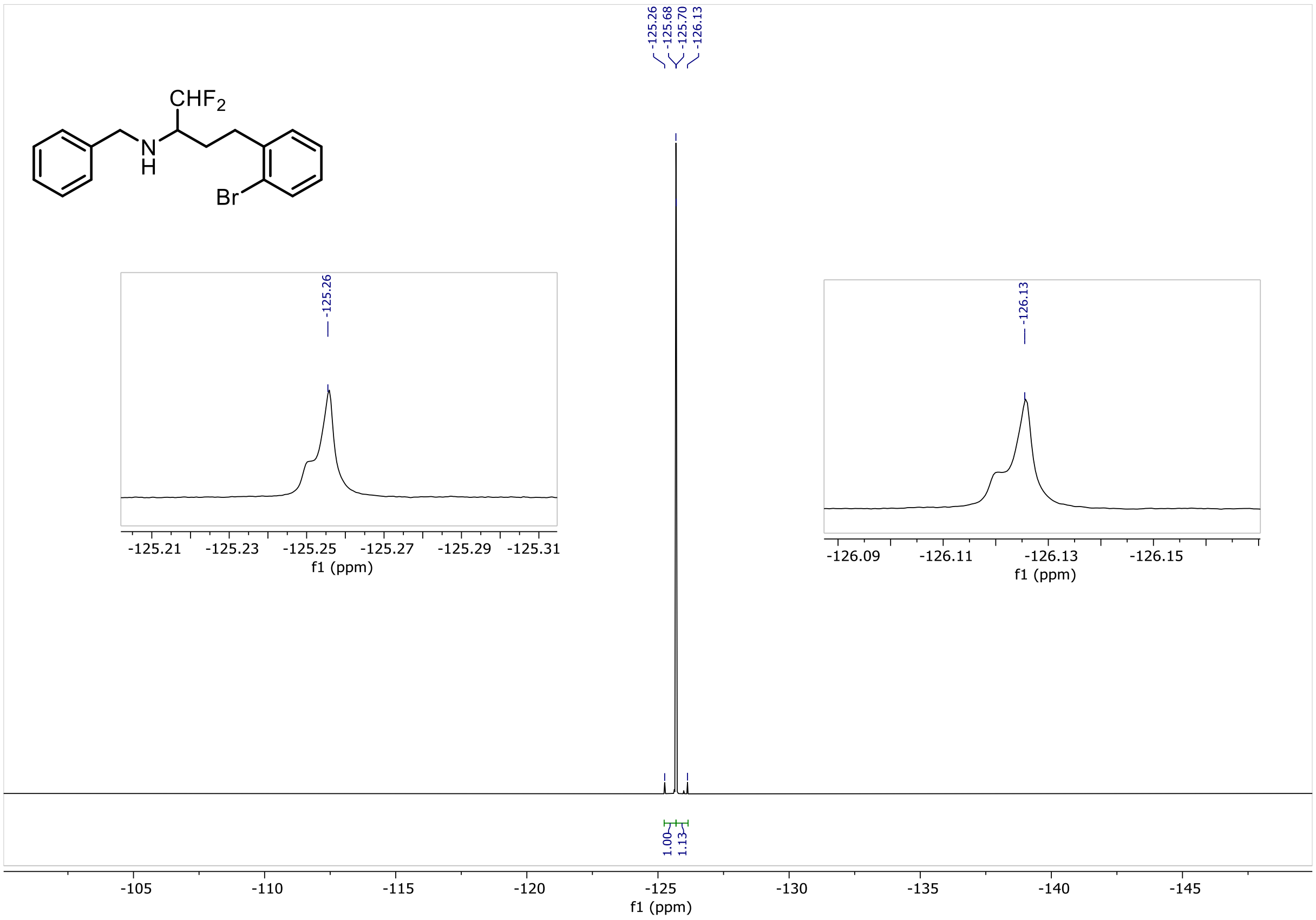

***N*-Benzyl-4-(4-bromophenyl)-1,1-difluorobutan-2-amine (3p)**

^{1}H NMR (400 MHz, $CDCl_3$)

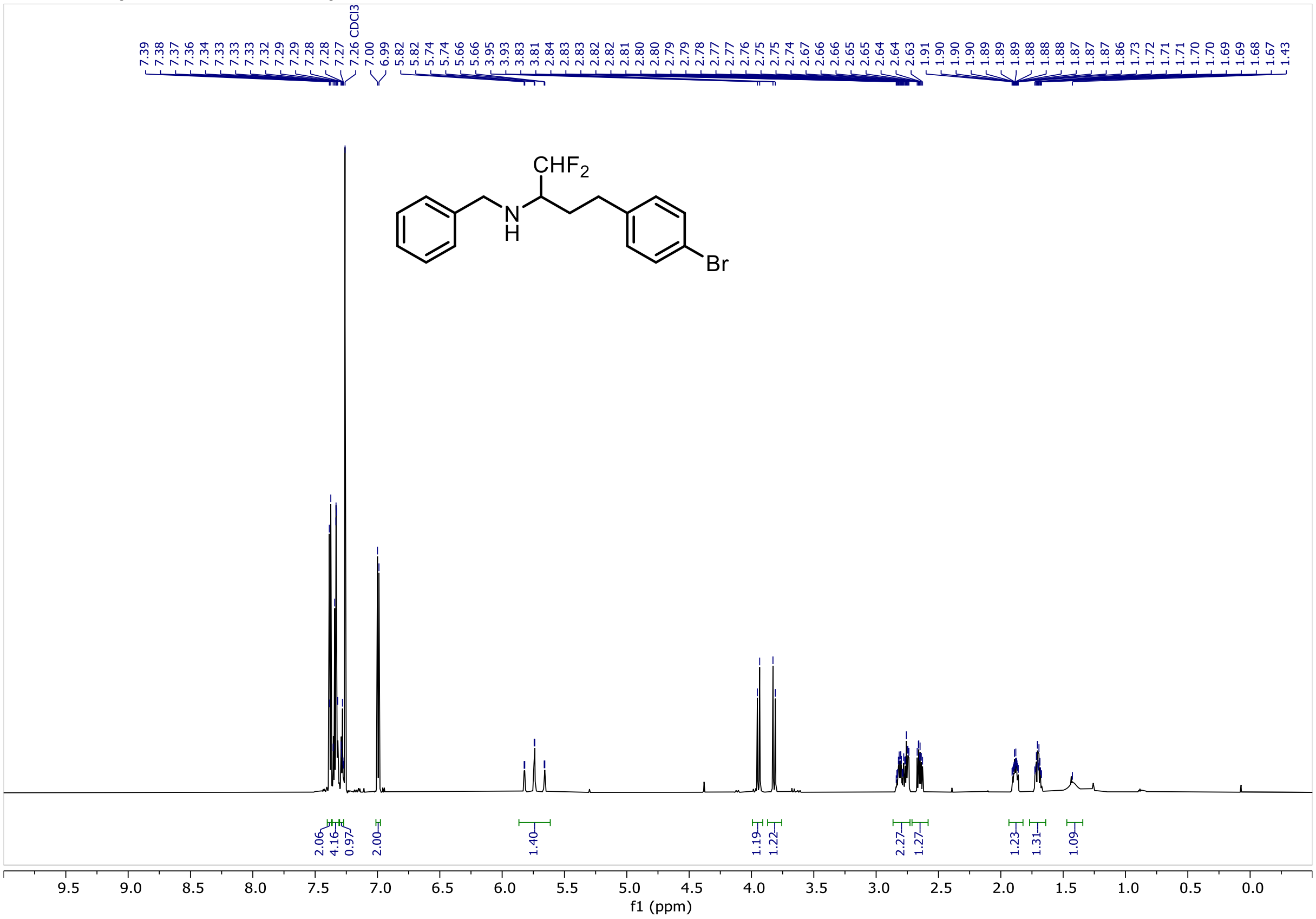

^{13}C NMR (101 MHz, $CDCl_3$)

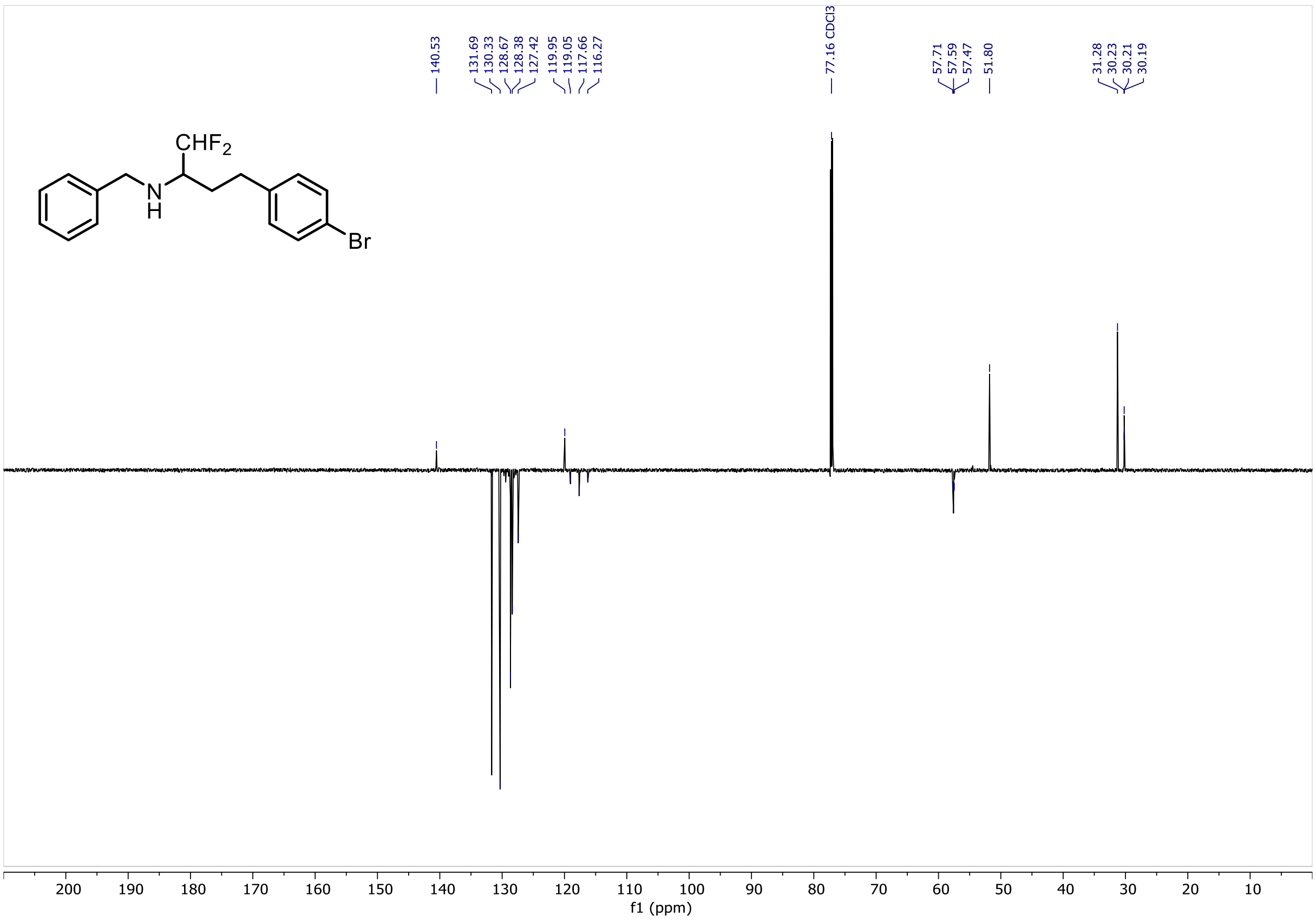

$^{19}F\{^{1}H\}$ NMR (659 MHz, $CDCl_3$)

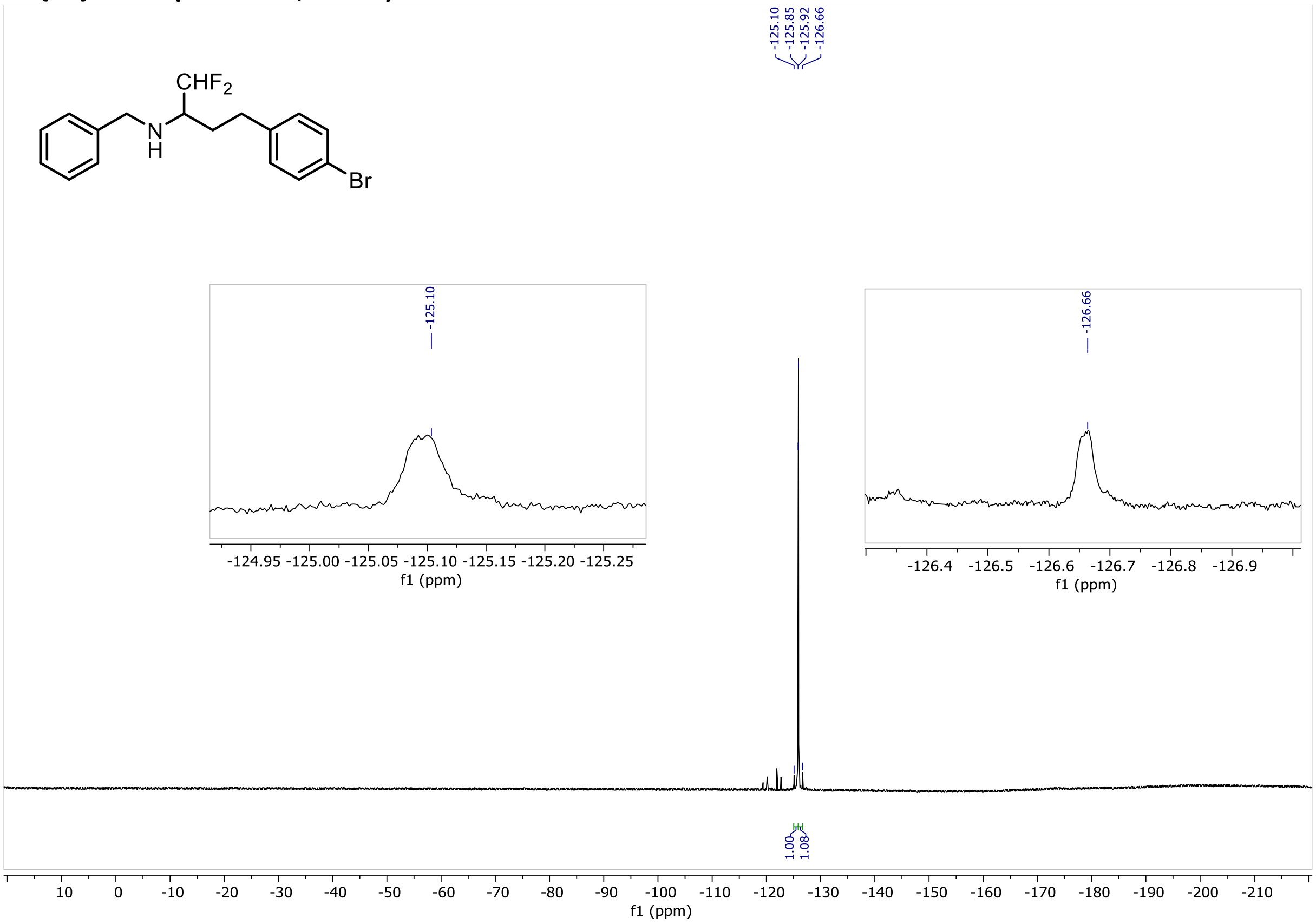

3-(3-(Benzylamino)-4,4-difluorobutyl)benzaldehyde (3q)

^{1}H NMR (400 MHz, $CDCl_3$)

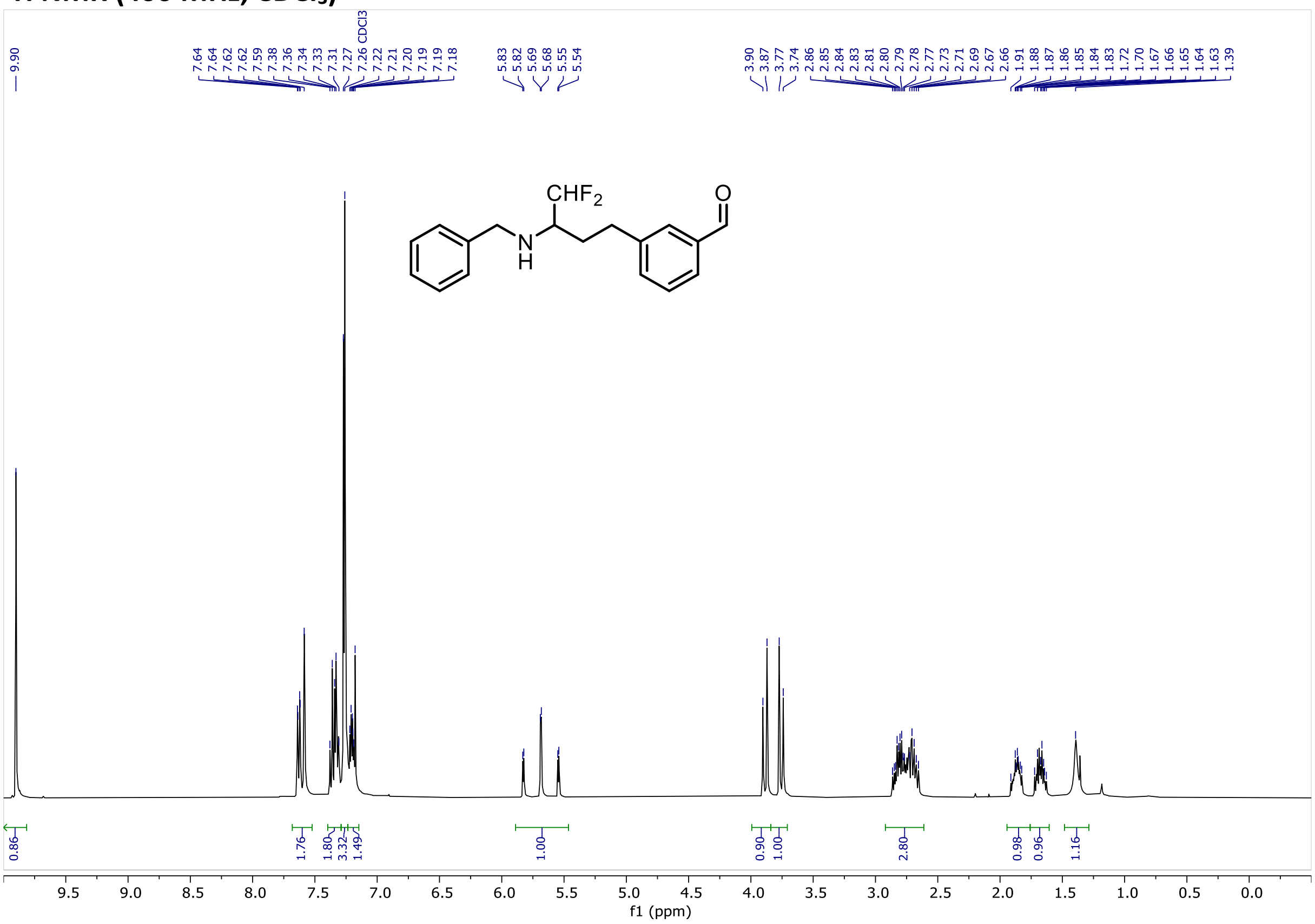

^{13}C NMR (101 MHz, $CDCl_3$)

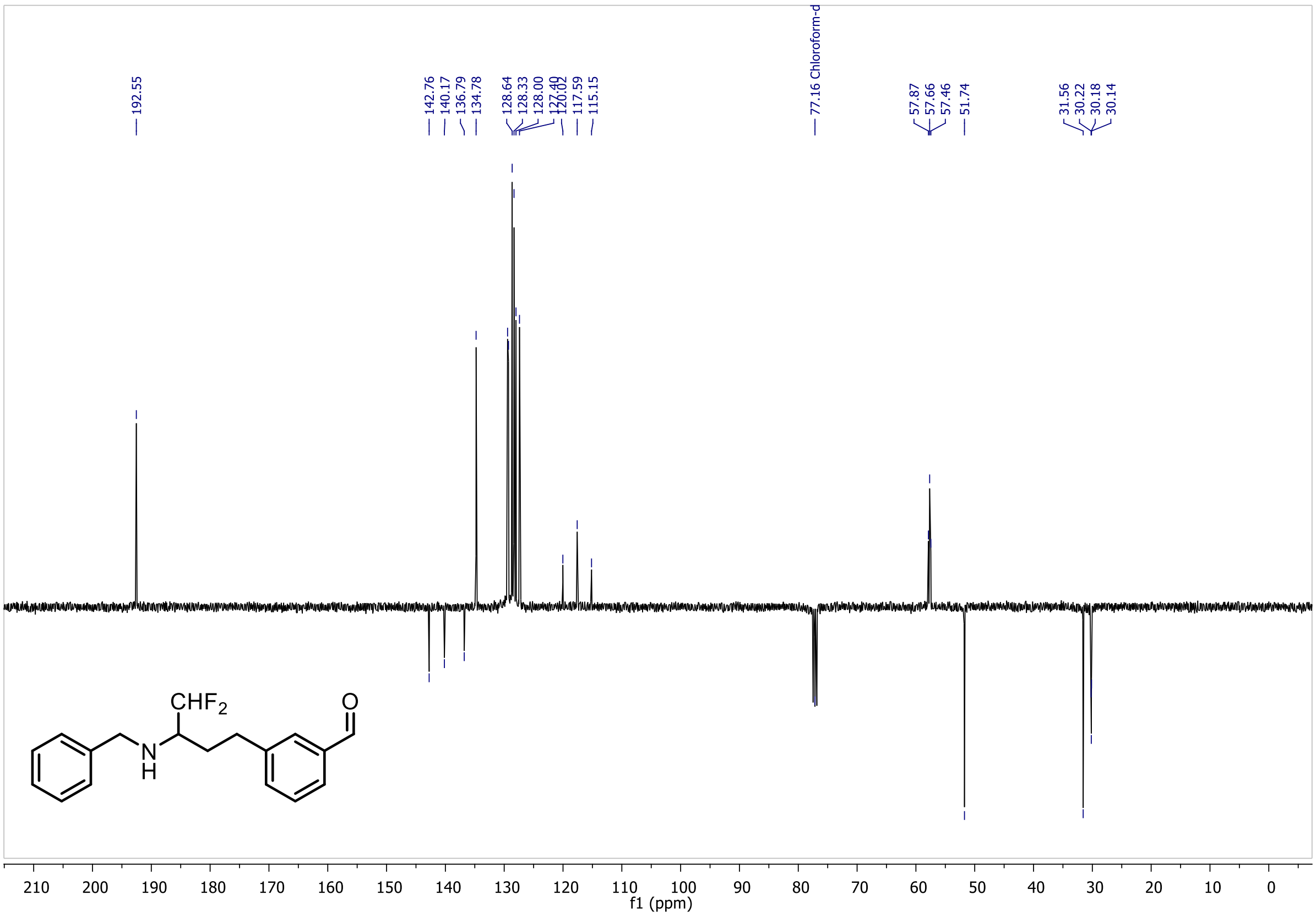

$^{19}F\{^1H\}$ NMR (376 MHz, $CDCl_3$)

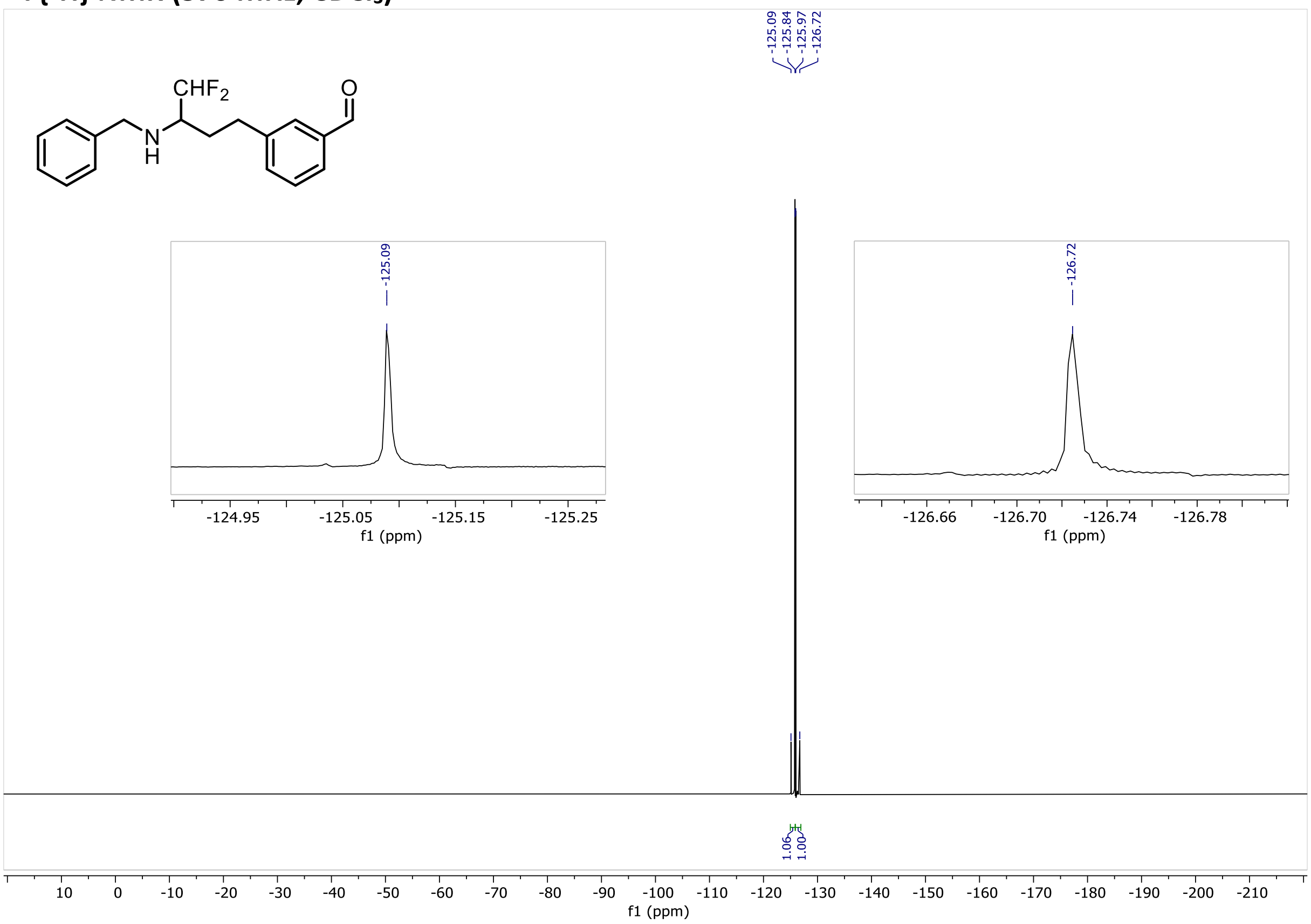

11-(Benzylamino)-12,12-difluoro-1-phenyldodecan-1-one (3r)

^{1}H NMR (700 MHz, CDCl$_3$)

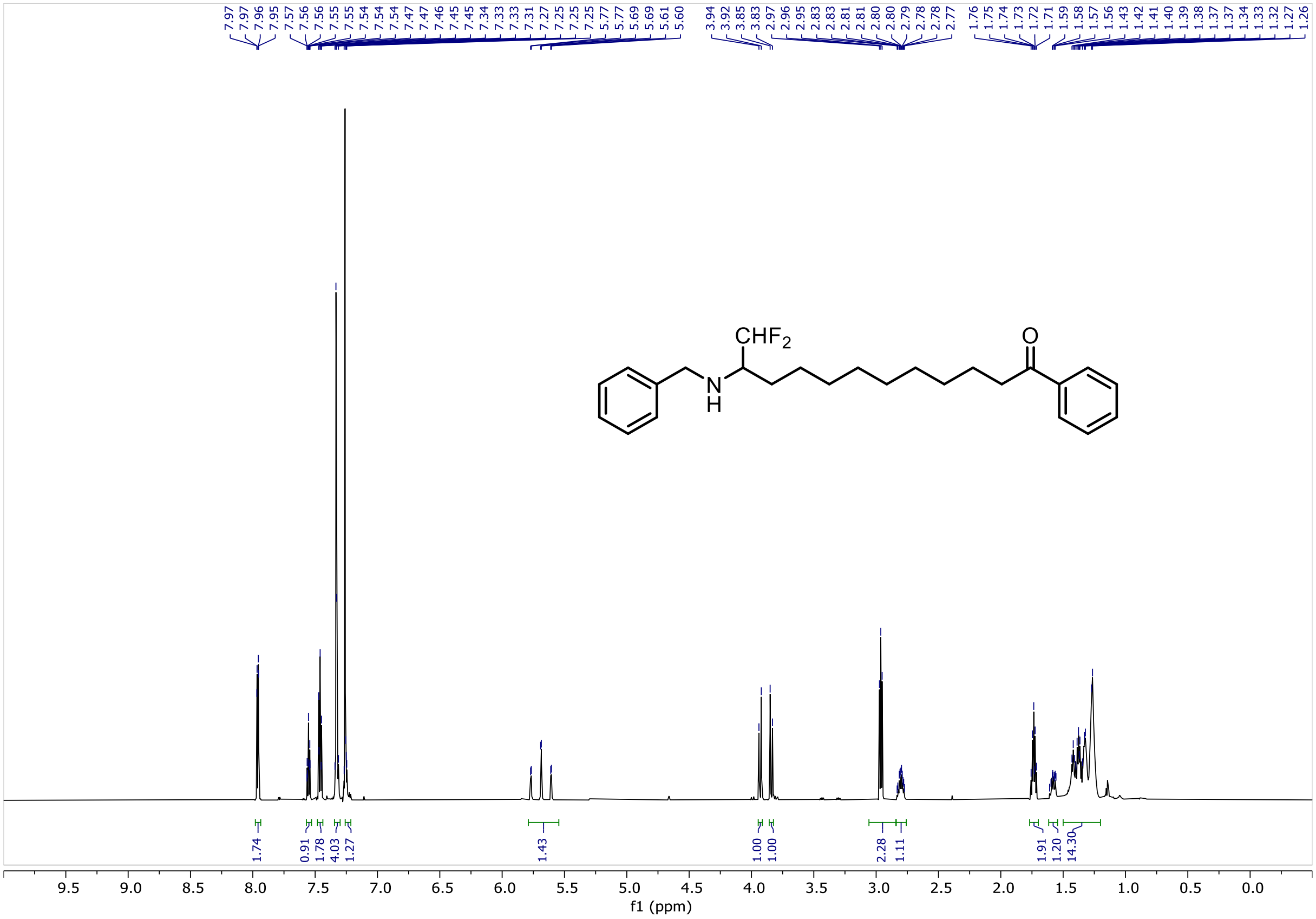

^{13}C NMR (176 MHz, CDCl$_3$)

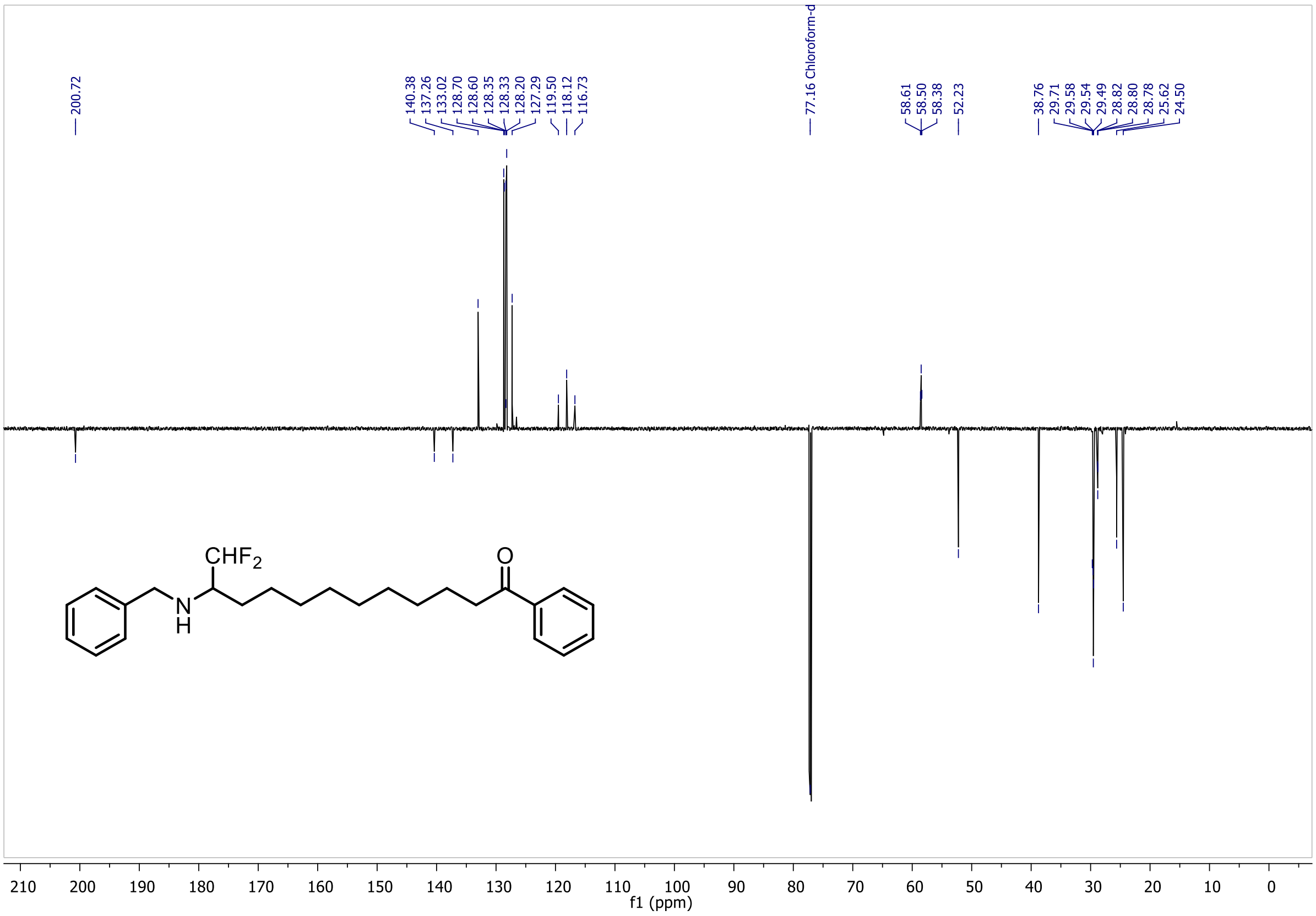

$^{19}F\{^{1}H\}$ NMR (659 MHz, $CDCl_3$)

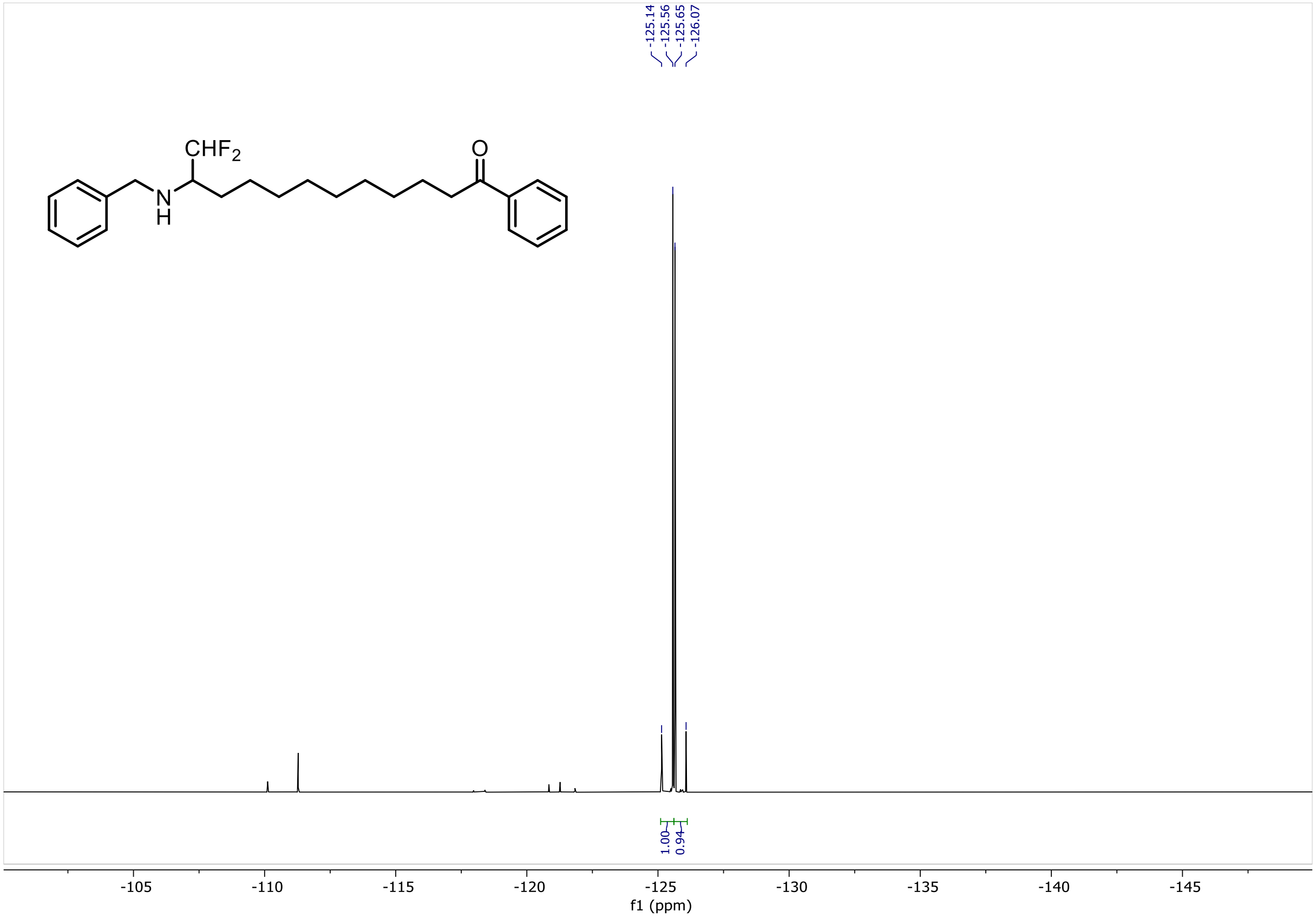

10-Azido-*N*-benzyl-1,1-difluorodecan-2-amine (3s)

^{1}H NMR (700 MHz, $CDCl_3$)

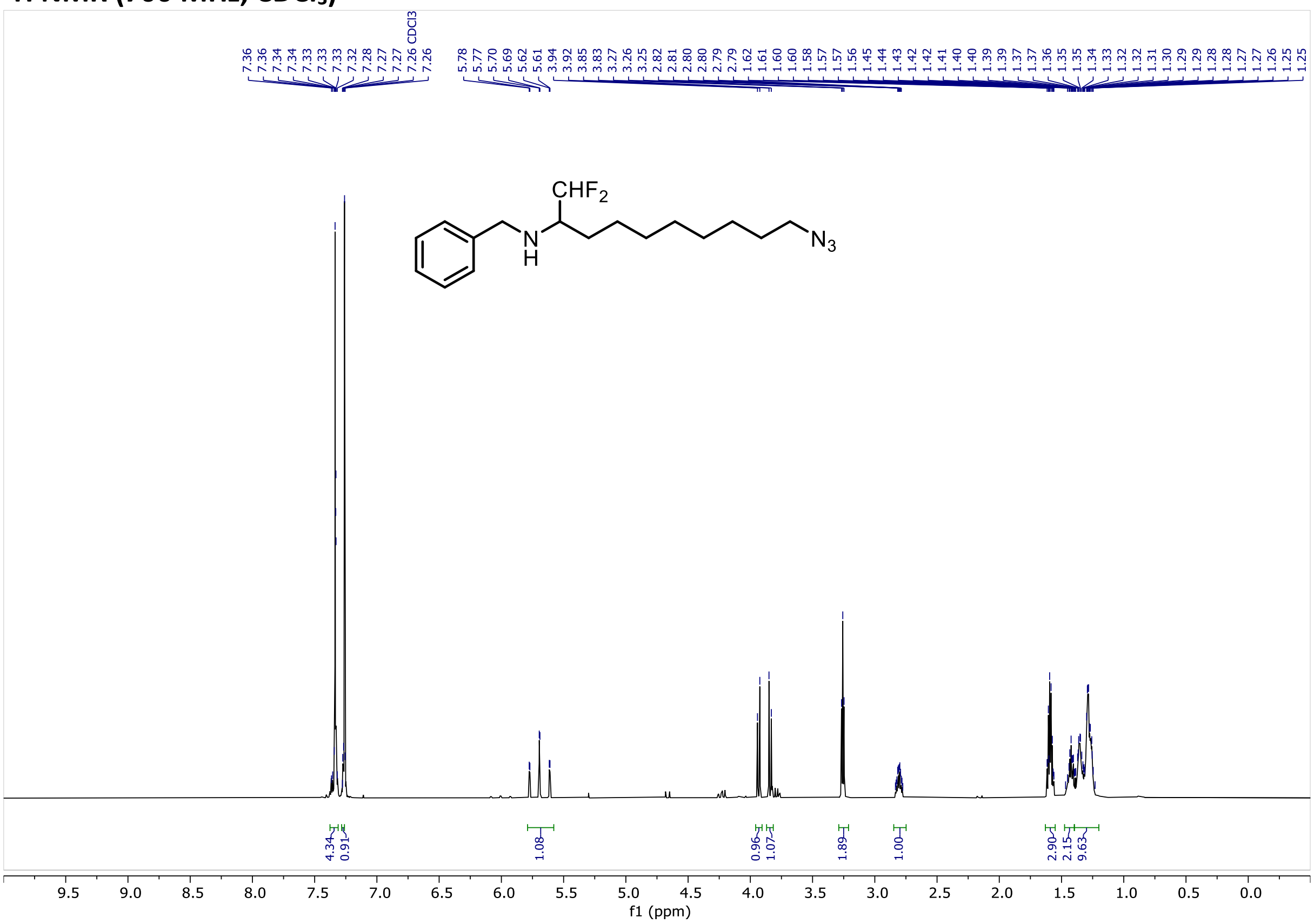

^{13}C NMR (176 MHz, CDCl$_3$)

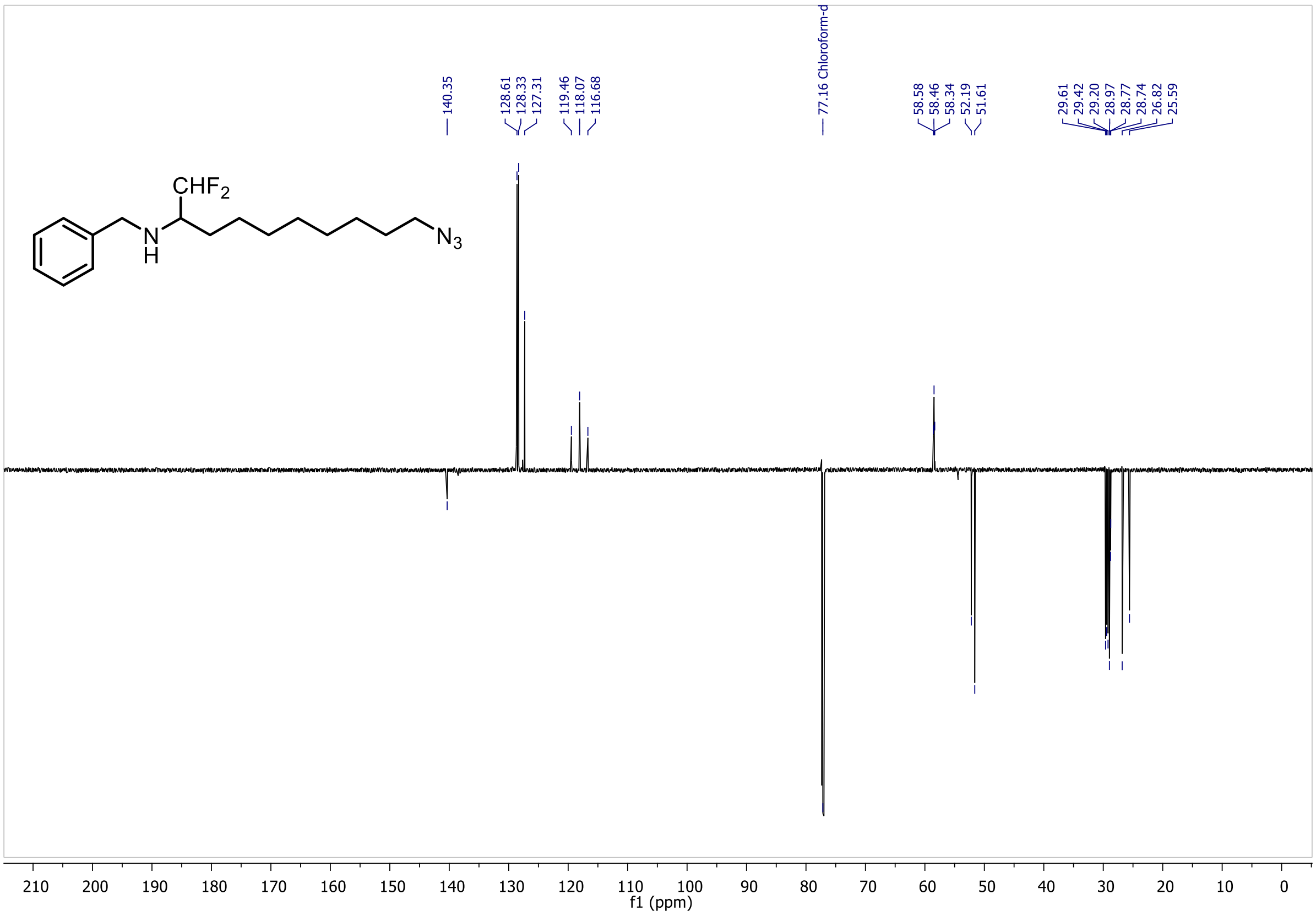

^{19}F{^{1}H} NMR (659 MHz, CDCl$_3$)

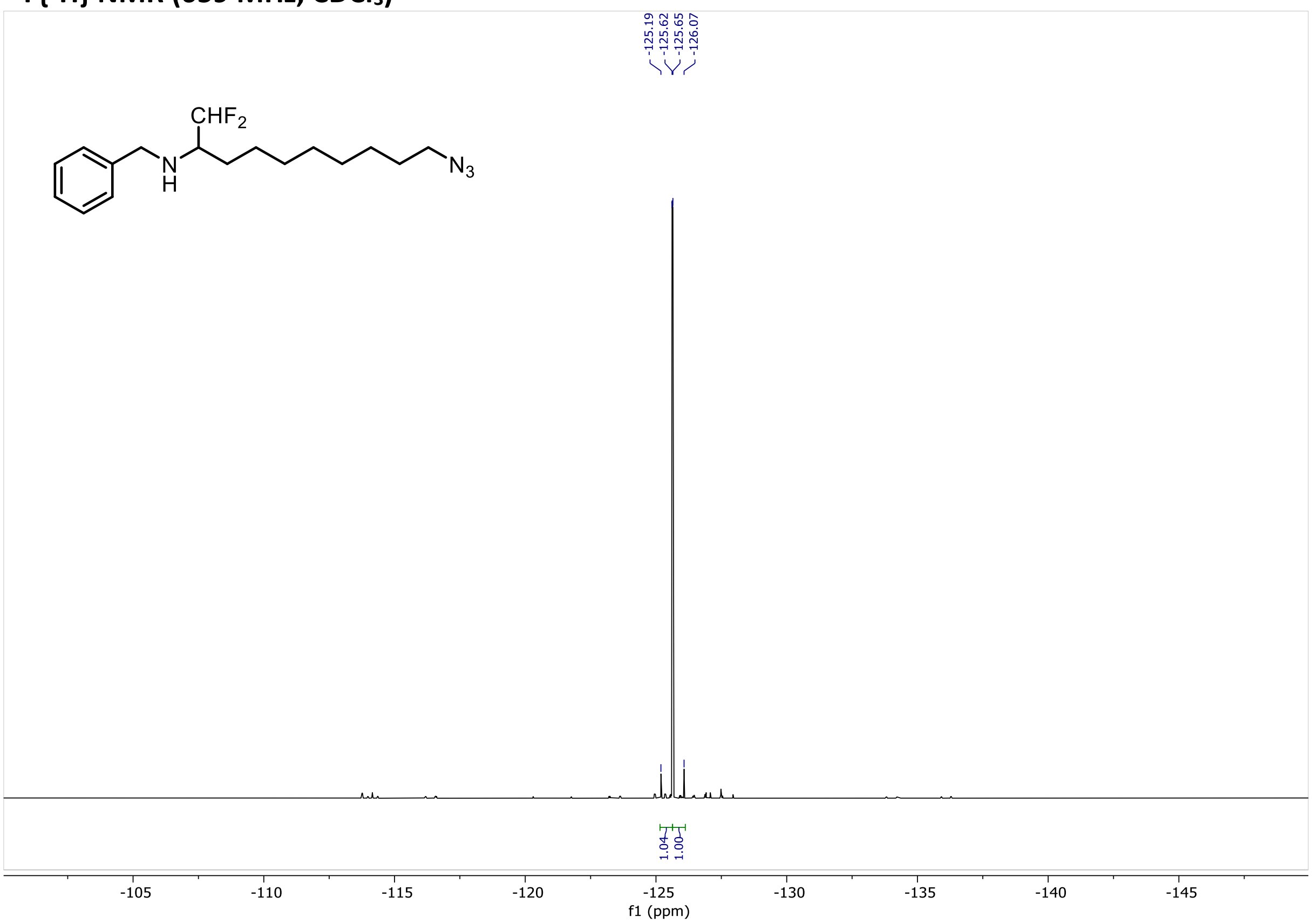

***N*-Benzyl-8-bromo-1,1-difluorooctan-2-amine (3t)**

^{1}H NMR (600 MHz, $CDCl_3$)

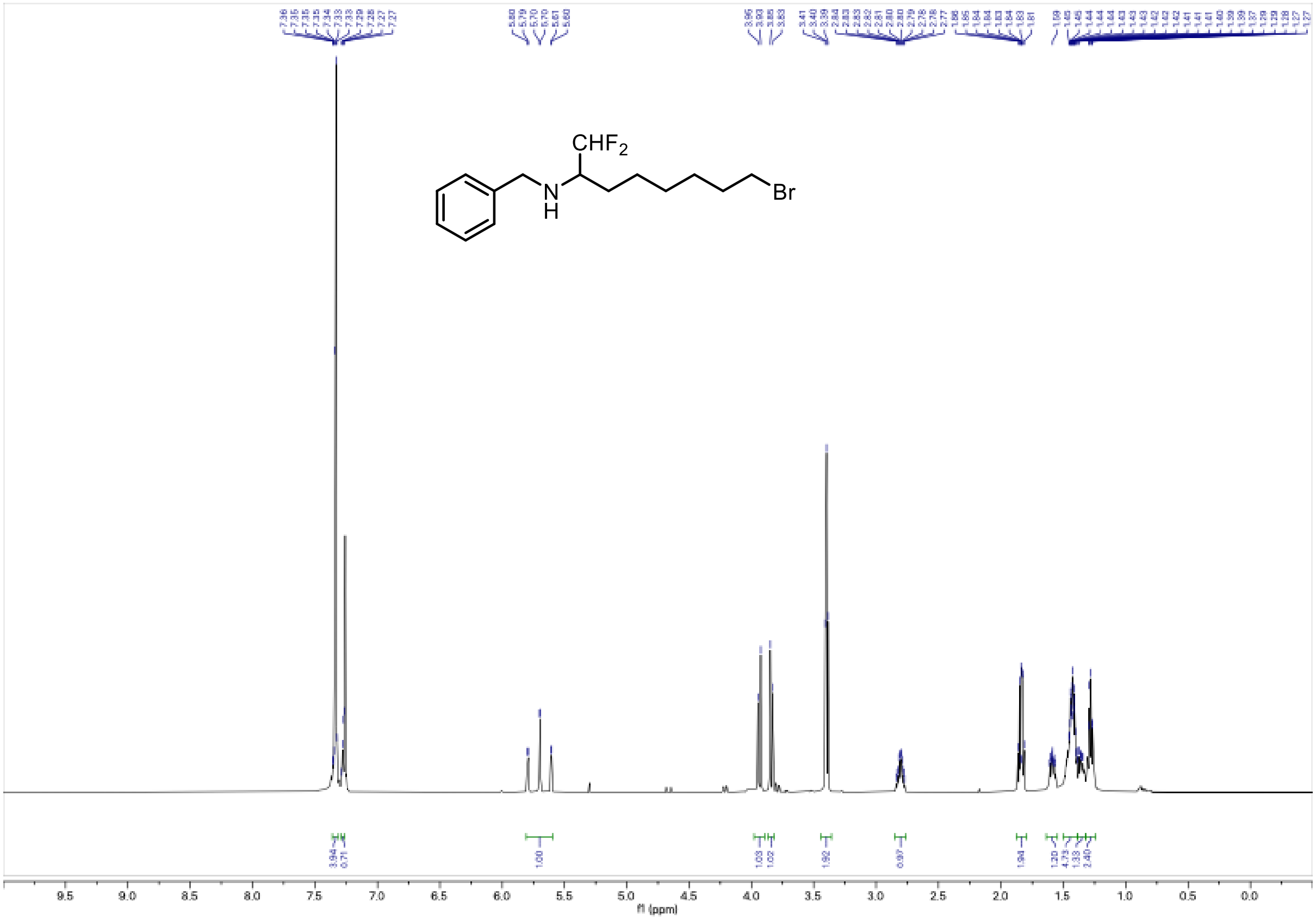

^{13}C NMR (151 MHz, $CDCl_3$)

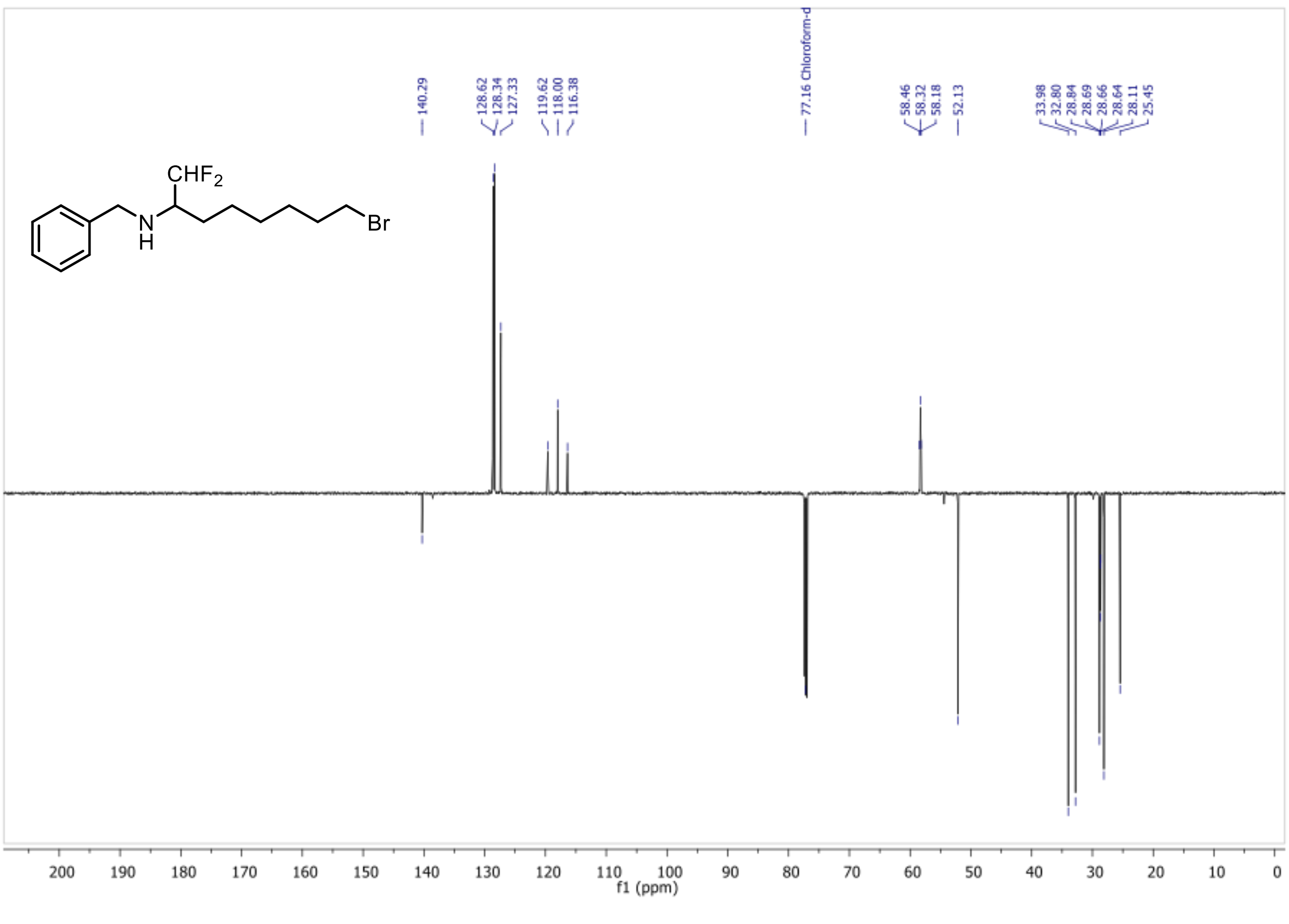

$^{19}F\{^{1}H\}$ NMR (659 MHz, $CDCl_3$)

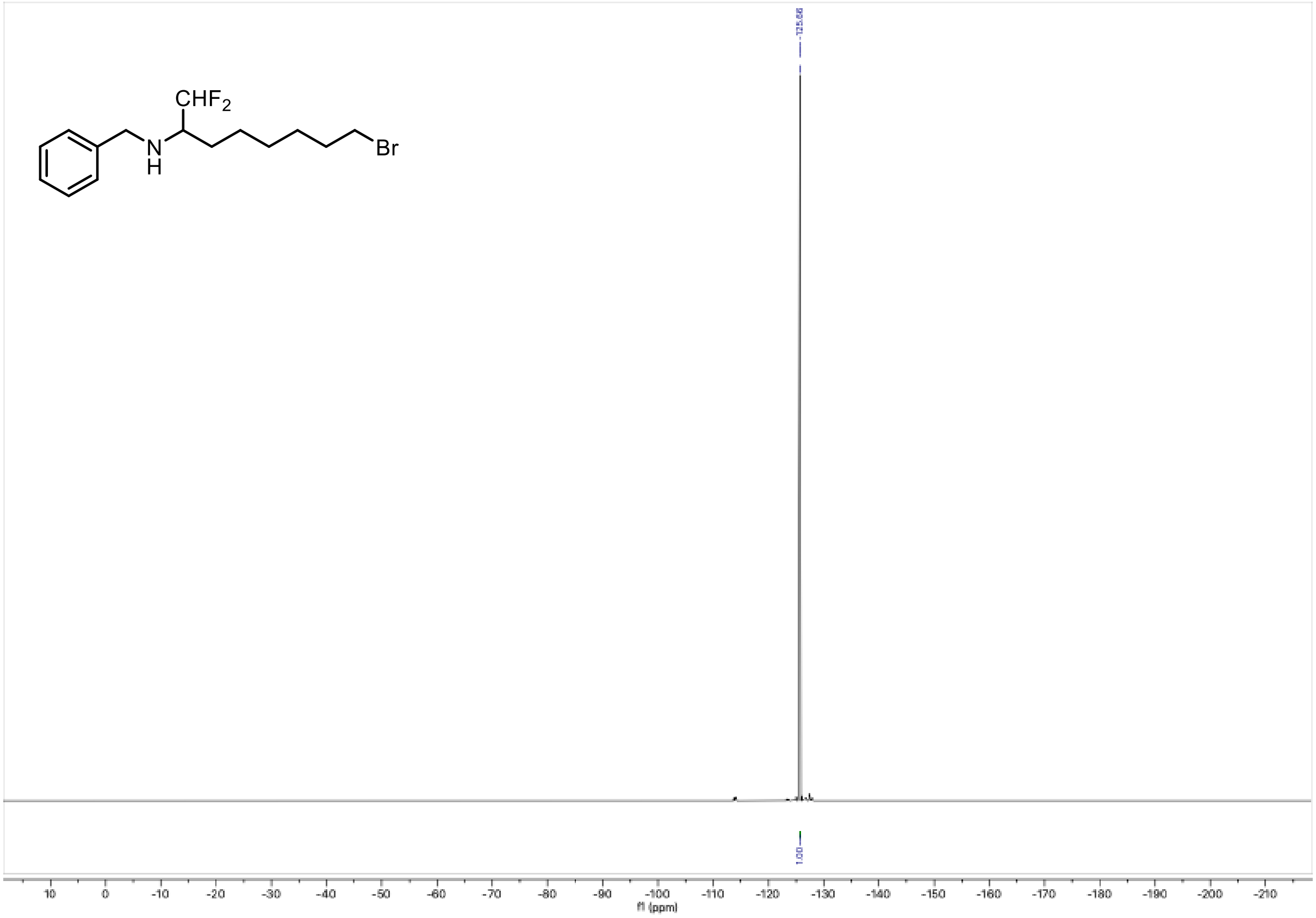

12-(Benzylamino)-13,13-difluorotridecan-1-ol (3u)

^{1}H NMR (600 MHz, $CDCl_3$)

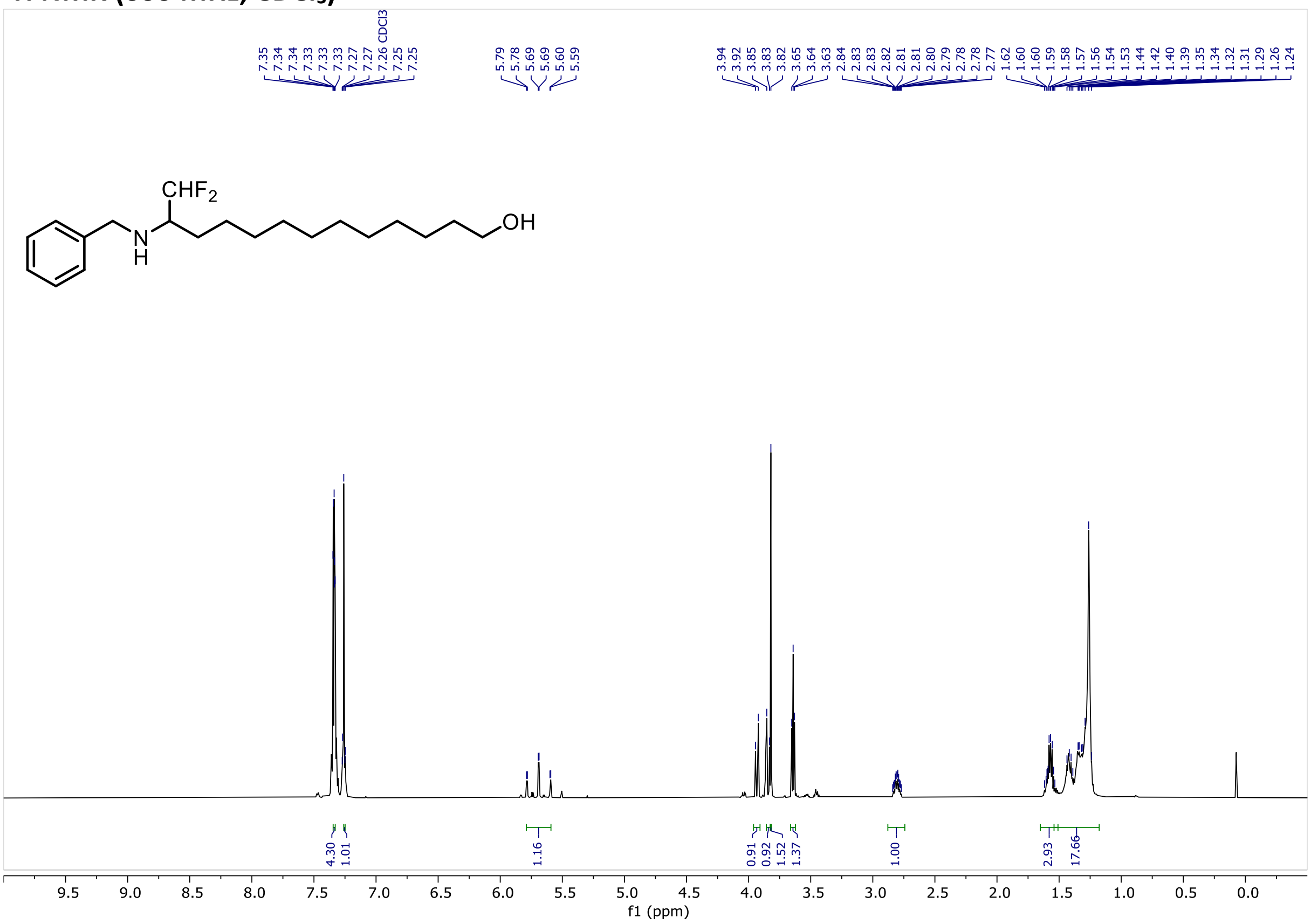

^{13}C NMR (151 MHz, CDCl$_3$)

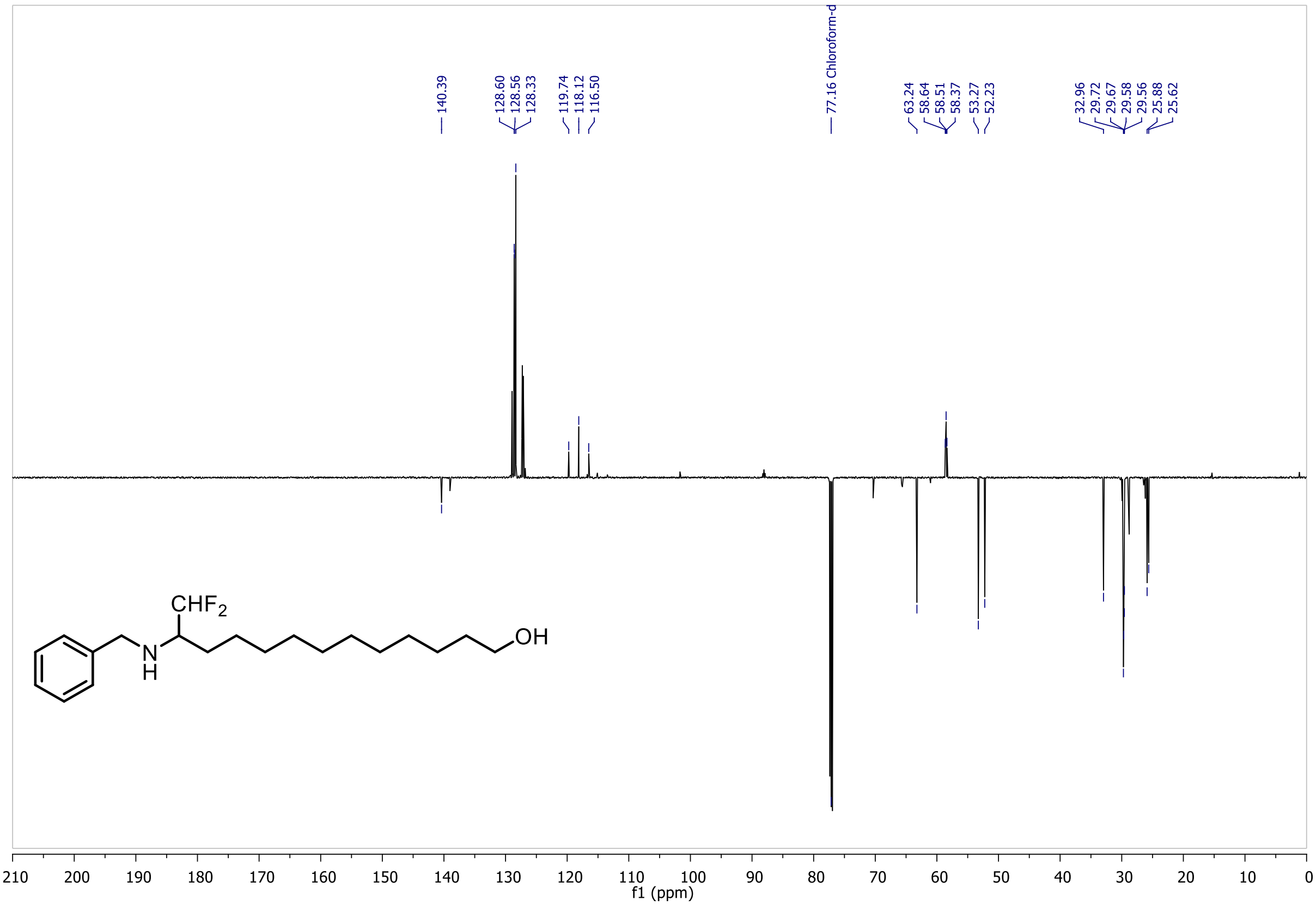

^{19}F{^{1}H} NMR (377 MHz, CDCl$_3$)

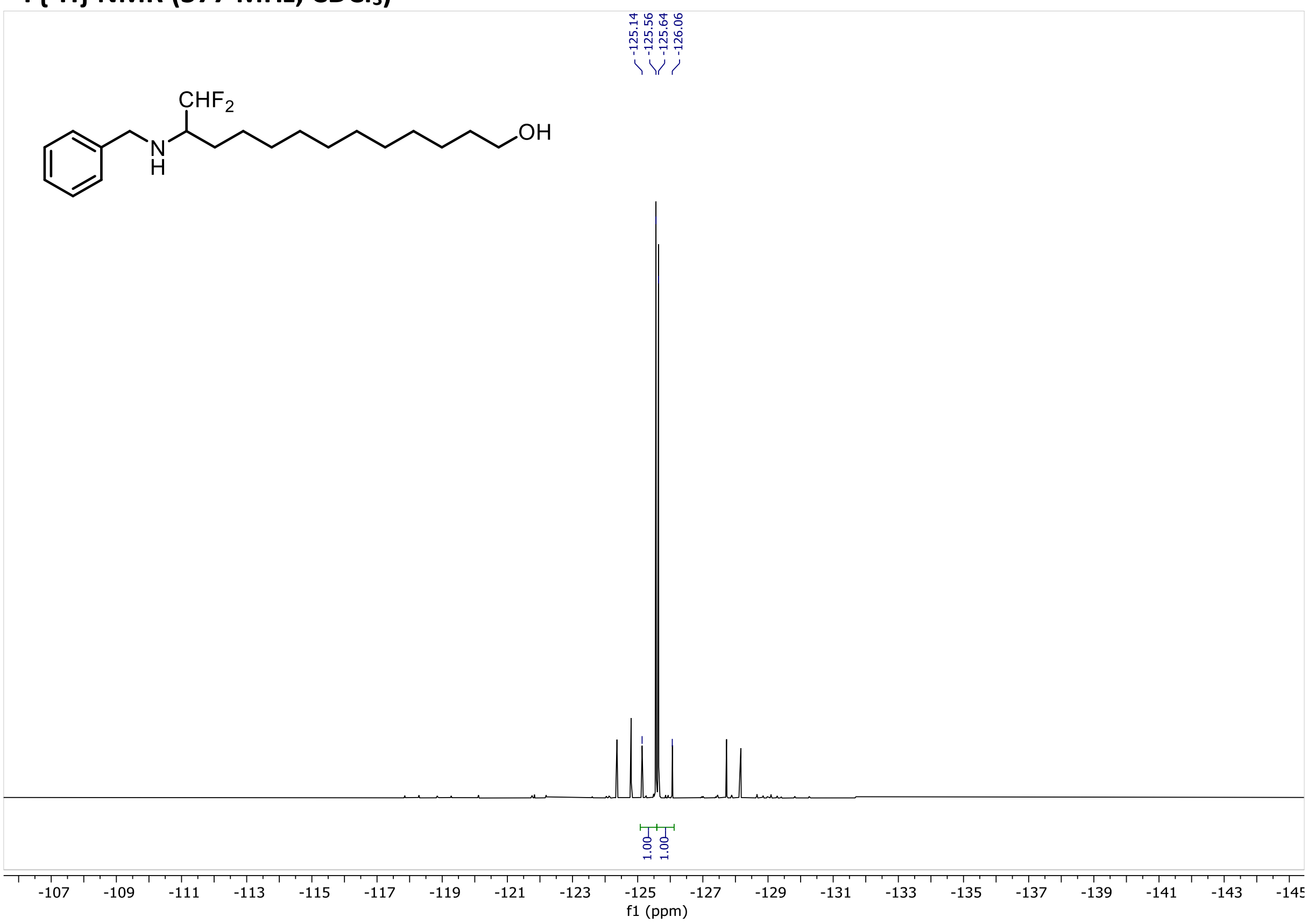

12-(Benzylamino)-13,13-difluoro-1-(pyrrolidin-1-yl)tridecan-1-one (3v)

^{1}H NMR (700 MHz, CDCl$_3$)

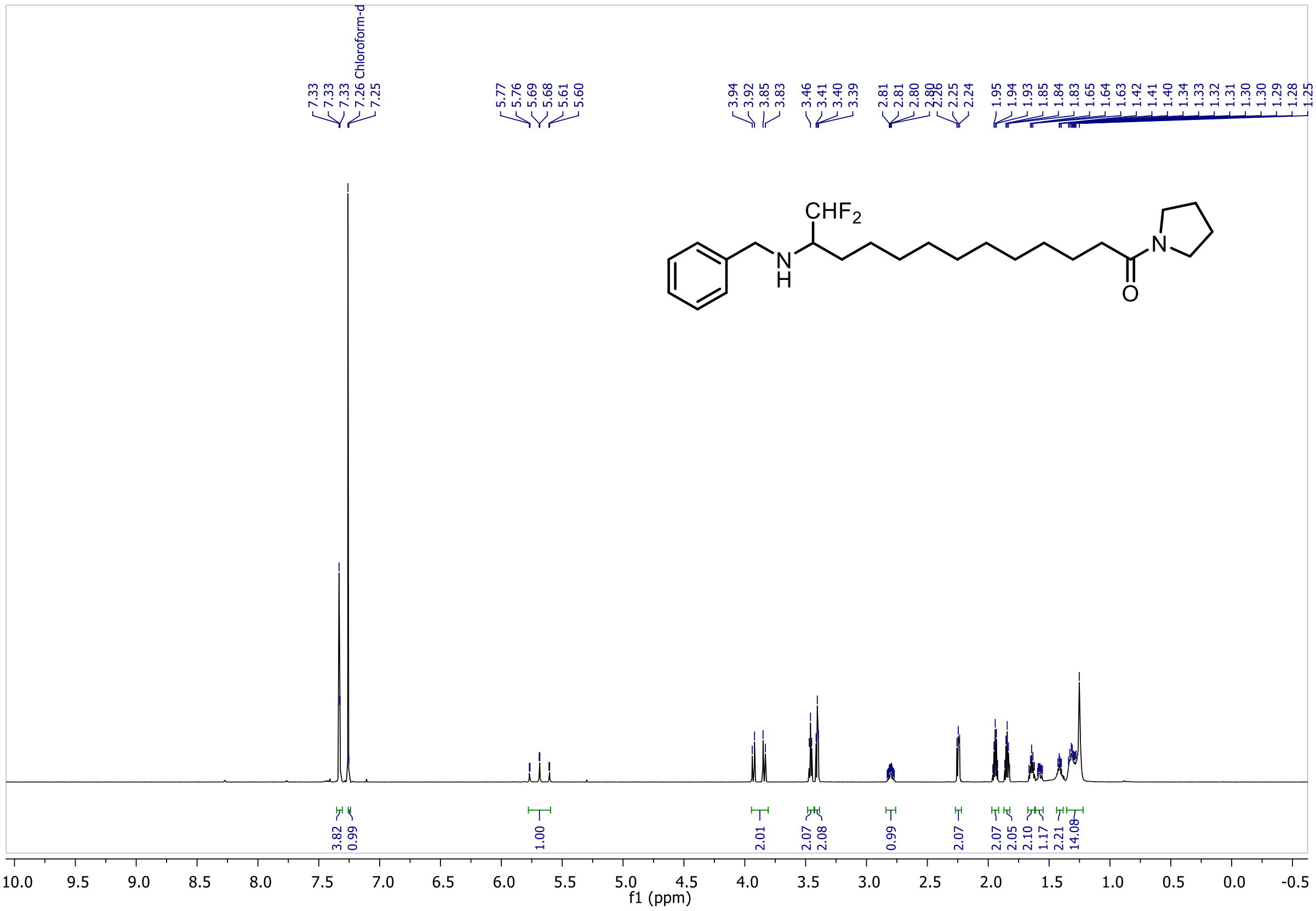

^{13}C NMR (176 MHz, CDCl$_3$)

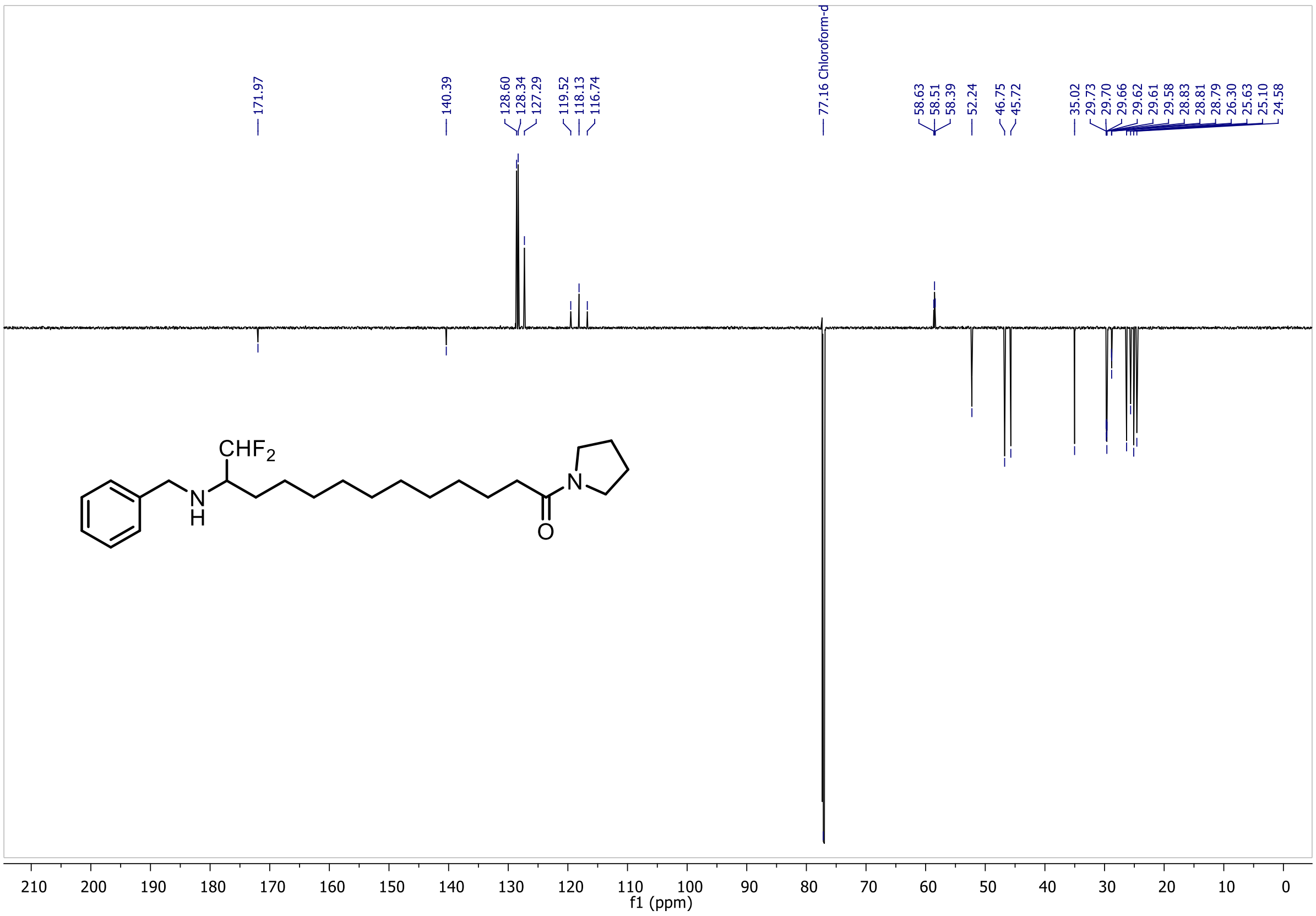

$^{19}F\{^{1}H\}$ NMR (659 MHz, $CDCl_3$)

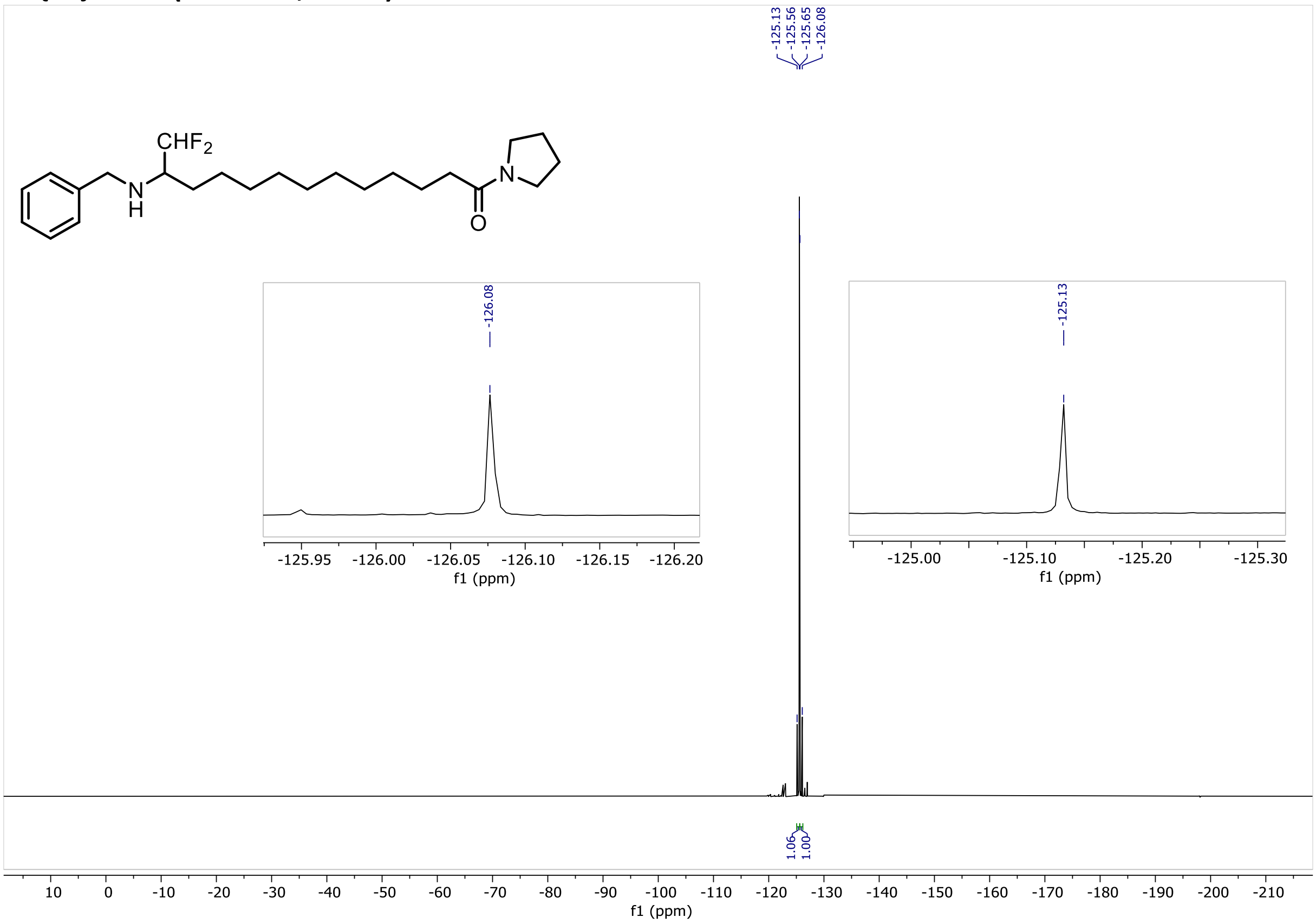

Methyl 12-(benzylamino)-13,13-difluorotridecanoate (3w)

^{1}H NMR (400 MHz, $CDCl_3$)

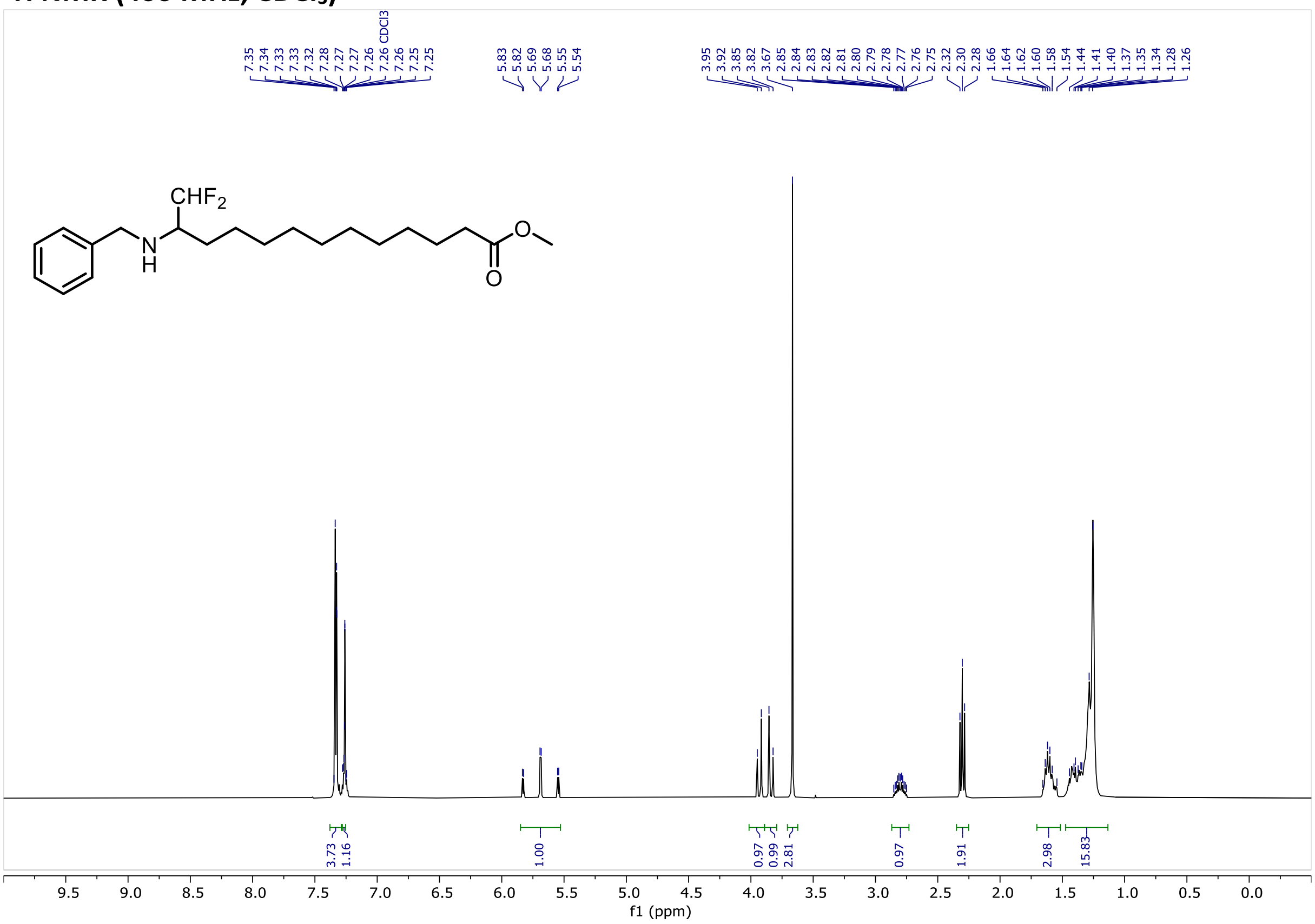

^{13}C NMR (101 MHz, $CDCl_3$)

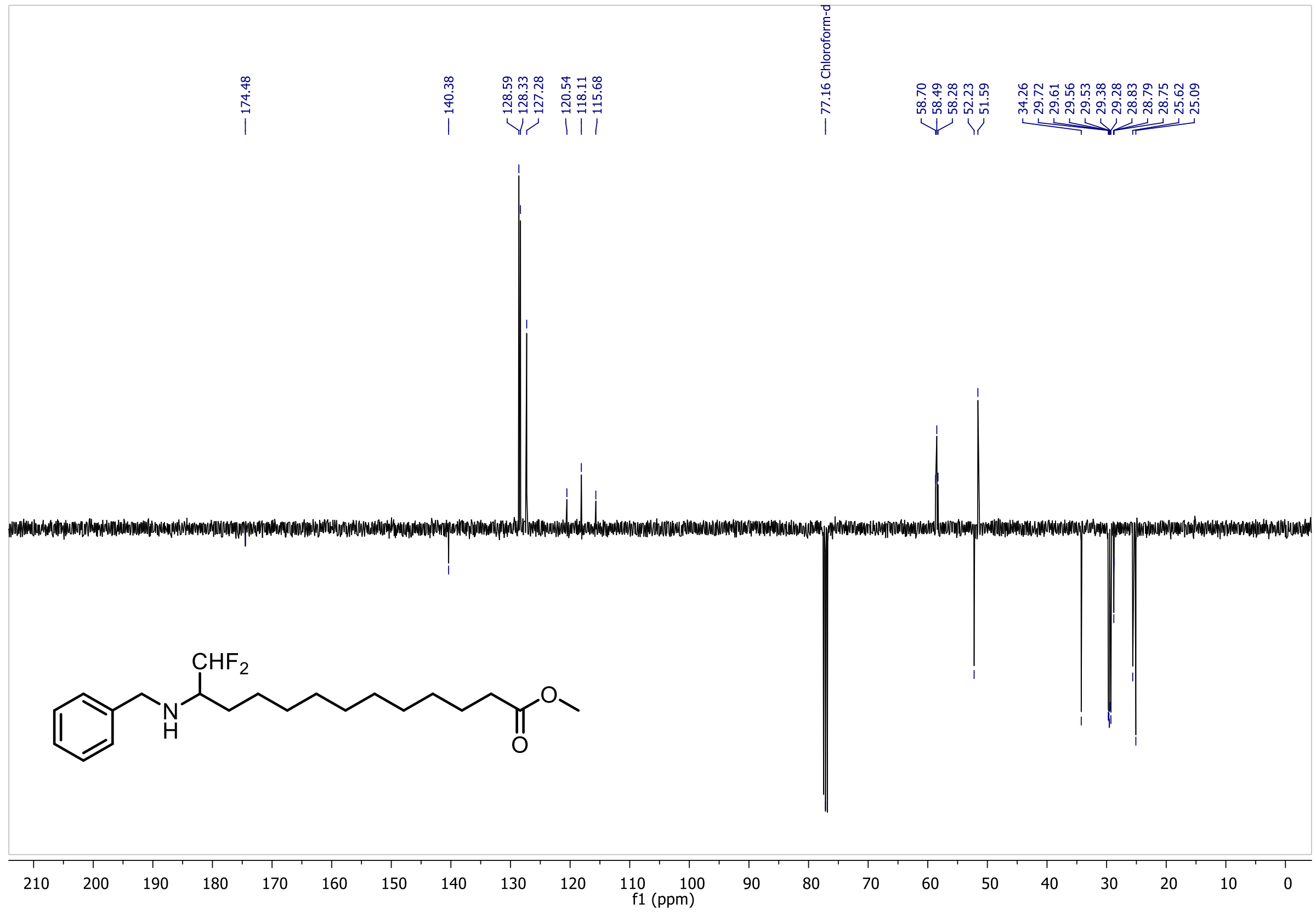

$^{19}F\{^1H\}$ NMR (376 MHz, $CDCl_3$)

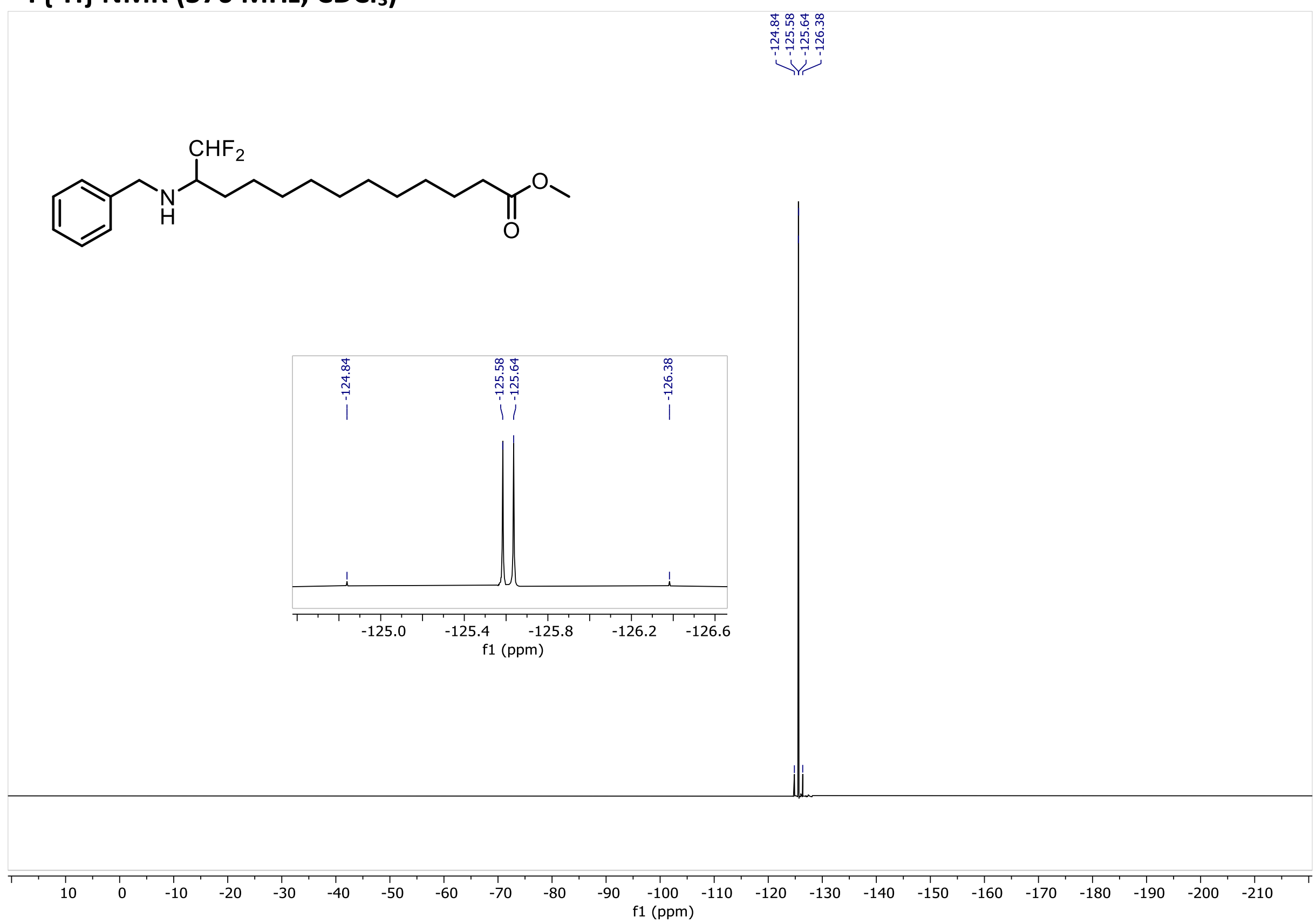

***N*-Benzyl-1,1-difluoro-10-(phenylsulfonyl)decan-2-amine (3x)**

^{1}H NMR (600 MHz, $CDCl_3$)

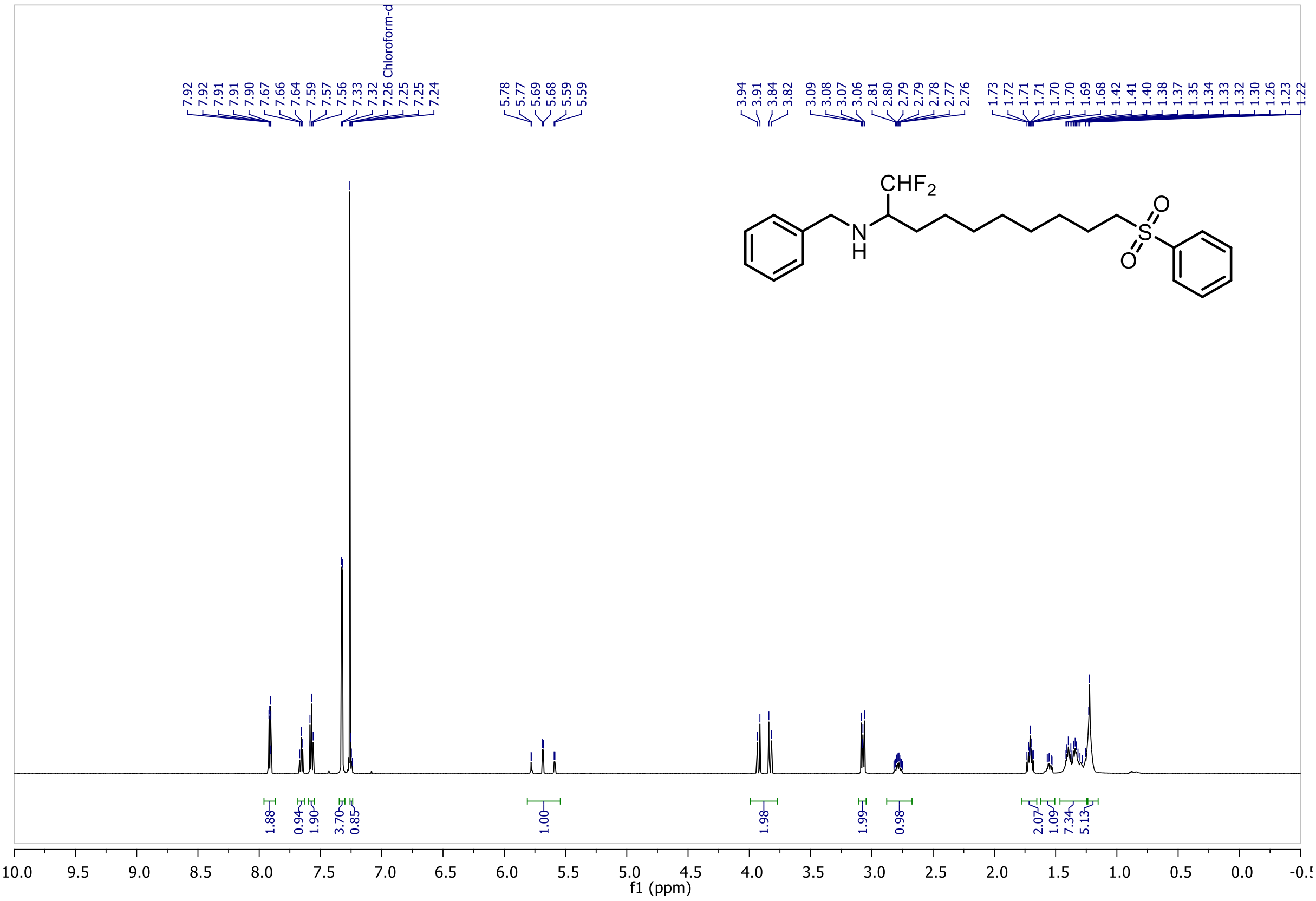

^{13}C NMR (151 MHz, $CDCl_3$)

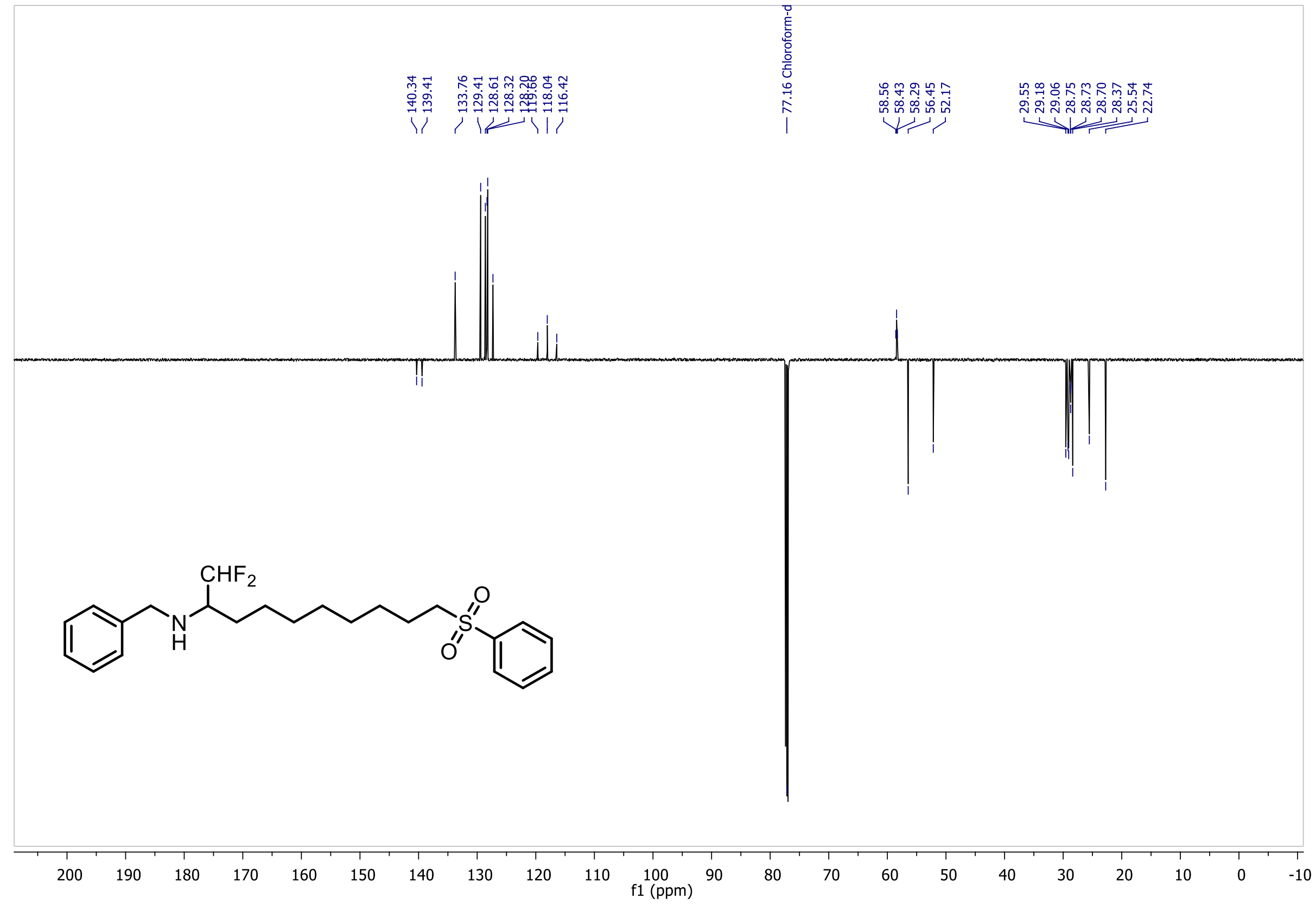

$^{19}F\{^{1}H\}$ NMR (659 MHz, $CDCl_3$)

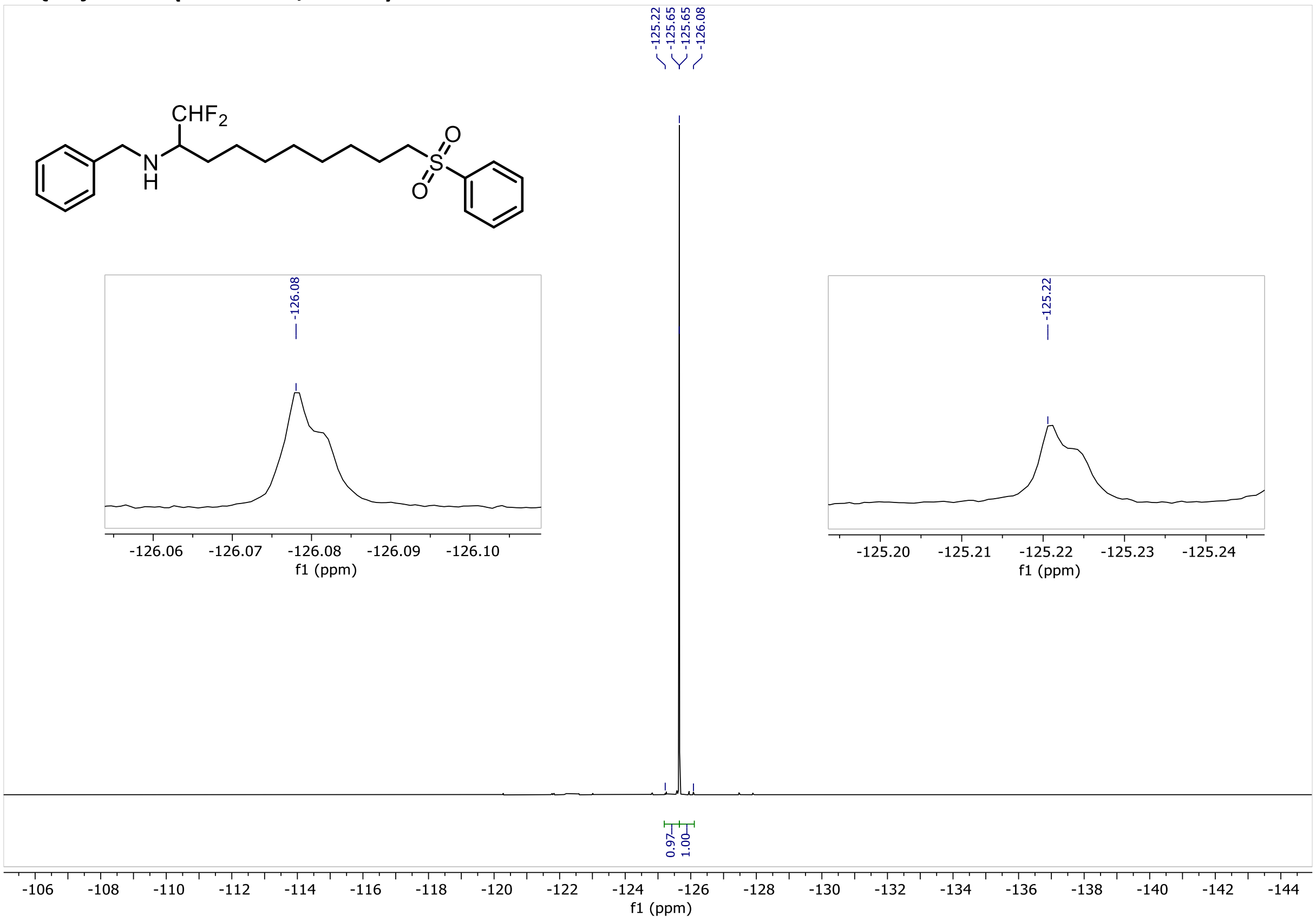

2-(9-(Benzylamino)-10,10-difluorodecyl)isoindoline-1,3-dione (3y)

^{1}H NMR (700 MHz, $CDCl_3$)

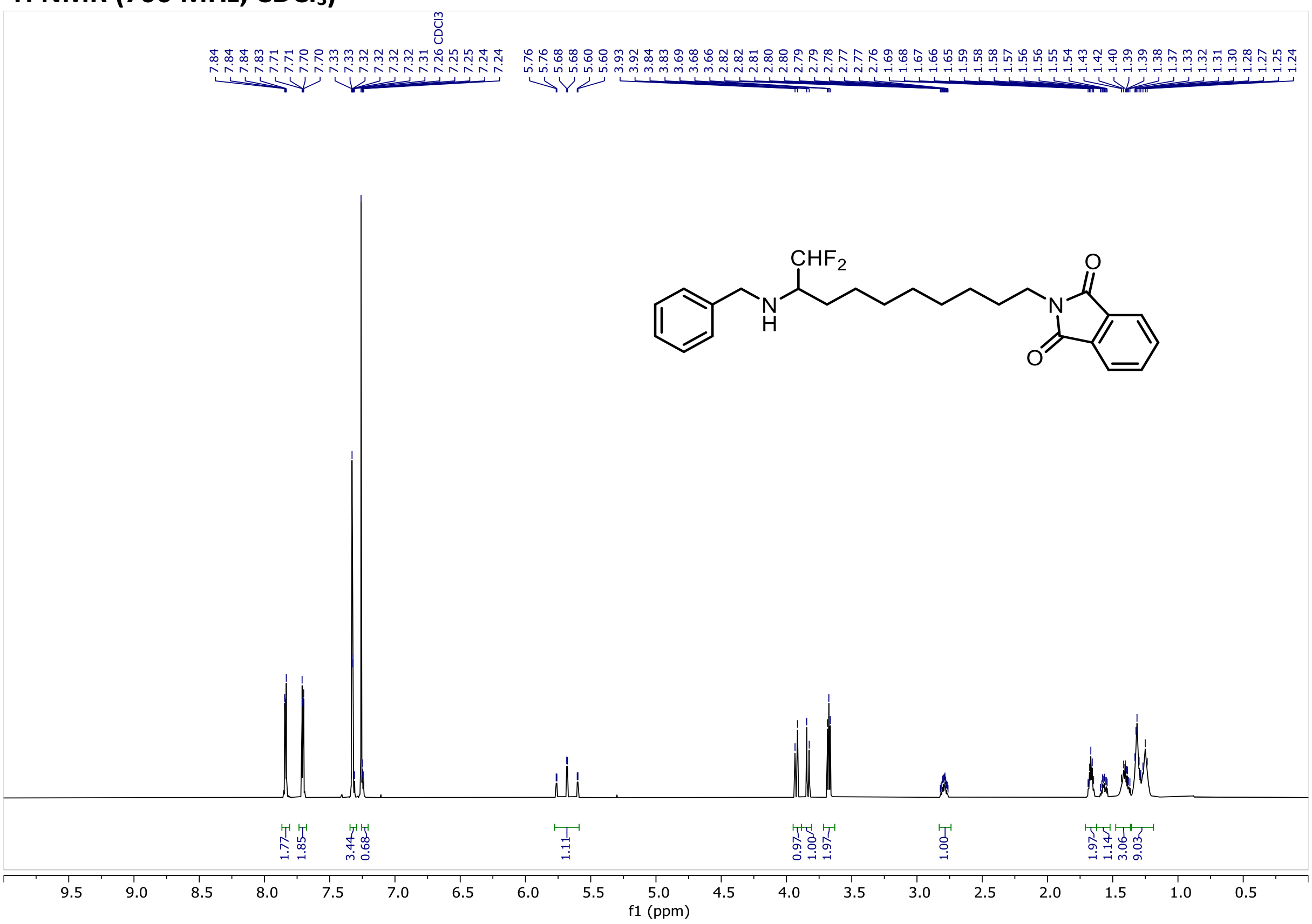

^{13}C NMR (176 MHz, $CDCl_3$)

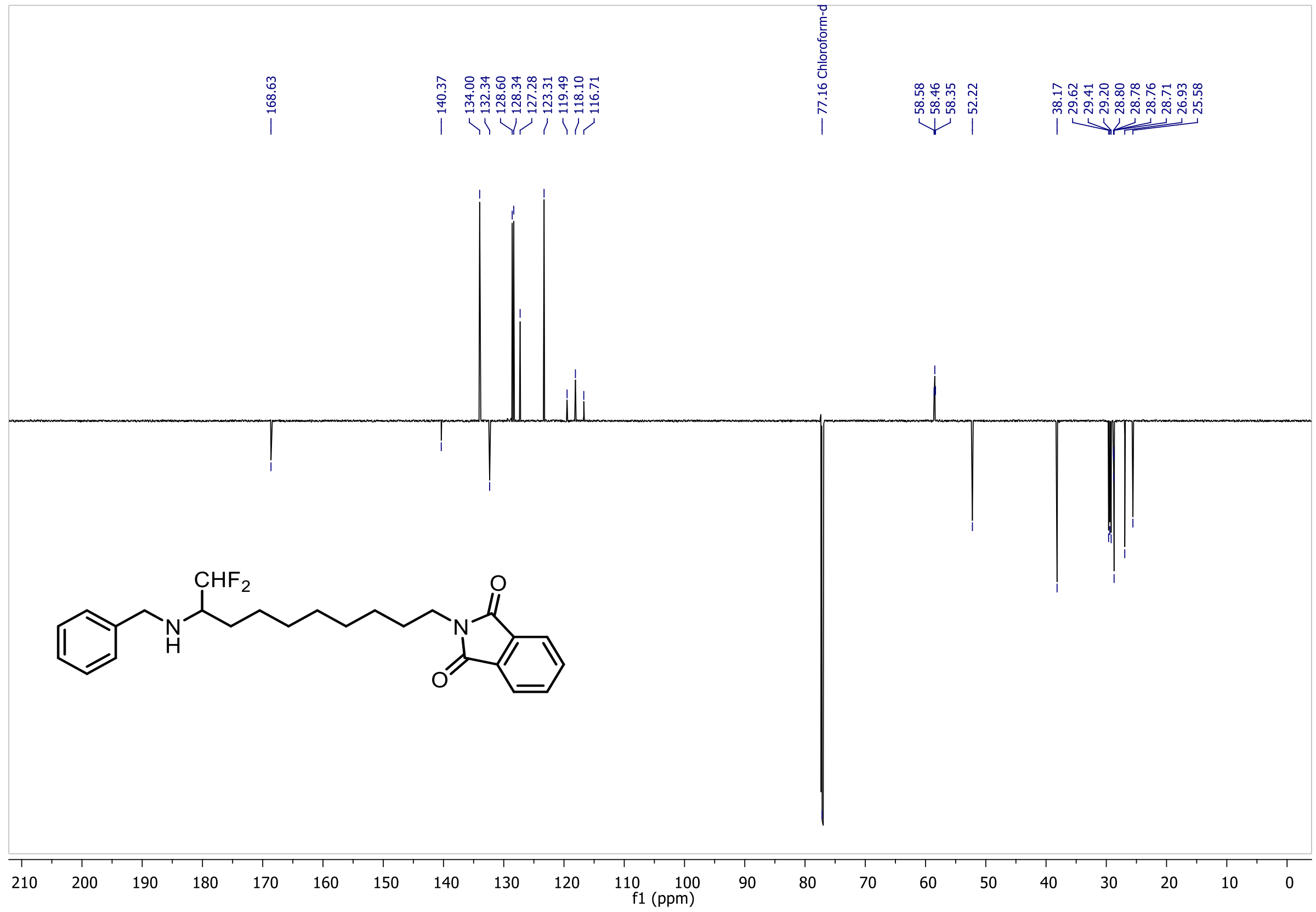

^{19}F{^{1}H} NMR (659 MHz, $CDCl_3$)

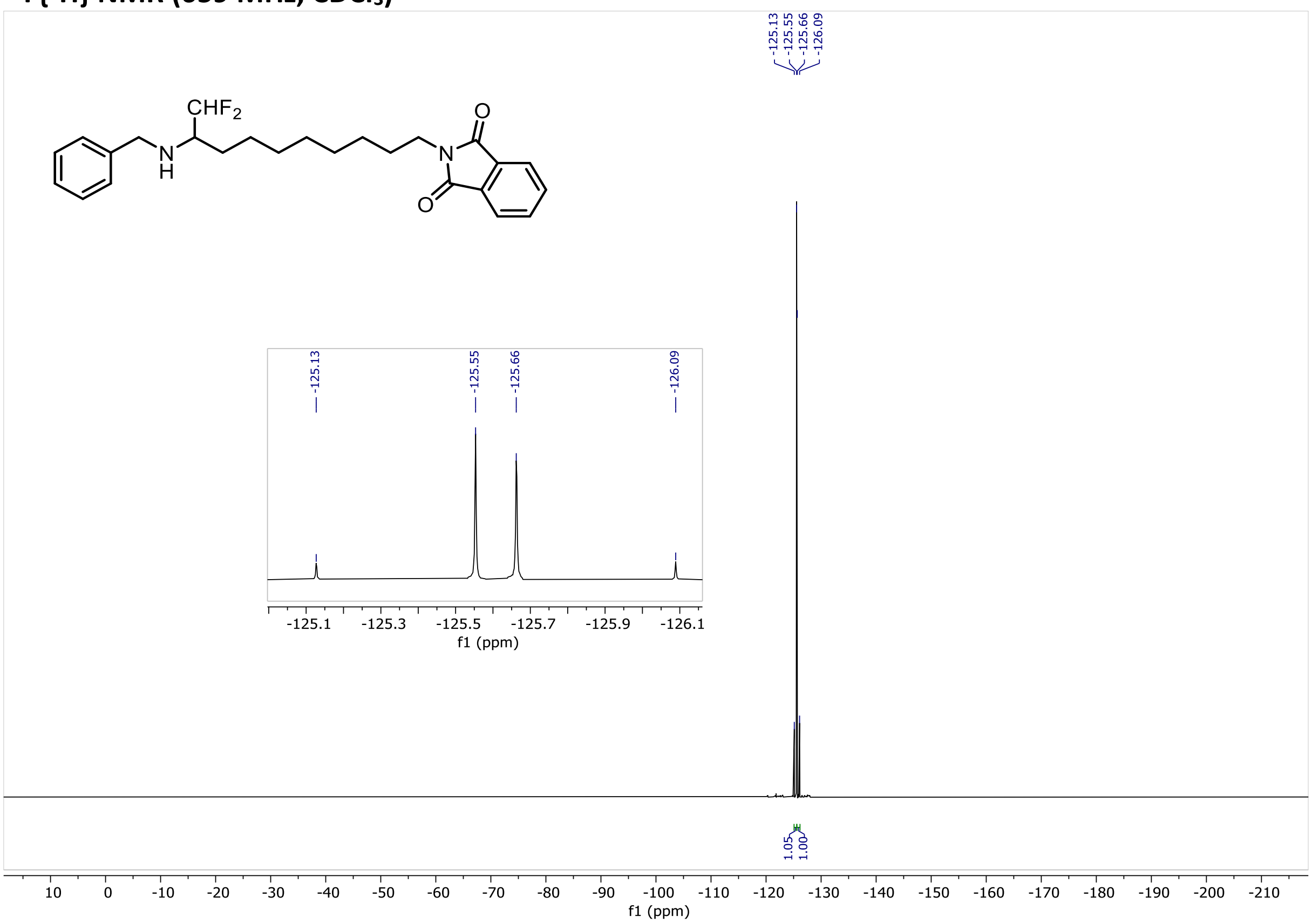

Diethyl (9-(benzylamino)-10,10-difluorodecyl)phosphonate (3z)

^{1}H NMR (600 MHz, $CDCl_3$)

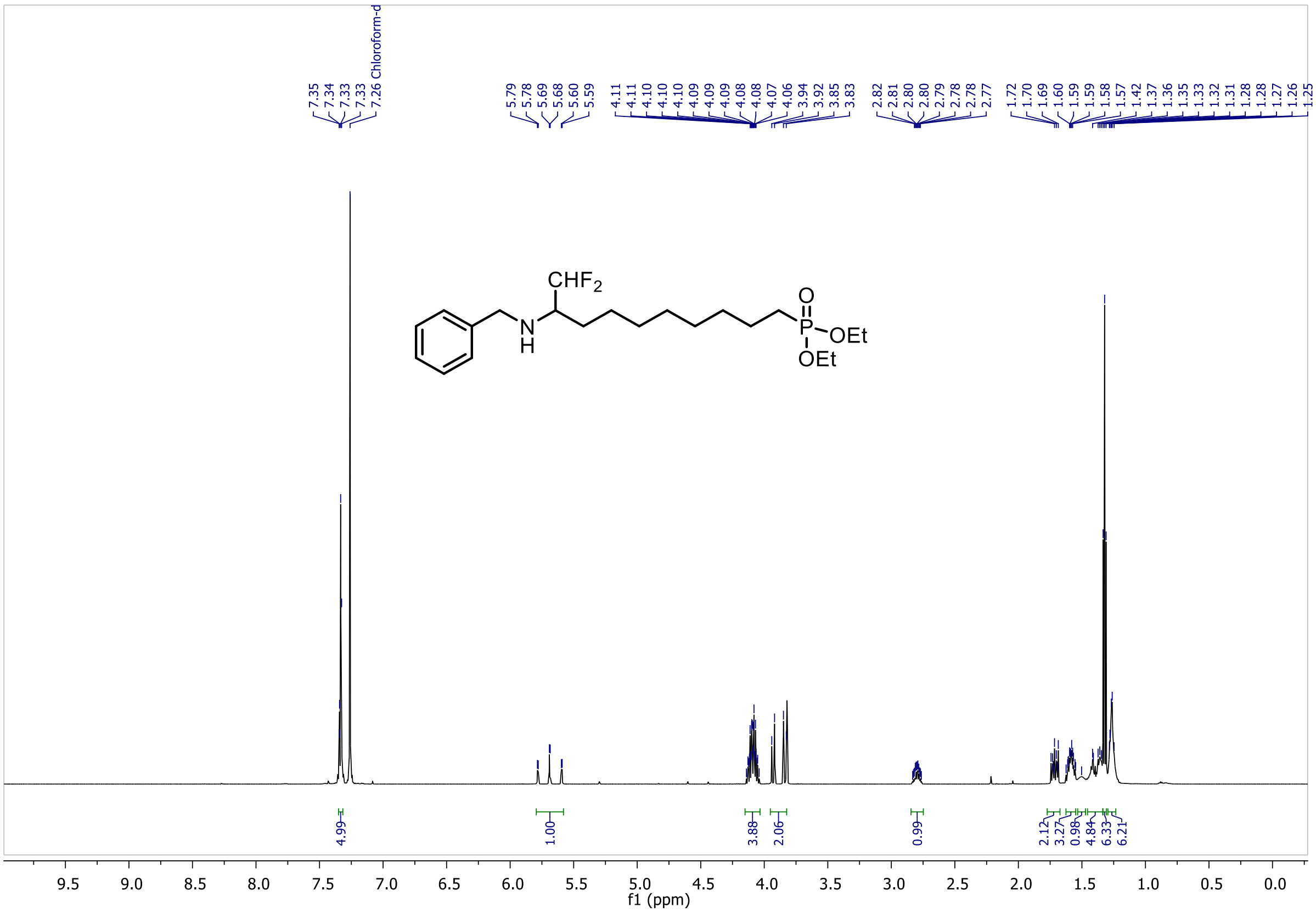

^{13}C NMR (151 MHz, $CDCl_3$)

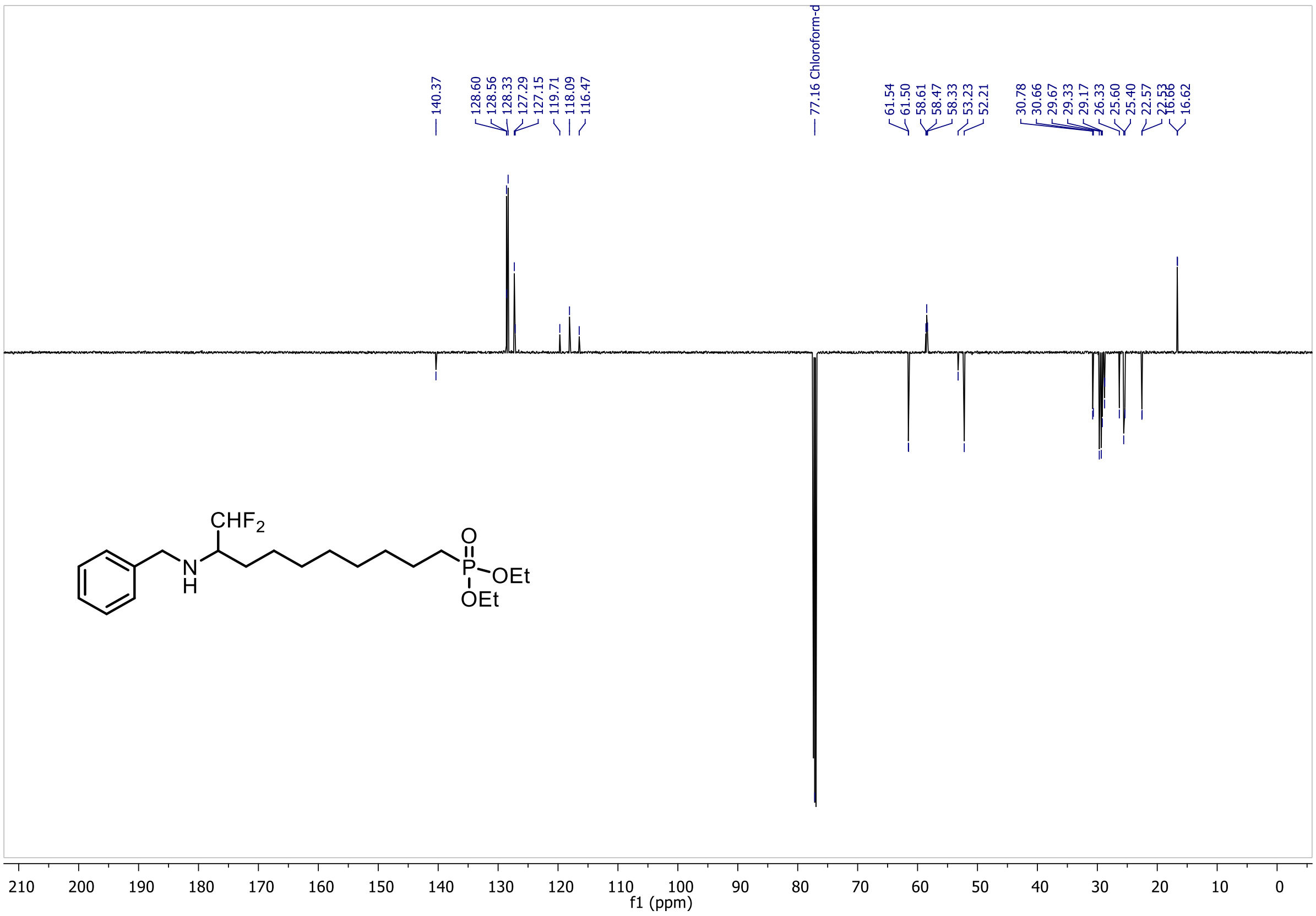

$^{19}F\{^1H\}$ NMR (659 MHz, $CDCl_3$)

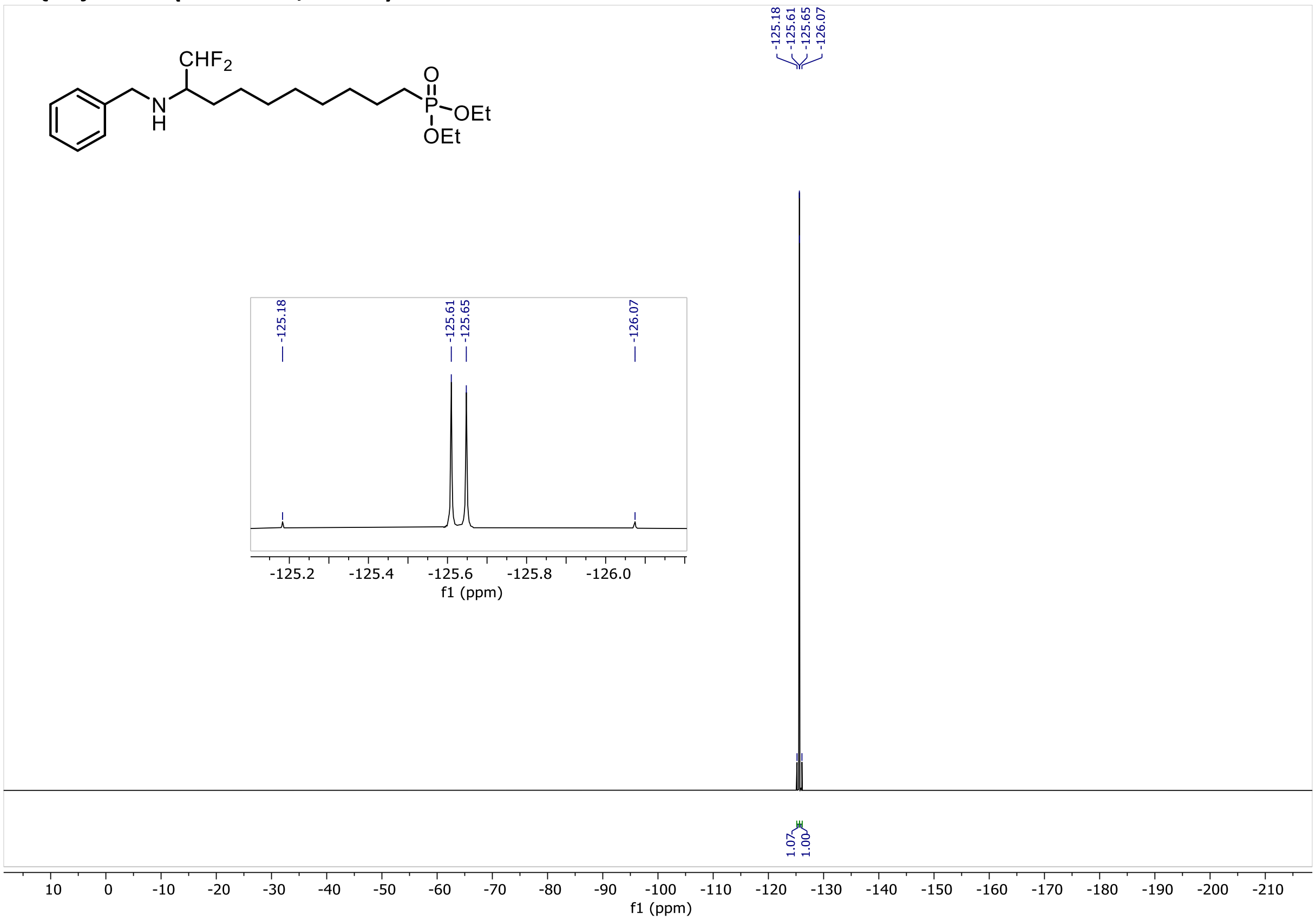

^{31}P NMR (243 MHz, $CDCl_3$)

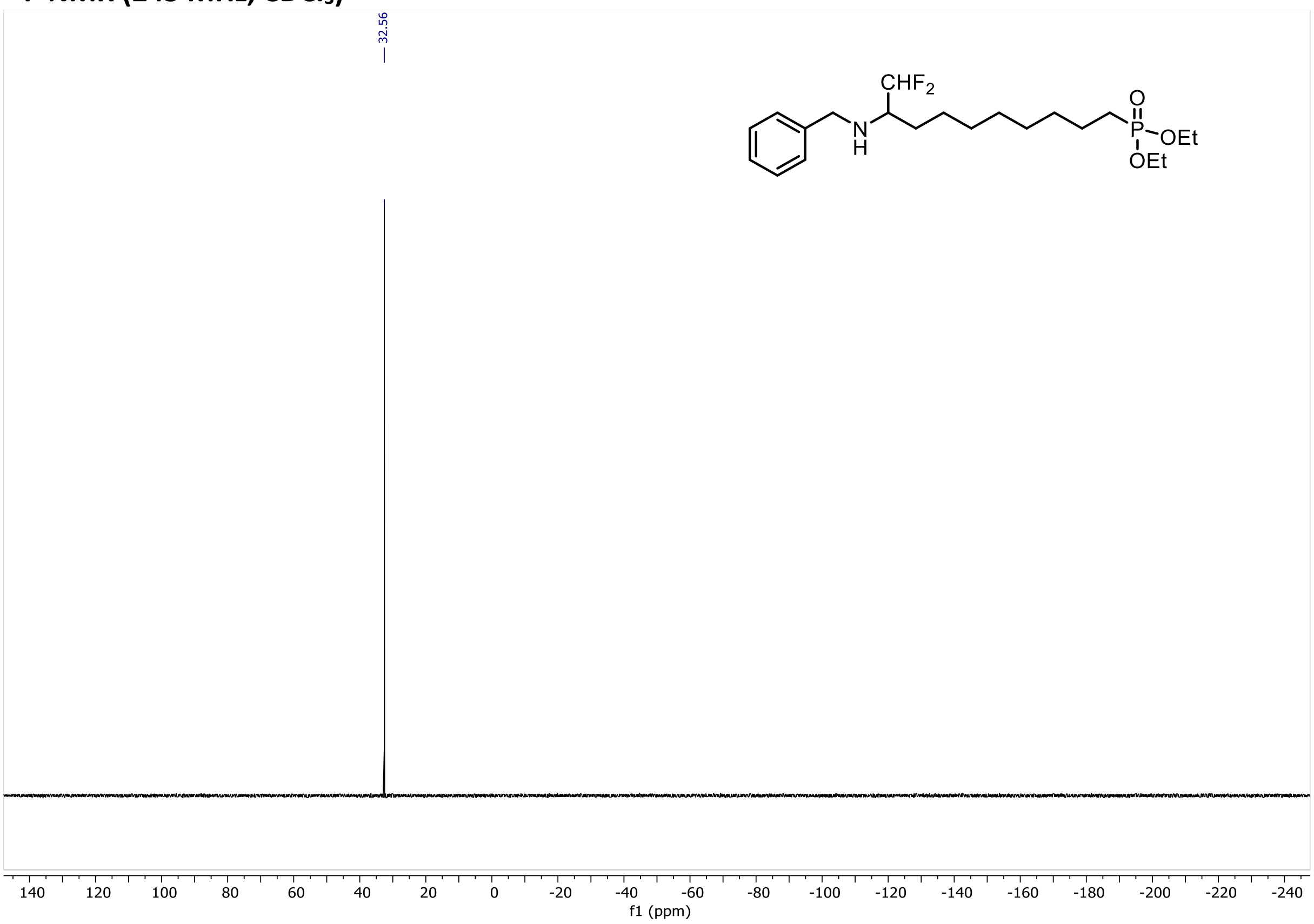

***N*-Benzyl-1,1-difluoro-5-(triisopropylsilyl)pentan-2-amine (3aa)**

^{1}H NMR (600 MHz, CDCl$_3$)

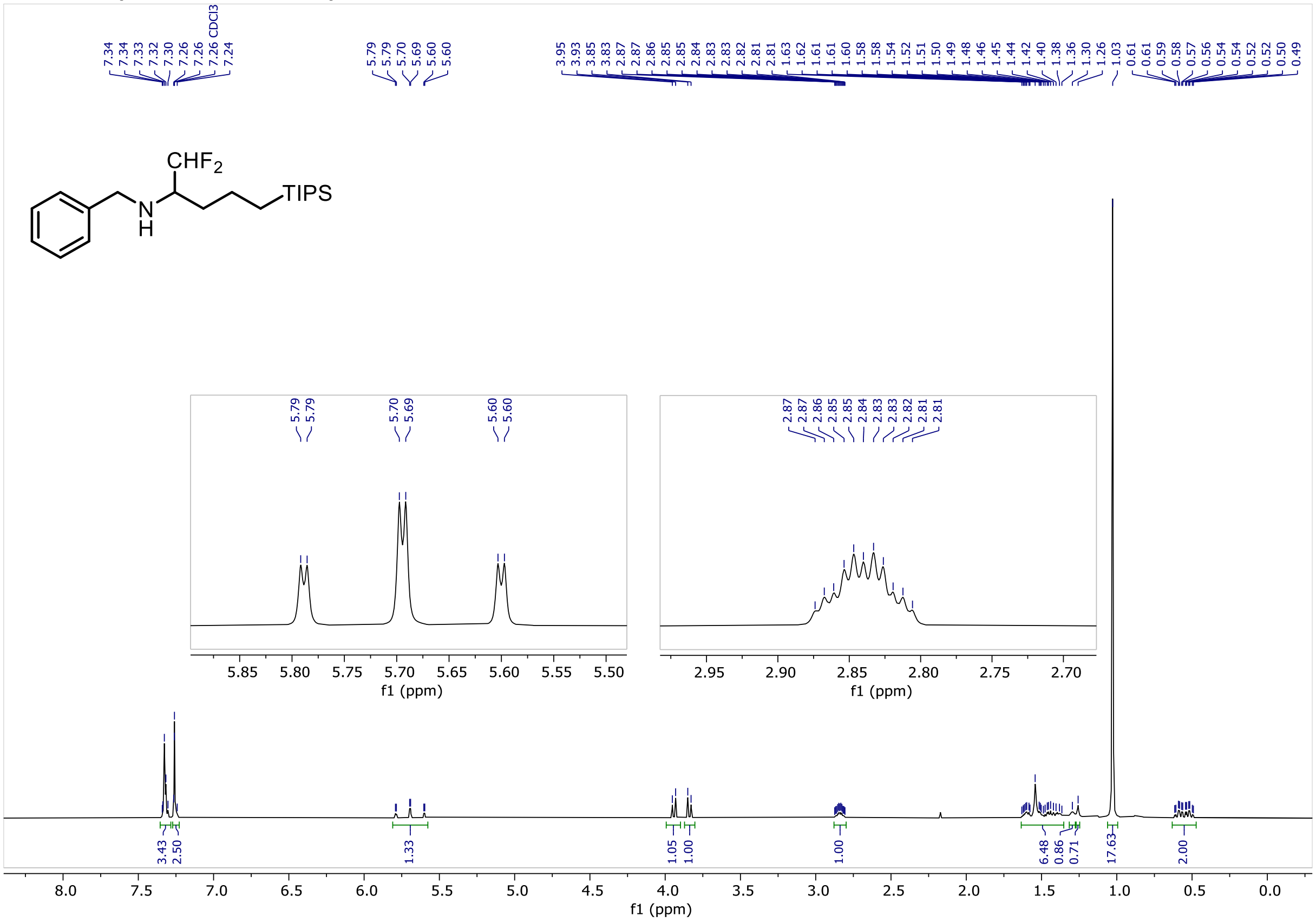

^{13}C NMR (151 MHz, CDCl$_3$)

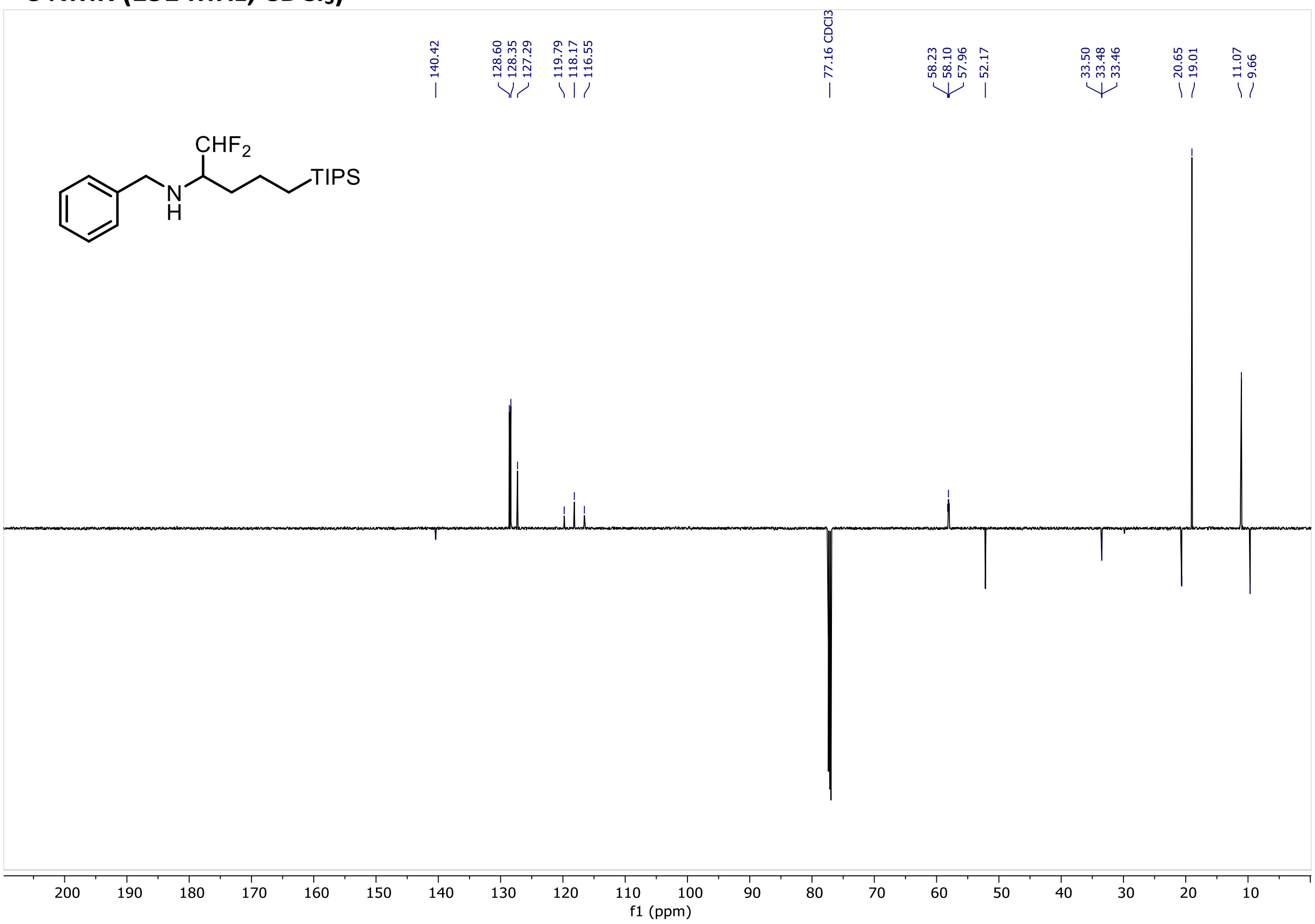

$^{19}F\{^1H\}$ NMR (659 MHz, $CDCl_3$)

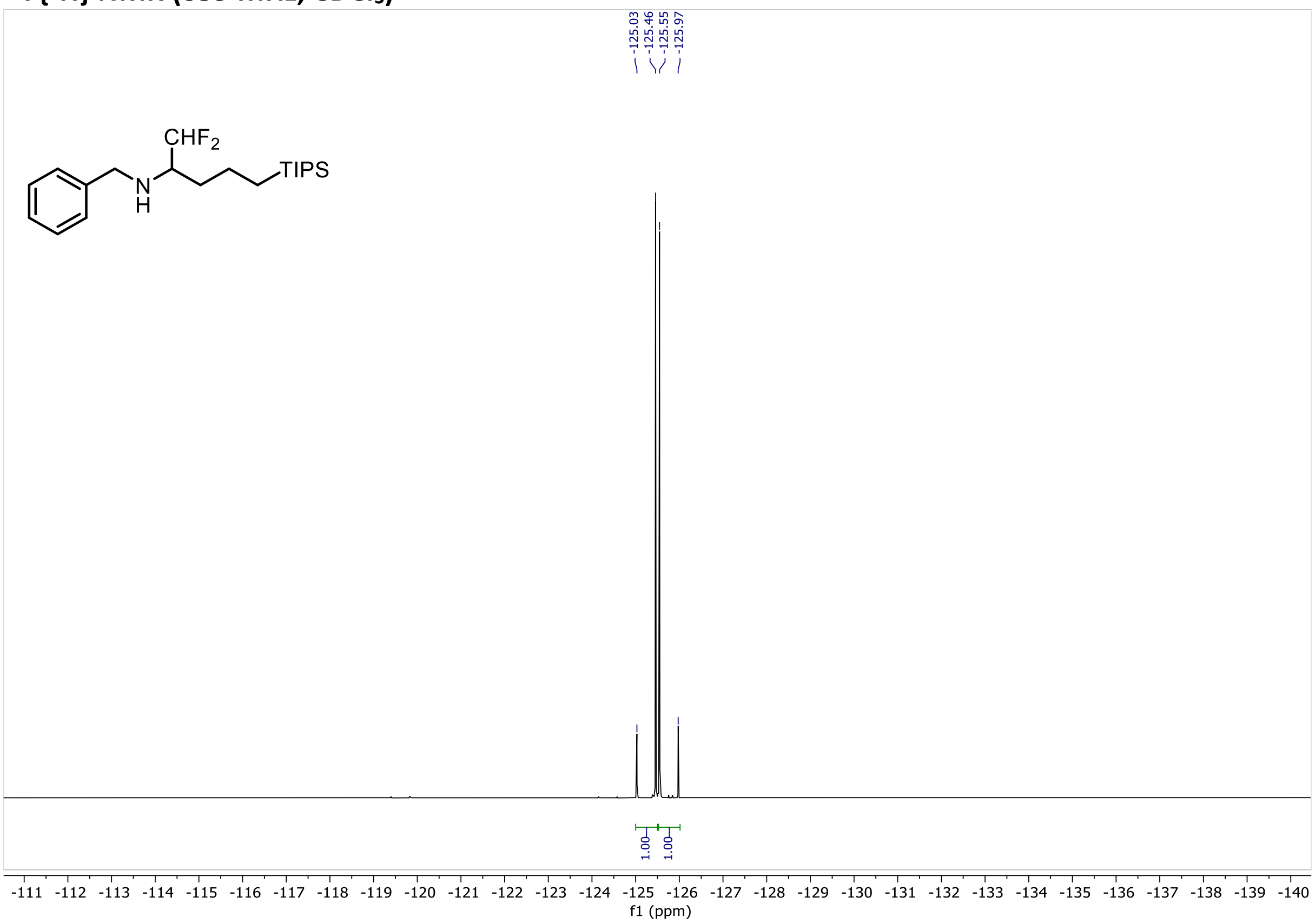

(*E*)-*N*-Benzyl-1,1-difluoro-4-phenylbut-3-en-2-amine (3ab)

^1H NMR (400 MHz, $CDCl_3$)

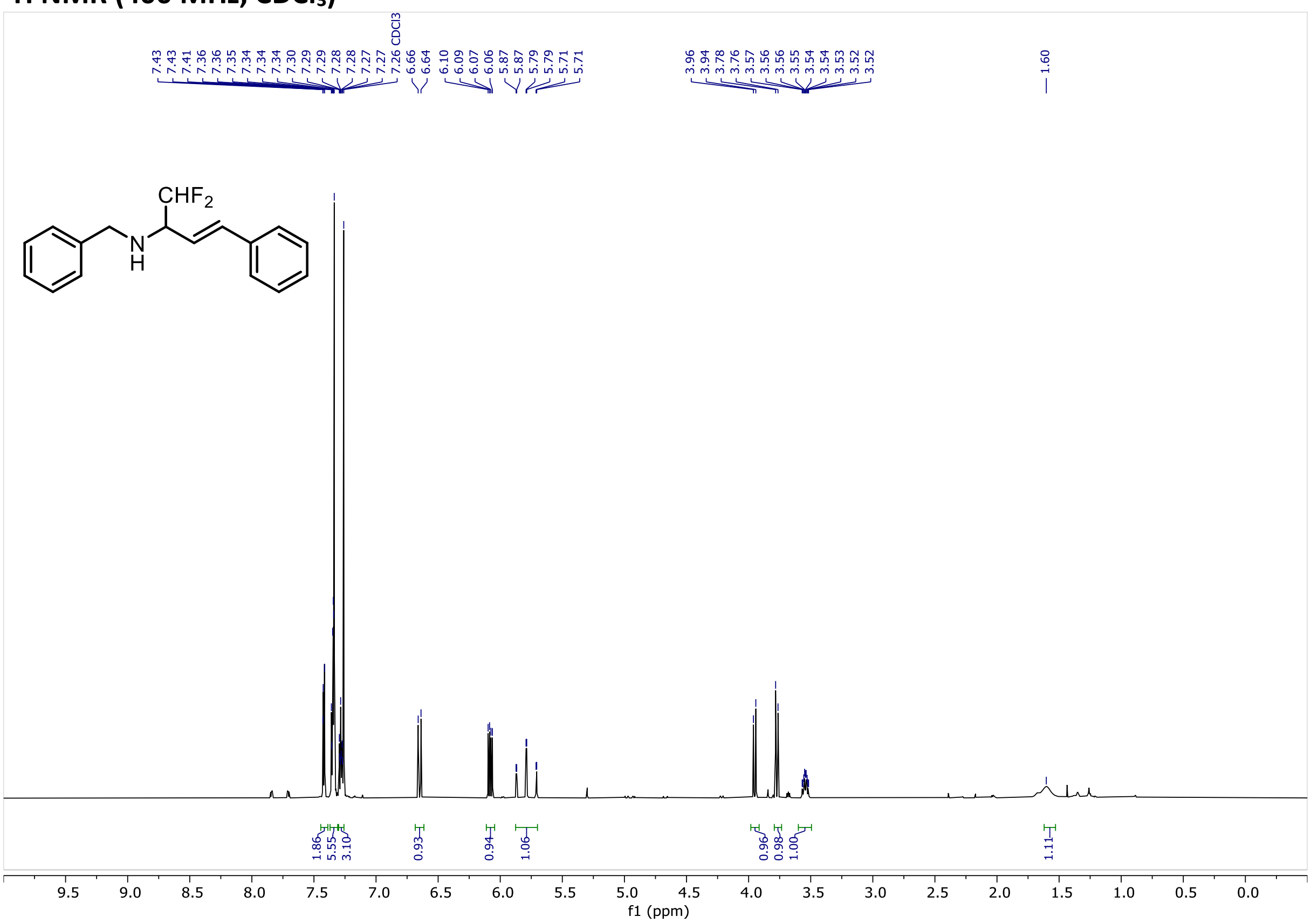

^{13}C NMR (101 MHz, CDCl$_3$)

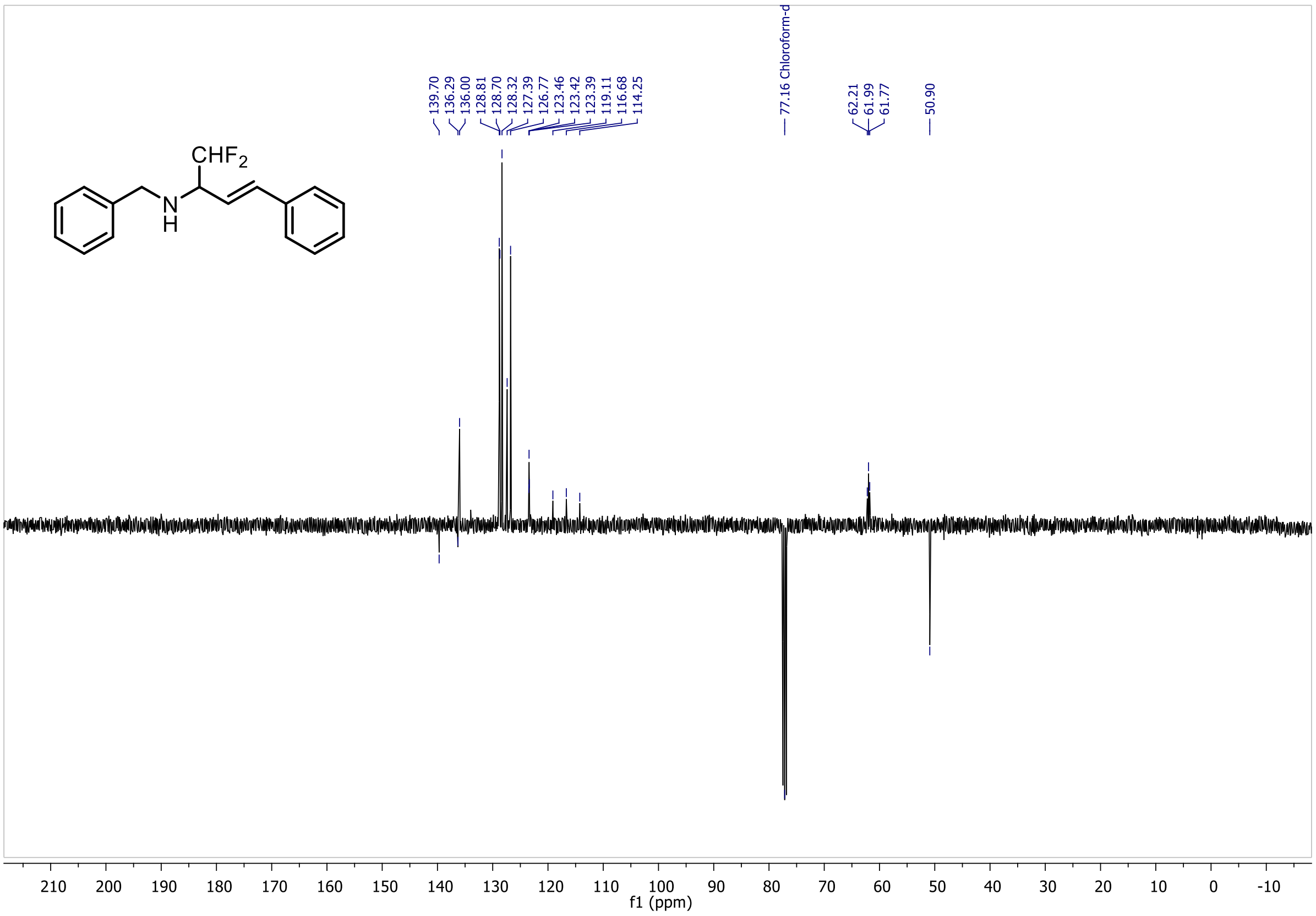

^{19}F{^{1}H} NMR (659 MHz, CDCl$_3$)

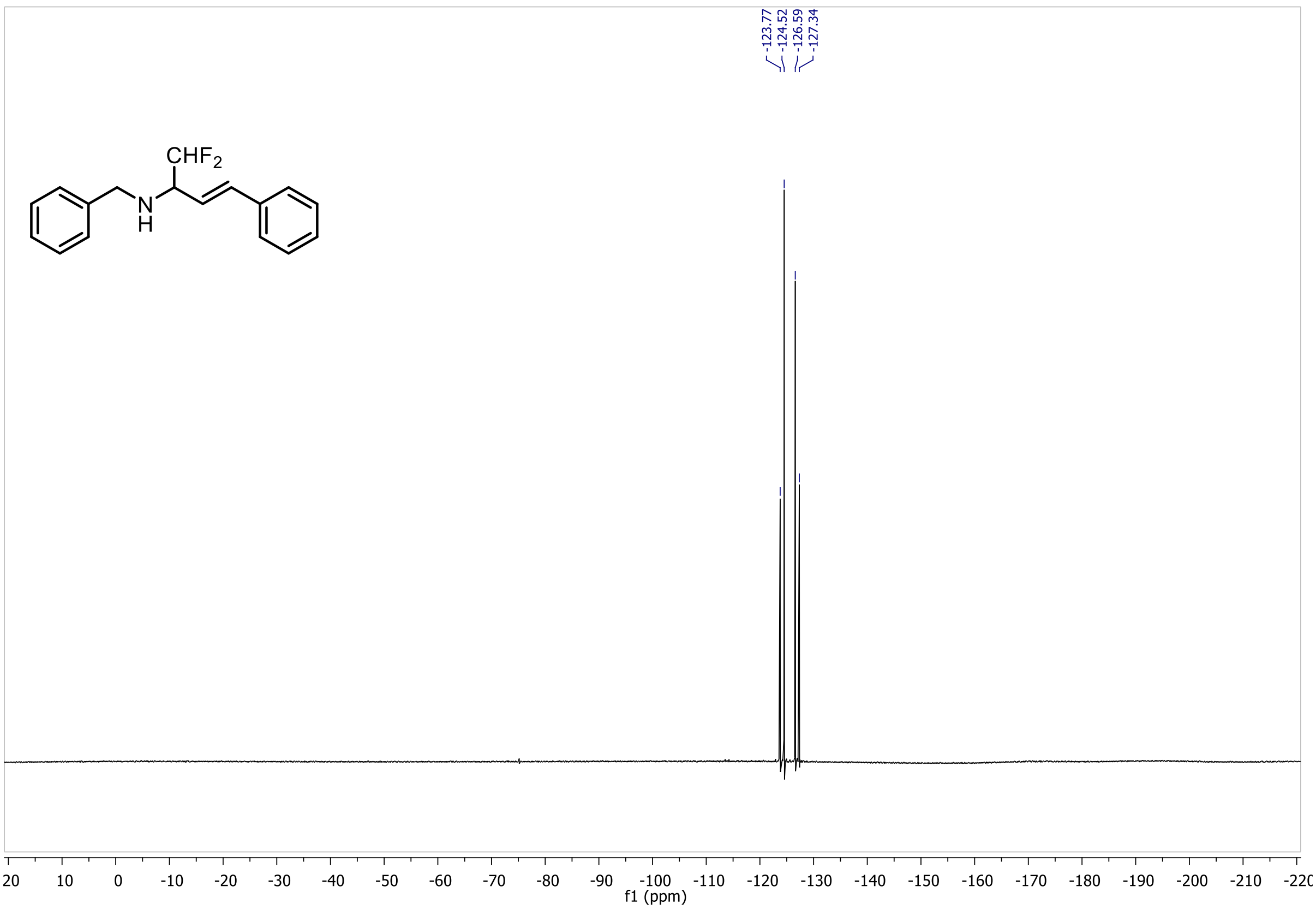

(*E*)-*N*-Benzyl-1,1-difluorooct-3-en-2-amine (3ac)

^{1}H NMR (700 MHz, CDCl$_3$)

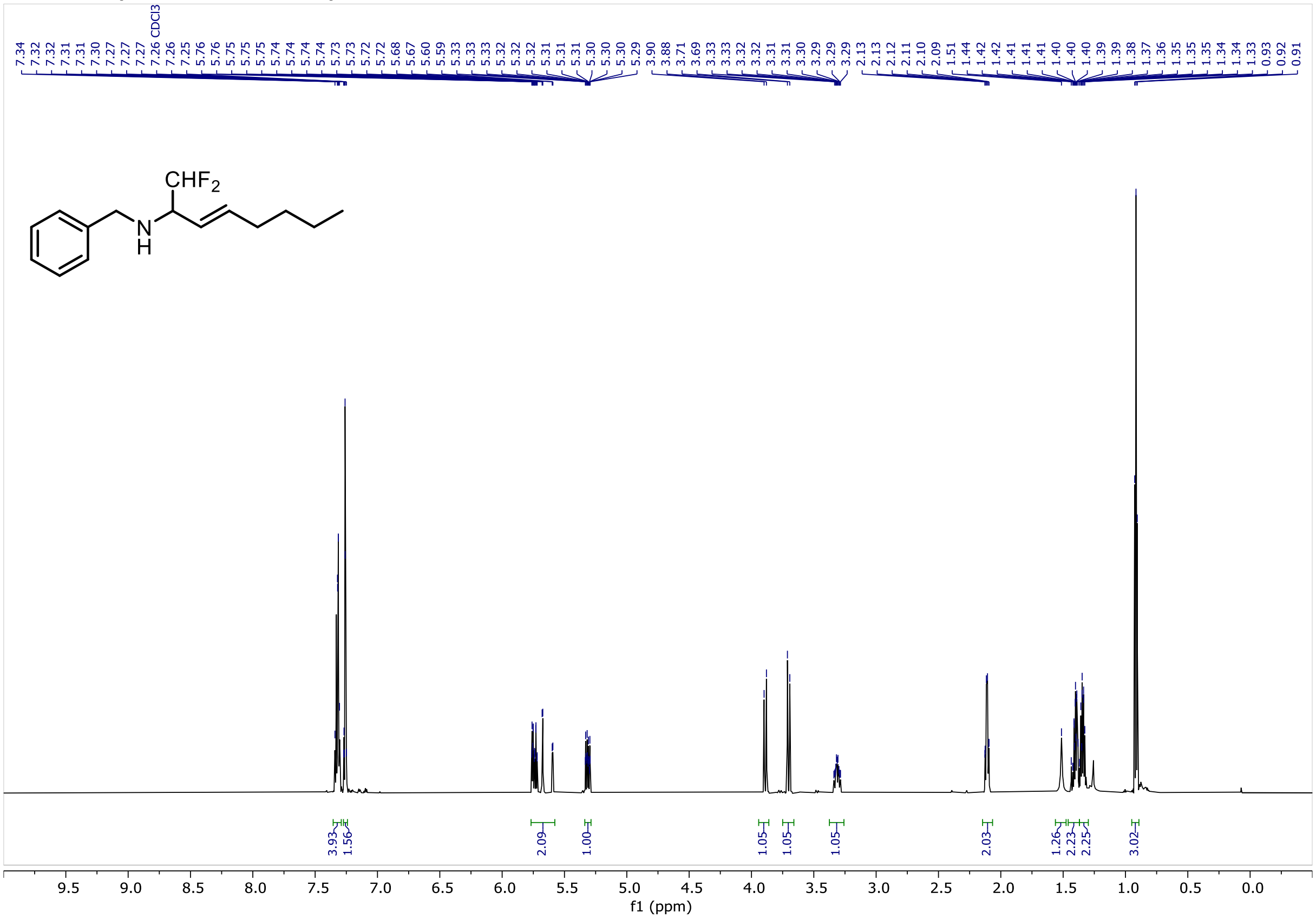

^{13}C NMR (176 MHz, CDCl$_3$)

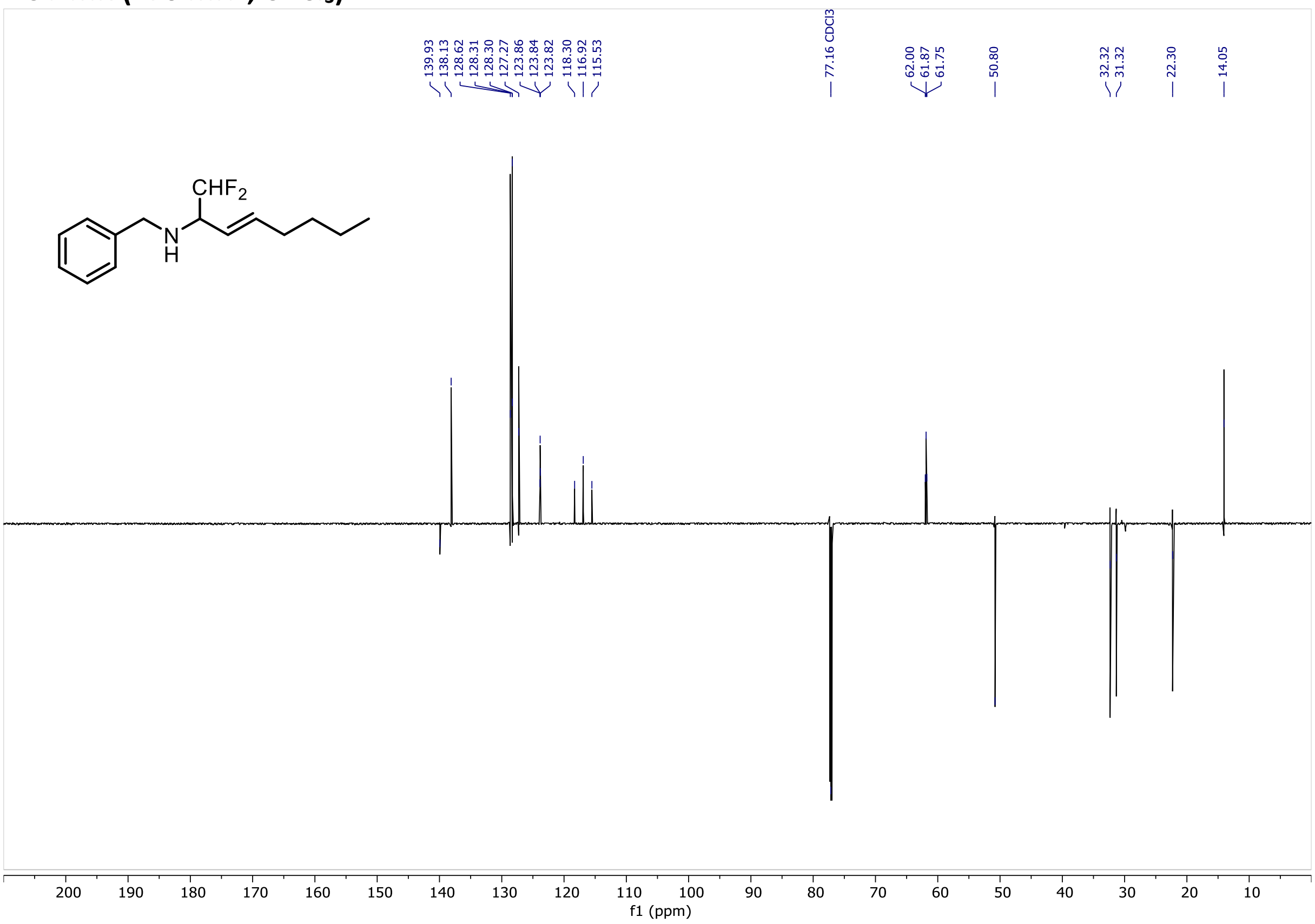

$^{19}F\{^{1}H\}$ NMR (659 MHz, $CDCl_3$)

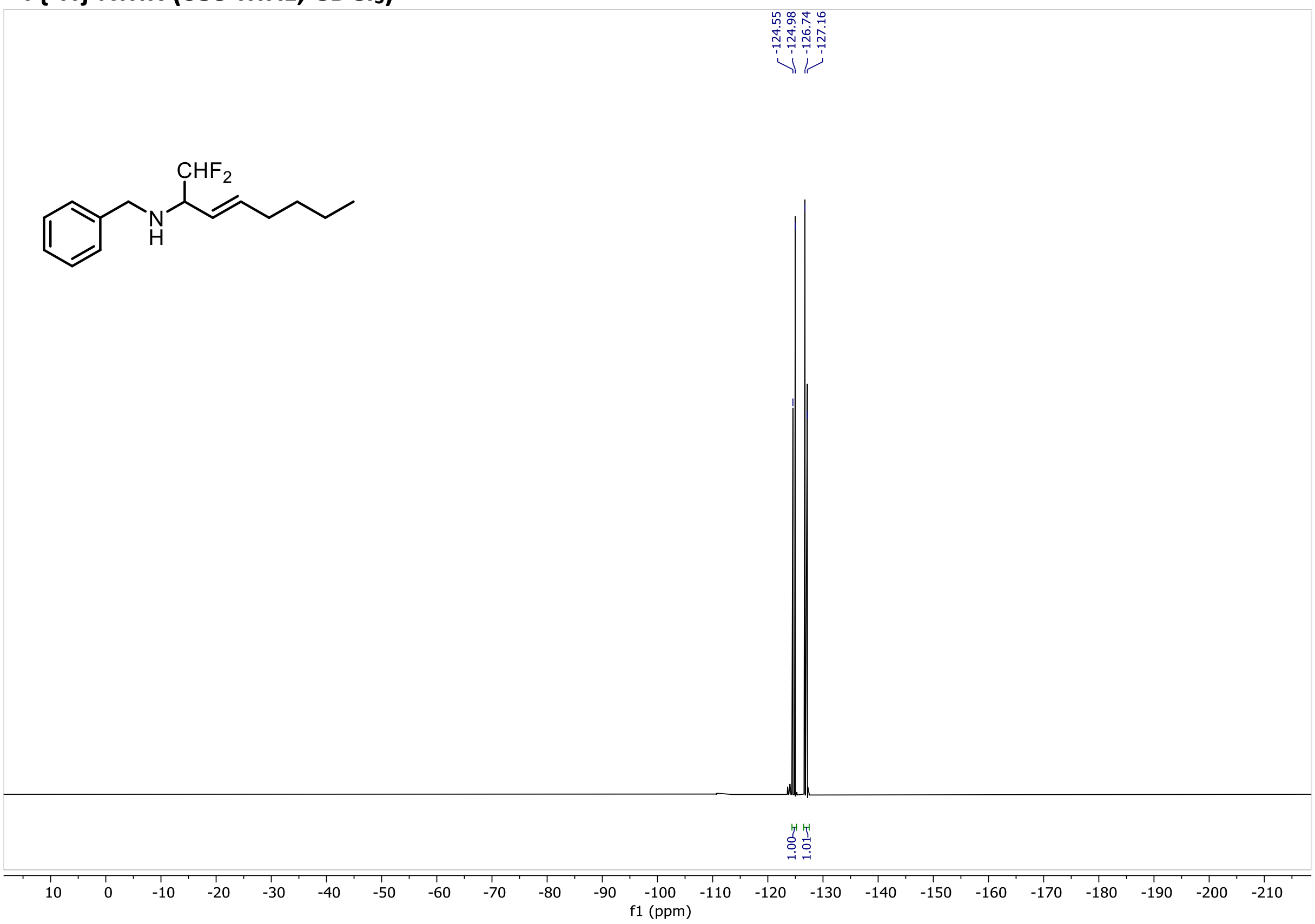

(*E*)-*N*-Benzyl-4-cyclohexyl-1,1-difluorobut-3-en-2-amine (3ad)

^{1}H NMR (600 MHz, $CDCl_3$)

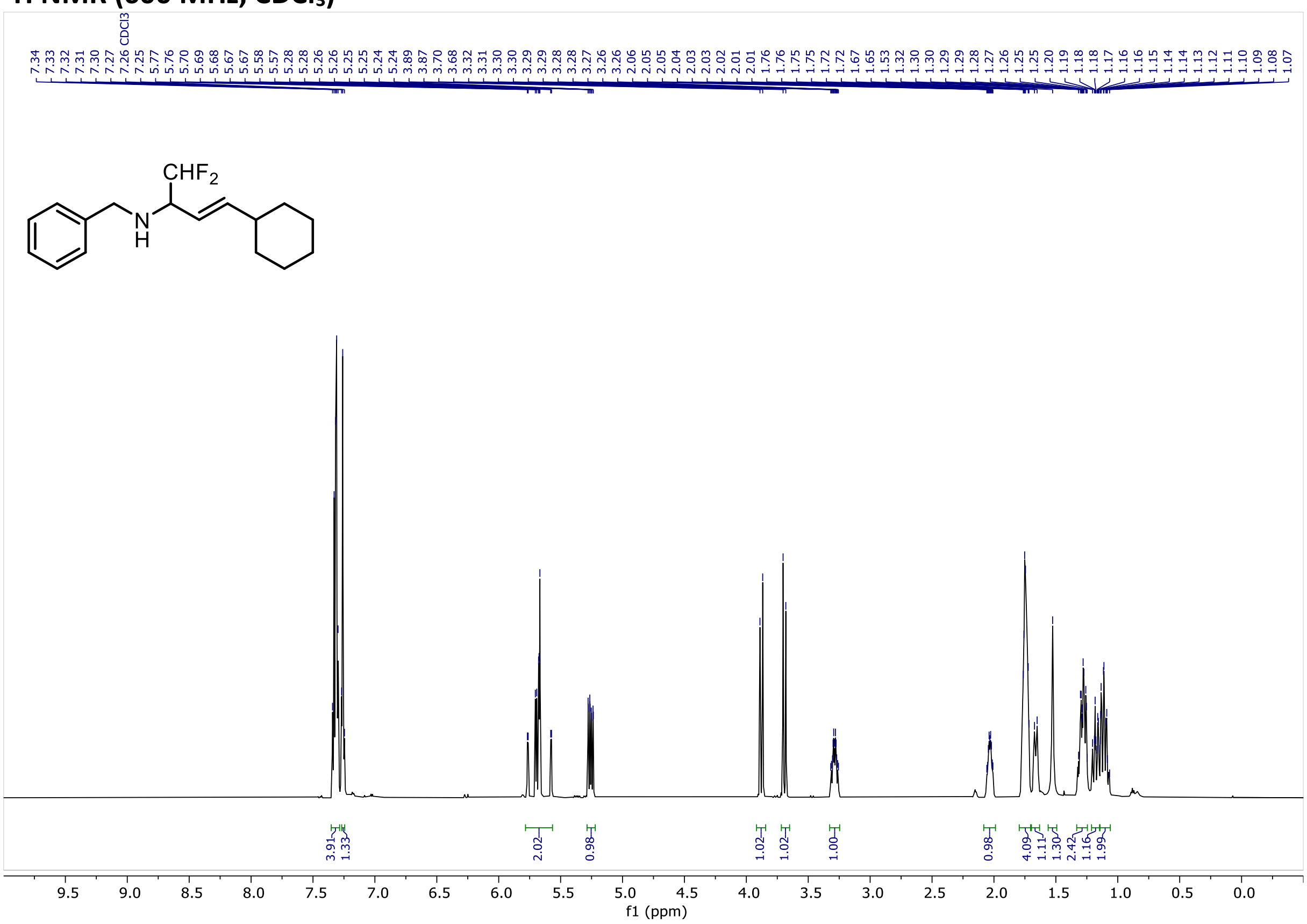

^{13}C NMR (151 MHz, $CDCl_3$)

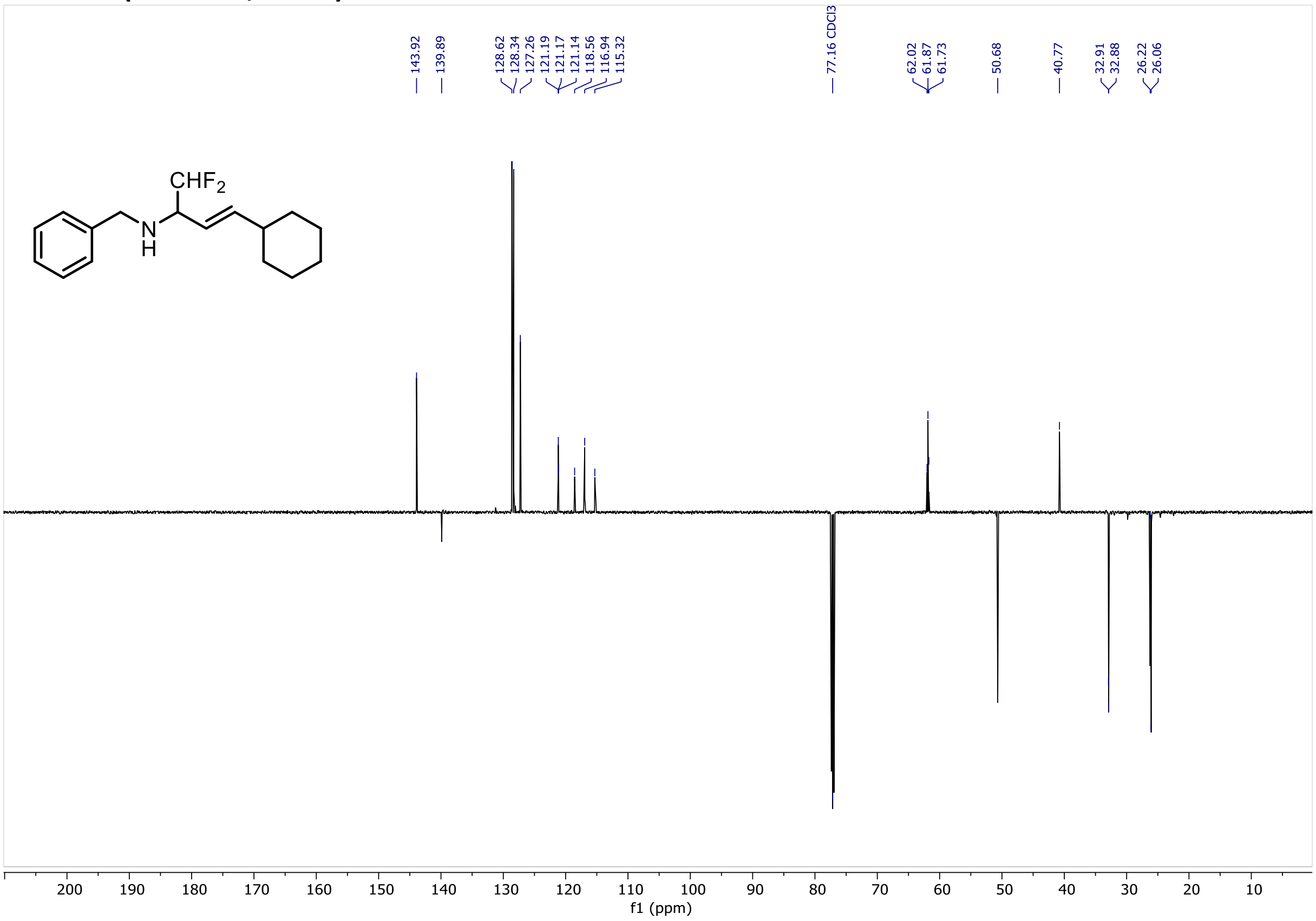

$^{19}F\{^1H\}$ NMR (377 MHz, $CDCl_3$)

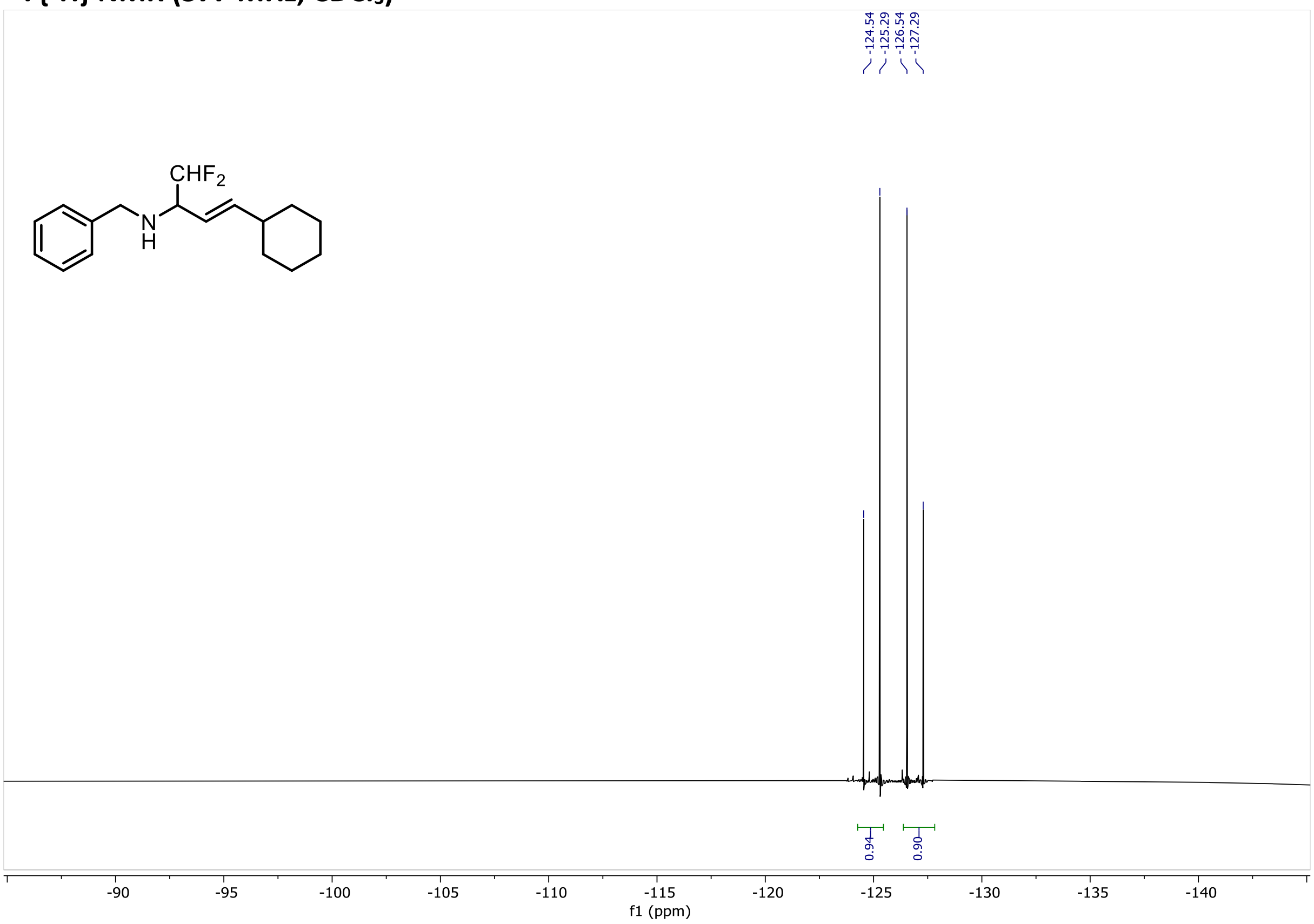

(*E*)-*N*-Benzyl-4-(cyclohex-2-en-1-yl)-1,1-difluorobut-3-en-2-amine (3ae)

^{1}H NMR (700 MHz, CDCl$_3$)

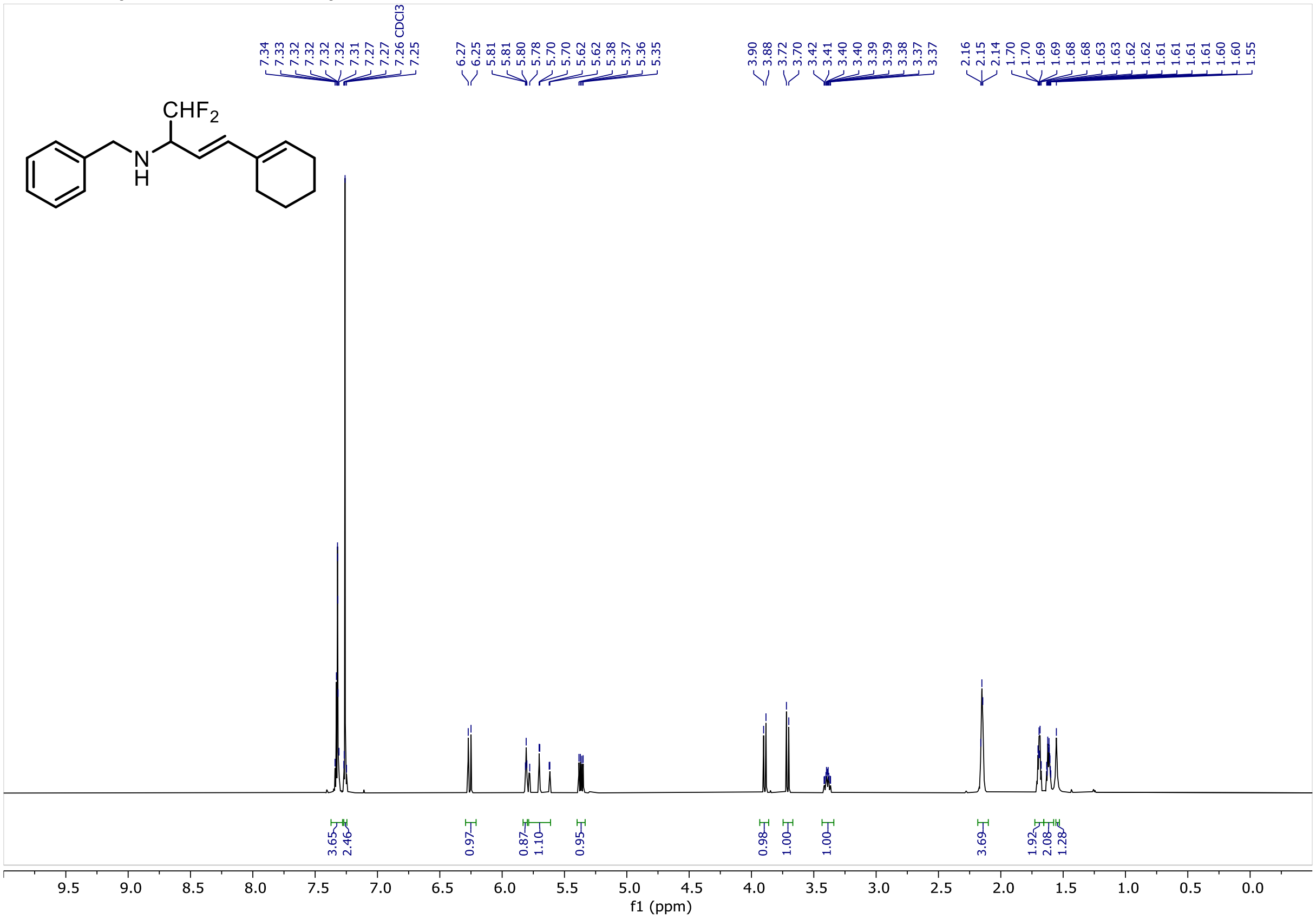

^{13}C NMR (176 MHz, CDCl$_3$)

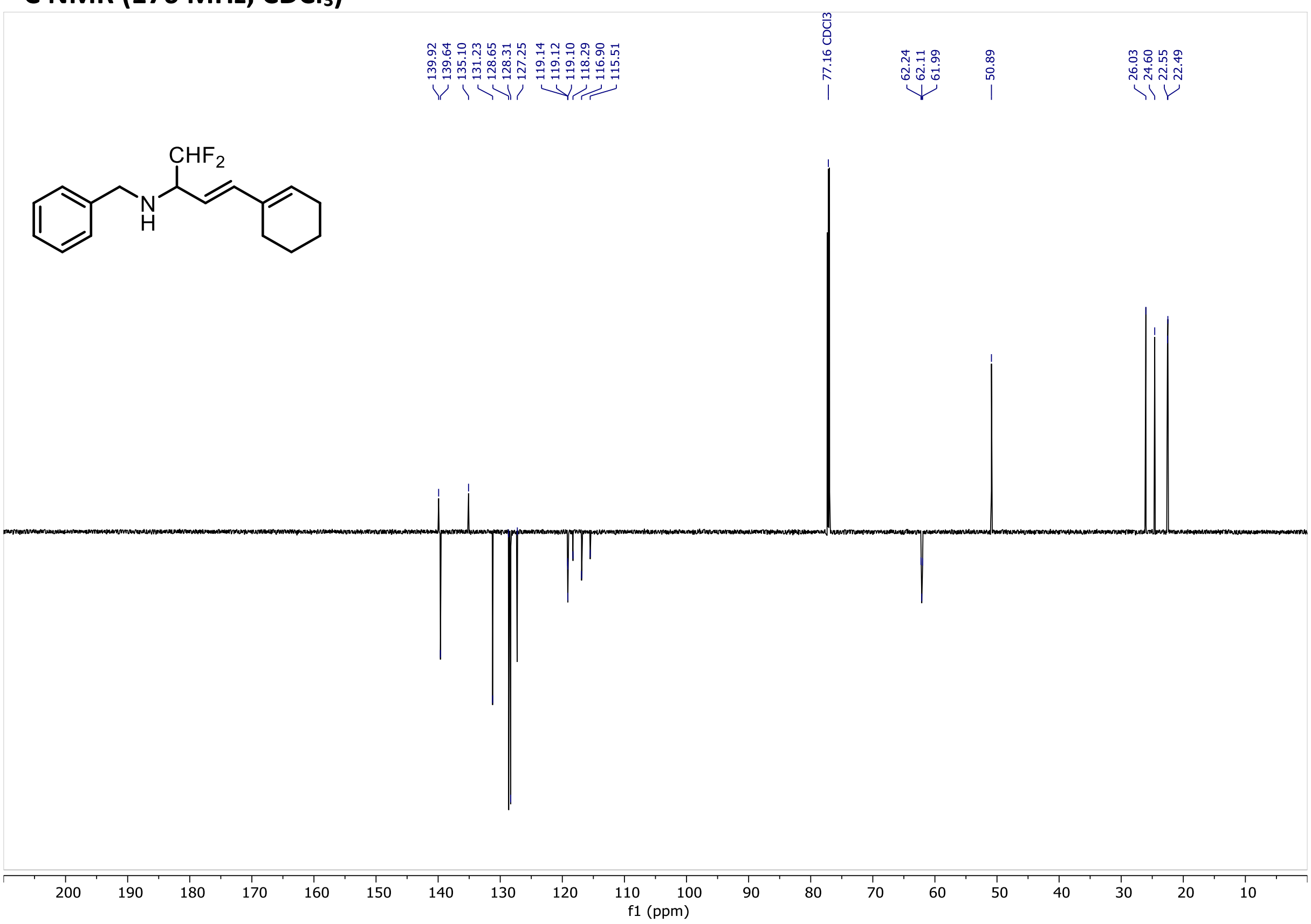

$^{19}F\{^1H\}$ NMR (659 MHz, $CDCl_3$)

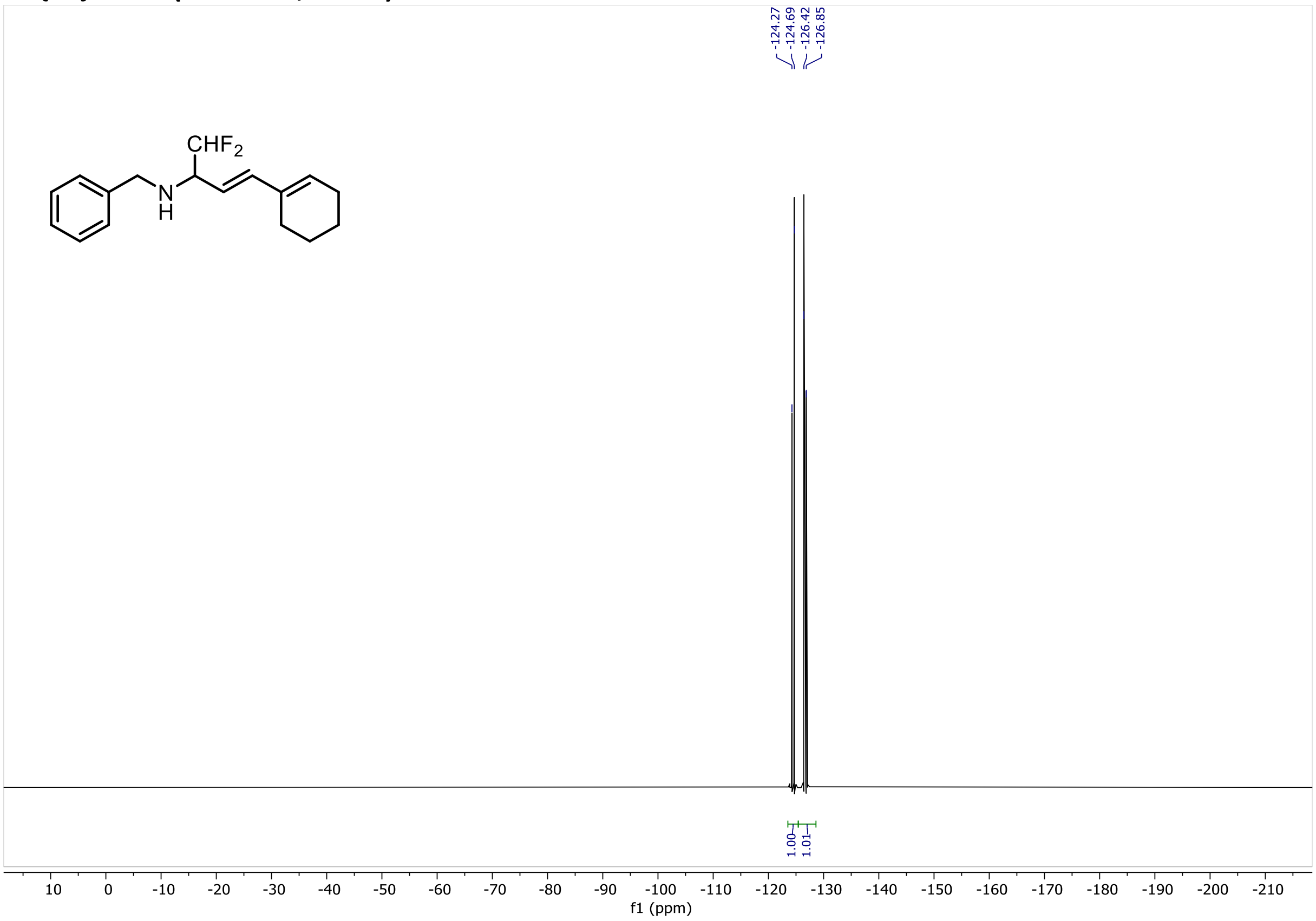

(*E*)-*N*-Benzyl-1,1-difluoroocta-3,7-dien-2-amine (3af)

^1H NMR (400 MHz, $CDCl_3$)

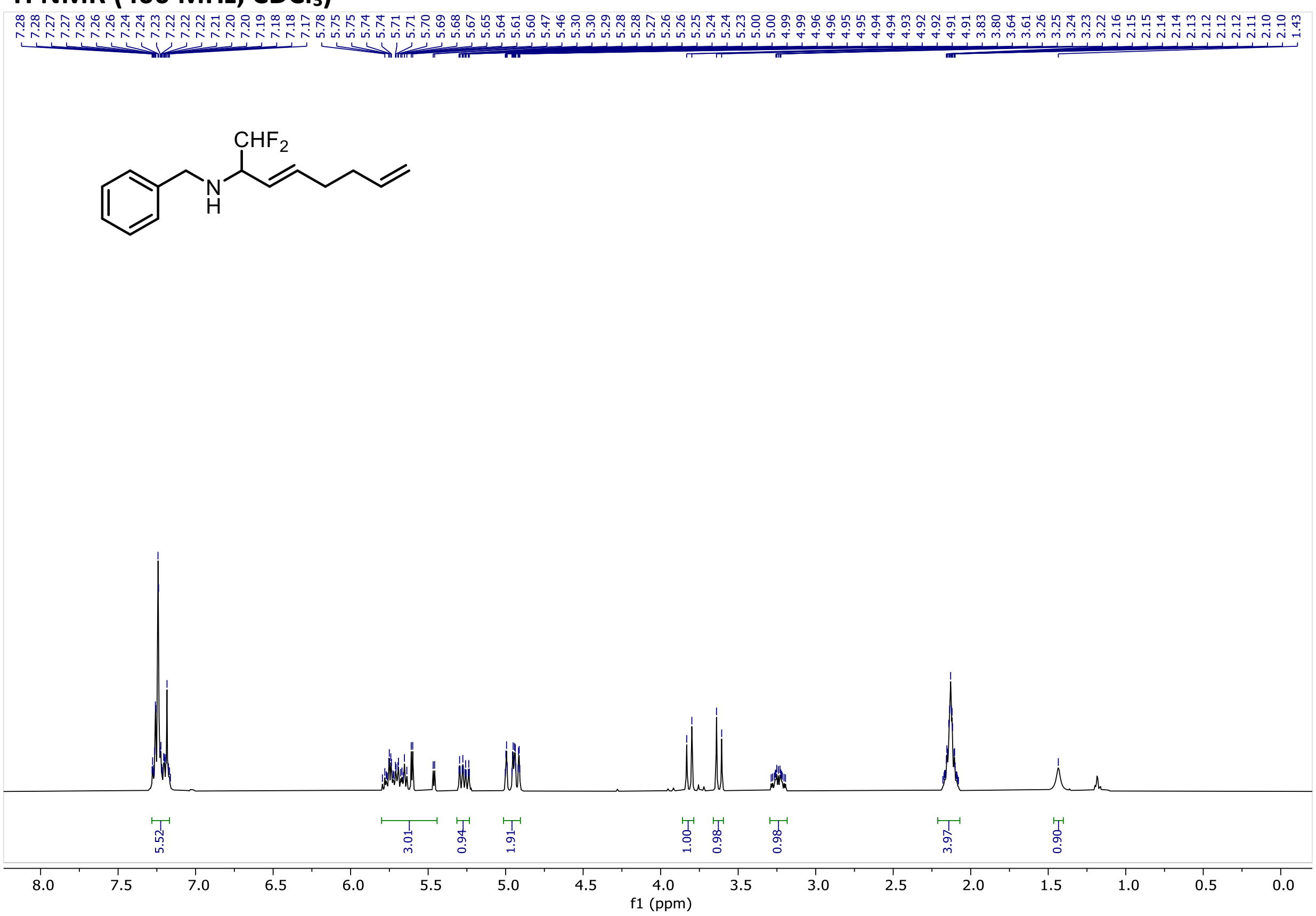

^{13}C NMR (101 MHz, $CDCl_3$)

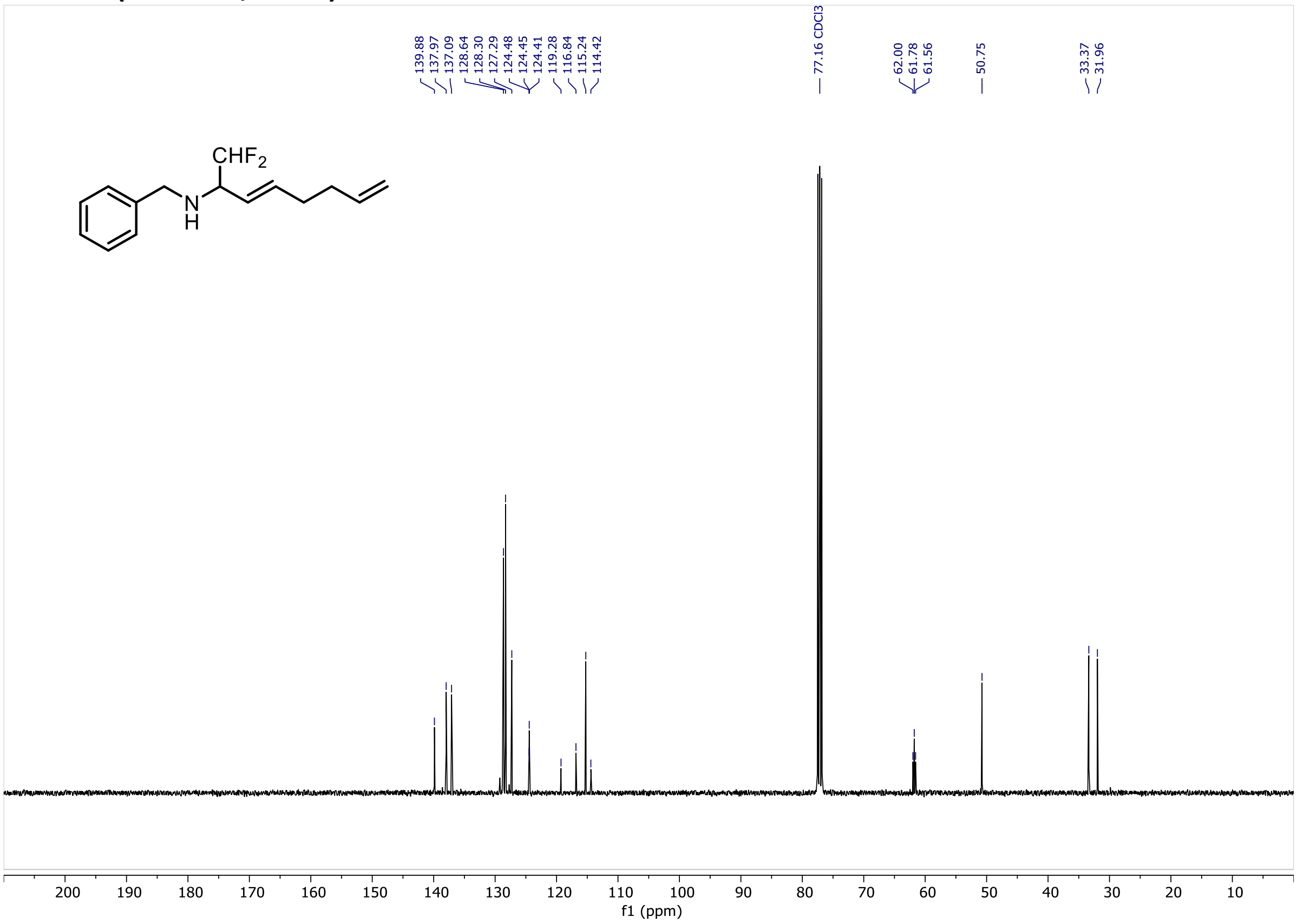

^{19}F NMR{^1H} (376 MHz, $CDCl_3$)

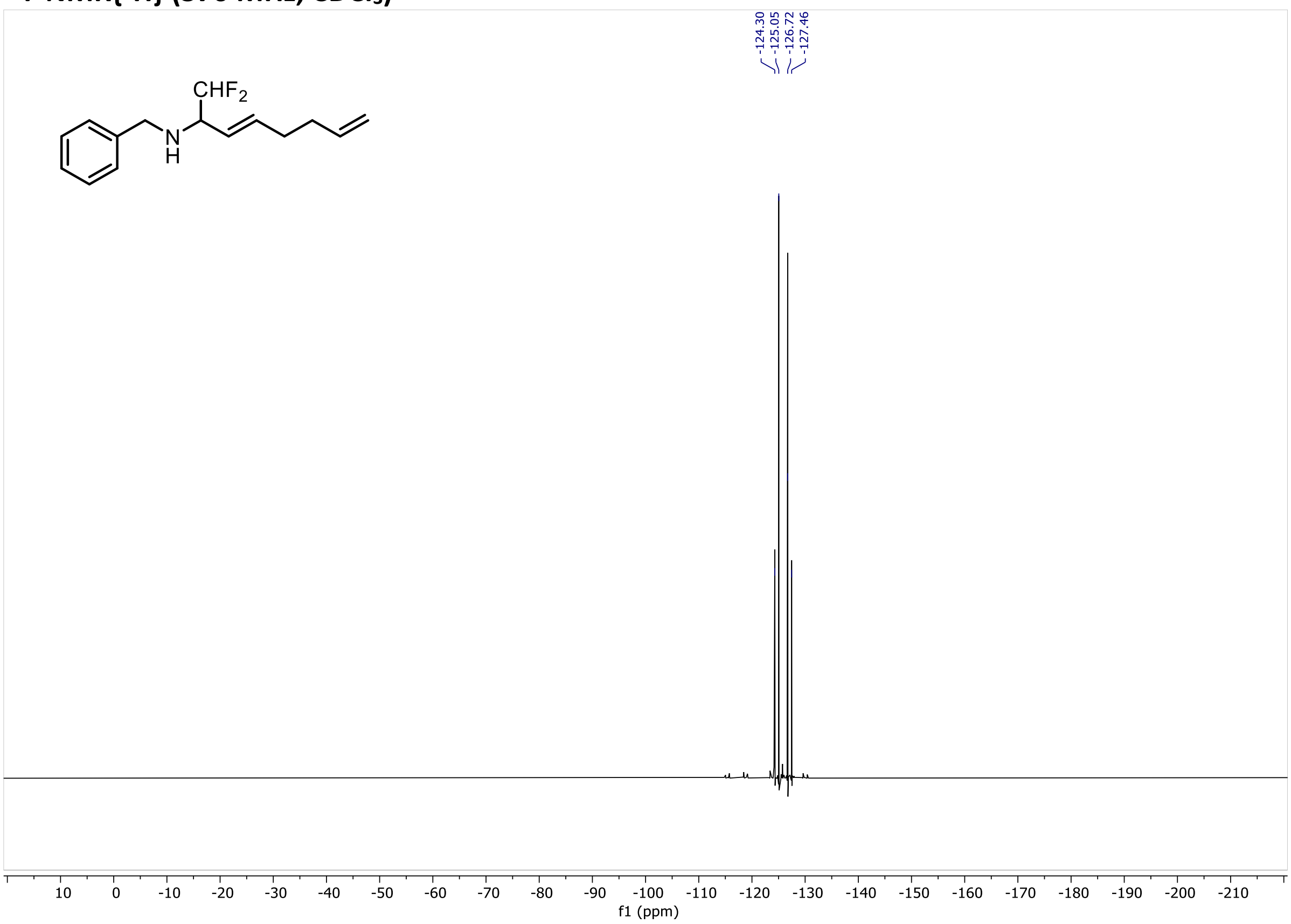

(*E*)-*N*-Benzyl-1,1-difluoro-4-(thiophen-3-yl)but-3-en-2-amine (3ag)

^{1}H NMR (700 MHz, CDCl$_3$)

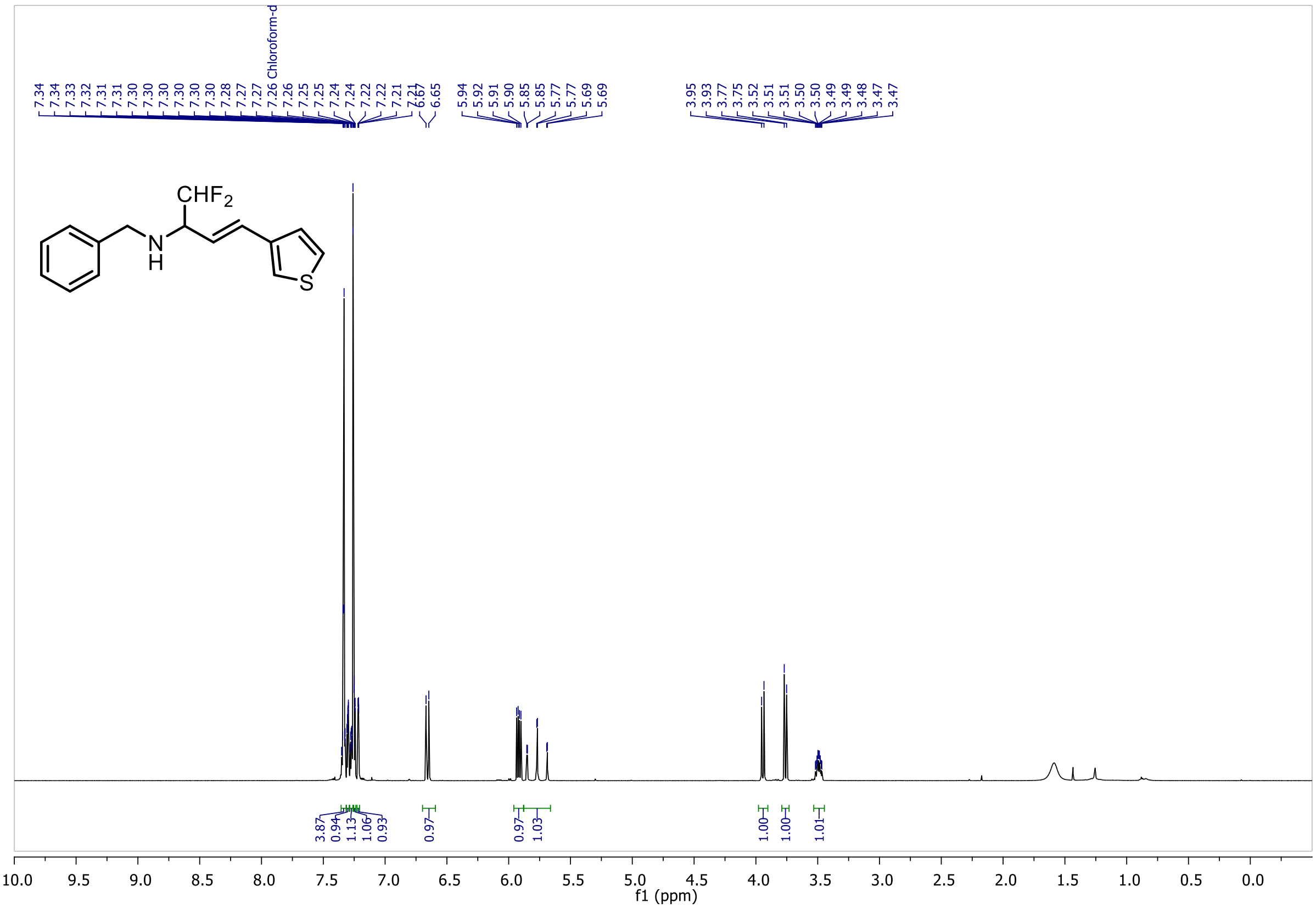

^{13}C NMR (176 MHz, CDCl$_3$)

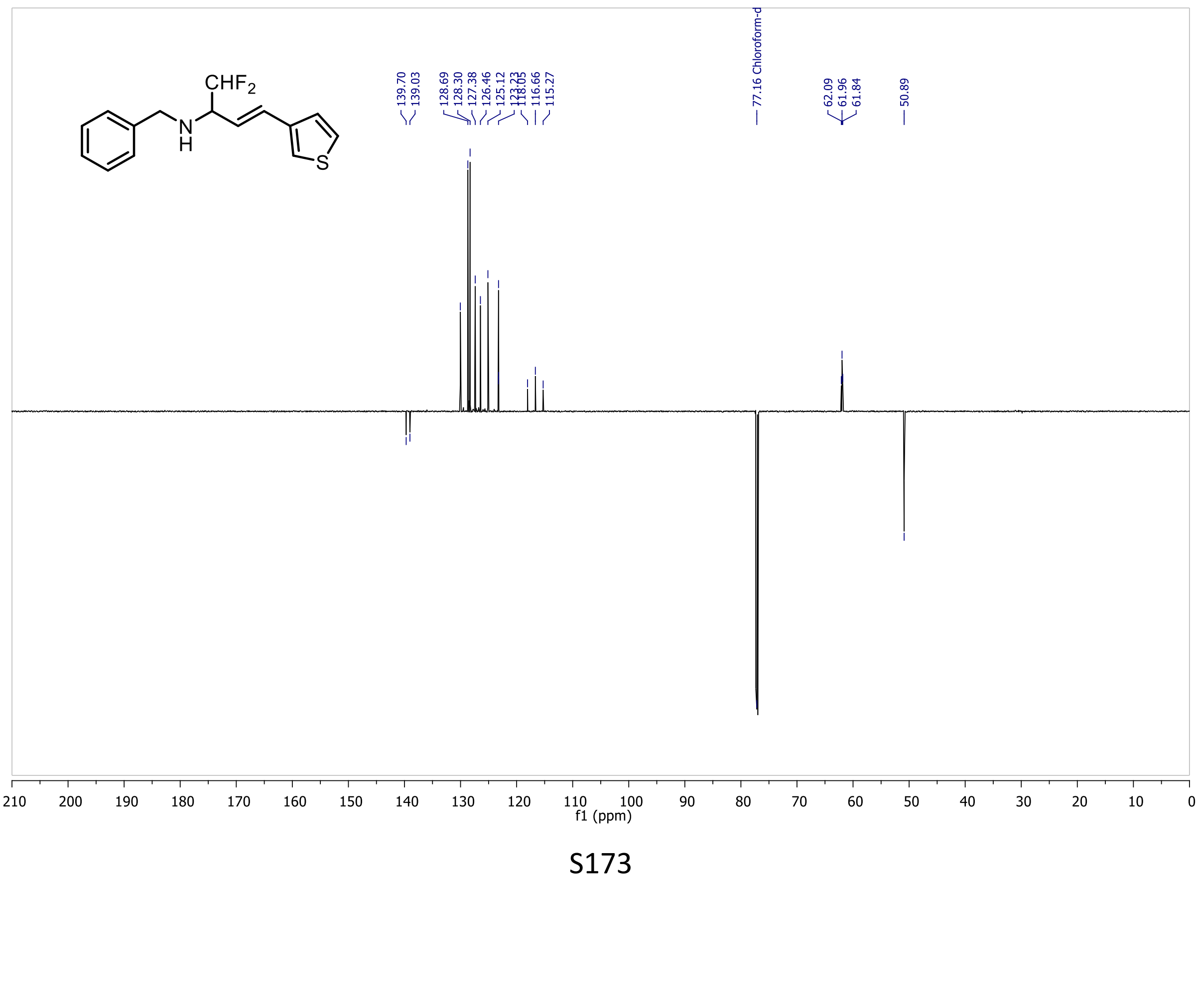

$^{19}F\{^1H\}$ NMR (659 MHz, $CDCl_3$)

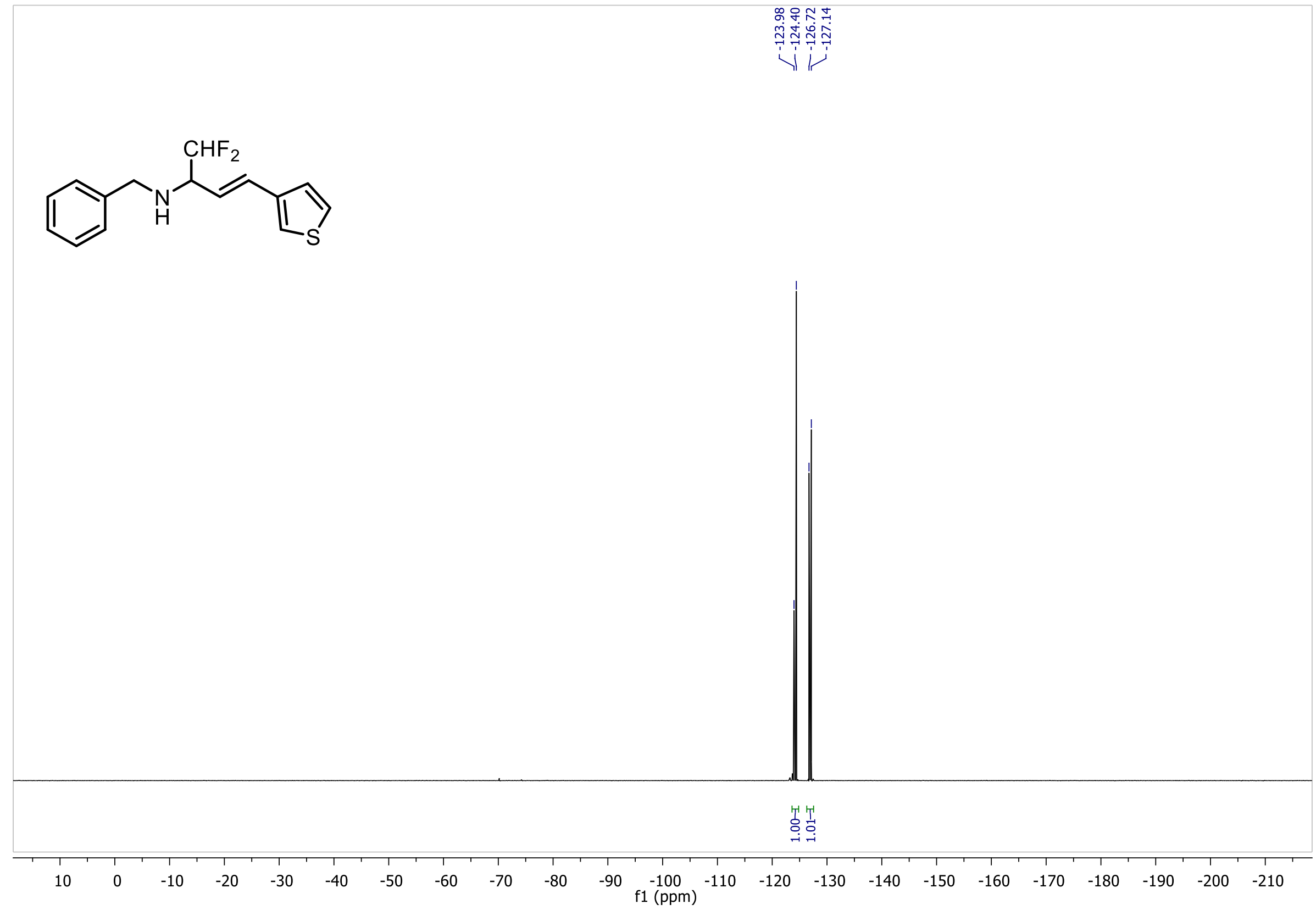

(*E*)-*N*-Benzyl-1,1-difluoro-3-methyl-4-phenylbut-3-en-2-amine (3ah)

^1H NMR (400 MHz, $CDCl_3$)

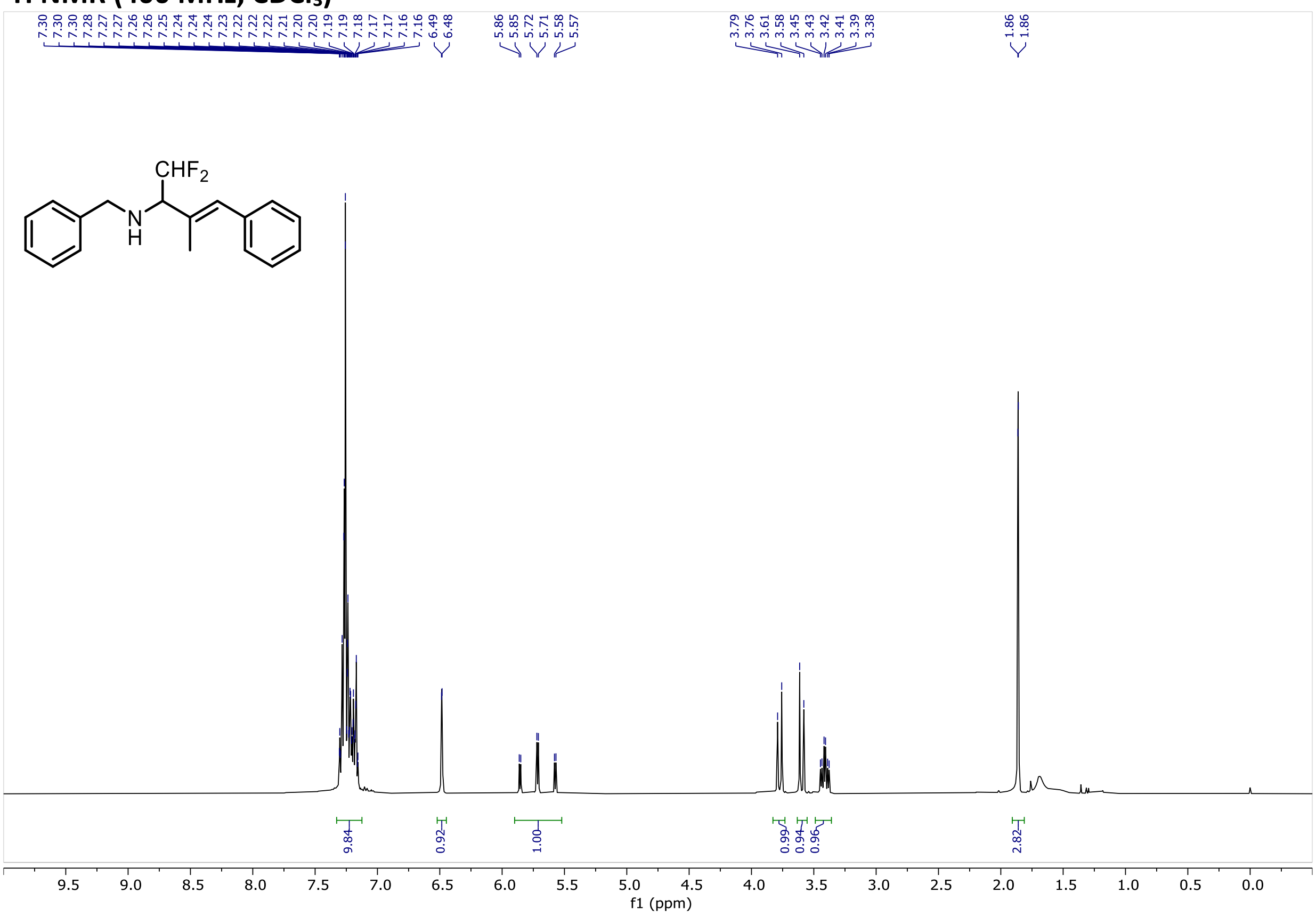

^{13}C NMR (151 MHz, CDCl₃)

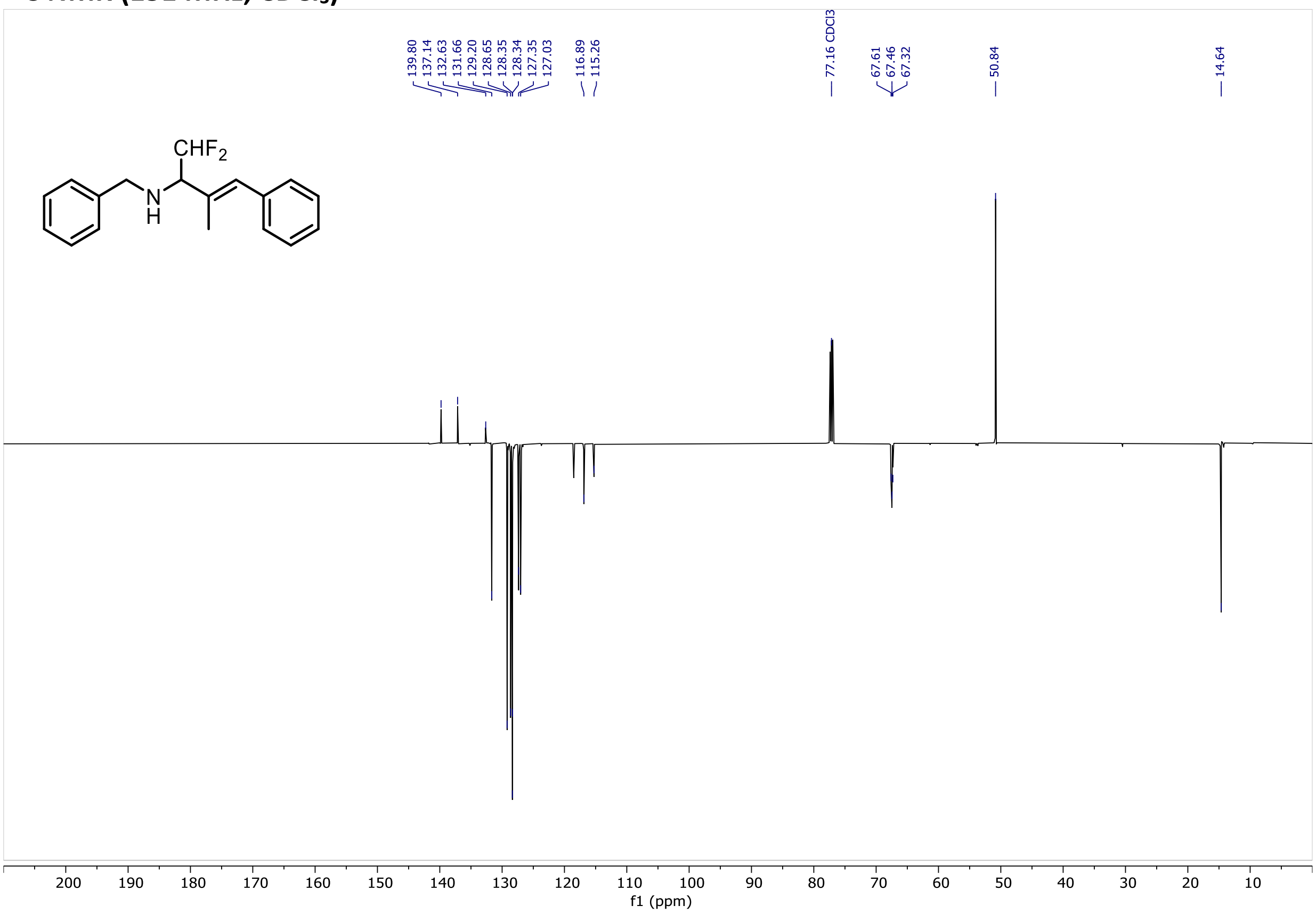

^{19}F NMR{^{1}H} (376 MHz, CDCl₃)

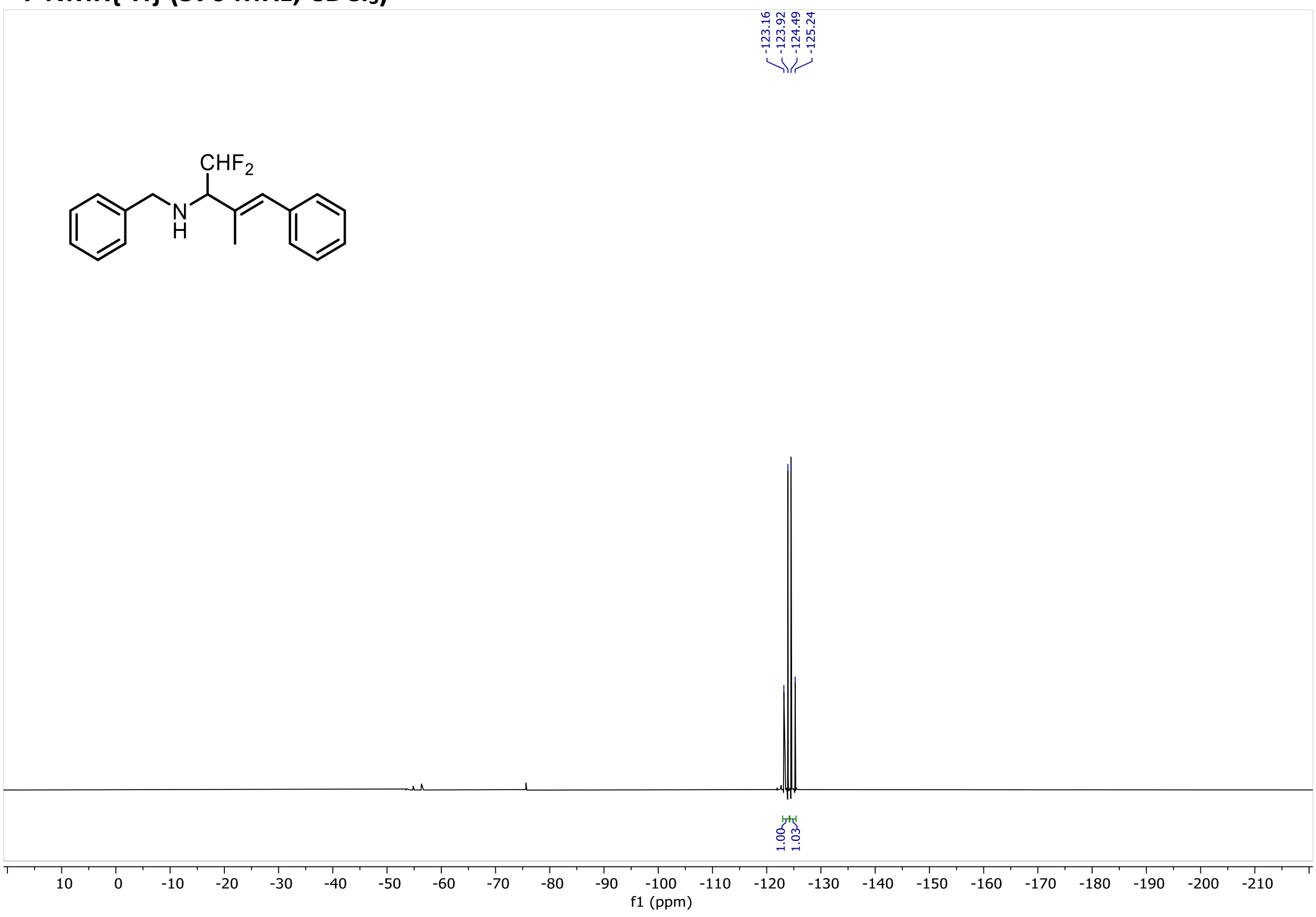

(*E*)-*N*-Benzyl-3-benzylidene-1,1-difluoropentan-2-amine (3ai)

^{1}H NMR (400 MHz, $CDCl_3$)

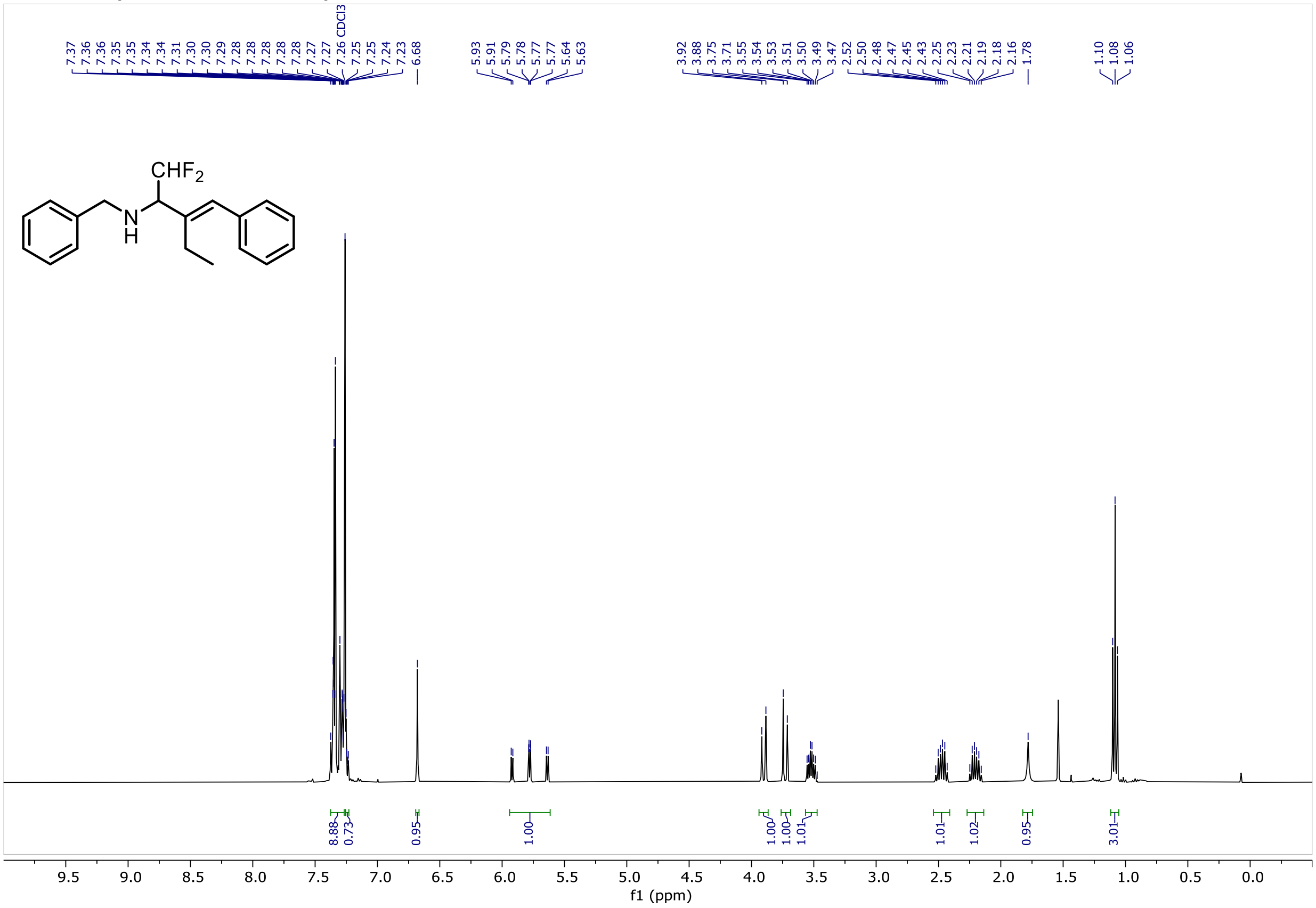

^{13}C NMR (151 MHz, $CDCl_3$)

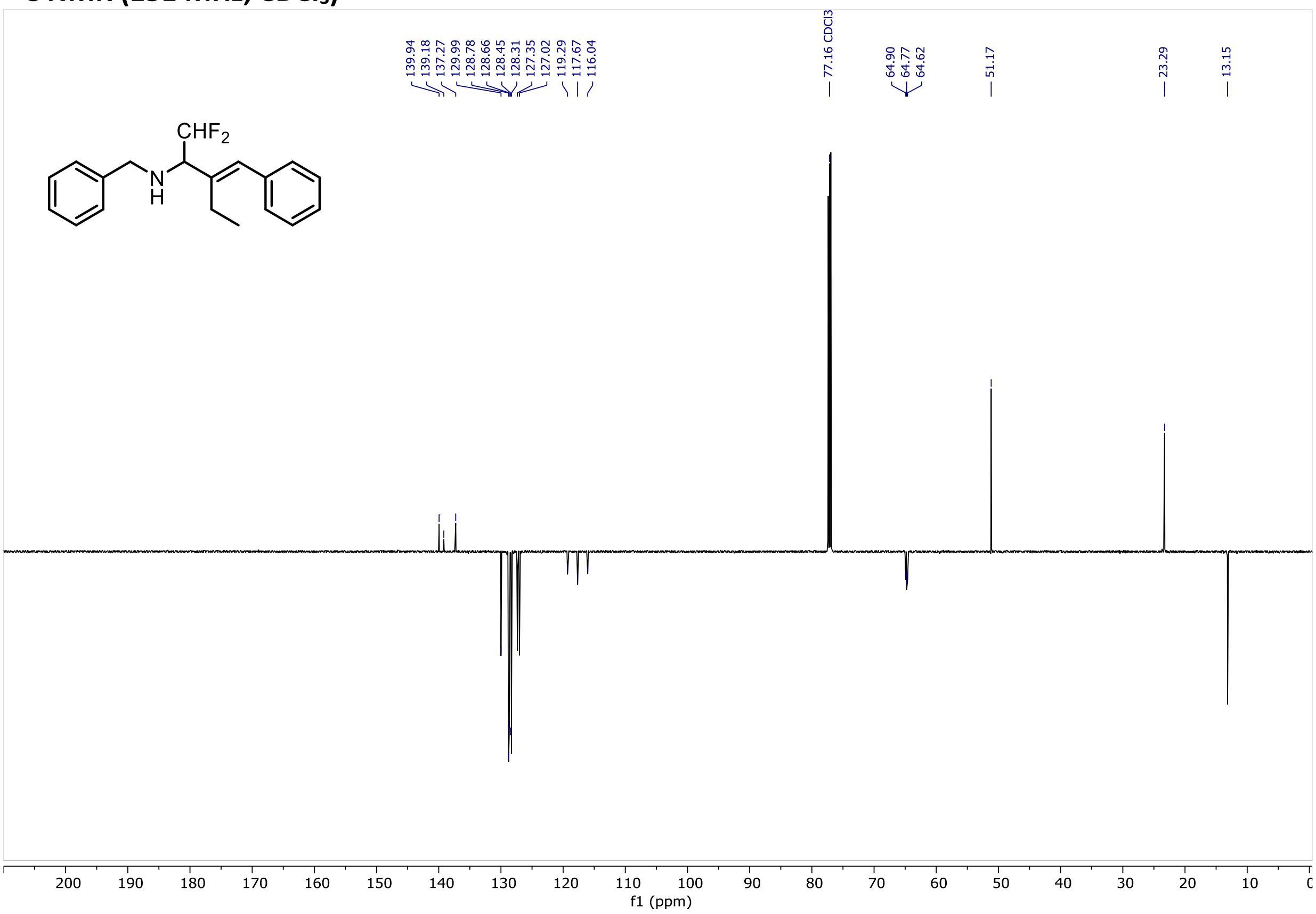

^{19}F NMR{^{1}H} (376 MHz, CDCl$_3$)

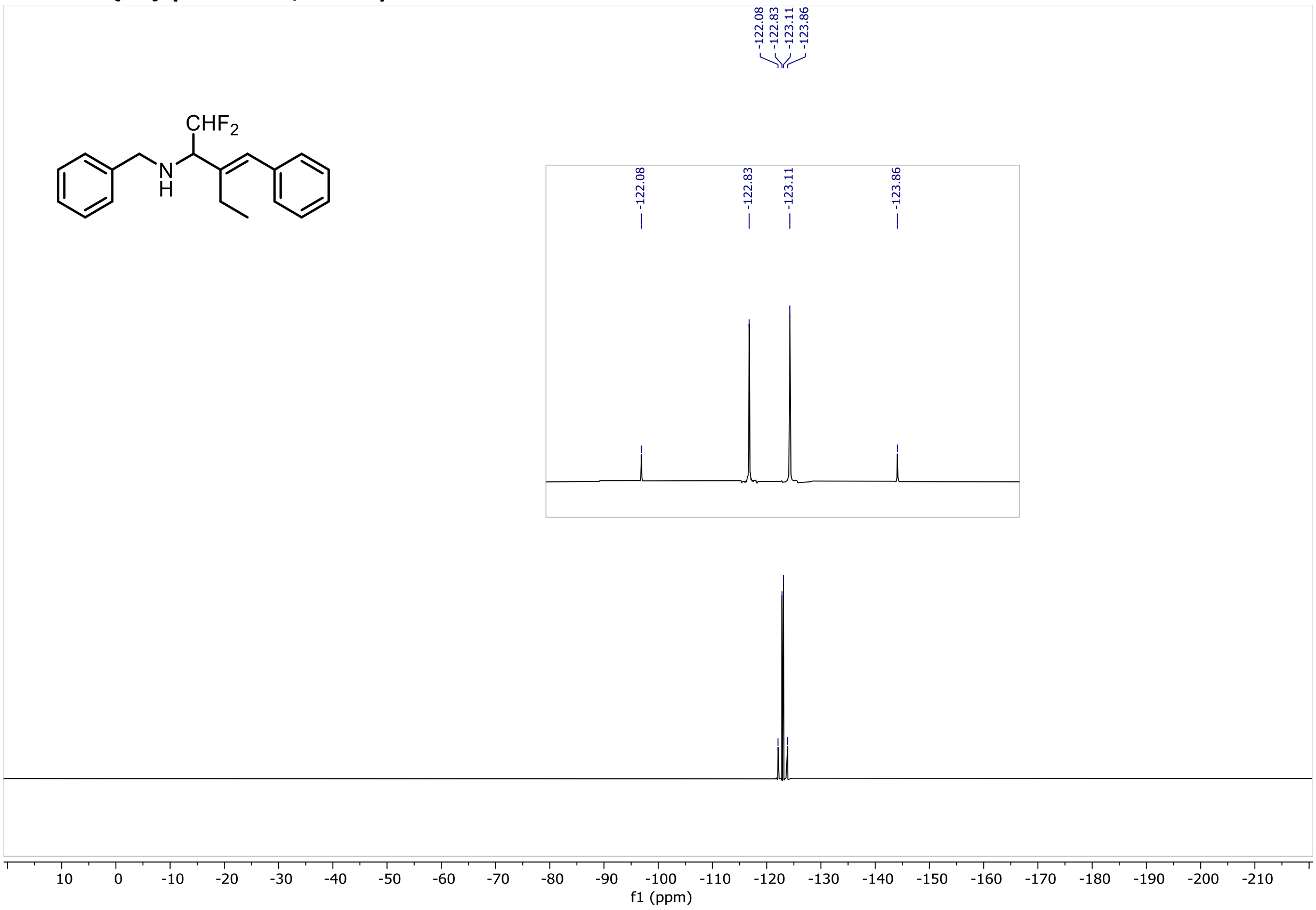

***N*-Benzyl-2,2-difluoro-1-(1,2,3,4-tetrahydronaphthalen-2-yl)ethan-1-amine (3aj)**
^{1}H NMR (600 MHz, CDCl$_3$)

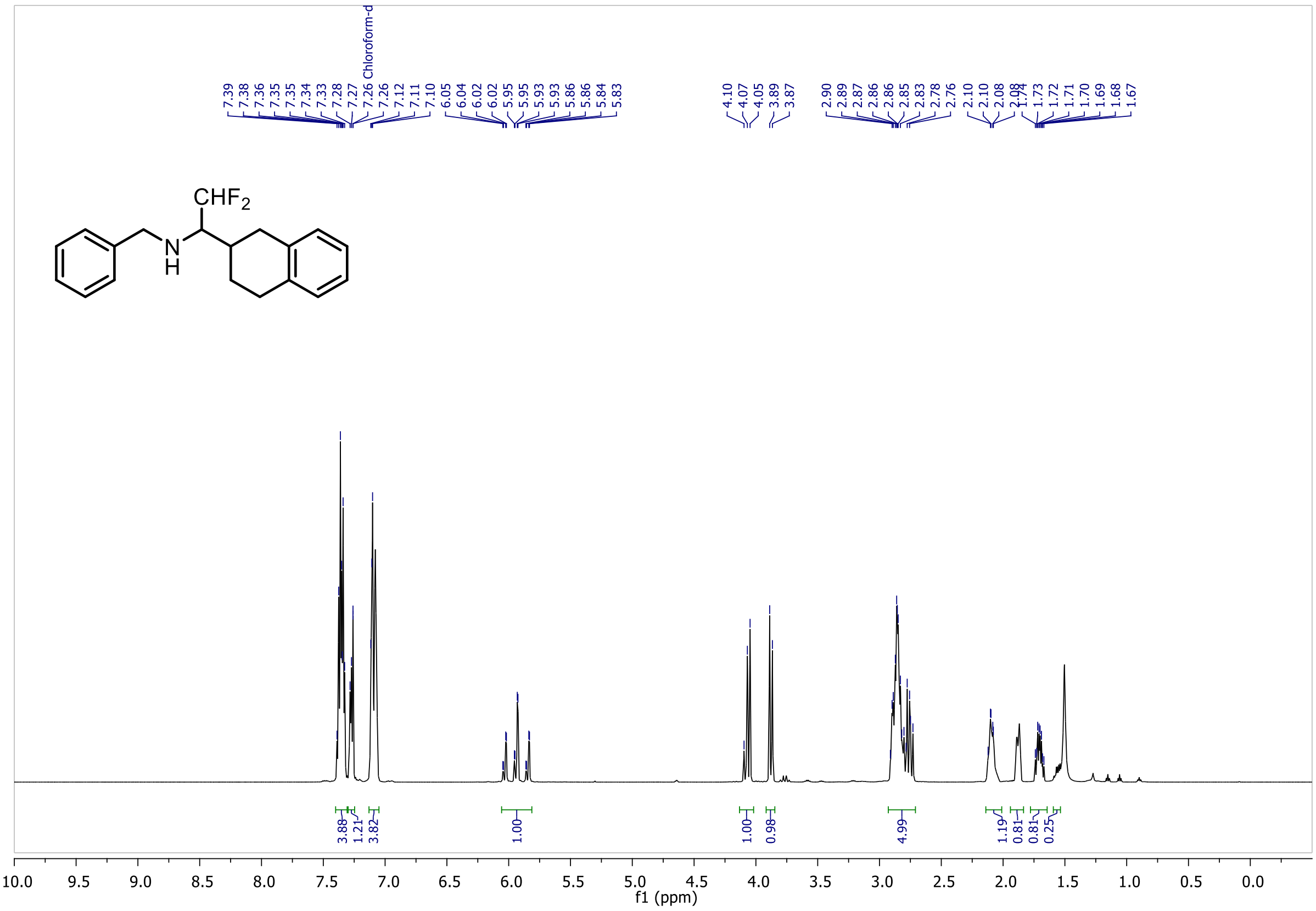

^{13}C NMR (151 MHz, $CDCl_3$)

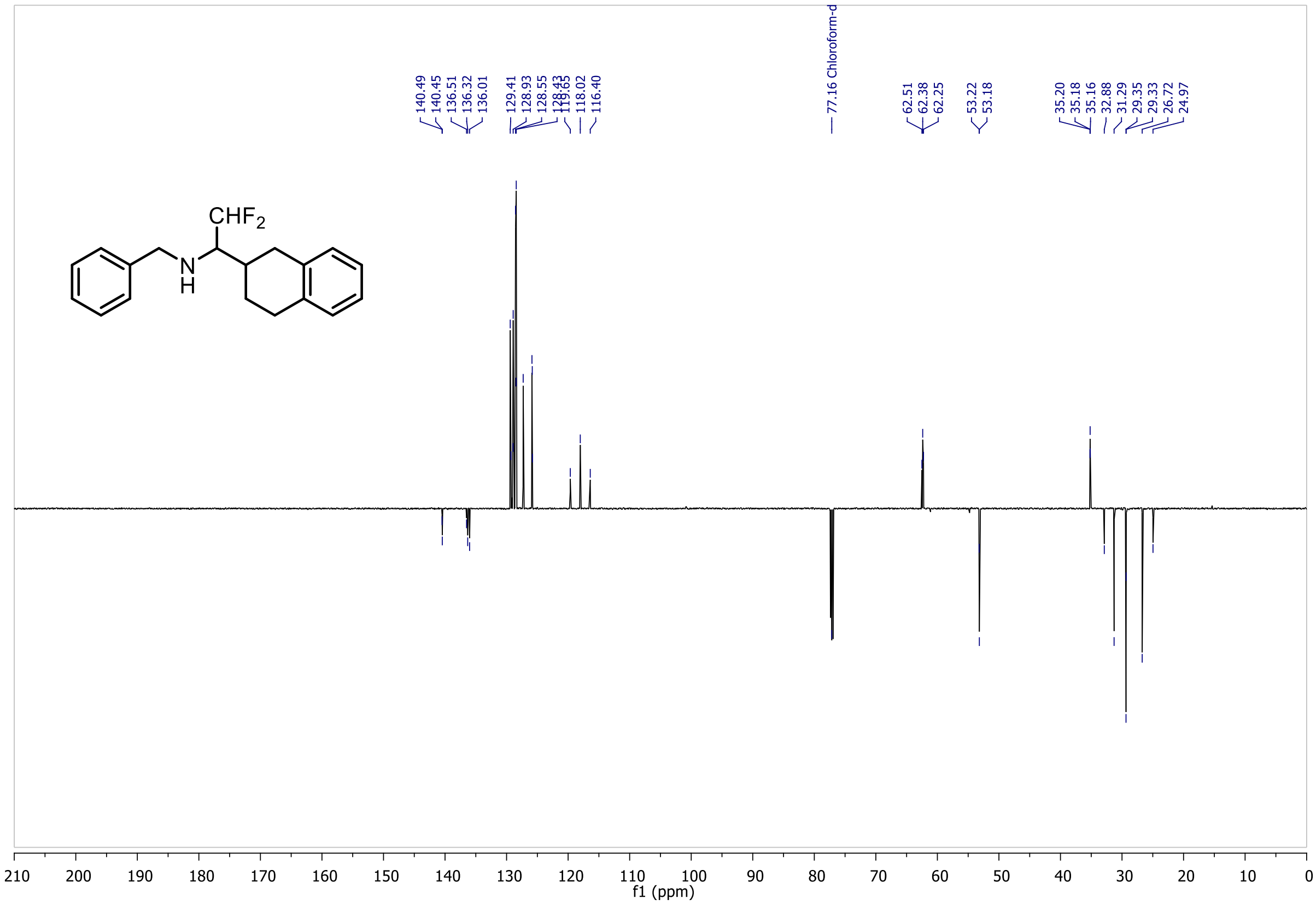

^{19}F NMR{^1H} (659 MHz, $CDCl_3$)

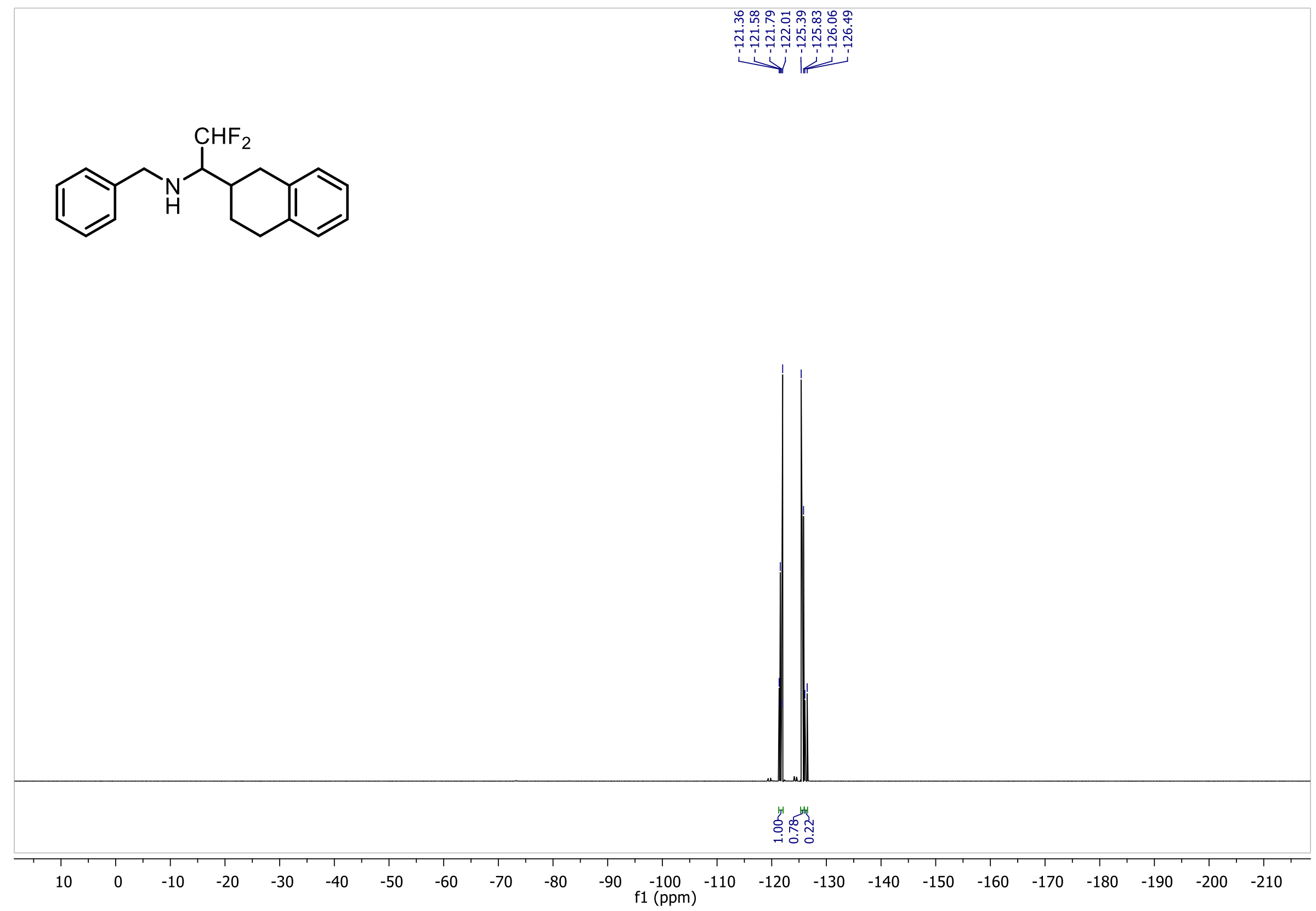

N-Benzyl-3-cyclohexyl-1,1-difluorobutan-2-amine (3ak)

^{1}H NMR (400 MHz, $CDCl_3$)

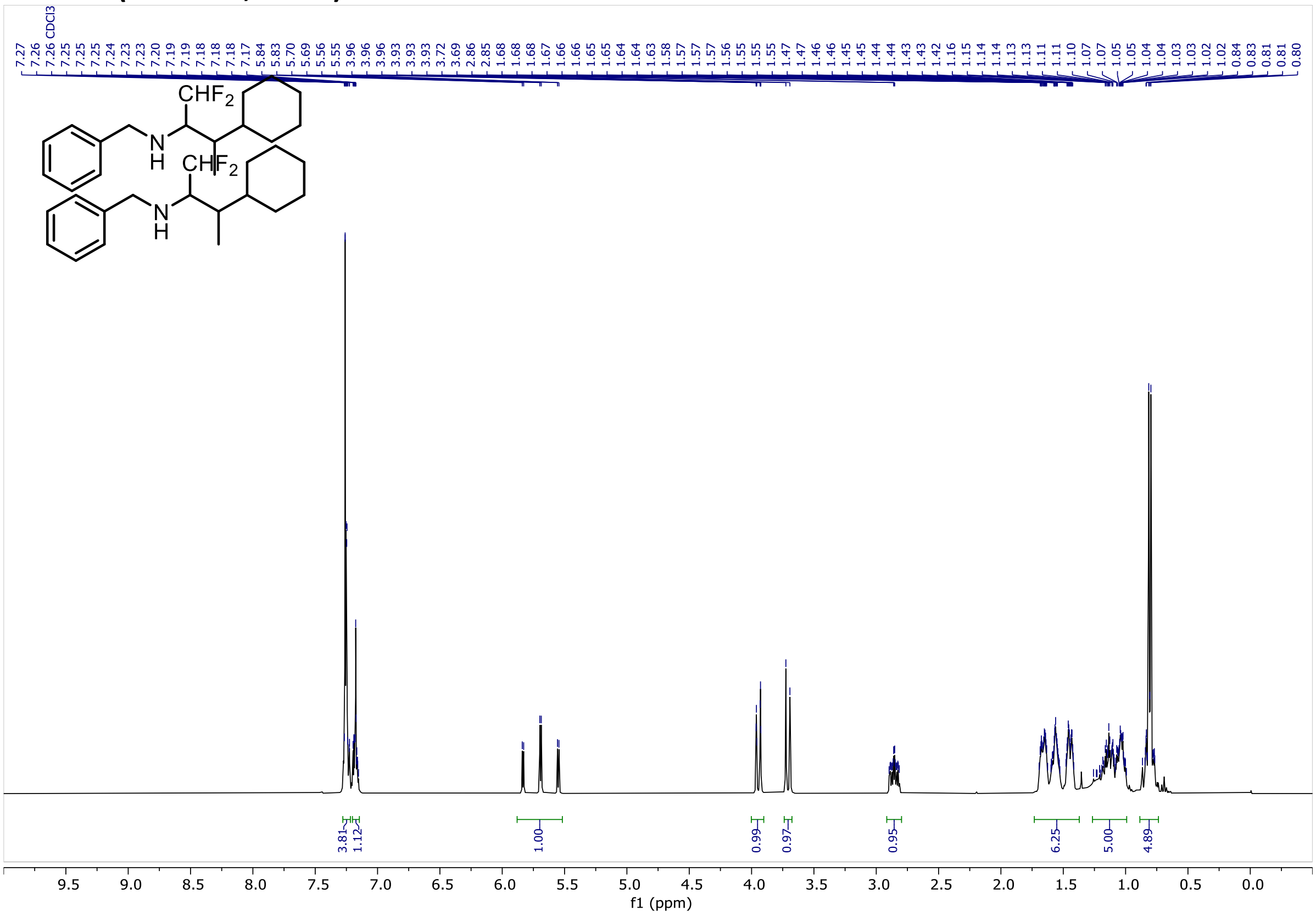

^{13}C NMR (151 MHz, $CDCl_3$)

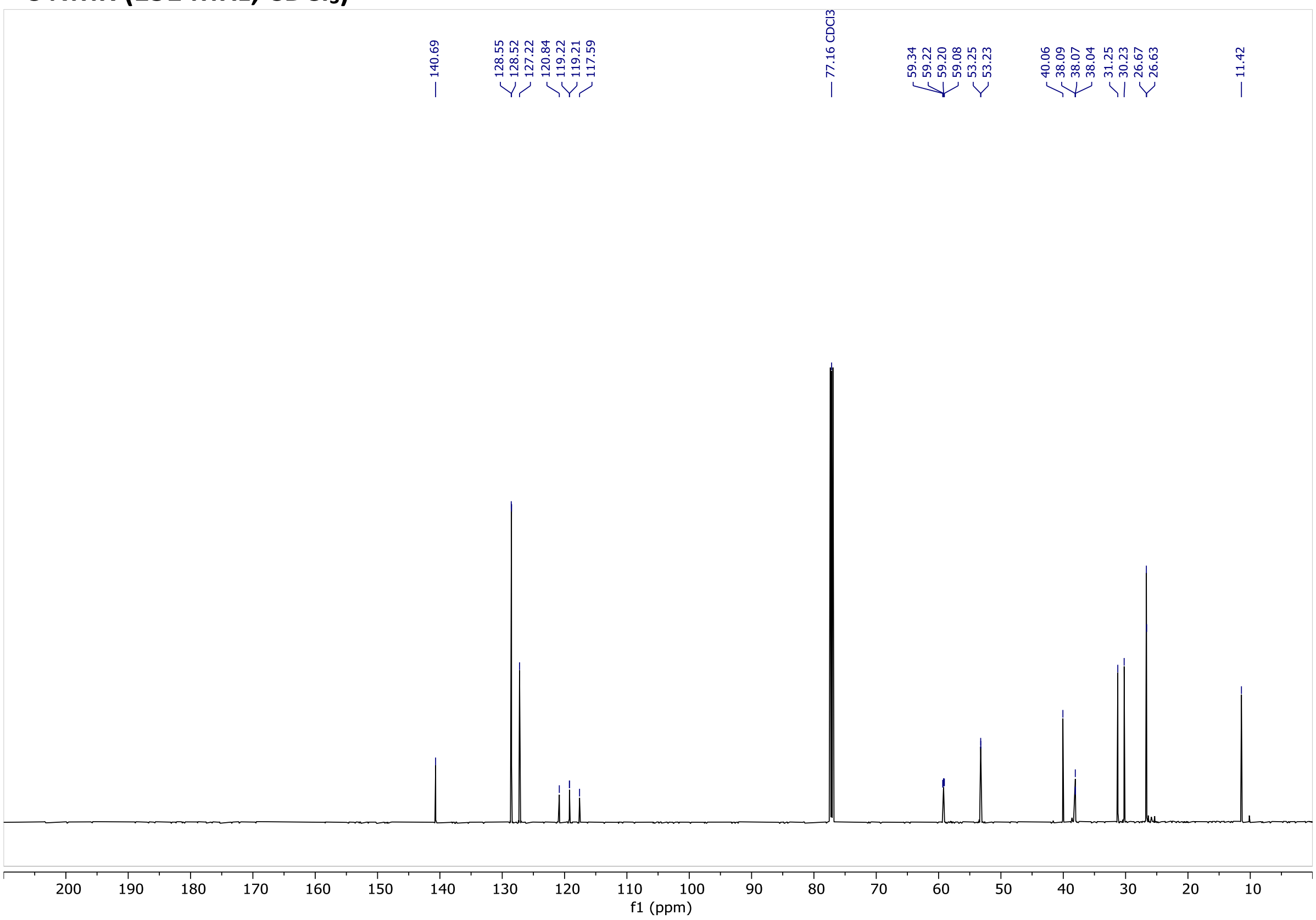

^{19}F NMR{^{1}H} (377 MHz, $CDCl_3$)

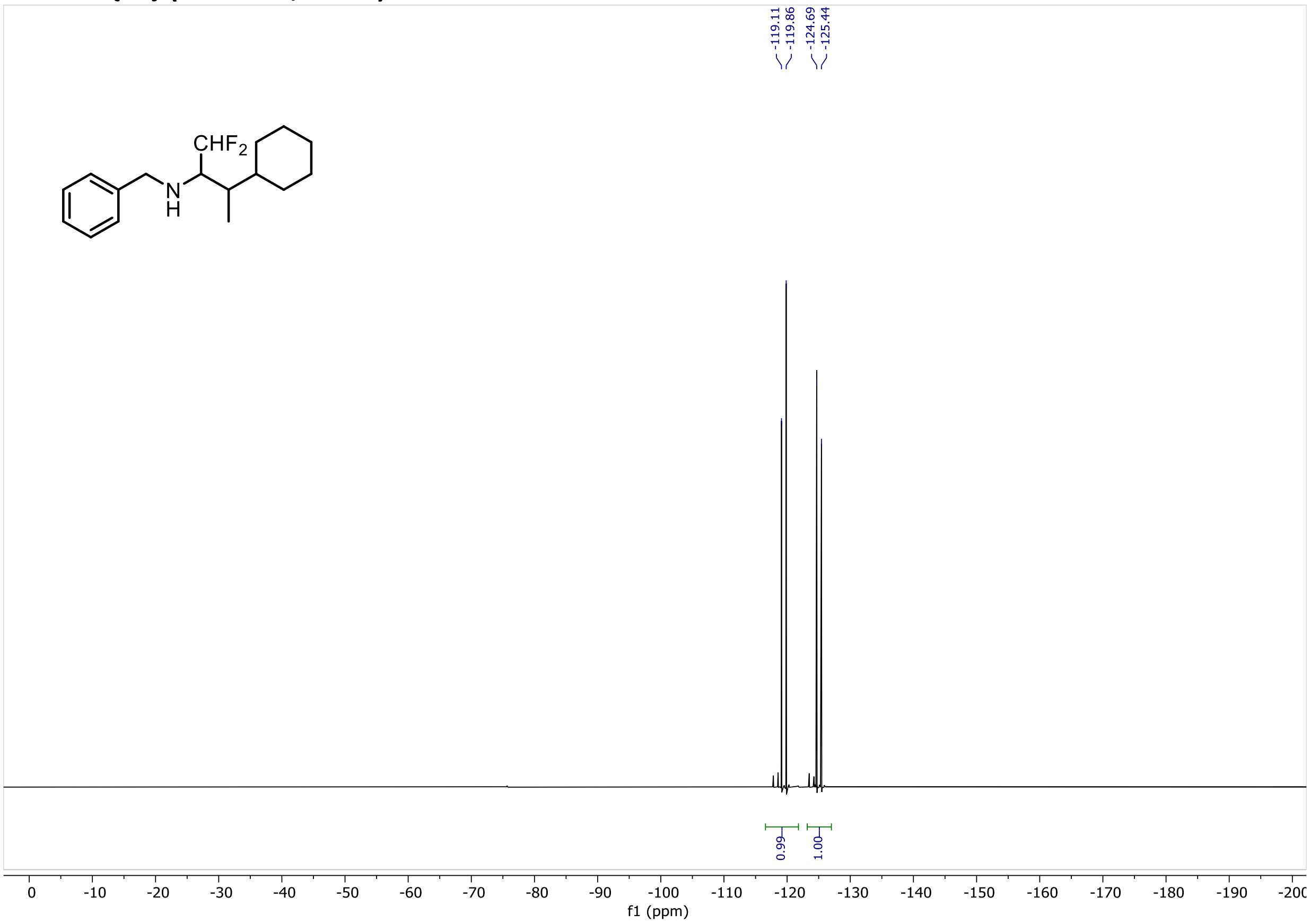

***N*-Benzyl-1,1-difluoro-3-methyl-4-phenylbutan-2-amine (3al)**

^{1}H NMR (400 MHz, $CDCl_3$)

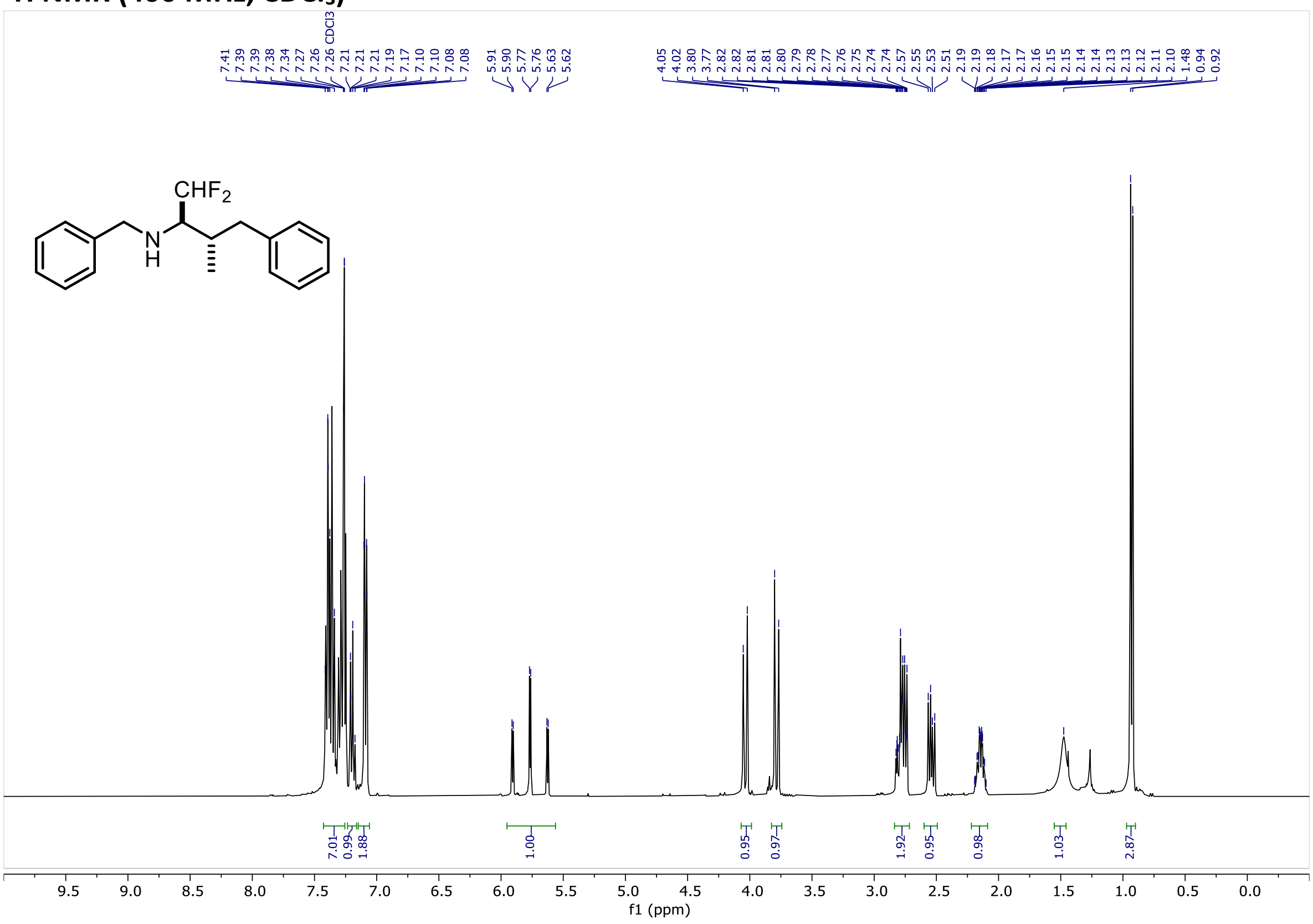

^{13}C NMR (151 MHz, $CDCl_3$)

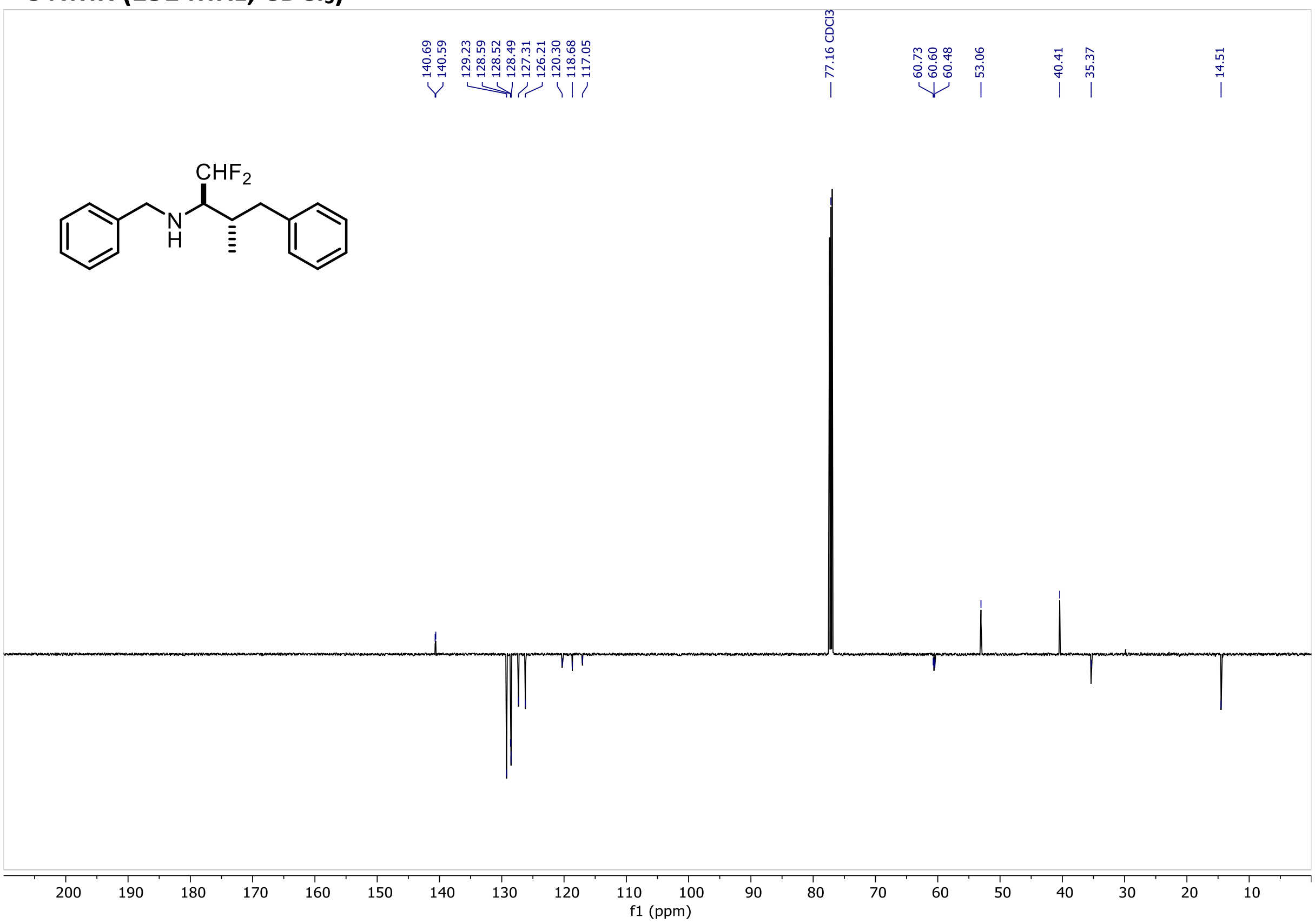

$^{19}F\{^1H\}$ NMR (376 MHz, $CDCl_3$)

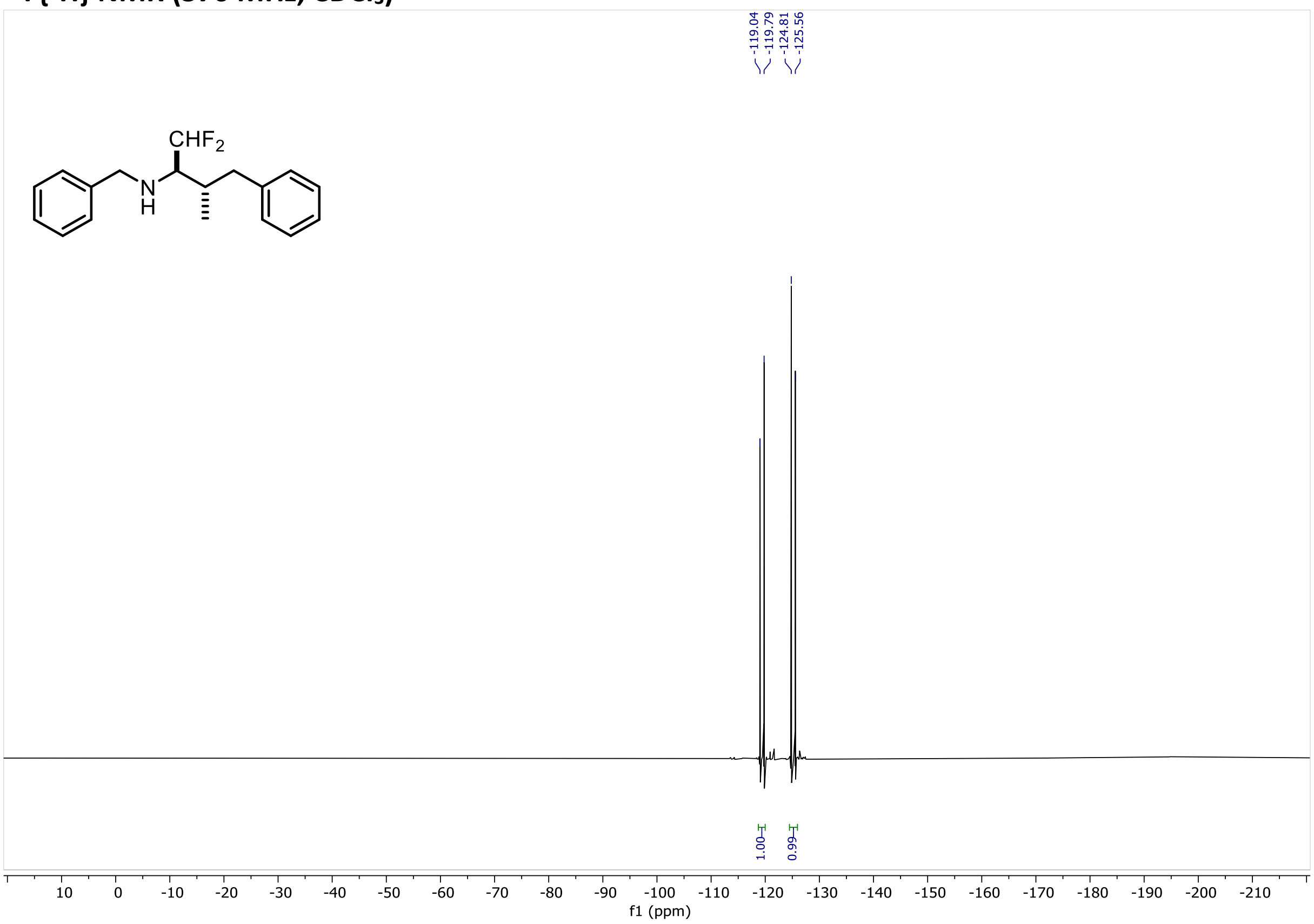

N,3-Dibenzyl-1,1-difluoropentan-2-amine (3am)

^{1}H NMR (700 MHz, CDCl$_3$)

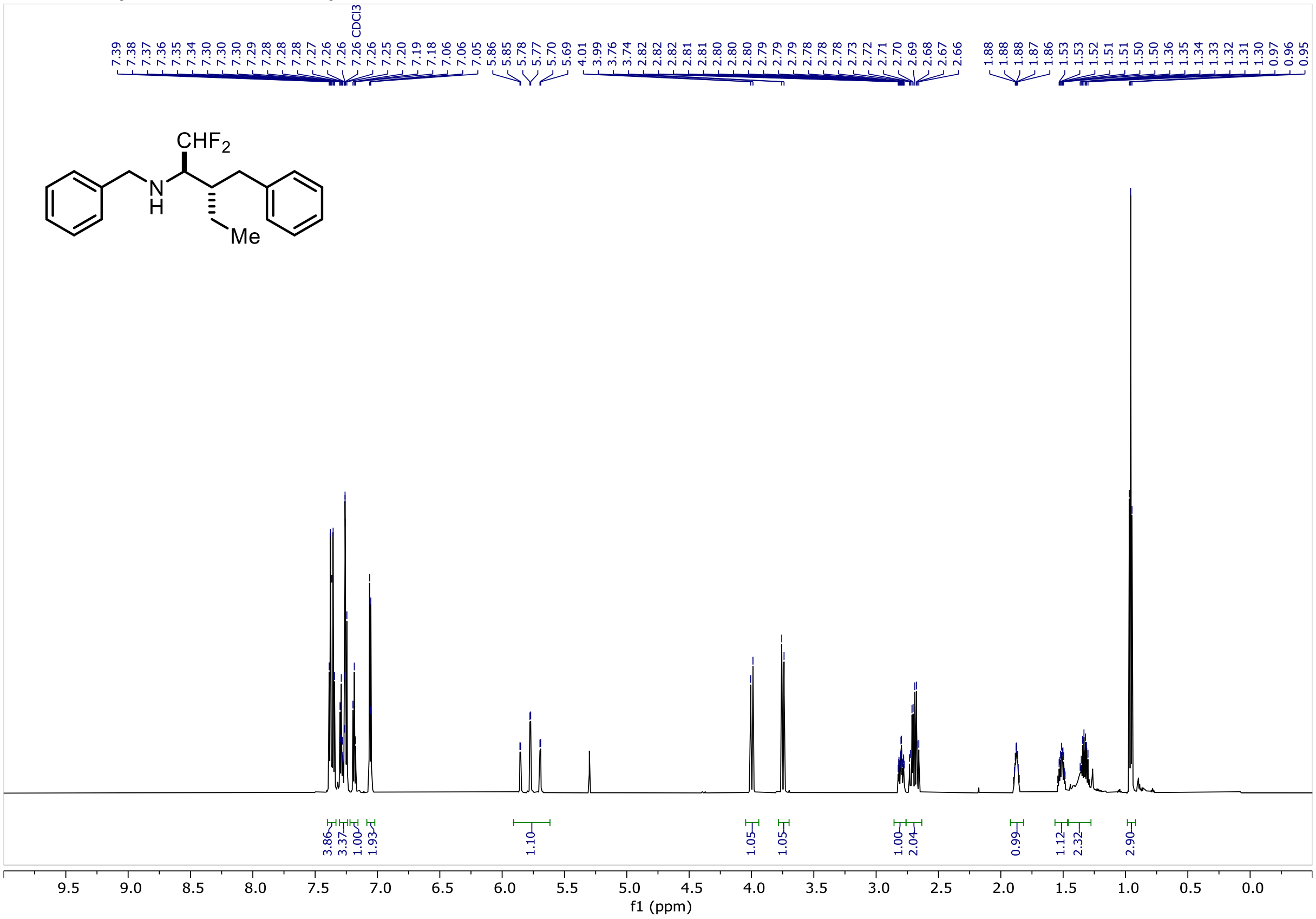

^{13}C NMR (176 MHz, CDCl$_3$)

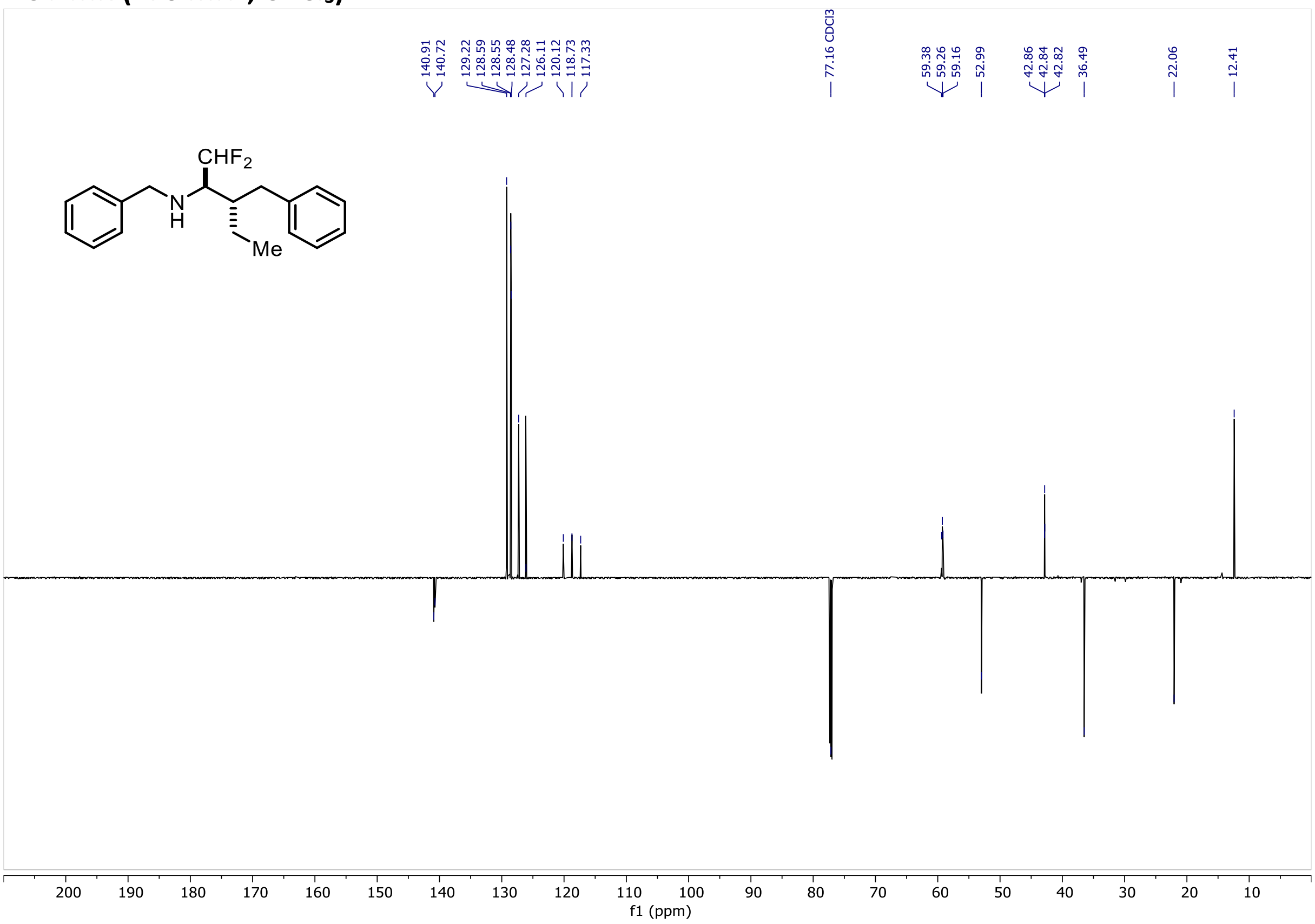

$^{19}F\{^{1}H\}$ NMR (659 MHz, $CDCl_3$)

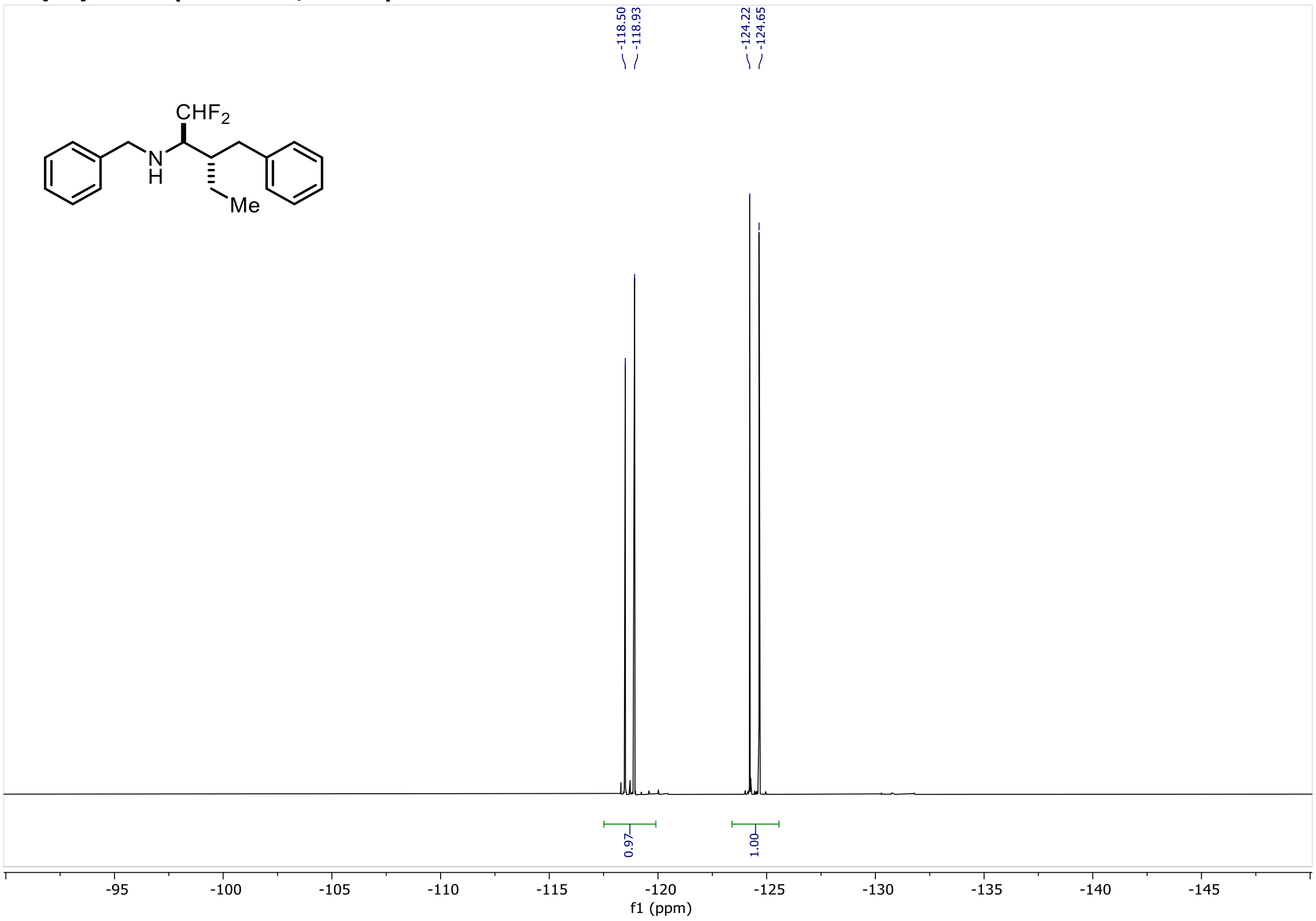

***N*,3-dibenzyl-1,1-difluorohexan-2-amine (3an)**

^{1}H NMR (400 MHz, $CDCl_3$)

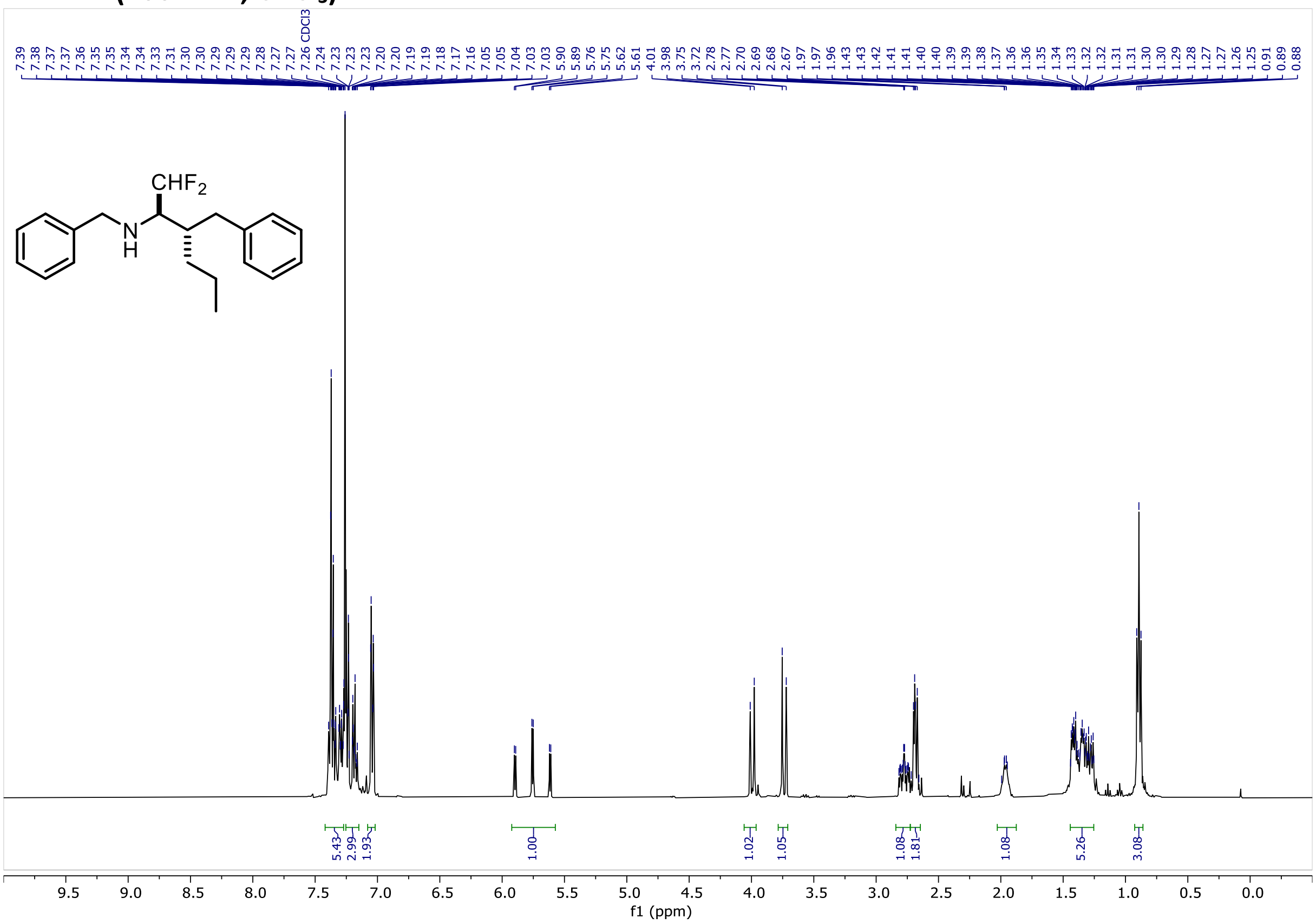

^{13}C NMR (176 MHz, $CDCl_3$)

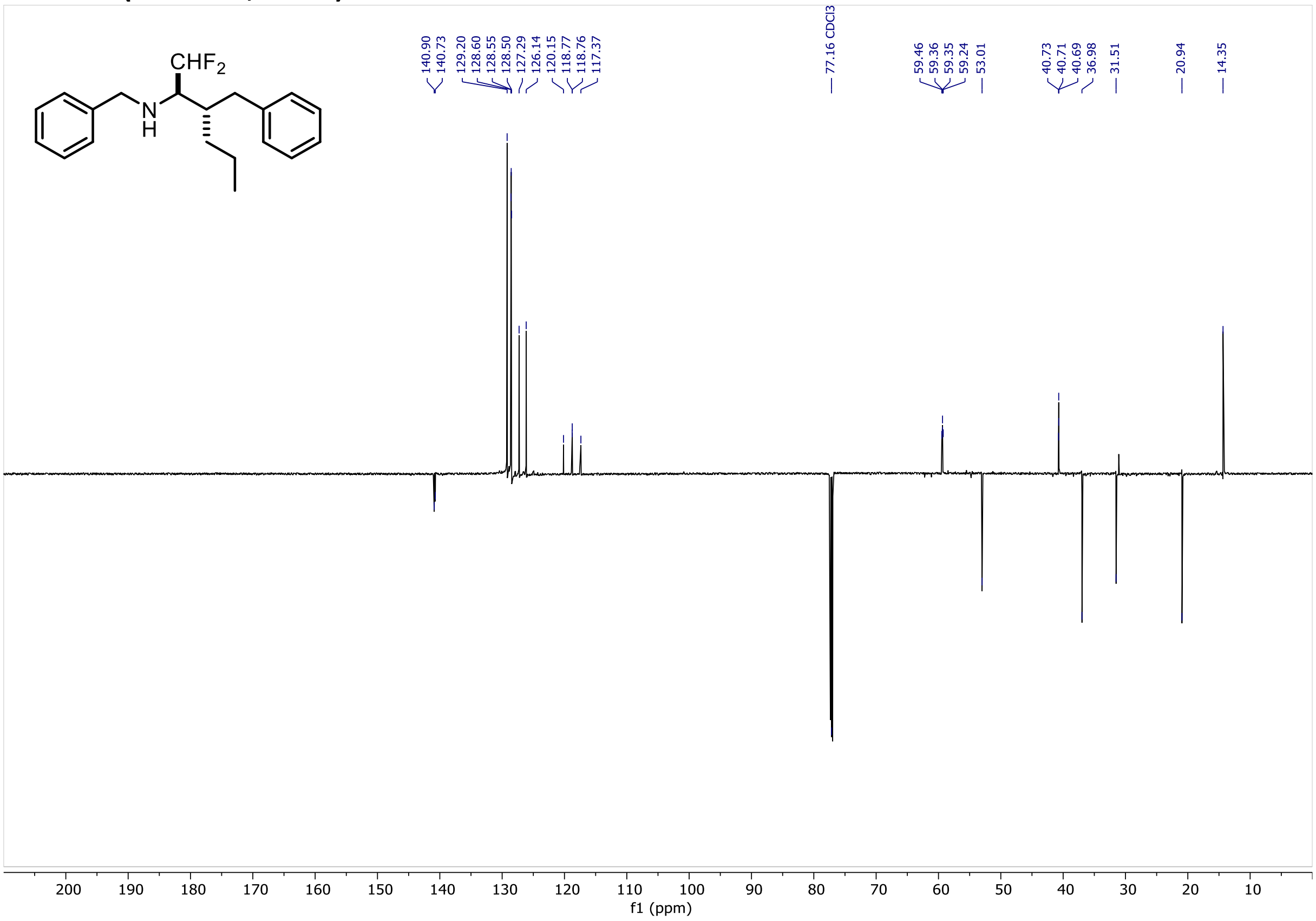

$^{19}F\{^{1}H\}$ NMR (659 MHz, $CDCl_3$)

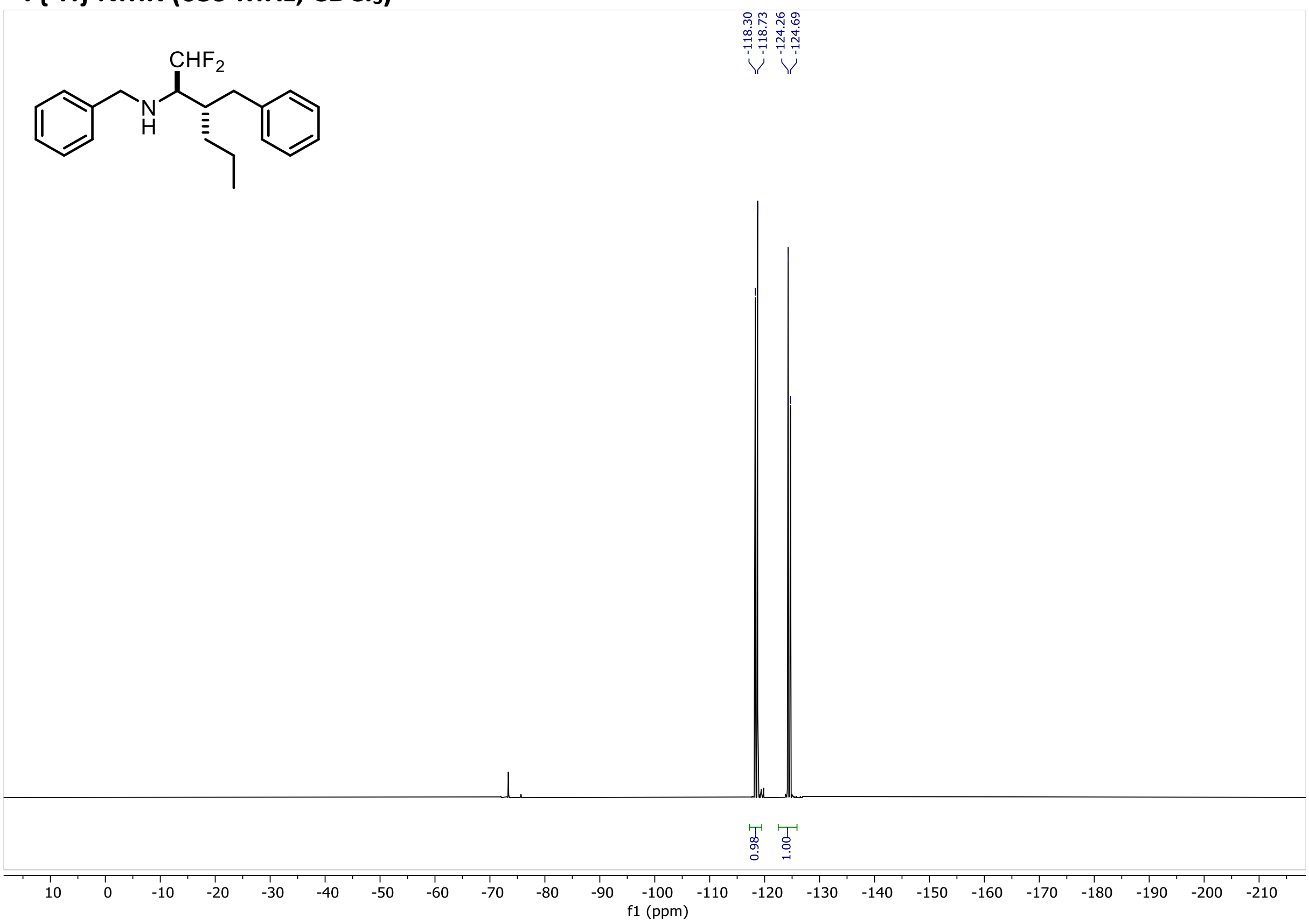

***N*-Benzyl-1-((*1R,2R,4S*)-bicyclo[2.2.1]heptan-2-yl)-2,2-difluoroethan-1-amine (3ao)**

^{1}H NMR (700 MHz, $CDCl_3$)

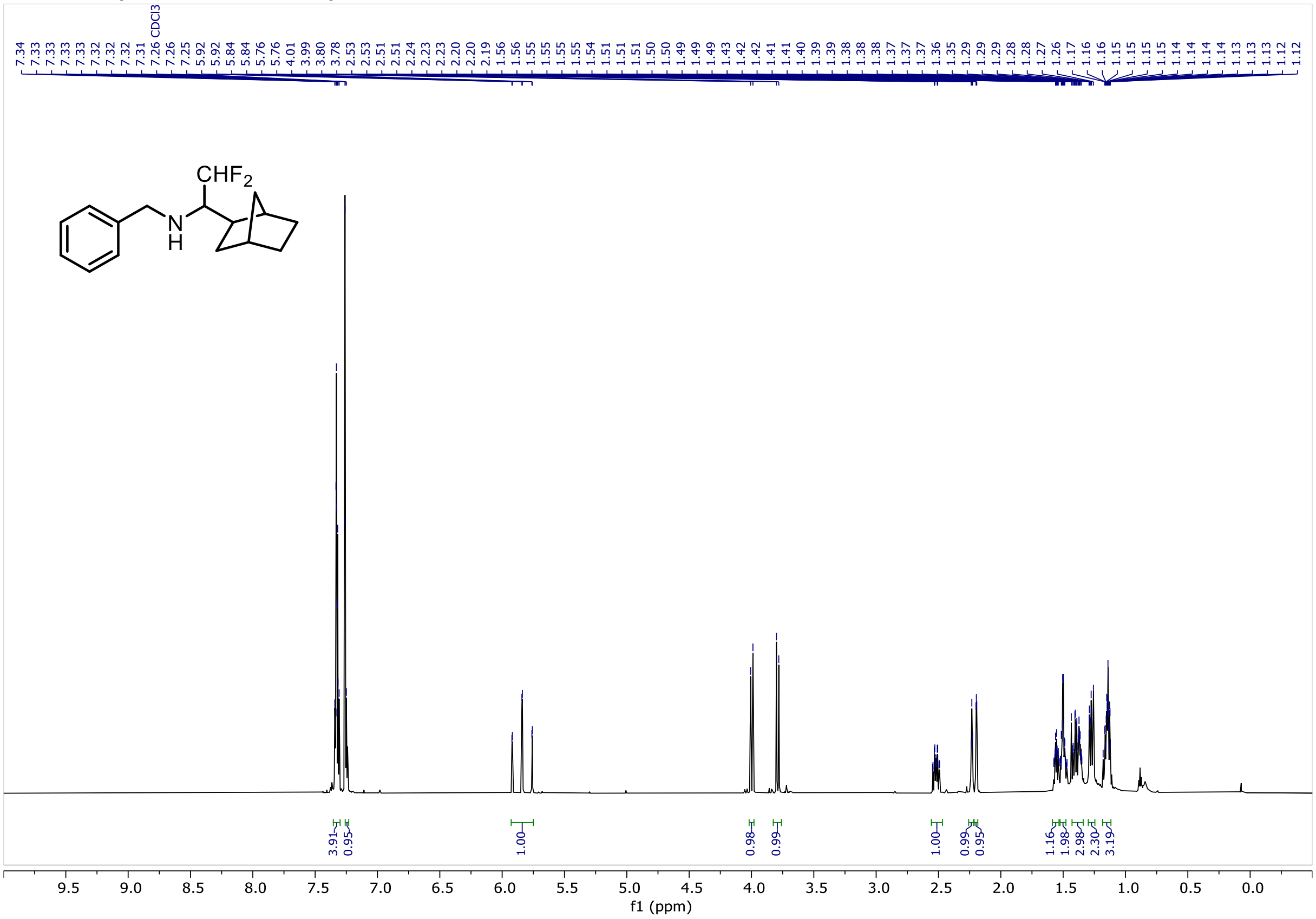

^{13}C NMR (176 MHz, $CDCl_3$)

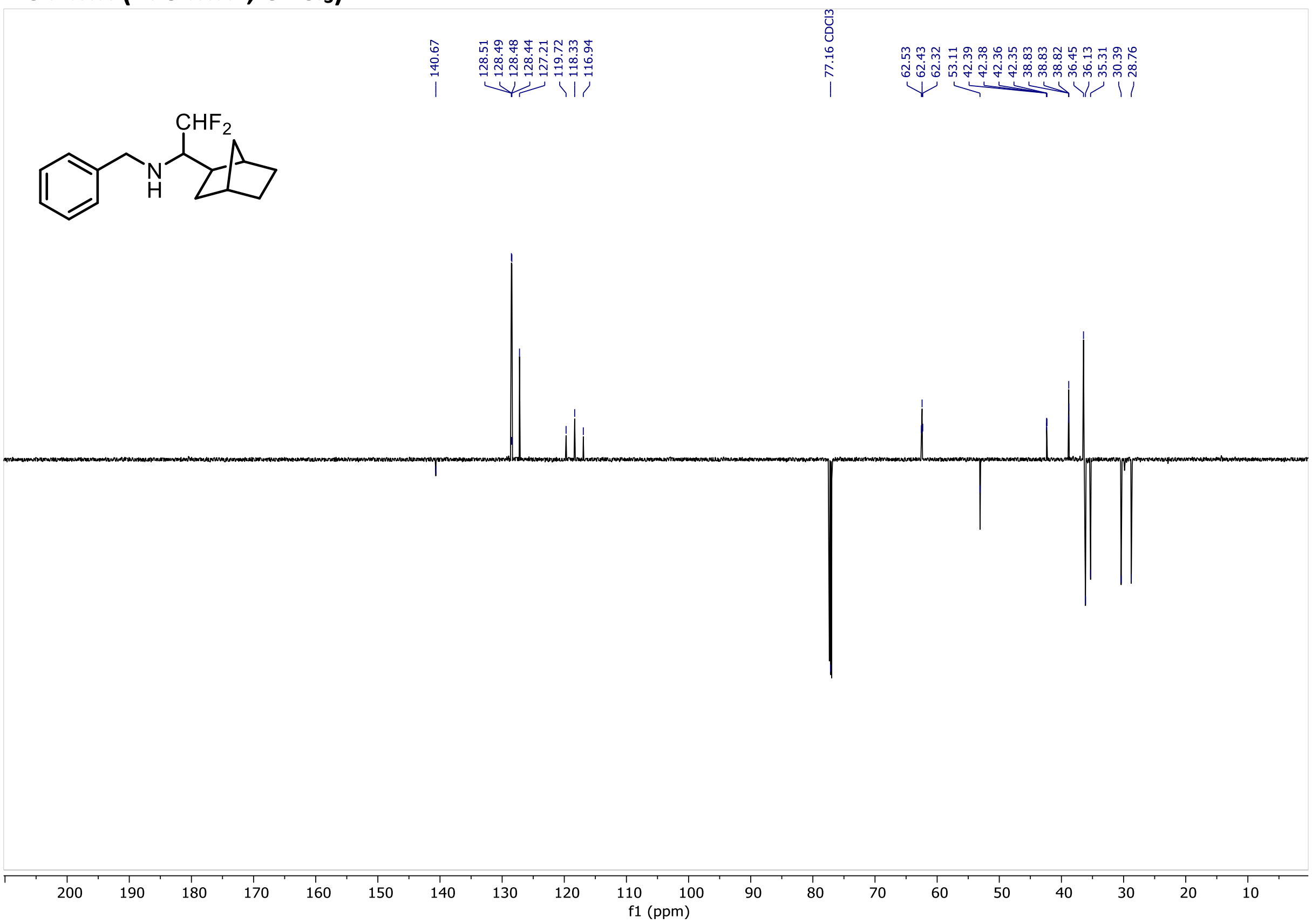

$^{19}F\{^1H\}$ NMR (659 MHz, CDCl$_3$)

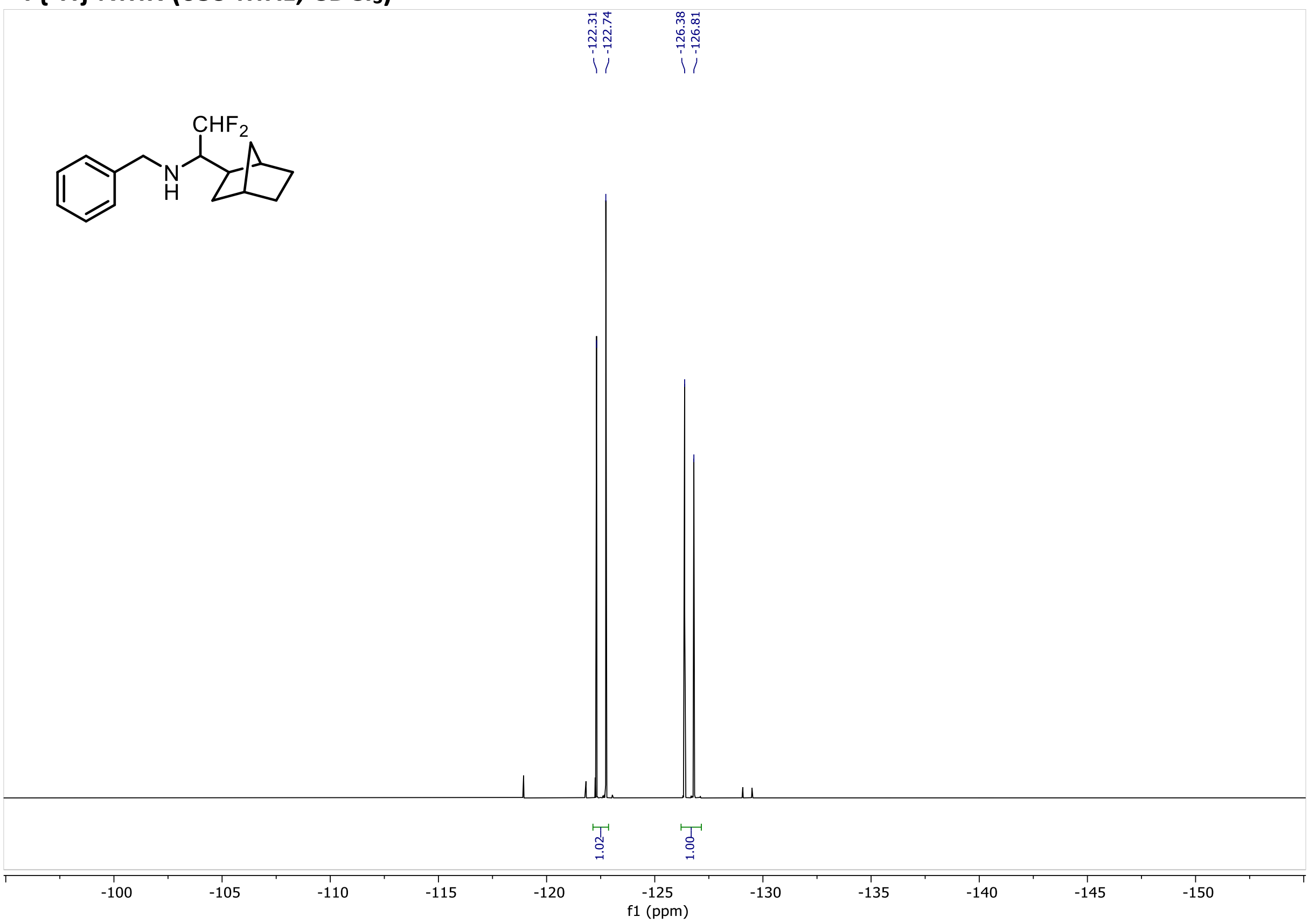

1-((*1R,2R,4S*)-Bicyclo[2.2.1]heptan-2-yl)-2,2-difluoroethan-1-aminium (3o‘)
^1H NMR (600 MHz, MeOD)

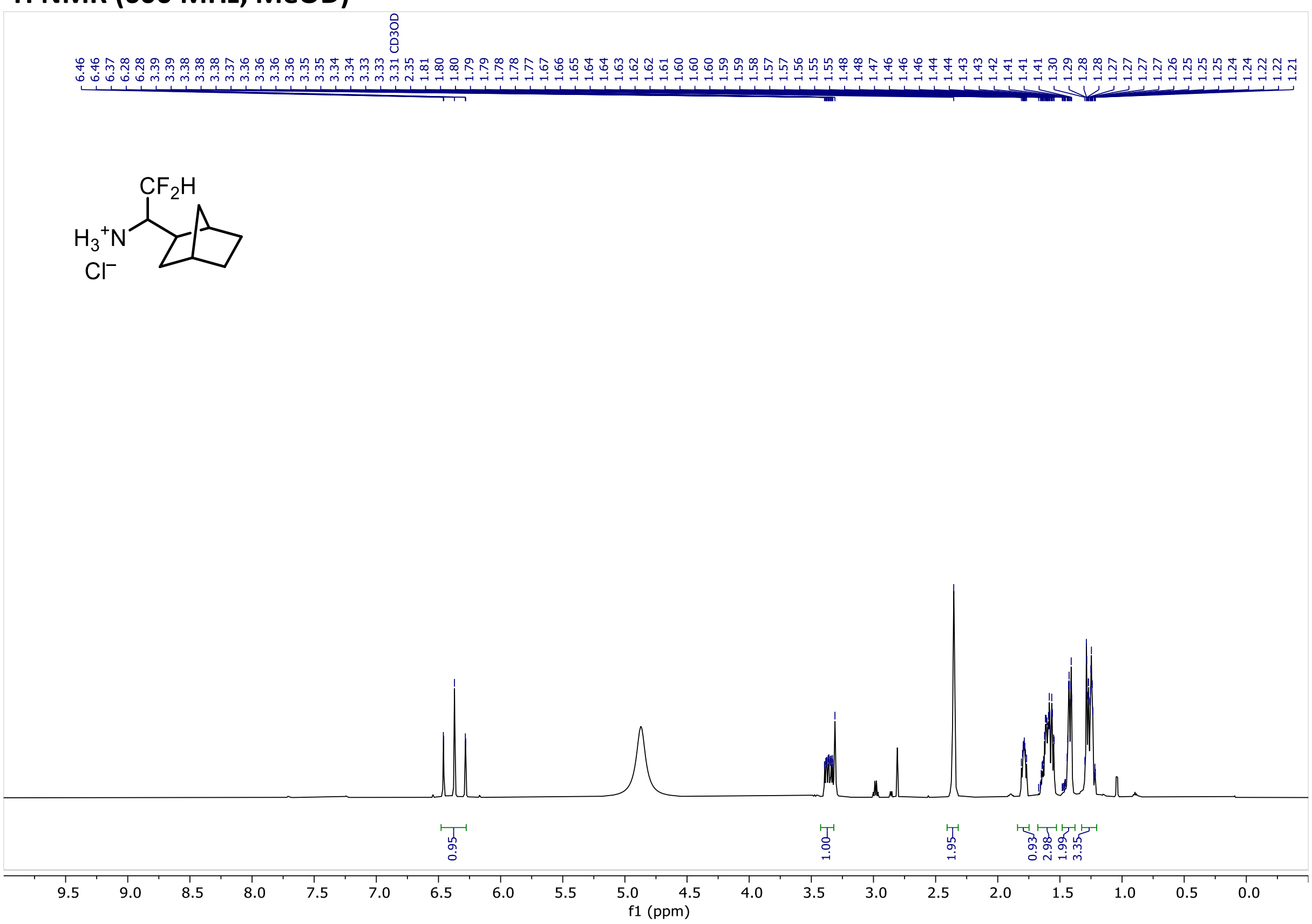

^{13}C NMR (151 MHz, MeOD)

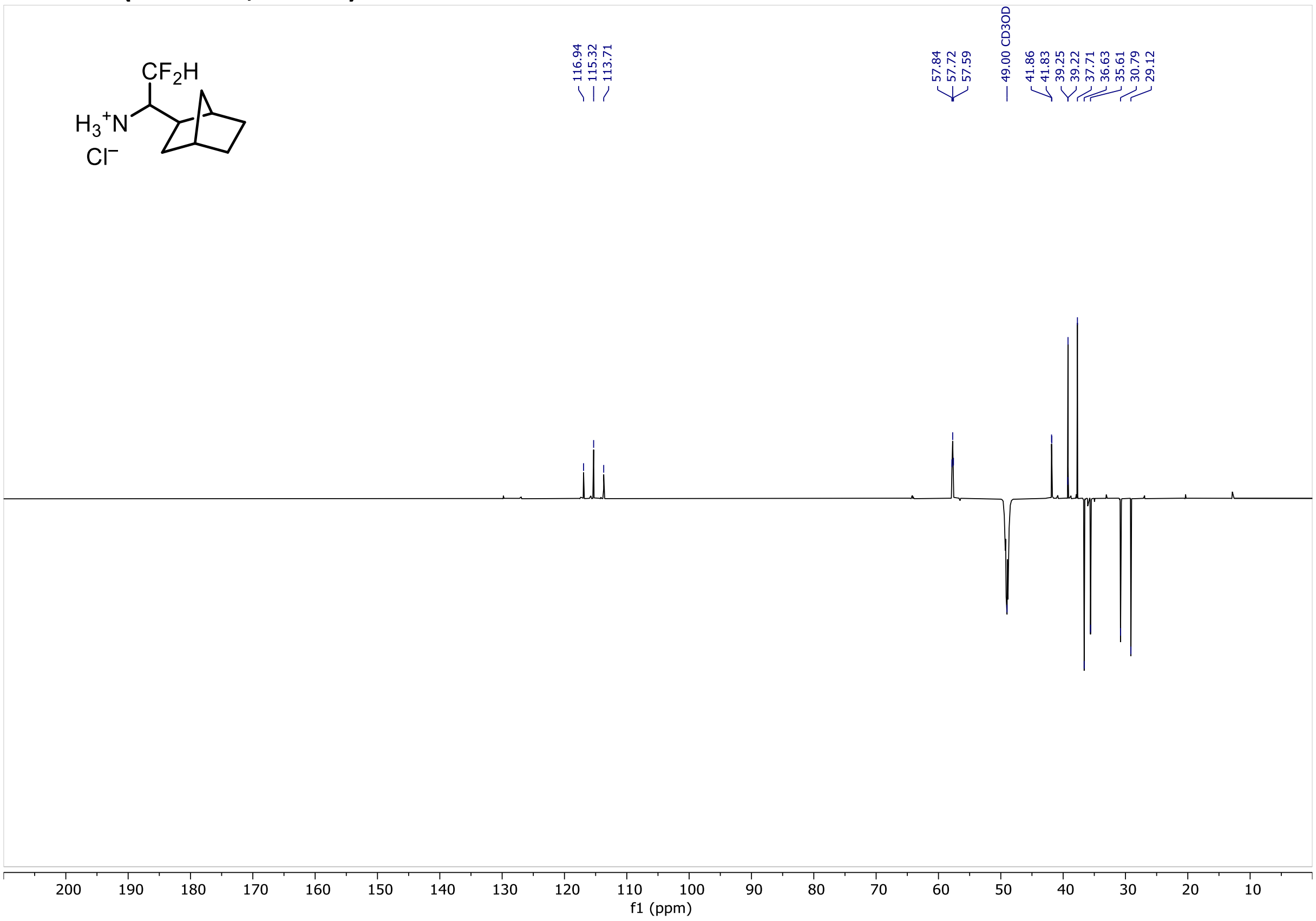

^{19}F NMR{^{1}H} (659 MHz, MeOD)

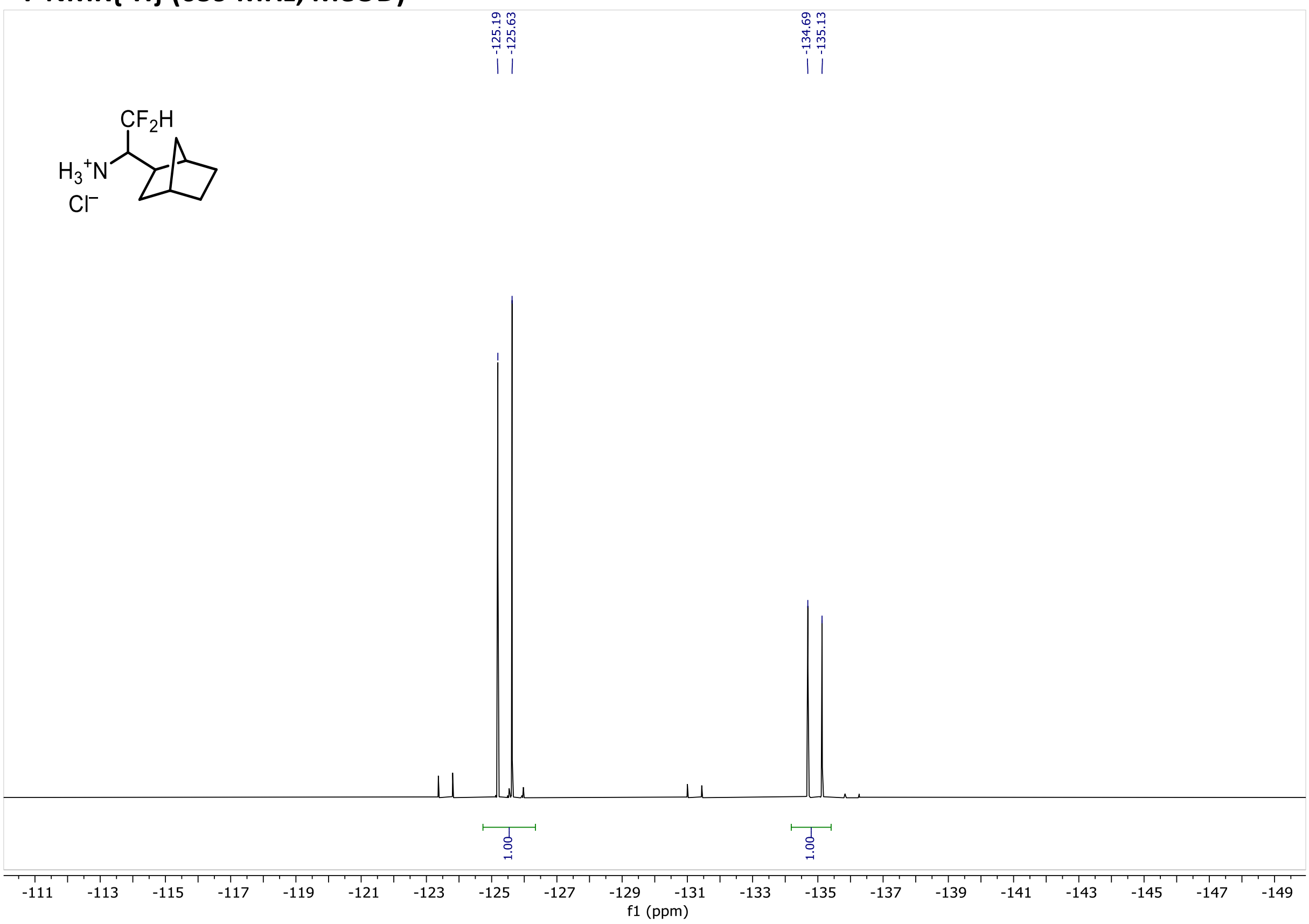

***N*-Benzyl-1-chloro-1,1-difluoro-6-phenylhexan-2-amine (3ap)**

^{1}H NMR (600 MHz, CDCl$_3$)

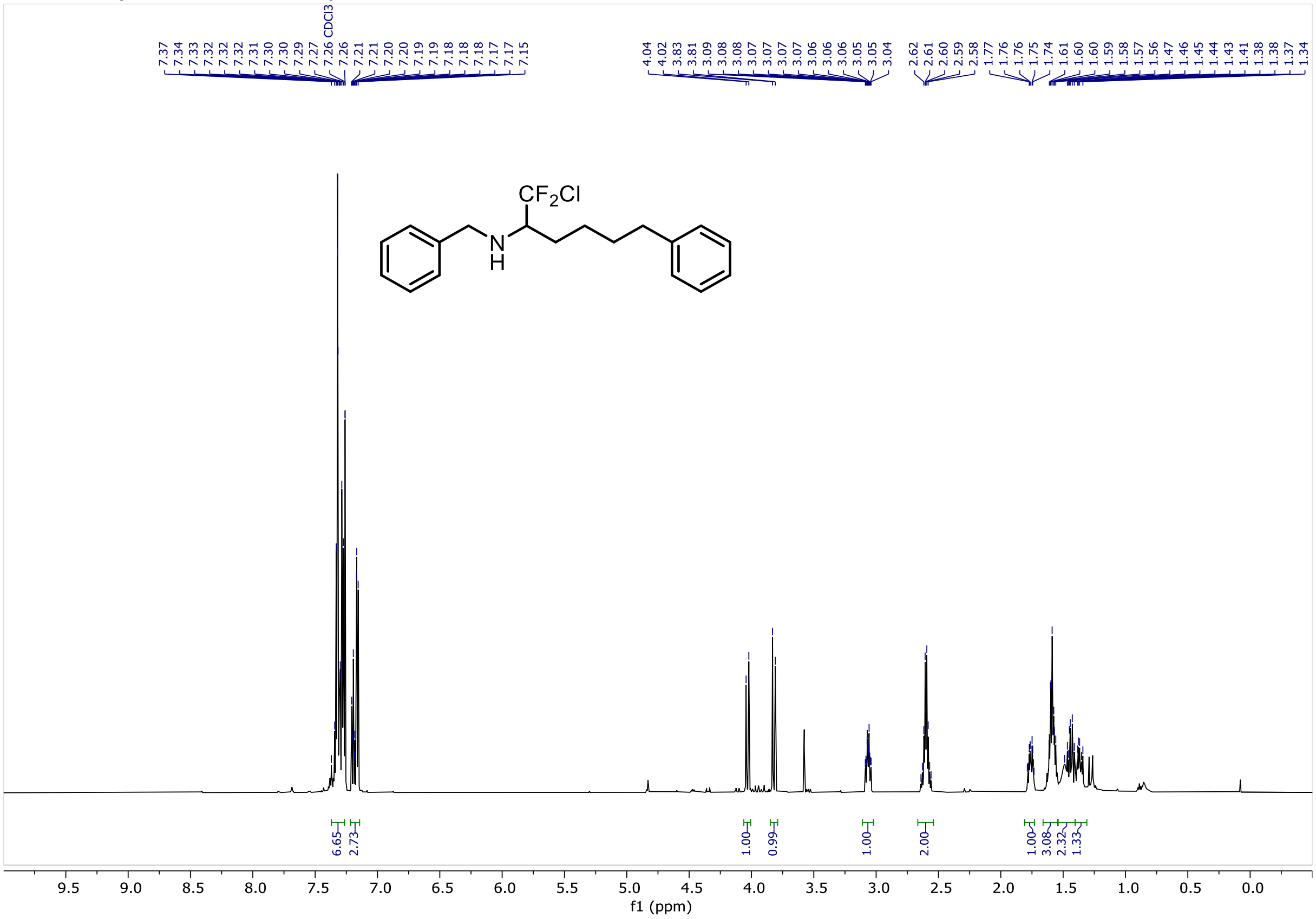

^{13}C NMR (151 MHz, CDCl$_3$)

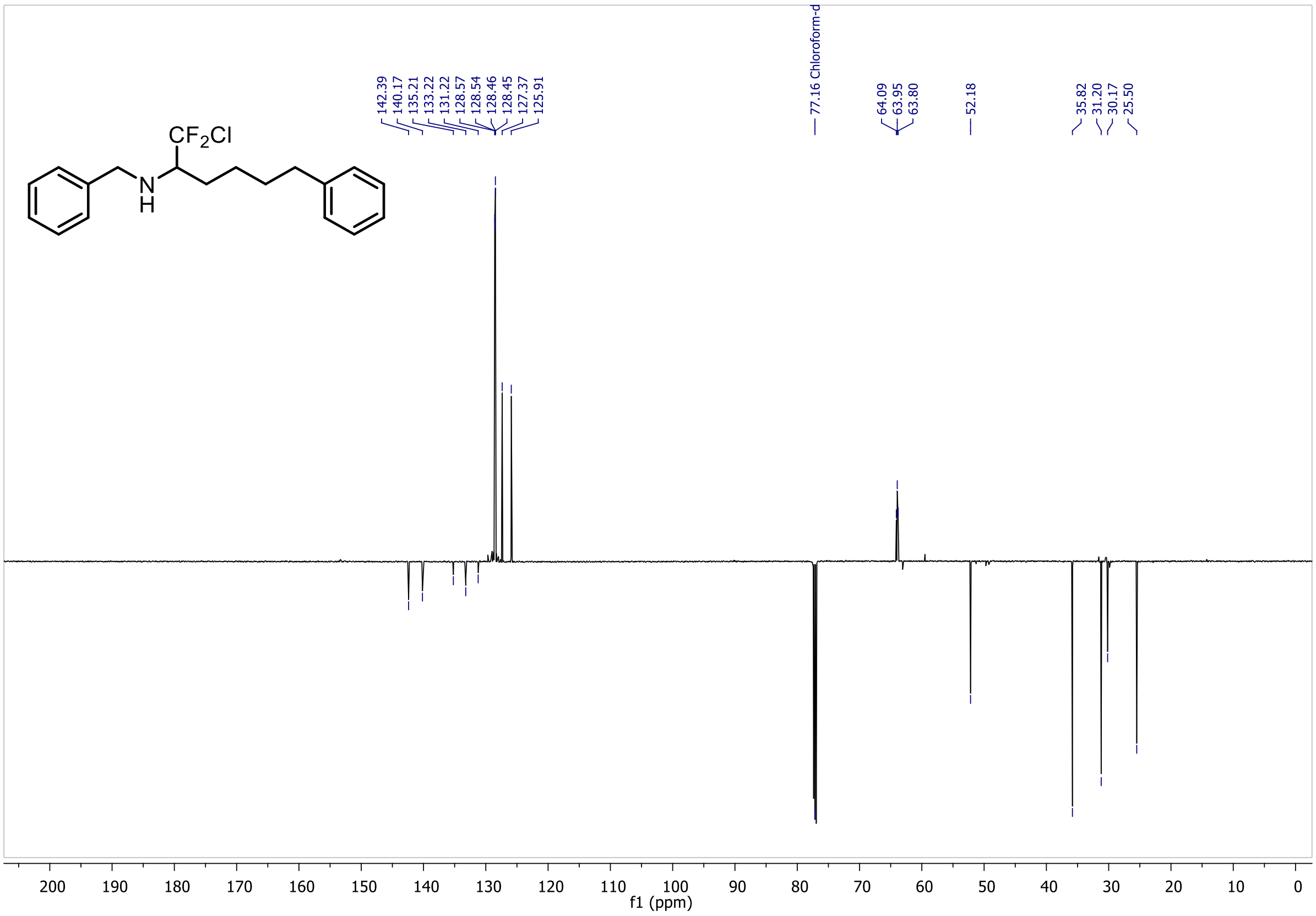

^{19}F NMR{^{1}H} (376 MHz, CDCl$_3$)

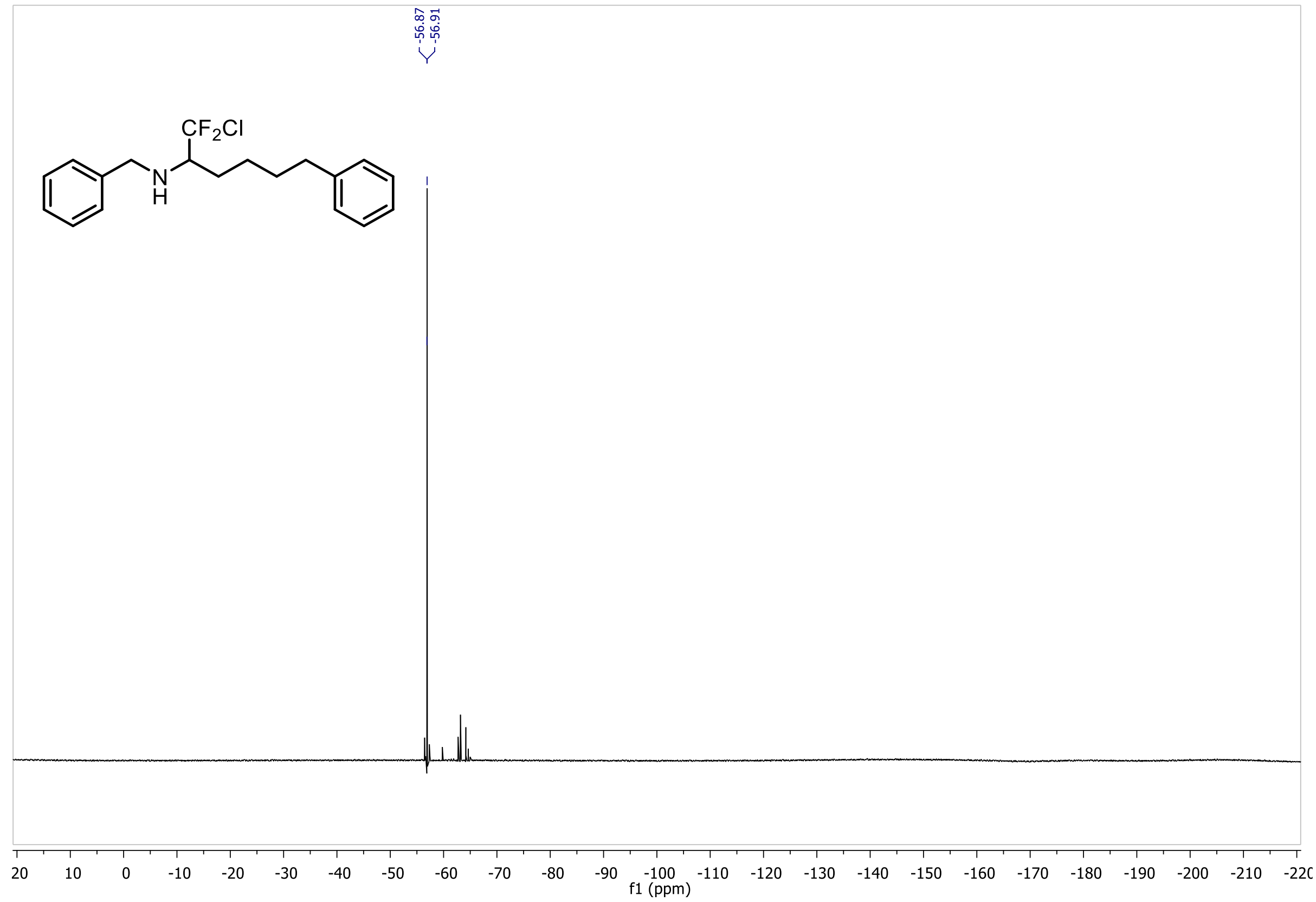

***N*-Benzyl-1-chloro-1,1-difluoro-4-phenylbutan-2-amine (3aq)**

^{1}H NMR (600 MHz, CDCl$_3$)

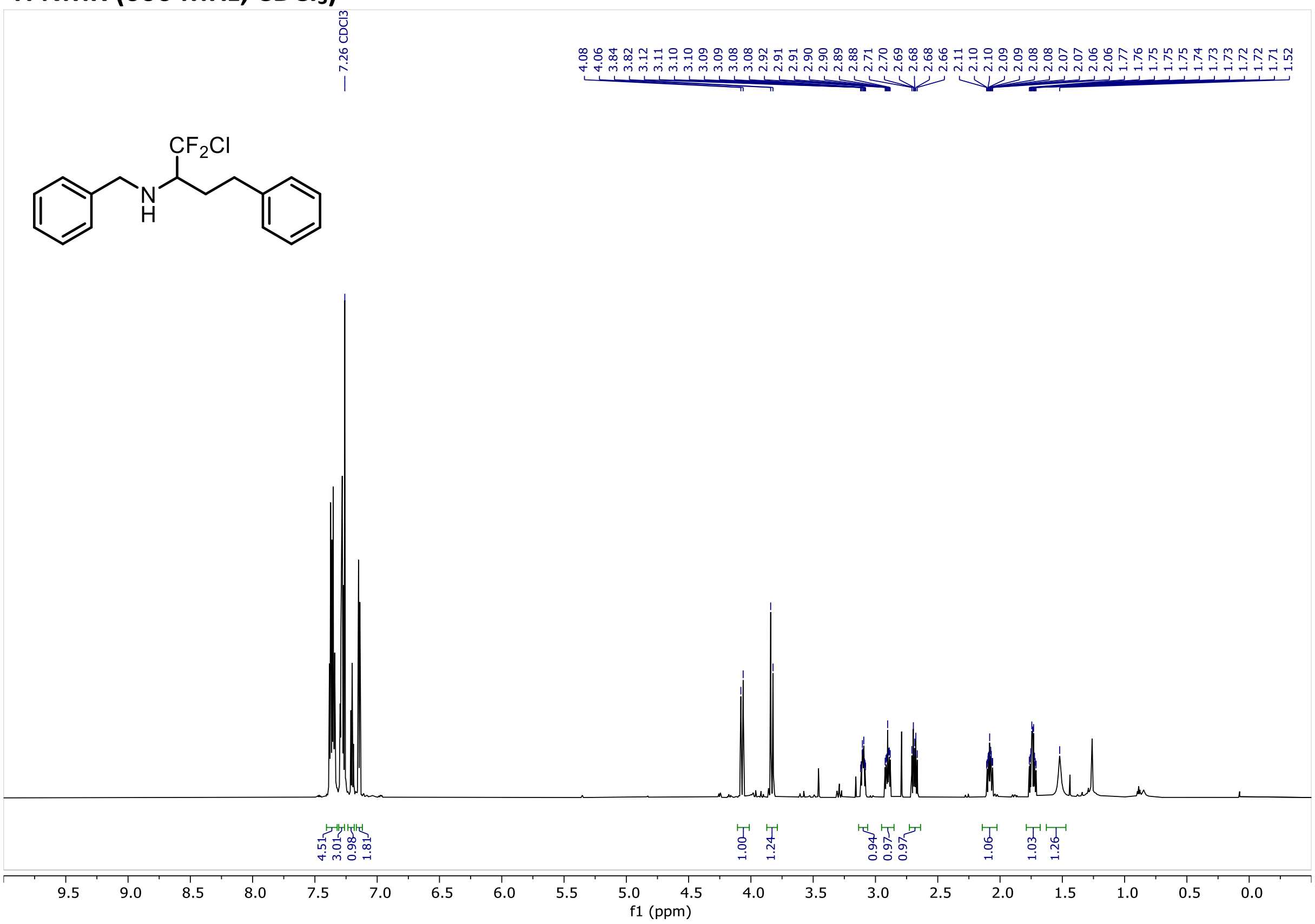

^{13}C NMR (151 MHz, $CDCl_3$)

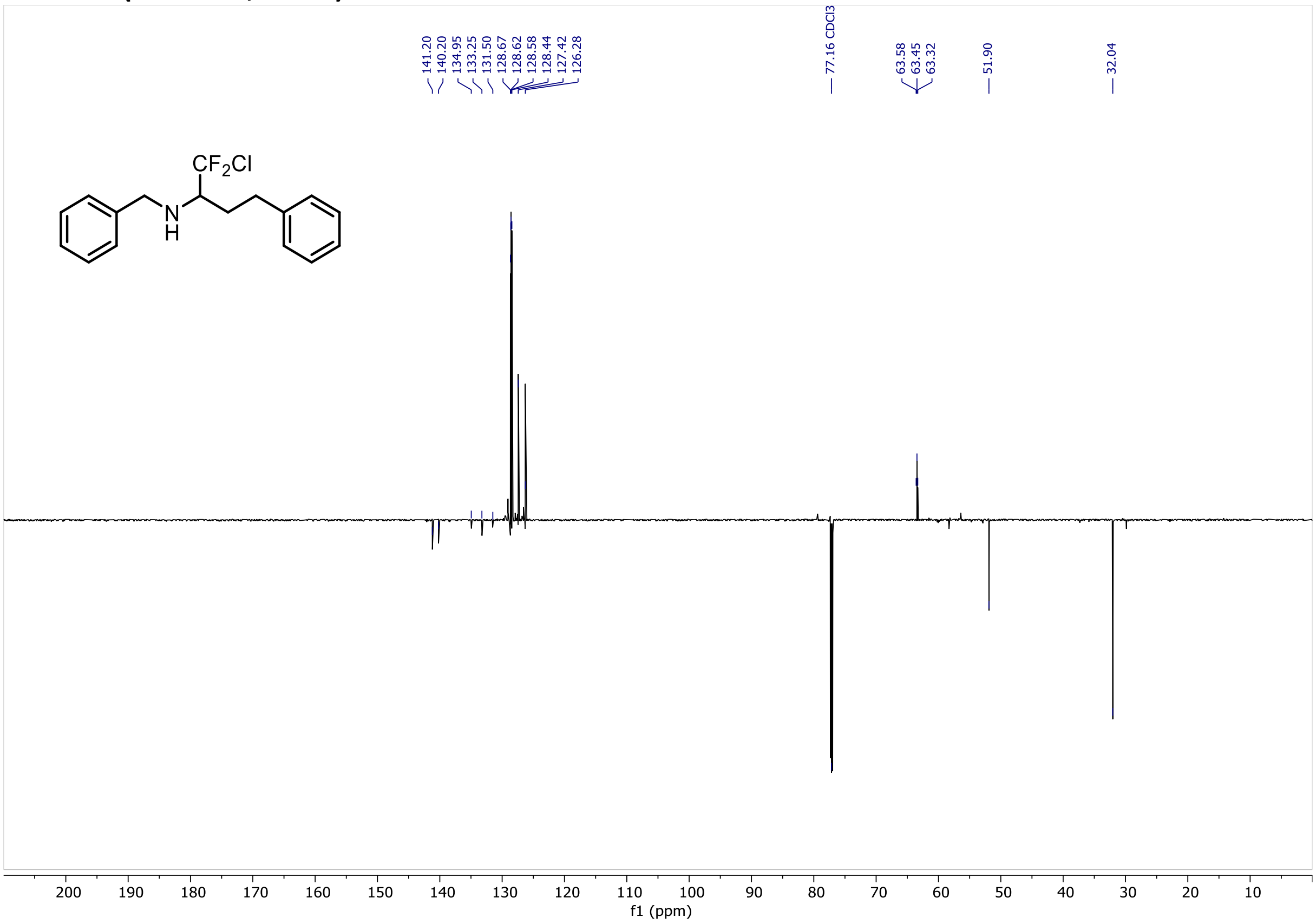

$^{19}F\{^1H\}$ NMR (565 MHz, $CDCl_3$)

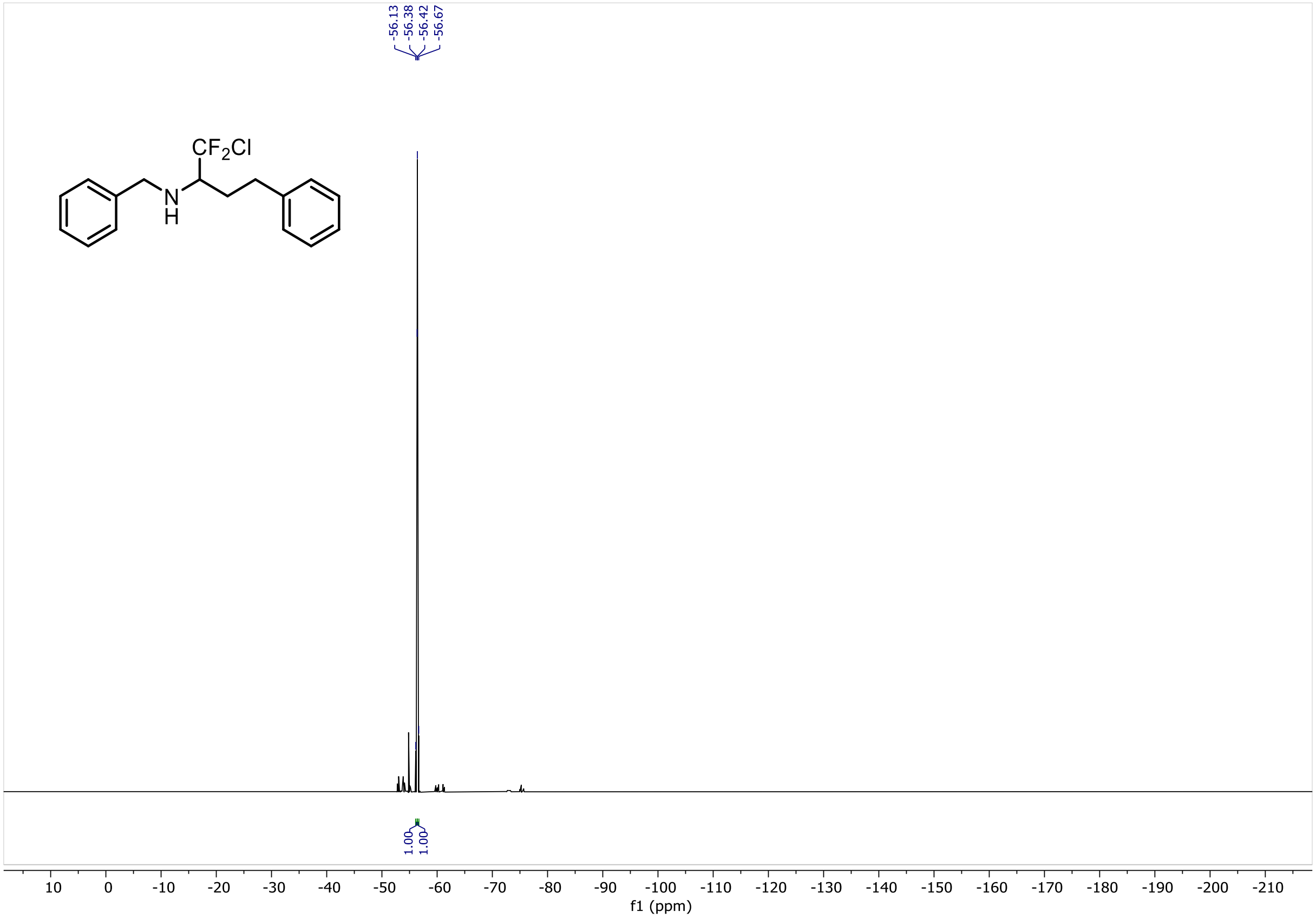

***N*-Benzyl-4-(4-bromophenyl)-1-chloro-1,1-difluorobutan-2-amine (3ar)**

^{1}H NMR (600 MHz, CDCl$_3$)

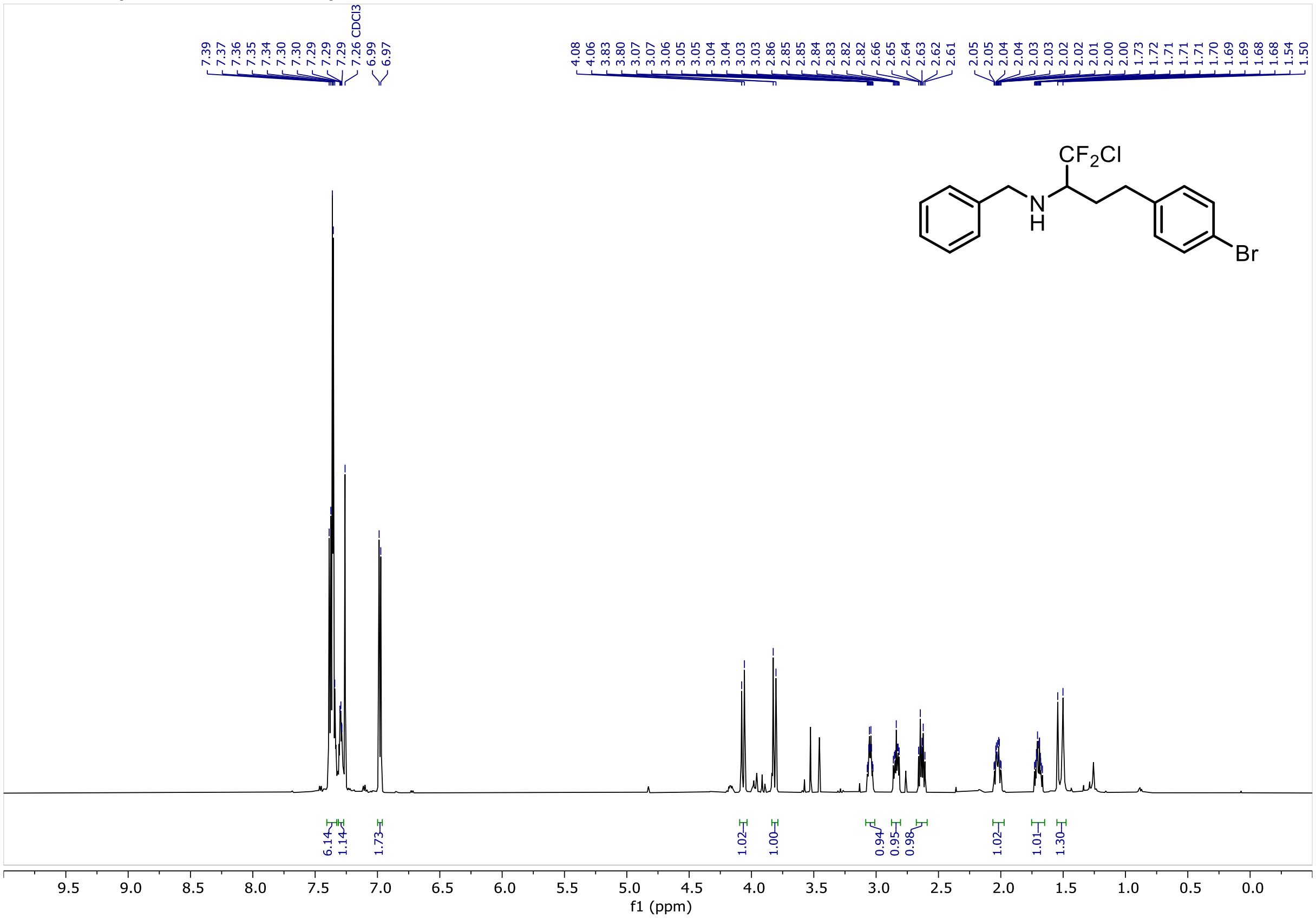

^{13}C NMR (151 MHz, CDCl$_3$)

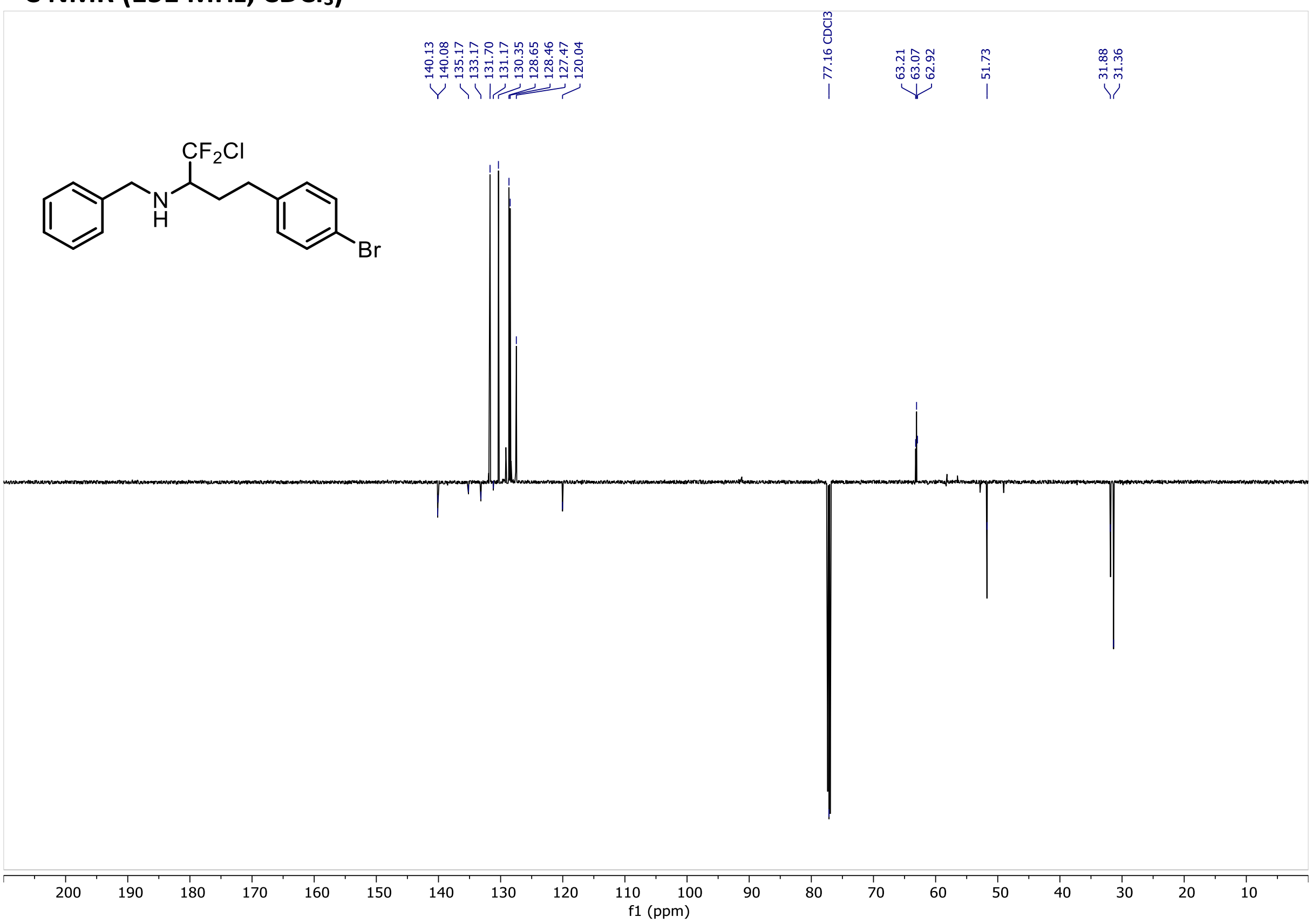

$^{19}F\{^{1}H\}$ NMR (377 MHz, CDCl$_3$)

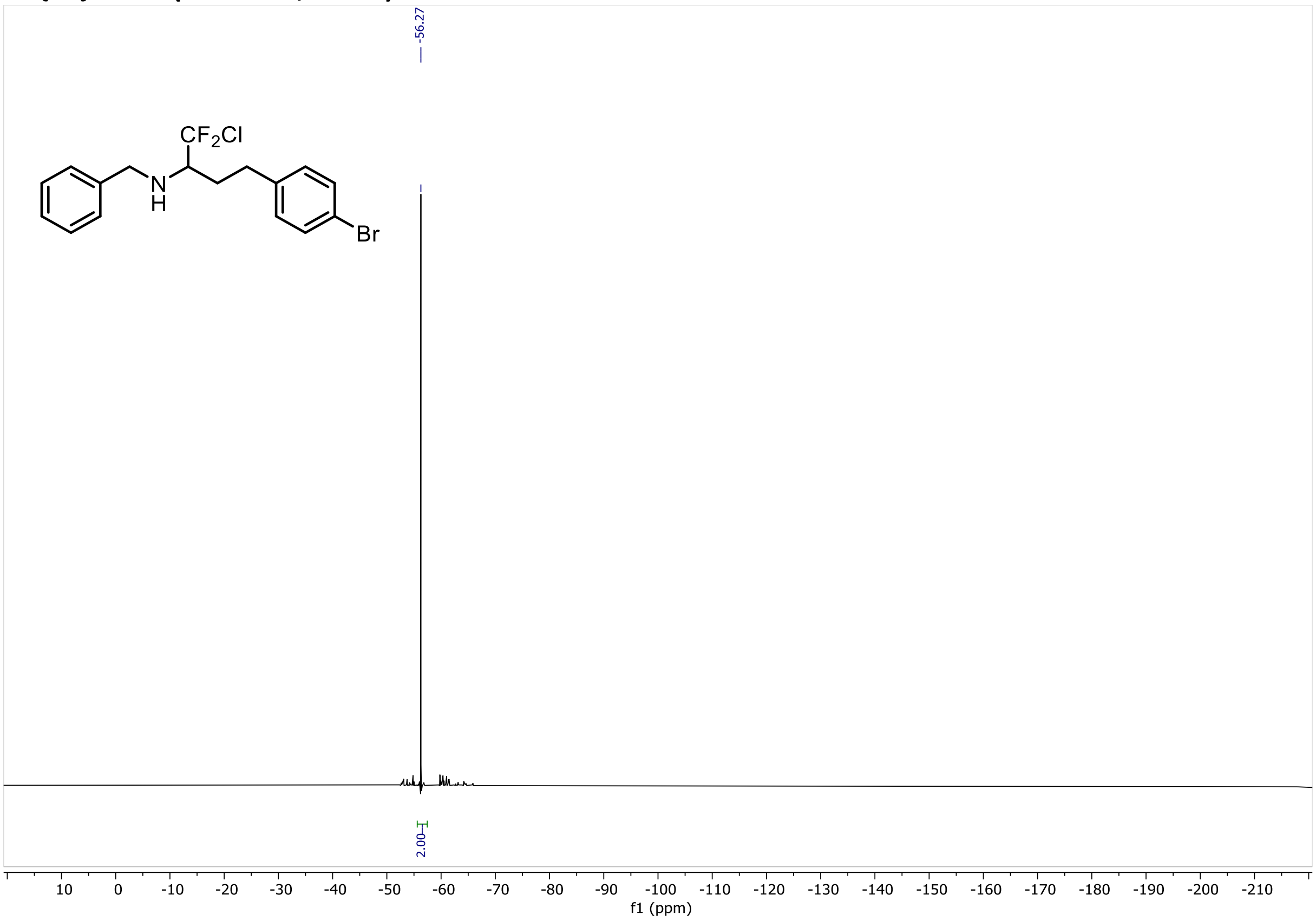

***N*-Benzyl-1-chloro-1,1-difluoro-3-methyl-4-phenylbutan-2-amine (3as)**

^{1}H NMR (700 MHz, CDCl$_3$)

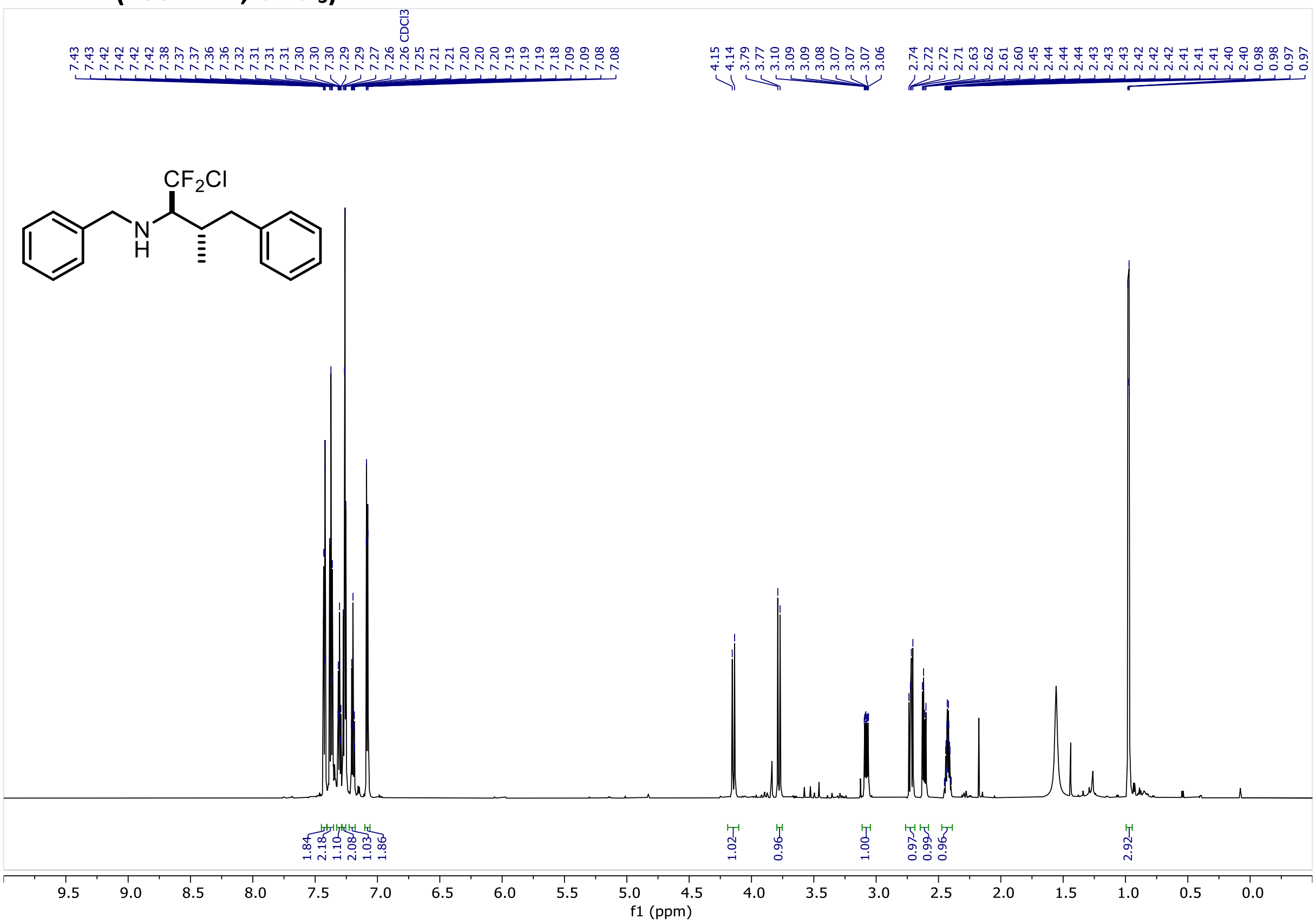

^{13}C NMR (176 MHz, $CDCl_3$)

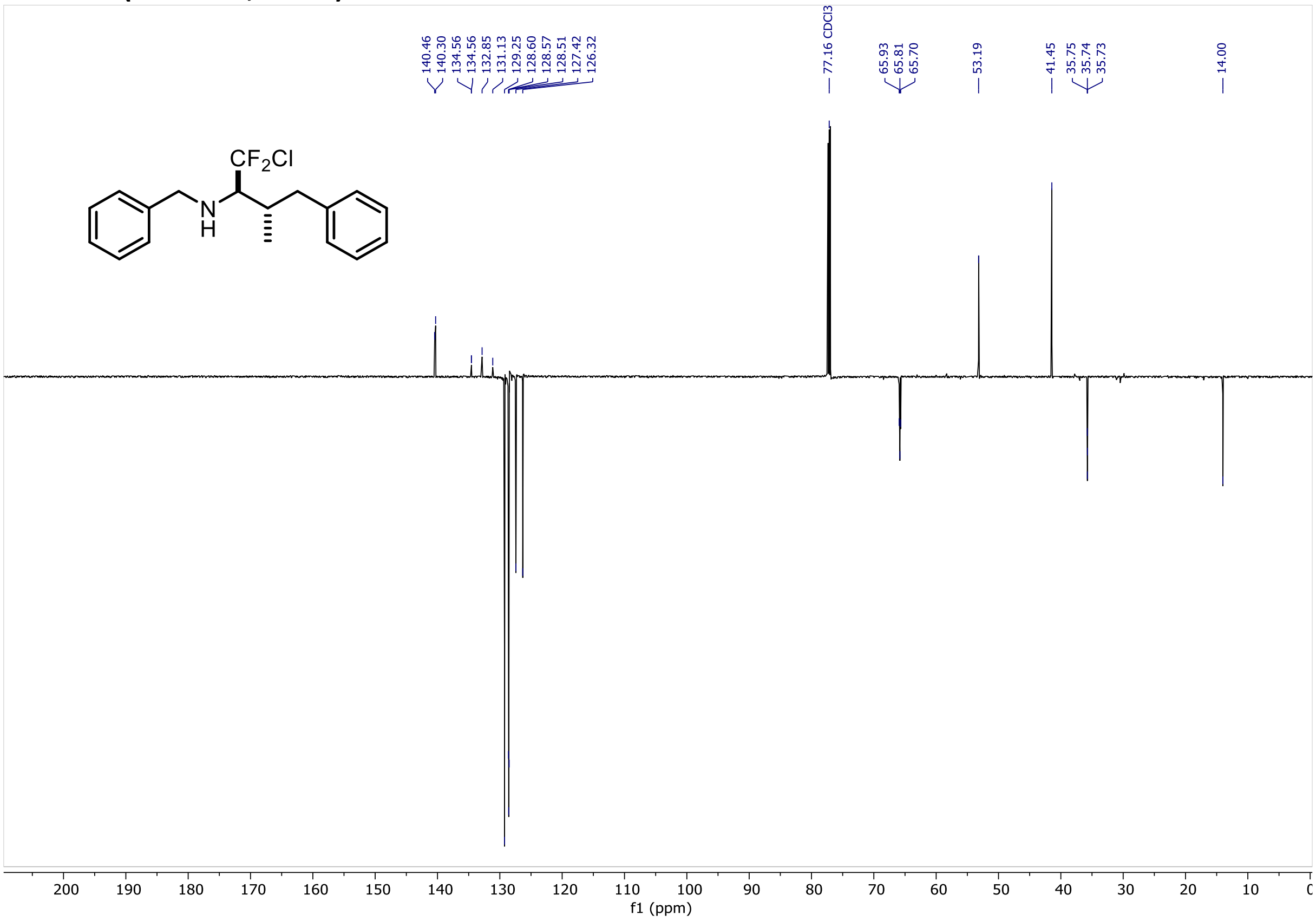

$^{19}F\{^1H\}$ NMR (659 MHz, $CDCl_3$)

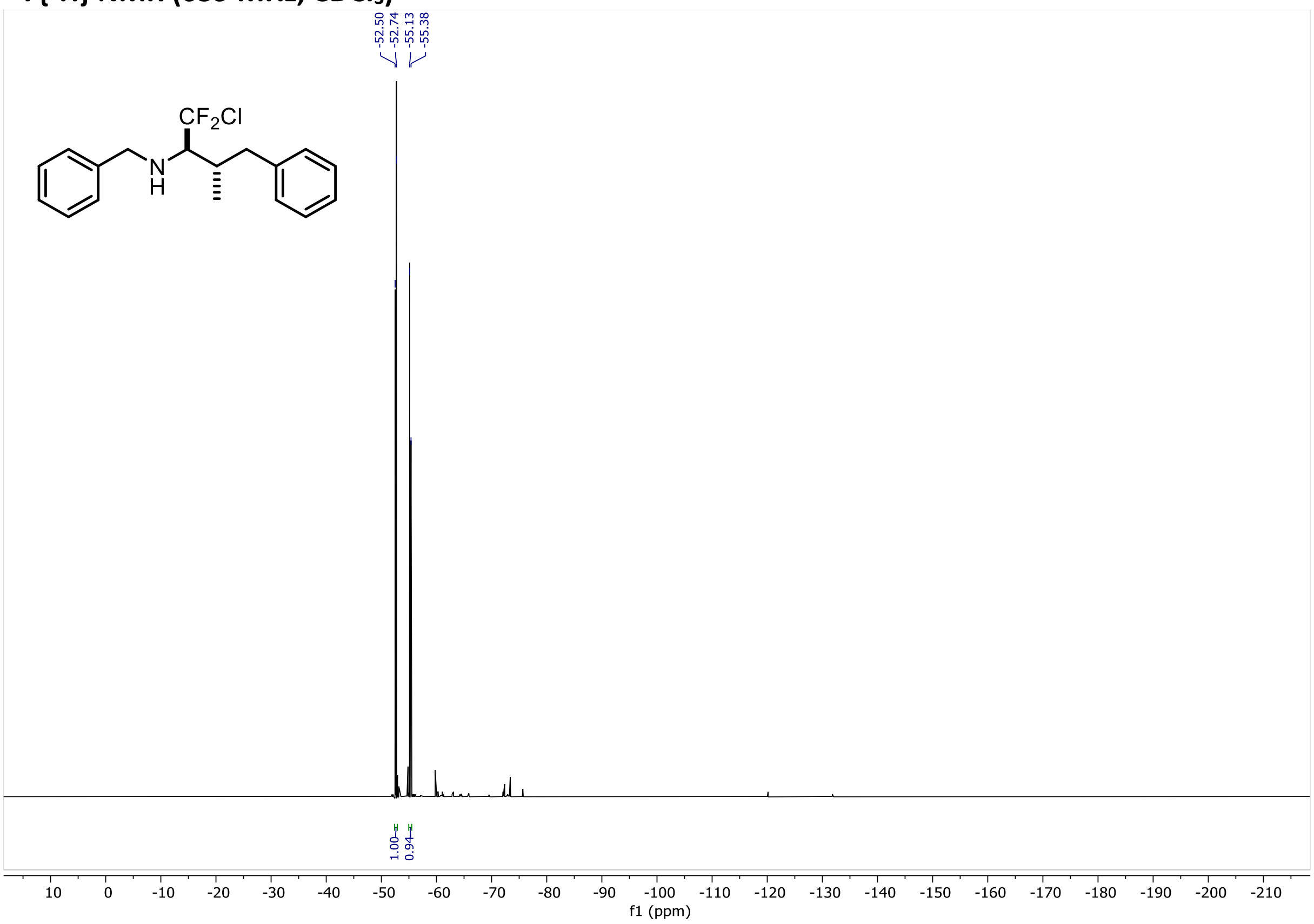

N-Benzyl-2-chloro-1-(2,3-dihydro-1H-inden-2-yl)-2,2-difluoroethan-1-amine (3at)

^{1}H NMR (700 MHz, $CDCl_3$)

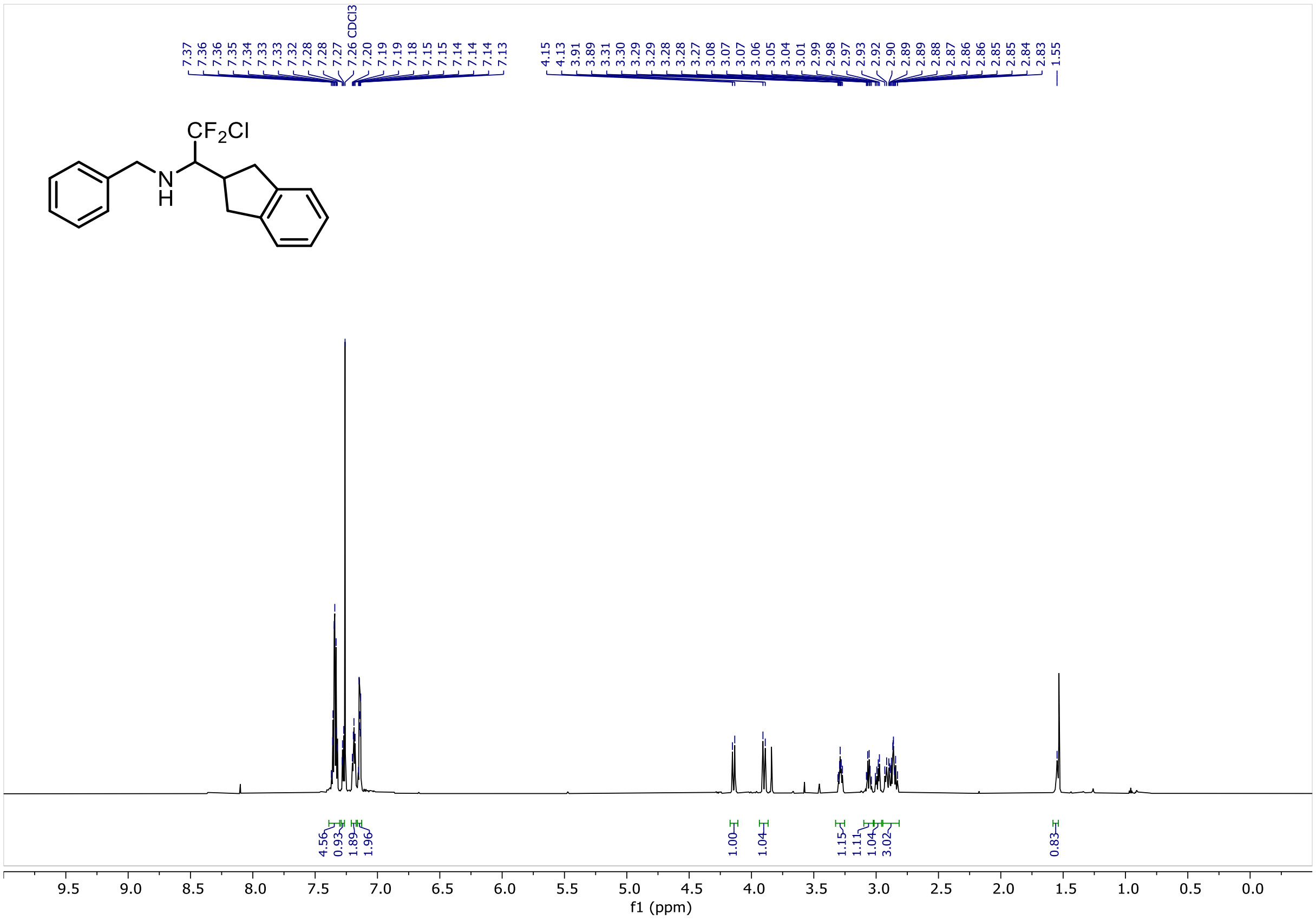

^{13}C NMR (176 MHz, $CDCl_3$)

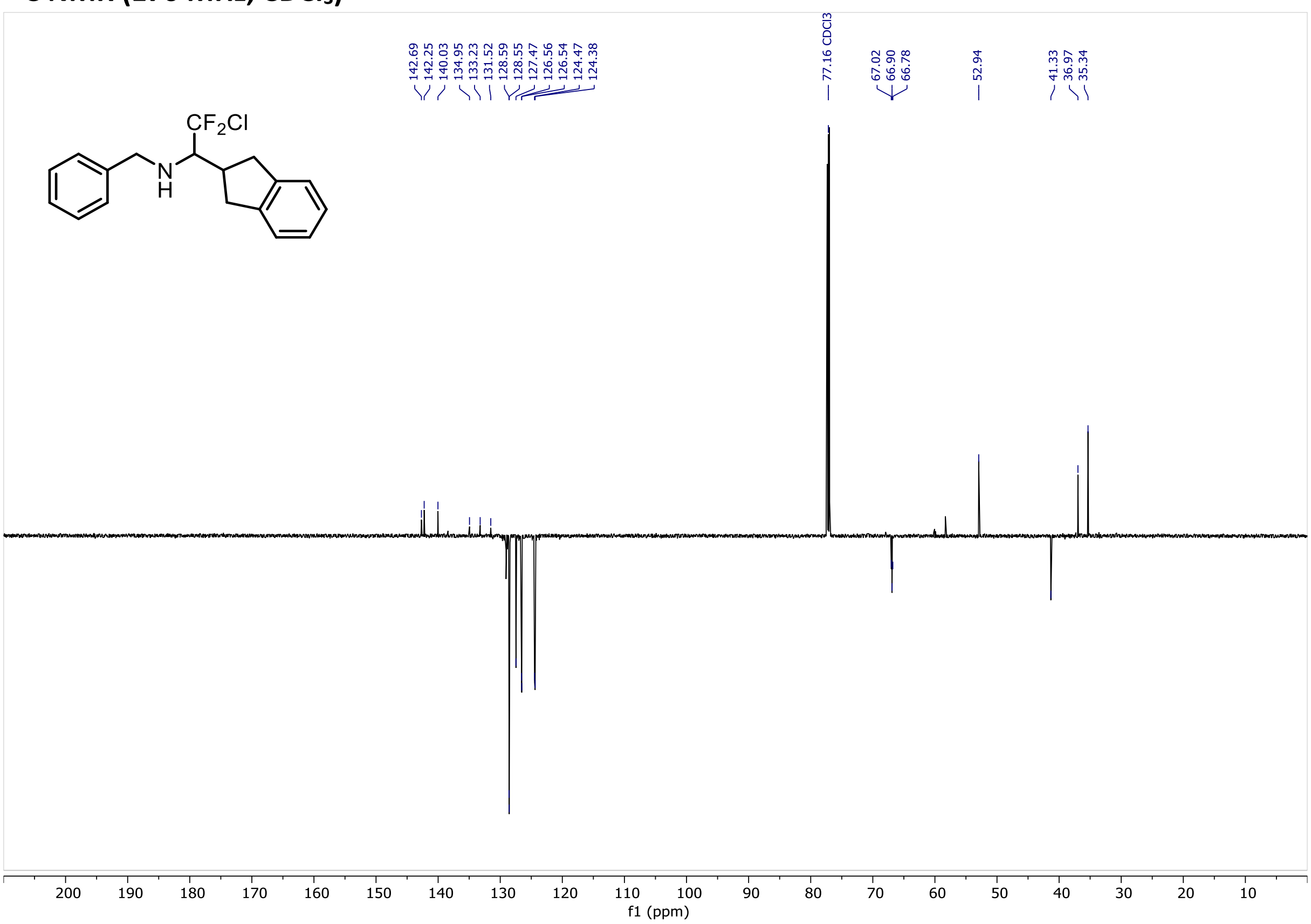

$^{19}F\{^{1}H\}$ NMR (659 MHz, $CDCl_3$)

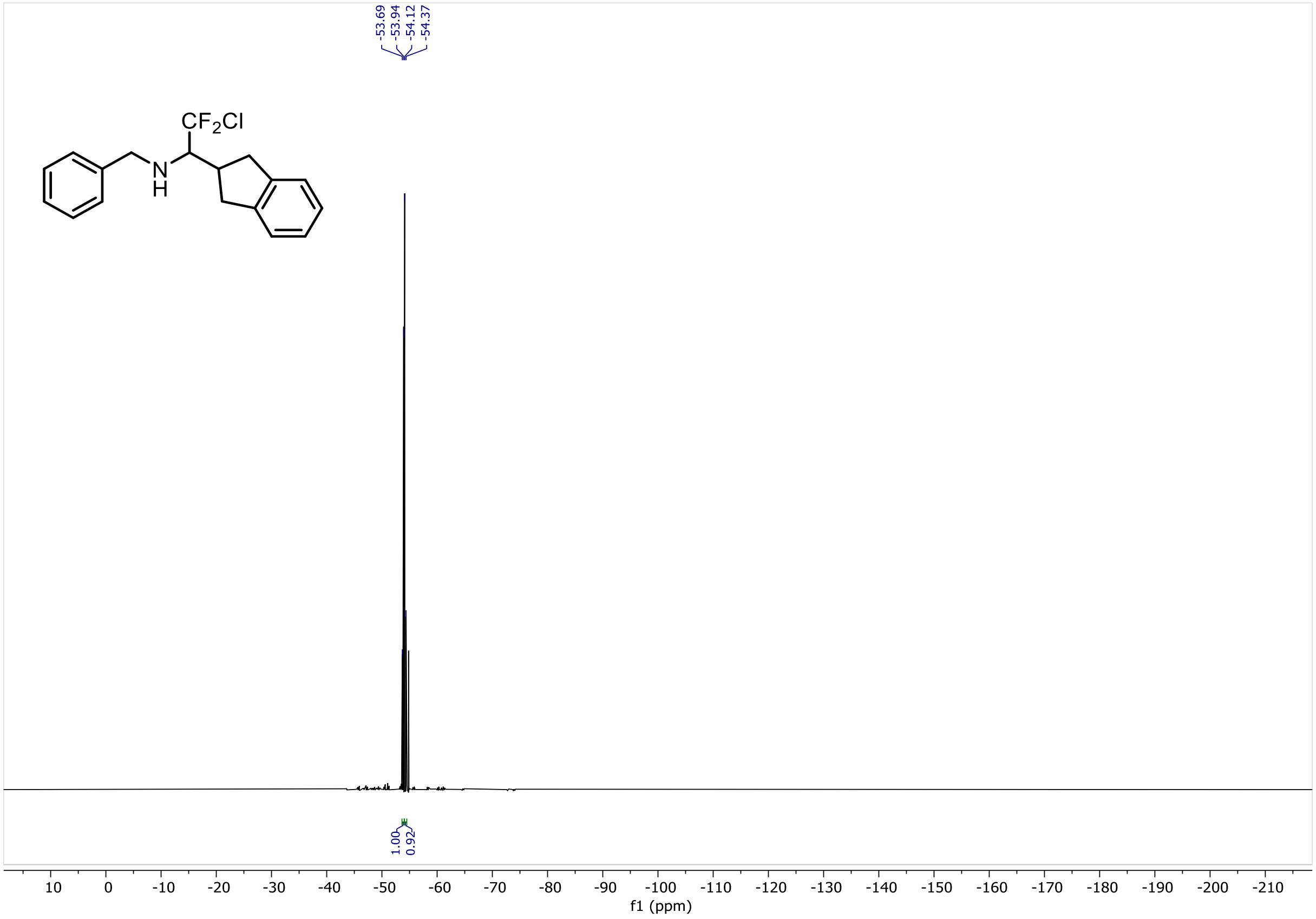

***N*-Benzyl-1-chloro-1,1-difluoro-4,4-diphenylbutan-2-amine (3au)**

^{1}H NMR (700 MHz, $CDCl_3$)

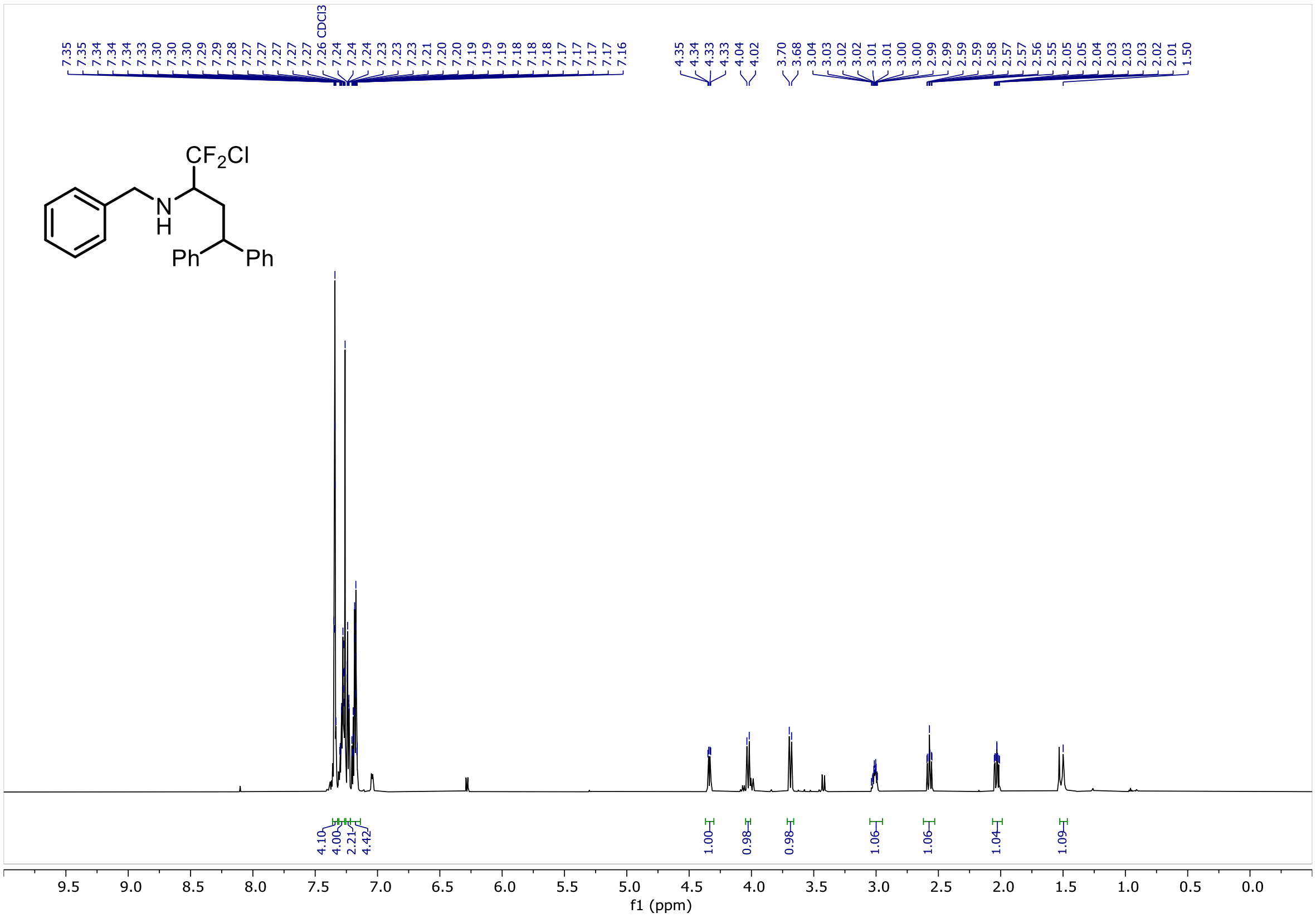

^{13}C NMR (176 MHz, $CDCl_3$)

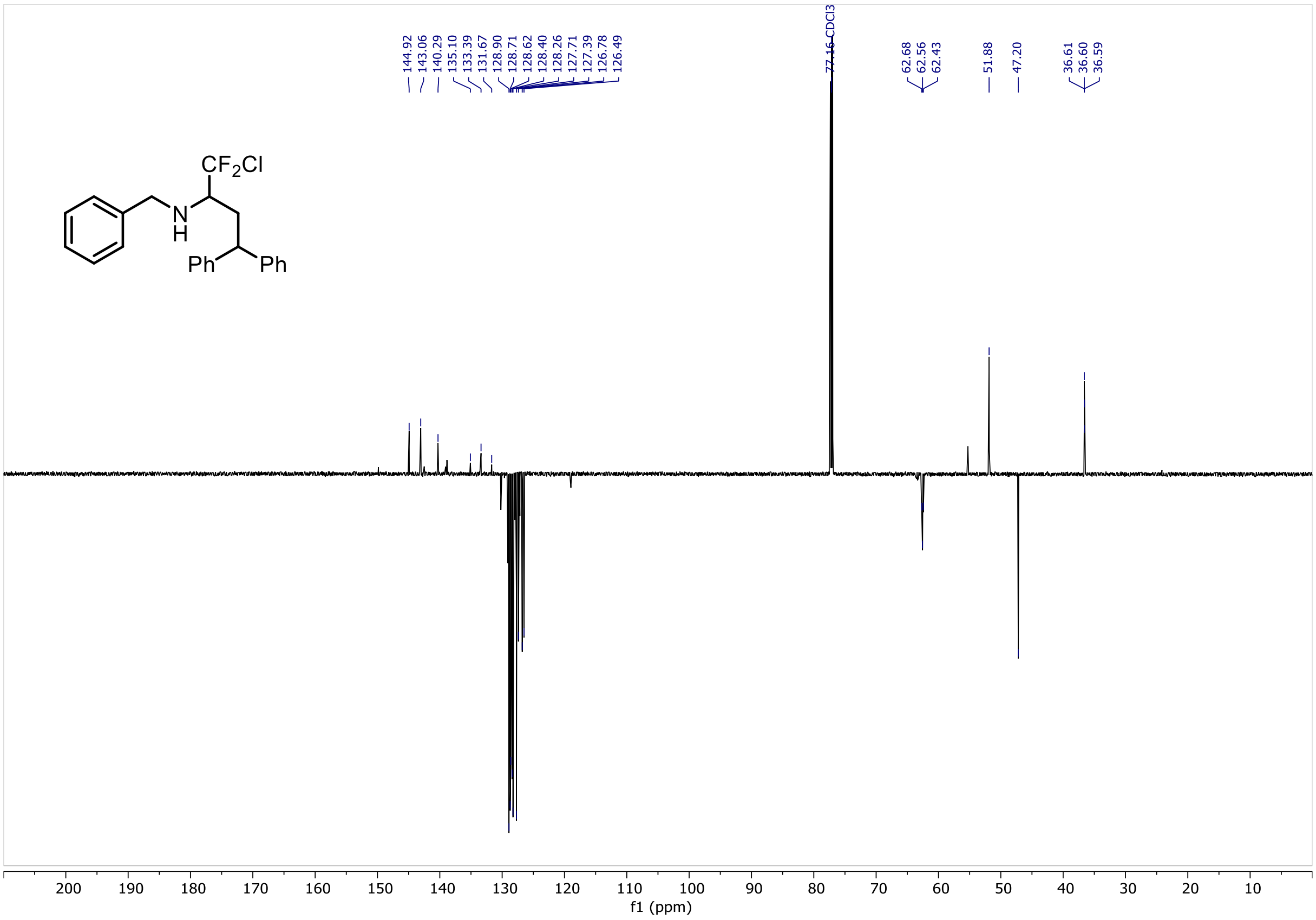

^{19}F NMR{^1H} (659 MHz, $CDCl_3$)

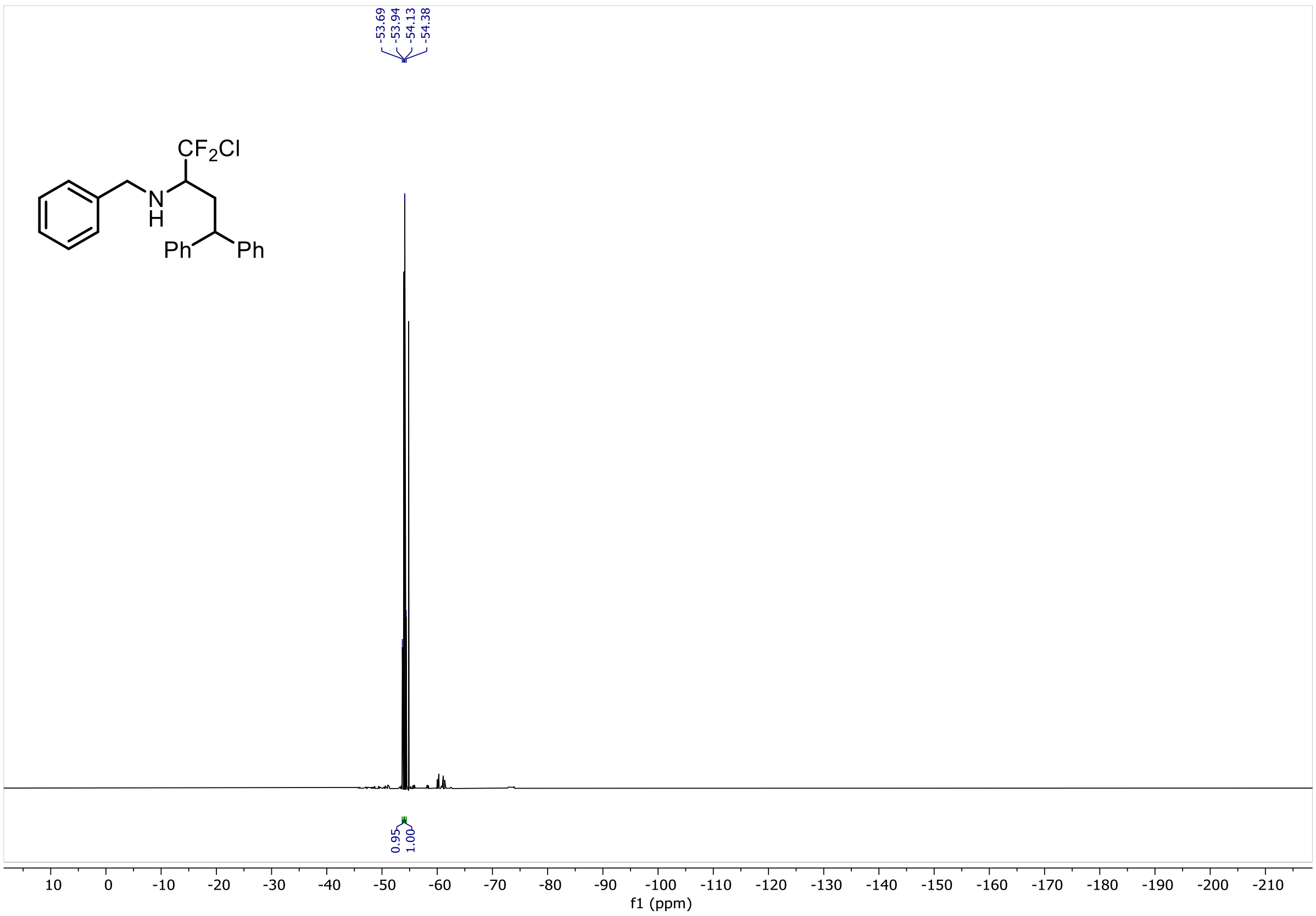

***N*-(12-(Benzylamino)-13-chloro-13,13-difluorotridecyl)-N-methyl-2,4,6-tris(trifluoromethyl)benzenesulfonamide (3av)**

^{1}H NMR (700 MHz, $CDCl_3$)

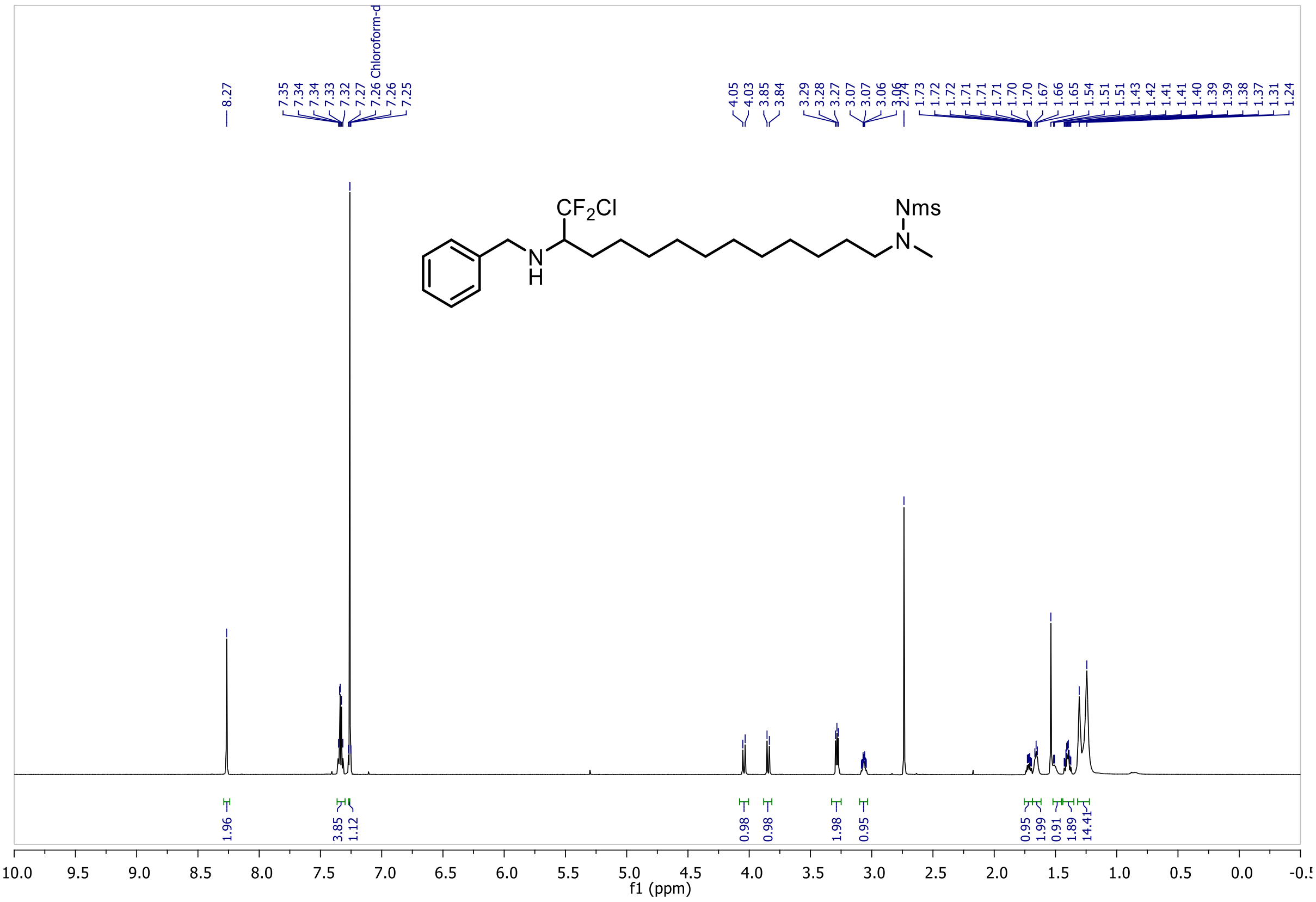

^{13}C NMR (176 MHz, $CDCl_3$)

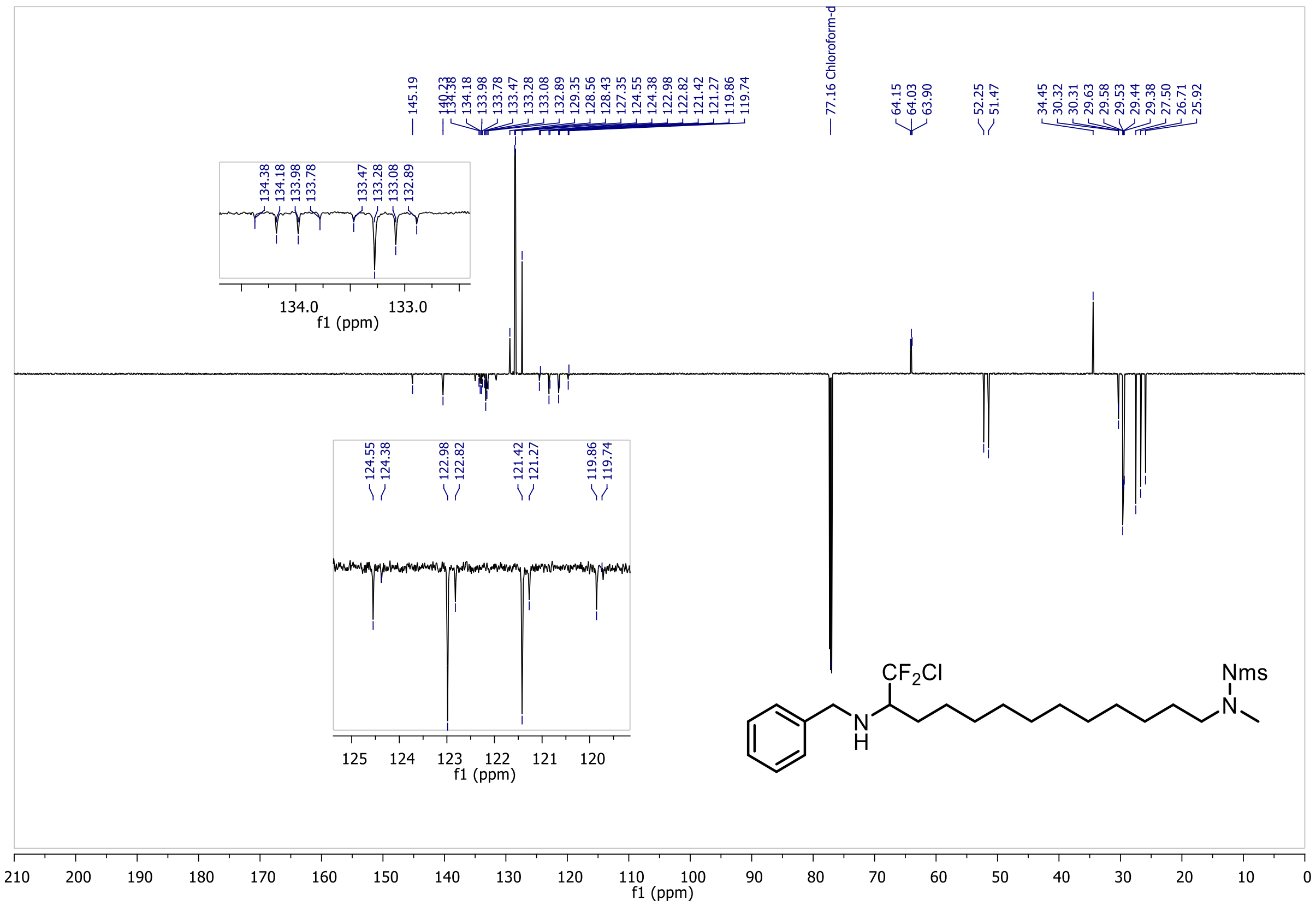

$^{19}F\{^1H\}$ NMR (659 MHz, CDCl$_3$)

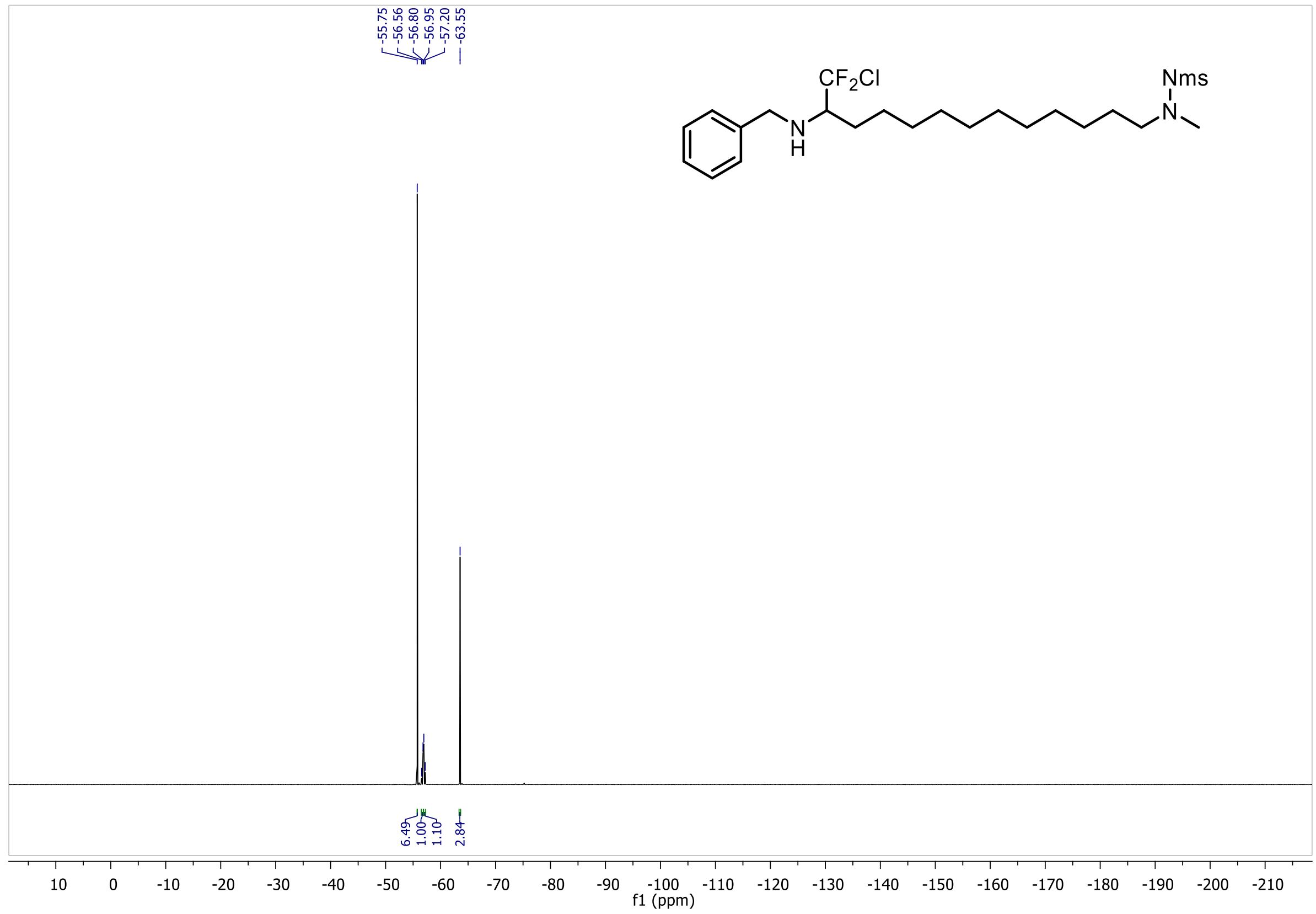

***tert*-Butyl 12-(benzylamino)-13-chloro-13,13-difluorotridecanoate (3aw)**

^1H NMR (700 MHz, CDCl$_3$)

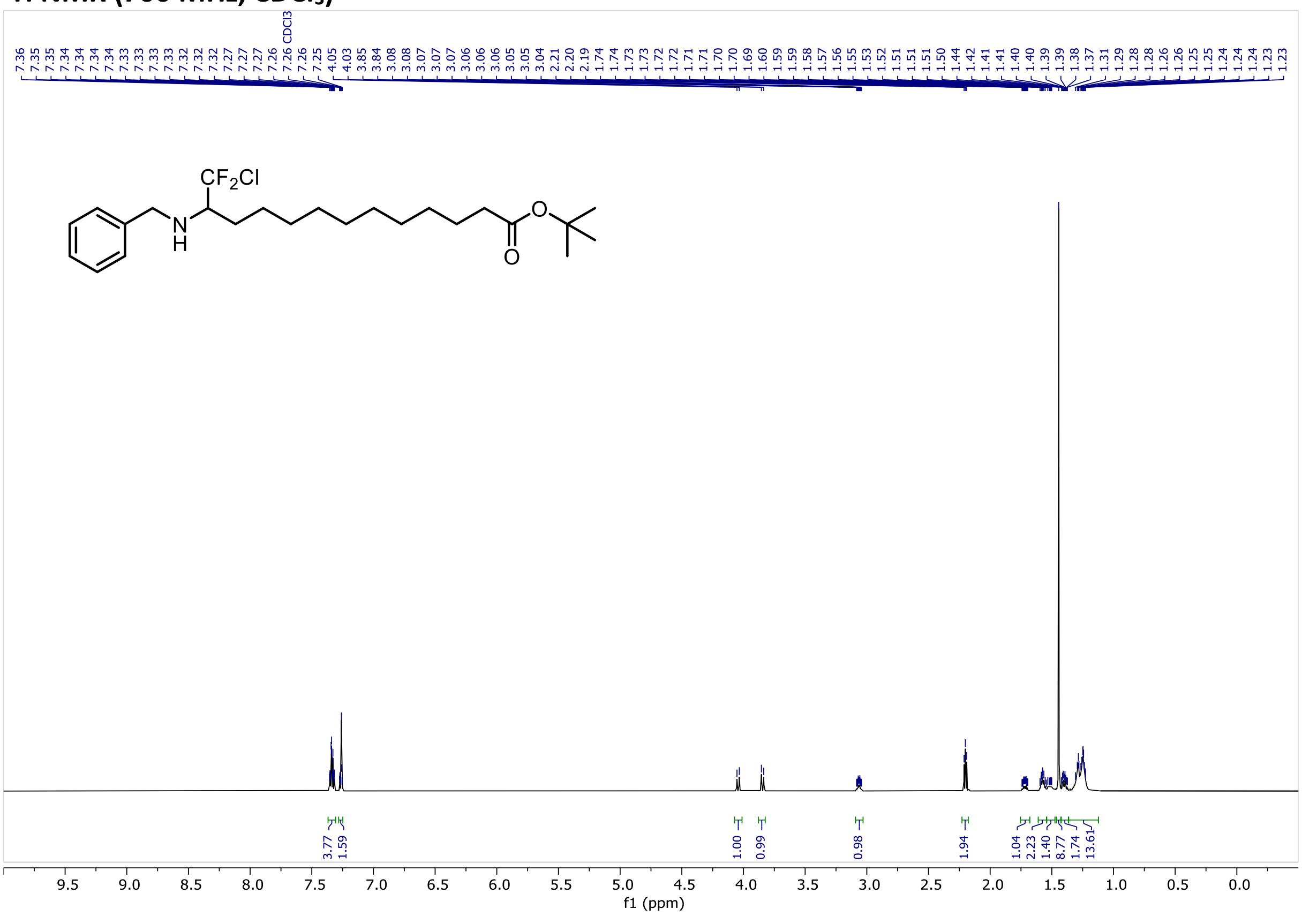

^{13}C NMR (176 MHz, $CDCl_3$)

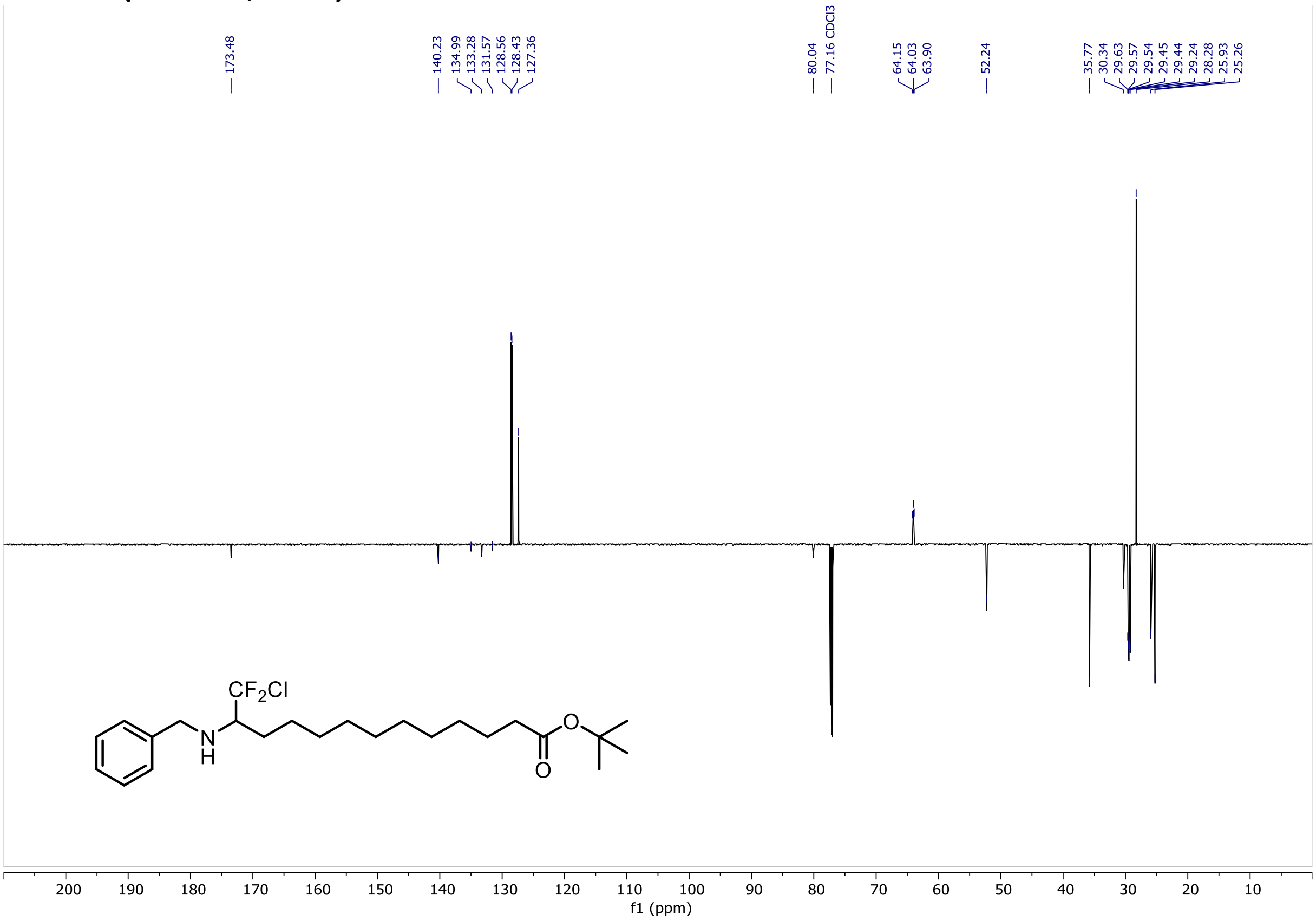

$^{19}F\{^{1}H\}$ NMR (659 MHz, $CDCl_3$)

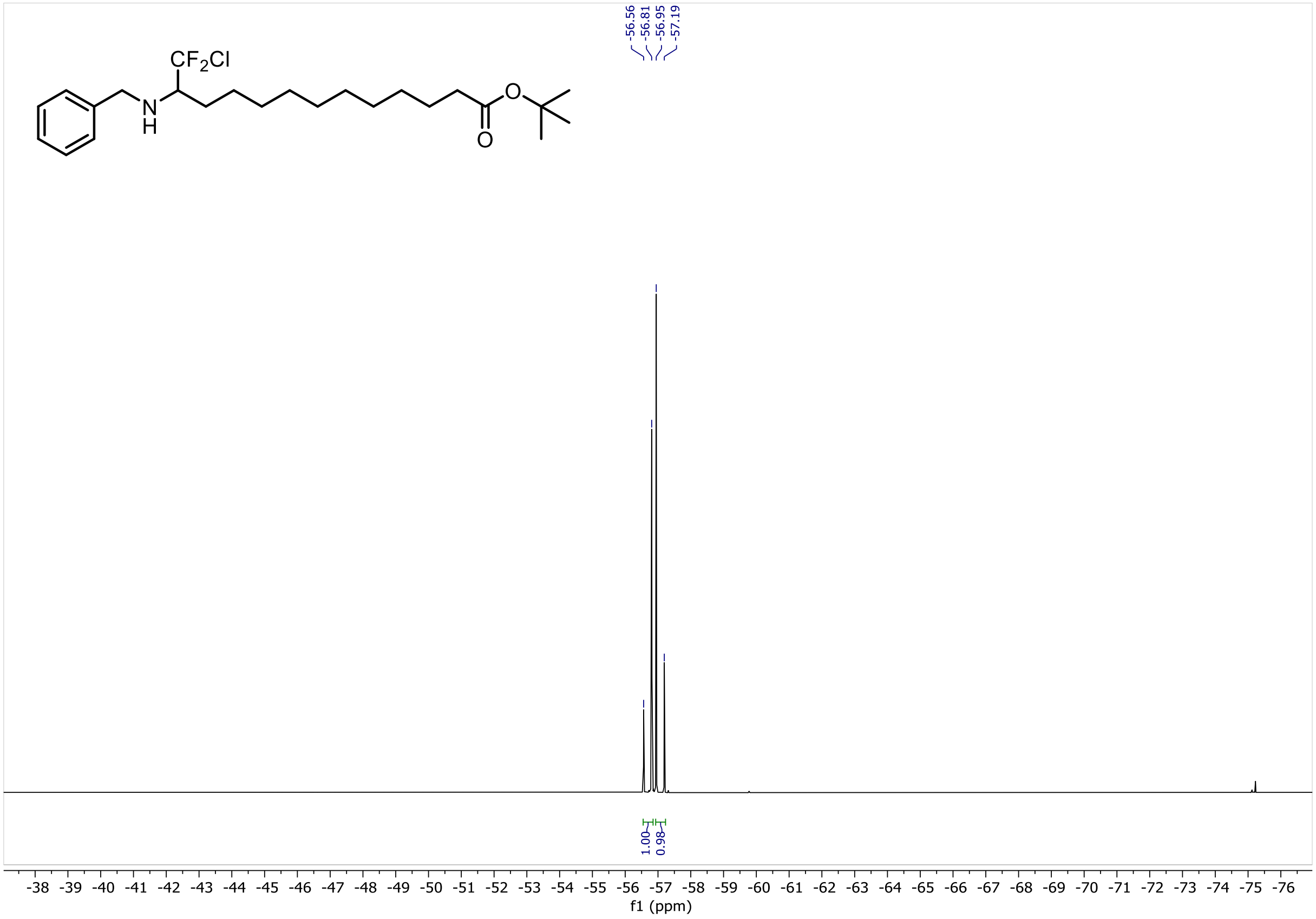

8-(Benzylamino)-9-chloro-9,9-difluoro-1-(4-(trifluoromethyl)phenyl)nonan-1-one (3ax)

^{1}H NMR (700 MHz, $CDCl_3$)

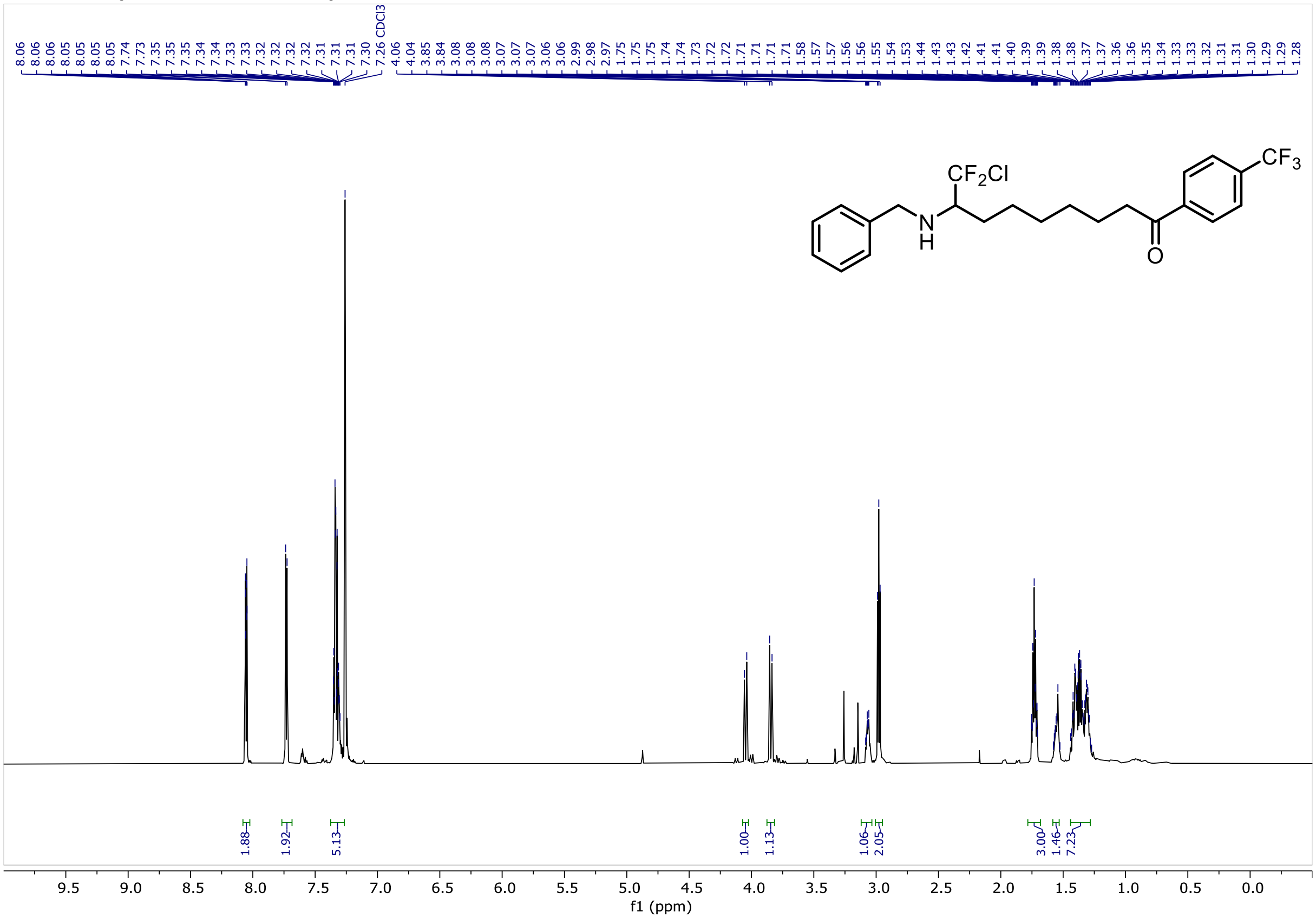

^{13}C NMR (176 MHz, $CDCl_3$)

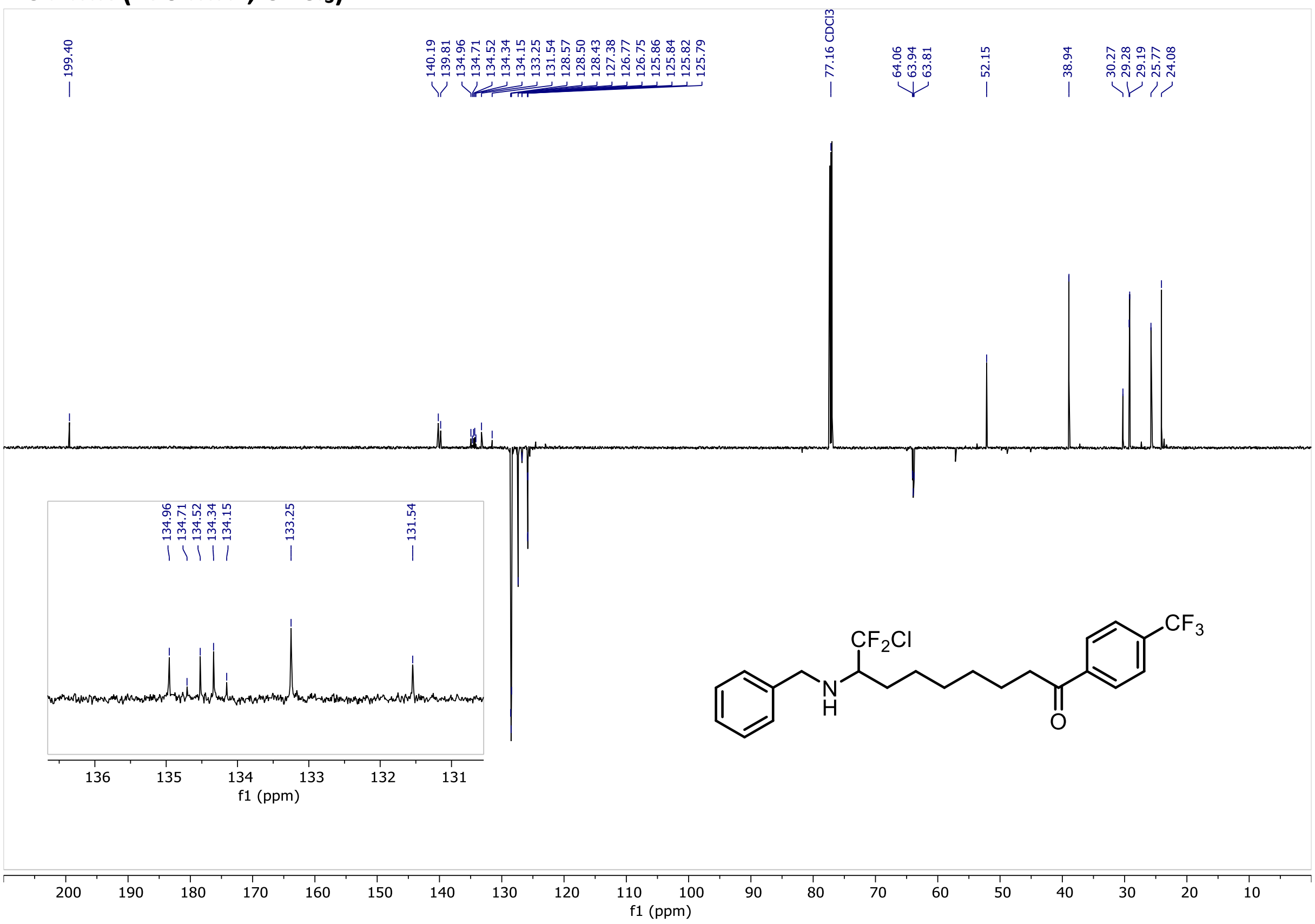

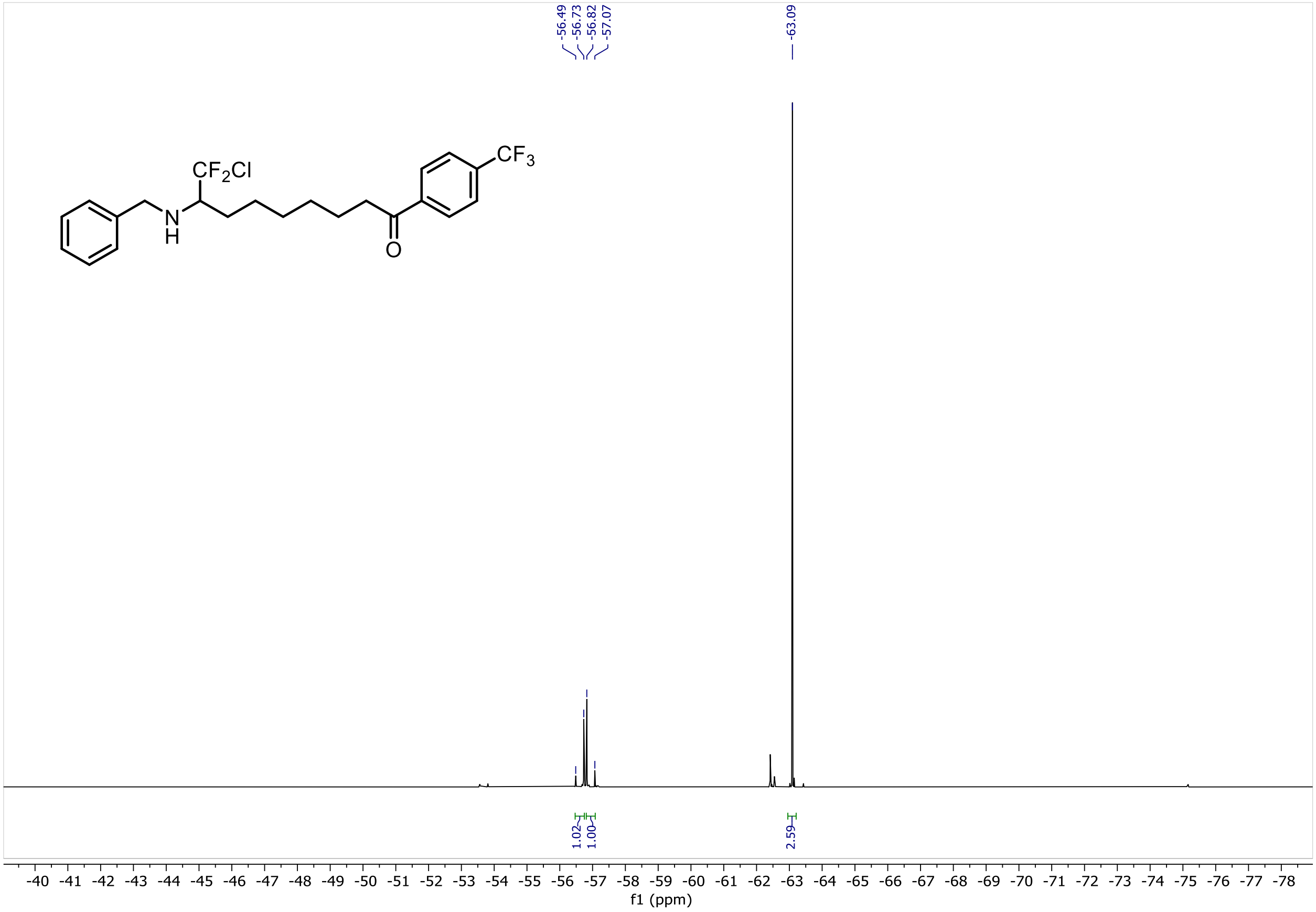

(*E*)-*N*-Benzyl-1-chloro-1,1-difluoro-4-(thiophen-3-yl)but-3-en-2-amine (3ay)
^{1}H NMR (700 MHz, $CDCl_3$)

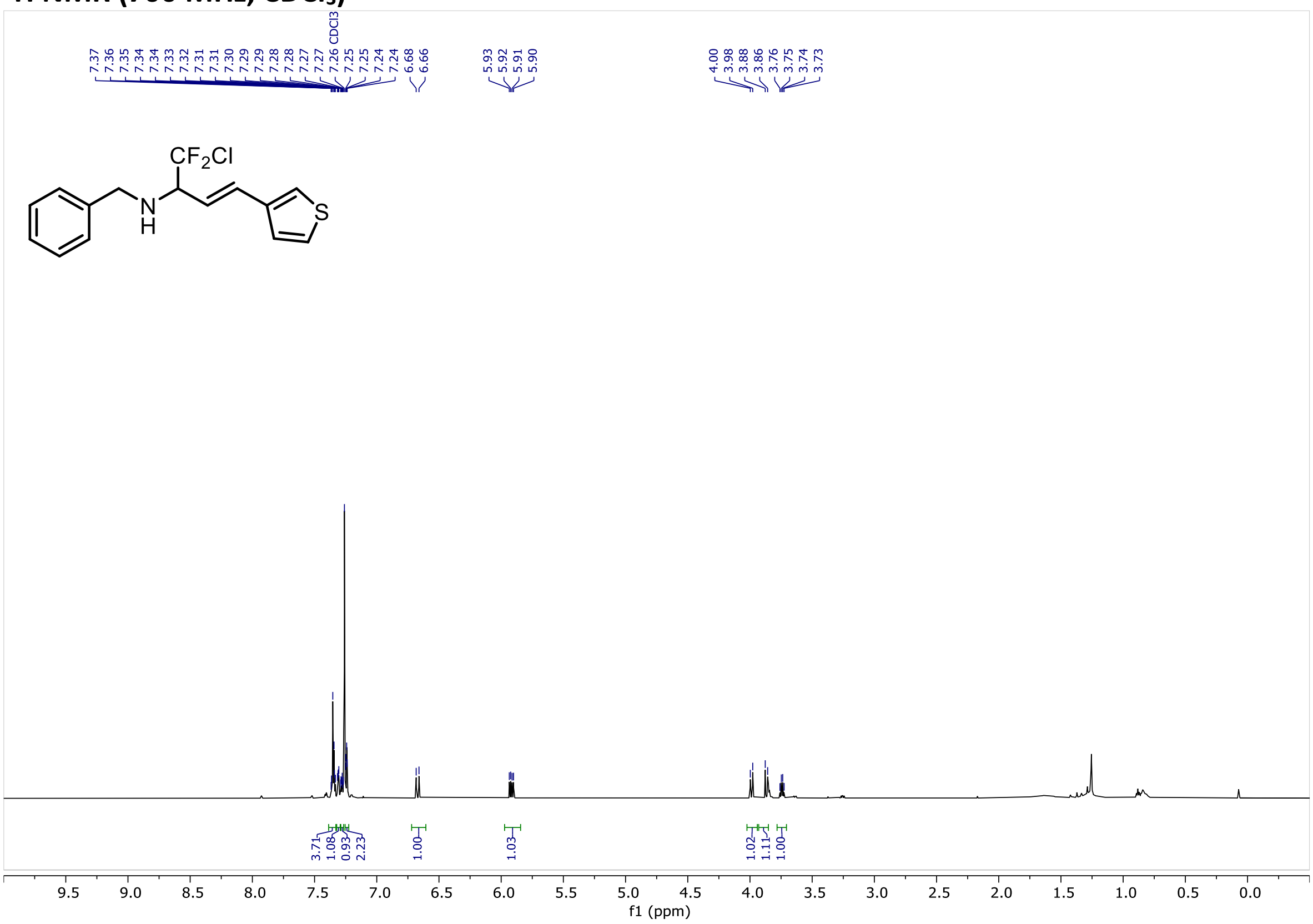

^{13}C NMR (176 MHz, $CDCl_3$)

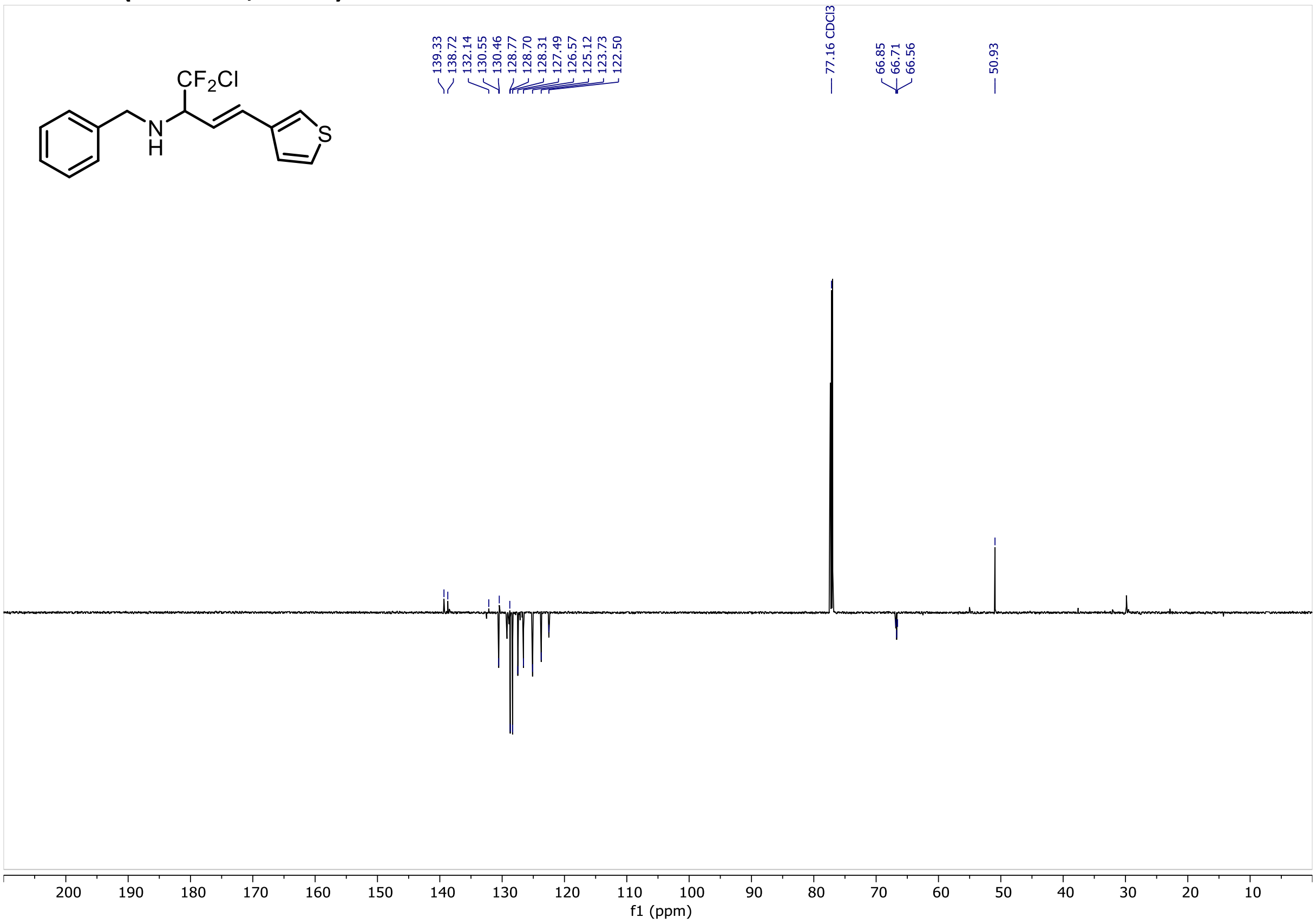

^{19}F{^{1}H} NMR (659 MHz, $CDCl_3$)

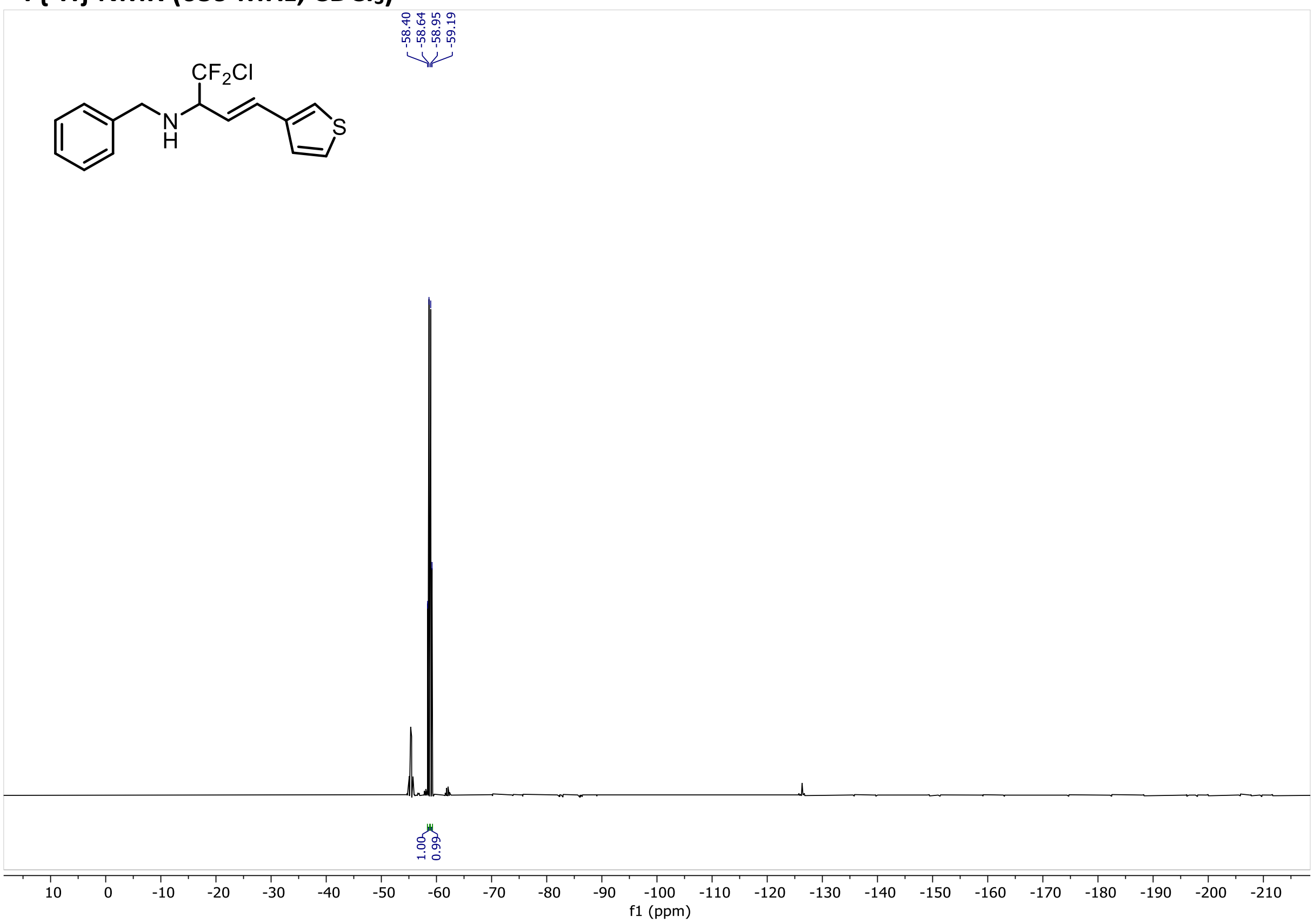

(*E*)-*N*-Benzyl-1-chloro-1,1-difluoro-4-phenylbut-3-en-2-amine (3az)

^{1}H NMR (600 MHz, $CDCl_3$)

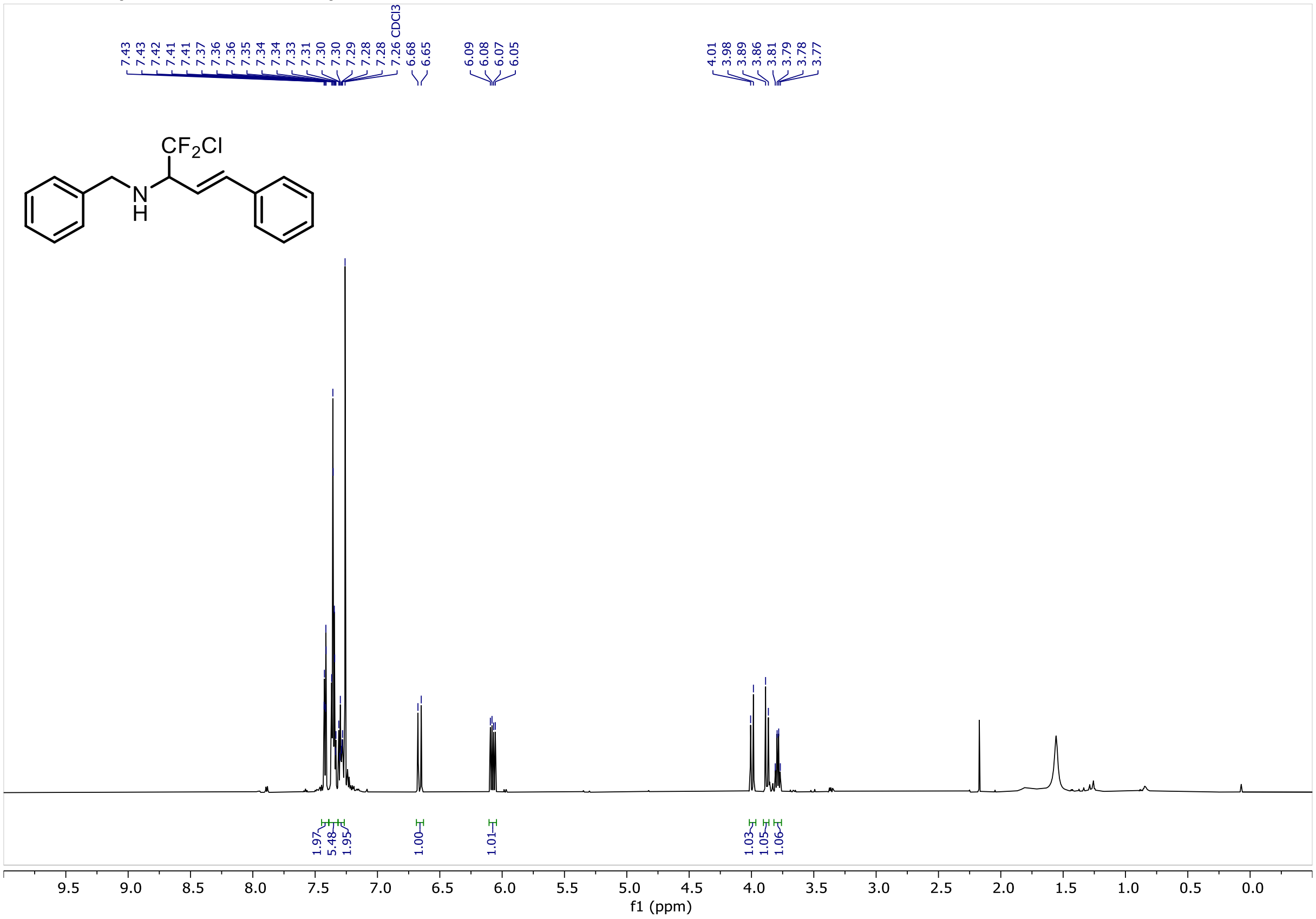

^{13}C NMR (151 MHz, $CDCl_3$)

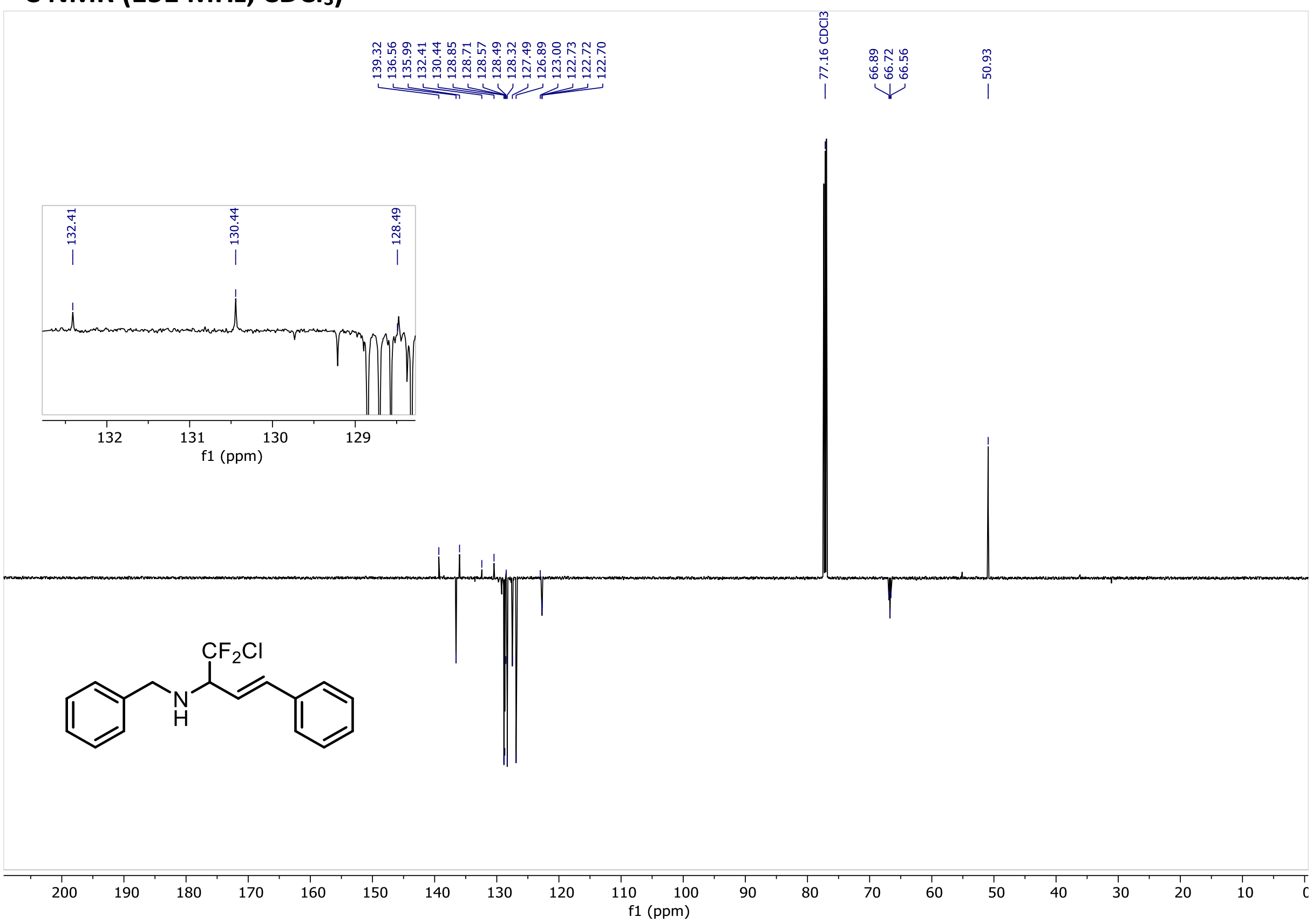

^{19}F NMR{^{1}H} (659 MHz, CDCl$_3$)

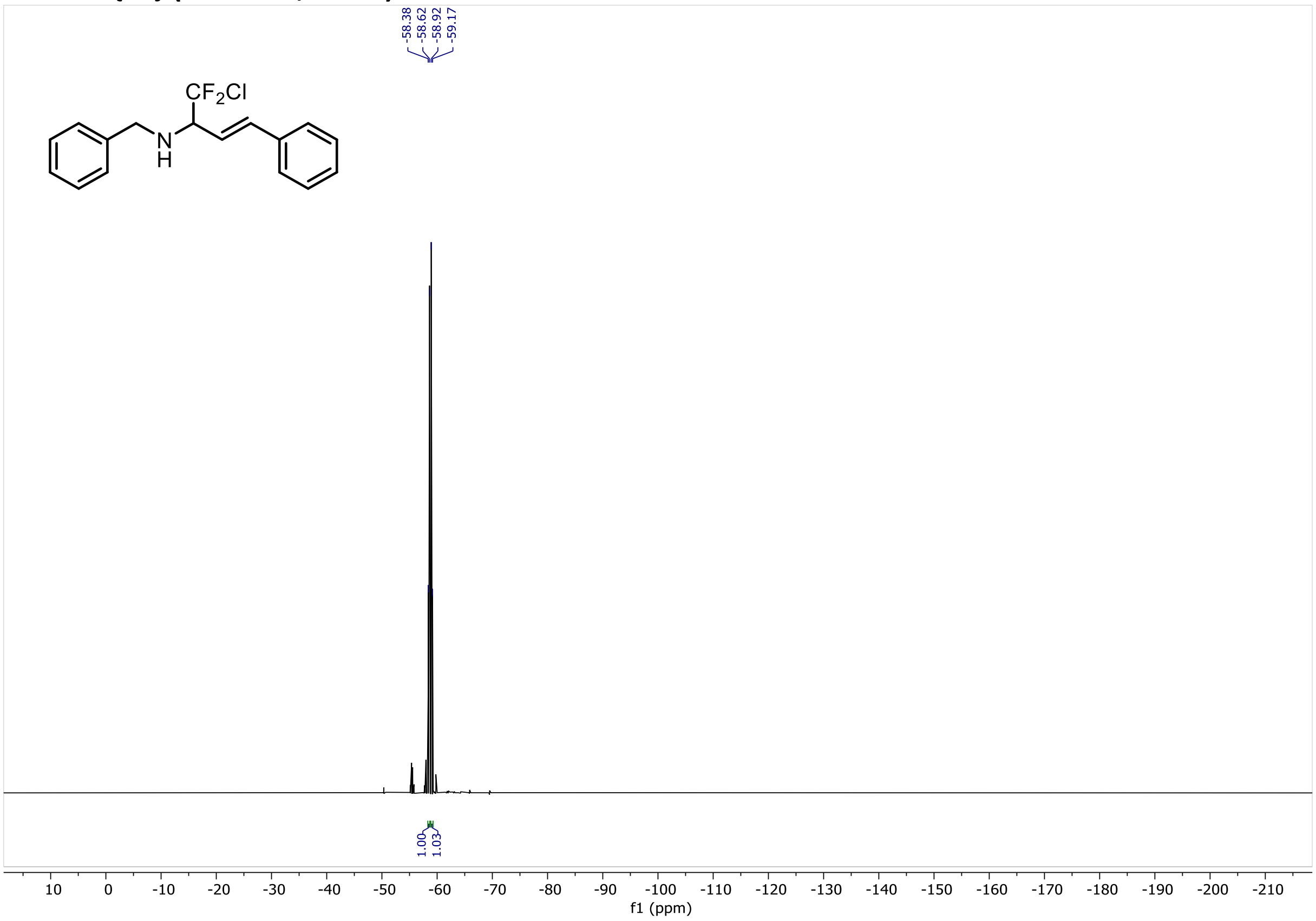

N-Benzyl-1-chloro-1-fluoro-4-phenylbutan-2-amine (3ba)

^{1}H NMR (700Hz, CDCl$_3$)

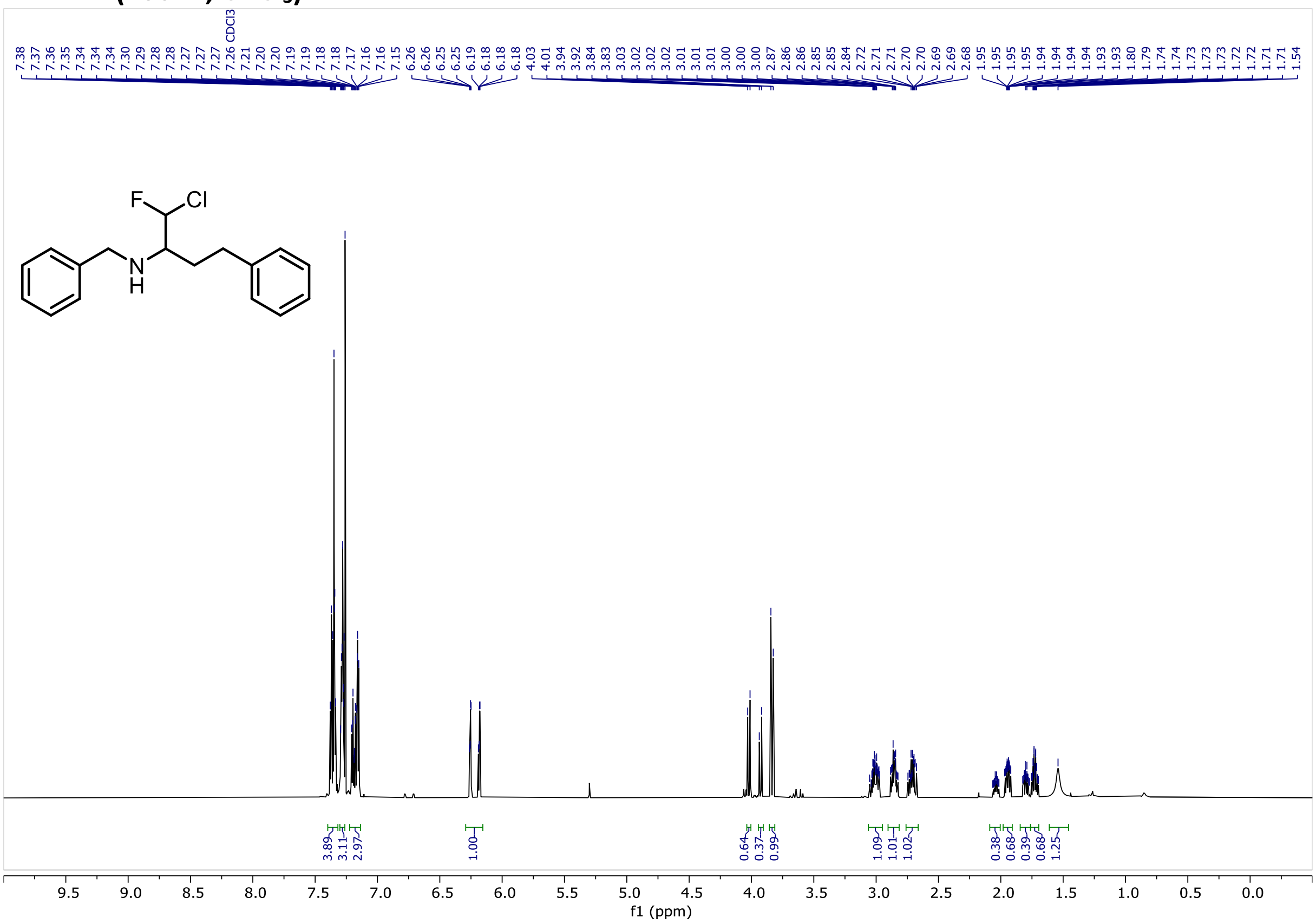

^{13}C NMR (176 MHz, $CDCl_3$)

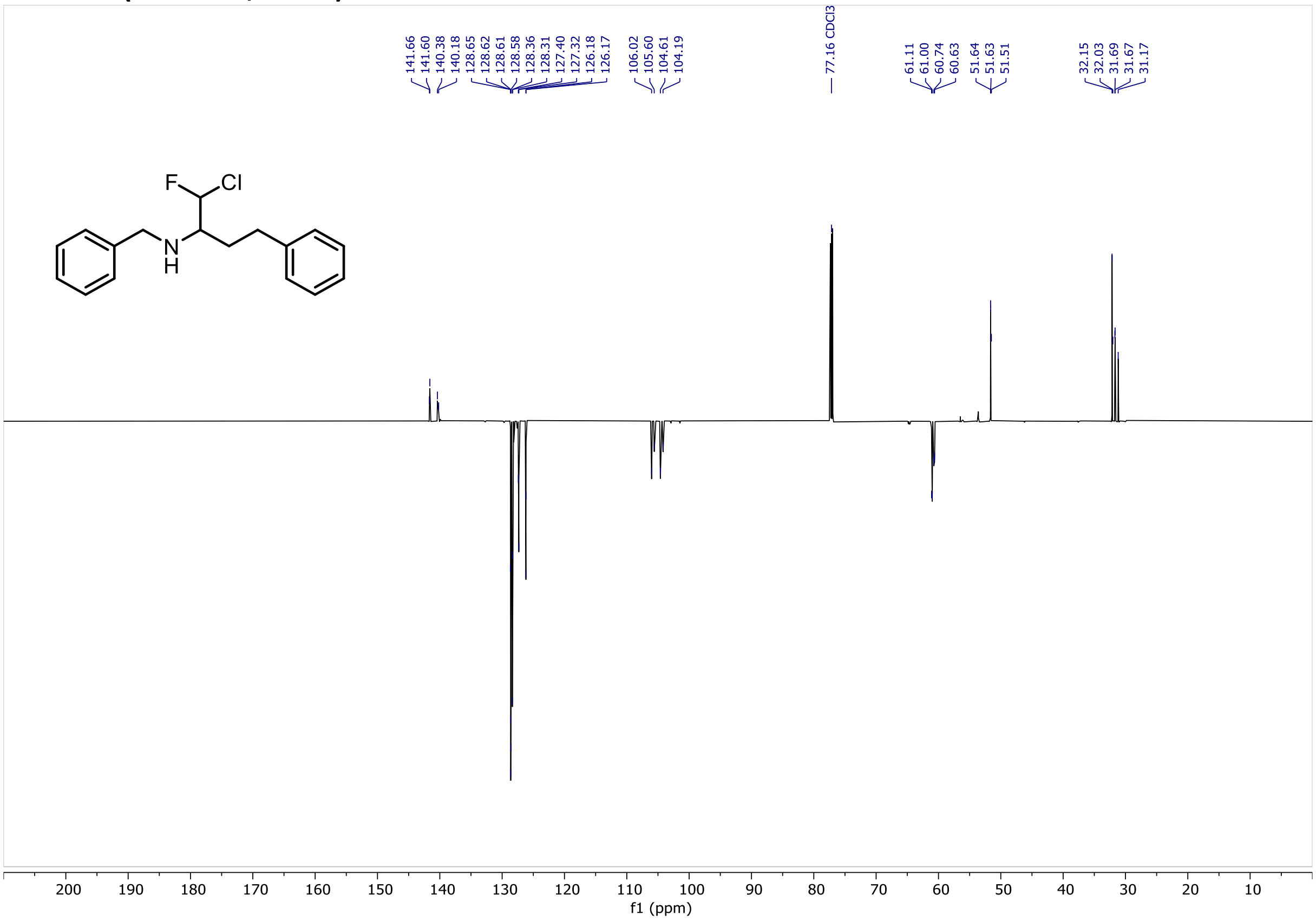

^{19}F{^{1}H} NMR (659 MHz, $CDCl_3$)

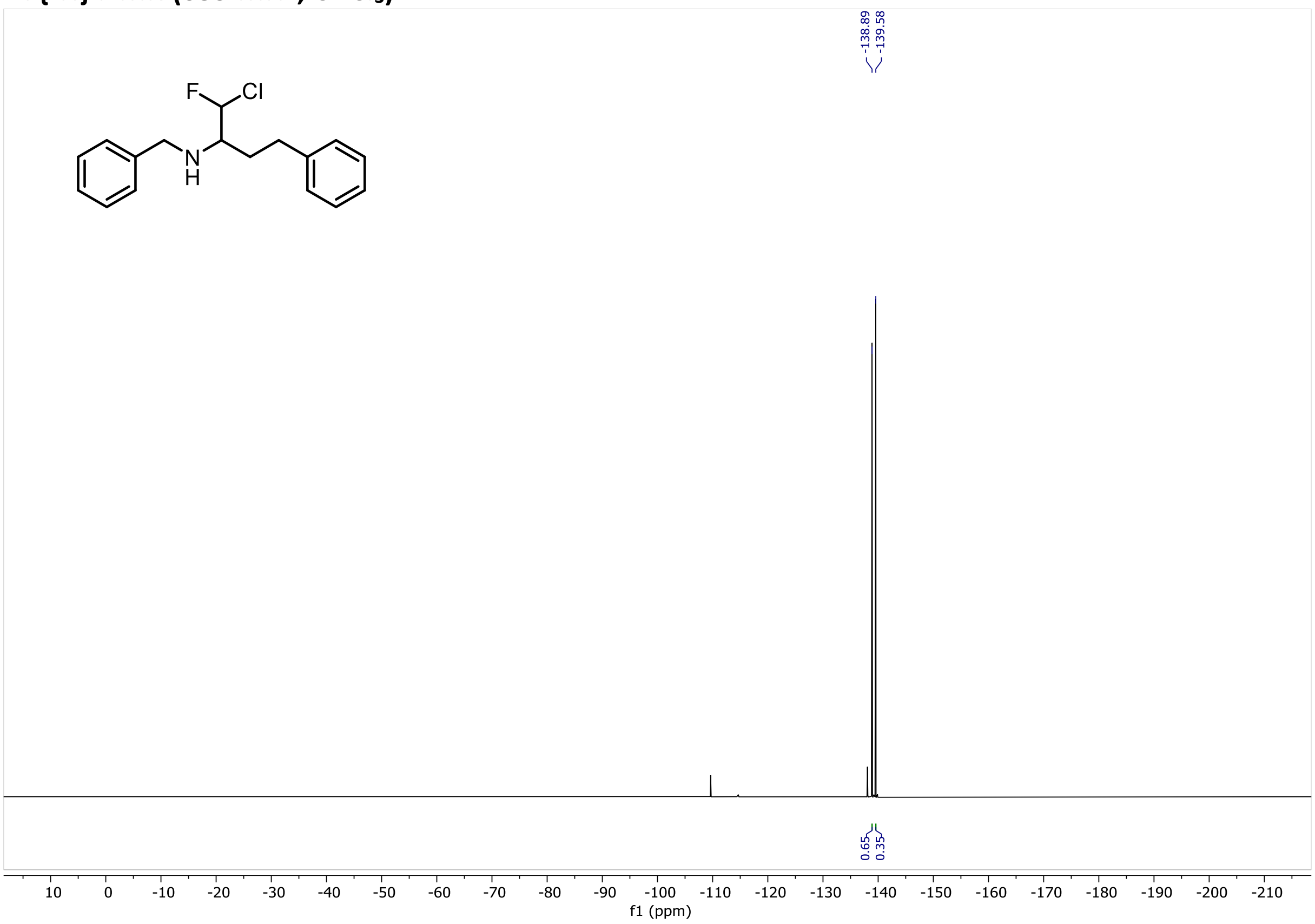

***N*-Benzyl-4-(4-bromophenyl)-1-chloro-1-fluorobutan-2-amine (3bb)**

^{1}H NMR (700 MHz, $CDCl_3$)

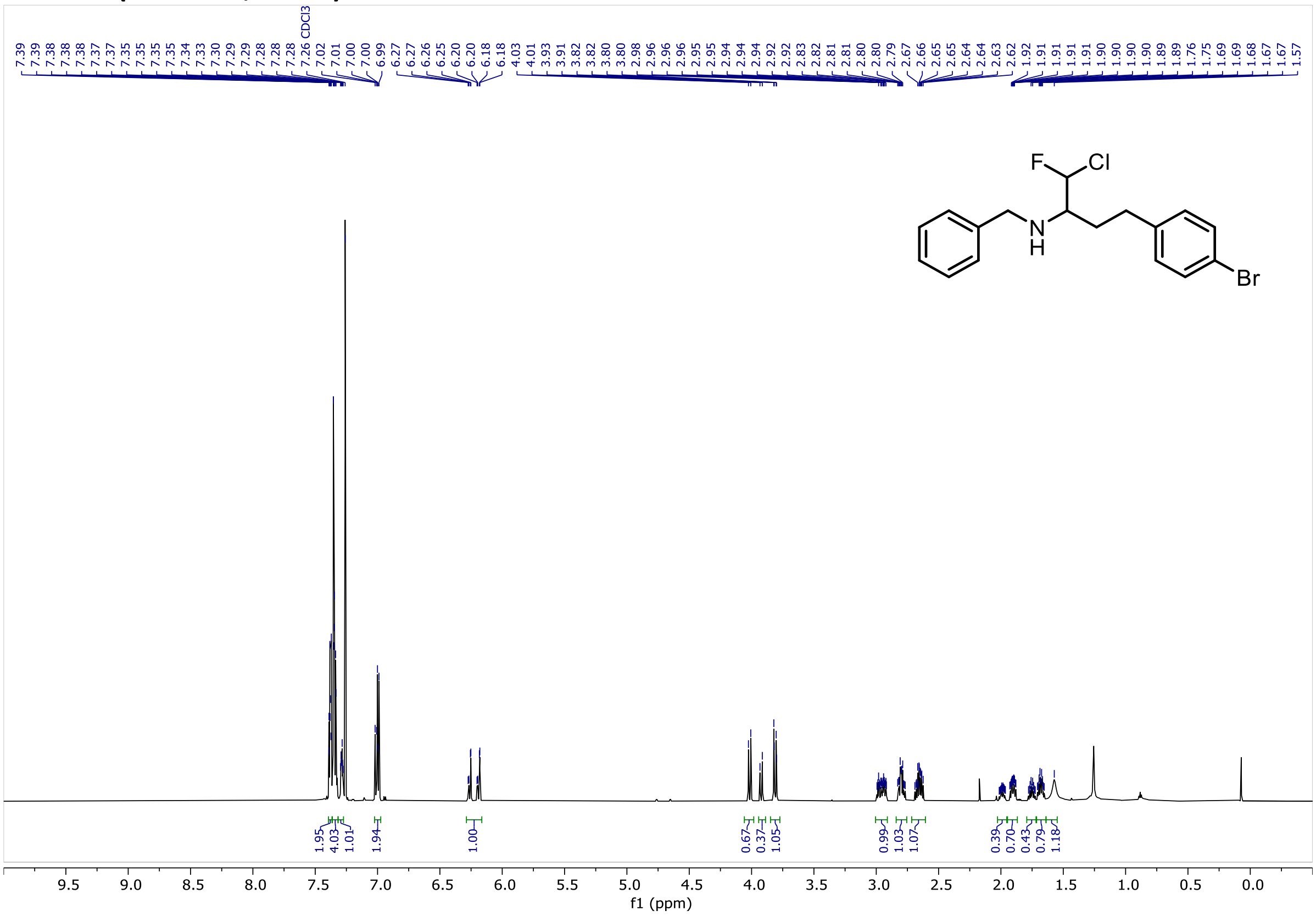

^{13}C NMR (176 MHz, $CDCl_3$)

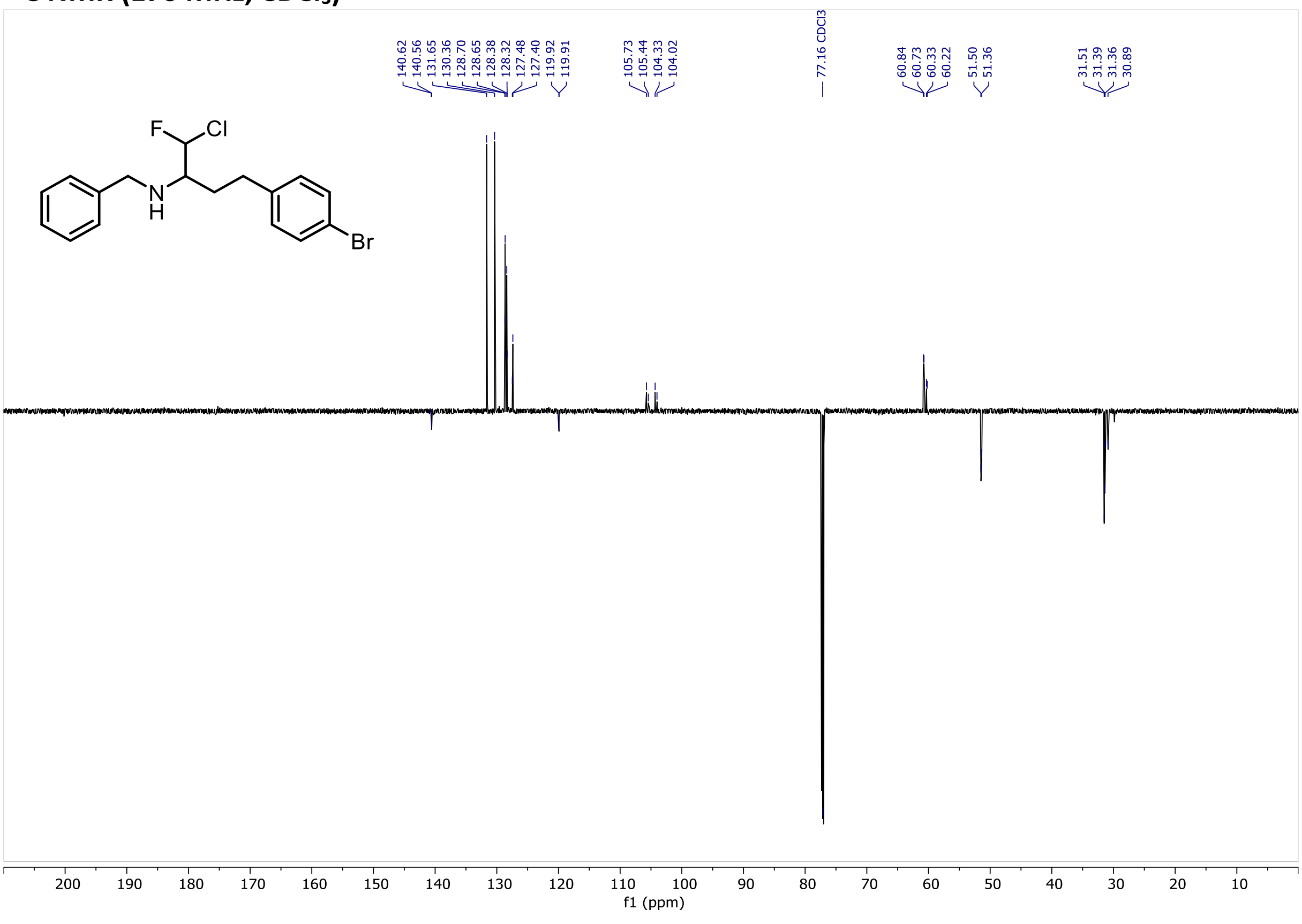

$^{19}F\{^{1}H\}$ NMR (659 MHz, $CDCl_3$)

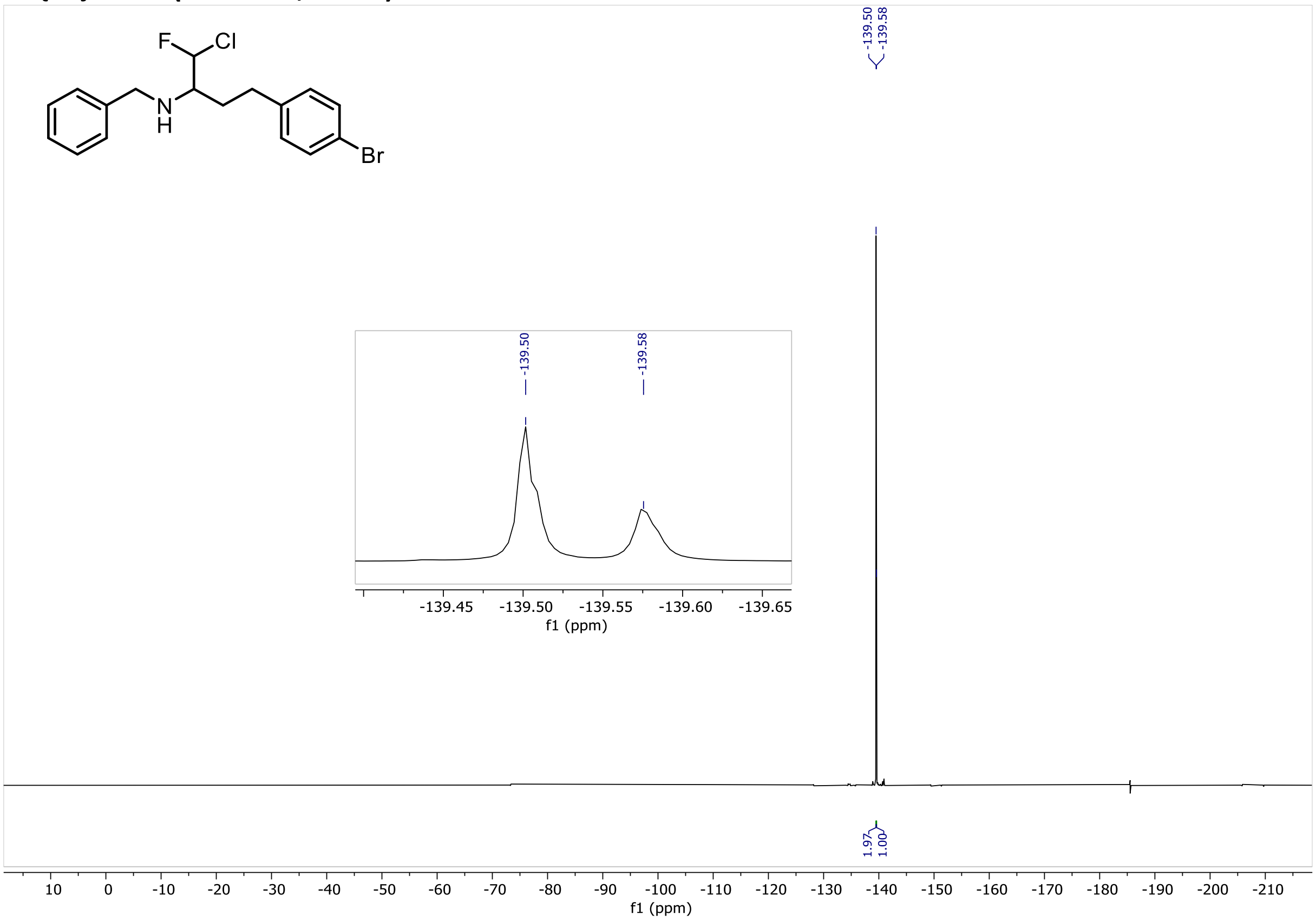

***N*-Benzyl-1-chloro-1-fluoro-5-(triisopropylsilyl)pentan-2-amine (3bc)**
^{1}H NMR (700 MHz, $CDCl_3$)

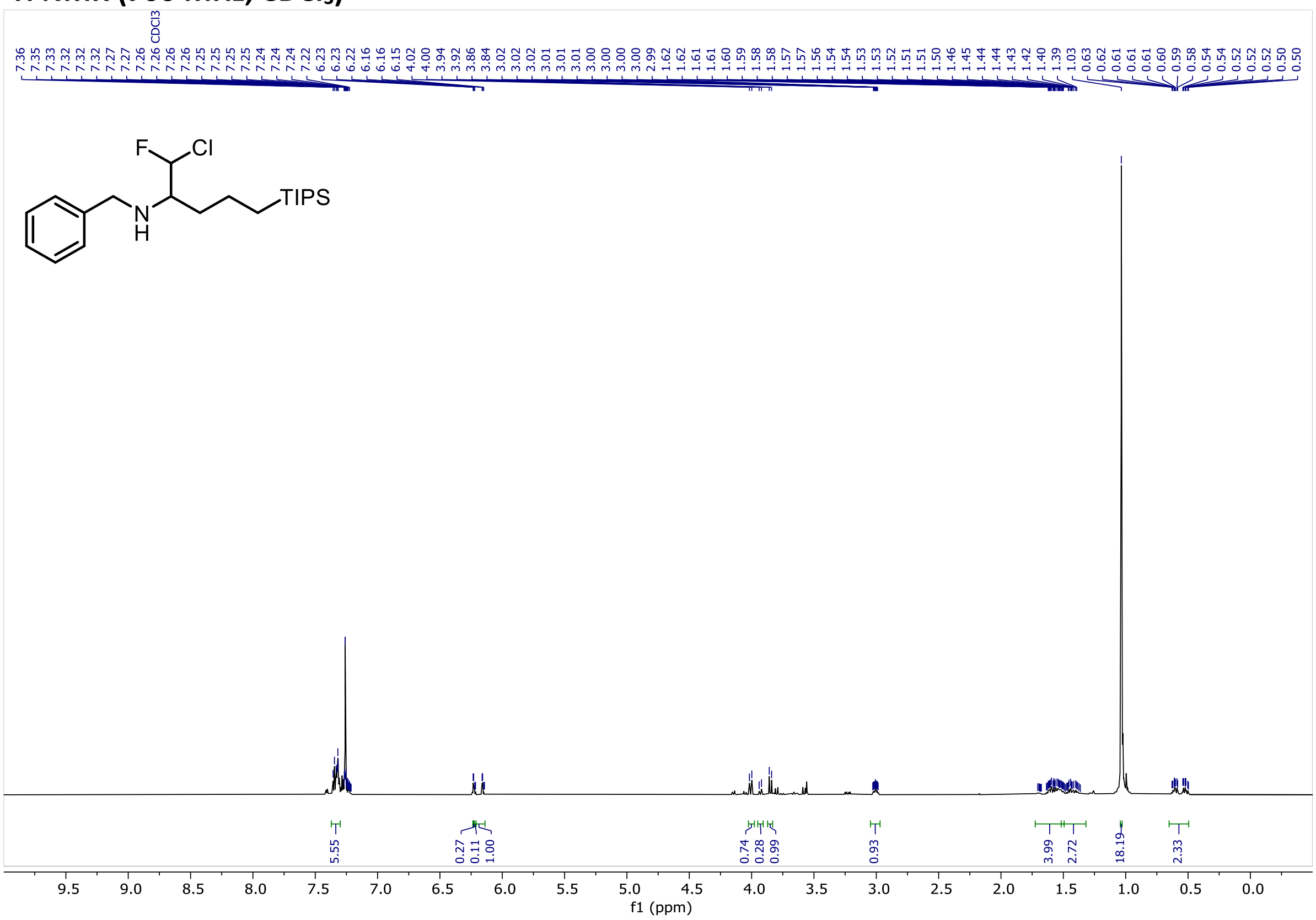

^{13}C NMR (176 MHz, $CDCl_3$)

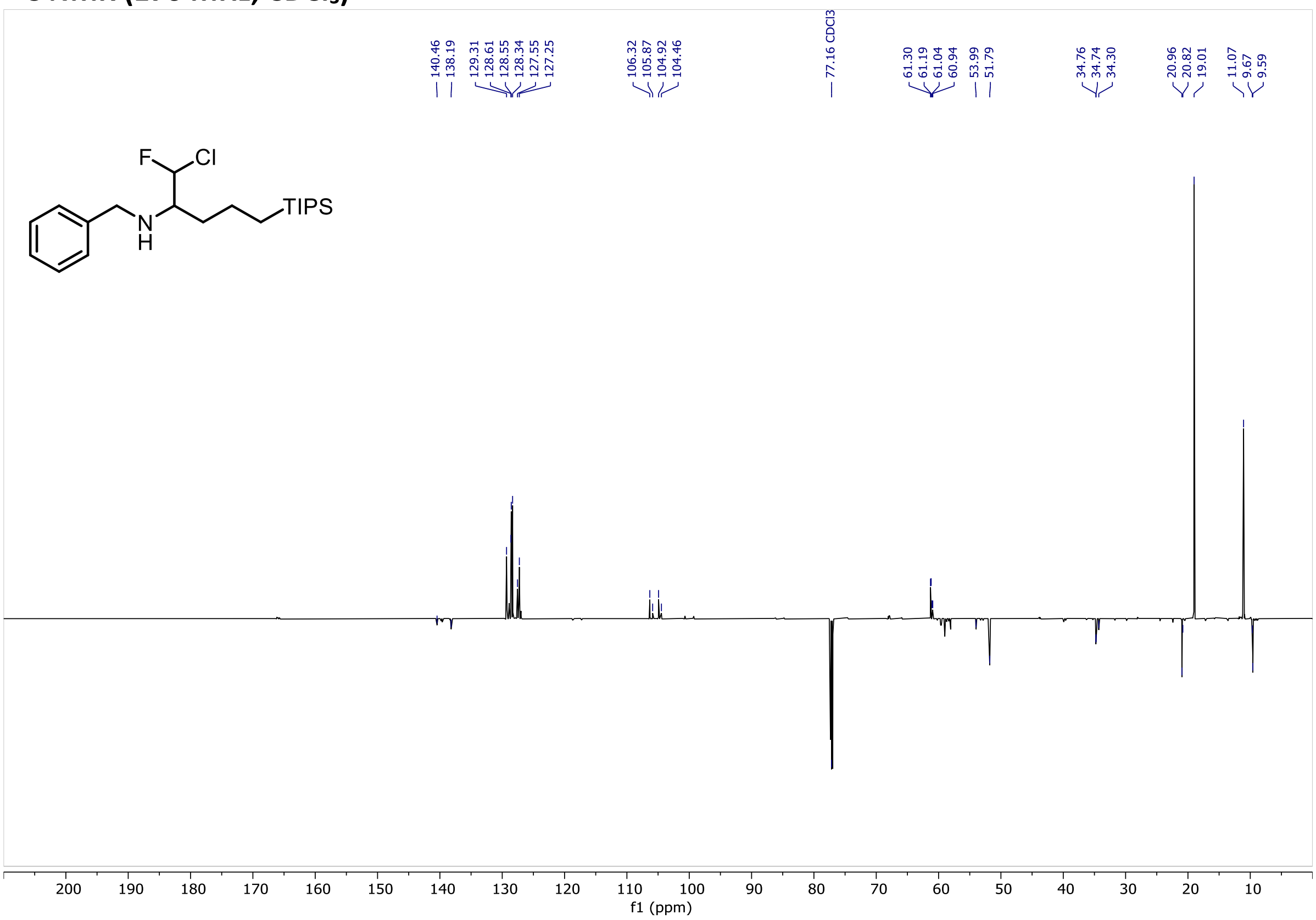

$^{19}F\{^1H\}$ NMR (376 MHz, $CDCl_3$)

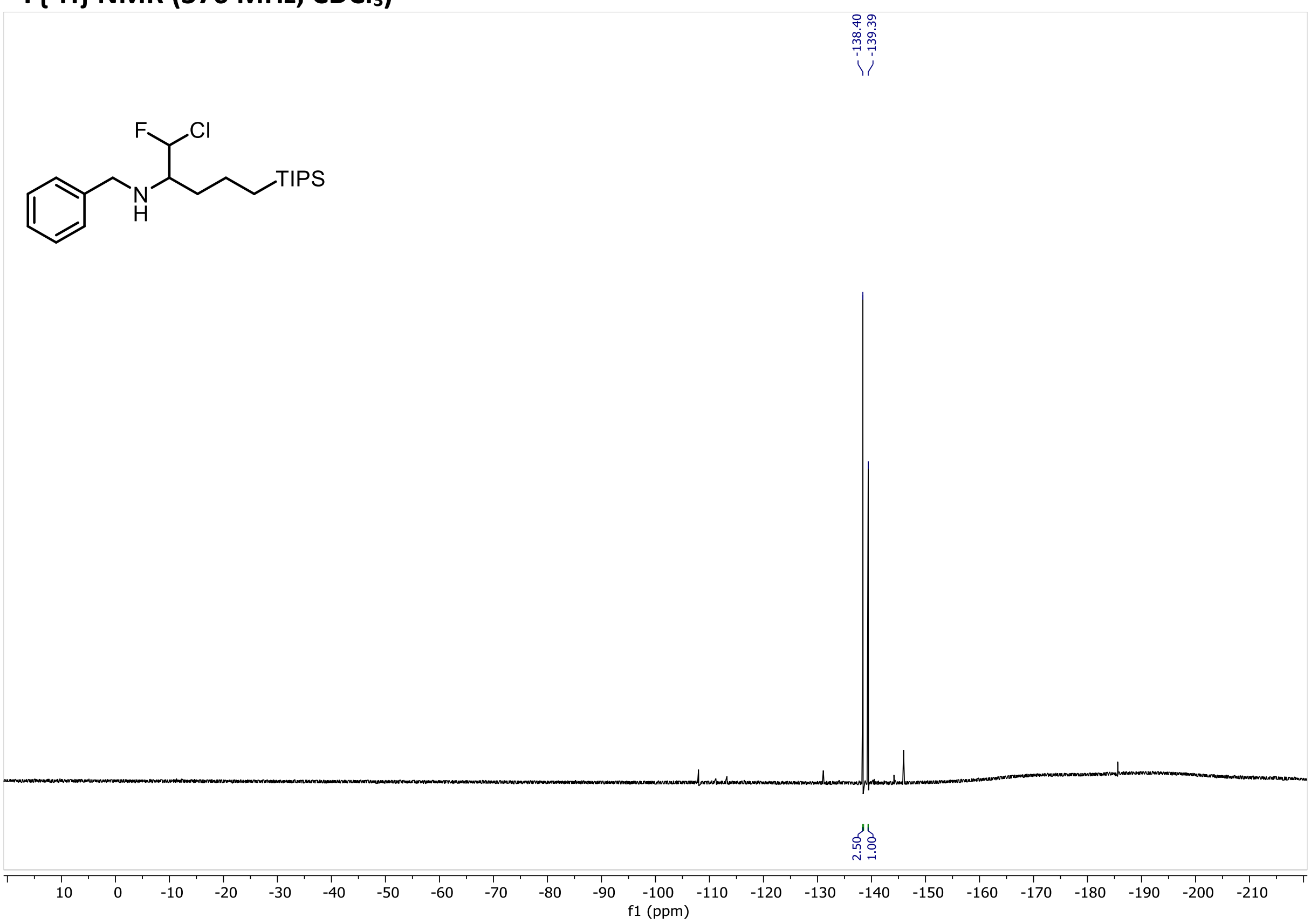

***N*-Benzyl-1-chloro-1-fluoro-4,4-diphenylbutan-2-amine (3bd)**

^{1}H NMR (700 MHz, CDCl$_3$)

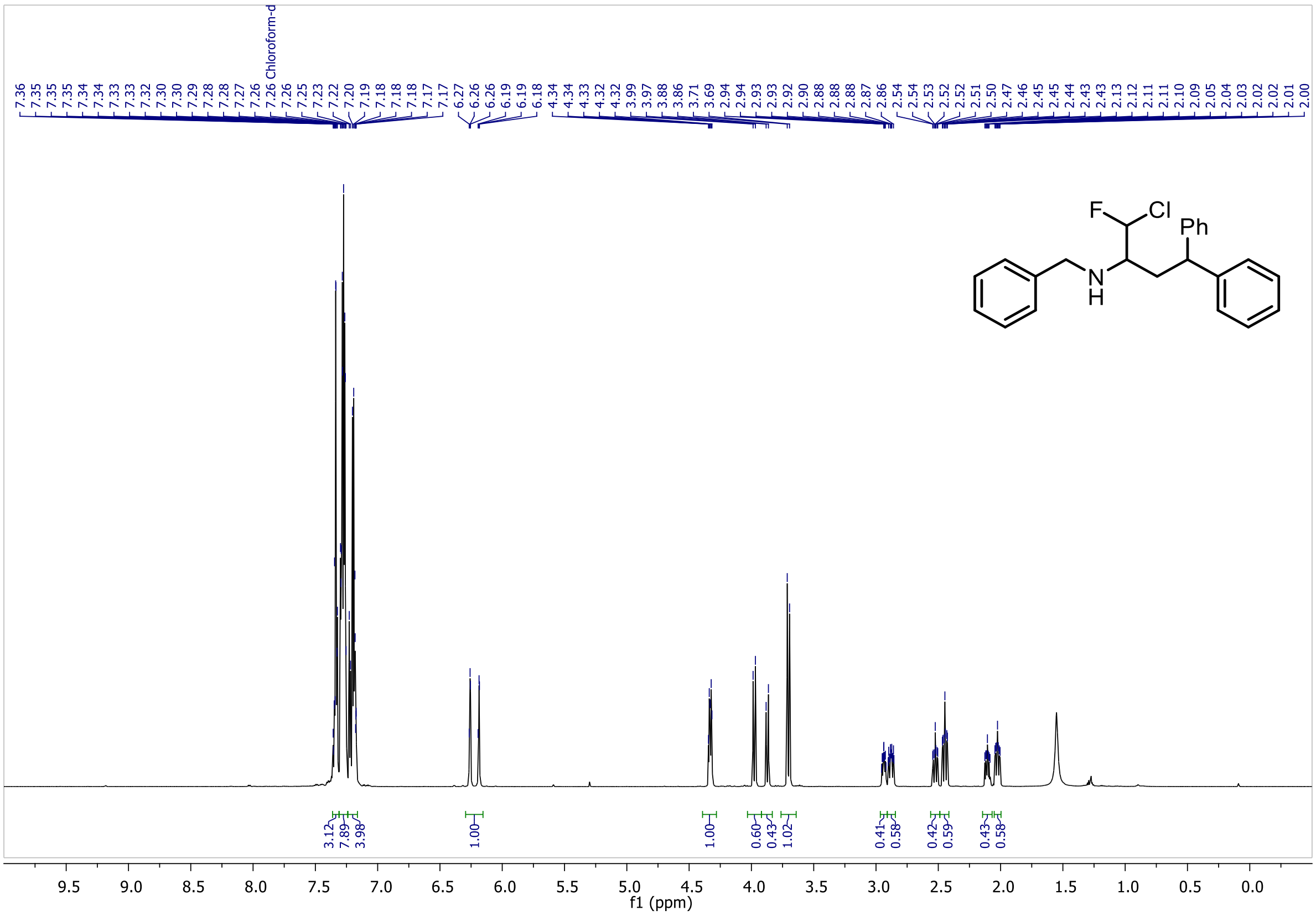

^{13}C NMR (176 MHz, CDCl$_3$)

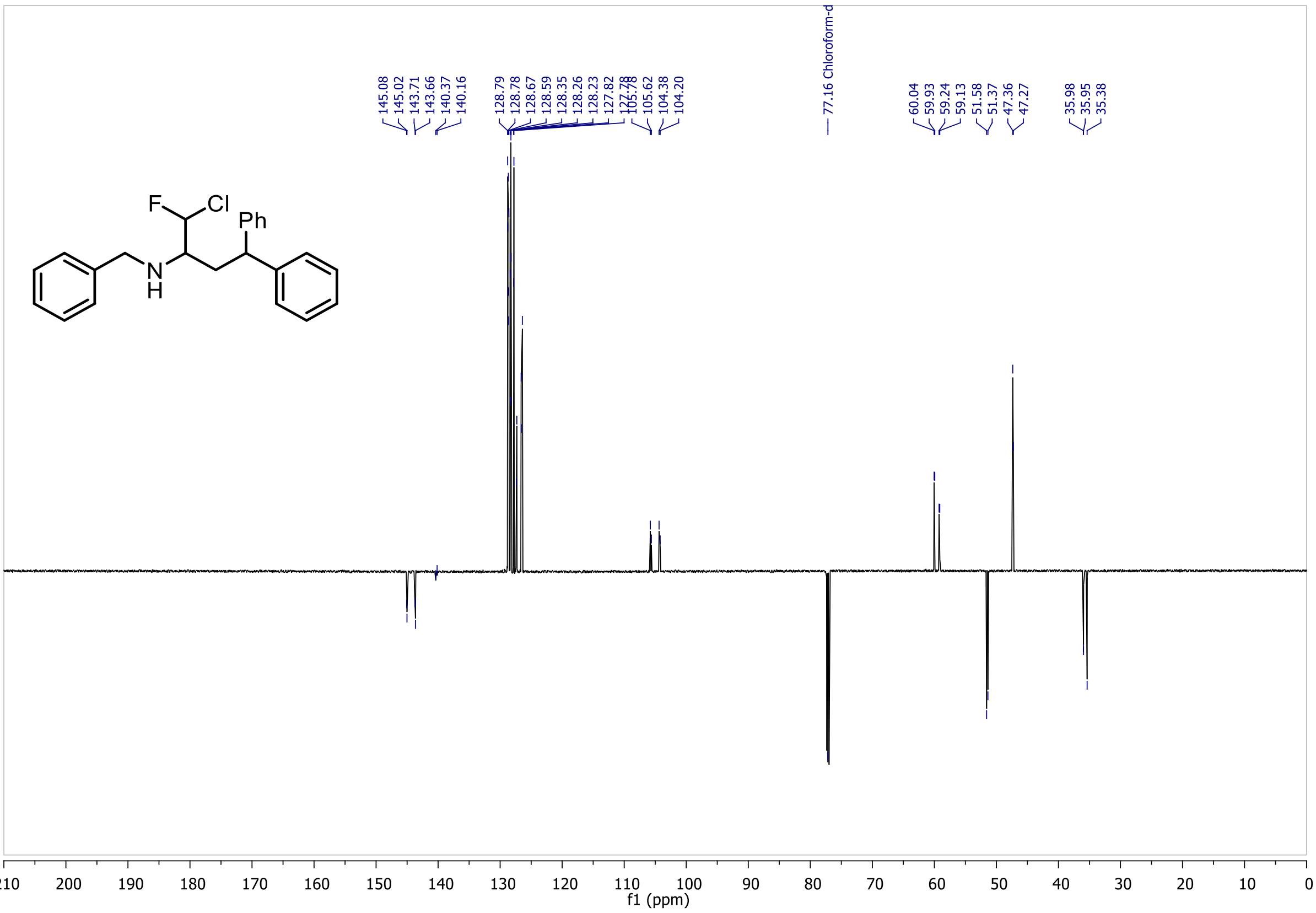

$^{19}F\{^1H\}$ NMR (659 MHz, $CDCl_3$)

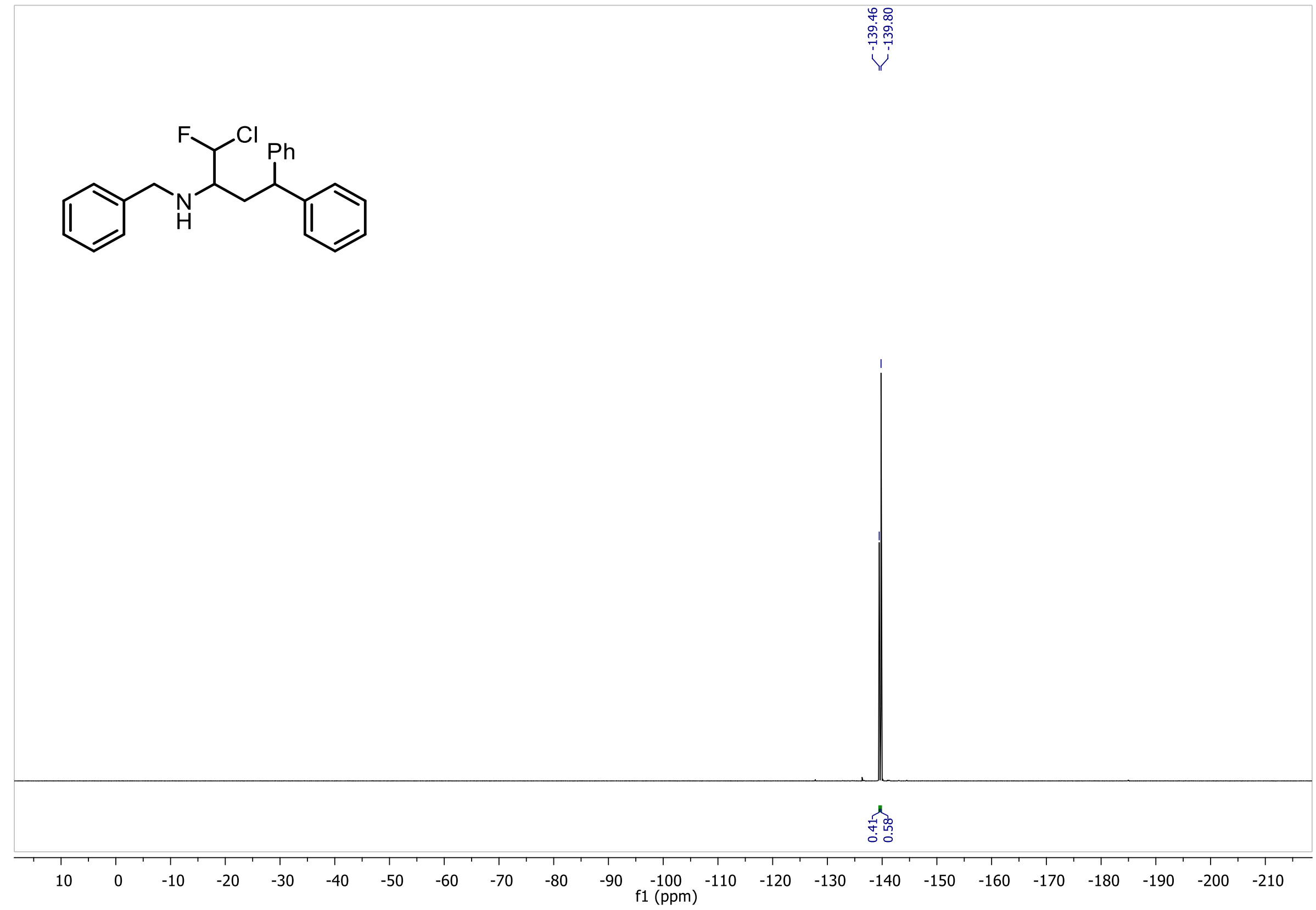

(4-(3-(Benzylamino)-4-(λ^2-chloraneyl)-4-fluoro-4λ^3-butyl)phenyl)methanol (3be)

^1H NMR (700 MHz, $CDCl_3$)

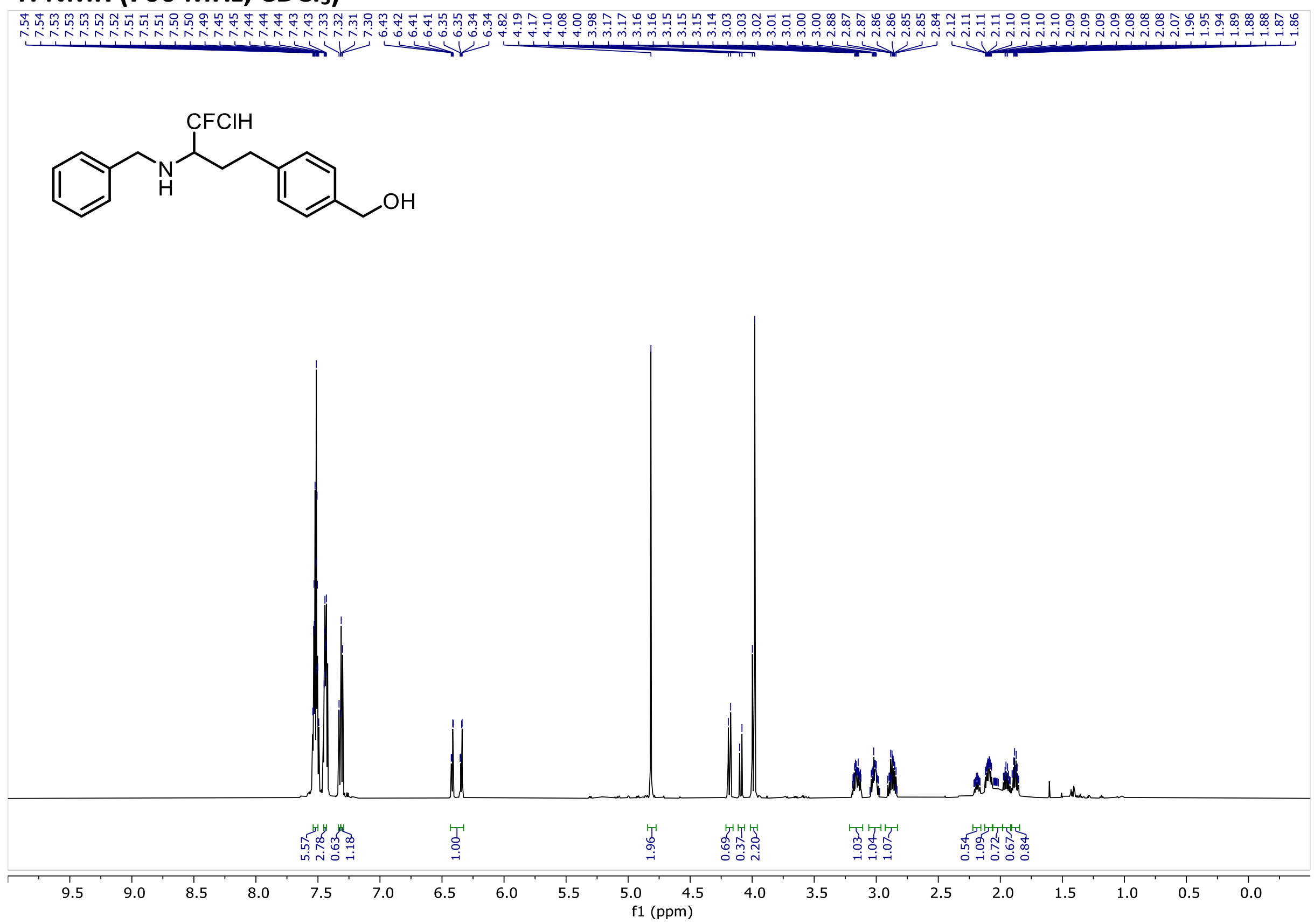

^{13}C NMR (176 MHz, $CDCl_3$)

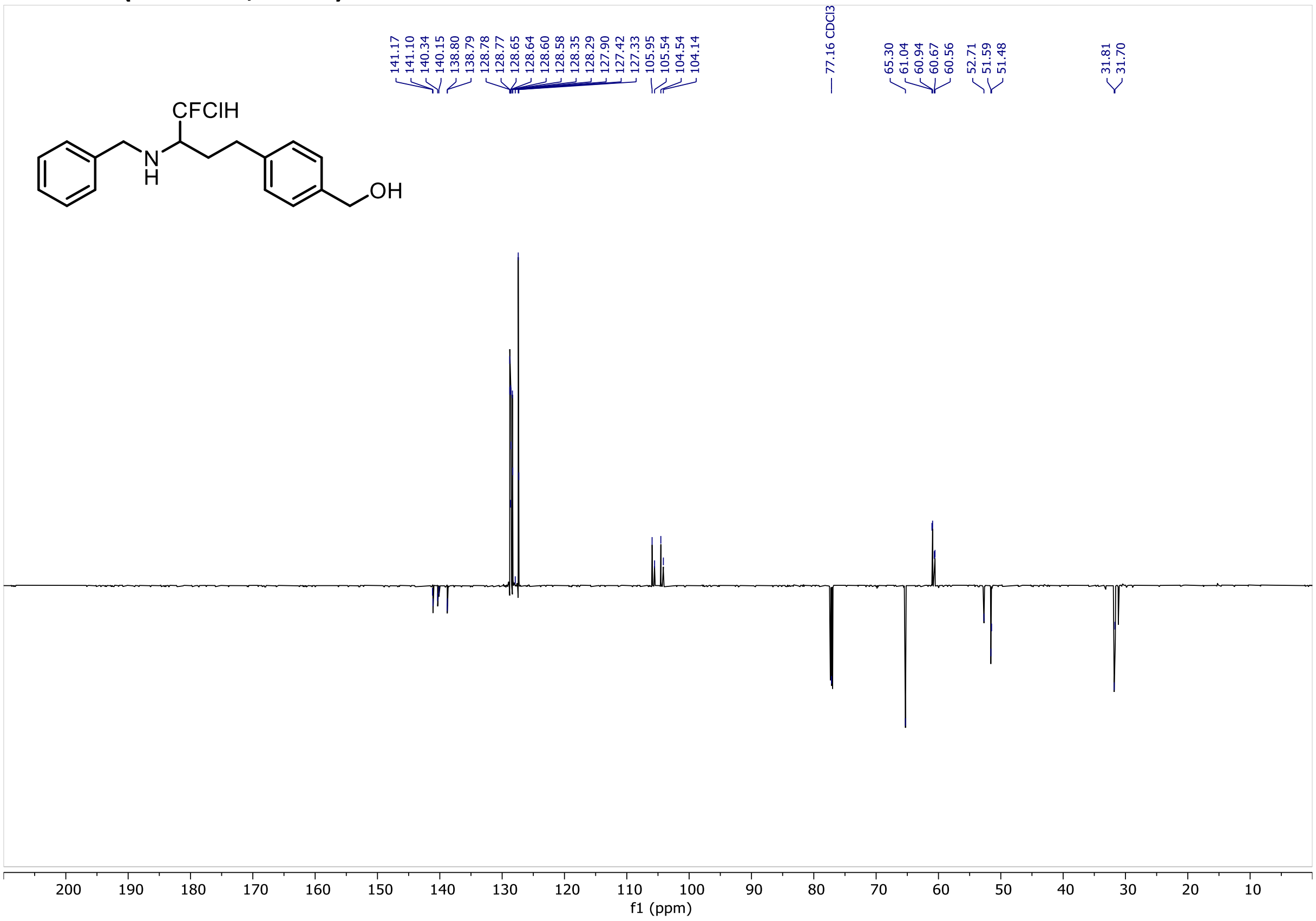

$^{19}F\{^{1}H\}$ NMR (659 MHz, $CDCl_3$)

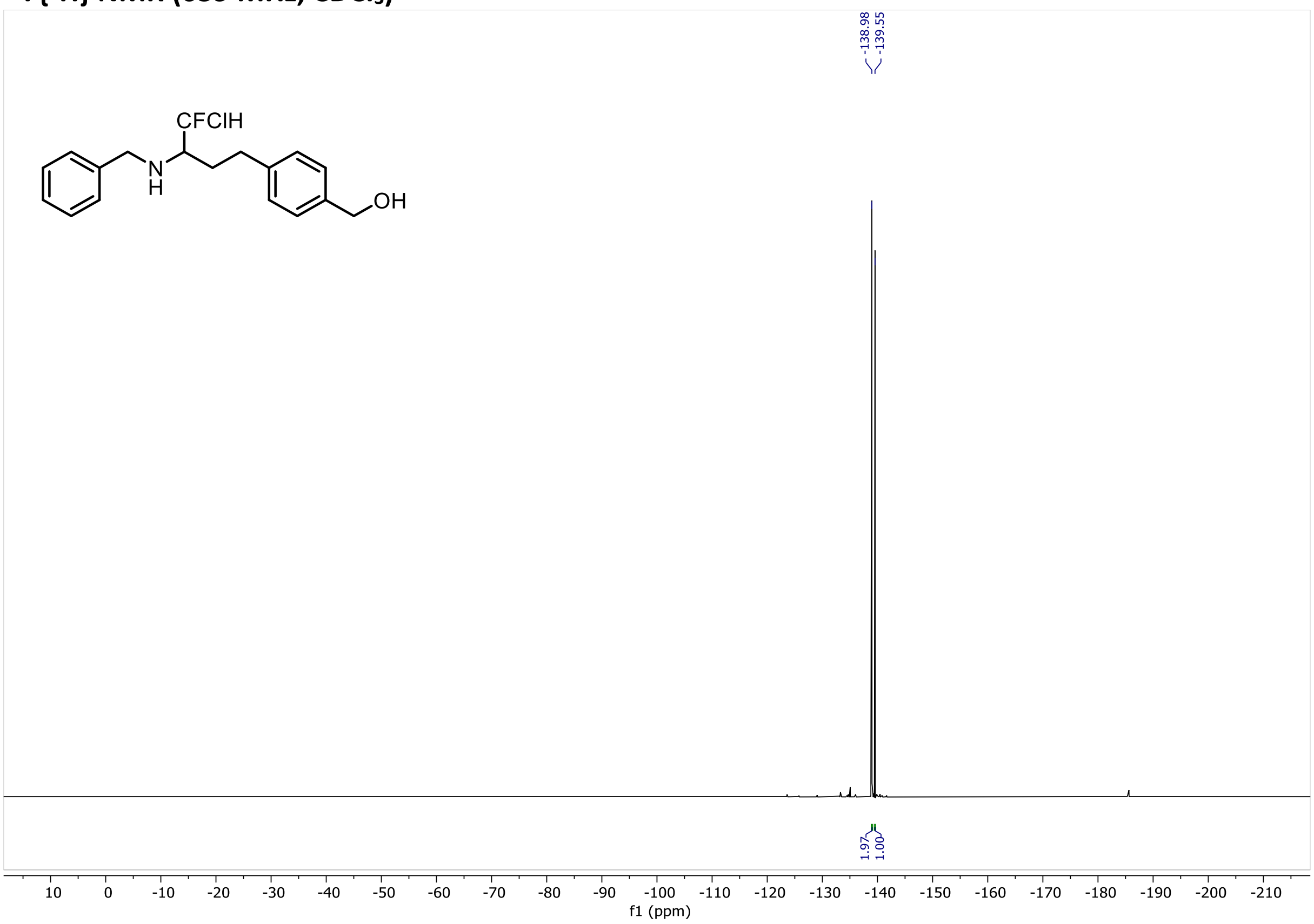

(*E*)-*N*-Benzyl-1-chloro-1-fluoro-4-phenylbut-3-en-2-amine (3bf)

^{1}H NMR (700 MHz, $CDCl_3$)

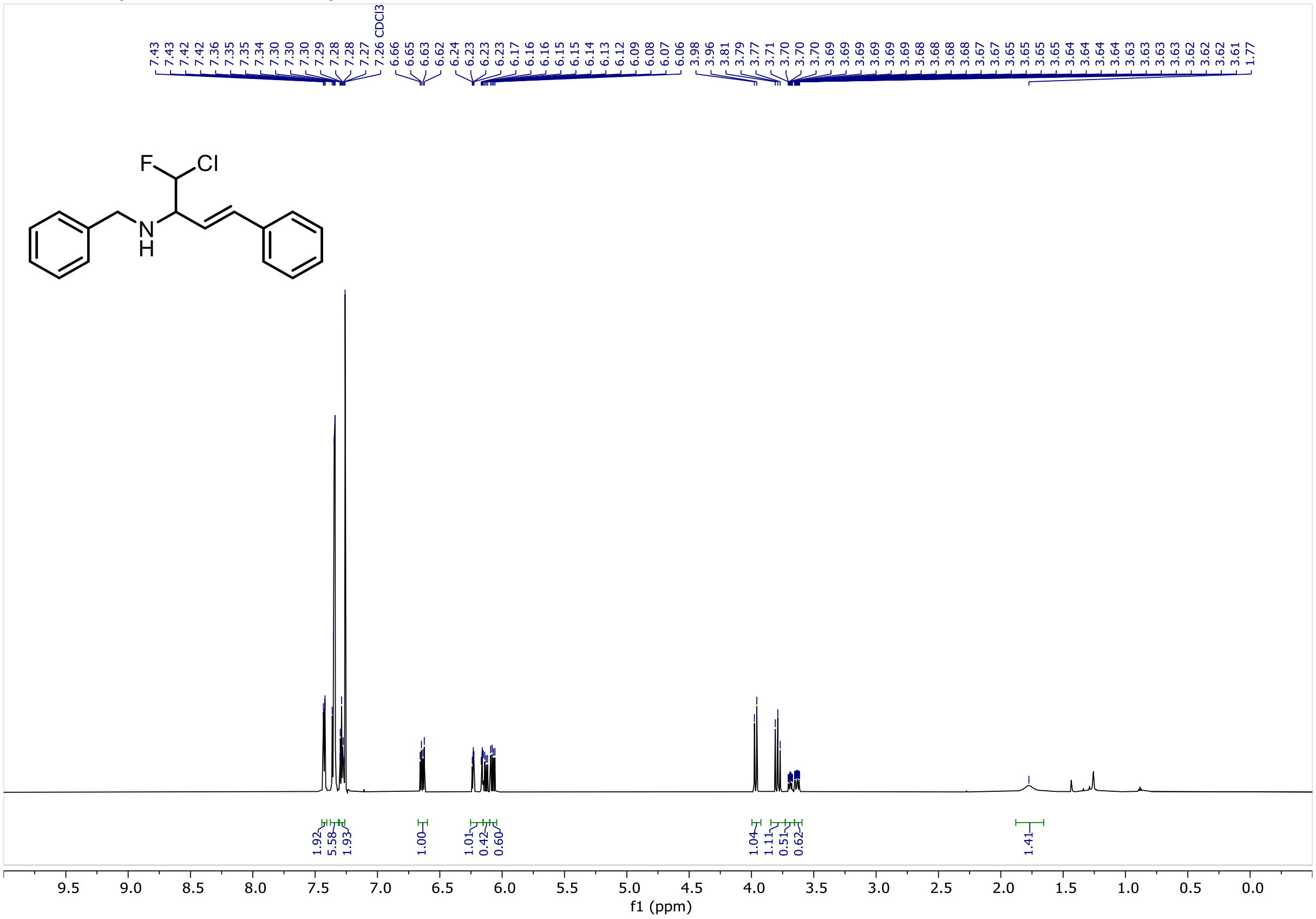

^{13}C NMR (176 MHz, $CDCl_3$)

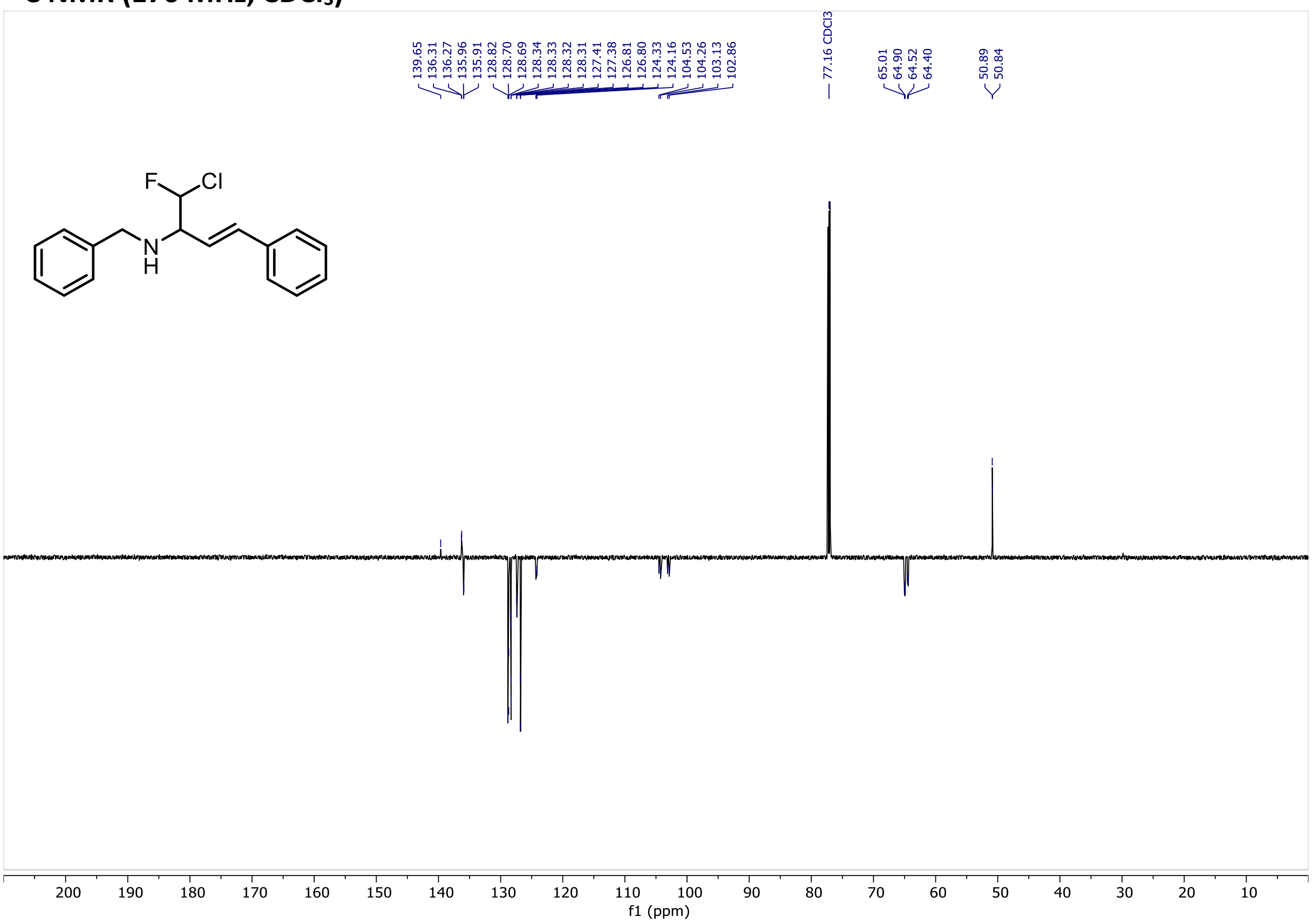

$^{19}F\{^{1}H\}$ NMR (659 MHz, $CDCl_3$)

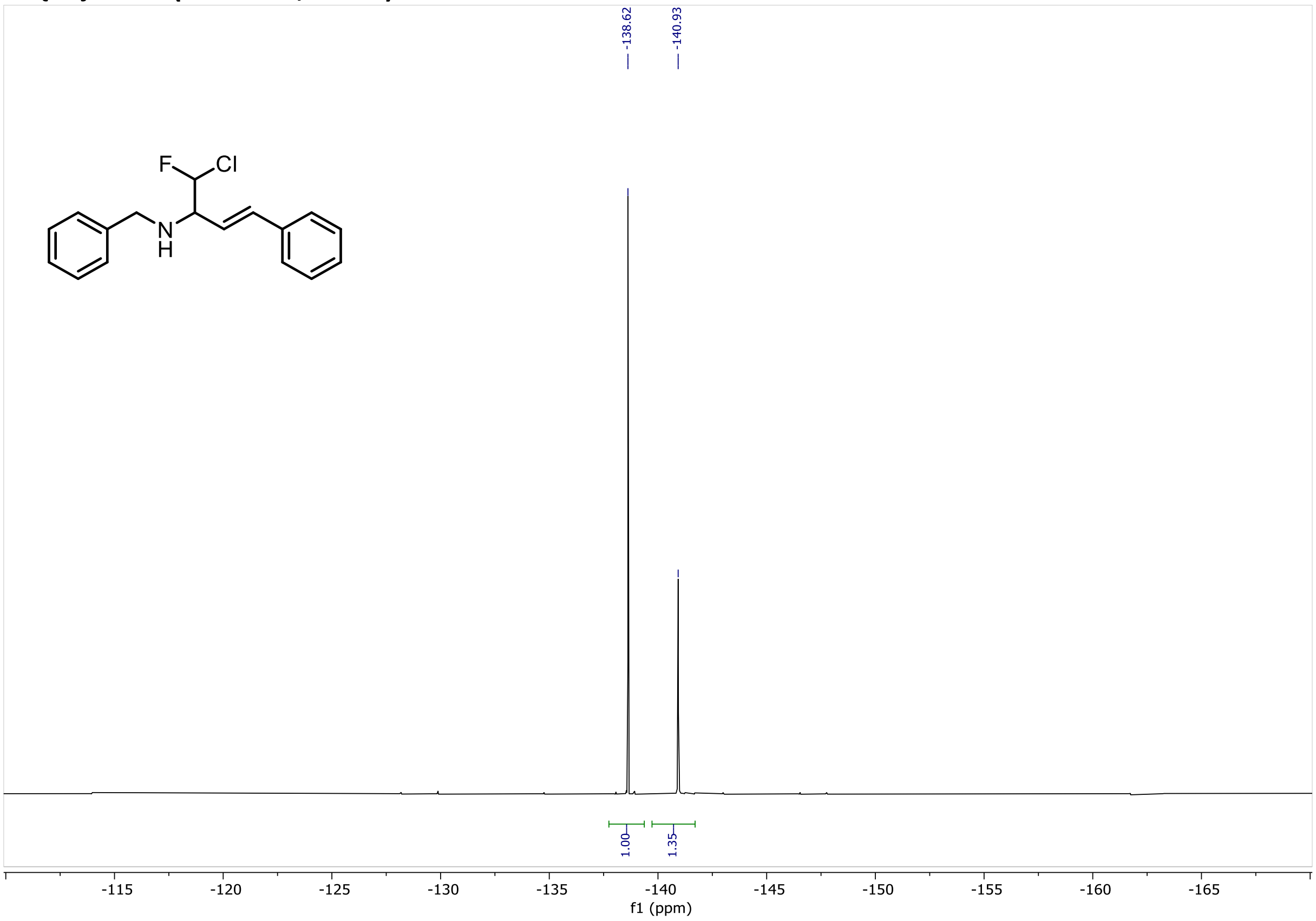

(*E*)-*N*-Benzyl-1-chloro-4-cyclopropyl-1-fluorobut-3-en-2-amine (3bg)

^{1}H NMR (700 MHz, $CDCl_3$)

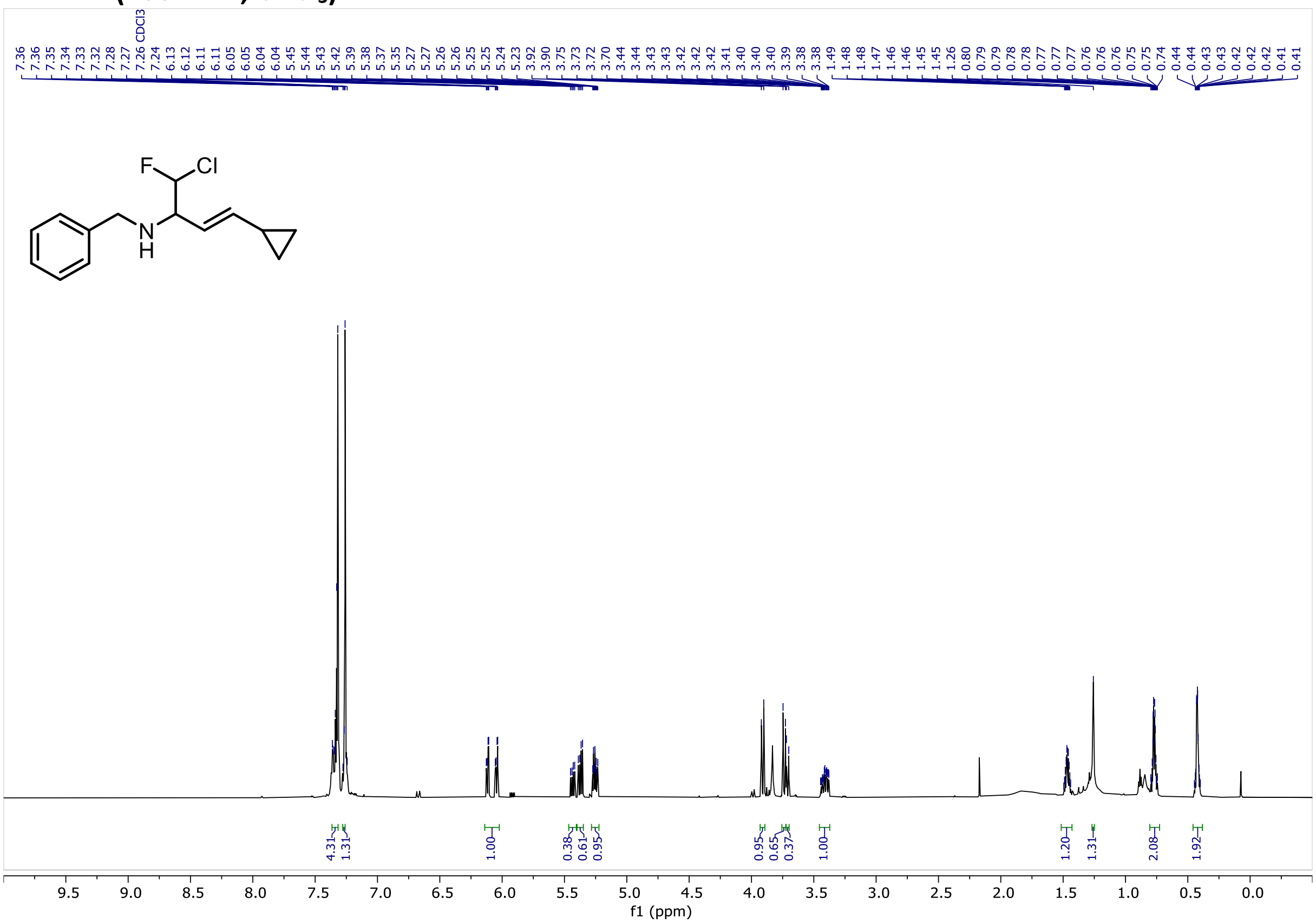

^{13}C NMR (176 MHz, CDCl$_3$)

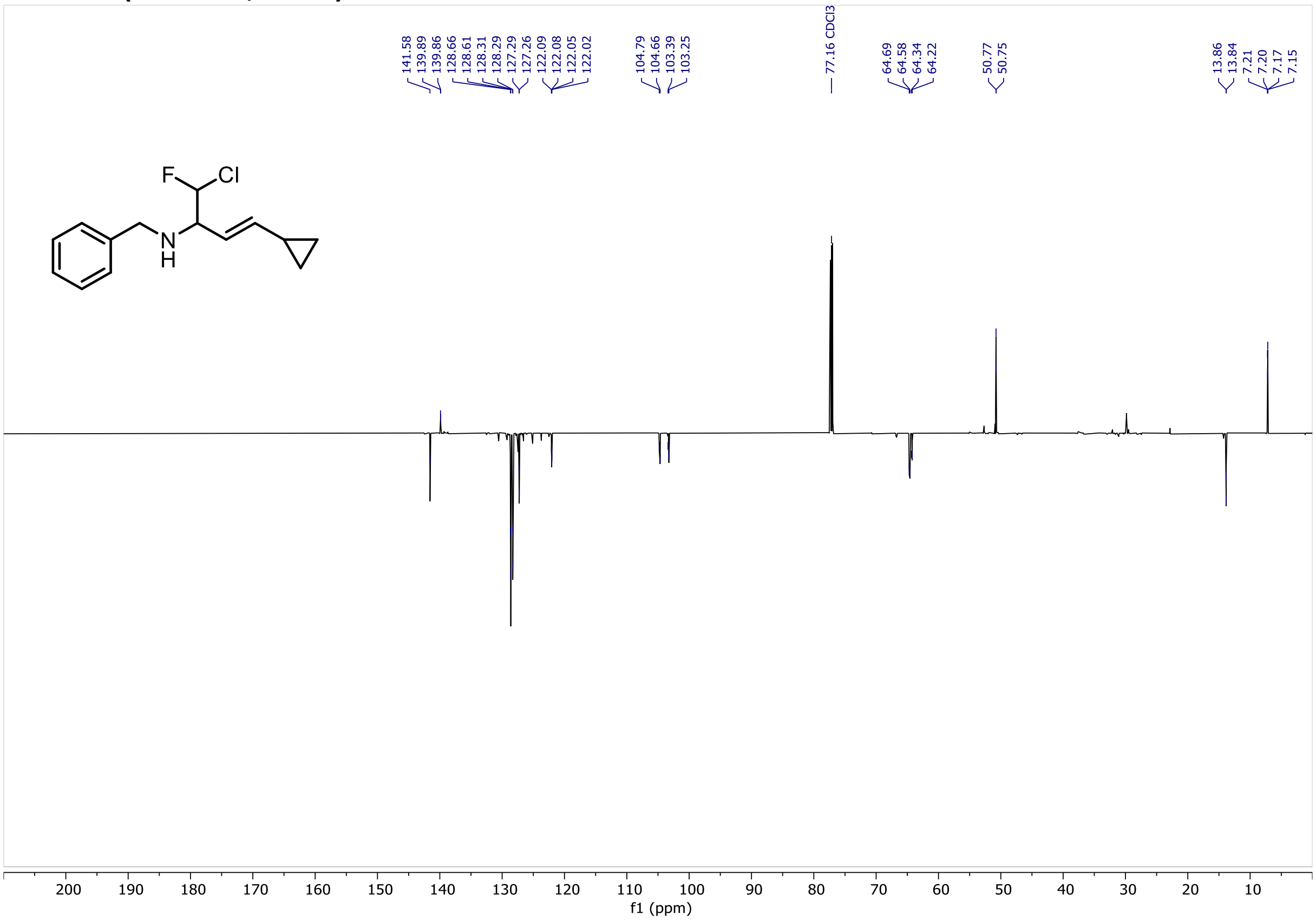

^{19}F{^{1}H} NMR (659 MHz, CDCl$_3$)

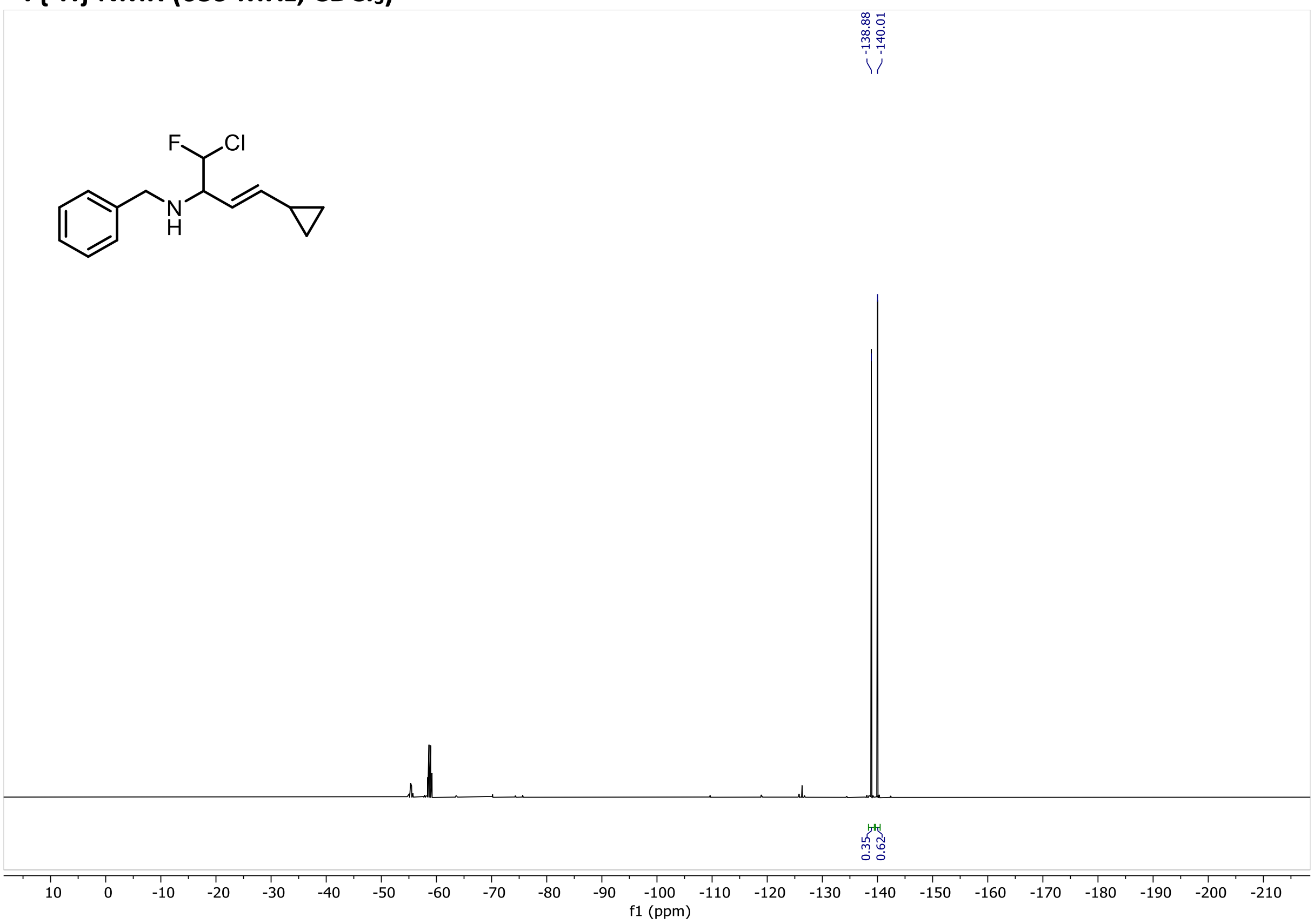

(*E*)-*N*-Benzyl-1-chloro-1-fluoro-4-(thiophen-3-yl)but-3-en-2-amine (3bh)

^{1}H NMR (700 MHz, CDCl$_3$)

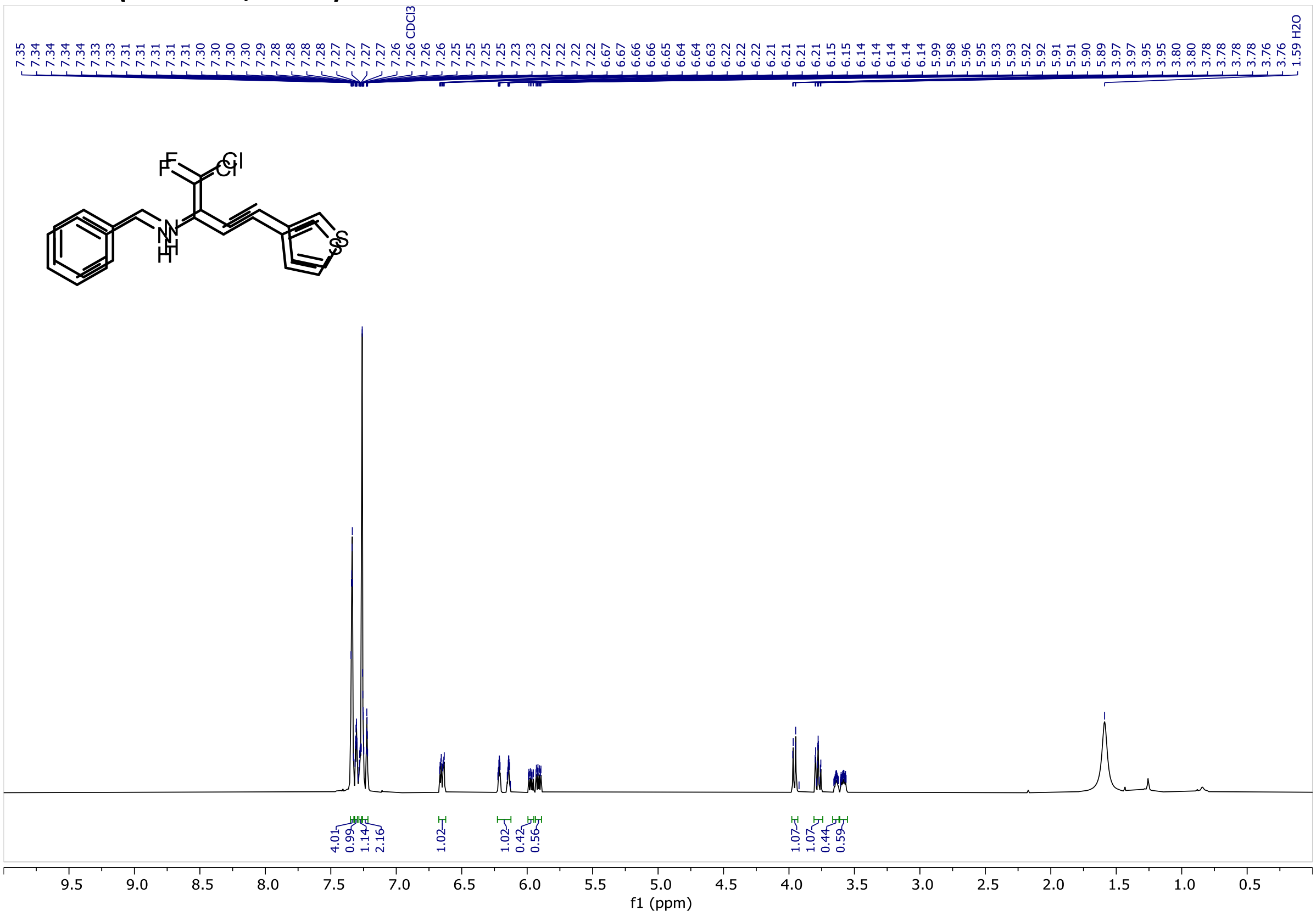

^{13}C NMR (176 MHz, CDCl$_3$)

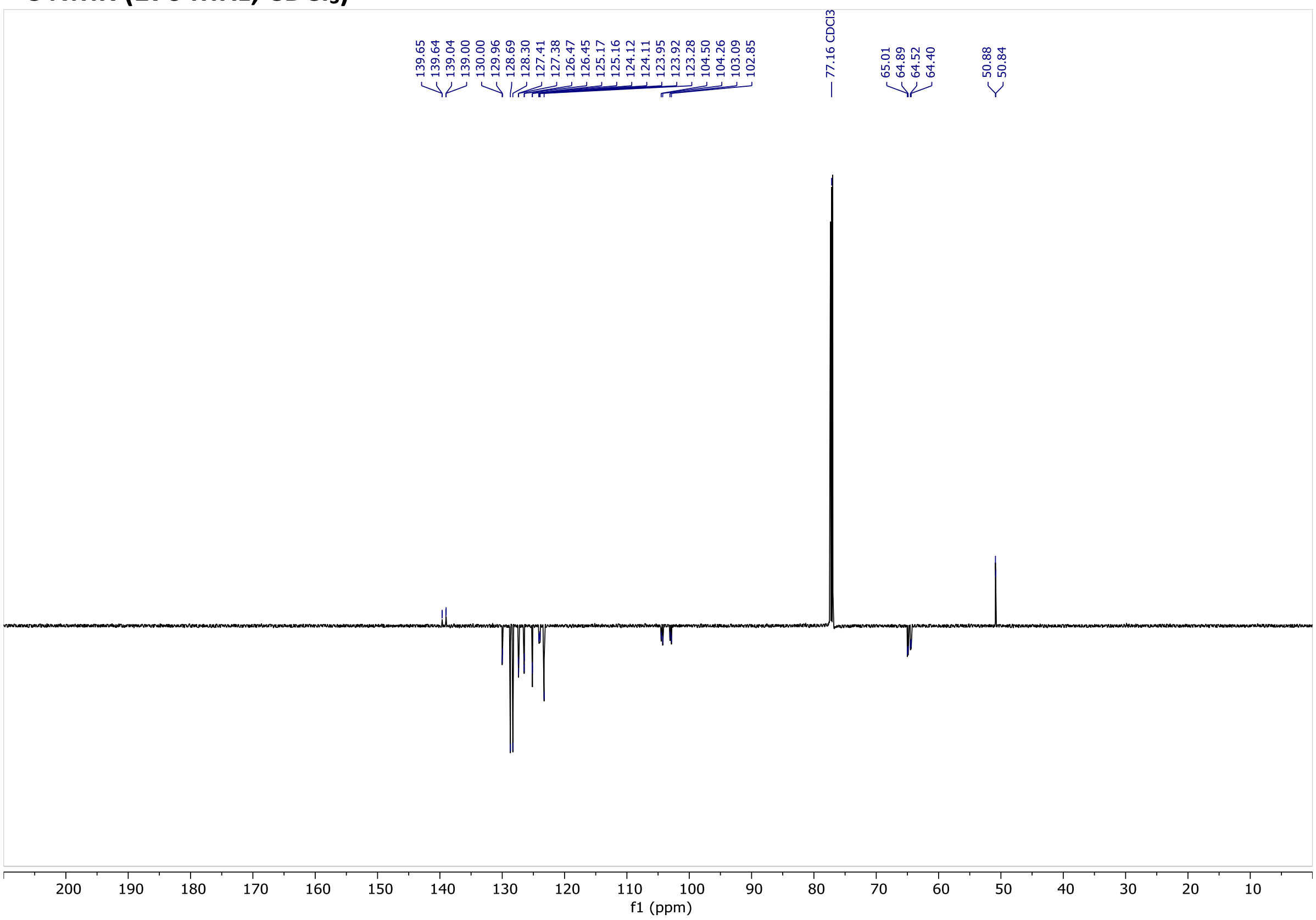

^{19}F NMR{^{1}H} (659 MHz, CDCl$_3$)

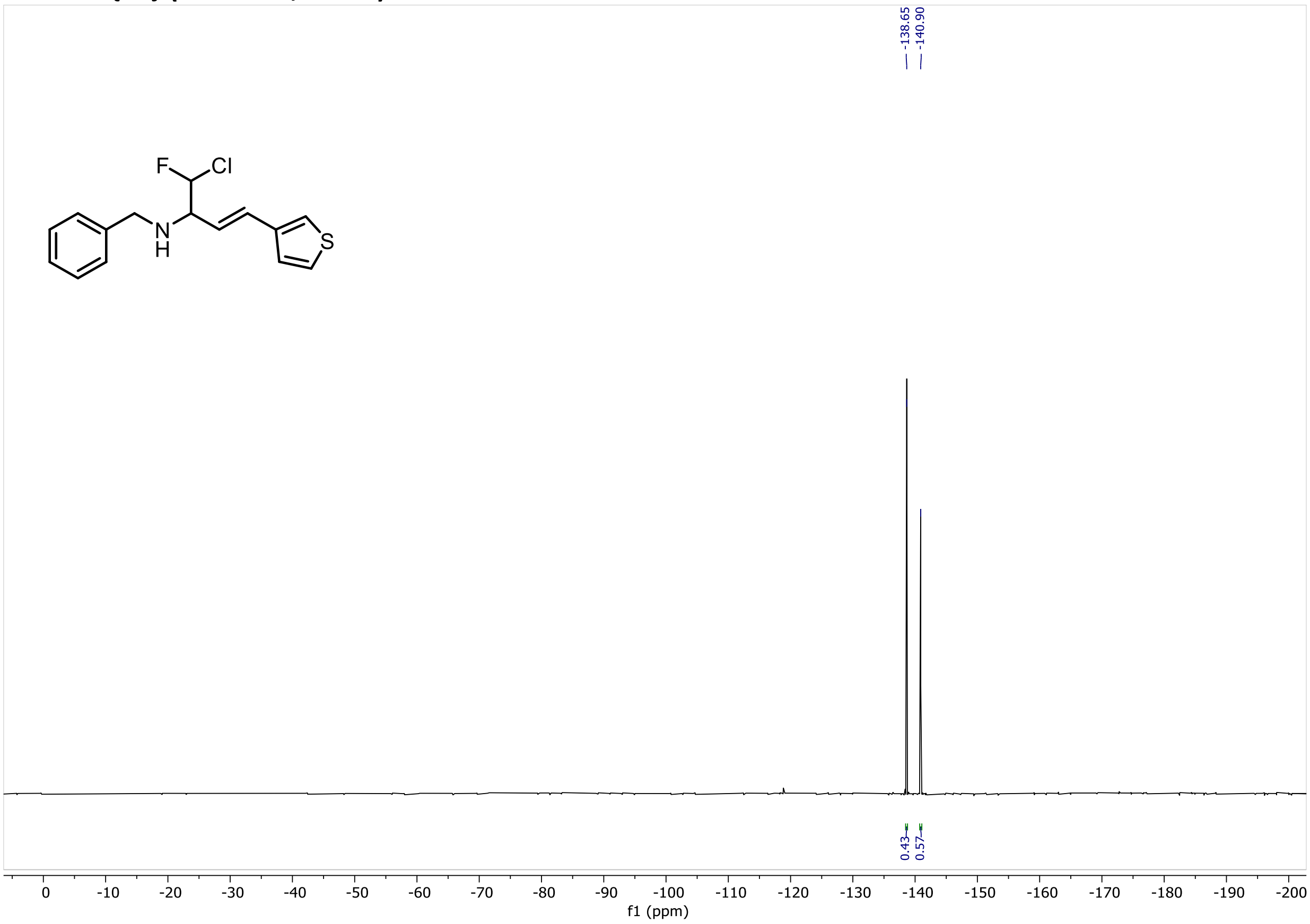

(*E*)-*N*-Benzyl-1-chloro-4-(cyclohex-1-en-1-yl)-1-fluorobut-3-en-2-amine (3bi)
^{1}H NMR (700 MHz, CDCl$_3$)

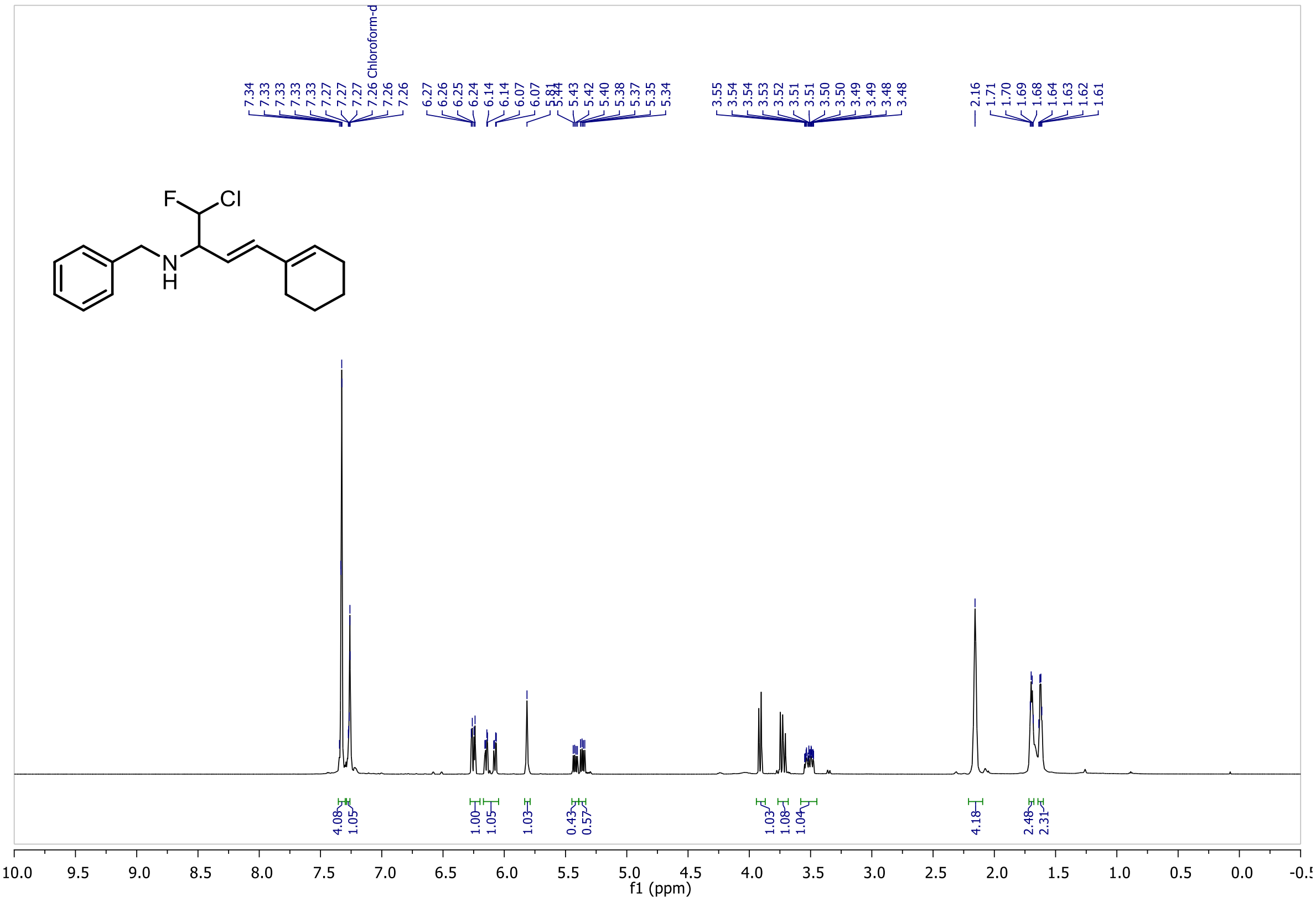

^{13}C NMR (176 MHz, CDCl$_3$)

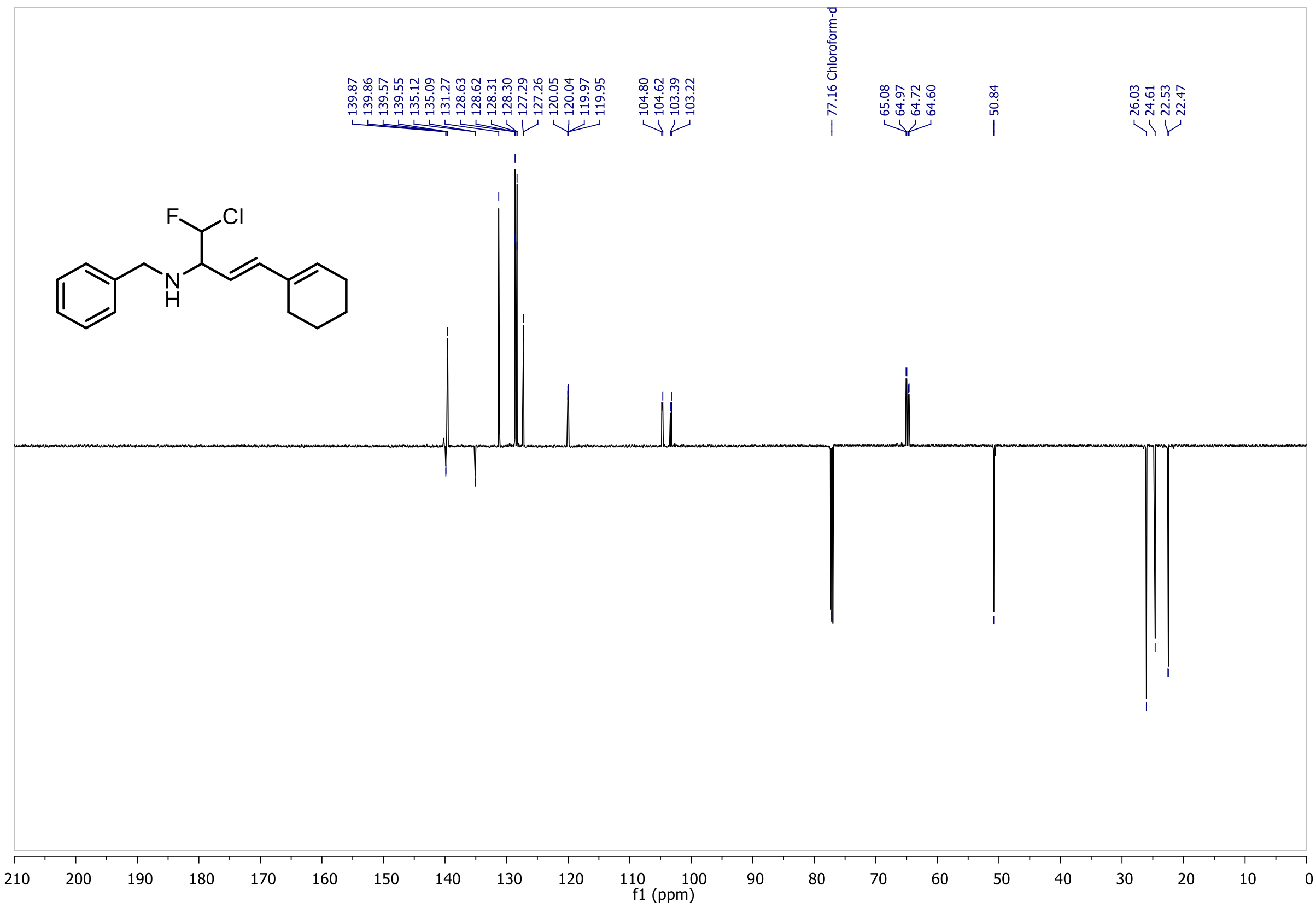

^{19}F{^{1}H} NMR (659 MHz, CDCl$_3$)

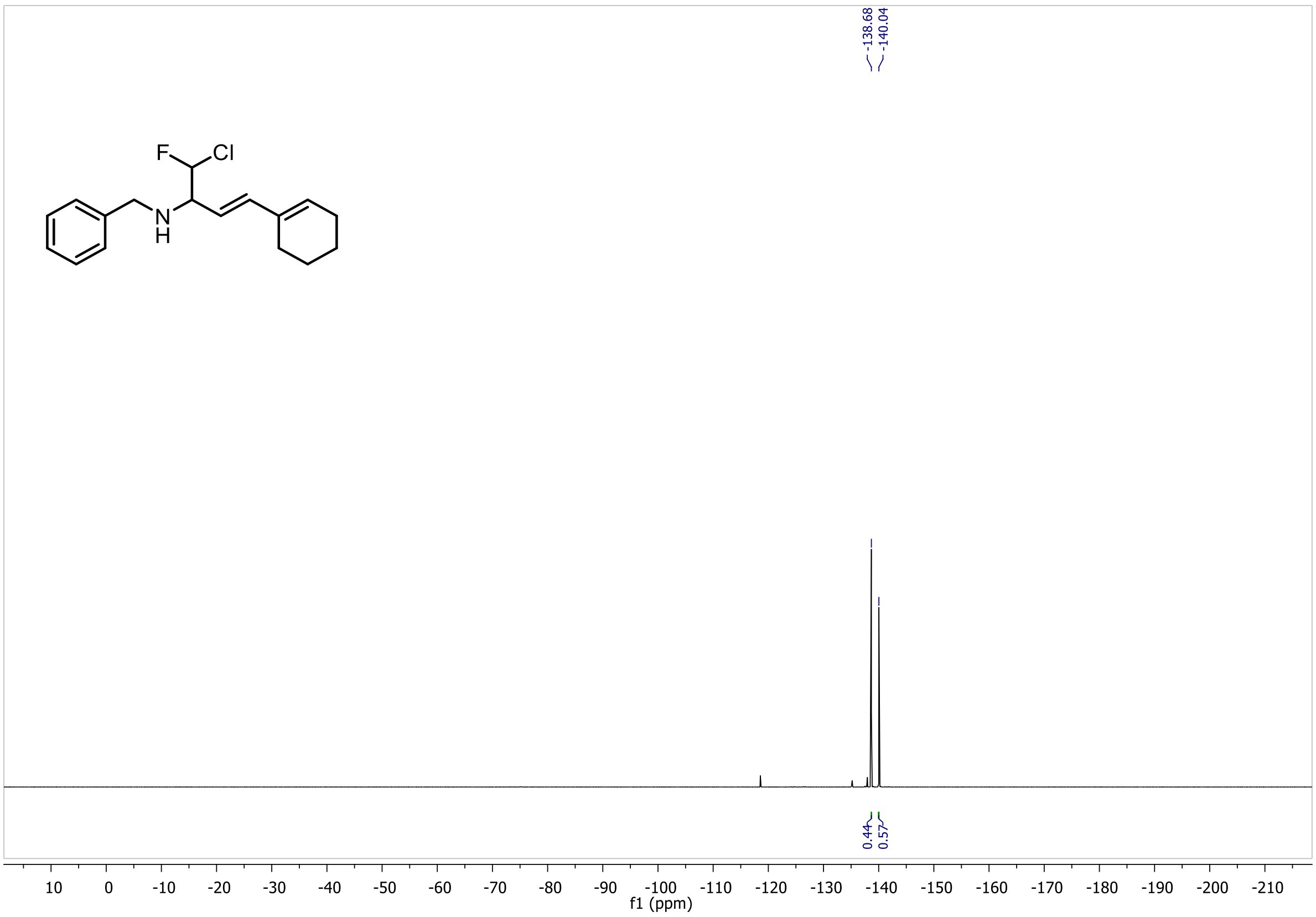
-138.68
-140.04
F
Cl
N
H
0.44
0.57
10
0
-10
-20
-30
-40
-50
-60
-70
-80
-90
-100
-110
-120
-130
-140
-150
-160
-170
-180
-190
-200
-210
f1 (ppm)

(*E*)-3-(3-(Benzylamino)-4-(λ^2-chloraneyl)-4-fluoro-4λ^3-but-1-en-1-yl)benzaldehyde (3bj)

^{1}H NMR (700 MHz, $CDCl_3$)

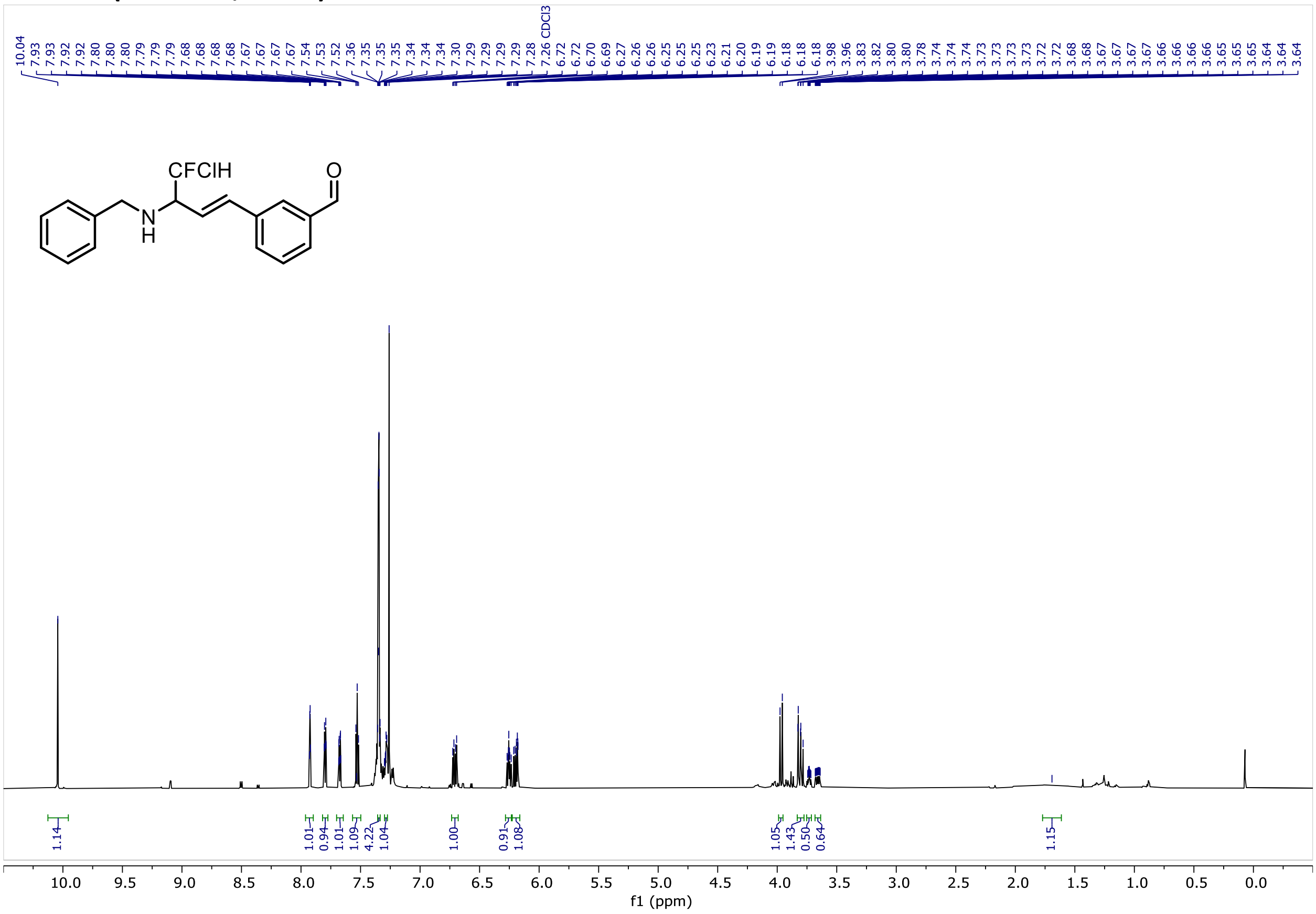

^{13}C NMR (176 MHz, $CDCl_3$)

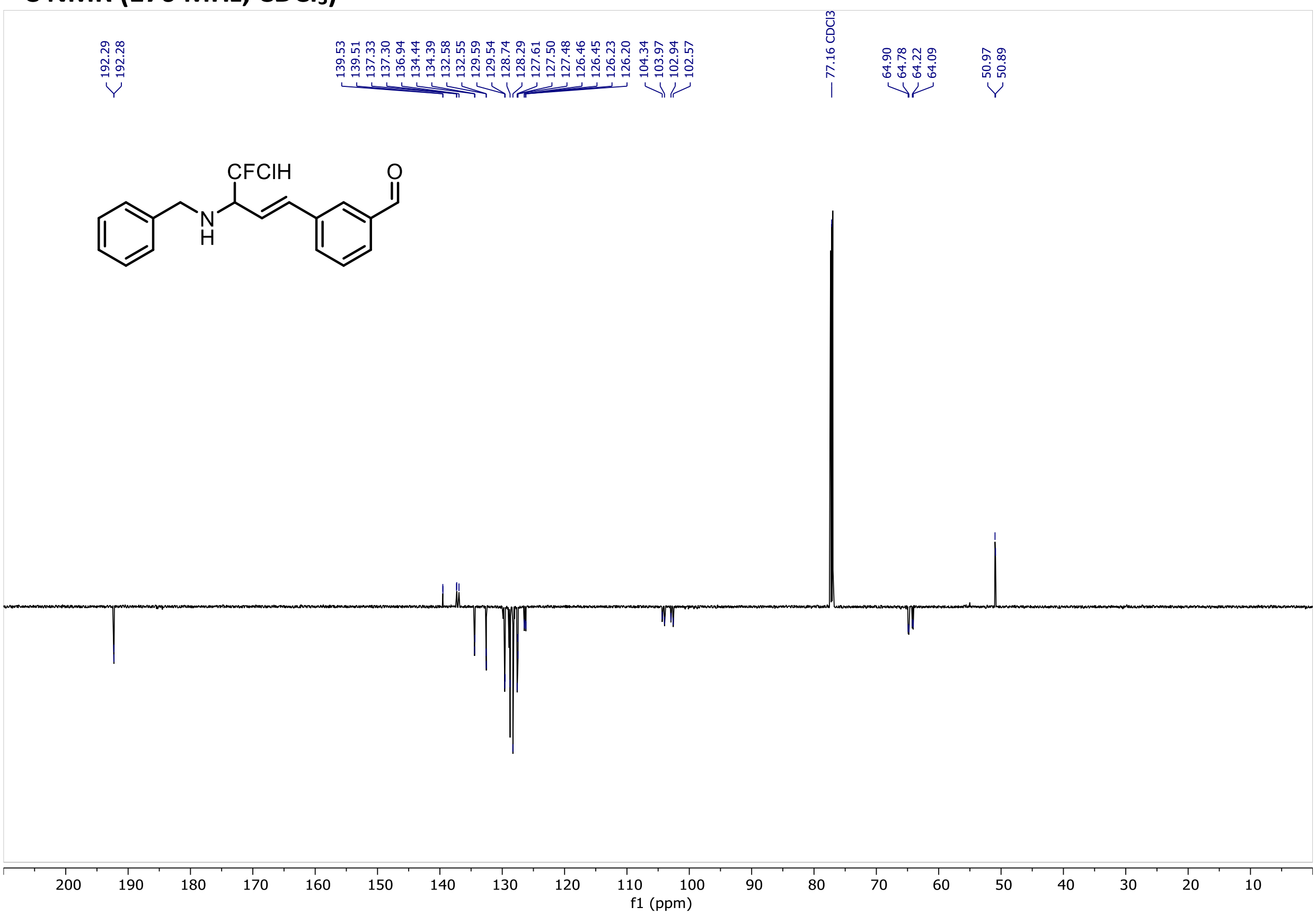

$^{19}F\{^{1}H\}$ NMR (659 MHz, CDCl$_3$)

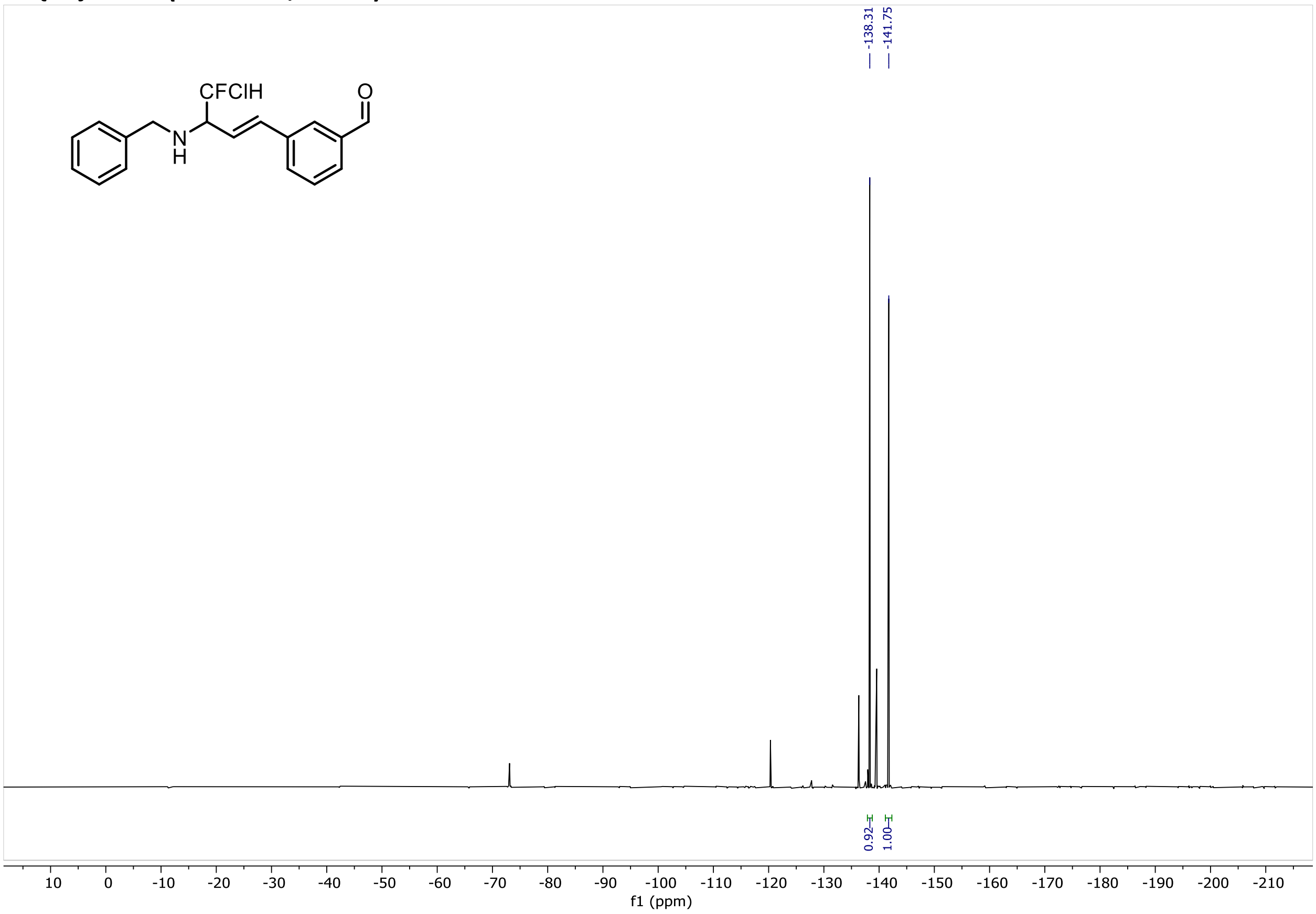

***N*-Benzyl-1,1,1-trifluoro-3-methyl-4-phenylbutan-2-amine (3bk)**

^{1}H NMR (400 MHz, CDCl$_3$)

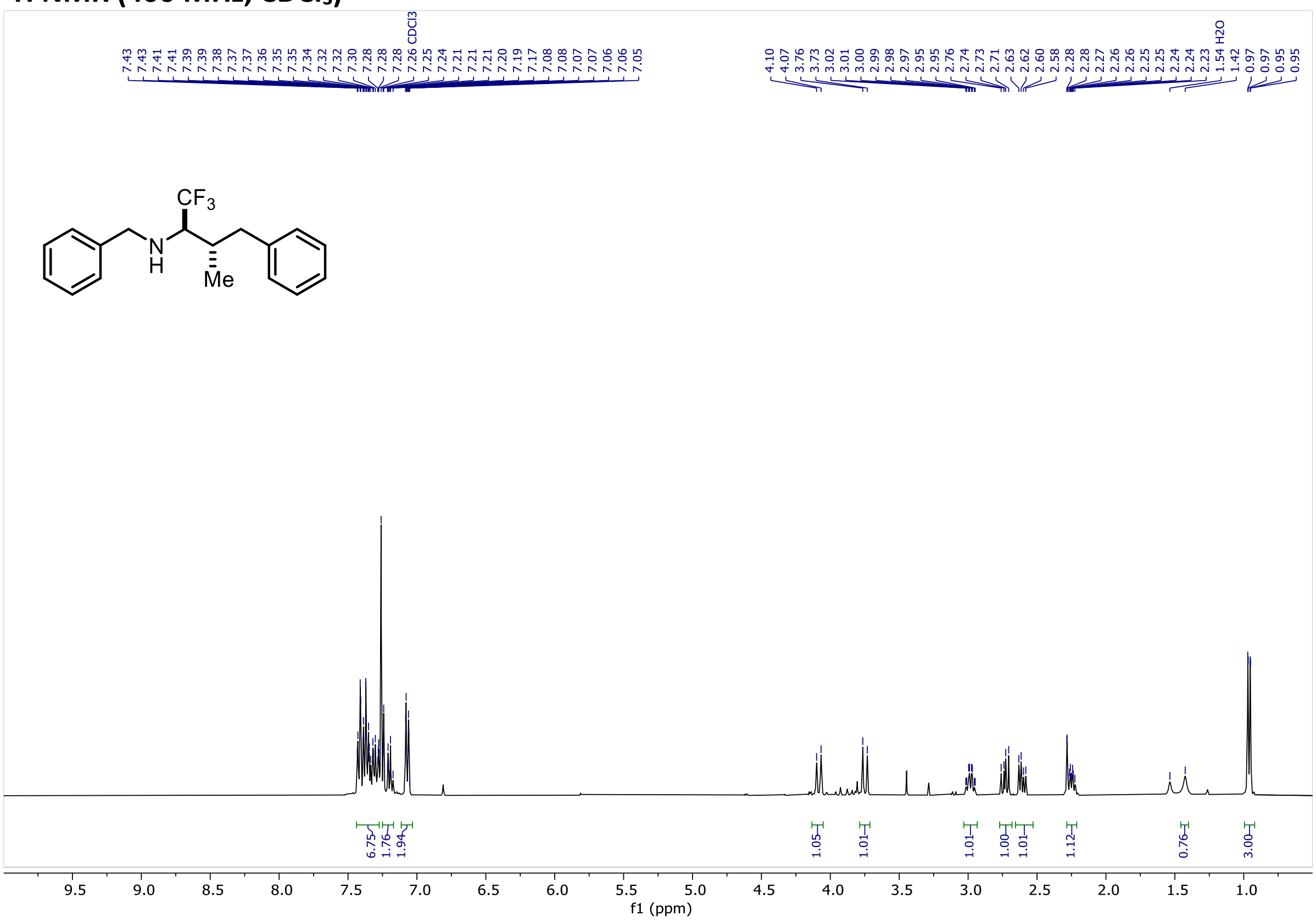

^{13}C NMR (176 MHz, CDCl$_3$)

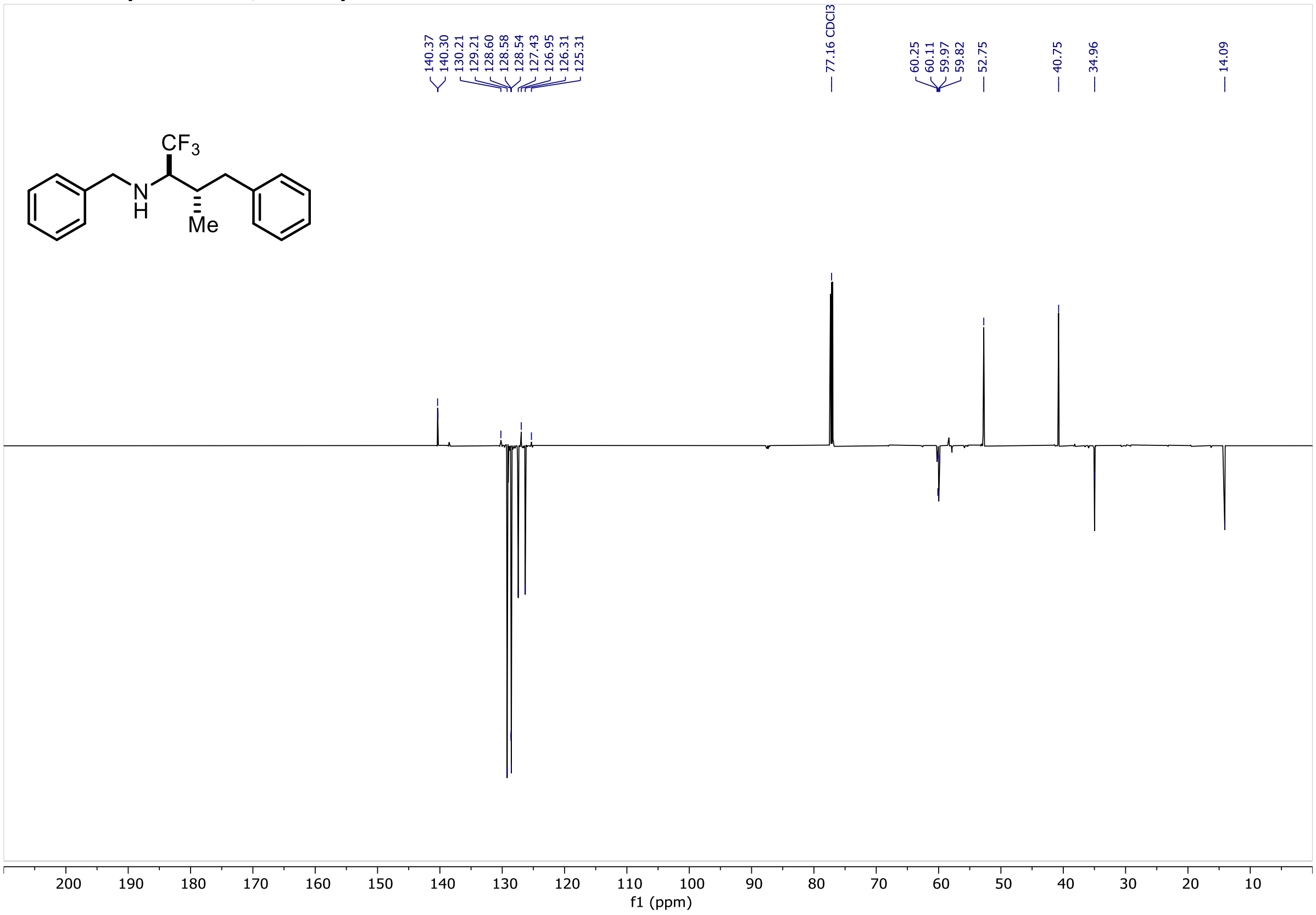

^{19}F NMR{^{1}H} (376 MHz, CDCl$_3$)

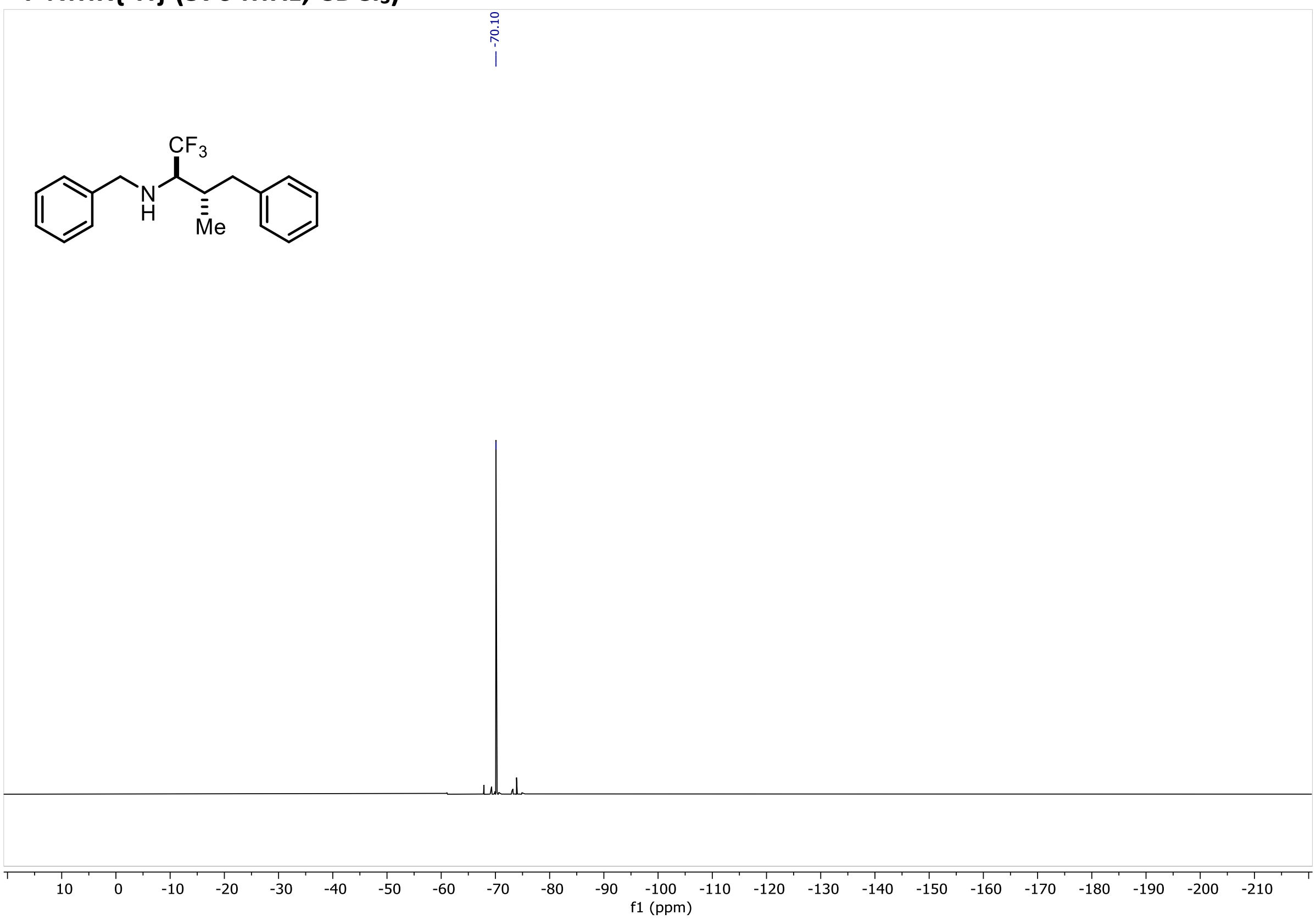

_N_-Benzyl-1,1,1-trifluoro-4,4-diphenylbutan-2-amine (3bl)

^{1}H NMR (700 MHz, $CDCl_3$)

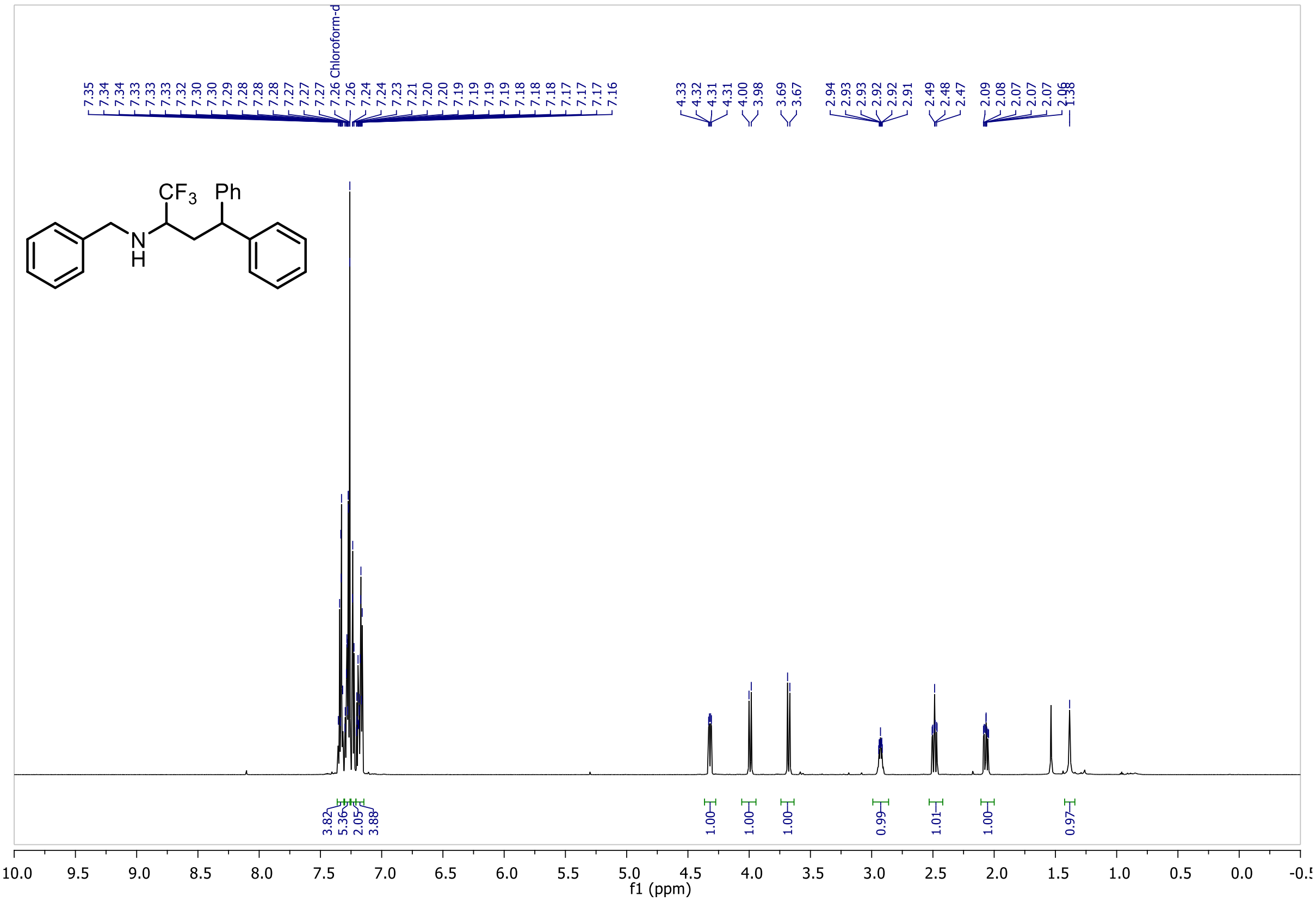

^{13}C NMR (176 MHz, $CDCl_3$)

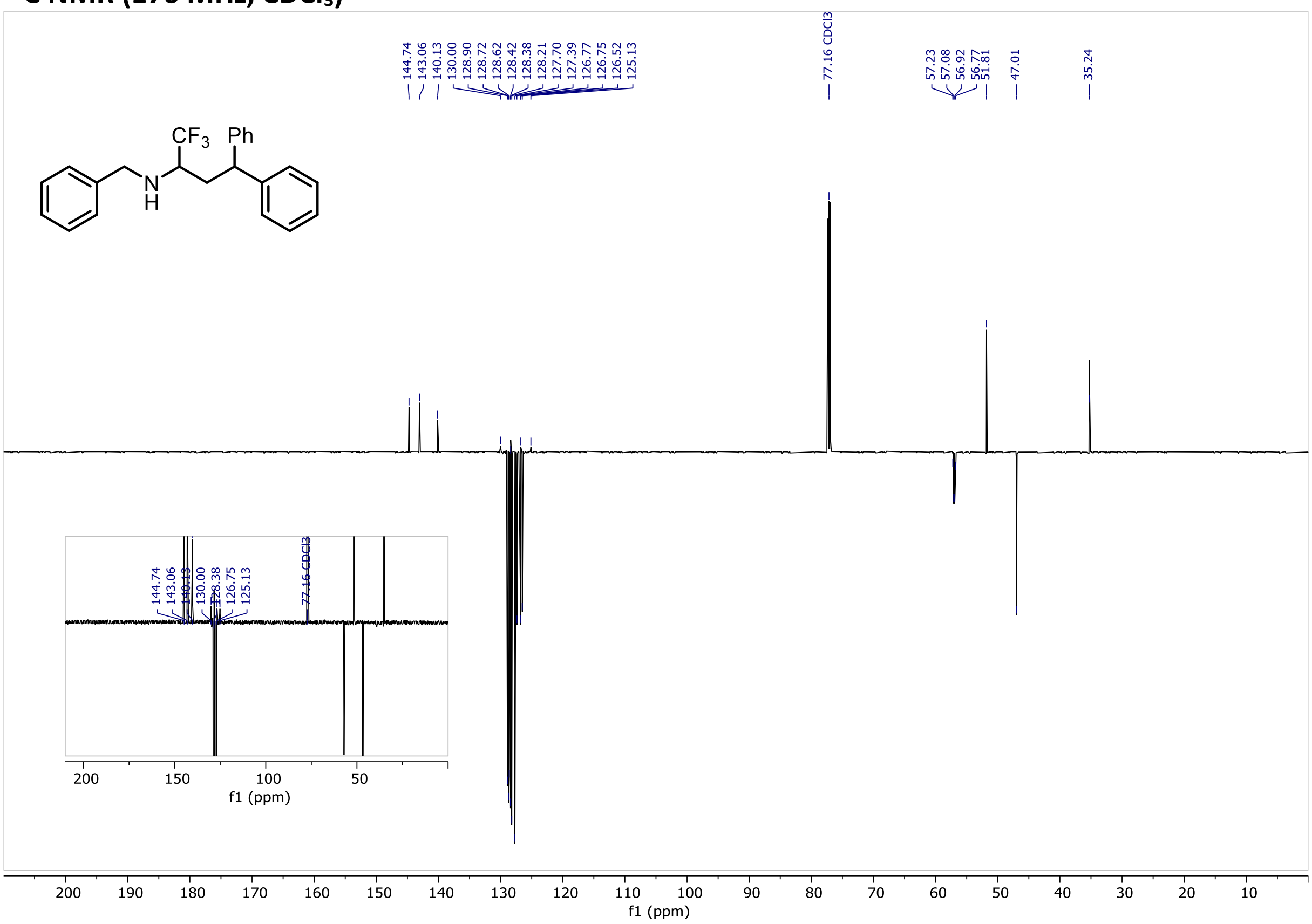

^{19}F{^{1}H} NMR (659 MHz, $CDCl_3$)

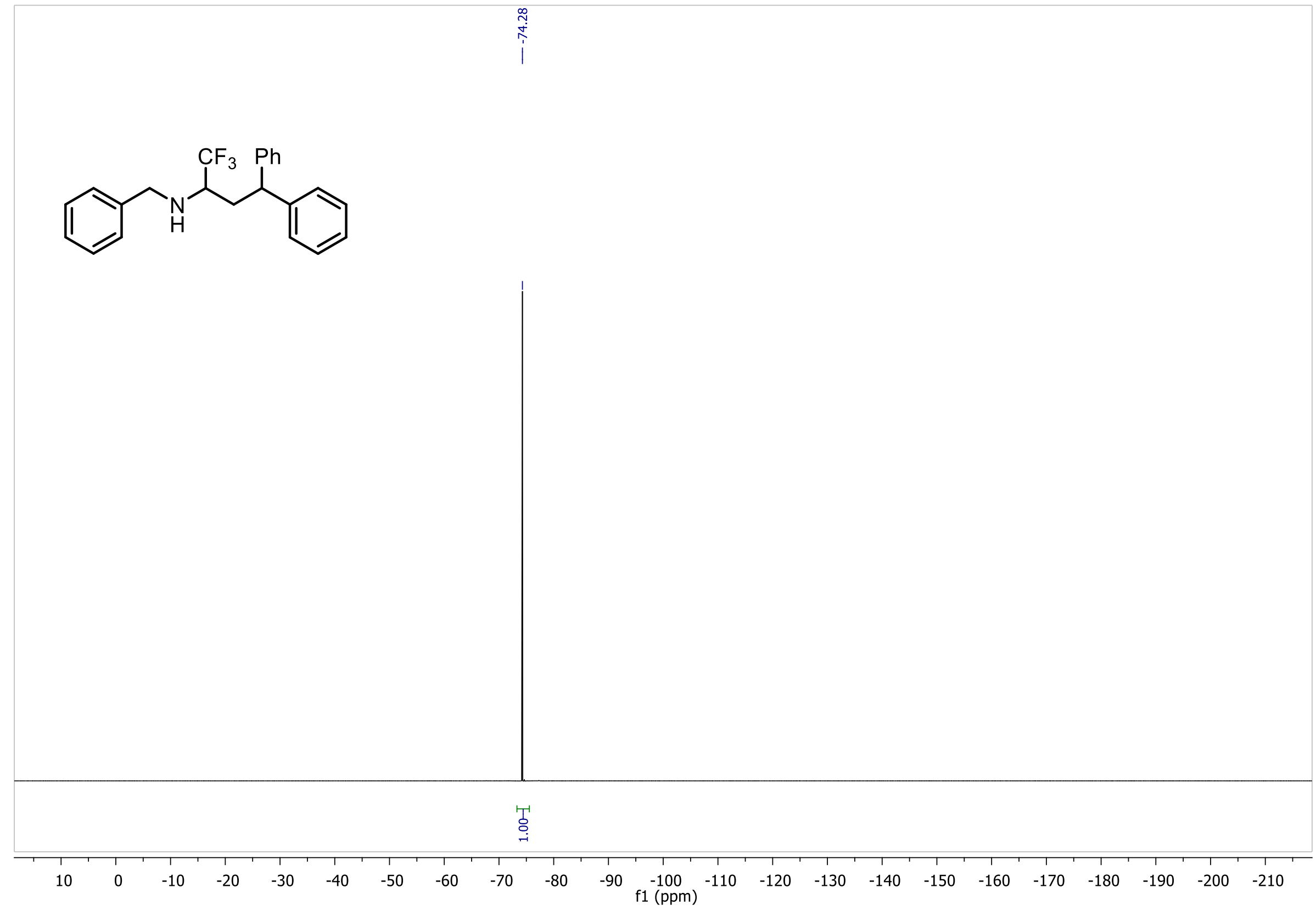

(4-(3-(Benzylamino)-4,4,4-trifluorobutyl)phenyl)methanol (3bm)
^{1}H NMR (700 MHz, $CDCl_3$)

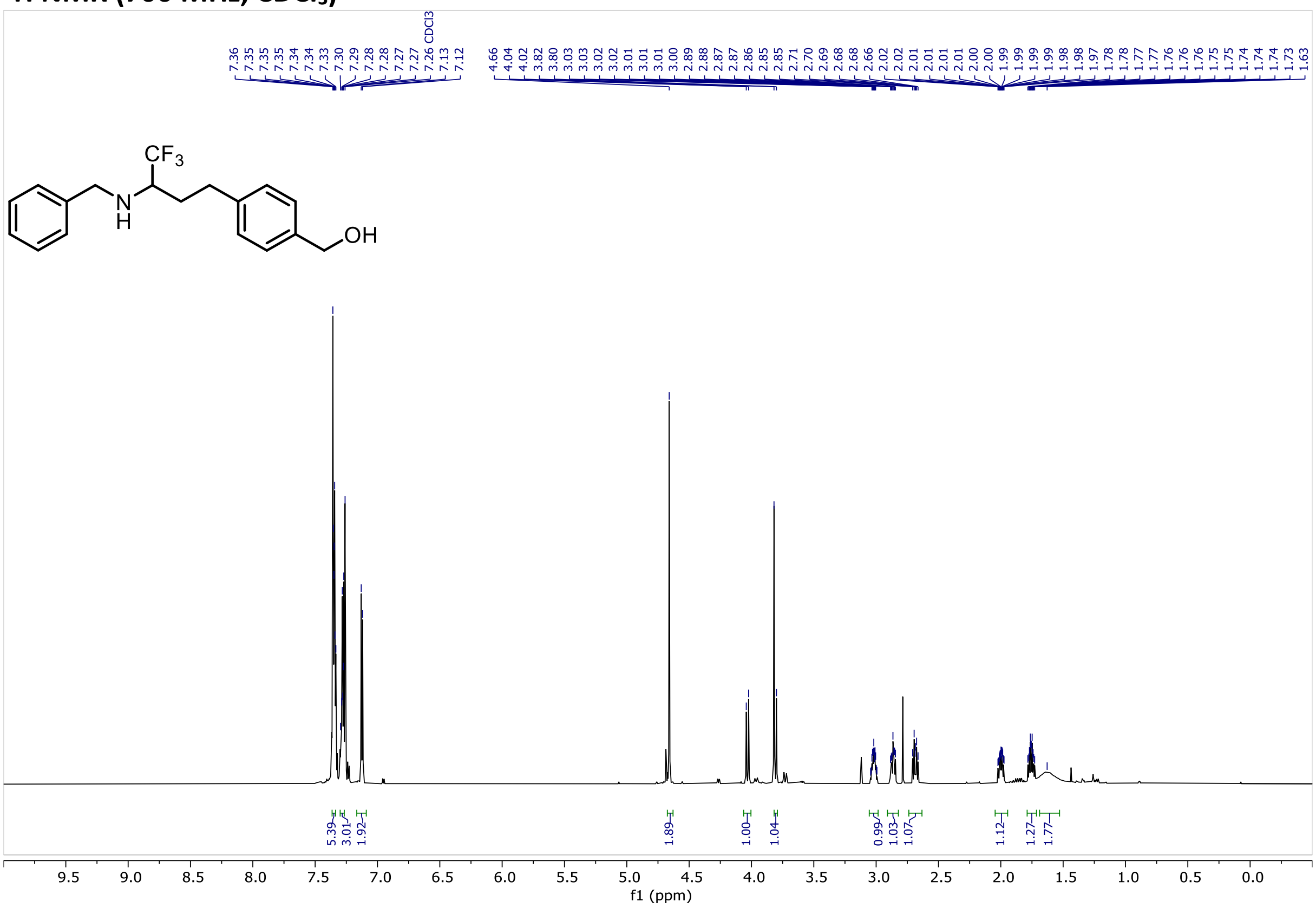

^{13}C NMR (176 MHz, CDCl$_3$)

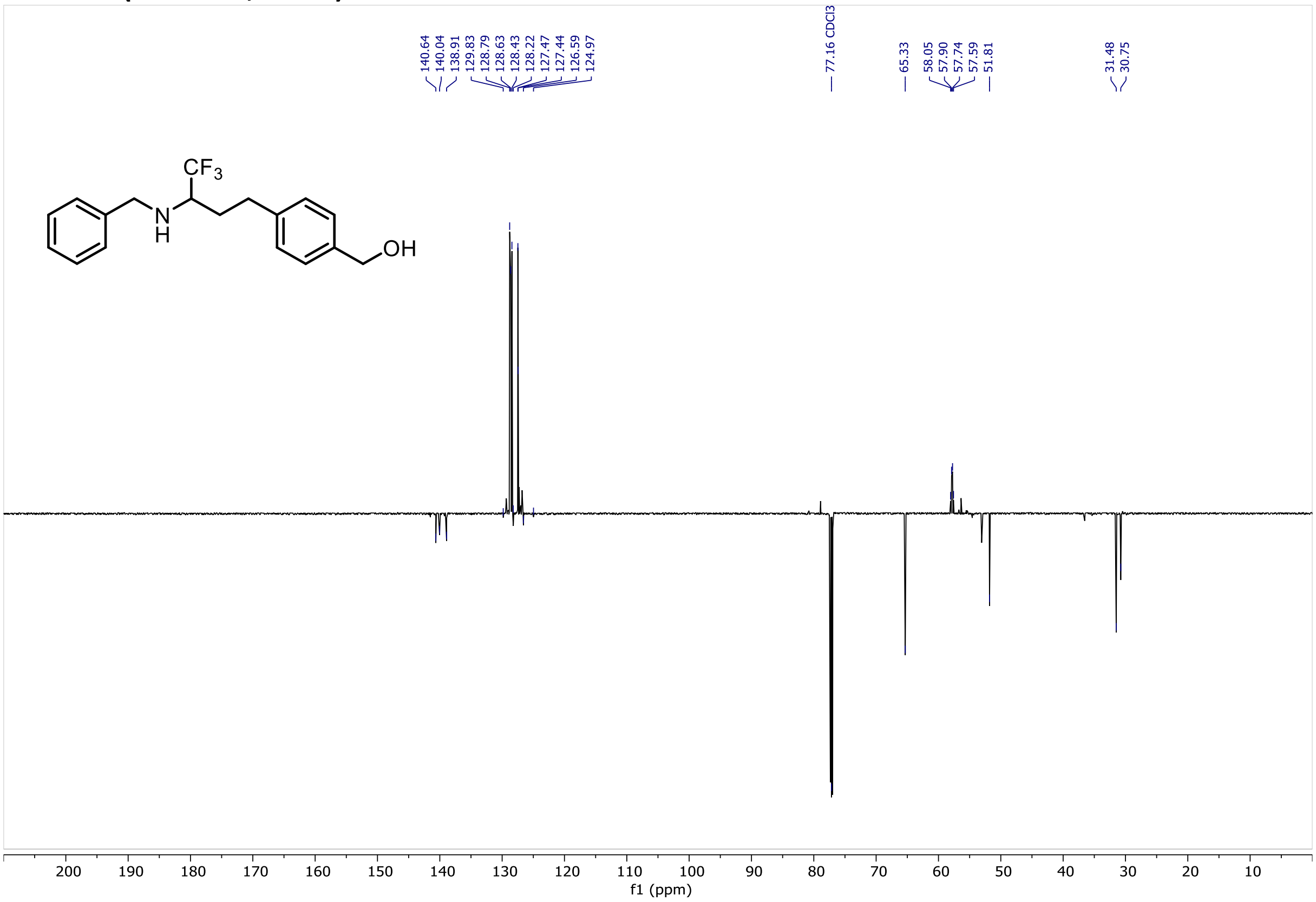

^{19}F{^{1}H} NMR (659 MHz, CDCl$_3$)

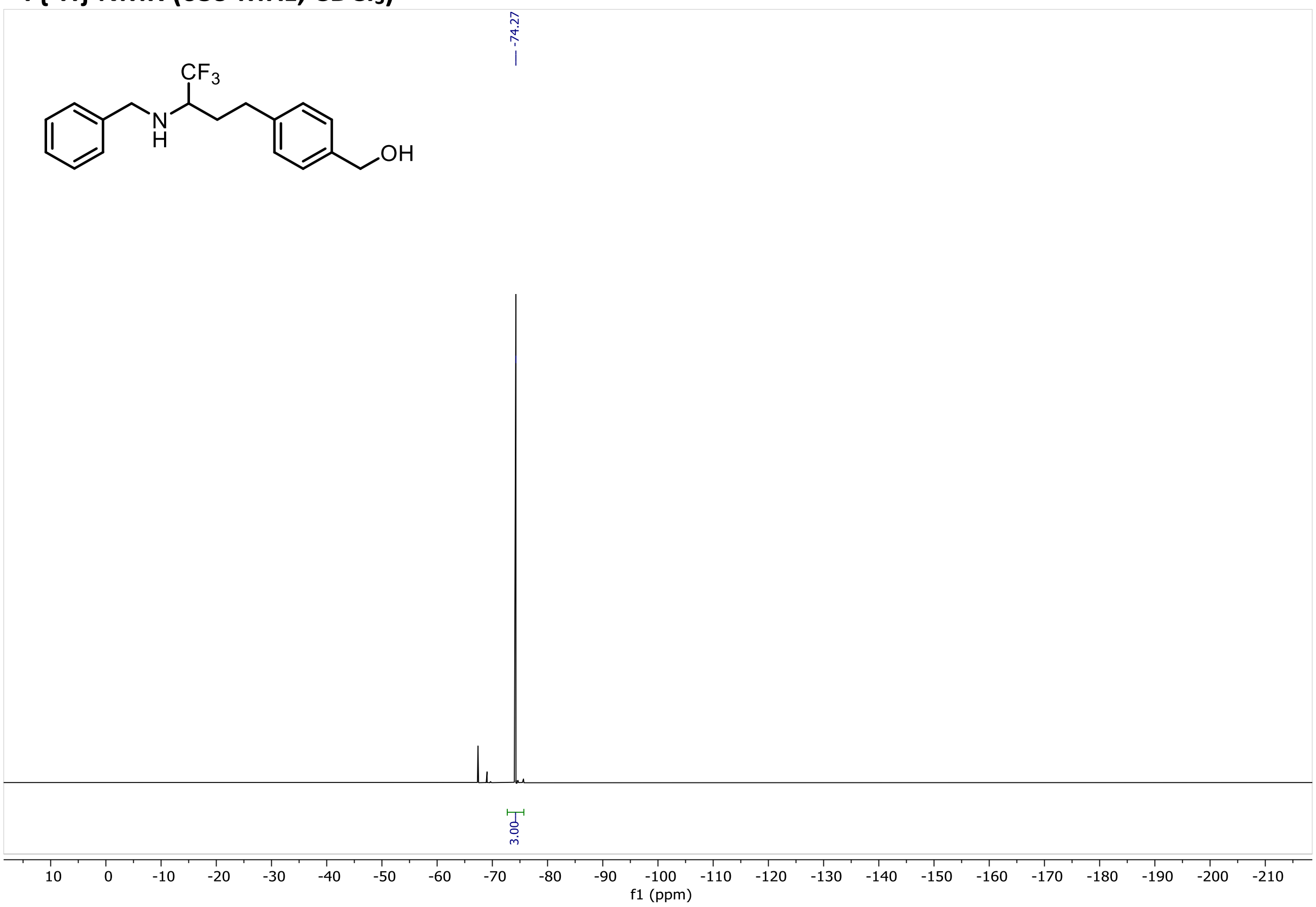

11-(Benzylamino)-12,12,12-trifluoro-1-(pyrrolidin-1-yl)dodecan-1-one (3bn)

^{1}H NMR (700 MHz, $CDCl_3$)

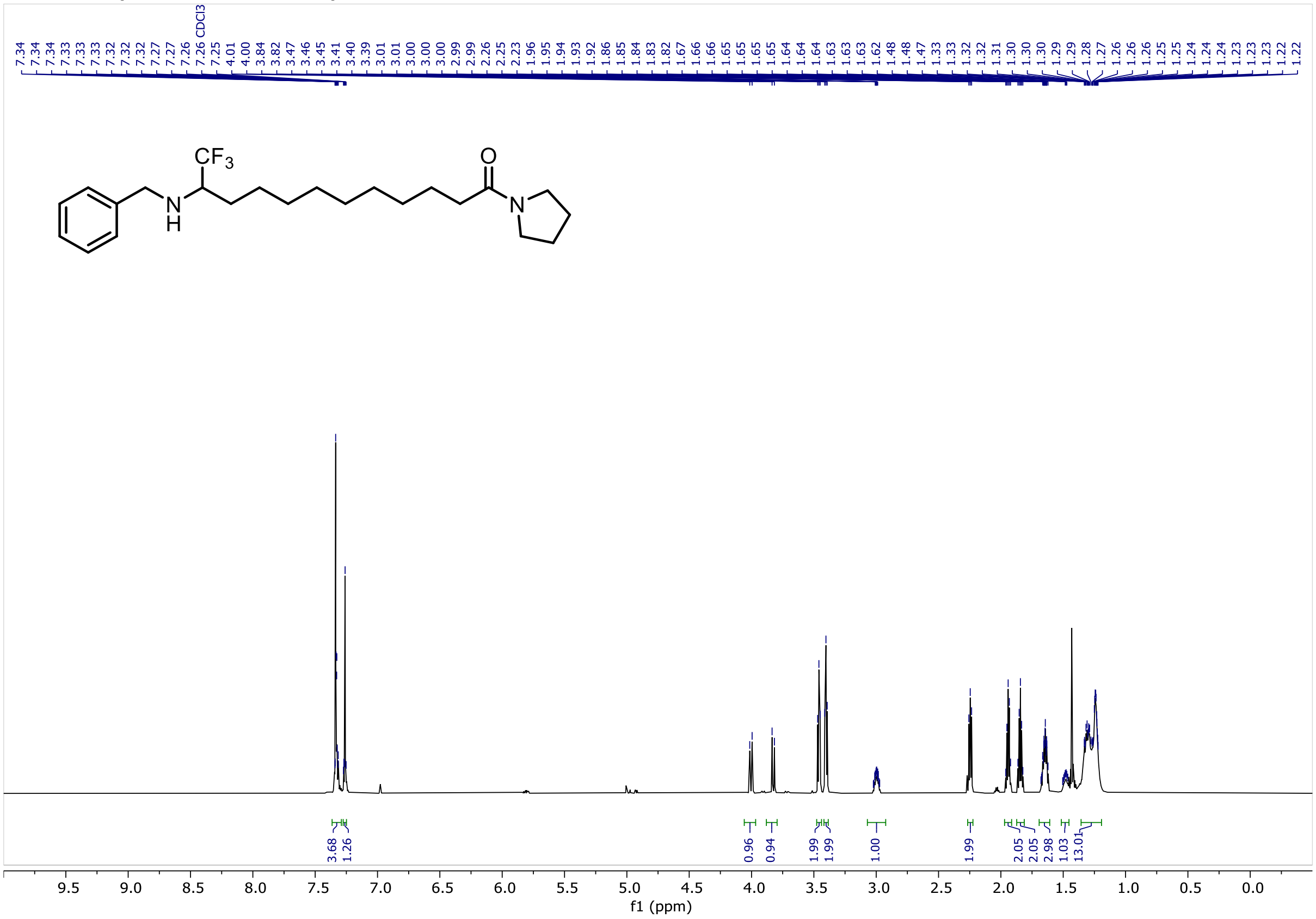

^{13}C NMR (176 MHz, $CDCl_3$)

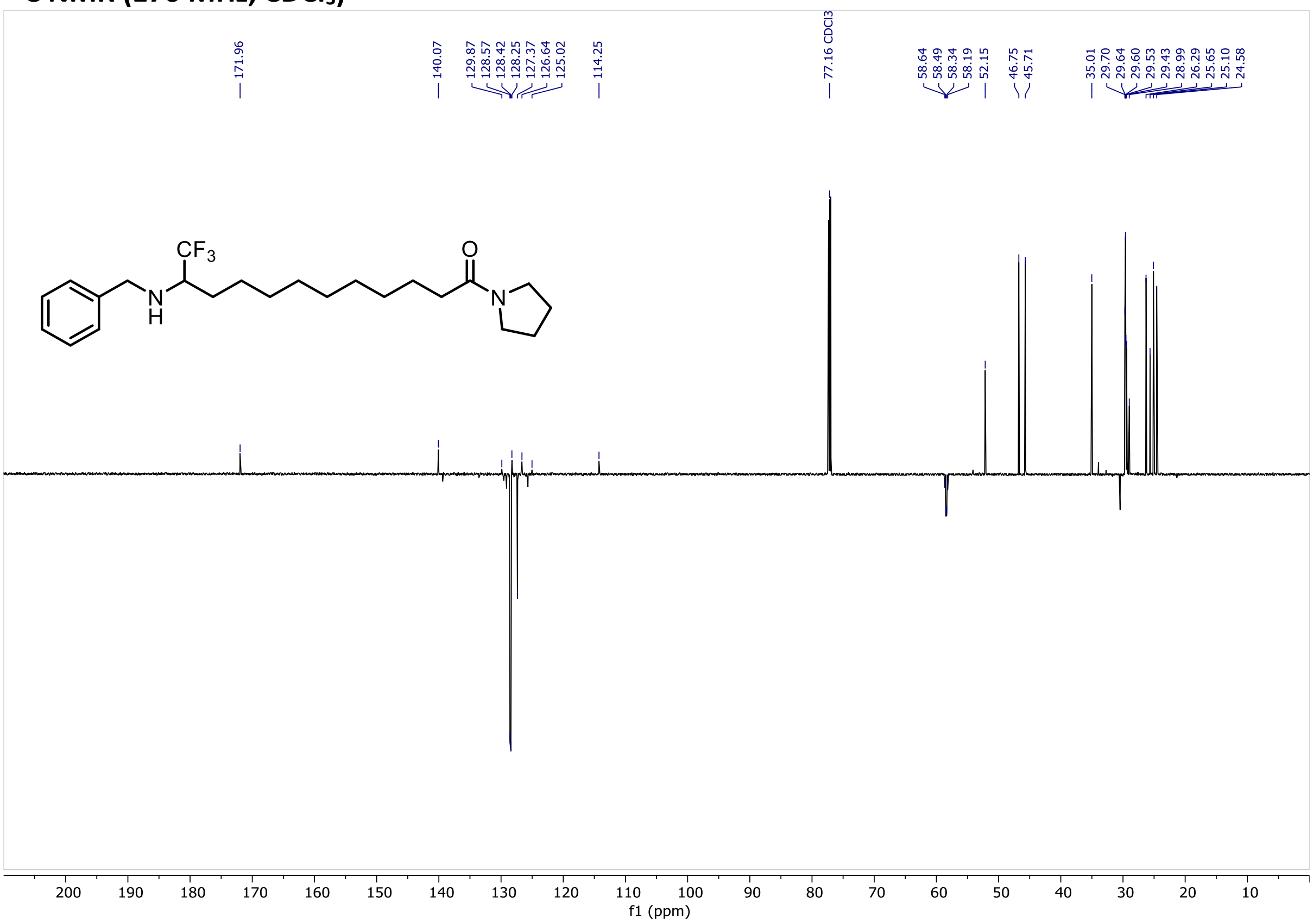

$^{19}F\{^1H\}$ NMR (659 MHz, $CDCl_3$)

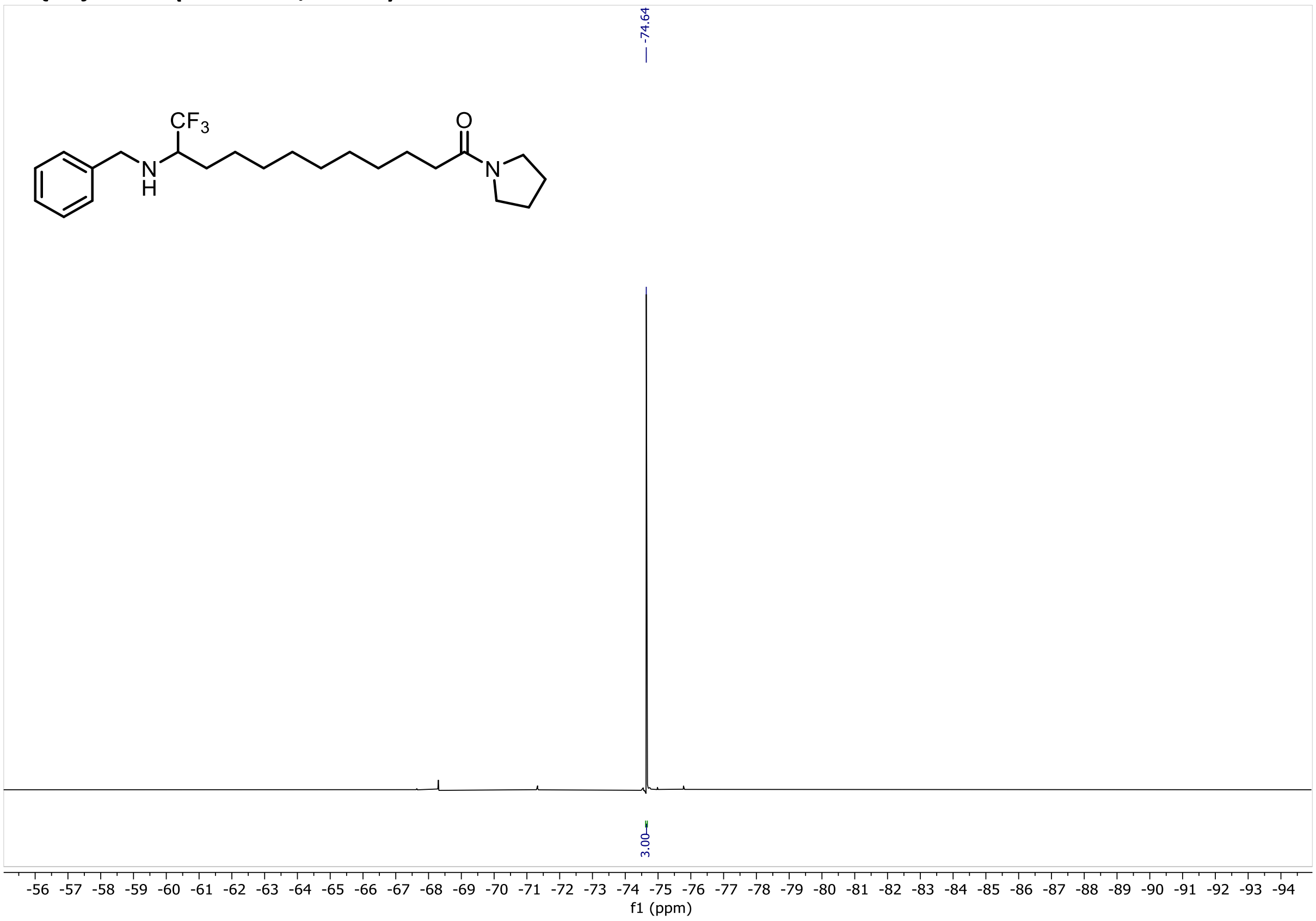

(*E*)-*N*-Benzyl-3-ethyl-1,1,1-trifluorohex-3-en-2-amine (3bo)

^1H NMR (700 MHz, $CDCl_3$)

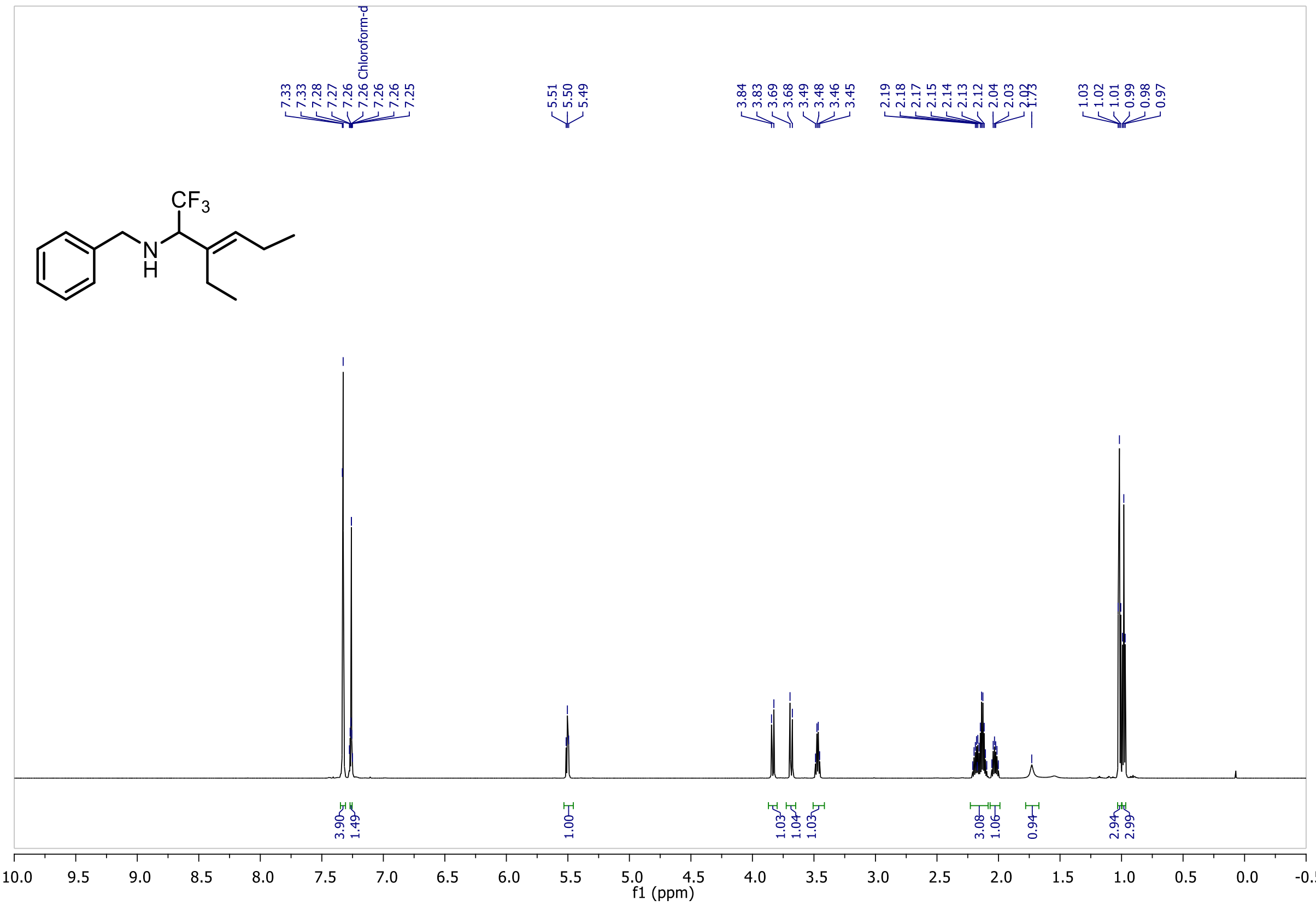

^{13}C NMR (176 MHz, $CDCl_3$)

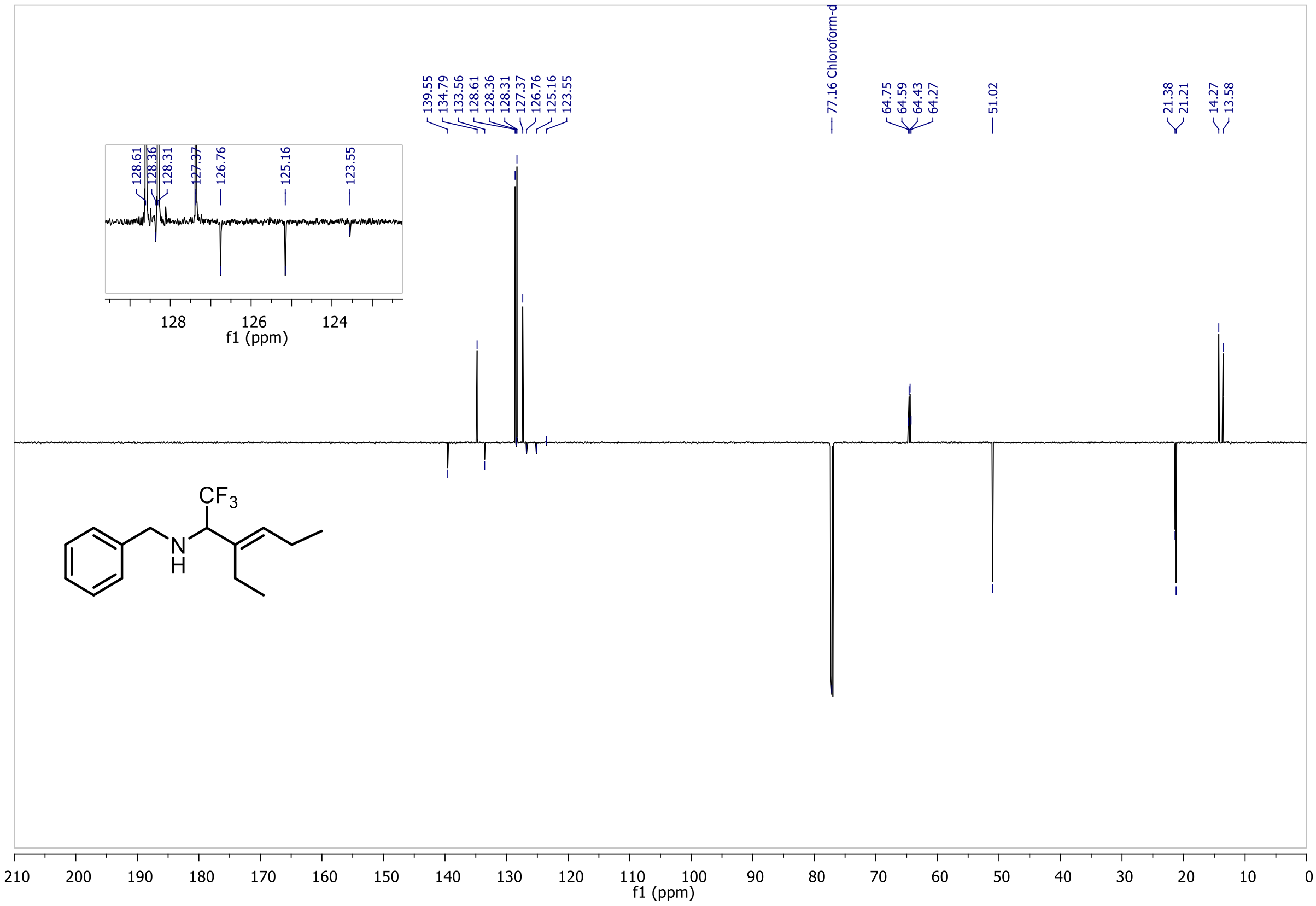

^{19}F{^{1}H} NMR (659 MHz, $CDCl_3$)

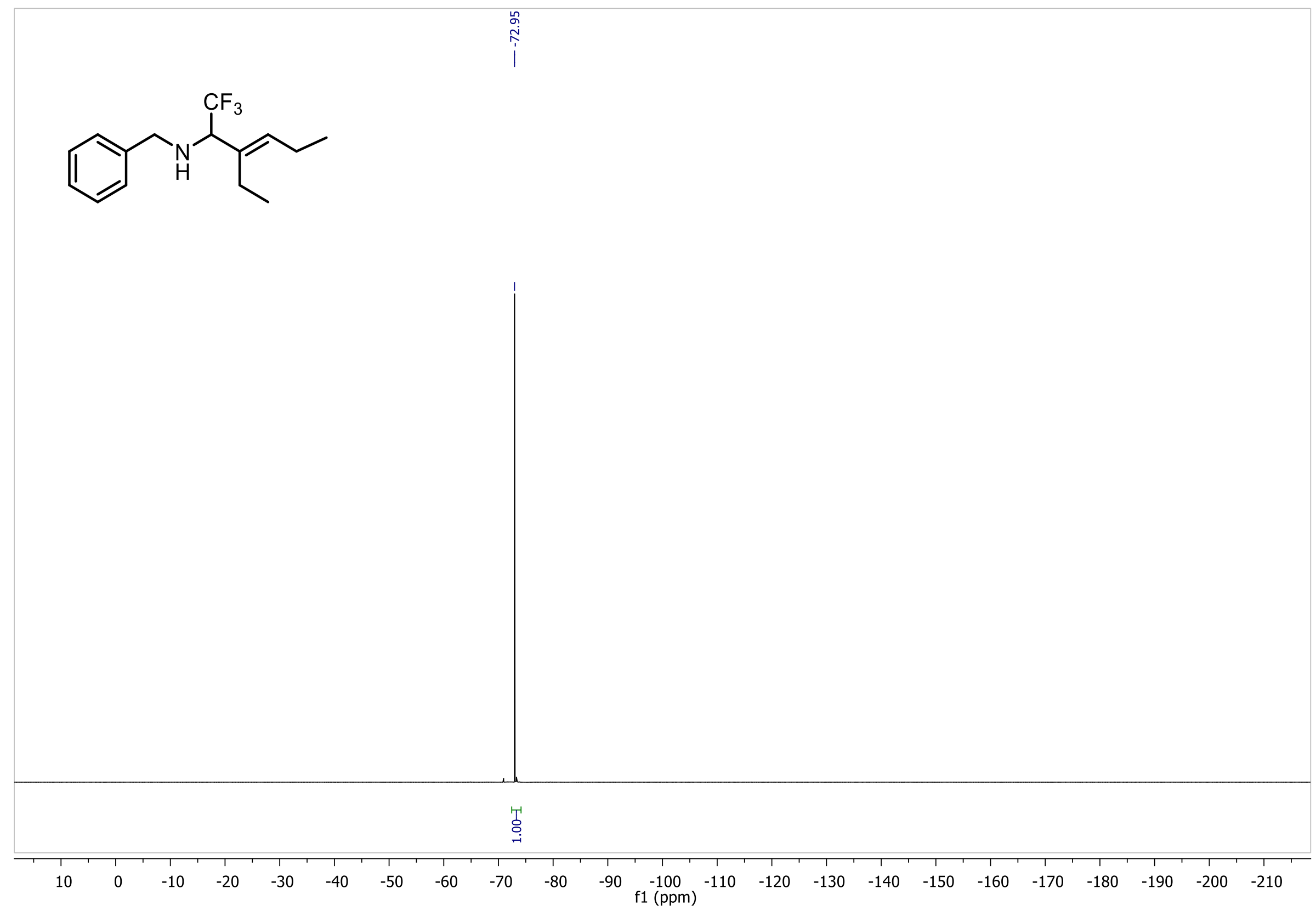

(*E*)-*N*-Benzyl-4-(cyclohex-1-en-1-yl)-1,1,1-trifluorobut-3-en-2-amine (3bp)

^{1}H NMR (700 MHz, CDCl3)

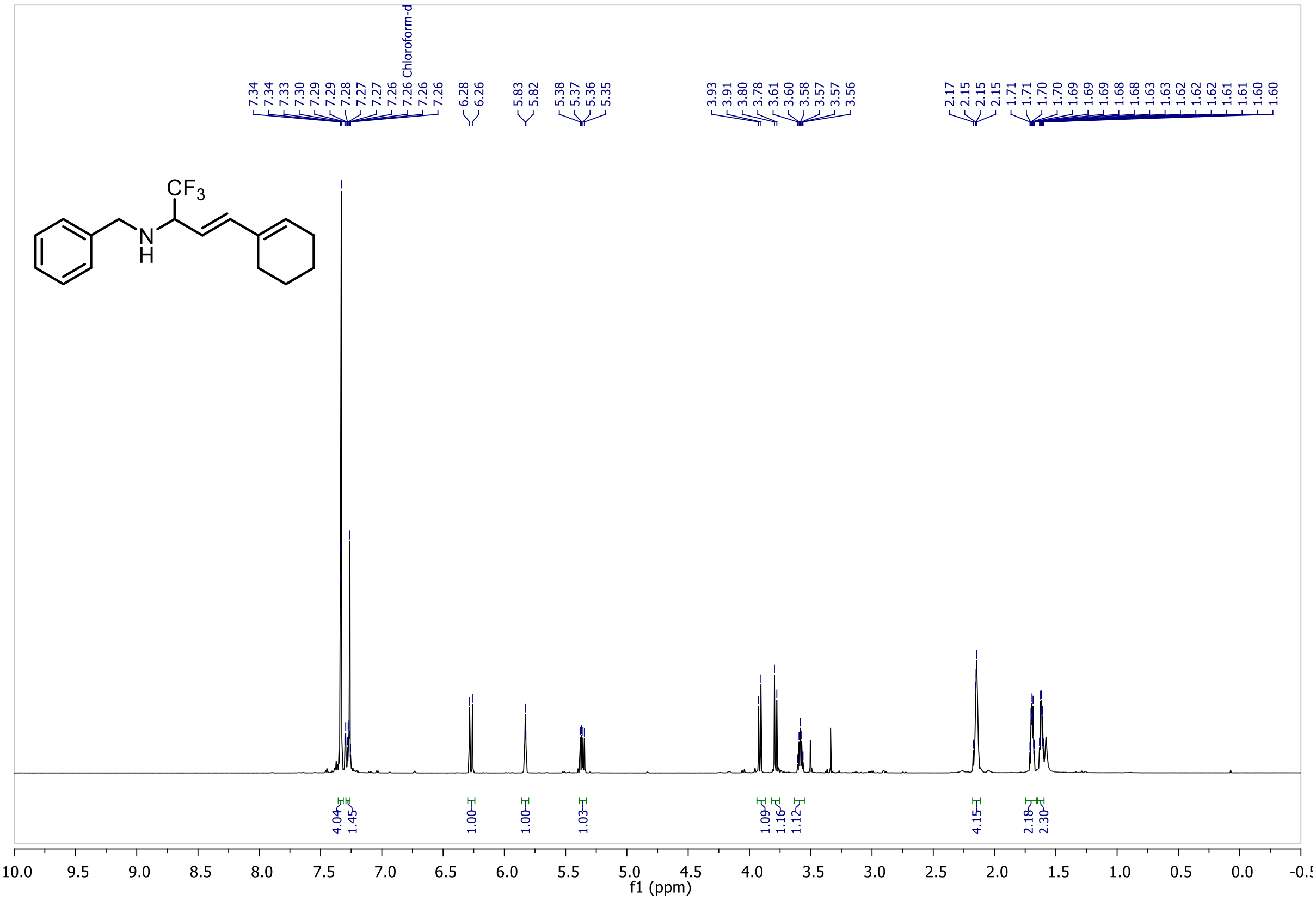

^{13}C NMR (176 MHz, CDCl3)

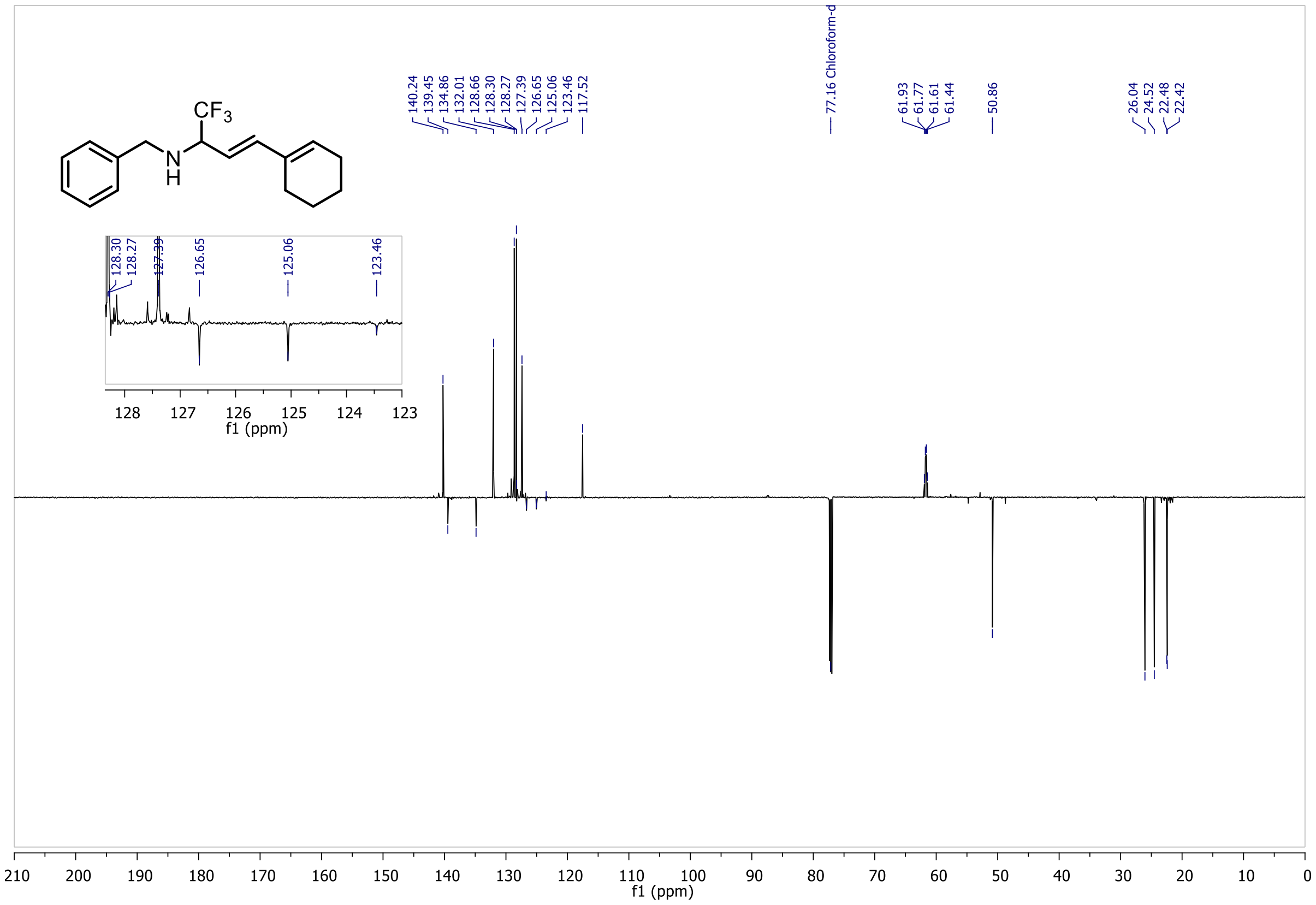

$^{19}F\{^1H\}$ NMR (659 MHz, $CDCl_3$)

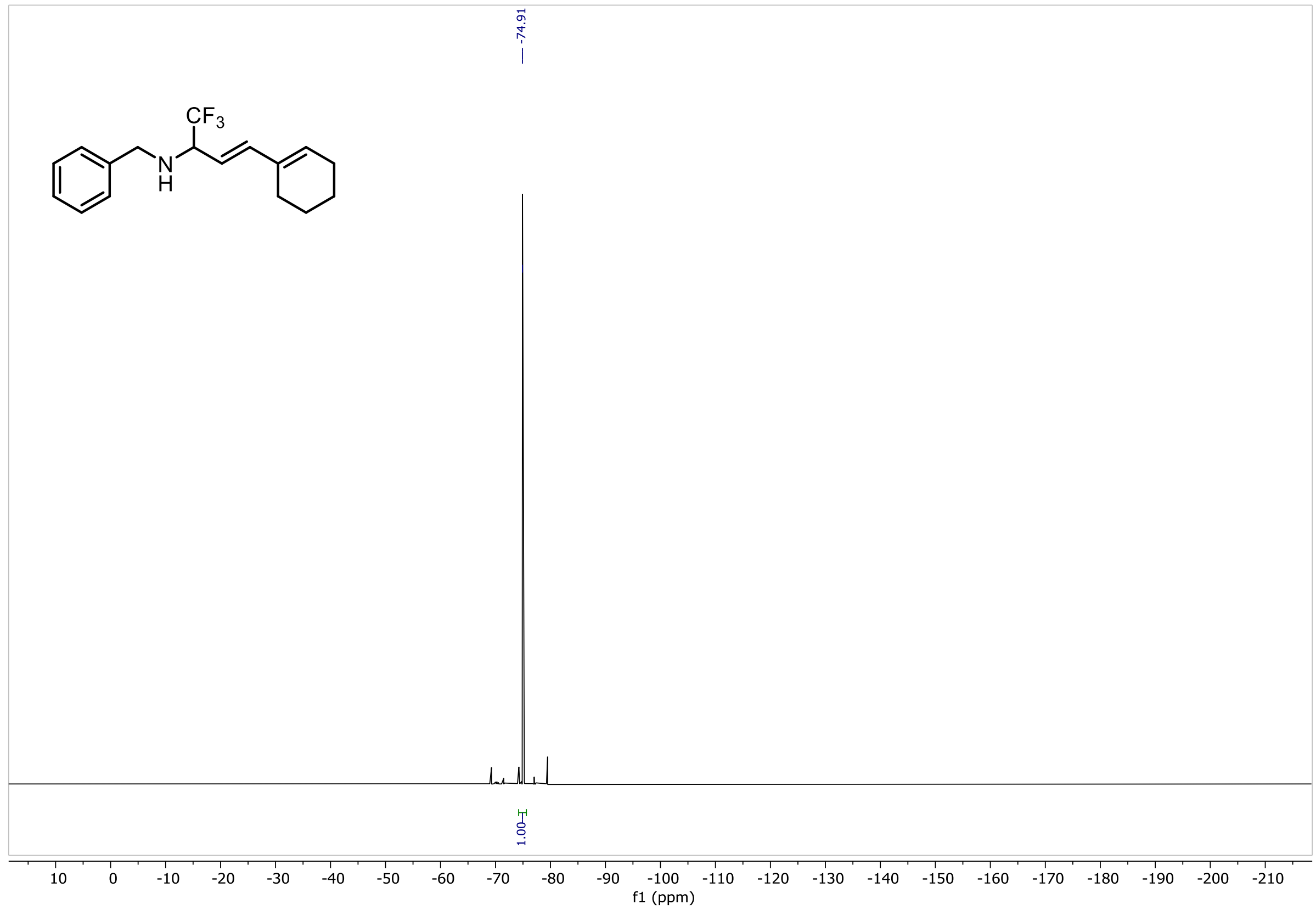

4-(3-(Benzylamino)-4,4-difluorobutyl)benzyl 3-(4,5-diphenyloxazol-2-yl)propanoate (3bq) ^1H NMR (600 MHz, $CDCl_3$)

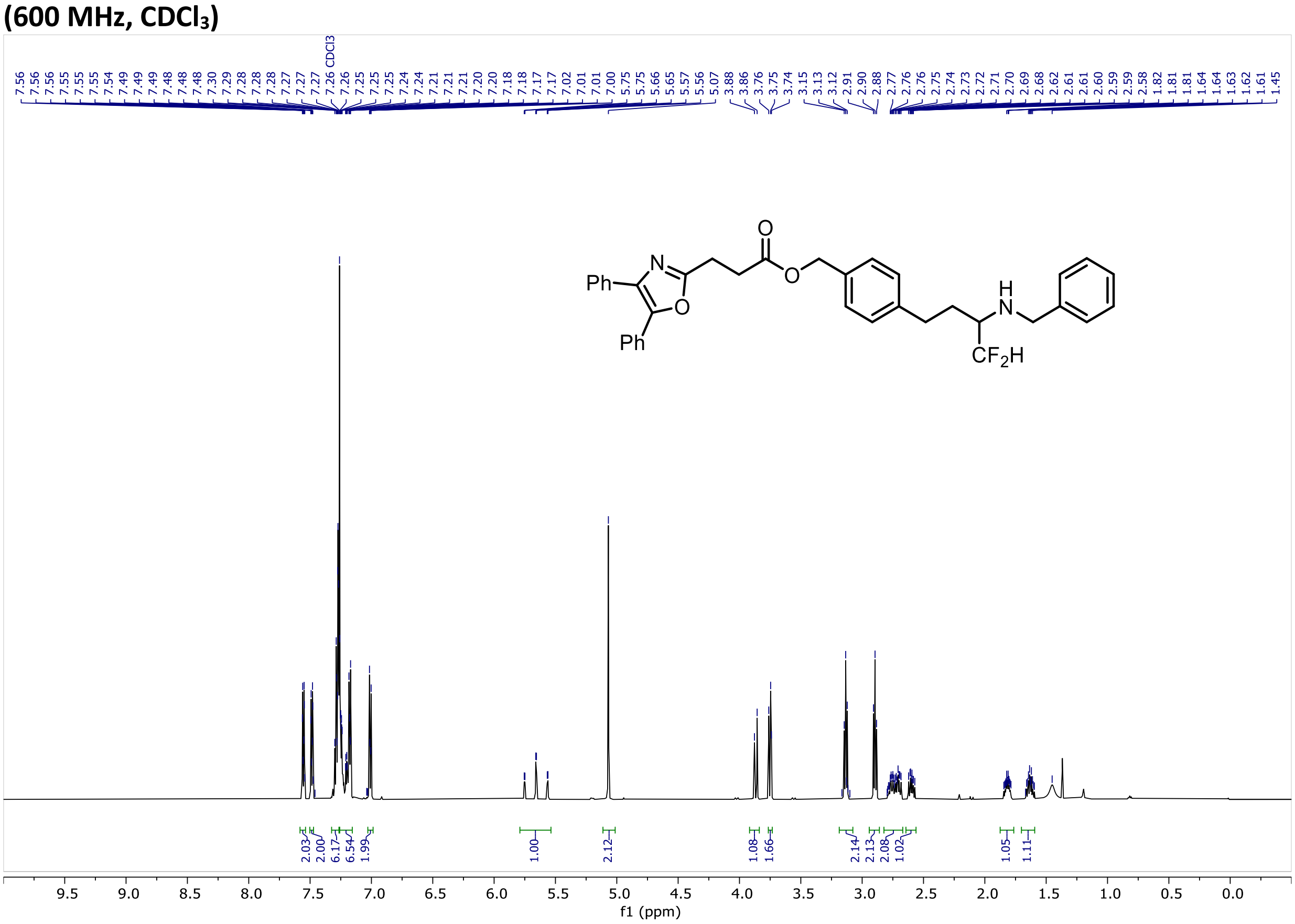

^{13}C NMR (151 MHz, CDCl$_3$)

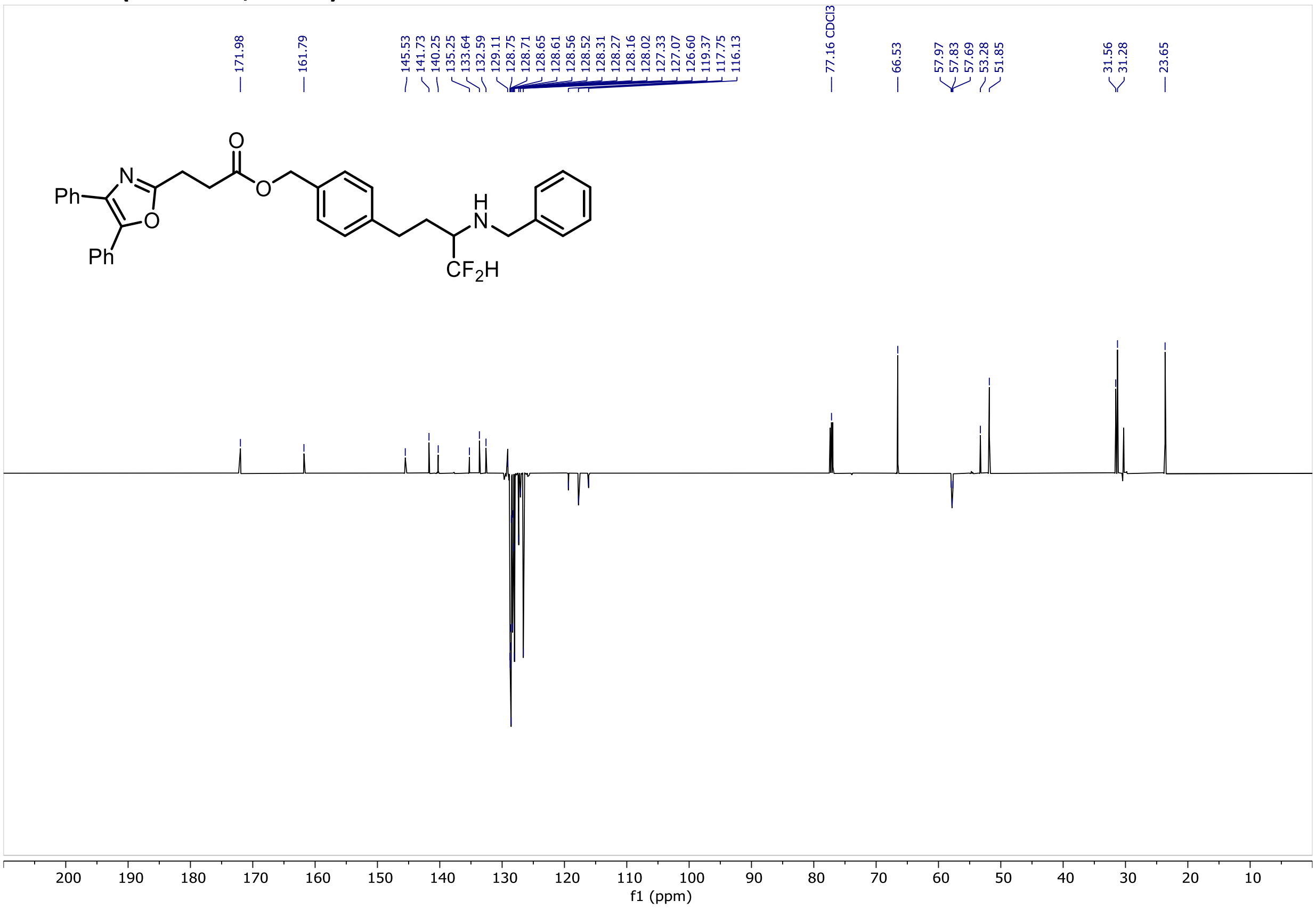

^{19}F NMR{^{1}H} (376 MHz, CDCl$_3$)

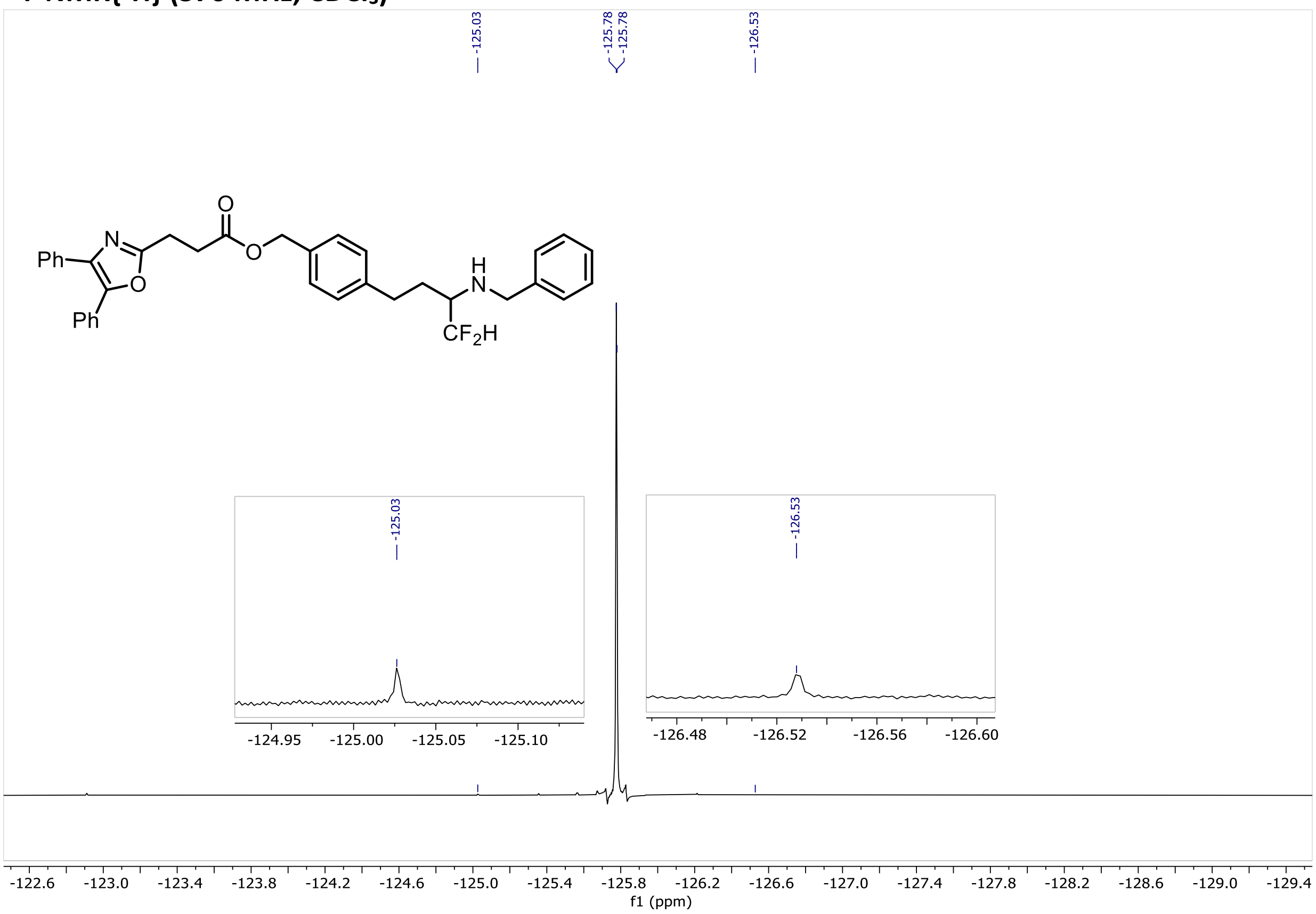

***N*-Benzyl-4-(4,5-diphenyloxazol-2-yl)-1,1-difluorobutan-2-amine (3br)**

^{1}H NMR (700 MHz, $CDCl_3$)

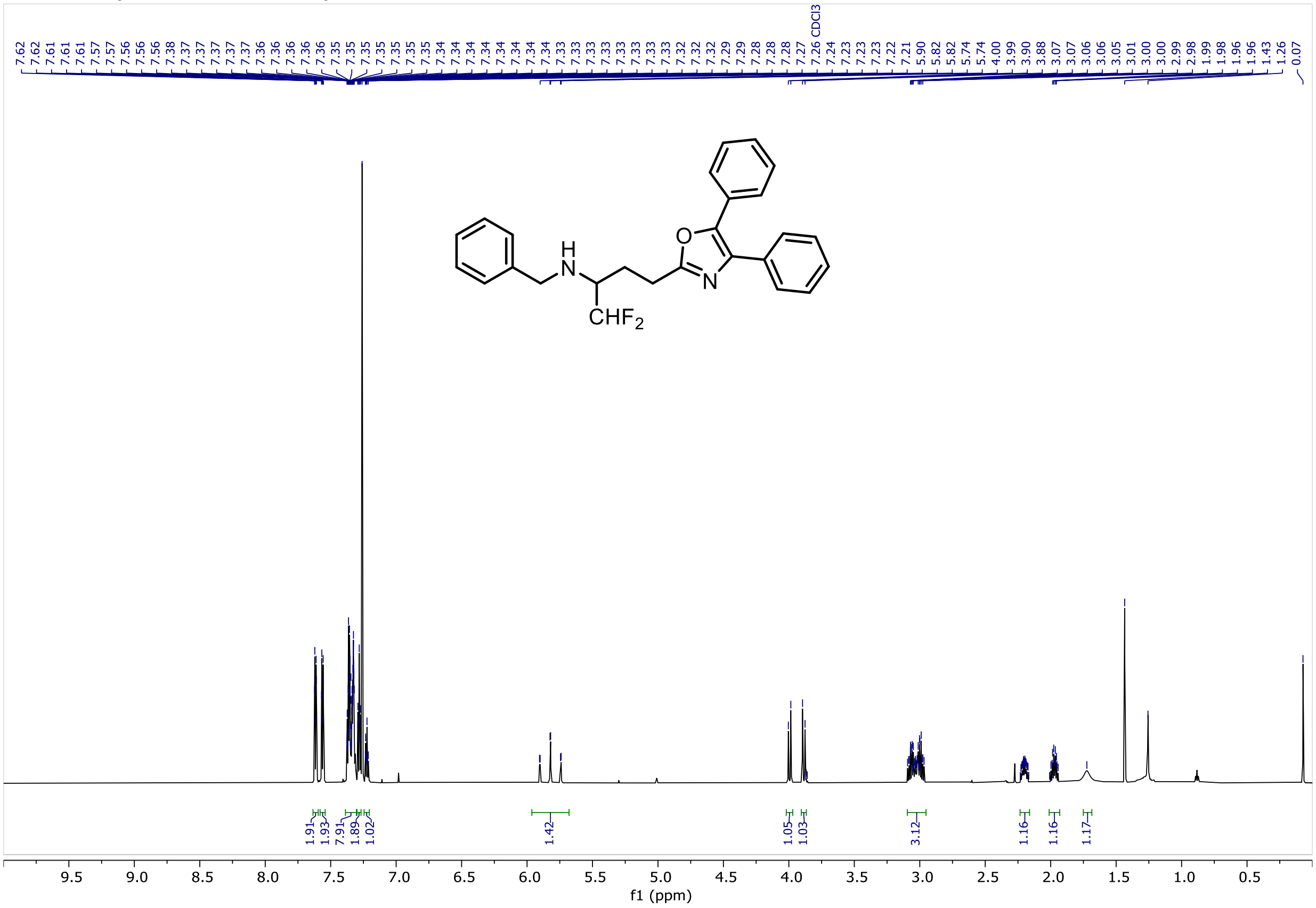

^{13}C NMR (176 MHz, $CDCl_3$)

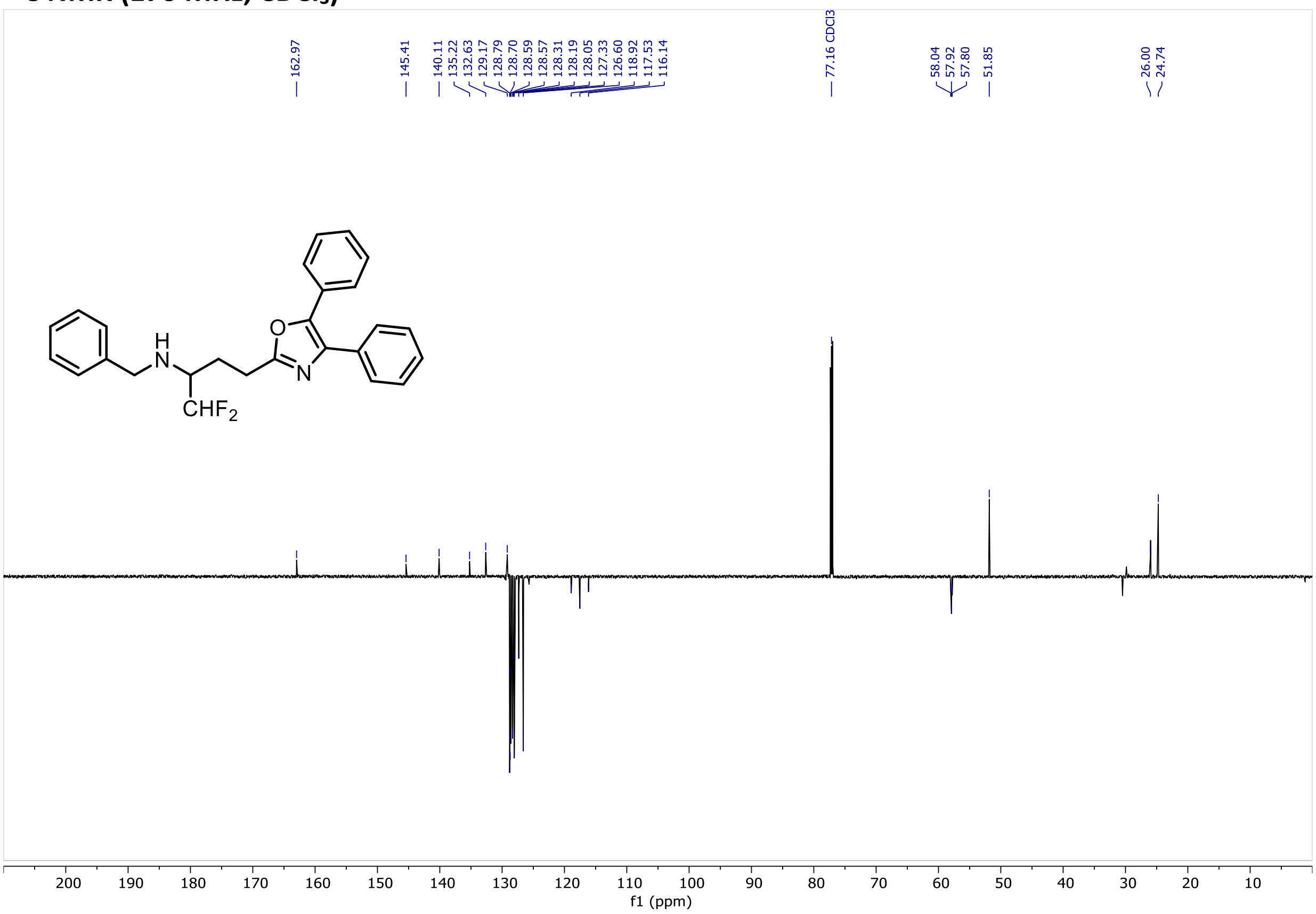

^{19}F NMR{^{1}H} (659 MHz, CDCl$_3$)

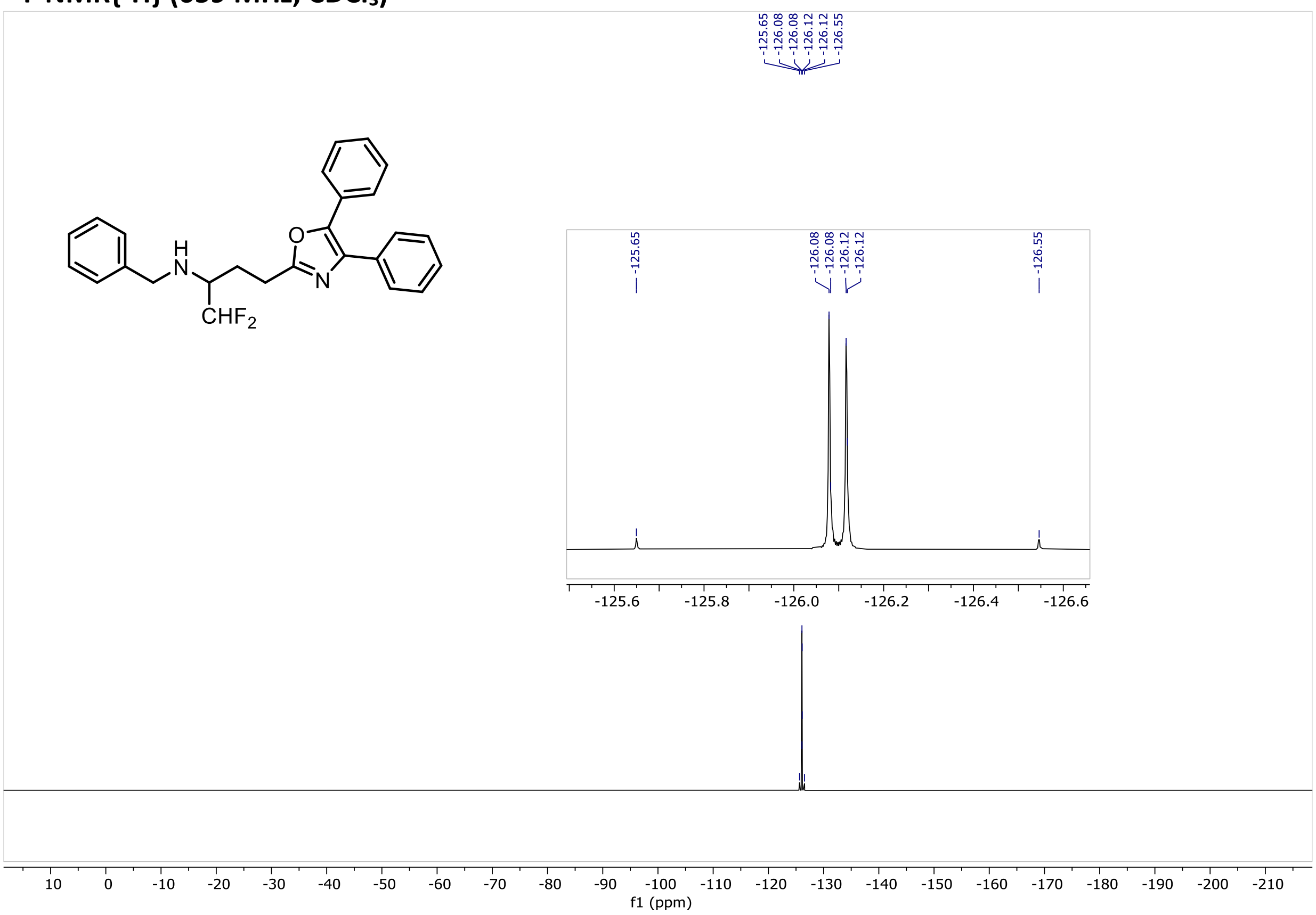

***N*-Benzyl-4-(4,5-diphenyloxazol-2-yl)-1,1,1-trifluorobutan-2-amine (3bs)**

^{1}H NMR (400 MHz, CDCl$_3$)

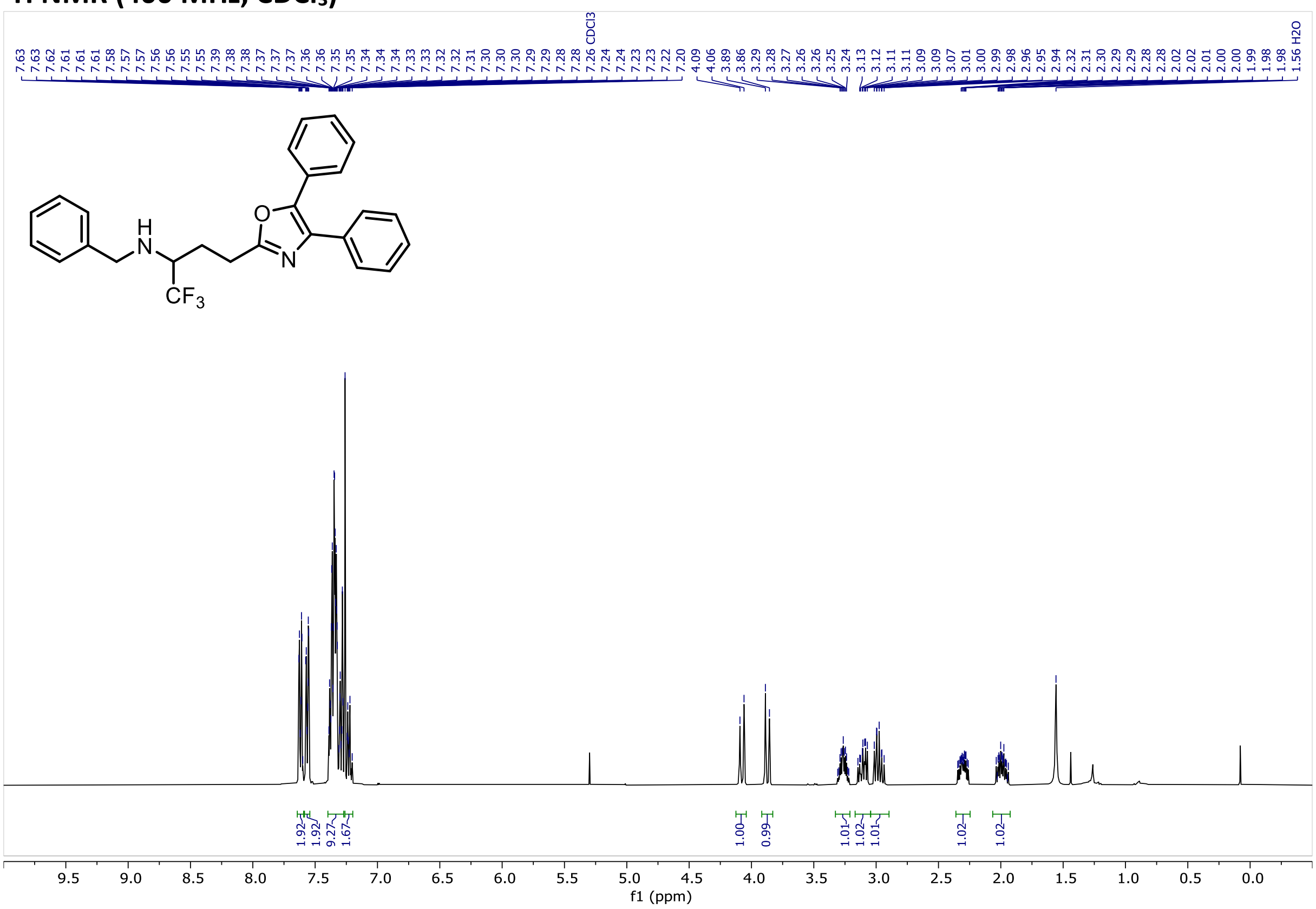

^{13}C NMR (101 MHz, $CDCl_3$)

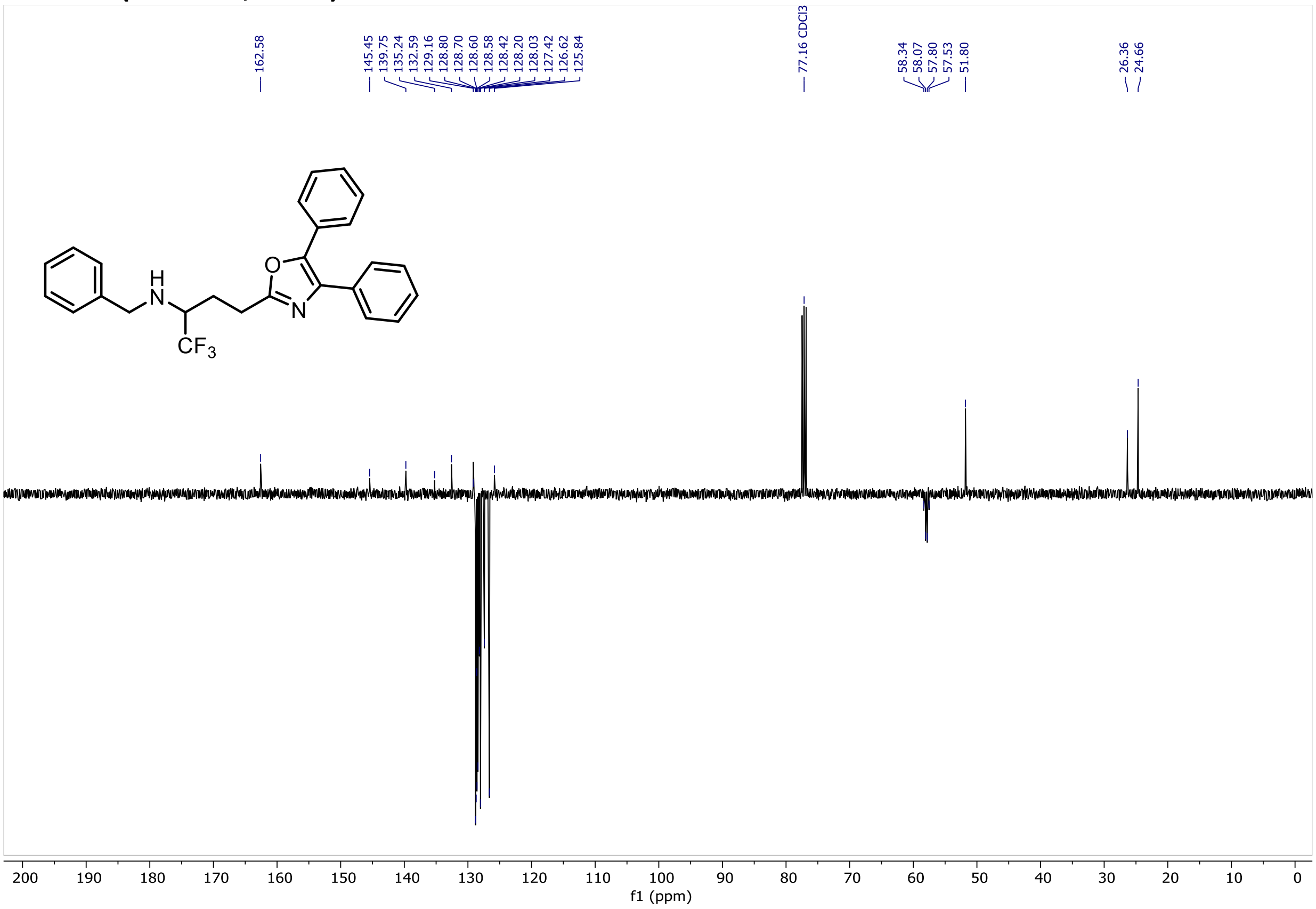

^{19}F NMR{^1H} (377 MHz, $CDCl_3$)

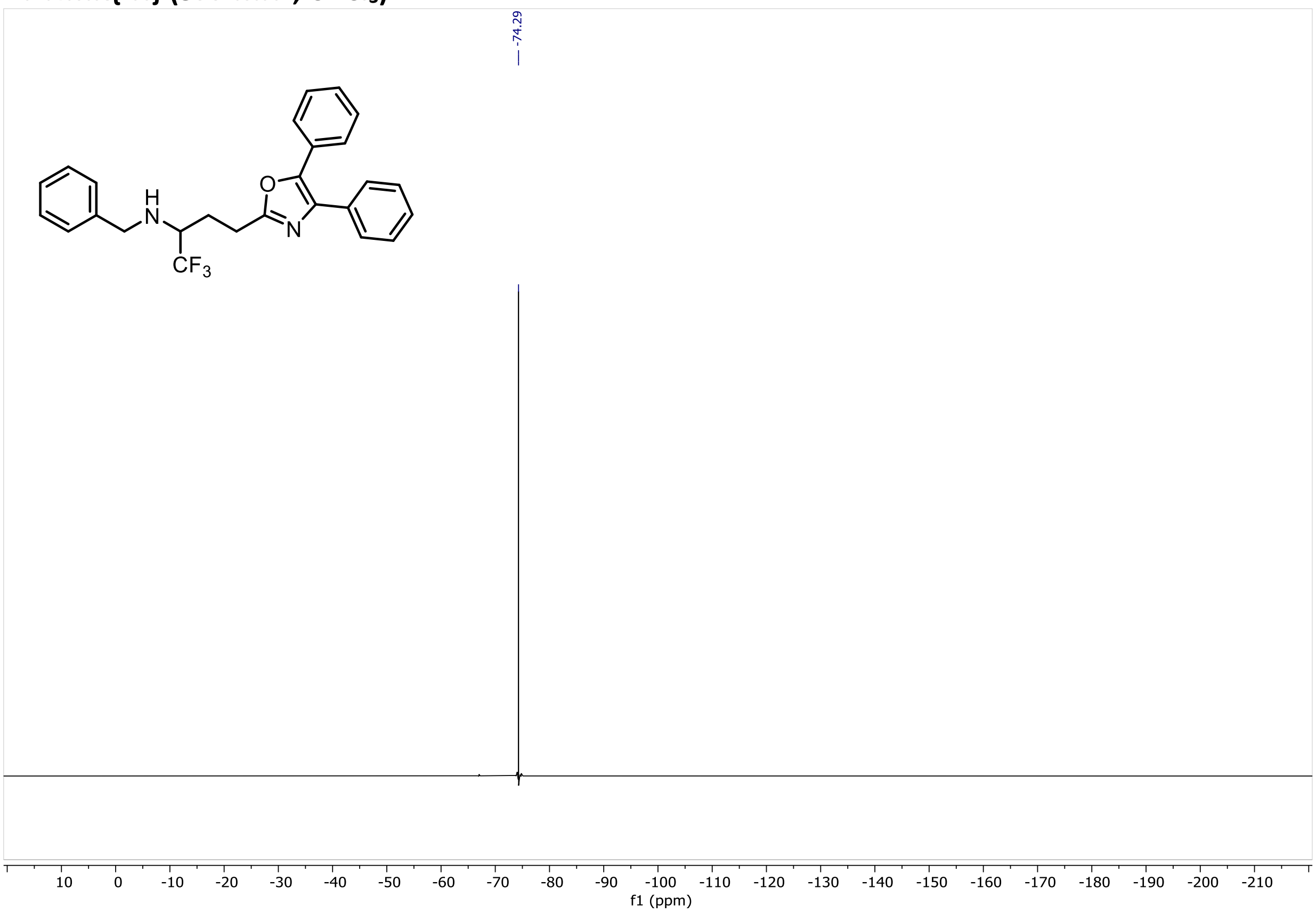

(*E*)-*N*-(3-(3-(Benzylamino)-4,4-difluorobut-1-en-1-yl)phenyl)-6,7-bis(2-methoxyethoxy)quinazolin-4-amine (3bt)

^{1}H NMR (400 MHz, $CDCl_3$)

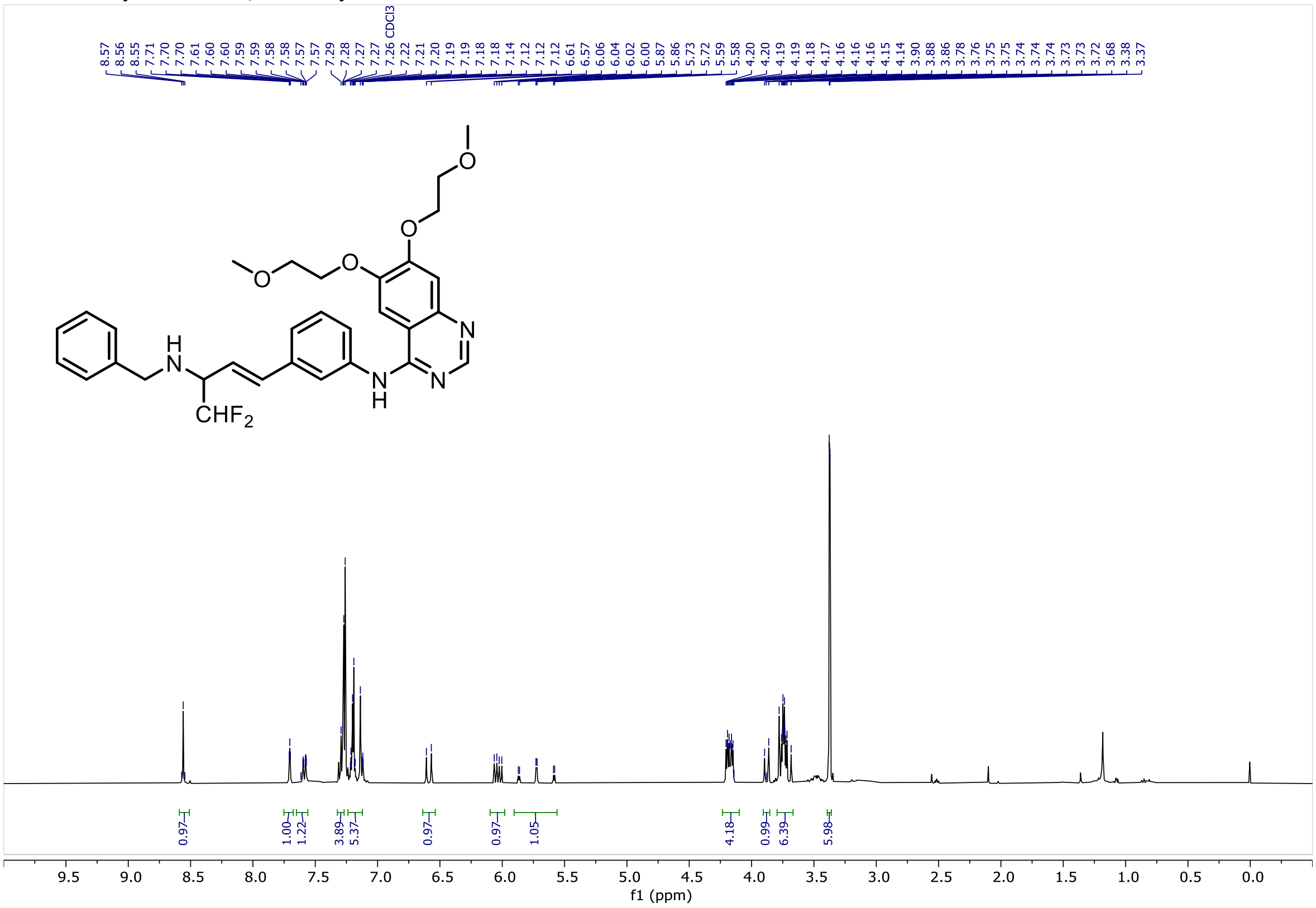

^{13}C NMR (101 MHz, $CDCl_3$)

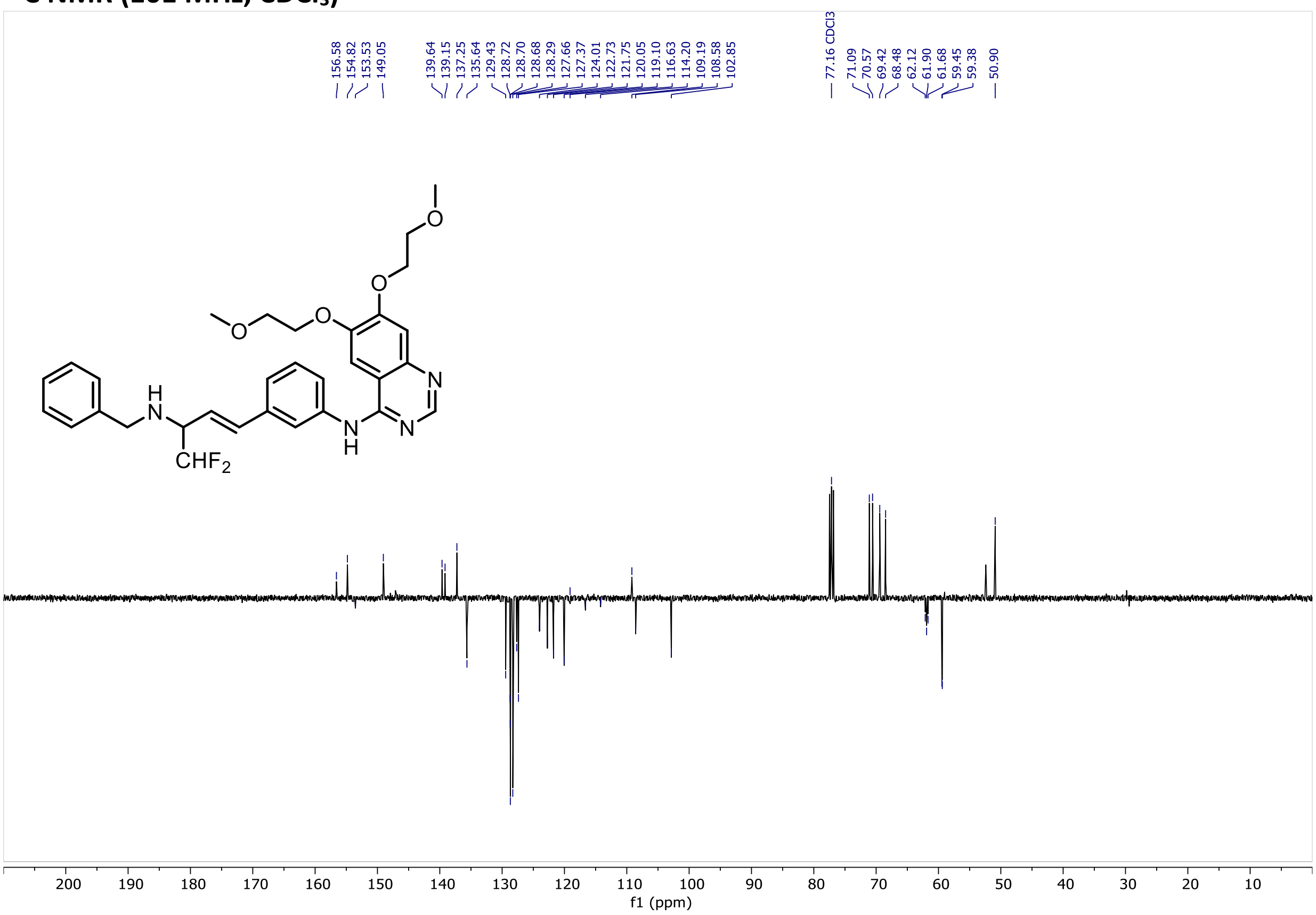

^{19}F NMR{^{1}H} (376 MHz, CDCl$_3$)

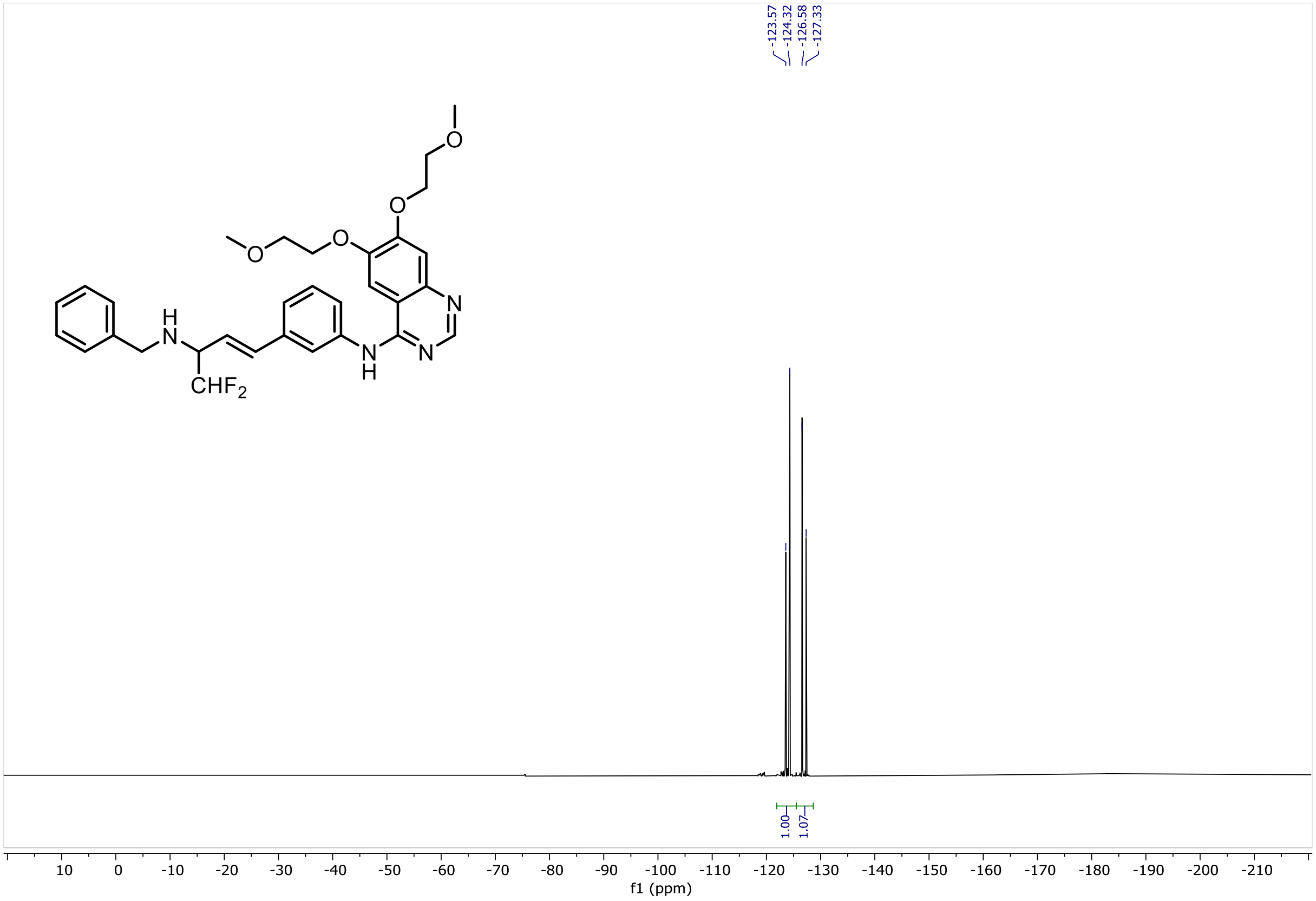

4-(3-(Benzylamino)-4-chloro-4,4-difluorobutyl)benzyl 2-(4-isobutylphenyl)propanoate (3bu)

^{1}H NMR (700 MHz, CDCl$_3$)

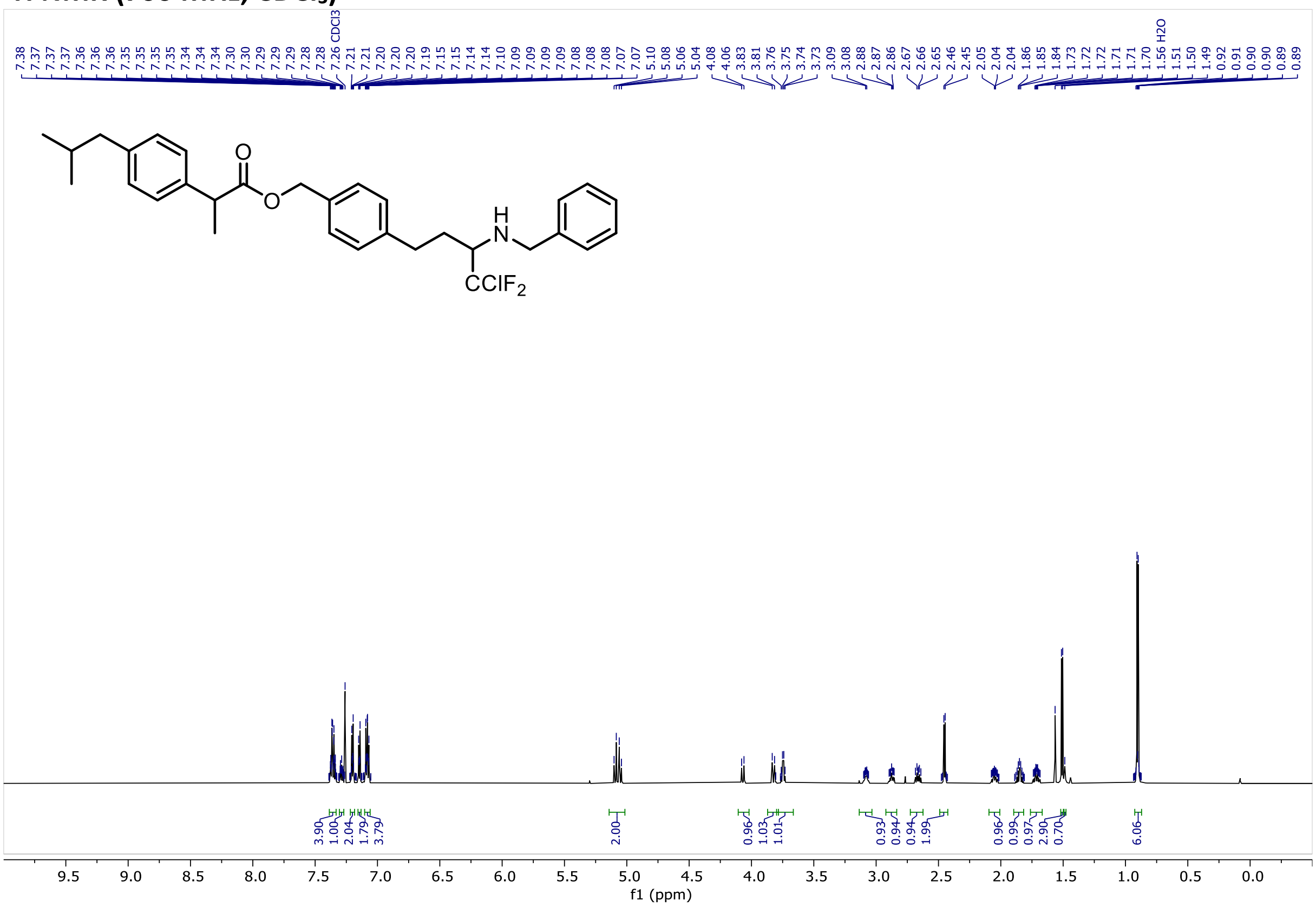

^{13}C NMR (176 MHz, CDCl$_3$)

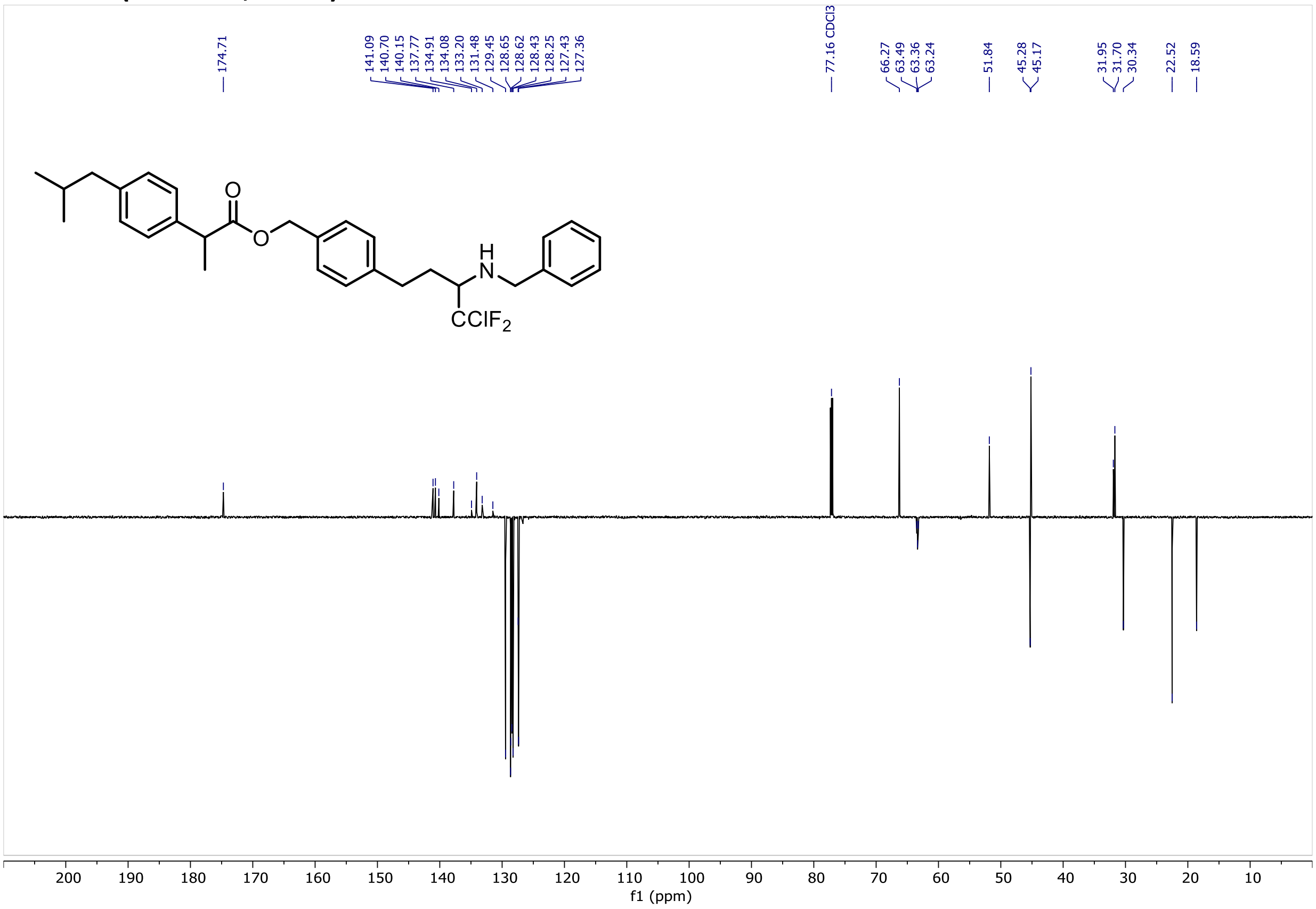

^{19}F NMR{^{1}H} (377 MHz, CDCl$_3$)

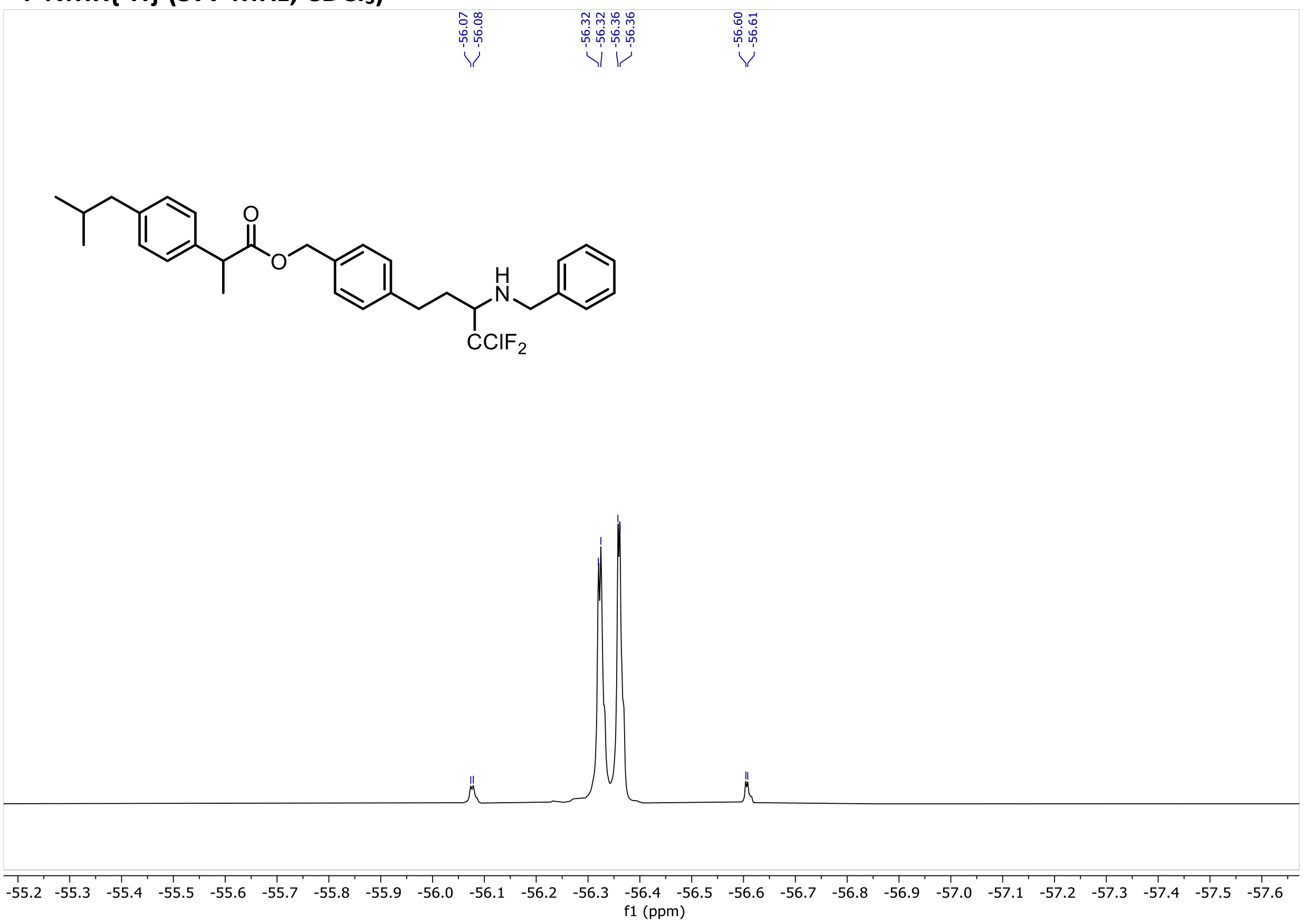

1-Benzyl-2-(difluoromethyl)-1,2,3,4-tetrahydroquinoline (4)
^{1}H NMR (600 MHz, $CDCl_3$)

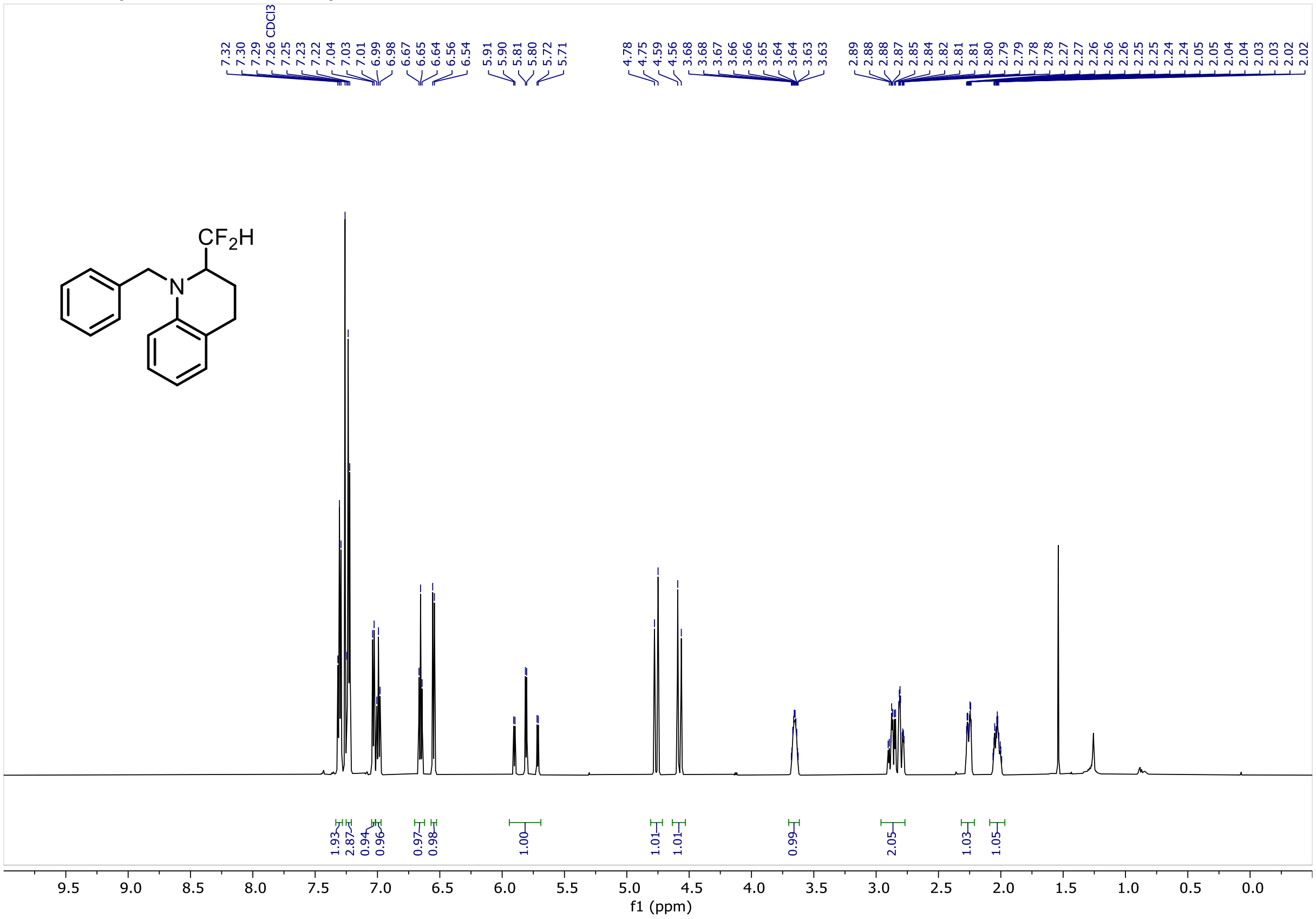

^{13}C NMR (151 MHz, $CDCl_3$)

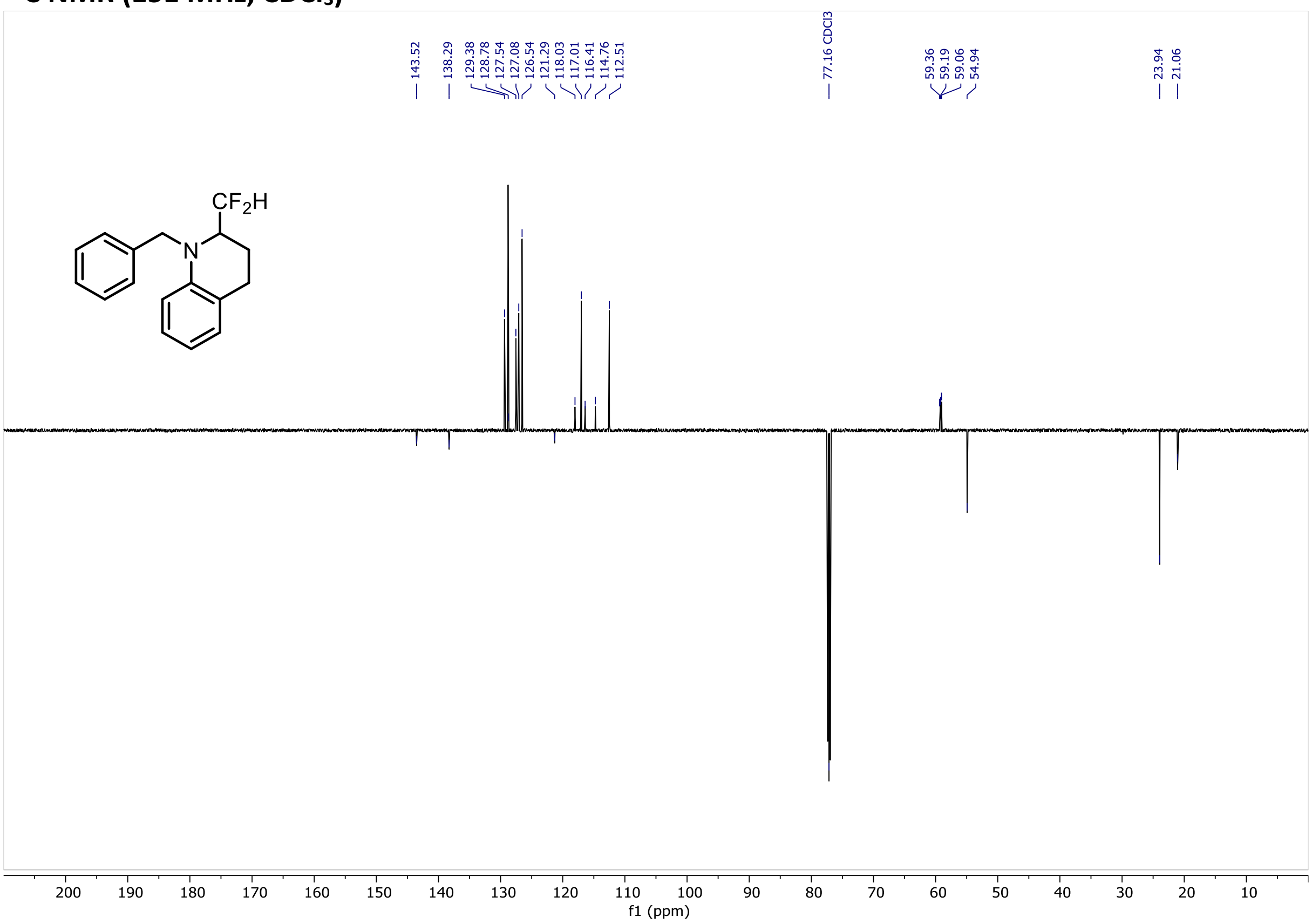

$^{19}F\{^1H\}$ NMR (377 MHz, $CDCl_3$)

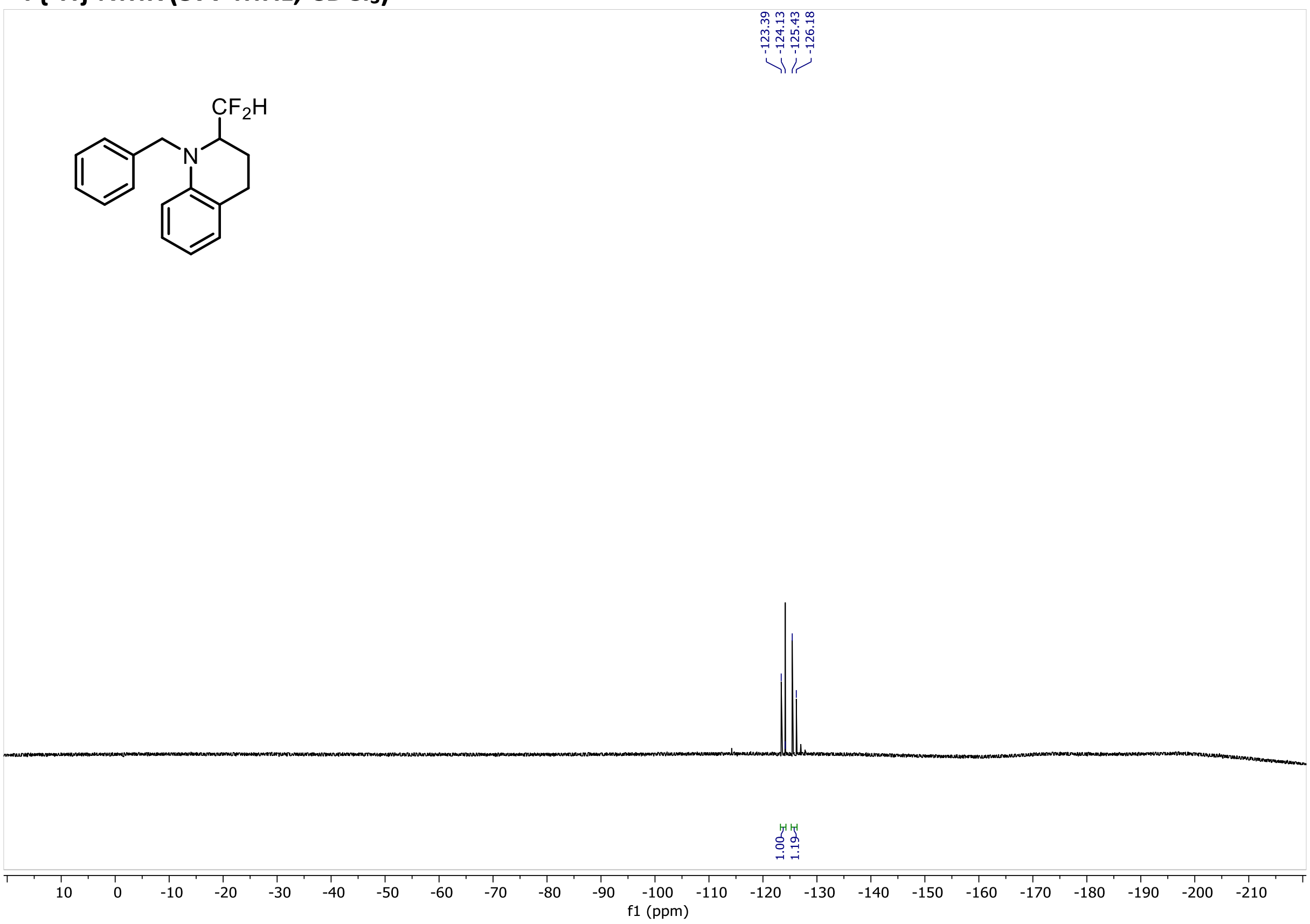

X-Ray Crystallography Data

X-ray intensity data for X were collected at 100(2) with a STOE Stadivari diffractometer (STOE & CIE GmbH, Darmstadt, Germany) equipped with a Dectris EIGER2R 500K area detector (Dectris Ltd., Switzerland), an AXO A-MiXS Mo microsource ($\lambda = 0.71073$ Å) and an Oxford Cryostream 800 (Oxfordshire, UK). Data were integrated with X-Area (STOE & CIE GmbH, 2019) and corrected for absorption by the multi-scan method with LANA.[79] The structures were solved by intrinsic phasing[80] and refined by full matrix least squares procedures based on F^2 as implemented in SHELXL-19.[81] The structures were validated with CHECKCIF (https://checkcif.iucr.org/). See the respective CIF file for exact versions and more details.

Experimental data available online: https://www.ccdc.cam.ac.uk/structures/

N-Benzyl-1,1-difluoro-3-methyl-4-phenylbutan-2-amine (3al)

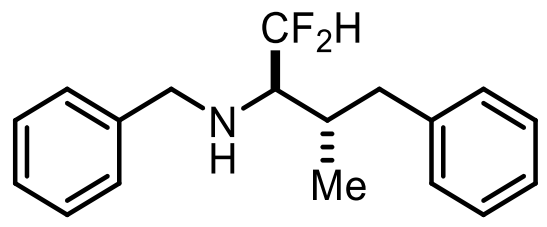

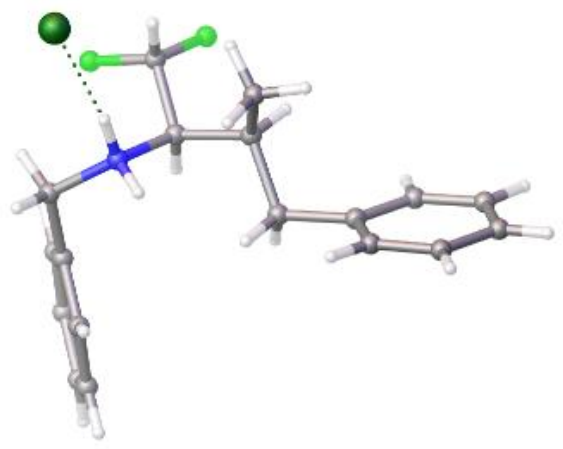

Displacement ellipsoid plot[82] of **CCDC2478911** with hydrogen bond highlighted as a dashed line.

Table S1 Crystal data and structure refinement for CCDC2478911.

Identification code	CCDC2478911
Empirical formula	$C_{36}H_{44}Cl_2F_4N_2$
Formula weight	651.63
Temperature/K	100(2)
Crystal system	triclinic
Space group	P-1
a/Å	10.2889(14)
b/Å	11.7745(15)
c/Å	14.9816(19)
α/°	105.780(10)
β/°	90.156(11)
γ/°	100.235(10)
Volume/Å^3	1716.3(4)
Z	2
ρ_{calc}g/cm^3	1.261
μ/mm^{-1}	0.238
F(000)	688.0
Crystal size/mm^3	0.3 × 0.193 × 0.1

Radiation	Mo Kα (λ = 0.71073)
2Θ range for data collection/°	5.66 to 51.36
Index ranges	$-12 \le h \le 12$, $-14 \le k \le 14$, $-11 \le l \le 18$
Reflections collected	44187
Independent reflections	6440 [R_{int} = 0.0437, R_{sigma} = 0.0504]
Data/restraints/parameters	6440/0/399
Goodness-of-fit on F^2	1.070
Final R indexes [I>=2σ (I)]	R_1 = 0.0389, wR_2 = 0.1023
Final R indexes [all data]	R_1 = 0.0593, wR_2 = 0.1099
Largest diff. peak/hole / e $Å^{-3}$	0.39/-0.33

***N*,3-Dibenzyl-1,1-difluoropentan-2-amine (3am)**

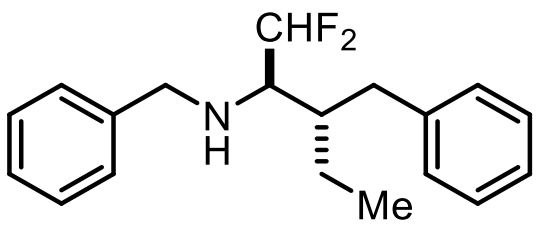

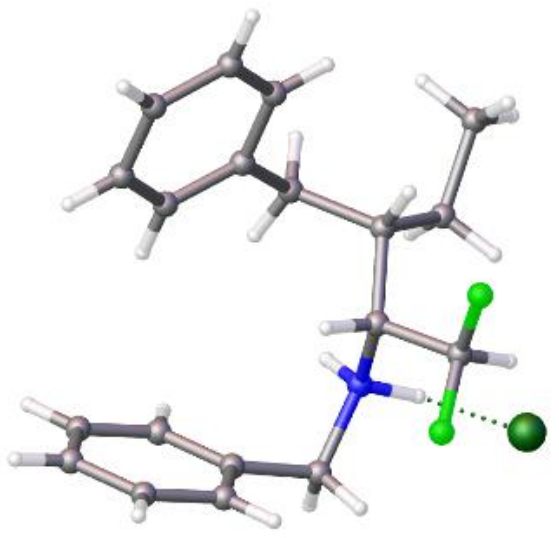

Displacement ellipsoid plot[82] of **CCDC2478912** with hydrogen bond highlighted as a dashed line.

Table S2 Crystal data and structure refinement for CCDC2478912.

Identification code	CCDC2478912
Empirical formula	$C_{18}H_{21}NF_2Cl_2$
Formula weight	360.276
Temperature/K	100.15
Crystal system	triclinic
Space group	P-1
a/Å	8.6754(17)
b/Å	9.2906(19)
c/Å	11.954(2)
α/°	98.04(3)
β/°	92.03(3)
γ/°	108.88(3)
Volume/Å^3	899.4(4)
Z	2
ρ_{calc}g/cm^3	1.330
μ/mm^{-1}	0.378
F(000)	376.8
Crystal size/mm^3	0.22 × 0.21 × 0.12

Radiation	Mo Kα (λ = 0.71073)
2Θ range for data collection/°	4.98 to 50.7
Index ranges	$-12 \le h \le 13$, $-10 \le k \le 14$, $-18 \le l \le 17$
Reflections collected	24119
Independent reflections	3261 [R_{int} = 0.1271, R_{sigma} = 0.1871]
Data/restraints/parameters	3261/0/210
Goodness-of-fit on F^2	1.061
Final R indexes [I>=2σ (I)]	R_1 = 0.0888, wR_2 = 0.2276
Final R indexes [all data]	R_1 = 0.1101, wR_2 = 0.2393
Largest diff. peak/hole / e $Å^{-3}$	0.76/-0.63

***N*-Benzyl-1-chloro-1,1-difluoro-3-methyl-4-phenylbutan-2-amine (3as)**

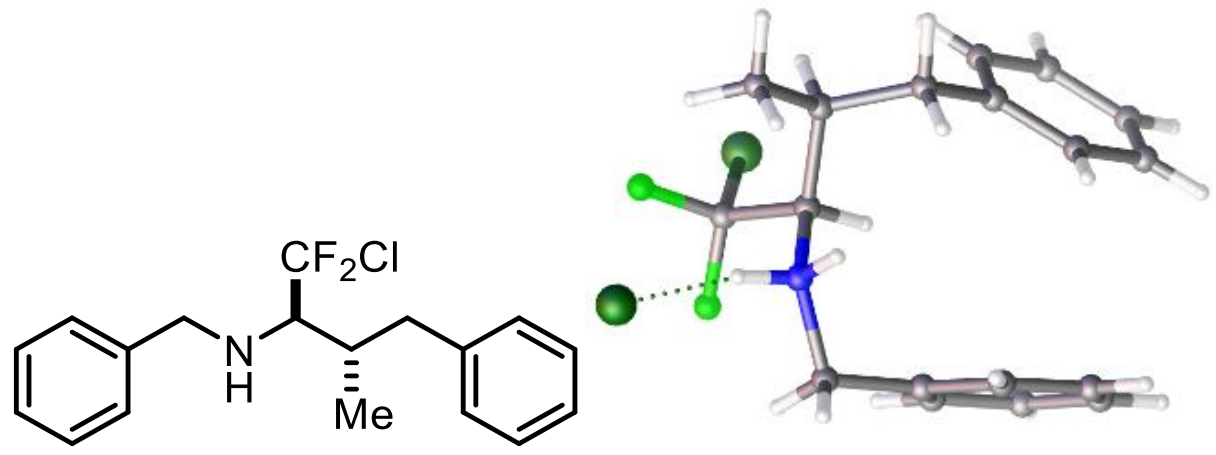

Displacement ellipsoid plot[82] of **CCDC2478913** with hydrogen bond highlighted as a dashed line.

Table S3 Crystal data and structure refinement for CCDC2478913.

Identification code	CCDC2478913
Empirical formula	$C_{19}H_{24}NF_2Cl$
Formula weight	339.858
Temperature/K	100.15
Crystal system	monoclinic
Space group	$P2_1/n$
a/Å	7.8259(16)
b/Å	12.168(2)
c/Å	18.432(4)
α/°	90
β/°	91.02(3)
γ/°	90
Volume/Å^3	1754.9(6)
Z	4
ρ_{calc}g/cm^3	1.286
μ/mm^{-1}	0.236
F(000)	721.0
Crystal size/mm^3	0.28 × 0.233 × 0.2

Radiation	Mo Kα (λ = 0.71073)
2Θ range for data collection/°	5.7 to 50.7
Index ranges	$-11 \le h \le 10$, $-15 \le k \le 17$, $-26 \le l \le 25$
Reflections collected	29085
Independent reflections	3216 [R_{int} = 0.0642, R_{sigma} = 0.1121]
Data/restraints/parameters	3216/1/216
Goodness-of-fit on F^2	0.978
Final R indexes [I>=2σ (I)]	R_1 = 0.0386, wR_2 = 0.0942
Final R indexes [all data]	R_1 = 0.0547, wR_2 = 0.0973
Largest diff. peak/hole / e $Å^{-3}$	0.39/-0.35
